# Microarray Profile of Long Noncoding RNA and Messenger RNA Expression in a Model of Alzheimer’s Disease

**DOI:** 10.3390/life10050064

**Published:** 2020-05-14

**Authors:** Linlin Wang, Li Zeng, Hailun Jiang, Zhuorong Li, Rui Liu

**Affiliations:** Institute of Medicinal Biotechnology, Chinese Academy of Medical Sciences and Peking Union Medical College, Beijing 100050, China; wanglinlin@wfmc.edu.cn (L.W.); zengsheng@imb.pumc.edu.cn (L.Z.); jianghailun@imb.pumc.edu.cn (H.J.)

**Keywords:** Alzheimer’s disease, long noncoding RNA, messenger RNA, microRNA, gene network

## Abstract

Alzheimer’s disease (AD) is a progressive neurodegenerative disease characterized by a deficiency in cognitive skills. Although long noncoding RNAs (lncRNAs) have been proposed as associated with AD, the aberrant lncRNAs expression and the co-expression of lncRNAs-mRNAs network in AD remains unclear. Therefore, in this study, lncRNA microarray was performed on the brain of APP/PS1 mice at different age, widely used as an AD mouse model, and on age-matched wide-type controls. Our results identified a total of 3306 lncRNAs and 2458 mRNAs as aberrantly expressed among AD mice at different age and their age-matched control. Gene Ontology and pathway analysis of the AD-related lncRNAs and mRNAs indicated that neuroinflammation-related and synaptic transmission signaling pathways represented the main enriched pathways. An lncRNA–mRNA–miRNA network between the differentially expressed transcripts was constructed. Moreover, an mRNA–miRNA network between both significantly dysregulated and highly conserved genes was also constructed, and among this network, the IGF1, P2RX7, TSPO, SERPINE1, EGFR, HMOX1, and NFE212 genes were predicted to play a role in the development of AD. In conclusion, this study illustrated the prognostic value of lncRNAs and mRNAs associated to AD pathology by microarray analysis and might provide potential novel biomarkers in the diagnosis and treatment of AD.

## 1. Introduction

Alzheimer’s disease (AD) is a neurodegenerative disease characterized by the accumulation of amyloid-β (Aβ), neurofibrillary tangles consisting of a phosphorylated Tau protein, and neuronal loss [1,2,3,4]. Most types of dementia that affect adults over 65 years of age are caused by AD, and AD patients gradually lose their ability to communicate and self-care as the disease progresses [5]. Nonetheless, the mechanisms underlying AD are poorly understood and currently no effective drugs or treatments are available to prevent the progress of the disease. Since AD is characterized by a complex genetic architecture with a high expression of amyloid precursor protein (APP), presenilin-1 (PS1), and other genes, it is of pivotal importance to elucidate the changes in the genes mediating the development of AD [2,6,7].

Long noncoding RNAs (lncRNAs) are RNAs are more than 200 nucleotides (nt) long, with little or no transcription activity [8,9]. Despite the lack of transcription function, lncRNAs are involved in the mechanism of regulation of gene expression and essential biological processes [10]. In addition to the epigenetic targeting, lncRNAs participate in mRNA processing, stability, splicing, and degradation [11]. More recently, lncRNAs have been proposed as closely connected with AD. Certain lncRNAs, such as BACE1-AS [12], BC200 [13], 51A [14], 17A [15], NDM29 [16], and NAT-Rad18 [17], have been identified in human brain tissues with AD. The lncRNA, β-site APP cleaving enzyme 1-antisense strand (BACE1-AS), regulates the expression of BACE1 mRNA by the formation of an RNA duplex, which can increase APP processing, Aβ overproduction, and plaque deposition [12]. lncRNA BC200, a neuron-specific non-coding RNA, causes inadequate RNA delivery to the synapses by abnormally depressing fragile X mental retardation 1 protein (FMR1) mRNA translation and results in neurodegenerative processes that lead to AD [13,18]. Some of the lncRNAs mentioned above such as 51A, 17A, NDM29, and NAT-Rad18 work through the translation repression of the corresponding targeted mRNAs [15,16,17]. Although these discoveries led to some novel insights into AD pathology, the current knowledge on the aberrant expression and pathophysiological function of lncRNAs, as well as the potential co-expression of lncRNAs-mRNAs network in AD, is far from clear.

The present study focused on the investigation of the abnormal expression of lncRNAs and associated mRNAs underlying AD development. Microarray analysis of 1-month-old, 3-month-old, 6-month-old, and 9-month-old digenic APP/PS1 mice on a C57Bl/6 J background were employed, which express a chimeric mouse/human APP bearing the Swedish mutation (K595N/M596L) and the PS1 protein with a deletion on exon 9, recognized as a routinely used mouse model of familial AD for investigating AD-associated pathogenesis [19], together with matched wild-type (WT) controls. The Gene Ontology (GO), the Kyoto Encyclopedia of Genes and Genomes (KEGG), and the co-expression of lncRNAs-mRNAs network were analyzed.

## 2. Materials and Methods

### 2.1. Animal and Tissue Preparation

Heterozygous APP/PS1 transgenic founder mice and age-matched WT littermates were purchased from the Jackson Laboratory (Bar Harbor, ME, USA). In this study, 1-month-old, 3-month-old, 6-month-old, and 9-month-old APP/PS1 mice and their respective age-matched WT control mice were chosen to accomplish microarray analysis. Each group contains one male and two females. All mice had access to food and water ad libitum and were kept in an environment with constant temperature and humidity according to the Guide for the Care and Use of Laboratory Animals. The mice were then sacrificed by cervical dislocation and their brains were quickly removed and preserved by flash freezing in liquid nitrogen. The experiment was approved by the ethical committee of the Institute of Medicinal Biotechnology (IMB-201808-D8).

### 2.2. RNA Extraction

The total RNA was extracted from the brain tissues using Invitrogen’s Trizol reagent (Thermo Fisher Scientific, Waltham, MA, USA). Subsequently, 0.2 mL chloroform was added per 1 mL Trizol Reagent and the aqueous phase was transferred into a fresh tube. An amount of 0.5 mL isopropyl alcohol was used per 1 mL Trizol Reagent for RNA precipitation followed by RNA washing with 1 mL of 75% ethanol. The RNA pellet was air-dried for 5–10 min, then dissolved in RNase-free water by pipetting, and incubated for 10 min at a temperature of 55 to 60 °C. The purity of the RNA samples was measured by the ratios of A260/280 and A260/230, with acceptable values ranging from 1.8 to 2.2 and 2.0 to 2.4, respectively.

### 2.3. RNA Labeling and Array Hybridization

The array experiment was performed using the Agilent Mouse LncRNA Microarray technology according to the manufacturer’s instructions (Agilent Technologies, Santa Clara, CA, USA). In brief, the Quick Amp Labeling Kit (Agilent Technologies, Santa Clara, CA, USA) was used to label the Cyanine-3-CTP in the cDNA synthesized from total RNA. Then, the labeled cDNA was purified by RNeasy Mini Kit (QIAGEN GmBH, Düsseldorf, Germany) and hybridized onto the lncRNA microarray using the Agilent Gene Expression Hybridization Kit (Agilent Technologies, Santa Clara, CA, USA). After washing, the Agilent Microarray Scanner was employed to scan the raw data of the microarray, and the Agilent Feature Extraction Software was used to extract the resulting data (Agilent Technologies, Santa Clara, CA, USA).

### 2.4. GO and Pathway Analysis

GO analysis [20] involving biological processes, cellular components, and molecular functions, was used to identify the function of the differentially expressed lncRNAs and mRNAs in the APP/PS1 mouse model. KEGG analysis [21] was used to determine the biological pathways of AD-related lncRNAs and mRNAs obtained in the microarray experiment. A False Discovery Rate (FDR) < 0.05 was considered statistically significant.

### 2.5. lncRNA–mRNA–miRNA Co-Expression Network Construction

In order to draw a network between significantly changed lncRNA, mRNA, and miRNA, the Pearson correlation coefficient (PCC) statistic measurement was employed to calculate each differently expressed lncRNA–mRNA–miRNA triplet. A PCC value greater than 0.99 was considered statistically significant. According to the selected conservative genes, the relevant miRNA target prediction was performed, including miRWalk [22], TargetScan [23], miRBase [24], and miRanda [25]. Based on the functional annotation of miRNA target genes and miRNA-mRNA-targeted relationship, an integrated diagram of mRNA–miRNA regulatory network was constructed.

### 2.6. Statistical Analysis

Raw count data were analyzed using the Empirical Analysis of Digital Gene Expression Data in R (edgeR). Replicate probes were averaged. A Fold Change ≥ 2.0 and a *p*-value ≤ 0.05 were chosen to distinguish the differentially expressed genes that were significant between the two groups. Finally, a hierarchical clustering was performed to show the distinguishable gene expression profiles among samples.

## 3. Results

### 3.1. Different Expression Profile of lncRNA and mRNA

**Figure 1:**
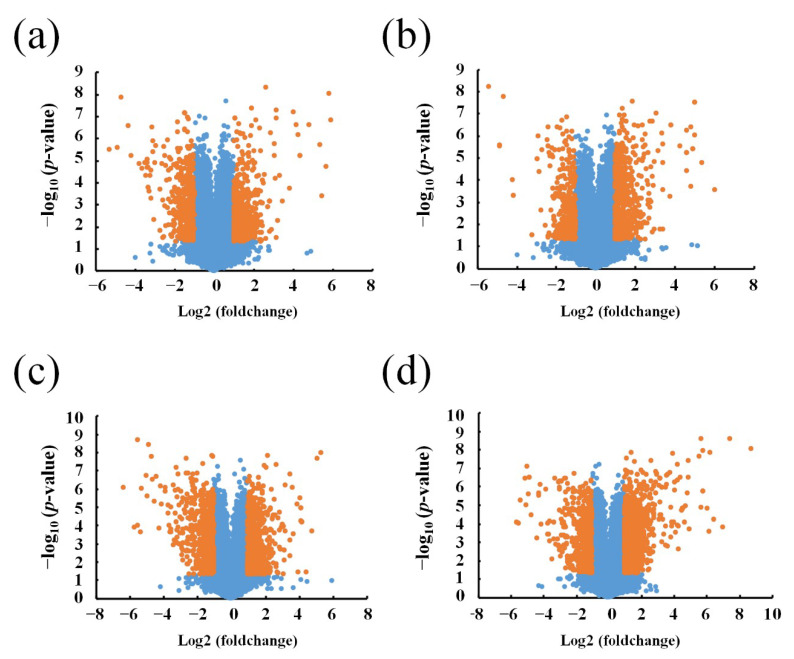
Volcano plots of differentially expressed long noncoding RNAs (lncRNAs). (**a**) 1-month-old, (**b**) 3-month-old, (**c**) 6-month-old, and (**d**) 9-month-old APP/PS1 mice, compared to their respective age-matched wild type (WT) control mice. Red points stand for lncRNAs that significantly changed, with a fold change ≥ 2 and *p*-values ≥ 0.5.

**Figure 2:**
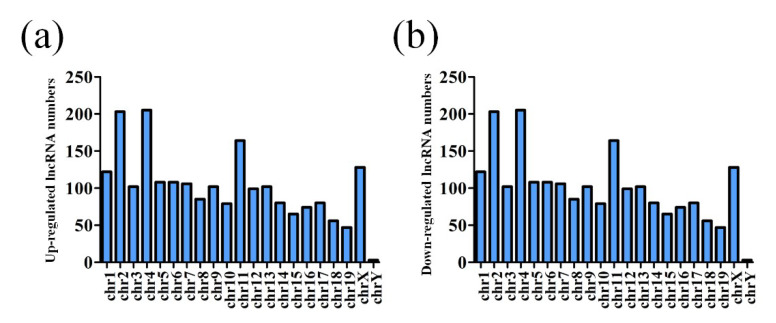
Chromosome location distribution of lncRNAs. (**a**) Upregulated lncRNAs and (**b**) downregulated lncRNAs.

Agilent LncRNA Microarray was performed to compare the expression of lncRNAs and protein-coding transcripts in the APP/PS1 mice and WT control mice, with a capacity to detect approximately 30,586 lncRNAs and 26,109 coding transcripts. The Volcano plot revealed the different lncRNA expression profiles as shown in Figure 1 (fold change ≥ 2, *p*-value ≤ 0.05). A total of 3306 lncRNAs were identified as aberrantly expressed among four groups of AD mice of different age and matched control. According to the age of each group of AD mice, 554 upregulated lncRNAs and 346 downregulated lncRNAs were found in the 1-month-old APP/PS1 mice, when compared to their age-matched WT mice used as controls. Moreover, the expression of 635 lncRNAs increased, whereas 362 lncRNAs decreased in the 3-month-old APP/PS1 mice, when compared to their WT counterparts. Similarly, the expression of 362 lncRNAs was increased, but reduced in 643 lncRNAs in the 6-month-old APP/PS1 mice, while the expression of 1145 lncRNAs was increased, but decreased in 649 lncRNAs in the 9-month-old APP/PS1 mice, when compared to their WT controls (Tables S1–S4). The distribution of the differentially expressed lncRNAs is shown in Figure 2.

**Figure 3:**
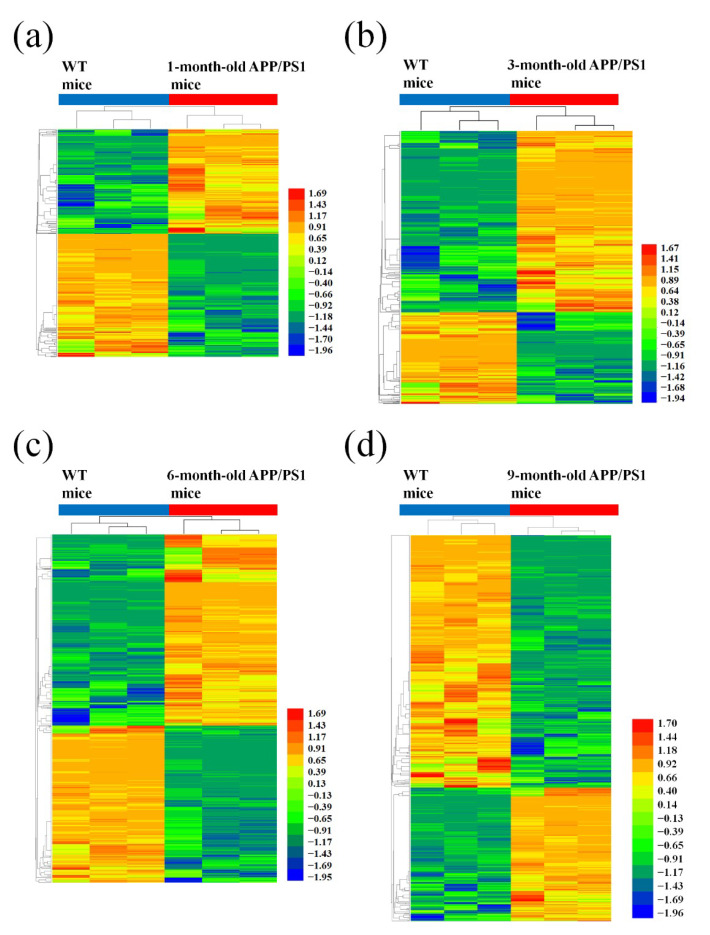
Hierarchical cluster analysis of differentially expressed mRNAs in APP/PS1 mice. (**a**) 1-month-old, (**b**) 3-month-old, (**c**) 6-month-old, and (**d**) 9-month-old APP/PS1 mice, compared to their respective age-matched WT control mice. A hierarchical cluster showing the differentially expressed genes indicating their differences from blue to red (the higher the value, the redder the cluster).

Correspondingly, a total of 2458 mRNAs were aberrantly expressed among the four AD mice groups of different age and corresponding matched control. The data showed significant changes in the expression of 619 mRNAs from the 1-month-old APP/PS1 mice and 694 mRNAs from the 3-month-old APP/PS1 mice. Prominently altered expression changes were also observed in 1191 mRNAs obtained from the 6-month-old APP/PS1 mice and in 1265 mRNAs obtained from the 9-month-old APP/PS1 mice, when compared to their WT control counterparts (Figure 3, Tables S5–S8).

**Figure 4:**
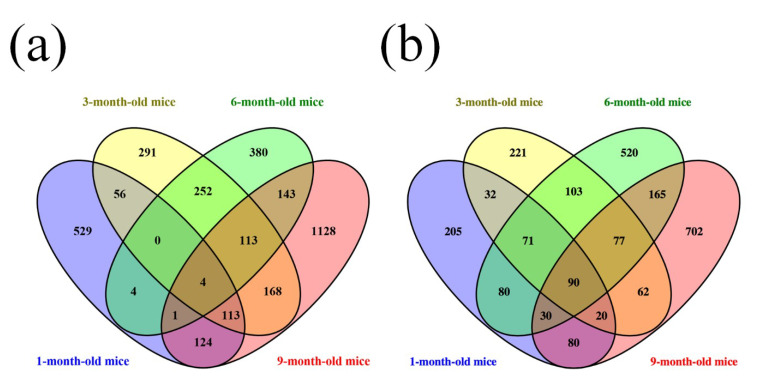
Venn diagram of differentially expressed lncRNAs and mRNAs. (**a**) LncRNA expression and (**b**) mRNA expression among 1-month-old, 3-month-old, 6-month-old, and 9-month-old APP/PS1 mice.

**Figure 5:**
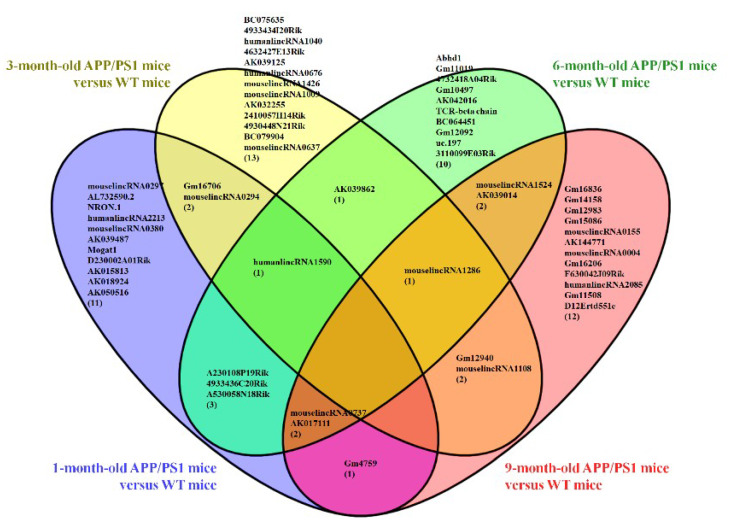
Venn diagram of the top 10 differentially expressed lncRNAs among 1-month-old, 3-month-old, 6-month-old, and 9-month-old APP/PS1 mice as compared to their respective age-matched WT control mice.

Furthermore, a Venn diagram was used to analyze the changes in lncRNA and mRNA expression among the different four age groups (Figure 4a,b). The diagram showed the significant change in the expression of 4 lncRNAs and 90 mRNAs at the different developmental stages of the APP/PS1 mice analyzed. Among these deregulated lncRNAs between the APP/PS1 mice and WT control mice, four lncRNAs were found to show continuous changes in every age group. AK081040 and ENSMUST00000119471 were significantly upregulated, and AK142586 and ENSMUST00000117578 were significantly downregulated. Additionally, the top 10 aberrantly expressed lncRNAs of each age group were selected and analyzed. The diagram indicated that among the top10 deregulated lncRNAs in all age groups (Figure 5, Table S9), when compared to their WT control mice, humanlincRNA1590, AK017111, mouselincRNA0737, and mouselincRNA1286 were found to show the relatively constant change. HumanlincRNA1590 was significantly upregulated in 1-month-old, 3-month-old, and 6-month-old APP/PS1 mice. AK017111 had the constant downregulated tendency in 1-month-old, 6-month-old, and 9-month-old APP/PS1 mice. MouselincRNA0737 and mouselincRNA1286 were both aberrantly increased, respectively, in 1-month-old, 6-month-old, and 9-month-old APP/PS1 mice and 3-month-old, 6-month-old, and 9-month-old APP/PS1 mice.

### 3.2. GO and KEGG Pathway Analysis

**Figure 6:**
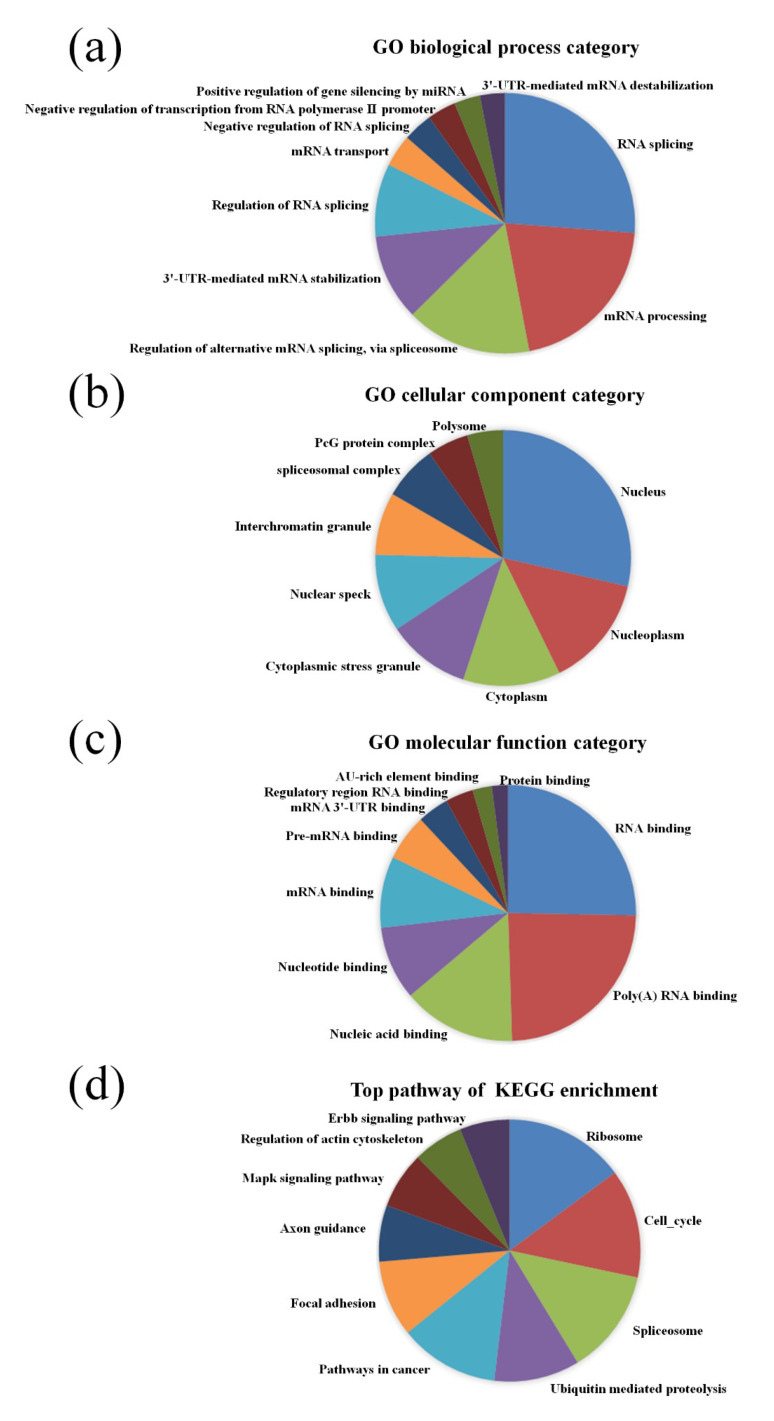
Gene Ontology (GO) enrichment and Kyoto Encyclopedia of Genes and Genomes (KEGG) pathway analysis of the aberrantly expressed lncRNAs between APP/PS1 mice and control. The top 10 most enriched GO categories and pathways were calculated and plotted. (**a**) Biological process; (**b**) cellular component; (**c**) molecular function; (**d**) KEGG pathway.

**Figure 7:**
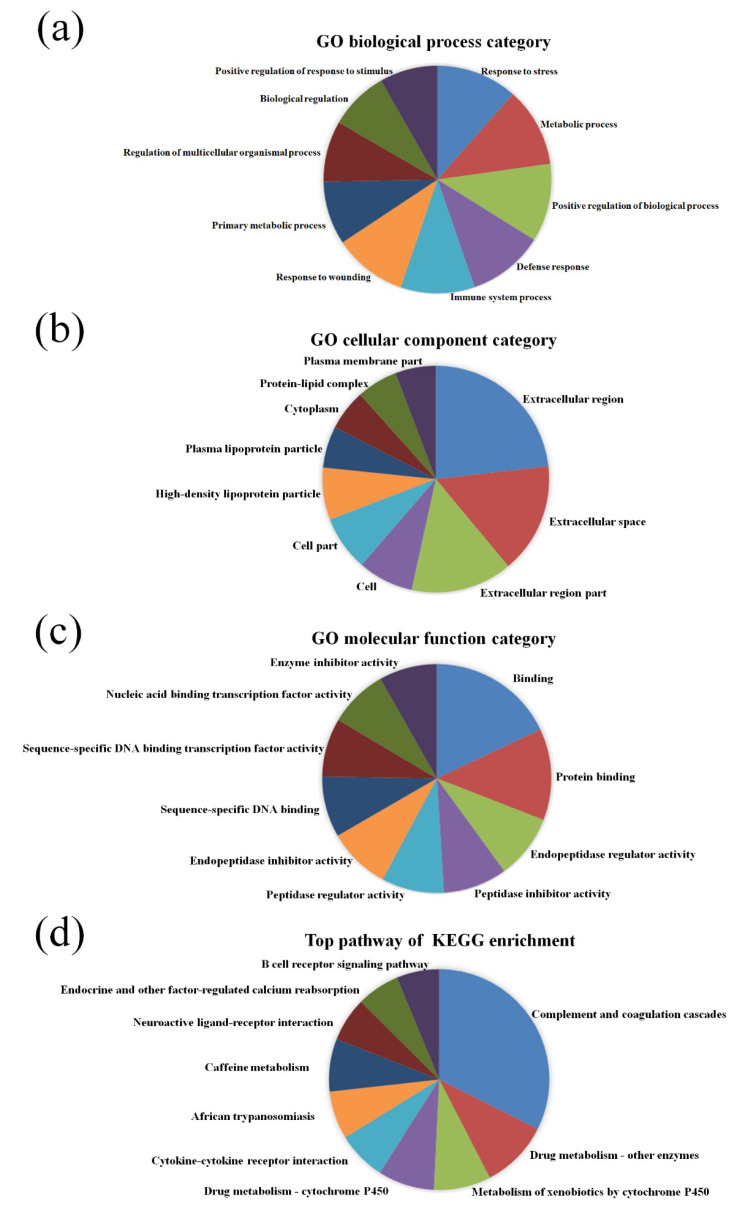
GO enrichment and KEGG pathway analysis of the aberrantly expressed mRNAs between APP/PS1 mice and control. The top 10 most enriched GO categories and pathways were calculated and plotted. (**a**) Biological process; (**b**) cellular component; (**c**) molecular function; (**d**) KEGG pathway.

**Figure 8:**
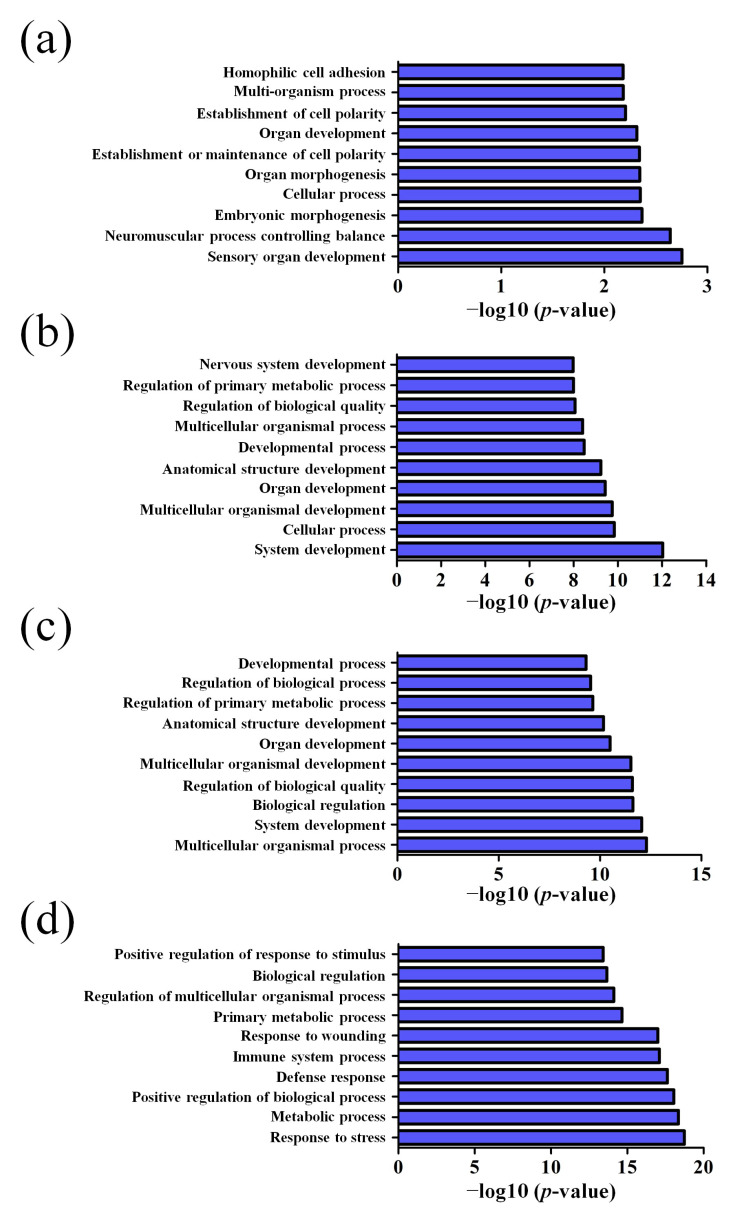
Top 10 GO biological processes of the upregulated mRNAs in APP/PS1 mice. (**a**) 1-month-old, (**b**) 3-month-old, (**c**) 6-month-old, and (**d**) 9-month-old APP/PS1 mice.

**Figure 9:**
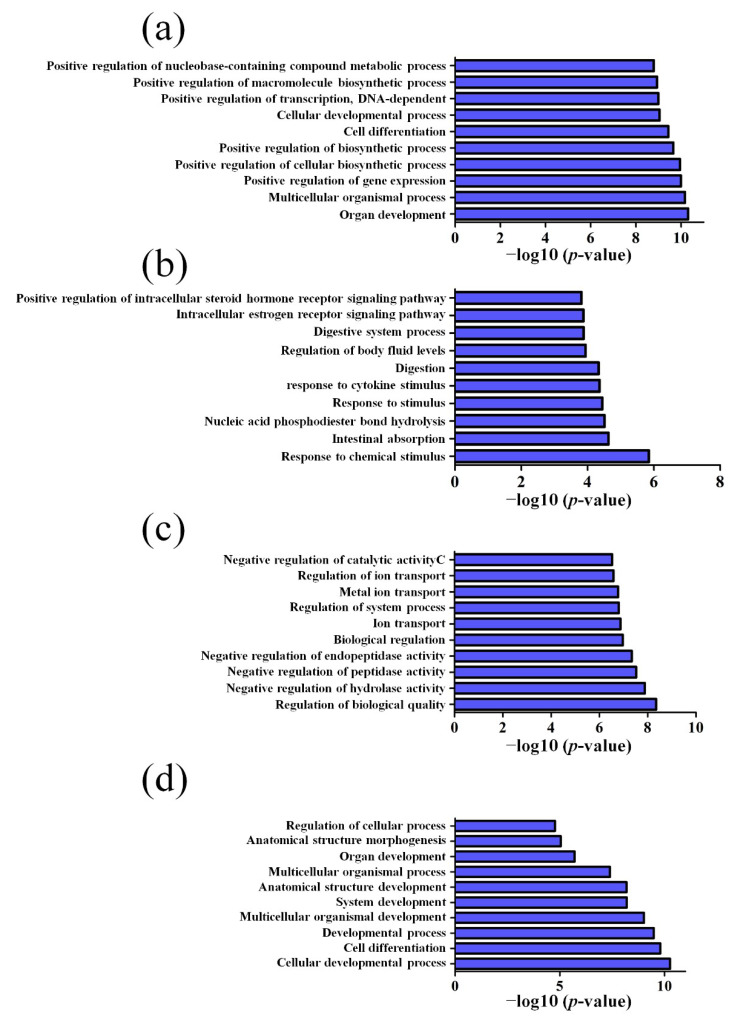
Top 10 GO biological processes of the downregulated mRNAs in APP/PS1 mice. (**a**) 1-month-old, (**b**) 3-month-old, (**c**) 6-month-old, and (**d**) 9-month-old APP/PS1 mice.

**Figure 10:**
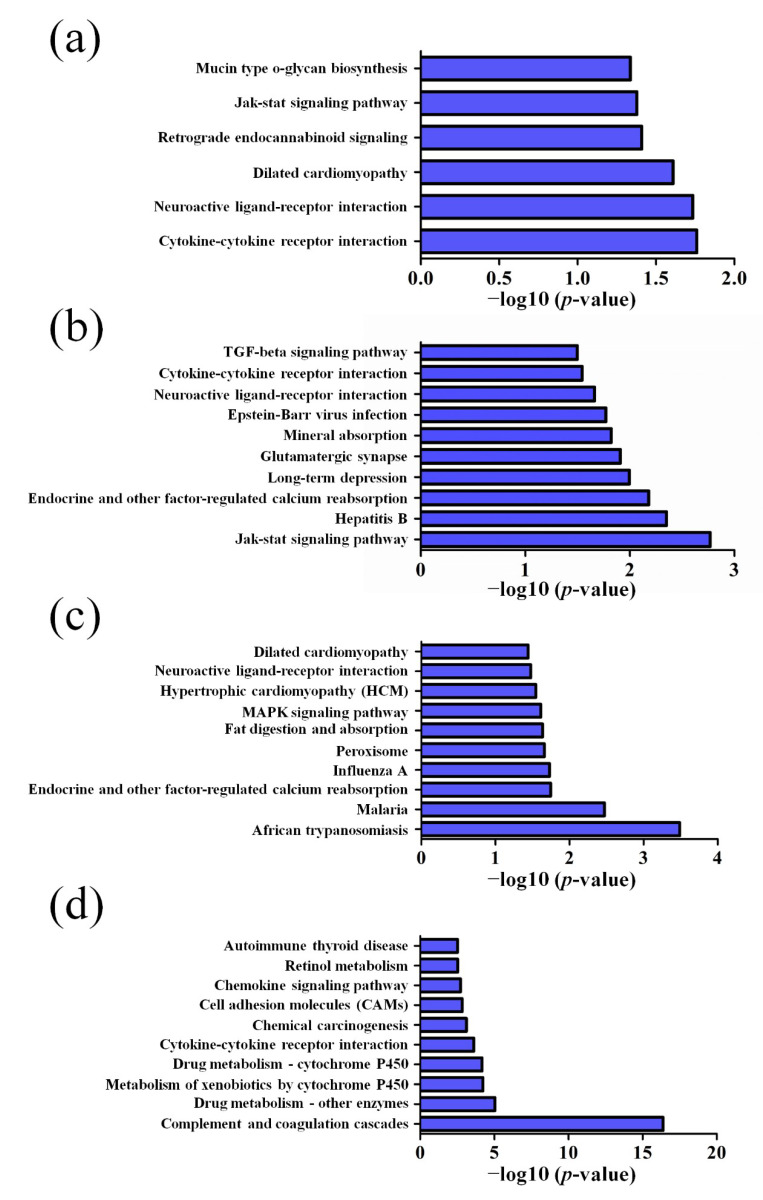
Top 10 KEGG pathways of the upregulated mRNAs in APP/PS1 mice. (**a**) 1-month-old, (**b**) 3-month-old, (**c**) 6-month-old, and (**d**) 9-month-old APP/PS1 mice.

**Figure 11:**
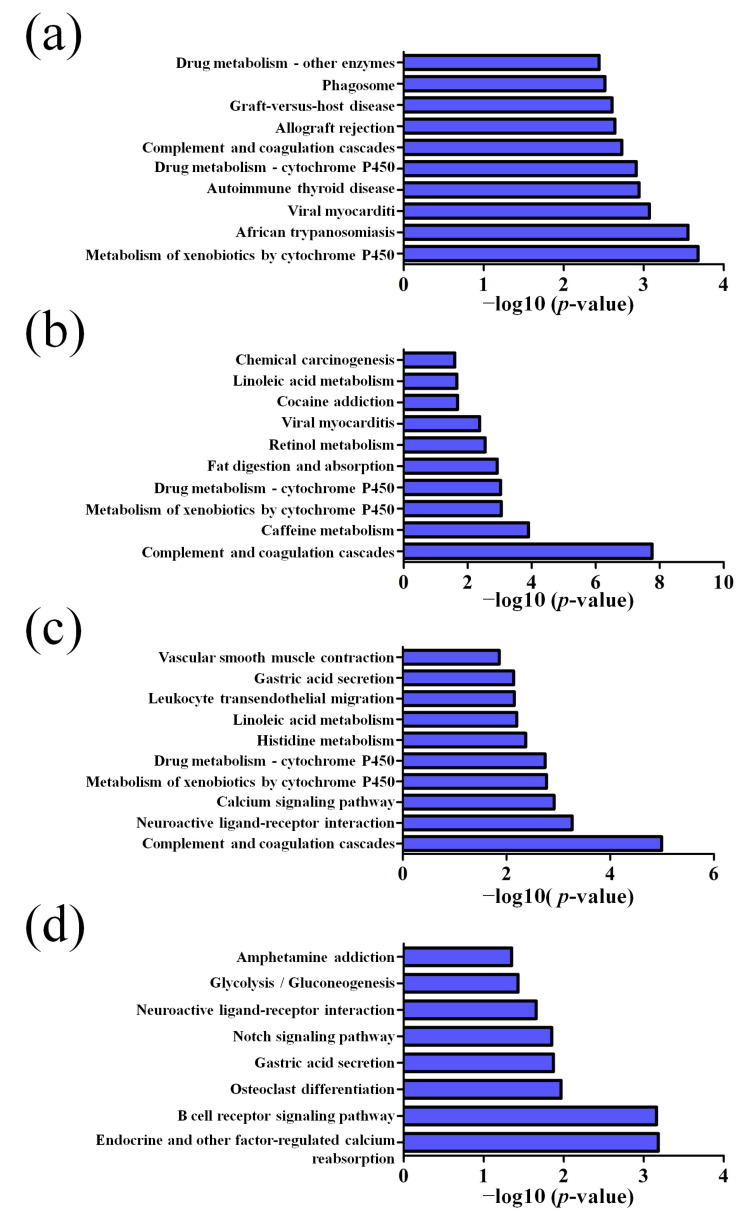
Top 10 KEGG pathways of the downregulated mRNAs in APP/PS1 mice. (**a**) 1-month-old, (**b**) 3-month-old, (**c**) 6-month-old, and (**d**) 9-month-old APP/PS1 mice.

GO pathway analysis was employed to analyze the biological functions of the differently expressed lncRNAs and mRNAs found in the different age groups of APP/PS1 mice. The KEGG pathway analysis was also performed to identify the enriched pathways of these differentially expressed lncRNAs and mRNAs obtained from the microarray experiment. The top 10 biological functions associated to the GO terms and KEGG pathways for the differently expressed lncRNAs and mRNAs are described in Figure 6 and Figure 7. The GO enrichment analysis indicated that the abnormally expressed lncRNAs were mostly enriched in RNA splicing in the Biological process (Figure 6a), nucleus in the Cellular component (Figure 6b), and RNA binding in the molecular function (Figure 6c), while the aberrant mRNAs were mostly inclined to a positive regulation of biological process, extracellular region, and binding corresponding to the Biological process (Figure 7a), Cellular component (Figure 7b), and molecular function (Figure 7c). The KEGG analysis indicated that the differently expressed lncRNAs were mainly involved in the ribosome, cell cycle, and spliceosome processes, ErbB signaling pathway, and MAPK signaling pathway (Figure 6d), while the differently expressed mRNAs were mainly involved in the complement and coagulation cascades, drug metabolism, neuroactive ligand-receptor interaction, and cytokine-cytokine receptor interaction (Figure 7d). Furthermore, the aberrantly upregulated and downregulated mRNAs at different ages were also separately subjected to GO biological process and KEGG analysis as an additional analysis for these different pathological stages of AD mice. The top 10 GO biological processes and KEGG analyses of up- and downregulated mRNAs in APP/PS1 mice are shown in Figure 8, Figure 9, Figure 10 and Figure 11.

### 3.3. lncRNA–mRNA–miRNA Co-Expression Network

**Figure 12:**
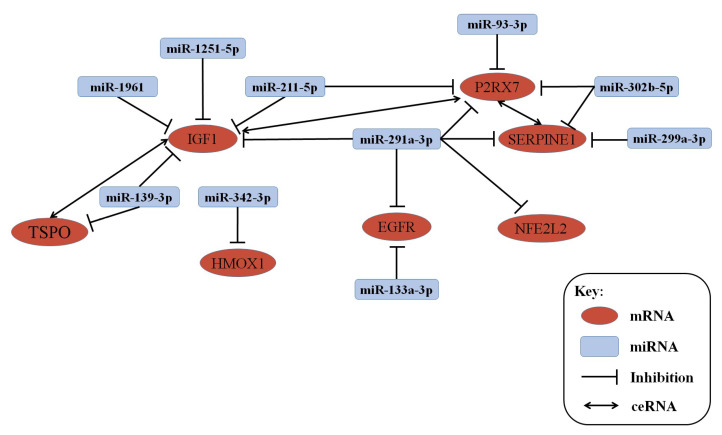
miRNA–mRNA network of miRNAs and mRNAs expressed at different developmental stages.

**Figure 13:**
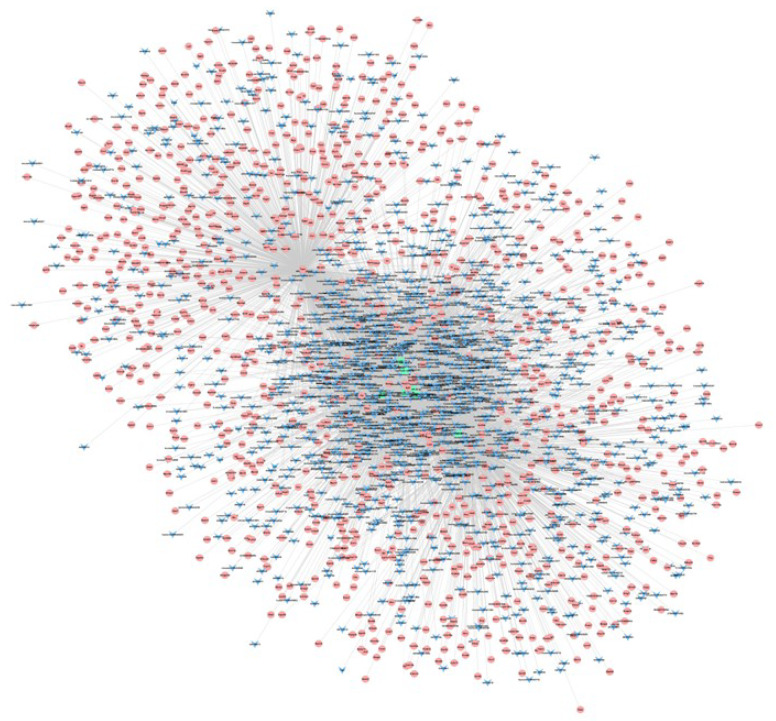
LncRNA–mRNA–miRNA co-expression network.

In our previous analysis, our results showed that the expression of 58 miRNAs were significantly changed in 1-month-old, 3-month-old, 6-month-old, and 9-month-old APP/PS1 mice [26]. A total of seven genes with conservative evolution, such as IGF1, P2RX7, EGFR, TSPO, SERPINE1, HMOX1, and NEF212, significantly changed in every age group of APP/PS1 mice, and then, the mRNA–miRNA co-expression network between them was established, as shown in Figure 12. Connected with the lncRNA data obtained in this study, the lncRNA–mRNA–miRNA network was also constructed, as shown in Figure 13 and Figure S1.

## 4. Discussion

AD is a progressive and irreversible neurodegenerative disease caused by a number of aberrant gene expressions that are still not well understood [27,28]. Markedly, the present study investigated the dysregulated lncRNAs and their associated mRNAs in the development and/or progression of AD, using the APP/PS1 transgenic mouse model that is reported as a well-known model presenting vital AD symptoms such as Aβ accumulation and cognitive decline from young age [29]. A total of 3306 lncRNAs and 2458 mRNAs were identified as differentially expressed between different age of AD mice groups and matched control. The pathway analysis showed that the neuroinflammation-related and synaptic transmission signaling pathways represented the main enriched pathways of the dysregulated lncRNAs and correlated mRNAs. An lncRNA–mRNA–miRNA network was constructed. An mRNA–miRNA network between both constantly dysregulated and highly conserved genes was also constructed; among them, the genes IGF1, P2RX7, TSPO, SERPINE1, EGFR, HMOX1, and NFE212 were predicted to play a role in the development of AD.

It is known that the APP/PS1 mouse with AD shows progressive cognitive deterioration and develops Aβ-associated pathology with increasing age [19,29]. The 1-month-old APP/PS1 mice do not show visible symptoms of AD and serve as the incubation stage of AD. The 3-month-old APP/PS1 mice were reported to show reduced brain and intracranial volumes but not cerebral Aβ plaques compared with controls [30]. Pet imaging studies showed that amyloid related signals can be found in the cortex, hippocampus, and striatum of the 6-month-old APP/PS1 mice and are easily recognizable in the 9-month-old APP/PS1 mice, suggesting that the Aβ symptom become significant with increasing age [31]. The 6-month-old APP/PS1 mice show short-term memory deficits, which are not evident at 3-month age [32]. The 9-month-old APP/PS1 mice show hypoactivity in an open field and deficits in spatial memory [33]. Thus, 1-month-old, 3-month-old, 6-month-old, and 9-month-old APP/PS1 mice were chosen because of the involvement of the latent period and the early and progressive stages of AD for investigating the abnormal gene changes.

A total of 3306 lncRNAs were dysregulated among the different age groups of the APP/PS1 transgenic mice. Notably, four lncRNAs continuously changed in every age group: two of them, AK081040 and ENSMUST00000119471, were significantly upregulated, while the remaining two, AK142586 and ENSMUST00000117578, were significantly downregulated. Additionally, humanlincRNA1590, AK017111, mouselincRNA0737, and mouselincRNA1286 were uniquely changed within the top 10 differentially expressed lncRNAs from 1-month-old or 3-month-old to 9-month-old APP/PS1 mice. Regarding current knowledge, no reports are available on the association of these lncRNAs with the roles in diseases, especially in the pathology of AD. These novel lncRNAs discovered from AD progression need to be investigated for the functional role and regulatory mechanisms in AD pathology in future research. Importantly, a total of 2458 mRNAs were dysregulated throughout the four age groups, with 90 mRNAs presenting significant changes during the progression of AD. Among them, some were closely associated with AD, for example DNA methyltransferase 1 (DNMT1). DNMT1 is a vital methyltransferase enzyme mediating epigenetic functions that influence cell processes, such as cell proliferation and invasion [34]. Several studies demonstrated that the hypermethylation of the APP gene in the hippocampus and cortex is linked to the overproduction and accumulation of Aβ [35]. In addition, DNA methylation can regulate the expression of specific genes, such as BACE1 and PS1, and thus modulate Aβ related processes as well [36].

Despite the previous findings on the abnormally expressed lncRNAs in postmortem human brains with AD or late-onset AD, the knowledge about the pathophysiological roles of lncRNAs in AD is still limited. According to the GO and KEGG analysis, dysregulated lncRNAs in the APP/PS1 mouse model are mainly involved in mRNA regulation, such as processing, splicing, stability, and transport, miRNA feedback regulation, and ribosome and spliceosome pathological process. Further KEGG analyses revealed that the mRNAs associated with the dysregulated lncRNAs in the APP/PS1 mouse model are involved in neuroinflammation-related pathways, such as JAK-STAT signaling pathway and MAPK signaling pathway. Additionally, the synaptic transmission signaling pathways, such as glutamatergic synapse, neuroactive ligand-receptor interaction, and long-term depression, are also involved in the different age groups representing different stages of AD mice. Therefore, inflammatory-related biological processes (JAK-STAT signaling pathway, MAPK signaling pathway, and ErbB signaling pathway) and neuron-related biological processes (glutamatergic synapse, neuroactive ligand-receptor interaction, long-term depression, and axon guidance) represented the main enriched pathways of the mRNAs correlated with the dysregulated lncRNAs. Compared with a previous study on the expression of lncRNAs in an AD-associated model, several similar KEGG pathways, such as the MAPK signaling pathway, JAK-STAT signaling pathway, glutamatergic synapse, and neuroactive ligand-receptor interaction, were predicted [37]. Since neither model could replicate the real progressive degeneration in AD, these signaling pathways in common between different AD models might exert an important role in the regulation of the neural system and pathogenesis of AD.

In addition, the combination with the miRNA microarray data allowed to construct an mRNA–miRNA network. Our results demonstrated that seven mRNAs and 10 miRNAs were significantly changed in each of the tested stage groups of the APP/PS1 mice and were highly conserved through evolution. The seven mostly correlated mRNAs with miRNAs in the different stage groups of APP/PS1 mouse model were IGF1, P2RX7, EGFR, TSPO, SERPINE1, HMOX1, and NEF212.

Previous studies showed that the IGF1 protein is of vital importance in brain development during embryogenesis, and during the process of aging, when circulating IGF1 and brain IGF1 receptors decline [38]. P2X7Rs are ATP-gated, non-selected channels, and key regulators of the inflammasome molecular complex [39,40]. It is reported that the inhibition of P2X7R in transgenic mice expressing a mutant form of the human APP resulted in a reduction of amyloid plaques in the hippocampus [41]. EGFR, TSPO, and SERPINE1 are also associated with AD through the induction of cell apoptosis, cell cycle regulation, synaptic vesicle cycle and inflammation [42,43,44]. Two genes, HMOX1 and NEF212 were found for the first time as linked with AD; thus, it is necessary to investigate further how they act in this disease.

Recently, lncRNAs have been reported to participate in competing endogenous RNAs (ceRNAs) activities to communicate with mRNAs, by sharing common miRNA-binding sites with mRNAs [45]. Correspondingly, an lncRNA–mRNA–miRNA network was constructed on the basis of the observed changes in the expression of miRNA-mRNA networks. As regard the selected mRNAs–miRNAs, IGF1 is the competing endogenous RNA (ceRNA) to promote P2RX7 and TSPO gene expression by targeting the same miRNA and P2RX7 is the ceRNA to promote Serpin E1 gene expression. The mRNA-circled lncRNA can also play a role through competitive binding. Considerably, these lncRNAs, mRNAs, and miRNAs may counteract each other in order to modulate the processes underlying the development and/or progression of AD.

Some limitations are present in our study. First, the sample size used in our analysis was small. Second, more suitable AD models, such as senescence-accelerated mouse prone 8 (SAMP8) mice, triple-transgenic mouse AD model, and even AD patient blood, should be utilized to validate the selected aberrant lncRNAs, mRNAs, and miRNAs from the microarray results using quantitative PCR. Additionally, further experiments involving RNA interference and RNA immunoprecipitation sequencing should be used to investigate the underlying biological functions and molecular mechanisms of these selected lncRNAs and mRNAs, to elucidate the contribution in the development and/or progression of AD. Our results could be improved by a deeper data analysis and more extensive validation of lncRNAs and mRNAs in AD.

## 5. Conclusions

In conclusion, this study was the first lncRNA and mRNA microarray analysis of the brain of an AD mouse model using different stages of AD by the use of different age groups, Through bioinformatics analysis, a number of dysregulated lncRNAs and mRNAs were identified between different age groups of AD mice and matched control, and the signaling pathways referred to AD were predicted. Furthermore, an lncRNA–mRNA–miRNA network was constructed. Many pathological genes, such as IGF-1, P2RX7, TSPO, SERPINE1, EGFR, HMOX1, and NFE212 were dysregulated and conserved in evolution with a potential target correlation to AD. Thus, our study provided a clue to understand the pathological hallmarks of AD and effective therapeutic targets based on the altered lncRNA in AD, which might act as “markers” to distinguish the pathological changes in this disease.

## Supplementary Materials

The following are available online at https://www.mdpi.com/2075-1729/10/5/64/s1. The differently expressed lncRNAs in the brain of APP/PS1 mice at different age compared with age-matched WT control are provided in the following Supplementary Tables: Table S1 for 1-month-old APP/PS1 mice, Table S2 for 3-month-old APP/PS1 mice, Table S3 for 6-month-old APP/PS1 mice, and Table S4 for 9-month-old APP/PS1 mice, compared with their corresponding age-matched WT mice, respectively. The differently expressed mRNAs in the brain of APP/PS1 mice at different age compared with age-matched WT control are provided in the following supplementary Tables: Table S5 for 1-month-old APP/PS1 mice, Table S6 for 3-month-old APP/PS1 mice, Table S7 for 6-month-old APP/PS1 mice, and Table S8 for 9-month-old APP/PS1 mice, compared with their corresponding age-matched WT mice, respectively. The top 10 differentially expressed lncRNAs at 1-month-old, 3-month-old, 6-month-old, and 9-month-old APP/PS1 mice as compared to their respective age-matched WT control mice are provided in Table S9. The unambiguous figure of lncRNA–mRNA–miRNA co-expression network (Figure 13 in the main text) was uploaded as Figure S1.
