# Correction: Wang et al. Microarray Profile of Long Noncoding RNA and Messenger RNA Expression in a Model of Alzheimer’s Disease. *Life* 2020, *10*, 64

**DOI:** 10.3390/life14030326

**Published:** 2024-02-29

**Authors:** Linlin Wang, Li Zeng, Hailun Jiang, Zhuorong Li, Rui Liu

**Affiliations:** Institute of Medicinal Biotechnology, Chinese Academy of Medical Sciences and Peking Union Medical College, Beijing 100050, China; wanglinlin@wfmc.edu.cn (L.W.); zengli@imb.pumc.edu.cn (L.Z.); jianghailun@bjtth.org (H.J.)

## Author’s Email Updates

We have updated the email addresses of Li Zeng and Hailun Jiang as the two authors’ previous email addresses are no longer in use. We have updated the email addresses of Li Zeng and Hailun Jiang to zengli@imb.pumc.edu.cn and jianghailun@bjtth.org.

## Error in Figure

In the original publication [1], there was a mistake in Figure 2b as published. In Figure 2, the chromosome location distribution of downregulated lncRNAs (Figure 2b) was inadvertently duplicated for those of the upregulated lncRNAs (Figure 2a). The corrected Figure 2 appears below. The authors state that the scientific conclusions are unaffected. The authors regret this error.

Corrected Figure 2:

**Figure 2:**
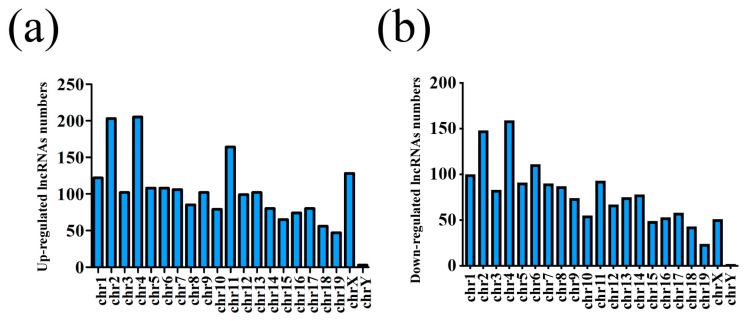
Chromosome location distribution of lncRNAs. (**a**) Upregulated lncRNAs and (**b**) downregulated lncRNAs.
