# Supplementary material for: Microarray Profile of Long Noncoding RNA and Messenger RNA Expression in a Model of Alzheimer’s Disease
Source: Life (Basel). 2020 May 14;10(5):64. doi: 10.3390/life10050064 (PMC7281340; doi:10.3390/life10050064)
Supplement: Supplementary file 1 [file life-10-00064-s001.zip › life-787240-supplemenatry-to be published - PDF/life-787240-supplementary/Table S1.pdf]

life

MDPI

Supplementary

Microarray Profile of Long Noncoding RNA and Messenger RNA Expression in a Model of Alzheimer's Disease

Linlin Wang †, Li Zeng †, Hailun Jiang, Zhuorong Li * and Rui Liu *

Institute of Medicinal Biotechnology, Chinese Academy of Medical Sciences and Peking Union Medical College, Beijing 100050, China; wanglinlin@wfmc.edu.cn (L.W.); zengsheng@imb.pumc.edu.cn (L.Z.); jianghailun@imb.pumc.edu.cn (H.J.)

* Correspondence: lizhourong@imb.pumc.edu.cn (Z.L.); +86-10-8352017; .liurui@imb.pumc.edu.cn (R.L.); Tel.: +86-10-67087731

Table S1. Differently expressed lncRNAs in the brain of 1-month-old APP/PS1 mice compared with age-matched WT mice.

Probe Name	Gene Symbol	*p*-Value	Fold Change	Regulation
MM9LINCRNAEXON11653-_P1	mouselincRNA0297	0.03853617	2.4391737	up
ASMM9PARTA018633	AL732590.2	0.002783807	2.15185	up
ASMM9PARTA018633	AL732590.2	0.002783807	2.15185	up
ASMM9PARTA017102	A230108P19Rik	0.001155623	2.6593003	up
mouselincRNA0294-_P1	mouselincRNA0294	0.024343088	2.5895295	up
MM9LINCRNAEXON11250-_P1	mouselincRNA0737	0.001355704	3.430497	up
humanlincRNA1590+_P1	humanlincRNA1590	0.0000437	5.9778385	up
ASMM9PARTA018203	NRON.1	0.003656608	3.9517019	up
humanlincRNA2213+_P1	humanlincRNA2213	0.017903363	2.0292795	up
MM9LINCRNAEXON11529-_P1	mouselincRNA0380	0.001012912	2.3298893	up
ASMM9PARTA016545	Gm16706	0.003379951	3.3808007	up
ASMM9PARTA016545	Gm16706	0.003379951	3.3808007	up
ASMM9PARTA006499		0.006989687	2.7657256	up
ASMM9PARTA045030	Pisd-ps3	0.0000905	2.024496	up
ASMM9PARTA002774		0.0000651	2.2739878	up
ASMM9PARTA002774		0.0000651	2.2739878	up
ASMM9PARTA005433		0.003971536	2.0467112	up
ASMM9PARTA005433		0.003971536	2.0467112	up
ASMM9PARTA005433		0.003971536	2.0467112	up
ASMM9PARTA005433		0.003971536	2.0467112	up
ASMM9PARTA010873	Gm15382	0.000167	2.064617	up
mouselincRNA0394-_P1	mouselincRNA0394	0.002045447	3.7254975	up
ASMM9PARTA015256	9130017K11Rik	0.003683128	4.075082	up
ASMM9PARTA015256	9130017K11Rik	0.003683128	4.075082	up
ASMM9PARTA046970	BC006965	0.0000554	4.5558357	up
ASMM9PARTA009122		0.037647396	2.2806401	up
ASMM9PARTA045330	Gm11149	0.0000477	2.4906142	up
ASMM9PARTA045330	Gm11149	0.0000477	2.4906142	up

ASMM9PARTA045330	Gm11149	0.0000477	2.4906142	up
CA465684_P1	mouselincRNA0967	0.0000159	3.0758736	up
ASMM9PARTA014137	Gm12347	0.038847048	2.133187	up
ASMM9PARTA047362	BC064451	0.000171	2.071279	up
ASMM9PARTA046506	mKIAA4192	0.03495005	3.2468708	up
ASMM9PARTA002041		0.040164176	5.1750097	up
ASMM9PARTA004295		0.001527703	2.9495368	up
ASMM9PARTA009911	4933401B06Rik	0.003712294	2.6109204	up
CJ058094_P1	mouselincRNA1482	0.007969993	2.0391238	up
CJ058094_P1	mouselincRNA1482	0.007969993	2.0391238	up
CJ058094_P1	mouselincRNA1482	0.007969993	2.0391238	up
ASMM9PARTA013368	Olfr1027-ps1	0.028849883	2.1941142	up
ASMM9PARTA005223		0.009152568	2.0864286	up
ASMM9PARTA012572	Gm13323	0.000122	2.0895674	up
MM9LINCRNAEXON11396+_P1		0.008838809	2.0950077	up
ASMM9PARTA045345	9630013A20Rik	0.000000864	3.6551394	up
ASMM9PARTA018592	Gm16343	0.025719661	2.954755	up
ASMM9PARTA018592	Gm16343	0.025719661	2.954755	up
ASMM9PARTA018592	Gm16343	0.025719661	2.954755	up
ASMM9PARTA018592	Gm16343	0.025719661	2.954755	up
ASMM9PARTA015766	9530082P21Rik	0.004168574	3.3884056	up
ASMM9PARTA005159		0.035993755	2.8546026	up
ASMM9PARTA013083	Gm12539	0.00075	2.0123758	up
ASMM9PARTA003991		0.000343	3.452115	up
ASMM9PARTA014466	Spag17-ps	0.041225985	2.3006616	up
ASMM9PARTA013061	Gm13913	0.000024	4.049263	up
ASMM9PARTA000138		0.015808158	2.5815477	up
ASMM9PARTA008375		0.011198638	2.6658108	up
ASMM9PARTA005494		0.00778921	3.6653523	up
ASMM9PARTA013683	Gm12037	0.027591476	4.2206707	up
ASMM9PARTA007231		0.002745976	3.5619643	up
ASMM9PARTA016815	Gm16534	0.00000966	2.5082474	up
ASMM9PARTA017337	1700021L23Rik	0.006257789	2.8913448	up
ASMM9PARTA007917		0.00000385	5.102149	up
ASMM9PARTA000849		0.008761234	3.799671	up
ASMM9PARTA005541		0.017299106	7.057314	up
ASMM9PARTA005492		0.015809843	2.2901855	up
ASMM9PARTA051615	mKIAA1781	0.049255732	5.220143	up
ASMM9PARTA051615	mKIAA1781	0.049255732	5.220143	up
ASMM9PARTA000911		0.01189079	2.0553722	up
ASMM9PARTA017280	Gm13976	0.004594597	4.2734804	up
ASMM9PARTA010969	Gm15399	0.012785287	2.777508	up
ASMM9PARTA005182		0.01946577	2.05024	up
MM9LINCRNAEXON11747+_P1	mouselincRNA0230	0.002470979	2.2702074	up
ASMM9PARTA006171		0.022997985	2.2174146	up
ASMM9PARTA014414	5430427M07Rik	0.01166787	4.3312845	up
ASMM9PARTA018542	AC164629.8	0.014509238	2.5523677	up
humanlincRNA2028-_P1	humanlincRNA2028	0.006392615	4.0605407	up
ASMM9PARTA005913		0.03353239	4.2164035	up
ASMM9PARTA016544	Gm13134	0.000381	4.21255	up
ASMM9PARTA016544	Gm13134	0.000381	4.21255	up
ASMM9PARTA007950		0.025528518	2.468305	up
humanlincRNA1576-_P1	humanlincRNA1576	0.00000755	3.3827791	up
ASMM9PARTA001768		0.014294921	2.2706404	up
humanlincRNA1422+_P1	humanlincRNA1422	0.000877	5.4090686	up
humanlincRNA1605+_P1	humanlincRNA1605	0.01659449	2.5616944	up
MM9LINCRNAEXON10275-_P1	mouselincRNA1493	0.003365036	2.934753	up

ASMM9PARTA011628	Olfr1063-ps1	0.00590611	2.1198711	up
ASMM9PARTA004846		0.010118948	2.7207916	up
ASMM9PARTA004846		0.010118948	2.7207916	up
ASMM9PARTA004846		0.010118948	2.7207916	up
CUST_423_PI426073487	uc.425	0.000112	2.734947	up
ASMM9PARTA017117	Gm13847	0.033471078	9.067197	up
ASMM9PARTA002726		0.026826017	2.6987271	up
MM9LINCRNAEXON10302-_P1	mouselincRNA1353	0.009161566	2.0896251	up
ASMM9PARTA047940	AK014435	0.000414	4.6516943	up
ASMM9PARTA049301	AK018940	0.03574843	6.1800933	up
ASMM9PARTA008118		0.047661927	2.3506238	up
ASMM9PARTA011622	Gm12377	0.0000738	2.1792977	up
ASMM9PARTA005824		0.03493068	2.3046925	up
ASMM9PARTA006453		0.010957906	2.9548705	up
MM9LINCRNAEXON10551-_P1	mouselincRNA1185	0.00593149	2.4590445	up
ASMM9PARTA046271	BC108272	0.000964	4.4767447	up
AI507909_P1	humanlincRNA1257	0.000396	2.8773766	up
ASMM9PARTA046107	AK039957	0.037938744	2.1578417	up
ASMM9PARTA005449		0.000423	2.6646652	up
ASMM9PARTA005449		0.000423	2.6646652	up
ASMM9PARTA000694		0.001420073	2.284793	up
ASMM9PARTA015960	9430065F17Rik	0.017808545	2.5310738	up
ASMM9PARTA015960	9430065F17Rik	0.017808545	2.5310738	up
ASMM9PARTA047687	AK015470	0.001142327	2.2665412	up
MM9LINCRNAEXON11061-_P1	mouselincRNA0945	0.03493923	2.1173036	up
ASMM9PARTA000471		0.008085524	2.287788	up
ASMM9PARTA016149	Gm15726	0.002023748	2.390047	up
ASMM9PARTA016149	Gm15726	0.002023748	2.390047	up
MM9LINCRNAEXON10406-_P1	mouselincRNA1260	0.02519224	2.2607043	up
ASMM9PARTA004872		0.02106976	2.4793973	up
ASMM9PARTA006207		0.007572139	3.6399913	up
ASMM9PARTA014358	E130003G02Rik	0.000532	2.0517626	up
ASMM9PARTA014358	E130003G02Rik	0.000532	2.0517626	up
ASMM9PARTA014358	E130003G02Rik	0.000532	2.0517626	up
ASMM9PARTA000492		0.006650831	2.5813942	up
ASMM9PARTA008992		0.002741891	2.1566684	up
ASMM9PARTA007865		0.003608599	2.932615	up
ASMM9PARTA011901	Gm11623	0.012665474	2.1208858	up
ASMM9PARTA007417		0.003090163	5.209807	up
ASMM9PARTA013190	Gm7386	0.006763252	3.4498134	up
ASMM9PARTA008581		0.007435879	2.453574	up
MM9LINCRNAEXON10455-_P1	mouselincRNA1287	0.02423389	2.5882087	up
ASMM9PARTA003640		0.004941439	4.5171905	up
ASMM9PARTA046949	AK150110	0.010360972	2.2313983	up
mouselincRNA0133+_P1	mouselincRNA0133	0.007893923	2.19937	up
mouselincRNA1546-_P1	mouselincRNA1546	0.02694875	2.0435512	up
ASMM9PARTA010114	Gm11041	0.000000161	4.414594	up
ASMM9PARTA001249		0.0003	3.18866	up
ASMM9PARTA018903	Smok3c	0.000588	2.2865016	up
MM9LINCRNAEXON11031-_P1	mouselincRNA0914	0.001849649	2.8037627	up
BB869594_P1	mouselincRNA1003	0.035452735	3.4142158	up
ASMM9PARTA013835	Gm12780	0.009454524	2.1172101	up
ASMM9PARTA008507		0.04051666	2.1475964	up
ASMM9PARTA017913	Gm12440	0.000415	2.38328	up
ASMM9PARTA048877	AK076706	0.001909963	2.009778	up
ASMM9PARTA007699		0.011515025	2.5529652	up
ASMM9PARTA048158	AK137010	0.000183	2.2145581	up

ASMM9PARTA013980	2810403D21Rik	0.004927495	3.7204018	up
ASMM9PARTA048349	AK018679	0.000571	2.664696	up
MM9LINCRNAEXON10681-_P1	mouselincRNA1110	0.000023	3.1860194	up
ASMM9PARTA047160	Slc38a6	0.019899402	5.269713	up
ASMM9PARTA047160	Slc38a6	0.019899402	5.269713	up
ASMM9PARTA015335	B230314M03Rik	0.036722288	2.7376027	up
MM9LINCRNAEXON11226-_P1	mouselincRNA0717	0.004714525	3.757954	up
ASMM9PARTA005805		0.001833606	2.413141	up
MM9LINCRNAEXON11010-_P1	mouselincRNA0889	0.017116968	2.1312296	up
humanlincRNA0409-_P1	humanlincRNA0409	0.028668752	2.1197705	up
ASMM9PARTA013023	Gm15383	0.008592616	2.2315118	up
ASMM9PARTA006284		0.023139529	3.1474016	up
mouselincRNA0491-_P1	mouselincRNA0491	0.009306679	2.387591	up
ASMM9PARTA004071		0.00888211	4.276418	up
ASMM9PARTA005086		0.019149791	2.1496463	up
ASMM9PARTA014856	1810059H22Rik	0.010172333	2.085558	up
humanlincRNA0970-_P1	humanlincRNA0970	0.026733814	2.0537314	up
ASMM9PARTA018240	7SK.318	0.000271	5.1898184	up
ASMM9PARTA000150		0.04165382	2.678476	up
ASMM9PARTA047628	AK143833	0.000334	2.9296582	up
ASMM9PARTA011791	Gm11921	0.03761249	2.2298455	up
ASMM9PARTA047844	AK015279	0.00081	2.1478488	up
ASMM9PARTA045504	4930583H14Rik	0.016456593	2.9131455	up
ASMM9PARTA015426	Gm14092	0.020735871	4.9228477	up
ASMM9PARTA015426	Gm14092	0.020735871	4.9228477	up
ASMM9PARTA049572	AK016731	0.0000165	3.2234983	up
MM9LINCRNAEXON10775+_P1	mouselincRNA1143	0.004435093	2.906301	up
mouselincRNA0303-_P1	mouselincRNA0303	0.004889886	2.658487	up
ASMM9PARTA050633	AK006412	0.004353627	2.5914717	up
ASMM9PARTA001180		0.009087118	3.5569837	up
ASMM9PARTA008433		0.001083974	2.5229979	up
ASMM9PARTA008412		0.03948813	2.569691	up
ASMM9PARTA007252		0.004828085	2.5939534	up
ASMM9PARTA003334		0.033744946	3.6882799	up
ASMM9PARTA009988	4930473A02Rik	0.004919326	2.2651217	up
ASMM9PARTA009988	4930473A02Rik	0.004919326	2.2651217	up
ASMM9PARTA009988	4930473A02Rik	0.004919326	2.2651217	up
CUST_322_PI426073487	uc.324	0.013892835	3.0091732	up
CUST_322_PI426073487	uc.324	0.013892835	3.0091732	up
CUST_850_PI426073487	uc.371	0.027196558	2.030118	up
CUST_850_PI426073487	uc.371	0.027196558	2.030118	up
CUST_850_PI426073487	uc.371	0.027196558	2.030118	up
ASMM9PARTA005626		0.001491764	3.9304707	up
mouselincRNA0413+_P1	mouselincRNA0413	0.003593903	2.5391424	up
humanlincRNA1576+_P1	humanlincRNA1576	0.031454343	2.2732453	up
ASMM9PARTA007884		0.025023691	3.7976923	up
MM9LINCRNAEXON10053+_P1	mouselincRNA1654	0.005884349	2.1847844	up
ASMM9PARTA013524	Gm16407	0.021851366	2.1022146	up
ASMM9PARTA006409		0.014835628	2.49838	up
ASMM9PARTA047151	MAIR-IIa	0.000873	3.2963796	up
ASMM9PARTA005747		0.000283	2.0381892	up
ASMM9PARTA010654	Gm12057	0.009653101	2.1286013	up
MM9LINCRNAEXON11597-_P1	mouselincRNA0422	0.00028	3.4359727	up
MM9LINCRNAEXON10991+_P1		0.0000977	3.246831	up
MM9LINCRNAEXON10788-_P1	mouselincRNA1158	0.003637178	3.1387908	up
ASMM9PARTA002929		0.007812527	4.445197	up
ASMM9PARTA046040	4931406H21Rik	0.006116352	2.2583747	up

ASMM9PARTA006147		0.008449999	2.7044325	up
ASMM9PARTA014539	Gm16095	0.04013583	2.0622075	up
ASMM9PARTA015438	B430212C06Rik	0.023355633	4.033507	up
mouselincRNA0935+_P1	mouselincRNA0935	0.002627854	2.3365896	up
mouselincRNA0953+_P1	mouselincRNA0953	0.004046383	2.506643	up
ASMM9PARTA050563	AK039589	0.027110359	2.4989367	up
ASMM9PARTA001301		0.004140169	3.808549	up
ASMM9PARTA016462	Gm14635	0.020431722	2.5554345	up
ASMM9PARTA014015	Gm13793	0.001002149	3.132261	up
MM9LINCRNAEXON11361-_P1	mouselincRNA0586	0.002789273	4.376738	up
ASMM9PARTA017005	Gm14340	0.008188108	2.333206	up
ASMM9PARTA005615		0.011714377	2.5922735	up
ASMM9PARTA018378	4930486I03Rik	0.01697491	2.1486802	up
ASMM9PARTA008221		0.000494	2.7395587	up
ASMM9PARTA002874		0.000000781	19.095455	up
ASMM9PARTA003592		0.000000529	2.2310605	up
ASMM9PARTA006865		0.000325	4.007223	up
CF585596_P1	humanlincRNA0369	0.00074	2.4564066	up
CF585596_P1	humanlincRNA0369	0.00074	2.4564066	up
CF585596_P1	humanlincRNA0369	0.00074	2.4564066	up
CF585596_P1	humanlincRNA0369	0.00074	2.4564066	up
CF585596_P1	humanlincRNA0369	0.00074	2.4564066	up
CF585596_P1	humanlincRNA0369	0.00074	2.4564066	up
MM9LINCRNAEXON11347-_P1	mouselincRNA0566	0.005428209	2.718914	up
ASMM9PARTA003335		0.000189	2.6276195	up
ASMM9PARTA004660		0.007580058	2.577663	up
ASMM9PARTA005963		0.00133358	6.5122476	up
ASMM9PARTA005963		0.00133358	6.5122476	up
ASMM9PARTA005238		0.000315	4.2467995	up
ASMM9PARTA004795		0.009538312	3.4787257	up
ASMM9PARTA010220	Gm14803	0.002420924	2.1448472	up
ASMM9PARTA015594	D430001F17Rik	0.0437562	2.0228877	up
mouselincRNA0295+_P1	mouselincRNA0295	0.041968614	5.0190034	up
ASMM9PARTA001200		0.017699424	2.5728662	up
ASMM9PARTA049623	Rzf	0.007529846	2.3152401	up
ASMM9PARTA049623	Rzf	0.007529846	2.3152401	up
CF968634_P1	humanlincRNA0134	0.002869968	3.297725	up
ASMM9PARTA005100		0.020054754	4.2711105	up
ASMM9PARTA018143	7SK.319	0.042404737	2.4173079	up
ASMM9PARTA006359		0.00000007	16.216913	up
AF282719_P1	humanlincRNA0719	0.000772	7.0957775	up
AF282719_P1	humanlincRNA0719	0.000772	7.0957775	up
AF282719_P1	humanlincRNA0719	0.000772	7.0957775	up
AF282719_P1	humanlincRNA0719	0.000772	7.0957775	up
AF282719_P1	humanlincRNA0719	0.000772	7.0957775	up
AF282719_P1	humanlincRNA0719	0.000772	7.0957775	up
AF282719_P1	humanlincRNA0719	0.000772	7.0957775	up
AF282719_P1	humanlincRNA0719	0.000772	7.0957775	up
ASMM9PARTA048810	AK042686	0.001606488	4.311061	up
ASMM9PARTA016490	Gm15850	0.043831065	2.161194	up
humanlincRNA2404-_P1	humanlincRNA2404	0.003765507	3.1941075	up
ASMM9PARTA014438	Pisd-ps2	0.0000295	3.9225237	up
ASMM9PARTA016412	Gm11337	0.011172816	3.2563884	up
ASMM9PARTA003273		0.000961	2.1440165	up
ASMM9PARTA015815	Gm15983	0.005759083	2.0799112	up
humanlincRNA0170-_P1	humanlincRNA0170	0.03513029	3.0277035	up
ASMM9PARTA006224		0.004209496	2.4723294	up

ASMM9PARTA006230		0.003181617	2.6246455	up
MM9LINCRNAEXON10995+_P1		0.000219	2.2065775	up
ASMM9PARTA001251		0.032343972	2.0799944	up
ASMM9PARTA013403	Gm13108	0.002014019	3.0960884	up
ASMM9PARTA011780	Gm11305	0.00735963	3.0413704	up
ASMM9PARTA015839	Gm12299	0.025767269	2.2247999	up
MM9LINCRNAEXON11718+_P1	mouselincRNA0341	0.004537967	2.886666	up
ASMM9PARTA003242		0.0185691	2.3176277	up
ASMM9PARTA006070		0.00086	5.218045	up
humanlincRNA1475-_P1	humanlincRNA1475	0.013494893	2.3496206	up
ASMM9PARTA003922		0.004797854	2.1118584	up
ASMM9PARTA003922		0.004797854	2.1118584	up
ASMM9PARTA003922		0.004797854	2.1118584	up
ASMM9PARTA003922		0.004797854	2.1118584	up
ASMM9PARTA003546		0.012164822	2.9510112	up
ASMM9PARTA013870	7SK.46	0.00653718	3.0775836	up
ASMM9PARTA000913		0.01030242	2.28674	up
mouselincRNA0303+_P1	mouselincRNA0303	0.03366837	2.0809026	up
ASMM9PARTA004587		0.020102844	2.3598976	up
MM9LINCRNAEXON10983-_P1	mouselincRNA0873	0.014677032	2.0352643	up
ASMM9PARTA008175		0.016396912	2.9928968	up
MM9LINCRNAEXON10071-_P1	mouselincRNA1506	0.016131137	3.0533679	up
MM9LINCRNAEXON11571+_P1	mouselincRNA0391	0.003376605	2.921329	up
ASMM9PARTA003751		5.75E-08	9.076029	up
ASMM9PARTA011562	Gm15039	0.000397	3.1455555	up
MM9LINCRNAEXON10841-_P1	mouselincRNA0984	0.030122142	2.888943	up
ASMM9PARTA003610		0.02455914	2.6326885	up
mouselincRNA0327+_P1	mouselincRNA0327	0.003797996	3.155185	up
MM9LINCRNAEXON10848+_P1	mouselincRNA0988	0.011432783	4.513205	up
humanlincRNA1007-_P1	humanlincRNA1007	0.016611377	3.5594263	up
CUST_124_PI426409190	AC142215.1	0.0000134	2.7843447	up
ASMM9PARTA004293		0.004630854	2.7189882	up
ASMM9PARTA004768		0.001119941	2.5852275	up
ASMM9PARTA049182	AK196015	0.006319387	2.0723906	up
ASMM9PARTA049182	AK196015	0.006319387	2.0723906	up
ASMM9PARTA045636	Ngrn	0.0000968	2.0350733	up
humanlincRNA1148-_P1	humanlincRNA1148	0.027692938	2.3294423	up
MM9LINCRNAEXON11076-_P1		0.000333	2.5105052	up
ASMM9PARTA008966		0.022396708	2.323597	up
humanlincRNA1751+_P1	humanlincRNA1751	0.049138177	2.884519	up
CJ270168_P1	mouselincRNA0215	0.019876016	2.1509004	up
ASMM9PARTA047903	AK039376	0.008608301	2.372991	up
ASMM9PARTA051767	AK042998	0.000551	2.8822157	up
ASMM9PARTA005065		0.01467525	7.6537714	up
MM9LINCRNAEXON12070+_P1	mouselincRNA0095	0.020558814	3.234725	up
ASMM9PARTA014527	Gm15581	0.013598269	3.6646116	up
ASMM9PARTA014527	Gm15581	0.013598269	3.6646116	up
CR518586_P1	mouselincRNA0187	0.025893642	3.7312312	up
ASMM9PARTA006133		0.03523575	3.1345823	up
ASMM9PARTA007358		0.0000675	2.2560449	up
ASMM9PARTA007105		0.031383168	2.376892	up
ASMM9PARTA011789	Gm12856	0.000104	2.4299927	up
ASMM9PARTA005998		0.01808093	2.2901597	up
ASMM9PARTA049586	Il7	0.009178804	2.281167	up
humanlincRNA2361+_P1	humanlincRNA2361	0.009182313	2.2967227	up
CUST_537_PI426073487	uc.58	0.000262	2.276107	up
ASMM9PARTA048789	AK196959	0.025871867	2.508649	up

ASMM9PARTA046723	AK087691	0.031588674	3.0785332	up
ASMM9PARTA004883		0.0000819	5.482869	up
ASMM9PARTA004883		0.0000819	5.482869	up
ASMM9PARTA049416	Slco4a1	0.017494341	2.0650449	up
ASMM9PARTA007774		0.002636387	2.146709	up
MM9LINCRNAEXON12111-_P1	mouselincRNA0122	0.000387	2.0534098	up
ASMM9PARTA019467	RP24-318H2.3	0.0195198	3.818625	up
CN686219_P1	mouselincRNA0474	0.000241	4.338695	up
CN686219_P1	mouselincRNA0474	0.000241	4.338695	up
CN686219_P1	mouselincRNA0474	0.000241	4.338695	up
CN686219_P1	mouselincRNA0474	0.000241	4.338695	up
CN686219_P1	mouselincRNA0474	0.000241	4.338695	up
CN686219_P1	mouselincRNA0474	0.000241	4.338695	up
CN686219_P1	mouselincRNA0474	0.000241	4.338695	up
CN686219_P1	mouselincRNA0474	0.000241	4.338695	up
CN686219_P1	mouselincRNA0474	0.000241	4.338695	up
CN686219_P1	mouselincRNA0474	0.000241	4.338695	up
ASMM9PARTA000820		0.007960998	2.4585204	up
ASMM9PARTA000820		0.007960998	2.4585204	up
humanlincRNA0518+_P1	humanlincRNA0518	0.001728922	2.3066185	up
AK145365_P1	mouselincRNA1288	0.022547744	2.299596	up
MM9LINCRNAEXON11321+_P1	mouselincRNA0524	0.020673875	2.8000972	up
ASMM9PARTA050637	smarp	0.000395	2.9558716	up
ASMM9PARTA009217		0.003658279	4.6805663	up
ASMM9PARTA049997	AK090251	0.000592	2.1281261	up
ASMM9PARTA049997	AK090251	0.000592	2.1281261	up
ASMM9PARTA000801		0.001110978	2.623008	up
CUST_118_PI426073487	uc.120	0.014781382	2.306064	up
CUST_118_PI426073487	uc.120	0.014781382	2.306064	up
ASMM9PARTA007224		0.030296622	2.900314	up
BX636194_P1	mouselincRNA1032	0.000592	3.4412906	up
ASMM9PARTA045201	Gm10565	0.036532503	2.4264839	up
ASMM9PARTA050227	BC028660	0.02773815	2.5893104	up
ASMM9PARTA017822	7SK.135	0.019660747	3.0378206	up
ASMM9PARTA009325		0.000021	3.2174046	up
ASMM9PARTA009325		0.000021	3.2174046	up
ASMM9PARTA009325		0.000021	3.2174046	up
MM9LINCRNAEXON12056-_P1	mouselincRNA0073	0.001231018	3.119503	up
ASMM9PARTA013963	6030471H07Rik	0.004164994	2.2393048	up
ASMM9PARTA014781	Gm16201	0.008633323	2.3121636	up
ASMM9PARTA002895		0.000149	2.3540666	up
MM9LINCRNAEXON11730-_P1		0.000000132	8.980893	up
ASMM9PARTA000137		0.000273	3.074779	up
MM9LINCRNAEXON11978+_P1	mouselincRNA0222	0.002160778	2.204473	up
MM9LINCRNAEXON11855-_P1		0.006662581	2.4833117	up
MM9LINCRNAEXON11855-_P1		0.006662581	2.4833117	up
MM9LINCRNAEXON11855-_P1		0.006662581	2.4833117	up
ASMM9PARTA017917	Gm16143	0.000922	5.5009575	up
MM9LINCRNAEXON10903-_P1		0.00000597	2.737371	up
ASMM9PARTA012091	Gm12262	0.004504351	2.108258	up
ASMM9PARTA014147	E130006D01Rik	0.043600935	2.0462427	up
humanlincRNA0574+_P1	humanlincRNA0574	0.008705057	2.7942004	up
ASMM9PARTA004140		0.027165925	2.977007	up
ASMM9PARTA008094		0.014263074	3.1741056	up
ASMM9PARTA013476	Cxcl11	0.04881224	2.052698	up
ASMM9PARTA013476	Cxcl11	0.04881224	2.052698	up
mouselincRNA1231-_P1	mouselincRNA1231	0.006263476	2.3302102	up

ASMM9PARTA004601		0.001504389	2.2137654	up
ASMM9PARTA009446	Mageb16-ps2	0.03255596	2.0165014	up
ASMM9PARTA009446	Mageb16-ps2	0.03255596	2.0165014	up
ASMM9PARTA015200	1700081H22Rik	0.0128969	3.2490816	up
MM9LINCRNAEXON11387-_P1	mouselincRNA0595	0.006707311	2.5604918	up
ASMM9PARTA015086	4930432B10Rik	0.011798902	2.2729568	up
ASMM9PARTA015086	4930432B10Rik	0.011798902	2.2729568	up
ASMM9PARTA017308	Gm12472	0.018170025	2.4549687	up
ASMM9PARTA045906	Gm7550	0.000251	2.2035732	up
ASMM9PARTA050898	AK040659	0.014976711	2.0857913	up
ASMM9PARTA051534	AK010793	0.047703233	2.4074008	up
ASMM9PARTA000900		0.000169	2.0460937	up
ASMM9PARTA003847		0.029512828	2.126018	up
ASMM9PARTA045746	G530011O06Rik	4.52E-08	3.7382073	up
ASMM9PARTA045746	G530011O06Rik	4.52E-08	3.7382073	up
CUST_600_PI426073487	uc.121	0.01564907	3.245898	up
ASMM9PARTA016400	Gm12204	0.006816887	2.7718306	up
ASMM9PARTA006996		0.000961	2.5933619	up
ASMM9PARTA000944		0.01944736	2.3564873	up
ASMM9PARTA046813	AK041795	0.007389697	2.3447719	up
ASMM9PARTA004073		0.01059565	2.0597837	up
ASMM9PARTA008655		0.001801538	2.7565634	up
ASMM9PARTA008655		0.001801538	2.7565634	up
ASMM9PARTA008655		0.001801538	2.7565634	up
ASMM9PARTA003440		0.0000222	3.326313	up
ASMM9PARTA046385	AK137370	0.000331	2.1035595	up
ASMM9PARTA014524	Gm16144	0.0168259	2.635315	up
ASMM9PARTA015108	AV039307	0.000213	2.0705316	up
ASMM9PARTA015108	AV039307	0.000213	2.0705316	up
ASMM9PARTA015108	AV039307	0.000213	2.0705316	up
ASMM9PARTA015108	AV039307	0.000213	2.0705316	up
ASMM9PARTA001481		0.007227806	4.7364945	up
ASMM9PARTA047595	AK035977	0.0000051	3.3314865	up
ASMM9PARTA046895	slc43a2	0.020435391	2.0050123	up
ASMM9PARTA046895	slc43a2	0.020435391	2.0050123	up
ASMM9PARTA046895	slc43a2	0.020435391	2.0050123	up
ASMM9PARTA005435		0.000000256	18.015913	up
ASMM9PARTA048214	AK170928	0.005106441	2.304618	up
ASMM9PARTA048214	AK170928	0.005106441	2.304618	up
ASMM9PARTA048214	AK170928	0.005106441	2.304618	up
ASMM9PARTA010117	Olfr833-ps1	0.0430436	2.5003648	up
ASMM9PARTA050677	AK085609	0.03988026	2.3959594	up
ASMM9PARTA000395		0.045012746	2.5709462	up
ASMM9PARTA000399		0.000328	2.6425545	up
ASMM9PARTA000595		0.020333577	2.0376506	up
ASMM9PARTA013771	2810408I11Rik	0.012482183	3.0549285	up
ASMM9PARTA014536	Gm11532	0.010399843	2.4161506	up
ASMM9PARTA018595	Gm15898	0.022621483	2.0472808	up
MM9LINCRNAEXON11454-_P1	mouselincRNA0481	0.013212452	3.1834896	up
ASMM9PARTA005235		0.001210142	3.3483496	up
ASMM9PARTA005235		0.001210142	3.3483496	up
ASMM9PARTA005235		0.001210142	3.3483496	up
ASMM9PARTA015476	Hoxa11as	0.0000104	3.8943572	up
CK378914_P1	humanlincRNA1553	0.034202967	2.6908047	up
ASMM9PARTA001502		0.003783918	2.0007417	up
ASMM9PARTA001502		0.003783918	2.0007417	up
ASMM9PARTA004458		0.004508045	2.5133686	up

mouselincRNA1171+_P1	mouselincRNA1171	0.032411795	2.5514	up
ASMM9PARTA012488	Gm11283	0.020013498	2.6281354	up
ASMM9PARTA049866	AK052888	0.005904906	2.0168877	up
mouselincRNA0238-_P1	mouselincRNA0238	0.000426	2.984266	up
ASMM9PARTA047894	myo 10	0.003380551	2.3656745	up
BB791535_P1	humanlincRNA2428	0.000016	2.3109844	up
ASMM9PARTA002051		0.00000194	8.409327	up
ASMM9PARTA051152	Lrrc36	0.008097591	2.061716	up
ASMM9PARTA051152	Lrrc36	0.008097591	2.061716	up
ASMM9PARTA051152	Lrrc36	0.008097591	2.061716	up
ASMM9PARTA049329	AK044780	0.010201347	5.548784	up
ASMM9PARTA010668	Gm11905	0.040006787	2.5514011	up
AI503337_P1	mouselincRNA0302	0.004814492	3.1701016	up
ASMM9PARTA044984	Dio3os	0.002766666	3.090687	up
ASMM9PARTA011429	Gm14109	0.0000683	2.9777064	up
ASMM9PARTA047707	AK036194	0.0000249	3.31411	up
ASMM9PARTA001427		0.043376524	2.8309004	up
ASMM9PARTA001427		0.043376524	2.8309004	up
ASMM9PARTA007597		0.000891	2.0801847	up
ASMM9PARTA009159		0.001721845	2.0417426	up
ASMM9PARTA048531	NR_003519	0.0000666	3.0680885	up
ASMM9PARTA047171	AK012841	0.0000459	2.8754818	up
ASMM9PARTA047171	AK012841	0.0000459	2.8754818	up
ASMM9PARTA051314	AK017891	0.003712478	2.5869553	up
ASMM9PARTA006005		0.006116074	4.829948	up
ASMM9PARTA016541	Zfp133-ps	0.009674044	3.043616	up
ASMM9PARTA048168	AK157795	0.000128	2.652638	up
ASMM9PARTA048168	AK157795	0.000128	2.652638	up
ASMM9PARTA006553		0.038864736	2.5163722	up
ASMM9PARTA003897		0.009810638	2.1432943	up
ASMM9PARTA050128	AK019745	0.00062	2.0429552	up
ASMM9PARTA010177	Gm6973	0.002415242	2.8041196	up
ASMM9PARTA049540	AK076817	0.012002505	2.6666303	up
humanlincRNA2366+_P1	humanlincRNA2366	0.019007077	3.577876	up
ASMM9PARTA010395	Gm13566	0.028192304	2.4371617	up
ASMM9PARTA014507	Gm15941	0.011355937	2.6680515	up
ASMM9PARTA014507	Gm15941	0.011355937	2.6680515	up
ASMM9PARTA014507	Gm15941	0.011355937	2.6680515	up
ASMM9PARTA014507	Gm15941	0.011355937	2.6680515	up
ASMM9PARTA045163	Asb7	0.03220917	2.2842948	up
ASMM9PARTA045163	Asb7	0.03220917	2.2842948	up
ASMM9PARTA045163	Asb7	0.03220917	2.2842948	up
ASMM9PARTA000711		0.007415217	2.1036763	up
ASMM9PARTA011226	Gm14996	0.000197	13.965036	up
AA265991_P1	mouselincRNA1273	0.000329	3.5148315	up
ASMM9PARTA017541	A230087F16Rik	0.000288	4.1284275	up
ASMM9PARTA006006		5.09E-09	6.061923	up
ASMM9PARTA051308	AK038653	0.000921	4.2577143	up
ASMM9PARTA015795	3110053B16Rik	0.005779855	9.20947	up
ASMM9PARTA045251	4930583K01Rik	0.038328867	2.0776012	up
ASMM9PARTA051637	Fxy	0.04784923	2.491814	up
ASMM9PARTA051637	Fxy	0.04784923	2.491814	up
ASMM9PARTA007173		0.005225923	2.351652	up
ASMM9PARTA004652		0.04397347	2.339422	up
ASMM9PARTA019226	Gm16759	0.012134996	2.3422337	up
ASMM9PARTA016310	Gm12868	0.001711269	2.8343163	up
ASMM9PARTA049238	AK029171	0.00629853	5.3820868	up

AA791803_P1	mouselincRNA0980	0.004229304	2.4797354	up
ASMM9PARTA003122		0.0000136	4.2286463	up
humanlincRNA0573-_P1	humanlincRNA0573	0.00521501	3.4760077	up
CK377992_P1	humanlincRNA1344	0.00982045	2.4746296	up
ASMM9PARTA002446		0.007966969	3.9831717	up
ASMM9PARTA004214		0.00000442	2.7079759	up
ASMM9PARTA044945	Pisd-ps2	0.000000698	2.59912	up
CUST_919_PI426073487	uc.440	0.00147135	2.01403	up
ASMM9PARTA045585	Gm839	0.000568	2.2649157	up
ASMM9PARTA016669	Gm16211	0.036198933	2.4129307	up
ASMM9PARTA005122		0.0000117	2.5832894	up
ASMM9PARTA047644	AK035875	0.0000127	2.6074126	up
ASMM9PARTA018523	Airn	0.0000714	8.554767	up
MM9LINCRNAEXON10694-_P1	mouselincRNA1113	0.001748241	2.7544942	up
ASMM9PARTA015549	A830036E02Rik	0.00854246	5.175746	up
MM9LINCRNAEXON10299-_P1	mouselincRNA1501	0.0017595	2.4022002	up
ASMM9PARTA047559	AK042724	0.000018	3.1664455	up
ASMM9PARTA008566		0.024145622	2.2763615	up
ASMM9PARTA008566		0.024145622	2.2763615	up
ASMM9PARTA051664	Trip4	0.001610819	3.1623545	up
ASMM9PARTA051664	Trip4	0.001610819	3.1623545	up
ASMM9PARTA051664	Trip4	0.001610819	3.1623545	up
ASMM9PARTA003375		0.000208	2.03154	up
mouselincRNA0963-_P1	mouselincRNA0963	0.041240107	2.8679602	up
mouselincRNA0436+_P1	mouselincRNA0436	0.041452058	3.3339229	up
ASMM9PARTA019298	AC157278.1	0.005295564	2.7677095	up
ASMM9PARTA049975	AK043904	0.00000966	2.6992455	up
ASMM9PARTA004654		0.010756833	2.3001642	up
humanlincRNA1385-_P1	humanlincRNA1385	0.0000278	2.7191672	up
ASMM9PARTA044892	E430016F16Rik	0.012964277	2.7999663	up
CUST_522_PI426073487	uc.43	0.04265385	2.3163753	up
ASMM9PARTA051782	AK142198	0.004491635	2.5334752	up
ASMM9PARTA051782	AK142198	0.004491635	2.5334752	up
ASMM9PARTA051782	AK142198	0.004491635	2.5334752	up
ASMM9PARTA051782	AK142198	0.004491635	2.5334752	up
ASMM9PARTA051782	AK142198	0.004491635	2.5334752	up
ASMM9PARTA003277		0.005563146	2.3854492	up
ASMM9PARTA047146	AK087290	0.02601443	2.4732277	up
ASMM9PARTA047146	AK087290	0.02601443	2.4732277	up
ASMM9PARTA006102		0.015191331	2.2511697	up
ASMM9PARTA016340	BC006965	0.0000523	4.867762	up
ASMM9PARTA047100	AK005722	0.036922194	2.3813727	up
ASMM9PARTA047100	AK005722	0.036922194	2.3813727	up
ASMM9PARTA049247	AK040741	0.009180733	3.4751656	up
ASMM9PARTA001889		0.0000573	3.1273277	up
ASMM9PARTA005953		0.016811585	3.20891	up
ASMM9PARTA003457		0.000000134	2.1274035	up
ASMM9PARTA003457		0.000000134	2.1274035	up
ASMM9PARTA003457		0.000000134	2.1274035	up
ASMM9PARTA010228	Gm10827	0.000221	3.3223596	up
MM9LINCRNAEXON10474+_P1	mouselincRNA1297	0.017352384	2.950174	up
ASMM9PARTA005229		0.000051	9.968039	up
ASMM9PARTA050840	AK050360	0.000188	2.1969428	up
ASMM9PARTA010591	Gm12131	0.012252663	2.3931608	up
ASMM9PARTA014625	Gm16662	0.005211503	2.5672529	up
ASMM9PARTA014625	Gm16662	0.005211503	2.5672529	up
ASMM9PARTA015228	Gm12576	0.045517977	2.0414014	up

DT905990_P1	humanlincRNA0233	0.006708864	2.0679245	up
CUST_178_PI426073487	uc.180	0.047720756	2.1331503	up
ASMM9PARTA016151	Gm15704	0.00034	2.0066426	up
MM9LINCRNAEXON10606+_P1	mouselincRNA1220	0.010221294	2.1154242	up
ASMM9PARTA017746	Gm12406	0.015883213	2.1940188	up
MM9LINCRNAEXON11100-_P1		0.0000266	2.758228	up
ASMM9PARTA003989		0.006342701	2.75745	up
ASMM9PARTA050047	Pon2	0.012031178	2.053553	up
ASMM9PARTA002287		0.026253045	2.8593476	up
ASMM9PARTA050636	AK036632	0.011405653	2.1171608	up
ASMM9PARTA003909		0.00798211	3.1412895	up
MM9LINCRNAEXON10533-_P1	mouselincRNA1174	0.012461234	3.108678	up
ASMM9PARTA048456	AK156750	0.03064462	2.0129848	up
ASMM9PARTA048456	AK156750	0.03064462	2.0129848	up
ASMM9PARTA014677	A830036E02Rik	0.000435	45.121548	up
ASMM9PARTA016435	Gm16598	0.035210583	2.7732875	up
ASMM9PARTA016435	Gm16598	0.035210583	2.7732875	up
ASMM9PARTA051330	Ttc13	0.00000107	2.935716	up
ASMM9PARTA046346	AK009784	0.008528904	2.3359718	up
ASMM9PARTA003355		0.011357634	2.2749739	up
ASMM9PARTA005787		0.013401753	2.6387243	up
ASMM9PARTA005787		0.013401753	2.6387243	up
ASMM9PARTA005787		0.013401753	2.6387243	up
ASMM9PARTA006188		0.000499	2.6555064	up
ASMM9PARTA006188		0.000499	2.6555064	up
ASMM9PARTA014359	Gm12648	0.024236048	2.649923	up
ASMM9PARTA007503		0.047398217	5.4163795	up
ASMM9PARTA013921	Gm15449	0.012926074	2.7023711	up
ASMM9PARTA048287	AK132606	0.000000579	2.4504342	up
ASMM9PARTA018983	AL606528.1	0.00045	3.7947605	up
MM9LINCRNAEXON10522+_P1	mouselincRNA1351	0.01097882	2.6133952	up
ASMM9PARTA049755	AK006706	0.049701747	2.6098168	up
ASMM9PARTA007292		0.000364	2.618107	up
ASMM9PARTA007292		0.000364	2.618107	up
ASMM9PARTA007292		0.000364	2.618107	up
MM9LINCRNAEXON11968+_P1		0.014316526	2.162776	up
ASMM9PARTA046016	Olfr856-ps1	0.002731215	2.6084263	up
CUST_153_PI426073487	uc.155	0.023007892	2.1121387	up
ASMM9PARTA004570		0.027941186	2.5921009	up
ASMM9PARTA009000		0.000242	3.0044978	up
ASMM9PARTA051692	AK019053	0.000123	2.651416	up
ASMM9PARTA051692	AK019053	0.000123	2.651416	up
ASMM9PARTA047197	AK046721	0.000707	2.1079693	up
ASMM9PARTA047197	AK046721	0.000707	2.1079693	up
humanlincRNA0891+_P1	humanlincRNA0891	0.03254211	2.1048813	up
MM9LINCRNAEXON11798-_P1	mouselincRNA0252	0.023145236	2.0043	up
CF586746_P1	humanlincRNA0707	0.002633335	3.4305167	up
CF586746_P1	humanlincRNA0707	0.002633335	3.4305167	up
CF586746_P1	humanlincRNA0707	0.002633335	3.4305167	up
CF586746_P1	humanlincRNA0707	0.002633335	3.4305167	up
CF586746_P1	humanlincRNA0707	0.002633335	3.4305167	up
CF586746_P1	humanlincRNA0707	0.002633335	3.4305167	up
CF586746_P1	humanlincRNA0707	0.002633335	3.4305167	up
CF586746_P1	humanlincRNA0707	0.002633335	3.4305167	up
CF586746_P1	humanlincRNA0707	0.002633335	3.4305167	up
CF586746_P1	humanlincRNA0707	0.002633335	3.4305167	up
ASMM9PARTA046691	AK158612	0.000779	11.221282	up

mouselincRNA0078-_P1	mouselincRNA0078	0.036236666	2.1452622	up
ASMM9PARTA013817	Gm15247	0.0000614	3.0098472	up
ASMM9PARTA013817	Gm15247	0.0000614	3.0098472	up
ASMM9PARTA048242	AK139359	0.000411	2.1412673	up
ASMM9PARTA008846		0.002321065	2.3002503	up
ASMM9PARTA015865	1700047F07Rik	0.002786112	3.2242582	up
ASMM9PARTA010290	Rps2-ps13	0.00000143	2.0094447	up
ASMM9PARTA003855		0.000803	2.6637871	up
ASMM9PARTA012460	Gm12876	0.003103023	2.1190248	up
ASMM9PARTA051121	Lrrc49	0.00000666	20.39547	up
ASMM9PARTA051121	Lrrc49	0.00000666	20.39547	up
ASMM9PARTA051121	Lrrc49	0.00000666	20.39547	up
ASMM9PARTA002375		0.000000269	28.71547	up
ASMM9PARTA003040		0.006775585	3.4083211	up
ASMM9PARTA051093	AK137397	0.033166505	2.1031444	up
ASMM9PARTA018971	AC165246.1	0.000334	4.7090154	up
ASMM9PARTA048621	Akd2	0.033046994	2.0939624	up
ASMM9PARTA004055		1.03E-08	57.48466	up
ASMM9PARTA050230	AK131834	0.0177835	3.267112	up
ASMM9PARTA047992	AK043393	0.0000131	3.4294982	up
ASMM9PARTA048439	BC023719	0.007264353	2.9192505	up
humanlincRNA1152+_P1	humanlincRNA1152	0.000165	2.4595704	up
ASMM9PARTA004548		0.000128	2.3919067	up
ASMM9PARTA047783	Ugcgl2	0.002353273	2.3200574	up
ASMM9PARTA003032		0.024736745	2.3304965	up
ASMM9PARTA003032		0.024736745	2.3304965	up
ASMM9PARTA003032		0.024736745	2.3304965	up
ASMM9PARTA011540	Pisd-ps1	0.0000007	2.758198	up
ASMM9PARTA002372		0.002291812	2.5871181	up
ASMM9PARTA018126	Gm11754	0.000905	3.2527559	up
ASMM9PARTA044960	BC006965	0.0000205	51.99762	up
ASMM9PARTA007750		0.000242	4.8765545	up
MM9LINCRNAEXON10121-_P1	mouselincRNA1557	0.03973443	2.4058142	up
ASMM9PARTA018650	Gm15562	0.000798	2.2684283	up
ASMM9PARTA018650	Gm15562	0.000798	2.2684283	up
ASMM9PARTA000537		0.00000377	4.017122	up
ASMM9PARTA050623	Kox-1	0.00758429	2.2887216	up
ASMM9PARTA049446	AK031919	0.001327318	2.0094407	up
ASMM9PARTA000684		0.00000023	2.414727	up
MM9LINCRNAEXON10518+_P1	mouselincRNA1347	0.00000652	8.44547	up
ASMM9PARTA045371	5033406O09Rik	0.006351451	4.404478	up
ASMM9PARTA047885	AK051464	0.00000465	3.3569086	up
ASMM9PARTA046971	AK138212	0.014335649	3.9975398	up
ASMM9PARTA001331		0.016304793	2.8768473	up
ASMM9PARTA013646	D230017M19Rik	0.004561038	3.5416203	up
ASMM9PARTA010437	Cd47	0.0000003	3.7367325	up
ASMM9PARTA045591	Gm11110	0.005692962	2.995289	up
ASMM9PARTA004077		0.0000266	2.2475781	up
ASMM9PARTA002181		0.00000226	3.4352078	up
ASMM9PARTA019401	9430021M05Rik	0.0000468	2.5355873	up
BC040767_P1	humanlincRNA1200	0.000000165	61.509308	up
ASMM9PARTA047452	AK035979	0.010244649	2.57117	up
ASMM9PARTA047452	AK035979	0.010244649	2.57117	up
ASMM9PARTA047452	AK035979	0.010244649	2.57117	up
ASMM9PARTA006908		0.02270672	2.914636	up
MM9LINCRNAEXON11245-_P1	mouselincRNA0732	0.003275278	2.5998127	up
ASMM9PARTA002778		0.000395	2.224805	up

MM9LINCRNAEXON10103+_P1	mouselincRNA1524	0.00215482	3.9604363	up
ASMM9PARTA004712		0.012554678	2.7331784	up
ASMM9PARTA046229	4933407L21Rik	0.000337	2.173688	up
ASMM9PARTA014122	Gm12336	0.004241258	2.0746315	up
BU563586_P1	mouselincRNA1020	0.0000528	2.3510246	up
BU563586_P1	mouselincRNA1020	0.0000528	2.3510246	up
BU563586_P1	mouselincRNA1020	0.0000528	2.3510246	up
BU563586_P1	mouselincRNA1020	0.0000528	2.3510246	up
BU563586_P1	mouselincRNA1020	0.0000528	2.3510246	up
BU563586_P1	mouselincRNA1020	0.0000528	2.3510246	up
BU563586_P1	mouselincRNA1020	0.0000528	2.3510246	up
BU563586_P1	mouselincRNA1020	0.0000528	2.3510246	up
BU563586_P1	mouselincRNA1020	0.0000528	2.3510246	up
BU563586_P1	mouselincRNA1020	0.0000528	2.3510246	up
BU563586_P1	mouselincRNA1020	0.0000528	2.3510246	up
BU563586_P1	mouselincRNA1020	0.0000528	2.3510246	up
ASMM9PARTA015625	Gm12531	0.044194296	2.0921938	up
ASMM9PARTA045275	A930015D03Rik	0.00000261	2.331452	up
ASMM9PARTA045275	A930015D03Rik	0.00000261	2.331452	up
ASMM9PARTA015864	4921508A21Rik	0.025709305	2.1973891	up
ASMM9PARTA002329		0.000000369	4.9992266	up
CUST_271_PI426073487	uc.273	0.026784262	2.2414715	up
ASMM9PARTA005505		0.045906883	2.9198754	up
ASMM9PARTA001562		0.010448659	3.90742	up
ASMM9PARTA014788	B930095G15Rik	0.00000127	2.2076585	up
ASMM9PARTA050069	BC038278	0.04067983	2.6454937	up
ASMM9PARTA002260		0.001183438	3.406155	up
ASMM9PARTA002260		0.001183438	3.406155	up
ASMM9PARTA004889		0.00563523	2.3324635	up
ASMM9PARTA017528	Gm13643	0.000000614	7.3649297	up
ASMM9PARTA016469	Gm14097	0.002668656	2.026974	up
MM9LINCRNAEXON11123-_P1	mouselincRNA0738	0.013369232	2.1454418	up
ASMM9PARTA019220	AC109232.1	0.001292565	2.7959533	up
ASMM9PARTA008820		0.00011	5.821497	up
ASMM9PARTA048196	AK040904	0.03488898	3.1997676	up
CF545861_P1	humanlincRNA1302	0.000783	2.3439813	up
CF545861_P1	humanlincRNA1302	0.000783	2.3439813	up
CF545861_P1	humanlincRNA1302	0.000783	2.3439813	up
CF545861_P1	humanlincRNA1302	0.000783	2.3439813	up
CF545861_P1	humanlincRNA1302	0.000783	2.3439813	up
CF545861_P1	humanlincRNA1302	0.000783	2.3439813	up
ASMM9PARTA001340		0.029590495	2.4117913	up
MM9LINCRNAEXON11105+_P1	mouselincRNA0821	0.000815	2.2092288	up
ASMM9PARTA008253		0.0000981	2.2476554	up
ASMM9PARTA008253		0.0000981	2.2476554	up
ASMM9PARTA010666	Gm13890	0.025866123	2.594979	up
ASMM9PARTA014069	A730017L22Rik	0.0000142	3.9973342	up
ASMM9PARTA004567		0.000168	2.0477622	up
ASMM9PARTA011990	Lamr1-ps1	0.00000206	41.54127	up
ASMM9PARTA015722	1700030C14Rik	0.010980662	2.0578198	up
ASMM9PARTA046496	AK039487	0.001401456	0.362343036	down
ASMM9PARTA008317		0.003412898	0.473867218	down
ASMM9PARTA001658		0.001328203	0.480557508	down
ASMM9PARTA045993	Mogat1	0.007517785	0.404617429	down
ASMM9PARTA009402	D230002A01Rik	0.005323117	0.342491804	down
ASMM9PARTA045726	4933436C20Rik	0.000151	0.413698244	down
ASMM9PARTA045034	Gm4759	0.027513668	0.462699567	down

ASMM9PARTA051359	AK015813	0.00000139	0.451870259	down
ASMM9PARTA006958		0.003484102	0.483782568	down
ASMM9PARTA015180	A530058N18Rik	0.000441	0.427147769	down
ASMM9PARTA049723	AK017111	0.000132	0.337344016	down
ASMM9PARTA047120	AK018924	0.030719675	0.416587029	down
ASMM9PARTA047358	AK050516	0.0000168	0.490624124	down
ASMM9PARTA047358	AK050516	0.0000168	0.490624124	down
ASMM9PARTA002716		0.034325533	0.376232944	down
CUST_282_PI426073487	uc.284	0.046482123	0.357398065	down
ASMM9PARTA002902		0.02499771	0.221094688	down
ASMM9PARTA049912	AK041061	0.0000277	0.483591358	down
ASMM9PARTA016047	G630018N14Rik	0.001970962	0.436000643	down
ASMM9PARTA017494	D230004N17Rik	0.033998575	0.472244803	down
ASMM9PARTA006658		0.04800194	0.261555523	down
ASMM9PARTA046988	AK079953	0.044223707	0.41940461	down
ASMM9PARTA047030	BC079904	0.03111449	0.48945295	down
ASMM9PARTA046525	BC072557	0.013454219	0.40896124	down
ASMM9PARTA004452		0.0000215	0.281461039	down
ASMM9PARTA004221		0.0000425	0.317601201	down
ASMM9PARTA004432		0.007780693	0.31441014	down
ASMM9PARTA011381	Mup-ps14	0.003041448	0.445417156	down
ASMM9PARTA001776		0.000233	0.274786785	down
ASMM9PARTA003806		0.001897902	0.478939177	down
ASMM9PARTA010105	Ear-ps2	0.0000234	0.440036569	down
ASMM9PARTA049103	AK042610	0.0000608	0.491740892	down
ASMM9PARTA018383	Gm16551	0.0000231	0.100222665	down
ASMM9PARTA044944	Neat1	0.00036	0.359952129	down
ASMM9PARTA051179	AK162599	0.0000151	0.07108291	down
ASMM9PARTA051030	AK034027	0.000782	0.415819504	down
MM9LINCRNAEXON10396+_P1	mouselincRNA1257	0.013060202	0.4850182	down
ASMM9PARTA048125	AK157947	0.017445752	0.338304092	down
ASMM9PARTA048125	AK157947	0.017445752	0.338304092	down
ASMM9PARTA019355	RP23-141L18.6	0.000253	0.315643215	down
ASMM9PARTA051038	BC065393	0.00266479	0.351991797	down
ASMM9PARTA051038	BC065393	0.00266479	0.351991797	down
ASMM9PARTA051038	BC065393	0.00266479	0.351991797	down
ASMM9PARTA019050	RP24-252L3.2	0.002593198	0.295423785	down
ASMM9PARTA013843	D230017M19Rik	0.0000497	0.386501198	down
ASMM9PARTA049450	AK039238	0.000000391	0.360403921	down
ASMM9PARTA002484		0.00000767	0.494338783	down
ASMM9PARTA048535	AK122507	0.000000285	0.04904395	down
ASMM9PARTA010309	Gm3371	0.003211698	0.496844787	down
ASMM9PARTA004491		0.000167	0.425024363	down
ASMM9PARTA019195	Mup-ps4	0.004270271	0.429479471	down
ASMM9PARTA012200	Gm12873	0.0231295	0.471783346	down
ASMM9PARTA003714		0.00000442	0.115731498	down
ASMM9PARTA018880	RP24-494O2.2	0.000731	0.247678288	down
ASMM9PARTA006132		0.000233	0.269760852	down
ASMM9PARTA006132		0.000233	0.269760852	down
ASMM9PARTA006132		0.000233	0.269760852	down
ASMM9PARTA048083	AK045744	0.013395895	0.298543867	down
ASMM9PARTA014152	Gm15856	0.010562672	0.447107276	down
ASMM9PARTA047388	AK012226	0.000295	0.397015649	down
ASMM9PARTA019265	RP23-48M16.7	0.005870366	0.242084971	down
humanlincRNA0699+_P1	humanlincRNA0699	0.007586535	0.494482172	down
ASMM9PARTA006529		0.0000152	0.267658072	down
ASMM9PARTA006529		0.0000152	0.267658072	down

ASMM9PARTA002624		0.000307	0.497419685	down
ASMM9PARTA006528		0.000798	0.264318531	down
ASMM9PARTA018679	RP23-353F16.2	0.006416766	0.379674793	down
ASMM9PARTA000084		0.021521907	0.383222371	down
ASMM9PARTA002196		0.0000657	0.493223114	down
ASMM9PARTA002534		0.001237931	0.358230175	down
ASMM9PARTA017459	Hoxb3os	0.00000939	0.095841633	down
ASMM9PARTA017459	Hoxb3os	0.00000939	0.095841633	down
ASMM9PARTA017459	Hoxb3os	0.00000939	0.095841633	down
ASMM9PARTA013927	Gm16002	0.000398	0.45618669	down
ASMM9PARTA014954	Gm11525	0.0000318	0.318872447	down
ASMM9PARTA018150	Gm16551	0.000253	0.342415529	down
ASMM9PARTA047402	AK131831	0.001354034	0.335270526	down
BX520759_P1	humanlincRNA2146	0.000921	0.311110774	down
ASMM9PARTA012912	Mup-ps22	0.00000307	0.4316216	down
ASMM9PARTA005312		0.0000167	0.242920826	down
BE952410_P1	mouselincRNA0913	0.010332281	0.397875077	down
ASMM9PARTA050018	AK054420	0.023911573	0.446465405	down
ASMM9PARTA050018	AK054420	0.023911573	0.446465405	down
ASMM9PARTA012332	Mup-ps21	0.000786	0.217376211	down
ASMM9PARTA009152		0.0000294	0.20673309	down
ASMM9PARTA000460		0.011800911	0.333811797	down
ASMM9PARTA006997		0.029949177	0.173930007	down
ASMM9PARTA016113	Gm15477	0.007053185	0.435548397	down
ASMM9PARTA016113	Gm15477	0.007053185	0.435548397	down
ASMM9PARTA012180	Mup-ps17	0.000000522	0.393828813	down
ASMM9PARTA010361	Gm14558	0.001708442	0.367512403	down
ASMM9PARTA019160	RP24-230H12.5	0.0000222	0.27810452	down
MM9LINCRNAEXON10784-_P1		0.000104	0.366977204	down
ASMM9PARTA015424	Gm12688	0.000572	0.1103806	down
ASMM9PARTA016956	Mup-ps4	0.000949	0.45369842	down
CUST_401_PI426073487	uc.403	0.004230779	0.454935914	down
ASMM9PARTA003754		0.0000127	0.124849728	down
ASMM9PARTA007000		0.000000453	0.358056605	down
ASMM9PARTA051795	AK039412	0.02025379	0.480248484	down
ASMM9PARTA003846		0.00313168	0.262216647	down
ASMM9PARTA003595		0.005015307	0.398119839	down
ASMM9PARTA003595		0.005015307	0.398119839	down
MM9LINCRNAEXON10811-_P1	mouselincRNA0961	0.024057826	0.308611495	down
CUST_185_PI426409190		0.0000112	0.313079559	down
CUST_33_PI426409190	AK029792	0.000177	0.434857631	down
ASMM9PARTA049722	LOC433791	7.61E-08	0.352886305	down
ASMM9PARTA049722	LOC433791	7.61E-08	0.352886305	down
ASMM9PARTA049722	LOC433791	7.61E-08	0.352886305	down
ASMM9PARTA049722	LOC433791	7.61E-08	0.352886305	down
ASMM9PARTA049722	LOC433791	7.61E-08	0.352886305	down
ASMM9PARTA049722	LOC433791	7.61E-08	0.352886305	down
ASMM9PARTA049722	LOC433791	7.61E-08	0.352886305	down
MM9LINCRNAEXON11236+_P1	mouselincRNA0720	0.000894	0.412037494	down
ASMM9PARTA003819		0.004296133	0.436223892	down
ASMM9PARTA014404	A530058N18Rik	0.0000332	0.462323116	down
ASMM9PARTA016661	Gm12829	0.001513297	0.44551461	down
ASMM9PARTA045479	Gm13629	0.000121	0.450152539	down
ASMM9PARTA045479	Gm13629	0.000121	0.450152539	down
ASMM9PARTA010983	Gm14356	0.008199115	0.459269901	down
ASMM9PARTA051561		0.001481297	0.200333848	down
ASMM9PARTA016730	Gm12027	0.0000392	0.417104714	down

ASMM9PARTA050890	Lrrk1	0.006577878	0.387302744	down
ASMM9PARTA000231		0.000884	0.391184195	down
AK139568_P1	humanlincRNA0333	0.00000128	0.194979724	down
ASMM9PARTA005120		0.000302	0.265824506	down
CUST_54_PI426409190	AK014857	0.0328176	0.467610973	down
mouselincRNA0158-_P1	mouselincRNA0158	0.034005515	0.450857746	down
ASMM9PARTA009527		0.016835675	0.346499014	down
ASMM9PARTA012440	Mup-ps9	0.0000229	0.429112282	down
ASMM9PARTA017008	Gm15327	0.000214	0.345882923	down
ASMM9PARTA012206	Mup-ps18	0.000000136	0.415858913	down
MM9LINCRNAEXON10524+_P1		0.000027	0.454779997	down
MM9LINCRNAEXON10212-_P1	mouselincRNA1479	0.0000175	0.257998402	down
ASMM9PARTA002713		0.025533654	0.450727912	down
ASMM9PARTA012677	Gm15842	0.002133467	0.359804407	down
ASMM9PARTA006352		0.03992566	0.486270351	down
ASMM9PARTA007875		0.03112585	0.227502282	down
ASMM9PARTA015770	Gm16956	0.002850667	0.405452969	down
ASMM9PARTA051001	AK087237	0.0332025	0.167575007	down
ASMM9PARTA051001	AK087237	0.0332025	0.167575007	down
ASMM9PARTA012988	Gm13508	0.001835251	0.245052795	down
MM9LINCRNAEXON10967+_P1		0.001195018	0.304812867	down
ASMM9PARTA049878	AK054344	0.000193	0.446810834	down
ASMM9PARTA045575	5830416P10Rik	0.002439405	0.276173982	down
ASMM9PARTA049286	1700012H17Rik	0.032262787	0.459180801	down
ASMM9PARTA016460	Scnm1	0.005798496	0.325017141	down
ASMM9PARTA016460	Scnm1	0.005798496	0.325017141	down
ASMM9PARTA016460	Scnm1	0.005798496	0.325017141	down
ASMM9PARTA001071		0.005560467	0.328727046	down
ASMM9PARTA004783		0.0000777	0.384043942	down
ASMM9PARTA002304		0.019730877	0.454541612	down
MM9LINCRNAEXON10385-_P1	mouselincRNA1418	0.015078089	0.352879481	down
ASMM9PARTA050576	AK158295	0.008188009	0.471654952	down
ASMM9PARTA002442		0.018229224	0.447887465	down
ASMM9PARTA014964	Gm15859	0.017069848	0.457206897	down
ASMM9PARTA000578		0.013787686	0.456652407	down
ASMM9PARTA000459		0.00385981	0.427441158	down
ASMM9PARTA050110	Ccdc64	0.03261617	0.401969716	down
ASMM9PARTA017850	Gm12869	0.029190477	0.395660084	down
ASMM9PARTA017850	Gm12869	0.029190477	0.395660084	down
ASMM9PARTA000679		0.016818283	0.309249278	down
humanlincRNA0095-_P1	humanlincRNA0095	0.026606258	0.472603751	down
ASMM9PARTA007172		0.015988328	0.484106939	down
MM9LINCRNAEXON10850+_P1	mouselincRNA1006	0.02514096	0.415607266	down
ASMM9PARTA047980	TCR-V[alpha]new	0.005961638	0.400660079	down
ASMM9PARTA015793	Gm13026	0.00867088	0.382351964	down
ASMM9PARTA047035	AK149240	0.000221	0.150775756	down
ASMM9PARTA005692		0.008178641	0.251093966	down
ASMM9PARTA001994		0.016821543	0.481259444	down
ASMM9PARTA001994		0.016821543	0.481259444	down
ASMM9PARTA017930	7SK.169	0.01591268	0.148920822	down
humanlincRNA1921-_P1	humanlincRNA1921	0.010268884	0.40555801	down
CUST_930_PI426073487	uc.451	0.000914	0.295643891	down
ASMM9PARTA008021		0.029019801	0.41680317	down
ASMM9PARTA013177	Gm14766	0.046619292	0.495165452	down
ASMM9PARTA015024	1700084C06Rik	0.006603594	0.439972718	down
ASMM9PARTA015024	1700084C06Rik	0.006603594	0.439972718	down
ASMM9PARTA015024	1700084C06Rik	0.006603594	0.439972718	down

ASMM9PARTA007013		0.001776751	0.407864941	down
ASMM9PARTA007013		0.001776751	0.407864941	down
ASMM9PARTA005010		0.029155014	0.416336026	down
ASMM9PARTA048499	C21orf63	0.025012154	0.445244103	down
ASMM9PARTA048499	C21orf63	0.025012154	0.445244103	down
ASMM9PARTA045641	A530058N18Rik	0.000032	0.405261132	down
ASMM9PARTA004720		0.003370456	0.274396376	down
ASMM9PARTA017349	Gm15609	0.005875194	0.429054121	down
MM9LINCRNAEXON10980-_P1	mouselincRNA0870	0.016191399	0.255913031	down
ASMM9PARTA049844	AK077204	0.017710544	0.455442158	down
ASMM9PARTA007155		0.014519695	0.360491046	down
ASMM9PARTA046269	TMEFF2	0.013982465	0.454104602	down
ASMM9PARTA047666	AK047145	0.0000729	0.423464794	down
ASMM9PARTA002527		0.000243	0.414173377	down
ASMM9PARTA048438	AK138161	0.00035	0.389151393	down
ASMM9PARTA048438	AK138161	0.00035	0.389151393	down
ASMM9PARTA016983	Gm13748	0.01364272	0.487372115	down
MM9LINCRNAEXON11702+_P1	mouselincRNA0335	0.037530392	0.494178674	down
ASMM9PARTA014593	Gm15169	0.003102606	0.447157558	down
ASMM9PARTA050026	AK018156	0.02313516	0.471849417	down
ASMM9PARTA017010	B630019A10Rik	0.000827	0.279547711	down
ASMM9PARTA017112	Spata5l1	0.005782161	0.494184731	down
ASMM9PARTA008695		0.023356978	0.220157827	down
ASMM9PARTA002592		0.007407138	0.450092991	down
ASMM9PARTA049650	AK078937	0.007783573	0.450551723	down
MM9LINCRNAEXON11302-_P1	mouselincRNA0654	0.0000659	0.360722253	down
ASMM9PARTA014143	4930443B20Rik	0.0000611	0.322571426	down
humanlincRNA1523+_P1	humanlincRNA1523	0.000158	0.468405049	down
ASMM9PARTA017429	Gm7076	0.00885977	0.442879211	down
ASMM9PARTA047473	AK139027	0.014451577	0.473453957	down
ASMM9PARTA008116		0.017804397	0.475114365	down
ASMM9PARTA008116		0.017804397	0.475114365	down
ASMM9PARTA008116		0.017804397	0.475114365	down
ASMM9PARTA008067		0.0000511	0.443449678	down
CUST_643_PI426073487	uc.164	0.003242937	0.254439861	down
ASMM9PARTA015896	BC039966	0.03143818	0.458021421	down
MM9LINCRNAEXON10172+_P1	mouselincRNA1430	0.005813236	0.381628073	down
ASMM9PARTA018457	Gm16556	0.030986499	0.327832339	down
ASMM9PARTA008027		0.011477151	0.446802449	down
ASMM9PARTA008027		0.011477151	0.446802449	down
ASMM9PARTA017404	4930562A09Rik	0.003670974	0.399255787	down
ASMM9PARTA014155	9330111N05Rik	0.004946098	0.384080626	down
ASMM9PARTA011298	Gm11913	0.018509272	0.424561205	down
ASMM9PARTA008473		0.00011	0.35159541	down
ASMM9PARTA001231		0.025542308	0.408158267	down
ASMM9PARTA002911		0.00430235	0.282395844	down
ASMM9PARTA018610	RP23-189E15.2	0.012221053	0.359472664	down
ASMM9PARTA045380	4930412O13Rik	0.000053	0.104949558	down
ASMM9PARTA050493	AK031498	0.008709515	0.201979059	down
ASMM9PARTA017116	1810010K12Rik	0.000724	0.491867705	down
ASMM9PARTA018960	RP24-376M8.1	0.001689828	0.409000799	down
ASMM9PARTA001738		0.00000277	0.033835069	down
ASMM9PARTA004274		0.014627503	0.493914724	down
ASMM9PARTA014937	Gm13838	0.0000299	0.420744735	down
ASMM9PARTA000825		0.000207	0.248911343	down
ASMM9PARTA004842		0.030639958	0.471529408	down
MM9LINCRNAEXON10597-_P1	mouselincRNA1217	0.002173039	0.354654009	down

BC025201_P1	mouselincRNA0389	0.038952243	0.488663066	down
ASMM9PARTA047397	Slc38a6	0.000182	0.25378663	down
ASMM9PARTA047397	Slc38a6	0.000182	0.25378663	down
ASMM9PARTA007736		0.012373405	0.428039498	down
ASMM9PARTA002040		0.001095863	0.473795059	down
ASMM9PARTA047077	Aanat	0.008963102	0.454725753	down
ASMM9PARTA006426		0.031522863	0.278475126	down
ASMM9PARTA045115	4933432I09Rik	0.0000574	0.375129879	down
MM9LINCRNAEXON10017-_P1	mouselincRNA1618	0.001855932	0.355313563	down
ASMM9PARTA016487	1700001G11Rik	0.006681506	0.484329096	down
CUST_432_PI426073487	uc.434	0.007812248	0.452584529	down
ASMM9PARTA046814	AK007947	0.033838507	0.455882745	down
ASMM9PARTA051103	AK076954	0.00029	0.433068036	down
ASMM9PARTA048873	AK163667	0.001728083	0.495378858	down
ASMM9PARTA002365		0.015103385	0.479415435	down
ASMM9PARTA010891	Gm12391	0.00041	0.30400449	down
ASMM9PARTA049459	AK136882	0.000694	0.394886691	down
ASMM9PARTA015587	Gm14453	0.01403609	0.3894544	down
ASMM9PARTA001615		0.00000222	0.131958439	down
ASMM9PARTA007098		0.002253658	0.493181762	down
ASMM9PARTA006877		0.008796744	0.233671503	down
ASMM9PARTA014572	B230112J18Rik	0.005671887	0.381307469	down
ASMM9PARTA001663		0.000387	0.355752424	down
ASMM9PARTA015758	Gm11789	0.0000871	0.359052426	down
ASMM9PARTA047518	BC067033	0.021743435	0.478865739	down
ASMM9PARTA015585	Gm14344	0.014160373	0.43196339	down
ASMM9PARTA008461		0.002052042	0.31660004	down
ASMM9PARTA051102	AK015772	0.0000893	0.189227219	down
ASMM9PARTA009401	Kctd12	0.0000033	0.40786193	down
ASMM9PARTA010758	Gm11306	0.001740878	0.4361002	down
ASMM9PARTA018741	Gm10345	0.003713405	0.335949597	down
ASMM9PARTA047428	1700049E17Rik	0.000425	0.494291428	down
ASMM9PARTA018902	AC160935.1	0.00000848	0.432794539	down
ASMM9PARTA017269	A530058N18Rik	0.000000119	0.38977093	down
MM9LINCRNAEXON12072+_P1	mouselincRNA0095	0.0000236	0.35816692	down
ASMM9PARTA049498	AK005639	0.00031	0.309770438	down
ASMM9PARTA008936		0.002604934	0.433416405	down
ASMM9PARTA017032	Gm16013	0.004035651	0.420528945	down
ASMM9PARTA006929		0.025868986	0.445257227	down
ASMM9PARTA018865	Gm6490	0.0006	0.201649346	down
MM9LINCRNAEXON10148+_P1	mouselincRNA1571	0.002281545	0.312583655	down
ASMM9PARTA008669		0.0000308	0.111388915	down
ASMM9PARTA006629		0.000525	0.470338635	down
ASMM9PARTA006044		0.041650496	0.491457728	down
ASMM9PARTA046930	AK018772	0.00000144	0.117129937	down
ASMM9PARTA003873		0.010305529	0.433508847	down
ASMM9PARTA012580	Gm4746	0.013480953	0.401072661	down
ASMM9PARTA002100		0.000658	0.330507025	down
ASMM9PARTA006636		0.000629	0.32266687	down
ASMM9PARTA019819	RP23-122J17.9	0.0000644	0.478930965	down
MM9LINCRNAEXON10860-_P1	mouselincRNA1009	0.0000842	0.426624	down
MM9LINCRNAEXON10133-_P1		0.0000232	0.096146829	down
ASMM9PARTA007853		0.0000063	0.436279236	down
ASMM9PARTA047504	Tcl1b2	0.047148082	0.38935564	down
ASMM9PARTA002628		0.0257711	0.399191733	down
ASMM9PARTA002628		0.0257711	0.399191733	down
MM9LINCRNAEXON11176-_P1	mouselincRNA0672	0.036750093	0.310307137	down

MM9LINCRNAEXON11362-_P1	mouselincRNA0586	0.035337172	0.452263211	down
ASMM9PARTA002541		0.035495844	0.45319746	down
ASMM9PARTA002541		0.035495844	0.45319746	down
ASMM9PARTA003829		0.000504	0.297347912	down
ASMM9PARTA014590	4930506C21Rik	0.00000765	0.37774105	down
ASMM9PARTA014590	4930506C21Rik	0.00000765	0.37774105	down
ASMM9PARTA007125		0.0000025	0.172173054	down
ASMM9PARTA002413		0.0000462	0.287728732	down
ASMM9PARTA012241	Gm14202	0.003053903	0.298227983	down
MM9LINCRNAEXON10392+_P1	mouselincRNA1254	0.010531249	0.466489494	down
ASMM9PARTA000534		0.000322	0.341782948	down
ASMM9PARTA007187		0.00000764	0.211143834	down
ASMM9PARTA014537	1500016L03Rik	0.0000509	0.093057856	down
ASMM9PARTA017215	1500002F19Rik	0.000206	0.293715645	down
ASMM9PARTA008042		0.04677815	0.391177737	down
BC156060_P1	mouselincRNA1038	0.0000828	0.465559058	down
BC156060_P1	mouselincRNA1038	0.0000828	0.465559058	down
BC156060_P1	mouselincRNA1038	0.0000828	0.465559058	down
BC156060_P1	mouselincRNA1038	0.0000828	0.465559058	down
BC156060_P1	mouselincRNA1038	0.0000828	0.465559058	down
ASMM9PARTA000466		0.009639558	0.185377851	down
MM9LINCRNAEXON10875+_P1		0.0000384	0.405267275	down
ASMM9PARTA001596		0.007536626	0.456133005	down
ASMM9PARTA011383	Gm12550	0.03376331	0.354617097	down
ASMM9PARTA047054	AK018624	0.030310832	0.46913907	down
ASMM9PARTA009795	Gm13775	0.0000119	0.361140945	down
CUST_377_PI426073487	uc.379	0.011175946	0.387693176	down
ASMM9PARTA013594	Gm11692	0.014341485	0.445321708	down
ASMM9PARTA049969	Gnpda2	0.004579535	0.401512691	down
ASMM9PARTA049969	Gnpda2	0.004579535	0.401512691	down
ASMM9PARTA009277		0.034954548	0.384677022	down
ASMM9PARTA016031	Gm15880	0.000544	0.491132794	down
ASMM9PARTA004381		0.013922407	0.463094516	down
MM9LINCRNAEXON10016-_P1	mouselincRNA1618	0.018046526	0.425992295	down
ASMM9PARTA050392	M34473	0.00001	0.218723114	down
ASMM9PARTA002466		0.000258	0.284501327	down
ASMM9PARTA017284	Gm16762	0.001798692	0.406920166	down
ASMM9PARTA002294		0.000389	0.49939633	down
ASMM9PARTA015093	A530058N18Rik	0.0000419	0.419307043	down
ASMM9PARTA004062		0.000533	0.466510821	down
ASMM9PARTA003147		0.002513561	0.260221616	down
ASMM9PARTA051221	Phkb	0.000201	0.490687728	down
ASMM9PARTA051221	Phkb	0.000201	0.490687728	down
MM9LINCRNAEXON10562+_P1	mouselincRNA1192	0.025209295	0.42777074	down
ASMM9PARTA048211	AK080982	0.000168	0.387971397	down
ASMM9PARTA019550	RP23-78F6.7	0.020624833	0.247524998	down
ASMM9PARTA047841	AK086741	0.00000226	0.474249733	down
ASMM9PARTA009136		0.0417169	0.382143504	down
CUST_76_PI426409190	Mup-ps11	0.0000131	0.48233094	down
CUST_76_PI426409190	Mup-ps11	0.0000131	0.48233094	down
CUST_483_PI426073487	uc.2	0.000129	0.406068614	down
CUST_483_PI426073487	uc.2	0.000129	0.406068614	down
ASMM9PARTA017021	Gm13003	0.000758	0.429297678	down
ASMM9PARTA005561		0.000104	0.483016465	down
ASMM9PARTA012454	Gm13230	0.00000676	0.180299545	down
ASMM9PARTA010530	Gm13243	0.002986418	0.455463483	down
ASMM9PARTA015845	A530058N18Rik	0.000253	0.473238415	down

ASMM9PARTA015845	A530058N18Rik	0.000253	0.473238415	down
ASMM9PARTA047453	AK039140	0.000255	0.100872069	down
ASMM9PARTA047453	AK039140	0.000255	0.100872069	down
ASMM9PARTA045267	C230035I16Rik	0.006529616	0.456212496	down
ASMM9PARTA007644		0.01691688	0.327623285	down
ASMM9PARTA007644		0.01691688	0.327623285	down
ASMM9PARTA003135		0.01831891	0.287584555	down
ASMM9PARTA003135		0.01831891	0.287584555	down
CUST_149_PI426073487	uc.151	0.03303717	0.349328833	down
ASMM9PARTA010272	Gm11141	0.009274874	0.480606333	down
ASMM9PARTA011946	Mup-ps10	0.0000163	0.458632495	down
ASMM9PARTA019132	9230115E21Rik	0.0000763	0.211702602	down
ASMM9PARTA019018	5830444B04Rik	0.001817204	0.243884094	down
ASMM9PARTA019018	5830444B04Rik	0.001817204	0.243884094	down
ASMM9PARTA018548	Airn	0.0000116	0.422970871	down
ASMM9PARTA018548	Airn	0.0000116	0.422970871	down
CUST_161_PI426409190		0.001147278	0.341333531	down
ASMM9PARTA047612	AK039634	1.55E-08	0.03905173	down
ASMM9PARTA005211		0.00000251	0.434686316	down
MM9LINCRNAEXON10217-_P1	mouselincRNA1479	0.00046	0.231320039	down
ASMM9PARTA003926		0.00000339	0.025523537	down
ASMM9PARTA019029	RP23-136I9.5	0.004065114	0.266066364	down
ASMM9PARTA018516	Gm16290	0.000578	0.344764578	down
ASMM9PARTA011888	Gm13408	0.00000311	0.282601631	down
ASMM9PARTA008301		0.005426915	0.330173191	down
ASMM9PARTA018654	Ear-ps9	0.0000227	0.306114724	down
ASMM9PARTA048567	wtap	0.00000638	0.463729254	down
ASMM9PARTA019083	RP23-11E12.1	0.001	0.354476812	down
ASMM9PARTA013585	Gm13935	0.0000145	0.162647961	down
ASMM9PARTA051041	Scoc	0.000179	0.099155119	down
ASMM9PARTA051041	Scoc	0.000179	0.099155119	down
mouselincRNA0963+_P1	mouselincRNA0963	0.001133863	0.480745332	down
ASMM9PARTA015038	D230002A01Rik	0.000695	0.166032698	down
ASMM9PARTA010625	Gm15087	0.000302	0.224170498	down
ASMM9PARTA019300	RP24-246L19.3	0.001332479	0.292714906	down
ASMM9PARTA007713		0.001818279	0.378875077	down
ASMM9PARTA047252	AK149260	0.001525681	0.334376119	down
ASMM9PARTA005609		0.012965485	0.426893068	down
ASMM9PARTA007817		0.027461277	0.363885063	down
ASMM9PARTA003086		0.001963345	0.311779934	down
ASMM9PARTA045668	Hhatl	0.031815484	0.387863834	down
AK029714_P1	humanlincRNA0408	0.000000545	0.283854068	down
MM9LINCRNAEXON10711-_P1	mouselincRNA1129	0.010001364	0.443039772	down
mouselincRNA1407+_P1	mouselincRNA1407	0.027479246	0.495960476	down
ASMM9PARTA014686	Gm14199	0.019699335	0.488429019	down
ASMM9PARTA014686	Gm14199	0.019699335	0.488429019	down
ASMM9PARTA016225	1700030C12Rik	0.005584021	0.239739049	down
ASMM9PARTA010260	Mup-ps2	0.00031	0.46470075	down
ASMM9PARTA047302	mKIAA0044	0.00000249	0.389698553	down
ASMM9PARTA009870	Gm5535	0.003075737	0.389670854	down
ASMM9PARTA010967	Gm12392	0.00886978	0.226143434	down
ASMM9PARTA048516	AK020236	0.013940549	0.496808403	down
ASMM9PARTA047208	AK217959	0.000012	0.306205674	down
ASMM9PARTA014504	8430419K02Rik	0.009455259	0.240161369	down
ASMM9PARTA048743	Ppp1r1c	0.003912812	0.286744238	down
ASMM9PARTA048270	AK213404	0.041083943	0.37046827	down
ASMM9PARTA046268	AK020188	0.0000639	0.389736933	down

ASMM9PARTA004186		0.00318922	0.410803137	down
ASMM9PARTA018089	RP23-11E12.5	0.000534	0.261600087	down
ASMM9PARTA050720	Usp29	0.0000665	0.458368246	down
ASMM9PARTA050720	Usp29	0.0000665	0.458368246	down
MM9LINCRNAEXON10276-_P1	mouselincRNA1493	0.027028384	0.341533507	down
ASMM9PARTA019681	RP23-273L20.6	0.0000631	0.267635958	down
MM9LINCRNAEXON10944-_P1	mouselincRNA0866	0.010310014	0.462037925	down
ASMM9PARTA005745		0.0000134	0.337694728	down
ASMM9PARTA009455		0.000000317	0.113642084	down
ASMM9PARTA014791	Gm15787	0.021934036	0.35541094	down
ASMM9PARTA014791	Gm15787	0.021934036	0.35541094	down
ASMM9PARTA004550		0.005766004	0.308531456	down
ASMM9PARTA004550		0.005766004	0.308531456	down
ASMM9PARTA008988		0.000558	0.440445008	down
ASMM9PARTA016760	Gm2694	0.00291606	0.26918967	down
ASMM9PARTA003994		0.001912721	0.377274778	down
ASMM9PARTA047619	Solt	0.011152606	0.329211994	down
ASMM9PARTA047619	Solt	0.011152606	0.329211994	down
ASMM9PARTA047619	Solt	0.011152606	0.329211994	down
ASMM9PARTA019105	RP24-84D8.2	0.000532	0.484175123	down
ASMM9PARTA002348		0.005730165	0.316064676	down
ASMM9PARTA016697	4930412C18Rik	0.000326	0.455663342	down
ASMM9PARTA016030	Gm13601	0.018711317	0.456077315	down
ASMM9PARTA016030	Gm13601	0.018711317	0.456077315	down
humanlincRNA2300+_P1	humanlincRNA2300	0.002569187	0.411118401	down
AV038163_P1	humanlincRNA2267	0.00000584	0.486305633	down
ASMM9PARTA018403	AC159314.2	0.00052	0.495462726	down
ASMM9PARTA019857		0.015543167	0.314756539	down
ASMM9PARTA019605	BC006965	0.0000253	0.079893824	down
ASMM9PARTA019379	RP24-553G19.6	0.00000324	0.309163479	down
ASMM9PARTA019477	AC123998.1	0.004564321	0.37972064	down
ASMM9PARTA050163	BC064108	0.00000416	0.290485485	down
ASMM9PARTA050163	BC064108	0.00000416	0.290485485	down
MM9LINCRNAEXON10539-_P1		0.00000643	0.053826644	down
ASMM9PARTA016109	Gm16682	0.007836073	0.40258341	down
ASMM9PARTA016109	Gm16682	0.007836073	0.40258341	down
ASMM9PARTA018144	Gm15051	0.00000946	0.295011184	down
ASMM9PARTA006626		0.0000136	0.089891567	down
ASMM9PARTA019398	RP24-376M8.4	0.00267173	0.211801809	down
ASMM9PARTA050618	AK007376	0.000739	0.281003621	down
ASMM9PARTA009360	Gm8935	0.0000844	0.310937442	down
ASMM9PARTA003826		0.000013	0.094834255	down
ASMM9PARTA009936	Mup-ps7	0.001257415	0.403149111	down
ASMM9PARTA013773	4930506C21Rik	0.0000767	0.470337905	down
ASMM9PARTA047001	Dlx4	0.010552801	0.448361892	down
ASMM9PARTA012215	Mup-ps19	0.0000242	0.216140088	down
ASMM9PARTA002462		0.019172987	0.395698019	down
ASMM9PARTA049404	Myef2	0.0000145	0.388917243	down
ASMM9PARTA049404	Myef2	0.0000145	0.388917243	down
ASMM9PARTA049404	Myef2	0.0000145	0.388917243	down
ASMM9PARTA049404	Myef2	0.0000145	0.388917243	down
ASMM9PARTA016845	Gm16192	0.004391233	0.365837642	down
ASMM9PARTA016845	Gm16192	0.004391233	0.365837642	down
ASMM9PARTA016845	Gm16192	0.004391233	0.365837642	down
ASMM9PARTA014276	Gm12923	0.044867862	0.413795666	down
ASMM9PARTA004298		0.0000184	0.182439172	down
MM9LINCRNAEXON10635-_P1	mouselincRNA1245	0.008426582	0.467752007	down

ASMM9PARTA017214	Gm15135	0.021248745	0.432028483	down
CK382063_P1	humanlincRNA0287	0.001561529	0.418112919	down
ASMM9PARTA018977	RP23-141L18.5	0.005367646	0.120579975	down
ASMM9PARTA016186	Gm14257	0.042050872	0.377661389	down
ASMM9PARTA045199	Rprl3	0.000555	0.333939801	down
ASMM9PARTA002137		0.0000451	0.318550732	down
ASMM9PARTA050587	AK038880	0.010033796	0.47581533	down
ASMM9PARTA016224	4930412C18Rik	0.000000189	0.283312123	down
ASMM9PARTA018791	CT009711.1	0.012244543	0.306174558	down
ASMM9PARTA001903		0.015719334	0.471004049	down
ASMM9PARTA019278	Gm5226	0.000125	0.487968169	down
BE949468_P1	mouselincRNA1019	0.003282253	0.338326125	down
ASMM9PARTA014333	1500016L03Rik	0.042245153	0.464325781	down
ASMM9PARTA004128		0.000156	0.438094988	down
ASMM9PARTA018327	Gm16577	0.023787472	0.484025536	down
MM9LINCRNAEXON11076+_P1		0.000015	0.478991894	down
ASMM9PARTA051199	4432416J03Rik	0.004704305	0.436495683	down
ASMM9PARTA016971	Gm15535	0.0000122	0.393825447	down
ASMM9PARTA015931	Mup-ps2	0.000296	0.451854721	down
ASMM9PARTA051808		0.001205673	0.175082485	down
ASMM9PARTA050787	AK008222	0.04769696	0.492105399	down
CUST_192_PI426073487	uc.194	0.006887151	0.467806712	down
ASMM9PARTA003853		0.000181	0.489570772	down
CD563094_P1	mouselincRNA0148	0.000361	0.288000566	down
ASMM9PARTA000191		0.000271	0.308673891	down
ASMM9PARTA045335	H2-K2	0.000264	0.385186646	down
ASMM9PARTA016836	Hoxb3os	0.002511187	0.226352245	down
ASMM9PARTA016836	Hoxb3os	0.002511187	0.226352245	down
ASMM9PARTA016836	Hoxb3os	0.002511187	0.226352245	down
ASMM9PARTA050456	A530053G22Rik	0.000118	0.390731383	down
ASMM9PARTA004371		0.005219395	0.328429918	down
ASMM9PARTA017530	Gm16129	0.00563162	0.445302671	down
ASMM9PARTA017530	Gm16129	0.00563162	0.445302671	down
ASMM9PARTA017530	Gm16129	0.00563162	0.445302671	down
CUST_692_PI426073487	uc.213	0.002271777	0.272040867	down
CUST_692_PI426073487	uc.213	0.002271777	0.272040867	down
