# Supplementary material for: Microarray Profile of Long Noncoding RNA and Messenger RNA Expression in a Model of Alzheimer’s Disease
Source: Life (Basel). 2020 May 14;10(5):64. doi: 10.3390/life10050064 (PMC7281340; doi:10.3390/life10050064)
Supplement: Supplementary file 1 [file life-10-00064-s001.zip › life-787240-supplemenatry-to be published - PDF/life-787240-supplementary/Table S2.pdf]

life

Supplementary

Microarray Profile of Long Noncoding RNA and Messenger RNA Expression in a Model of Alzheimer's Disease

Linlin Wang †, Li Zeng †, Hailun Jiang, Zhuorong Li * and Rui Liu *

Institute of Medicinal Biotechnology, Chinese Academy of Medical Sciences and Peking Union Medical College, Beijing 100050, China; wanglinlin@wfmc.edu.cn (L.W.); zengsheng@imb.pumc.edu.cn (L.Z.); jianghailun@imb.pumc.edu.cn (H.J.)

* Correspondence: lizhourong@imb.pumc.edu.cn (Z.L.); +86-10-8352017; .liurui@imb.pumc.edu.cn (R.L.); Tel.: +86-10-67087731

Table S2. Differently expressed lncRNAs in the brain of 3-month-old APP/PS1 mice compared with age-matched WT mice.

Probe Name	Gene Symbol	*p*-Value	Fold Change	Regulation
ASMM9PARTA006853		0.000201	28.02498	up
ASMM9PARTA047480	BC075635	0.035933297	2.031355	up
ASMM9PARTA051288	4933434I20Rik	0.009978537	2.0194137	up
CJ228476_P1	humanlincRNA1040	0.040220477	2.0302415	up
MM9LINCRNAEXON10451+_P1	mouselincRNA1286	0.002579583	2.456823	up
MM9LINCRNAEXON10482-_P1		0.000712	3.868857	up
ASMM9PARTA014628	Gm12940	0.0000735	3.5512953	up
mouselincRNA0294-_P1	mouselincRNA0294	0.018257162	2.6640735	up
humanlincRNA1590+_P1	humanlincRNA1590	0.0000642	4.6550817	up
ASMM9PARTA046759	AK039862	0.000282	10.566481	up
ASMM9PARTA016398	4632427E13Rik	0.003185133	2.1290658	up
ASMM9PARTA016545	Gm16706	0.028662696	2.2541282	up
ASMM9PARTA016545	Gm16706	0.028662696	2.2541282	up
ASMM9PARTA008337		0.008436107	2.0281687	up
ASMM9PARTA005648		0.008224883	2.336319	up
ASMM9PARTA012922	Gm13153	0.000539	2.218174	up
ASMM9PARTA012922	Gm13153	0.000539	2.218174	up
ASMM9PARTA012922	Gm13153	0.000539	2.218174	up
ASMM9PARTA012176	Gm15719	0.02269258	2.4400444	up
mouselincRNA0394-_P1	mouselincRNA0394	0.001435107	3.2710023	up
ASMM9PARTA050284	Gak	0.005066246	2.3687022	up
ASMM9PARTA046970	BC006965	0.00988696	3.1420465	up
ASMM9PARTA045330	Gm11149	0.003619997	2.375882	up
ASMM9PARTA045330	Gm11149	0.003619997	2.375882	up
ASMM9PARTA045330	Gm11149	0.003619997	2.375882	up
CA465684_P1	mouselincRNA0967	0.000124	3.5200474	up
ASMM9PARTA004295		0.000887	3.888033	up
ASMM9PARTA048352	AK013372	0.044562105	2.4789171	up
ASMM9PARTA003398		0.006502184	2.127753	up
ASMM9PARTA018592	Gm16343	0.02400128	2.7728627	up
ASMM9PARTA018592	Gm16343	0.02400128	2.7728627	up

ASMM9PARTA018592	Gm16343	0.02400128	2.7728627	up
ASMM9PARTA018592	Gm16343	0.02400128	2.7728627	up
ASMM9PARTA007995		0.000164	2.0518236	up
ASMM9PARTA013083	Gm12539	0.000000125	2.2940176	up
ASMM9PARTA003991		0.015835704	2.2564034	up
ASMM9PARTA001575		0.026314057	2.788662	up
ASMM9PARTA013061	Gm13913	0.00000275	5.738725	up
ASMM9PARTA051639	Fxy	0.000000031	32.739185	up
ASMM9PARTA051639	Fxy	0.000000031	32.739185	up
ASMM9PARTA004379		0.041796762	2.4507213	up
ASMM9PARTA005351		0.000172	2.3210182	up
MM9LINCRNAEXON11103-_P1	mouselincRNA0821	0.000533	2.4097216	up
ASMM9PARTA049439	mKIAA1125	0.016850177	2.4635985	up
ASMM9PARTA003621		0.019347534	3.077017	up
ASMM9PARTA051477	AK028224	0.000000587	2.7877867	up
ASMM9PARTA048047	Pigz	0.000636	2.181859	up
ASMM9PARTA011710	Gm13689	0.017739022	9.814646	up
ASMM9PARTA050830	AK081140	0.0000141	2.7990656	up
ASMM9PARTA050830	AK081140	0.0000141	2.7990656	up
ASMM9PARTA050830	AK081140	0.0000141	2.7990656	up
ASMM9PARTA047940	AK014435	0.00017	6.3723054	up
ASMM9PARTA005824		0.018023334	3.3533084	up
ASMM9PARTA006453		0.000836	3.3330455	up
ASMM9PARTA003773		0.000316	3.0333865	up
ASMM9PARTA013174	Gm11645	0.001008383	2.66276	up
ASMM9PARTA010558	Xlr5d-ps	0.01289542	4.388562	up
ASMM9PARTA010441	Gm15560	0.000201	2.4514873	up
ASMM9PARTA046107	AK039957	0.0425243	2.4415624	up
ASMM9PARTA002609		0.020581115	2.037375	up
ASMM9PARTA046534	AK005214	0.000213	2.0863976	up
ASMM9PARTA016451	2810008D09Rik	0.000004	2.9708154	up
ASMM9PARTA048537	NR_002853	0.00000167	5.235411	up
BI107540_P1	mouselincRNA1064	0.010709045	2.2569528	up
ASMM9PARTA007417		0.032403223	2.987935	up
ASMM9PARTA047800	AK145614	0.0000712	2.375798	up
ASMM9PARTA013040	Gm13577	0.0000603	2.6024327	up
ASMM9PARTA007811		0.000215	2.0386467	up
ASMM9PARTA003806		0.0000348	2.0197408	up
ASMM9PARTA003377		0.002833969	3.1438978	up
ASMM9PARTA010114	Gm11041	0.001024195	3.0102236	up
DV051927_P1	mouselincRNA0186	0.000917	2.2757103	up
MM9LINCRNAEXON10459+_P1	mouselincRNA1289	0.03823436	2.1349344	up
ASMM9PARTA011188	Hmgb1-ps4	0.0000916	2.662592	up
ASMM9PARTA046284	AK036470	0.001845743	2.6669564	up
ASMM9PARTA019893		0.029229416	2.773302	up
BI180507_P1	mouselincRNA1445	0.02127676	4.2447176	up
ASMM9PARTA013980	2810403D21Rik	0.018716123	2.7028453	up
humanlincRNA0409-_P1	humanlincRNA0409	0.025608456	2.139139	up
ASMM9PARTA015426	Gm14092	0.023995245	2.5788743	up
ASMM9PARTA015426	Gm14092	0.023995245	2.5788743	up
MM9LINCRNAEXON10775+_P1	mouselincRNA1143	0.000746	2.4562912	up
ASMM9PARTA046039	LOC100415784	0.010484707	2.6013057	up
ASMM9PARTA008433		0.03190274	3.5405626	up
ASMM9PARTA008687		0.048377767	2.1047337	up
ASMM9PARTA005626		0.03985472	2.751476	up
ASMM9PARTA013524	Gm16407	0.010861034	2.033524	up
ASMM9PARTA005747		0.000158	2.4484985	up

ASMM9PARTA012161	Gm13239	0.0000698	2.0747144	up
MM9LINCRNAEXON11597-_P1	mouselincRNA0422	0.000128	2.4034324	up
ASMM9PARTA046040	4931406H21Rik	0.001222916	3.587823	up
MM9LINCRNAEXON11375-_P1	mouselincRNA0587	0.0000309	2.5215328	up
ASMM9PARTA006147		0.00110766	3.3976183	up
ASMM9PARTA014539	Gm16095	0.032567773	3.56036	up
MM9LINCRNAEXON10335+_P1	mouselincRNA1390	0.0367432	2.5335274	up
ASMM9PARTA005761		0.0000957	3.036182	up
ASMM9PARTA019063	AC125279.2	0.000576	2.6485398	up
ASMM9PARTA017049	Gm2694	0.00041	6.694751	up
CUST_909_PI426073487	uc.430	0.002456055	2.7067525	up
CUST_909_PI426073487	uc.430	0.002456055	2.7067525	up
CUST_909_PI426073487	uc.430	0.002456055	2.7067525	up
CUST_909_PI426073487	uc.430	0.002456055	2.7067525	up
CUST_909_PI426073487	uc.430	0.002456055	2.7067525	up
ASMM9PARTA014015	Gm13793	0.028507689	2.1264756	up
ASMM9PARTA006865		0.000000351	5.233165	up
ASMM9PARTA013293	Gm14237	0.03541867	2.0105338	up
ASMM9PARTA005238		0.000173	4.28692	up
ASMM9PARTA002704		0.000651	2.0285163	up
ASMM9PARTA011300	Gm9078	0.000000761	2.2853441	up
ASMM9PARTA011532	Gm14627	0.016236996	3.8309026	up
ASMM9PARTA001200		0.017641718	2.142461	up
CUST_580_PI426073487	uc.101	0.000126	2.6631348	up
CUST_580_PI426073487	uc.101	0.000126	2.6631348	up
ASMM9PARTA044891	1700108J01Rik	0.000166	2.864782	up
CUST_453_PI426073487	uc.455	0.004614567	2.5171196	up
ASMM9PARTA046659	4933439F18Rik	0.001098361	2.8961835	up
ASMM9PARTA046659	4933439F18Rik	0.001098361	2.8961835	up
ASMM9PARTA006637		0.04772968	2.5070615	up
ASMM9PARTA006637		0.04772968	2.5070615	up
ASMM9PARTA006637		0.04772968	2.5070615	up
ASMM9PARTA048810	AK042686	0.000011	2.6402147	up
ASMM9PARTA051750	Smpx	0.00371115	5.275266	up
ASMM9PARTA046321	Pde7b	0.0000731	2.1502957	up
ASMM9PARTA014438	Pisd-ps2	0.0000128	4.6784444	up
humanlincRNA0170-_P1	humanlincRNA0170	0.0000126	3.417167	up
ASMM9PARTA004940		0.017105231	10.639719	up
ASMM9PARTA003714		0.0000822	2.0025506	up
ASMM9PARTA006224		0.003340893	2.4044788	up
ASMM9PARTA006230		0.006936705	2.1423283	up
ASMM9PARTA004414		0.043111637	2.557581	up
ASMM9PARTA048835	notch1	0.035429593	2.5399373	up
ASMM9PARTA002107		0.003513236	3.2391667	up
ASMM9PARTA003242		0.0000765	5.0079494	up
ASMM9PARTA006070		0.004084174	3.3641777	up
ASMM9PARTA049104	Caper	0.000143	2.037399	up
ASMM9PARTA000913		0.035069387	2.2505288	up
ASMM9PARTA004587		0.026349595	2.137127	up
ASMM9PARTA014282	Gm15991	0.024864864	2.0553732	up
ASMM9PARTA005146		0.003754153	2.4023252	up
ASMM9PARTA016609	9530027J09Rik	0.046474524	2.3144073	up
ASMM9PARTA046608	Mif1	0.000101	3.7655482	up
ASMM9PARTA014753	Gm12603	0.030143302	3.1725302	up
MM9LINCRNAEXON11571+_P1	mouselincRNA0391	0.001430225	4.6859727	up
ASMM9PARTA003751		0.0000127	2.8992462	up
MM9LINCRNAEXON10848+_P1	mouselincRNA0988	0.00841801	4.0725727	up

humanlincRNA1007-_P1	humanlincRNA1007	0.007820128	2.4125884	up
CUST_124_PI426409190	AC142215.1	0.002284916	2.2577686	up
ASMM9PARTA011180	Gm13379	0.000000448	2.6032276	up
ASMM9PARTA011275	Gm4875	0.011883662	2.4527984	up
ASMM9PARTA008119		0.044492822	2.5162828	up
ASMM9PARTA008119		0.044492822	2.5162828	up
ASMM9PARTA008119		0.044492822	2.5162828	up
MM9LINCRNAEXON11874+_P1	mouselincRNA0150	0.034672875	2.1015608	up
ASMM9PARTA007946		0.0000199	2.4174669	up
ASMM9PARTA008966		0.026900392	2.336617	up
humanlincRNA1751+_P1	humanlincRNA1751	0.000584	4.1130133	up
ASMM9PARTA009943	Gm8062	0.000133	2.2242513	up
CJ270168_P1	mouselincRNA0215	0.00987388	2.1367917	up
ASMM9PARTA047903	AK039376	0.003887294	3.144464	up
ASMM9PARTA008423		0.000599	2.406223	up
ASMM9PARTA006133		0.03928489	3.4627278	up
ASMM9PARTA011789	Gm12856	0.04890321	4.878074	up
ASMM9PARTA005998		0.039257552	2.0160089	up
ASMM9PARTA003225		0.017415788	7.812916	up
ASMM9PARTA013663	Gm13409	0.018440949	2.2564707	up
ASMM9PARTA006952		0.001813855	2.049881	up
ASMM9PARTA002798		0.028890472	2.5611622	up
ASMM9PARTA003298		0.014963471	4.607936	up
ASMM9PARTA004219		0.043227404	2.1320503	up
humanlincRNA2361+_P1	humanlincRNA2361	0.009836117	2.8532429	up
ASMM9PARTA001842		0.004284751	3.8786342	up
ASMM9PARTA008541		0.000181	3.318477	up
ASMM9PARTA010185	Gm12568	0.00000672	2.4526753	up
ASMM9PARTA011470	Gm13106	0.000216	2.631364	up
humanlincRNA1222-_P1	humanlincRNA1222	0.016312262	2.1951003	up
ASMM9PARTA007721		0.0000104	2.4280338	up
ASMM9PARTA018655	Gm10075	0.0000493	2.5575054	up
ASMM9PARTA050637	smarp	0.005516077	2.8053808	up
ASMM9PARTA017459	Hoxb3os	0.00000637	3.7972898	up
ASMM9PARTA017459	Hoxb3os	0.00000637	3.7972898	up
ASMM9PARTA017459	Hoxb3os	0.00000637	3.7972898	up
ASMM9PARTA005665		0.000242	2.0385213	up
ASMM9PARTA000801		0.000398	3.3385348	up
ASMM9PARTA045178	Sycp1-ps1	0.000000566	24.496584	up
ASMM9PARTA050305	AK031300	0.030277932	3.0967844	up
ASMM9PARTA045616	Serpina3h	0.0000276	7.02877	up
ASMM9PARTA007593		0.003643089	2.2934573	up
ASMM9PARTA050227	BC028660	0.02751271	2.640427	up
ASMM9PARTA017822	7SK.135	0.000327	3.7635431	up
MM9LINCRNAEXON12089+_P1	mouselincRNA0102	0.019466547	2.9601	up
MM9LINCRNAEXON12056-_P1	mouselincRNA0073	0.0000599	2.0071633	up
ASMM9PARTA017493	Gm15692	0.000283	2.5770912	up
ASMM9PARTA017493	Gm15692	0.000283	2.5770912	up
ASMM9PARTA007175		0.0000168	2.715501	up
ASMM9PARTA012697	Gm14049	0.0000045	2.4967067	up
MM9LINCRNAEXON11755+_P1		0.016476968	2.1778228	up
BI108441_P1	mouselincRNA0637	0.002379217	2.123651	up
BI108441_P1	mouselincRNA0637	0.002379217	2.123651	up
BI108441_P1	mouselincRNA0637	0.002379217	2.123651	up
BI108441_P1	mouselincRNA0637	0.002379217	2.123651	up
BI108441_P1	mouselincRNA0637	0.002379217	2.123651	up
BI108441_P1	mouselincRNA0637	0.002379217	2.123651	up

BI108441_P1	mouselincRNA0637	0.002379217	2.123651	up
BI108441_P1	mouselincRNA0637	0.002379217	2.123651	up
BI108441_P1	mouselincRNA0637	0.002379217	2.123651	up
ASMM9PARTA014781	Gm16201	0.009322906	6.104802	up
ASMM9PARTA002895		0.000157	2.264759	up
ASMM9PARTA016945	A230056P14Rik	0.0000557	2.1729	up
MM9LINCRNAEXON11978+_P1	mouselincRNA0222	0.003396192	2.076546	up
MM9LINCRNAEXON11855-_P1		0.006603306	2.9693842	up
MM9LINCRNAEXON11855-_P1		0.006603306	2.9693842	up
MM9LINCRNAEXON11855-_P1		0.006603306	2.9693842	up
ASMM9PARTA008934		0.020554073	2.2058582	up
ASMM9PARTA017917	Gm16143	0.000258	5.1132674	up
mouselincRNA0627-_P1	mouselincRNA0627	0.011872051	2.291906	up
ASMM9PARTA012091	Gm12262	0.000499	3.8495035	up
humanlincRNA0574+_P1	humanlincRNA0574	0.003752235	3.301639	up
ASMM9PARTA008094		0.044795886	3.183368	up
ASMM9PARTA046956	AK163075	0.000102	3.097045	up
mouselincRNA1231-_P1	mouselincRNA1231	0.006379338	2.4360187	up
MM9LINCRNAEXON11228-_P1	mouselincRNA0717	0.014125631	2.849154	up
ASMM9PARTA004601		0.003045624	2.6471167	up
ASMM9PARTA009446	Mageb16-ps2	0.016874	2.0087073	up
ASMM9PARTA009446	Mageb16-ps2	0.016874	2.0087073	up
MM9LINCRNAEXON11546-_P1	mouselincRNA0380	0.007522167	2.2332575	up
MM9LINCRNAEXON10673+_P1	mouselincRNA1108	0.036223482	2.458636	up
ASMM9PARTA015200	1700081H22Rik	0.027703231	2.4271917	up
ASMM9PARTA012726	Gm5645	0.043349586	2.5988264	up
ASMM9PARTA007145		0.044273324	2.0477214	up
ASMM9PARTA010111	Gm10977	0.0000293	2.9272864	up
ASMM9PARTA004199		0.03758711	2.1414397	up
ASMM9PARTA047973	Senp7	0.022190634	2.3485007	up
ASMM9PARTA047973	Senp7	0.022190634	2.3485007	up
ASMM9PARTA047973	Senp7	0.022190634	2.3485007	up
ASMM9PARTA047973	Senp7	0.022190634	2.3485007	up
ASMM9PARTA013547	Gm13656	0.012281641	2.0847113	up
ASMM9PARTA015619	Gm16201	0.00000441	2.076095	up
ASMM9PARTA015619	Gm16201	0.00000441	2.076095	up
ASMM9PARTA003655		0.0000218	2.4161873	up
MM9LINCRNAEXON11494-_P1		0.008357966	2.6027863	up
ASMM9PARTA006650		0.03521108	2.344825	up
ASMM9PARTA016400	Gm12204	0.009661051	2.626455	up
ASMM9PARTA006996		0.000166	3.5157826	up
ASMM9PARTA003216		0.0000564	2.098189	up
ASMM9PARTA008655		0.000939	7.163107	up
ASMM9PARTA008655		0.000939	7.163107	up
ASMM9PARTA008655		0.000939	7.163107	up
ASMM9PARTA003440		0.0000109	3.1455529	up
MM9LINCRNAEXON10837+_P1	mouselincRNA0984	0.043724947	3.4329667	up
ASMM9PARTA001481		0.018752499	2.160191	up
ASMM9PARTA012145	Gm11830	0.00054	2.6202261	up
AV570450_P1	humanlincRNA0329	0.0000175	2.737319	up
ASMM9PARTA005435		0.000000103	8.464946	up
ASMM9PARTA048214	AK170928	0.0002	3.4664276	up
ASMM9PARTA048214	AK170928	0.0002	3.4664276	up
ASMM9PARTA048214	AK170928	0.0002	3.4664276	up
ASMM9PARTA000460		0.003875399	2.395071	up
ASMM9PARTA010117	Olfr833-ps1	0.006457352	2.093858	up
ASMM9PARTA013449	Gm15052	0.014026786	2.614133	up

ASMM9PARTA015078	Gm15050	0.044956733	2.340066	up
MM9LINCRNAEXON11234-_P1	mouselincRNA0719	0.013766194	2.6233292	up
BE691366_P1	mouselincRNA0319	0.000189	2.3660378	up
MM9LINCRNAEXON11619+_P1	mouselincRNA0430	0.00008	2.308619	up
ASMM9PARTA011435	Gm11877	0.000515	2.6916611	up
CUST_72_PI426409190	Gm13155	0.00000594	2.5891275	up
ASMM9PARTA019336	AC132234.1	0.000000382	4.485177	up
MM9LINCRNAEXON11460-_P1	mouselincRNA0481	0.000181	2.0892007	up
ASMM9PARTA018622	Gm16110	0.045262147	2.7501729	up
ASMM9PARTA003470		0.000099	3.4856825	up
ASMM9PARTA002195		0.014832666	2.0956483	up
ASMM9PARTA002066		0.028386137	2.4190736	up
ASMM9PARTA001716		0.013537709	2.5782273	up
ASMM9PARTA003484		0.00000945	2.0131752	up
ASMM9PARTA045654	1700069L16Rik	0.018702513	2.1981976	up
ASMM9PARTA003674		0.03600639	2.4271142	up
ASMM9PARTA004306		0.04805554	2.3787801	up
ASMM9PARTA000626		0.01748408	2.102359	up
ASMM9PARTA011989	Gm13170	0.0000835	2.18883	up
ASMM9PARTA011989	Gm13170	0.0000835	2.18883	up
MM9LINCRNAEXON11063+_P1	mouselincRNA0809	0.012436418	3.5161963	up
ASMM9PARTA005235		0.001182655	3.184923	up
ASMM9PARTA005235		0.001182655	3.184923	up
ASMM9PARTA005235		0.001182655	3.184923	up
ASMM9PARTA015476	Hoxa11as	0.0000583	3.0729964	up
ASMM9PARTA012488	Gm11283	0.004371232	4.44906	up
ASMM9PARTA005058		0.000337	2.3116035	up
CJ067790_P1	humanlincRNA2277	0.040010467	2.1346138	up
ASMM9PARTA050768	AK020061	0.03546323	2.800051	up
ASMM9PARTA049866	AK052888	0.010720461	2.1673434	up
mouselincRNA0238-_P1	mouselincRNA0238	0.000153	2.3517025	up
ASMM9PARTA012701	AA413626	0.001630186	2.1547787	up
ASMM9PARTA045090	Defa-ps1	0.023542585	2.3300216	up
ASMM9PARTA002051		0.0000157	7.7211747	up
ASMM9PARTA006154		0.0000728	2.181867	up
ASMM9PARTA019549	9930038B18Rik	0.027896596	2.2347085	up
ASMM9PARTA017474	0610009B14Rik	0.000603	4.0031505	up
ASMM9PARTA000388		0.000199	2.4585037	up
ASMM9PARTA010884	Gm15709	0.004452877	2.0239234	up
ASMM9PARTA005191		0.000000924	5.7952065	up
ASMM9PARTA050583	AK085986	0.005123622	2.3558533	up
AI503337_P1	mouselincRNA0302	0.001020711	4.2963243	up
MM9LINCRNAEXON11740-_P1	mouselincRNA0227	0.03587346	2.8577254	up
BY568487_P1	humanlincRNA0565	0.04374709	2.4912837	up
ASMM9PARTA044984	Dio3os	0.0042765	2.76891	up
ASMM9PARTA018081	Airn	0.01318755	2.7345448	up
ASMM9PARTA016368	Gm15958	0.014977548	3.0862184	up
ASMM9PARTA002292		0.022137187	2.0757802	up
MM9LINCRNAEXON10629+_P1	mouselincRNA1245	0.03437413	2.3566105	up
ASMM9PARTA009332	4930463O16Rik	0.031841278	2.083905	up
ASMM9PARTA001680		0.03233562	2.1490126	up
ASMM9PARTA003081		0.027165085	3.1705647	up
ASMM9PARTA005944		0.002503568	2.0024552	up
MM9LINCRNAEXON10769-_P1	mouselincRNA1141	0.04111953	2.0597034	up
ASMM9PARTA017209	Gm4755	0.000295	2.7423983	up
mouselincRNA0342+_P1	mouselincRNA0342	0.024407785	3.2423823	up
ASMM9PARTA018923	AL732527.1	0.013862874	4.9024773	up

ASMM9PARTA048531	NR_003519	0.0000706	3.1228685	up
ASMM9PARTA005255		0.002170519	2.0907397	up
ASMM9PARTA006989		0.025893586	2.0403616	up
ASMM9PARTA000869		0.01575989	3.2248693	up
ASMM9PARTA007855		0.005455921	3.4755526	up
ASMM9PARTA018758	Gm6413	0.0000551	2.3117208	up
ASMM9PARTA048894	AK085760	0.001428144	2.894351	up
MM9LINCRNAEXON11779-_P1	mouselincRNA0246	0.000000196	2.587728	up
MM9LINCRNAEXON11845+_P1	mouselincRNA0275	0.002915741	3.8656807	up
ASMM9PARTA017125	Gm16952	0.002141229	2.265149	up
ASMM9PARTA017125	Gm16952	0.002141229	2.265149	up
ASMM9PARTA017125	Gm16952	0.002141229	2.265149	up
ASMM9PARTA017125	Gm16952	0.002141229	2.265149	up
ASMM9PARTA019316	Gm11010	0.00000946	2.2463148	up
ASMM9PARTA010409	5730446D14Rik	0.041413274	2.7933743	up
ASMM9PARTA004299		0.000685	5.0032954	up
ASMM9PARTA007731		0.000232	2.3440502	up
ASMM9PARTA001349		0.0000454	2.3884733	up
ASMM9PARTA016541	Zfp133-ps	0.003890402	4.361934	up
ASMM9PARTA006553		0.02260813	2.7823277	up
ASMM9PARTA003726		0.009176646	4.618068	up
ASMM9PARTA012608	Gm13574	0.01647377	2.2820442	up
ASMM9PARTA015212	Gm16548	0.018230565	2.9374247	up
ASMM9PARTA015212	Gm16548	0.018230565	2.9374247	up
ASMM9PARTA017761	Gm5432	0.013280321	2.371175	up
ASMM9PARTA050128	AK019745	0.021588406	2.8310044	up
ASMM9PARTA010177	Gm6973	0.008229041	5.5513196	up
ASMM9PARTA049540	AK076817	0.03513201	2.2847736	up
ASMM9PARTA002531		0.000397	2.3821747	up
humanlincRNA2366+_P1	humanlincRNA2366	0.005269527	2.6376154	up
ASMM9PARTA005275		0.012955572	2.2008467	up
ASMM9PARTA045805	Srsf9	0.0000432	2.59727	up
ASMM9PARTA045163	Asb7	0.03463487	3.2096903	up
ASMM9PARTA045163	Asb7	0.03463487	3.2096903	up
ASMM9PARTA045163	Asb7	0.03463487	3.2096903	up
MM9LINCRNAEXON10684+_P1	mouselincRNA1112	0.0424008	2.006136	up
ASMM9PARTA015391	C030037D09Rik	0.00000036	13.944588	up
ASMM9PARTA015391	C030037D09Rik	0.00000036	13.944588	up
ASMM9PARTA000711		0.01786163	2.4367018	up
ASMM9PARTA048011	Hnrnpa1	0.0000618	2.5927157	up
ASMM9PARTA048011	Hnrnpa1	0.0000618	2.5927157	up
ASMM9PARTA048011	Hnrnpa1	0.0000618	2.5927157	up
ASMM9PARTA046655	AK051928	0.016259091	2.2046125	up
ASMM9PARTA010353	Gm14886	0.034508757	3.5951035	up
humanlincRNA1841+_P1	humanlincRNA1841	0.002860075	4.3917775	up
ASMM9PARTA009005		0.047907405	2.333349	up
MM9LINCRNAEXON10120-_P1	mouselincRNA1557	0.0226214	2.0646715	up
ASMM9PARTA005318		0.016437741	2.132883	up
ASMM9PARTA018864	AC132684.1	0.009493374	2.9903214	up
ASMM9PARTA018864	AC132684.1	0.009493374	2.9903214	up
ASMM9PARTA010524	Gm15161	0.049292143	5.663095	up
ASMM9PARTA017541	A230087F16Rik	0.001168542	2.465981	up
ASMM9PARTA011284	Gm11784	0.02605261	2.0255508	up
ASMM9PARTA004317		0.000579	3.0922732	up
ASMM9PARTA004317		0.000579	3.0922732	up
ASMM9PARTA004317		0.000579	3.0922732	up
ASMM9PARTA051308	AK038653	0.001050724	3.424361	up

ASMM9PARTA000349		0.04724779	2.1572495	up
ASMM9PARTA000349		0.04724779	2.1572495	up
ASMM9PARTA015795	3110053B16Rik	0.001300726	6.3197002	up
ASMM9PARTA002722		0.0000174	2.1170118	up
ASMM9PARTA009632	5830416P10Rik	0.000911	6.434741	up
ASMM9PARTA048852	AK085172	0.0000131	2.1616309	up
ASMM9PARTA051637	Fxy	0.014516354	3.745992	up
ASMM9PARTA051637	Fxy	0.014516354	3.745992	up
AA265833_P1	humanlincRNA1036	0.000095	2.1338434	up
humanlincRNA1995+_P1	humanlincRNA1995	0.0161522	2.6371143	up
ASMM9PARTA019226	Gm16759	0.001865016	4.036291	up
ASMM9PARTA004423		0.02430542	3.0734124	up
ASMM9PARTA003841		0.00727315	2.2232213	up
ASMM9PARTA047266	Rbm25	0.0000809	2.264259	up
ASMM9PARTA008150		0.028322306	2.390619	up
ASMM9PARTA002439		0.029382218	3.0561523	up
ASMM9PARTA002439		0.029382218	3.0561523	up
ASMM9PARTA002439		0.029382218	3.0561523	up
ASMM9PARTA002439		0.029382218	3.0561523	up
ASMM9PARTA002439		0.029382218	3.0561523	up
ASMM9PARTA002439		0.029382218	3.0561523	up
ASMM9PARTA001848		0.015368355	2.1348777	up
ASMM9PARTA011938	Hmgb1-ps1	0.000000231	3.0026157	up
ASMM9PARTA049238	AK029171	0.032021042	2.8493066	up
ASMM9PARTA011190	Olfr388-ps1	0.016305704	2.264089	up
AA791803_P1	mouselincRNA0980	0.0000302	2.2852154	up
ASMM9PARTA003122		0.00000655	6.451795	up
MM9LINCRNAEXON11355-_P1	mouselincRNA0580	0.0000473	2.1077094	up
ASMM9PARTA013353	Gm15079	0.000185	2.861106	up
ASMM9PARTA018405	D030054H15Rik	0.015063091	2.4134104	up
ASMM9PARTA018405	D030054H15Rik	0.015063091	2.4134104	up
ASMM9PARTA019391	AL928605.2	0.008974592	3.0529222	up
ASMM9PARTA019391	AL928605.2	0.008974592	3.0529222	up
ASMM9PARTA019391	AL928605.2	0.008974592	3.0529222	up
ASMM9PARTA019391	AL928605.2	0.008974592	3.0529222	up
ASMM9PARTA019391	AL928605.2	0.008974592	3.0529222	up
humanlincRNA0573-_P1	humanlincRNA0573	0.001393203	2.7621884	up
ASMM9PARTA005817		0.004817067	5.037504	up
ASMM9PARTA005817		0.004817067	5.037504	up
ASMM9PARTA005817		0.004817067	5.037504	up
ASMM9PARTA005817		0.004817067	5.037504	up
ASMM9PARTA005817		0.004817067	5.037504	up
ASMM9PARTA004849		0.00000247	2.4513738	up
CK377992_P1	humanlincRNA1344	0.014633579	2.450874	up
ASMM9PARTA044906	2410002O22Rik	0.00000133	3.0086505	up
ASMM9PARTA044906	2410002O22Rik	0.00000133	3.0086505	up
ASMM9PARTA044906	2410002O22Rik	0.00000133	3.0086505	up
ASMM9PARTA044906	2410002O22Rik	0.00000133	3.0086505	up
MM9LINCRNAEXON10048-_P1		0.004848478	2.6849837	up
ASMM9PARTA011011	Gm13550	0.02613266	2.057723	up
ASMM9PARTA004214		0.00000135	3.7932343	up
ASMM9PARTA016720	B930086A06Rik	0.01631534	3.2284963	up
CUST_365_PI426073487	uc.367	0.00000279	2.331606	up
ASMM9PARTA014985	1500016L03Rik	0.000386	2.0803518	up
ASMM9PARTA047505	AK045952	0.04970699	2.0488245	up
ASMM9PARTA045585	Gm839	0.00036	3.0297437	up
ASMM9PARTA001255		0.000828	2.4208212	up

MM9LINCRNAEXON10124+_P1	mouselincRNA1558	0.002081217	2.2109227	up
ASMM9PARTA050616	AK049852	0.000886	2.2020626	up
ASMM9PARTA009548	Gm8556	0.000038	2.8215811	up
ASMM9PARTA003936		0.00000431	2.1985137	up
ASMM9PARTA005122		0.0000296	2.90204	up
ASMM9PARTA016359	Gm7546	0.00029	2.8354077	up
ASMM9PARTA051381	AK019286	0.0000536	2.0747974	up
ASMM9PARTA018523	Airn	0.000000972	32.12065	up
ASMM9PARTA005165		0.019971391	3.3899665	up
ASMM9PARTA006585		0.011876562	5.0438857	up
ASMM9PARTA010191	Gm10561	0.003211423	2.4564886	up
MM9LINCRNAEXON10257-_P1	mouselincRNA1492	0.007304789	2.0143862	up
ASMM9PARTA015549	A830036E02Rik	0.005435409	6.48569	up
MM9LINCRNAEXON10299-_P1	mouselincRNA1501	0.0000139	5.202261	up
ASMM9PARTA049471	Crls1	0.0000102	2.0639067	up
ASMM9PARTA049471	Crls1	0.0000102	2.0639067	up
ASMM9PARTA045633	4930511M06Rik	0.012056232	3.579406	up
MM9LINCRNAEXON11262-_P1	mouselincRNA0611	0.000201	2.9127474	up
ASMM9PARTA008566		0.008138077	2.888241	up
ASMM9PARTA008566		0.008138077	2.888241	up
MM9LINCRNAEXON10998-_P1	mouselincRNA0882	0.023818165	2.9317777	up
ASMM9PARTA051664	Trip4	0.025422448	2.3340158	up
ASMM9PARTA051664	Trip4	0.025422448	2.3340158	up
ASMM9PARTA051664	Trip4	0.025422448	2.3340158	up
ASMM9PARTA013480	Gm13024	5.77E-08	2.608804	up
ASMM9PARTA050025	nssr 1	0.000115	2.3342924	up
ASMM9PARTA050025	nssr 1	0.000115	2.3342924	up
ASMM9PARTA016994	Gm14636	0.001218206	2.3110693	up
ASMM9PARTA012429	Gm12587	0.00000616	2.4938812	up
ASMM9PARTA003654		0.00000687	2.0081527	up
ASMM9PARTA006085		0.001200238	2.1680171	up
ASMM9PARTA006085		0.001200238	2.1680171	up
ASMM9PARTA051195	AK077358	0.012521365	2.392778	up
MM9LINCRNAEXON11518+_P1	mouselincRNA0380	0.0197234	2.1626685	up
ASMM9PARTA004654		0.003526608	2.8325448	up
humanlincRNA1385-_P1	humanlincRNA1385	0.000239	3.0829844	up
ASMM9PARTA010528	Gm12904	0.026047934	2.242843	up
ASMM9PARTA019357	AC115807.1	0.00000888	2.2221916	up
ASMM9PARTA049188	AK080416	0.0000268	2.868227	up
ASMM9PARTA006829		0.000205	3.305291	up
ASMM9PARTA012197	Gm12331	0.0000293	2.440733	up
ASMM9PARTA017357	Gm14241	0.023490833	7.235909	up
ASMM9PARTA009653	Airn	0.00000408	2.464345	up
ASMM9PARTA004251		0.00000545	2.9137685	up
ASMM9PARTA018993	RP23-122J17.10	0.039848574	2.3952775	up
ASMM9PARTA006719		0.0000392	2.3721611	up
ASMM9PARTA016340	BC006965	0.009729628	5.2280593	up
ASMM9PARTA046645	IL-TIFb	0.00000293	19.12357	up
ASMM9PARTA011900	Gm13902	0.0000068	2.1565235	up
ASMM9PARTA009944	Gm10075	0.0000666	2.6336267	up
ASMM9PARTA005417		0.000336	2.7443876	up
ASMM9PARTA049247	AK040741	0.011870915	3.3874133	up
MM9LINCRNAEXON10743-_P1	mouselincRNA1136	0.003704932	4.004229	up
ASMM9PARTA018302	Gm4475	0.000178	2.1480916	up
AI550063_P1	mouselincRNA0690	0.041834004	4.489893	up
ASMM9PARTA051448	mKIAA0797	0.0000108	2.144439	up
MM9LINCRNAEXON11089-_P1	mouselincRNA0819	0.007006874	2.4465892	up

ASMM9PARTA011099	Gm14080	0.000202	2.2792876	up
ASMM9PARTA002853		0.00000754	2.4123323	up
ASMM9PARTA002853		0.00000754	2.4123323	up
ASMM9PARTA002853		0.00000754	2.4123323	up
ASMM9PARTA002573		0.00000187	3.255407	up
ASMM9PARTA008360		0.011631306	2.0885925	up
ASMM9PARTA050920	AK134755	0.026181862	2.5197067	up
ASMM9PARTA050920	AK134755	0.026181862	2.5197067	up
ASMM9PARTA050920	AK134755	0.026181862	2.5197067	up
ASMM9PARTA050920	AK134755	0.026181862	2.5197067	up
ASMM9PARTA050920	AK134755	0.026181862	2.5197067	up
ASMM9PARTA050920	AK134755	0.026181862	2.5197067	up
ASMM9PARTA050920	AK134755	0.026181862	2.5197067	up
ASMM9PARTA050920	AK134755	0.026181862	2.5197067	up
ASMM9PARTA017328	Gm14396	0.00047	4.0243692	up
MM9LINCRNAEXON10474+_P1	mouselincRNA1297	0.01472569	3.2404318	up
ASMM9PARTA048564	AK009785	0.03556601	3.09798	up
ASMM9PARTA050840	AK050360	0.000294	2.796767	up
ASMM9PARTA010734	Gm13681	0.0000179	2.0720553	up
ASMM9PARTA018651	Gm15900	0.000282	2.4469993	up
ASMM9PARTA004130		0.034731563	2.9782627	up
humanlincRNA1395+_P1	humanlincRNA1395	0.001504081	2.0674143	up
ASMM9PARTA004792		0.008924713	2.8415487	up
ASMM9PARTA015228	Gm12576	0.020730088	2.4878552	up
ASMM9PARTA003730		0.009118591	2.017128	up
ASMM9PARTA012081	Gm14842	0.03005221	3.0849192	up
ASMM9PARTA012560	Gm8152	0.0000259	2.2772627	up
MM9LINCRNAEXON11832+_P1		0.000003	4.128426	up
ASMM9PARTA005321		0.001206461	2.0656667	up
MM9LINCRNAEXON10606+_P1	mouselincRNA1220	0.004105524	2.7398615	up
ASMM9PARTA050390	IAN5	0.04108326	2.0568163	up
ASMM9PARTA012956	Gm11246	0.0000107	2.833935	up
ASMM9PARTA018153	Gm3555	0.002138133	3.5924873	up
ASMM9PARTA018153	Gm3555	0.002138133	3.5924873	up
ASMM9PARTA048105	AK154225	0.048674565	2.1999137	up
ASMM9PARTA045481	Acin1	0.001137984	2.930942	up
ASMM9PARTA045481	Acin1	0.001137984	2.930942	up
ASMM9PARTA045481	Acin1	0.001137984	2.930942	up
ASMM9PARTA045481	Acin1	0.001137984	2.930942	up
ASMM9PARTA000875		0.01336103	3.4429834	up
ASMM9PARTA045894	Gm2694	0.00000456	2.367614	up
ASMM9PARTA002182		0.0000739	2.066629	up
ASMM9PARTA011795	Gm5939	0.030428339	2.9933884	up
ASMM9PARTA050410	Sart3	0.032490447	2.2755787	up
ASMM9PARTA002287		0.034672294	2.5321636	up
ASMM9PARTA003909		0.006051574	2.93003	up
ASMM9PARTA014215	Gm15326	0.000372	2.1516373	up
ASMM9PARTA014677	A830036E02Rik	0.000295	66.14013	up
ASMM9PARTA006503		0.013109443	2.5266209	up
ASMM9PARTA006503		0.013109443	2.5266209	up
ASMM9PARTA046346	AK009784	0.03122611	3.678357	up
ASMM9PARTA003355		0.012020879	2.1215281	up
ASMM9PARTA048576	AK139806	0.000554	2.706758	up
ASMM9PARTA049336	Phc3	0.041802477	2.2484055	up
ASMM9PARTA049336	Phc3	0.041802477	2.2484055	up
ASMM9PARTA049336	Phc3	0.041802477	2.2484055	up
ASMM9PARTA049336	Phc3	0.041802477	2.2484055	up

ASMM9PARTA017655	Gm14033	0.0000308	3.381938	up
mouselincRNA1619+_P1	mouselincRNA1619	0.035568483	2.4425995	up
ASMM9PARTA010820	Gm13682	0.0351184	2.0219026	up
ASMM9PARTA048287	AK132606	0.00000451	2.2182393	up
MM9LINCRNAEXON11366-_P1	mouselincRNA0586	0.014363263	2.676287	up
MM9LINCRNAEXON10522+_P1	mouselincRNA1351	0.005230516	2.0842113	up
ASMM9PARTA050649	AK006397	0.004241719	2.9088519	up
MM9LINCRNAEXON11828+_P1	mouselincRNA0271	0.009686475	2.0017784	up
ASMM9PARTA046930	AK018772	0.000000342	3.8772461	up
ASMM9PARTA007292		0.002808958	2.4767034	up
ASMM9PARTA007292		0.002808958	2.4767034	up
ASMM9PARTA007292		0.002808958	2.4767034	up
ASMM9PARTA048920	AK078367	0.0000252	2.0531669	up
ASMM9PARTA048920	AK078367	0.0000252	2.0531669	up
ASMM9PARTA048920	AK078367	0.0000252	2.0531669	up
ASMM9PARTA012893	Hmgb1-ps5	0.000115	2.3804212	up
ASMM9PARTA047260	AK039495	0.005271091	2.366137	up
ASMM9PARTA047260	AK039495	0.005271091	2.366137	up
ASMM9PARTA047260	AK039495	0.005271091	2.366137	up
ASMM9PARTA010688	Gm15059	0.0000167	2.8139498	up
ASMM9PARTA046016	Olfr856-ps1	0.000678	2.6691747	up
ASMM9PARTA002453		0.017013626	2.6575408	up
ASMM9PARTA018017	Gm16169	0.004413288	4.2493205	up
ASMM9PARTA009000		0.000655	2.386869	up
ASMM9PARTA045101	C030034L19Rik	0.027725356	2.2098374	up
ASMM9PARTA046691	AK158612	0.000578	13.532002	up
MM9LINCRNAEXON10071+_P1	mouselincRNA1506	0.000516	2.4702082	up
ASMM9PARTA001943		0.0000458	2.0189033	up
mouselincRNA0078-_P1	mouselincRNA0078	0.042731665	2.1083522	up
ASMM9PARTA005470		0.009119179	2.27836	up
ASMM9PARTA005470		0.009119179	2.27836	up
DV645173_P1	humanlincRNA0146	0.000425	2.3809586	up
ASMM9PARTA013817	Gm15247	0.0000484	2.220241	up
ASMM9PARTA013817	Gm15247	0.0000484	2.220241	up
ASMM9PARTA003161		0.000378	2.779386	up
ASMM9PARTA014167	Gm13944	0.00000637	24.713064	up
ASMM9PARTA014167	Gm13944	0.00000637	24.713064	up
ASMM9PARTA014167	Gm13944	0.00000637	24.713064	up
ASMM9PARTA047833	AK142028	0.0000286	2.3726356	up
ASMM9PARTA047833	AK142028	0.0000286	2.3726356	up
ASMM9PARTA016977	Gm16801	0.002010423	2.1518593	up
ASMM9PARTA016977	Gm16801	0.002010423	2.1518593	up
ASMM9PARTA016977	Gm16801	0.002010423	2.1518593	up
ASMM9PARTA016977	Gm16801	0.002010423	2.1518593	up
MM9LINCRNAEXON12014+_P1	mouselincRNA0024	0.04592268	2.5345564	up
ASMM9PARTA002758		0.0000283	2.4080474	up
ASMM9PARTA049905	AK131821	0.002145707	2.0053005	up
ASMM9PARTA048635	2810051F02Rik	0.000153	2.5020425	up
ASMM9PARTA006390		0.006344566	2.147089	up
CUST_150_PI426073487	uc.152	0.001006108	2.1621218	up
ASMM9PARTA049815	AK042933	0.0000436	3.694951	up
ASMM9PARTA011317	Hmgb1-ps3	0.0000112	2.4633458	up
ASMM9PARTA051121	Lrrc49	0.00000406	30.136993	up
ASMM9PARTA051121	Lrrc49	0.00000406	30.136993	up
ASMM9PARTA051121	Lrrc49	0.00000406	30.136993	up
ASMM9PARTA049391	Ptpn22	0.037405808	3.3586593	up
ASMM9PARTA000678		0.000705	2.010737	up

ASMM9PARTA002375		0.00000236	10.672522	up
ASMM9PARTA018971	AC165246.1	0.000978	3.2285278	up
ASMM9PARTA016392	Gm13944	0.00056	2.0245235	up
ASMM9PARTA016392	Gm13944	0.00056	2.0245235	up
ASMM9PARTA016392	Gm13944	0.00056	2.0245235	up
ASMM9PARTA049191	A230067G21Rik	0.0000169	3.3046813	up
CUST_62_PI426073487	uc.64	0.0000197	2.16086	up
ASMM9PARTA005370		0.028455436	2.2531095	up
ASMM9PARTA048621	Akd2	0.01106173	2.9409096	up
ASMM9PARTA006158		0.001226889	2.3245618	up
CUST_280_PI426073487	uc.282	0.000848	2.0808525	up
CUST_280_PI426073487	uc.282	0.000848	2.0808525	up
CUST_280_PI426073487	uc.282	0.000848	2.0808525	up
CUST_375_PI426073487	uc.377	0.009526183	5.7803087	up
ASMM9PARTA050230	AK131834	0.001892743	6.9195056	up
ASMM9PARTA007495		0.00000988	2.4579487	up
ASMM9PARTA007256		0.00063	2.2241955	up
ASMM9PARTA003879		0.0000433	2.259249	up
ASMM9PARTA007283		0.000184	7.688201	up
MM9LINCRNAEXON12034-_P1	mouselincRNA0038	0.002067127	2.0816345	up
ASMM9PARTA003916		0.000000772	3.034404	up
MM9LINCRNAEXON10204-_P1	mouselincRNA1471	0.00026	2.1629808	up
MM9LINCRNAEXON10413-_P1	mouselincRNA1260	0.00340574	2.0790362	up
ASMM9PARTA044959	E230029C05Rik	0.000134	2.215237	up
ASMM9PARTA005240		0.000645	2.5264568	up
ASMM9PARTA048669	AK165766	0.0000759	2.680739	up
ASMM9PARTA048669	AK165766	0.0000759	2.680739	up
ASMM9PARTA048669	AK165766	0.0000759	2.680739	up
ASMM9PARTA047938	AK082117	0.00000995	2.7594564	up
ASMM9PARTA019295	RP23-133I8.1	0.0000967	2.4956837	up
ASMM9PARTA047783	Ugcgl2	0.0000784	3.3411317	up
ASMM9PARTA003032		0.024053346	2.558817	up
ASMM9PARTA003032		0.024053346	2.558817	up
ASMM9PARTA003032		0.024053346	2.558817	up
ASMM9PARTA012510	Gm13121	0.000000653	2.3313408	up
ASMM9PARTA044960	BC006965	0.0000415	24.99514	up
ASMM9PARTA010161	Gm9387	0.000167	2.3674896	up
ASMM9PARTA012055	Gm12118	0.0000623	2.2615724	up
ASMM9PARTA011936	Gm13320	0.000000125	2.7886217	up
ASMM9PARTA000775		0.015329428	2.0237582	up
ASMM9PARTA013691	Gm14162	0.000122	2.7063563	up
ASMM9PARTA018548	Airn	0.000185	2.4098134	up
ASMM9PARTA018548	Airn	0.000185	2.4098134	up
ASMM9PARTA014091	4930419G24Rik	0.03582335	3.5791323	up
ASMM9PARTA014091	4930419G24Rik	0.03582335	3.5791323	up
ASMM9PARTA010447	Gm6088	0.002167696	2.0168116	up
ASMM9PARTA046327	AK009351	2.97E-08	3.6528134	up
ASMM9PARTA010097	Gm10361	0.0000913	2.6537814	up
ASMM9PARTA000537		0.000127	2.5941472	up
ASMM9PARTA012255	Gm11668	0.038839106	2.4875536	up
CUST_626_PI426073487	uc.147	0.002982467	2.655264	up
CUST_626_PI426073487	uc.147	0.002982467	2.655264	up
CUST_626_PI426073487	uc.147	0.002982467	2.655264	up
ASMM9PARTA005002		0.0000951	2.8442383	up
ASMM9PARTA014139	Gm3160	0.0000163	2.5387328	up
ASMM9PARTA014139	Gm3160	0.0000163	2.5387328	up
ASMM9PARTA007789		0.00000244	2.0733392	up

ASMM9PARTA001661		0.00305647	4.7534437	up
ASMM9PARTA005550		0.000101	2.3074667	up
humanlincRNA2125-_P1	humanlincRNA2125	0.001283047	2.162889	up
ASMM9PARTA002121		0.001094694	2.2519882	up
ASMM9PARTA015409	Gm10565	0.009211536	2.8152647	up
ASMM9PARTA013565	Gm14473	0.00000376	2.1654344	up
ASMM9PARTA003572		0.000015	2.6028886	up
ASMM9PARTA046351	Ppil4	0.000693	4.7250004	up
ASMM9PARTA050623	Kox-1	0.043408602	2.1488585	up
ASMM9PARTA018884	AC126606.1	0.0000685	2.1529355	up
ASMM9PARTA018884	AC126606.1	0.0000685	2.1529355	up
ASMM9PARTA000684		0.00000768	4.0846176	up
MM9LINCRNAEXON10518+_P1	mouselincRNA1347	0.000000229	6.925032	up
ASMM9PARTA013453	Gm16471	0.0000142	2.5961287	up
ASMM9PARTA015970	A730036I17Rik	0.007606336	2.025186	up
ASMM9PARTA012955	Gm13932	0.0000572	2.3989727	up
ASMM9PARTA045371	5033406O09Rik	0.033640627	3.1963263	up
ASMM9PARTA002899		0.00000316	2.6463146	up
ASMM9PARTA002899		0.00000316	2.6463146	up
ASMM9PARTA002899		0.00000316	2.6463146	up
ASMM9PARTA049183	AK036371	0.004450032	2.2127364	up
ASMM9PARTA002490		0.00328782	2.010554	up
ASMM9PARTA001846		0.000444	2.4273815	up
ASMM9PARTA003213		0.0000529	2.3987834	up
CUST_846_PI426073487	uc.367	0.012606558	2.2397985	up
ASMM9PARTA046971	AK138212	0.025324816	4.1287336	up
ASMM9PARTA001331		0.005287835	3.7277648	up
ASMM9PARTA011302	Gm5762	0.002200684	2.2737384	up
ASMM9PARTA003364		0.046860997	2.690849	up
ASMM9PARTA001787		0.0000628	3.137793	up
MM9LINCRNAEXON11899+_P1	mouselincRNA0173	0.01598255	2.7152762	up
MM9LINCRNAEXON12113-_P1		0.0000157	2.2590175	up
ASMM9PARTA048395	Gng13	0.0000249	2.0286946	up
ASMM9PARTA048395	Gng13	0.0000249	2.0286946	up
MM9LINCRNAEXON11573-_P1	mouselincRNA0396	0.013408884	2.0755594	up
ASMM9PARTA045591	Gm11110	0.018723879	2.361407	up
ASMM9PARTA007871		0.0000386	2.3022263	up
ASMM9PARTA002181		0.0000148	2.5523238	up
ASMM9PARTA010492	Gm13378	0.00016	2.0541239	up
EL605756_P1	humanlincRNA0451	0.000341	3.1301522	up
ASMM9PARTA016760	Gm2694	0.0000766	7.4226823	up
ASMM9PARTA019401	9430021M05Rik	0.00000354	3.767519	up
BC040767_P1	humanlincRNA1200	0.000000439	28.610907	up
ASMM9PARTA049783	Rps6ka1	0.00000531	2.581213	up
ASMM9PARTA011684	Gm12345	0.0000723	2.2303417	up
MM9LINCRNAEXON10767+_P1	mouselincRNA1138	0.0000169	4.1780615	up
MM9LINCRNAEXON10396-_P1	mouselincRNA1257	0.001013944	3.5003083	up
ASMM9PARTA048987	cAMP-GEFII	0.0000561	2.544815	up
ASMM9PARTA048987	cAMP-GEFII	0.0000561	2.544815	up
ASMM9PARTA048987	cAMP-GEFII	0.0000561	2.544815	up
ASMM9PARTA048987	cAMP-GEFII	0.0000561	2.544815	up
ASMM9PARTA006908		0.007612485	4.023833	up
ASMM9PARTA000659		0.0000145	2.0331874	up
MM9LINCRNAEXON11795+_P1	mouselincRNA0252	0.0000168	41.30106	up
MM9LINCRNAEXON10103+_P1	mouselincRNA1524	0.0000332	5.9307623	up
ASMM9PARTA018377	Gm9727	0.00000368	2.1676364	up
MM9LINCRNAEXON10539-_P1		0.000000804	10.322508	up

ASMM9PARTA011271	Gm13228	0.0000257	2.9032066	up
BU563586_P1	mouselincRNA1020	0.0000607	2.3051958	up
BU563586_P1	mouselincRNA1020	0.0000607	2.3051958	up
BU563586_P1	mouselincRNA1020	0.0000607	2.3051958	up
BU563586_P1	mouselincRNA1020	0.0000607	2.3051958	up
BU563586_P1	mouselincRNA1020	0.0000607	2.3051958	up
BU563586_P1	mouselincRNA1020	0.0000607	2.3051958	up
BU563586_P1	mouselincRNA1020	0.0000607	2.3051958	up
BU563586_P1	mouselincRNA1020	0.0000607	2.3051958	up
BU563586_P1	mouselincRNA1020	0.0000607	2.3051958	up
BU563586_P1	mouselincRNA1020	0.0000607	2.3051958	up
BU563586_P1	mouselincRNA1020	0.0000607	2.3051958	up
BU563586_P1	mouselincRNA1020	0.0000607	2.3051958	up
ASMM9PARTA018144	Gm15051	0.000032	2.0457785	up
ASMM9PARTA011414	Gm15778	0.000000297	2.4930751	up
ASMM9PARTA003826		0.0000516	2.724293	up
ASMM9PARTA049172	Csnk2a1	0.023294207	3.2138712	up
ASMM9PARTA001365		0.0000871	2.5701458	up
ASMM9PARTA002329		0.00000023	7.3232145	up
ASMM9PARTA012094	Gm6461	0.000526	2.8002222	up
ASMM9PARTA004765		0.001587989	2.1544375	up
ASMM9PARTA045708	3930402G23Rik	0.000181	2.1272426	up
ASMM9PARTA018060	E130111B04Rik	0.007533206	2.5757177	up
ASMM9PARTA012898	Gm5396	0.00000217	2.5345893	up
ASMM9PARTA001969		0.005341575	2.1894698	up
CUST_174_PI426073487	uc.176	0.008749773	3.340239	up
ASMM9PARTA018095	Gm16990	0.001112052	2.4591026	up
ASMM9PARTA046635	AK046981	0.024679642	3.0707684	up
ASMM9PARTA046635	AK046981	0.024679642	3.0707684	up
ASMM9PARTA046635	AK046981	0.024679642	3.0707684	up
ASMM9PARTA046635	AK046981	0.024679642	3.0707684	up
ASMM9PARTA046635	AK046981	0.024679642	3.0707684	up
ASMM9PARTA046635	AK046981	0.024679642	3.0707684	up
ASMM9PARTA046635	AK046981	0.024679642	3.0707684	up
ASMM9PARTA046635	AK046981	0.024679642	3.0707684	up
ASMM9PARTA046635	AK046981	0.024679642	3.0707684	up
CUST_271_PI426073487	uc.273	0.006045092	3.300645	up
ASMM9PARTA002204		0.0000286	2.802411	up
ASMM9PARTA013698	Gm13366	0.005261274	4.8298717	up
ASMM9PARTA051114	Dnchc2	0.000213	2.3465252	up
ASMM9PARTA012120	Gm5937	0.0000265	2.2877882	up
ASMM9PARTA001998		0.031154037	2.6208203	up
ASMM9PARTA014788	B930095G15Rik	0.00000683	5.532669	up
ASMM9PARTA007333		0.010852524	2.2434957	up
ASMM9PARTA050069	BC038278	0.017552208	3.5265713	up
CUST_155_PI426409190		0.009918	2.0826352	up
ASMM9PARTA048236	AK182695	0.018163228	2.247597	up
ASMM9PARTA049027	AK047243	0.0000543	2.1900108	up
ASMM9PARTA002260		0.000839	2.7180097	up
ASMM9PARTA002260		0.000839	2.7180097	up
ASMM9PARTA017175	E130307A14Rik	0.023805067	2.0302386	up
ASMM9PARTA048313	AK153988	0.0000741	2.4665794	up
ASMM9PARTA004889		0.012255047	2.4339495	up
ASMM9PARTA017814	Gm12840	0.00000586	2.3893678	up
ASMM9PARTA045192	Ipw	0.0000109	5.409522	up
ASMM9PARTA017528	Gm13643	0.00000531	8.988608	up
ASMM9PARTA010788	Gm12261	0.00000501	2.414087	up

ASMM9PARTA014333	1500016L03Rik	0.000878	6.8242226	up
ASMM9PARTA011755	Hmgb1-ps6	0.000174	2.4944484	up
ASMM9PARTA003370		0.00339874	2.5733292	up
ASMM9PARTA001844		0.000108	6.169028	up
ASMM9PARTA004751		0.023230705	2.95953	up
CF545861_P1	humanlincRNA1302	0.001655845	2.0089698	up
CF545861_P1	humanlincRNA1302	0.001655845	2.0089698	up
CF545861_P1	humanlincRNA1302	0.001655845	2.0089698	up
CF545861_P1	humanlincRNA1302	0.001655845	2.0089698	up
CF545861_P1	humanlincRNA1302	0.001655845	2.0089698	up
CF545861_P1	humanlincRNA1302	0.001655845	2.0089698	up
ASMM9PARTA046423	AK016061	0.000154	2.4863772	up
MM9LINCRNAEXON10221-_P1	mouselincRNA1483	0.009539064	3.5606596	up
MM9LINCRNAEXON11105+_P1	mouselincRNA0821	0.004060553	3.4164205	up
ASMM9PARTA014299	Gm13375	0.000103	2.5118647	up
ASMM9PARTA051714	AK163556	0.000427	2.1364088	up
ASMM9PARTA012250	Gm15387	0.000014	2.225801	up
ASMM9PARTA003552		0.01268047	2.4038773	up
ASMM9PARTA016836	Hoxb3os	0.000823	2.5090508	up
ASMM9PARTA016836	Hoxb3os	0.000823	2.5090508	up
ASMM9PARTA016836	Hoxb3os	0.000823	2.5090508	up
ASMM9PARTA044894	C330024D21Rik	0.00000351	5.3520403	up
ASMM9PARTA014069	A730017L22Rik	0.0000979	2.6616864	up
MM9LINCRNAEXON11310-_P1	mouselincRNA0658	0.000146	2.061943	up
ASMM9PARTA004567		0.000783	2.2554648	up
ASMM9PARTA011990	Lamr1-ps1	0.0000172	10.635635	up
ASMM9PARTA010121	Gm14162	0.0000521	2.6393824	up
ASMM9PARTA015799	5530601H04Rik	0.00024	2.2407029	up
DV653038_P1	mouselincRNA1246	0.000301	2.5972872	up
DV653038_P1	mouselincRNA1246	0.000301	2.5972872	up
DV653038_P1	mouselincRNA1246	0.000301	2.5972872	up
DV653038_P1	mouselincRNA1246	0.000301	2.5972872	up
DV653038_P1	mouselincRNA1246	0.000301	2.5972872	up
DV653038_P1	mouselincRNA1246	0.000301	2.5972872	up
ASMM9PARTA008317		0.002834839	0.325945061	down
ASMM9PARTA001658		0.000265	0.342372234	down
ASMM9PARTA050096	AK039125	0.04244341	0.412248327	down
BY668977_P1	humanlincRNA0676	0.01711409	0.495437516	down
ASMM9PARTA007767		0.001897486	0.412644075	down
MM9LINCRNAEXON10677-_P1	mouselincRNA1108	0.024073955	0.389109585	down
ASMM9PARTA004651		0.042547293	0.279583788	down
ASMM9PARTA002902		0.040899456	0.295395179	down
AK138338_P1	mouselincRNA1426	0.000292	0.288348306	down
MM9LINCRNAEXON10863-_P1	mouselincRNA1009	0.031525224	0.468723422	down
ASMM9PARTA051451	AK032255	0.007611876	0.339942518	down
ASMM9PARTA016463	2410057H14Rik	0.006866116	0.499634767	down
ASMM9PARTA006658		0.03766076	0.228369545	down
ASMM9PARTA016370	4930448N21Rik	0.002114768	0.425981788	down
ASMM9PARTA047030	BC079904	0.02010789	0.43873808	down
BI738939_P1	mouselincRNA0637	0.014044236	0.456293078	down
ASMM9PARTA007676		0.003625626	0.456742803	down
ASMM9PARTA011381	Mup-ps14	0.000208	0.317072885	down
ASMM9PARTA001776		0.001569815	0.383517786	down
ASMM9PARTA048053	AK086749	0.000964	0.361725297	down
ASMM9PARTA048053	AK086749	0.000964	0.361725297	down
ASMM9PARTA010105	Ear-ps2	0.002450254	0.426082304	down
ASMM9PARTA045860	A630072M18Rik	0.041306496	0.443967268	down

ASMM9PARTA051179	AK162599	0.000106	0.054249905	down
ASMM9PARTA001669		0.00000937	0.225942593	down
ASMM9PARTA017326	C030005K06Rik	0.0000048	0.243144776	down
ASMM9PARTA017326	C030005K06Rik	0.0000048	0.243144776	down
ASMM9PARTA017326	C030005K06Rik	0.0000048	0.243144776	down
ASMM9PARTA014132	4930500J02Rik	0.007932568	0.489576237	down
ASMM9PARTA051038	BC065393	0.0000314	0.37705612	down
ASMM9PARTA051038	BC065393	0.0000314	0.37705612	down
ASMM9PARTA051038	BC065393	0.0000314	0.37705612	down
MM9LINCRNAEXON11848-_P1	mouselincRNA0275	0.002884034	0.487236689	down
ASMM9PARTA014587	1110050K14Rik	0.049582146	0.477183336	down
ASMM9PARTA018348	Gm15717	0.026301028	0.425540607	down
ASMM9PARTA004962		0.043338127	0.464302907	down
ASMM9PARTA004491		0.00000453	0.155603262	down
ASMM9PARTA050182	AB294528	0.000119	0.42473044	down
ASMM9PARTA019195	Mup-ps4	0.000207	0.300166803	down
ASMM9PARTA017897	7SK.148	0.004638983	0.490170489	down
ASMM9PARTA018658	4930524O07Rik	0.016588803	0.416587446	down
ASMM9PARTA018658	4930524O07Rik	0.016588803	0.416587446	down
CUST_844_PI426073487	uc.365	0.002935674	0.400308398	down
CF723729_P1	humanlincRNA0214	0.001162223	0.35999182	down
ASMM9PARTA011084	Gm11212	0.041761033	0.472175991	down
ASMM9PARTA048083	AK045744	0.00000881	0.203487429	down
ASMM9PARTA003556		0.03140472	0.427254715	down
ASMM9PARTA012178	Mup-ps13	0.0000849	0.270685465	down
ASMM9PARTA005430		0.000481	0.297645455	down
ASMM9PARTA006529		0.00264838	0.359784446	down
ASMM9PARTA006529		0.00264838	0.359784446	down
CUST_685_PI426073487	uc.206	0.000929	0.432024303	down
ASMM9PARTA007358		0.00858018	0.381737043	down
ASMM9PARTA018679	RP23-353F16.2	0.012067884	0.408693015	down
ASMM9PARTA006695		0.0000413	0.404202199	down
ASMM9PARTA002534		0.00048	0.327512047	down
ASMM9PARTA050887	AK135845	0.003916789	0.409443738	down
ASMM9PARTA013927	Gm16002	0.0000318	0.32125911	down
ASMM9PARTA047402	AK131831	0.001281894	0.37229095	down
BX520759_P1	humanlincRNA2146	0.001458974	0.353990245	down
ASMM9PARTA012912	Mup-ps22	0.00000183	0.360630905	down
ASMM9PARTA011898	Mup-ps16	0.0000106	0.41563741	down
ASMM9PARTA012332	Mup-ps21	0.002465222	0.149434592	down
AW060438_P1	humanlincRNA0797	0.035770245	0.40650694	down
ASMM9PARTA012212	Gm14900	0.000599	0.279539123	down
ASMM9PARTA006997		0.03711573	0.189660481	down
ASMM9PARTA014368	2410018L13Rik	0.001096903	0.471016295	down
ASMM9PARTA012180	Mup-ps17	0.00000043	0.191345079	down
ASMM9PARTA016956	Mup-ps4	0.000219	0.465786709	down
ASMM9PARTA003754		0.0000447	0.137530122	down
ASMM9PARTA047376	AK038606	0.002498919	0.443905523	down
ASMM9PARTA003416		0.032367382	0.110696589	down
ASMM9PARTA003416		0.032367382	0.110696589	down
CUST_185_PI426409190		0.000166	0.375639818	down
ASMM9PARTA049776	4933409K07Rik	0.001588723	0.477583998	down
ASMM9PARTA001480		0.001743645	0.487042953	down
ASMM9PARTA008552		0.001418641	0.360786439	down
ASMM9PARTA013751	Mup-ps8	0.00092	0.37462312	down
ASMM9PARTA051561		0.000069	0.328938063	down
ASMM9PARTA000231		0.013362653	0.327757479	down

AK139568_P1	humanlincRNA0333	0.000000471	0.285184714	down
ASMM9PARTA004804		0.00000439	0.28165144	down
ASMM9PARTA013055	Gm15488	0.03341248	0.442977814	down
MM9LINCRNAEXON10979-_P1	mouselincRNA0870	0.011793463	0.377246399	down
ASMM9PARTA010282	Gm14622	0.037775133	0.435901226	down
ASMM9PARTA012440	Mup-ps9	0.0000877	0.359256117	down
CUST_718_PI426073487	uc.239	0.00000519	0.464708848	down
ASMM9PARTA046584	AK083606	0.030209748	0.344149611	down
ASMM9PARTA012206	Mup-ps18	0.000000449	0.257153708	down
MM9LINCRNAEXON10524+_P1		0.00241745	0.211124576	down
ASMM9PARTA007875		0.03281851	0.23333765	down
MM9LINCRNAEXON11962+_P1		0.002282949	0.473719914	down
ASMM9PARTA049878	AK054344	0.024687165	0.485430107	down
ASMM9PARTA016460	Scnm1	0.003497421	0.291113001	down
ASMM9PARTA016460	Scnm1	0.003497421	0.291113001	down
ASMM9PARTA016460	Scnm1	0.003497421	0.291113001	down
ASMM9PARTA046753	AK148130	0.038980793	0.489715149	down
ASMM9PARTA018074	Gm15825	0.037588257	0.327626731	down
ASMM9PARTA045237	Wbscr25	0.04906466	0.341883731	down
ASMM9PARTA009221		0.031619318	0.448748098	down
ASMM9PARTA000459		0.022845458	0.341923793	down
ASMM9PARTA010972	Mup-ps6	0.00017	0.378956787	down
ASMM9PARTA007969		0.042256314	0.478996001	down
MM9LINCRNAEXON10686+_P1	mouselincRNA1112	0.008306421	0.427966626	down
ASMM9PARTA001994		0.007103732	0.441272188	down
ASMM9PARTA001994		0.007103732	0.441272188	down
BY303620_P1	humanlincRNA0185	0.025144543	0.247526897	down
ASMM9PARTA010242	Gm13303	0.00206964	0.472094851	down
ASMM9PARTA008294		0.036973476	0.381051643	down
CUST_930_PI426073487	uc.451	0.001019414	0.334900825	down
ASMM9PARTA015024	1700084C06Rik	0.00405092	0.435887489	down
ASMM9PARTA015024	1700084C06Rik	0.00405092	0.435887489	down
ASMM9PARTA015024	1700084C06Rik	0.00405092	0.435887489	down
ASMM9PARTA004720		0.003627082	0.281434582	down
ASMM9PARTA049717	AK054442	0.04830087	0.34250093	down
MM9LINCRNAEXON10980-_P1	mouselincRNA0870	0.017233474	0.262477133	down
ASMM9PARTA018910	RP24-354E11.1	0.002419276	0.490134812	down
ASMM9PARTA002885		0.000145	0.26056117	down
ASMM9PARTA051544	AK149842	0.04774288	0.473746216	down
humanlincRNA0563+_P1	humanlincRNA0563	0.030669505	0.396400902	down
ASMM9PARTA008695		0.011885835	0.169646496	down
ASMM9PARTA002592		0.011317737	0.489475542	down
BY230763_P1	mouselincRNA0359	0.01940859	0.489401329	down
ASMM9PARTA004722		0.0000285	0.41771077	down
ASMM9PARTA051071	AK041267	0.018018754	0.449932116	down
ASMM9PARTA014155	9330111N05Rik	0.005453904	0.393932184	down
ASMM9PARTA002617		0.029633755	0.328758711	down
ASMM9PARTA009030		0.002954771	0.354516598	down
ASMM9PARTA015481	C030037D09Rik	0.0000856	0.470896878	down
ASMM9PARTA015481	C030037D09Rik	0.0000856	0.470896878	down
ASMM9PARTA001738		0.00000287	0.03470293	down
BC025201_P1	mouselincRNA0389	0.011639745	0.386186257	down
ASMM9PARTA049940	DQ687153	0.000000632	0.438451668	down
ASMM9PARTA006426		0.00061	0.311727471	down
ASMM9PARTA051168	AK040058	0.005162951	0.376838226	down
mouselincRNA1626+_P1	mouselincRNA1626	0.001683128	0.322159572	down
ASMM9PARTA001615		0.0000954	0.466553045	down

MM9LINCRNAEXON10453-_P1	mouselincRNA1287	0.01703895	0.462398195	down
humanlincRNA2212-_P1	humanlincRNA2212	0.000672	0.404965902	down
ASMM9PARTA006964		0.000591	0.353772292	down
ASMM9PARTA006929		0.006582165	0.235152888	down
ASMM9PARTA003873		0.016945459	0.49061334	down
ASMM9PARTA019819	RP23-122J17.9	0.00000168	0.183313184	down
ASMM9PARTA010868	Gm14868	0.017027851	0.46902674	down
ASMM9PARTA045965	Gm7271	0.008372348	0.357834244	down
mouselincRNA0166-_P1	mouselincRNA0166	0.042414196	0.328897947	down
humanlincRNA0588+_P1	humanlincRNA0588	0.002251251	0.377420373	down
ASMM9PARTA000389		0.04213884	0.186569033	down
ASMM9PARTA003829		0.022246433	0.486559523	down
ASMM9PARTA014590	4930506C21Rik	0.0000244	0.448390983	down
ASMM9PARTA014590	4930506C21Rik	0.0000244	0.448390983	down
ASMM9PARTA046008	AU019990	0.002397055	0.407011224	down
ASMM9PARTA012618	Gm12504	0.009183766	0.464597702	down
ASMM9PARTA015284	AI314831	0.000541	0.485814815	down
ASMM9PARTA016496	A630031M04Rik	0.00000875	0.405608199	down
ASMM9PARTA001674		0.0000169	0.390314319	down
ASMM9PARTA000534		0.000571	0.223818947	down
ASMM9PARTA003905		0.0000184	0.466658575	down
ASMM9PARTA007668		0.002011493	0.360835701	down
CUST_896_PI426073487	uc.417	0.020961627	0.367770073	down
ASMM9PARTA008035		0.030102266	0.47106908	down
ASMM9PARTA018337	4930428N03Rik	0.008029678	0.241634551	down
ASMM9PARTA017215	1500002F19Rik	0.0000754	0.29998977	down
BC156060_P1	mouselincRNA1038	0.0000957	0.477500533	down
BC156060_P1	mouselincRNA1038	0.0000957	0.477500533	down
BC156060_P1	mouselincRNA1038	0.0000957	0.477500533	down
BC156060_P1	mouselincRNA1038	0.0000957	0.477500533	down
BC156060_P1	mouselincRNA1038	0.0000957	0.477500533	down
ASMM9PARTA000466		0.04625773	0.25759035	down
MM9LINCRNAEXON10875+_P1		0.000031	0.33696369	down
ASMM9PARTA017438	Gm11940	0.000000158	0.36170813	down
AK133000_P1	mouselincRNA1340	0.015340349	0.399196338	down
ASMM9PARTA000567		0.007308432	0.47430968	down
CUST_744_PI426073487	uc.265	0.009108285	0.449108042	down
CUST_744_PI426073487	uc.265	0.009108285	0.449108042	down
CUST_417_PI426073487	uc.419	0.00888963	0.441513209	down
CUST_417_PI426073487	uc.419	0.00888963	0.441513209	down
ASMM9PARTA011383	Gm12550	0.04315498	0.374331869	down
ASMM9PARTA046888	AK140919	0.007853717	0.327055653	down
ASMM9PARTA046888	AK140919	0.007853717	0.327055653	down
ASMM9PARTA046888	AK140919	0.007853717	0.327055653	down
ASMM9PARTA046888	AK140919	0.007853717	0.327055653	down
ASMM9PARTA048619	AK043286	0.000663	0.329969732	down
ASMM9PARTA048619	AK043286	0.000663	0.329969732	down
ASMM9PARTA048619	AK043286	0.000663	0.329969732	down
ASMM9PARTA015461	BC024582	0.03336372	0.458112653	down
ASMM9PARTA051421	AK007249	0.000925	0.316565593	down
ASMM9PARTA009795	Gm13775	0.00000602	0.193723588	down
CUST_377_PI426073487	uc.379	0.000559	0.248450026	down
ASMM9PARTA049969	Gnpda2	0.003828351	0.395382377	down
ASMM9PARTA049969	Gnpda2	0.003828351	0.395382377	down
CUST_797_PI426073487	uc.318	0.045477662	0.454628734	down
CUST_797_PI426073487	uc.318	0.045477662	0.454628734	down
CUST_797_PI426073487	uc.318	0.045477662	0.454628734	down

ASMM9PARTA004381		0.005547478	0.413752196	down
ASMM9PARTA000860		0.03906888	0.359682653	down
ASMM9PARTA013178	Gm13300	0.0000814	0.448011753	down
ASMM9PARTA050392	M34473	0.00000928	0.469587781	down
ASMM9PARTA004363		0.027164806	0.199458371	down
ASMM9PARTA017284	Gm16762	0.002006253	0.417357567	down
ASMM9PARTA011773	Gm12135	0.043920394	0.484771721	down
ASMM9PARTA017777	2900041M22Rik	0.001522013	0.336594372	down
ASMM9PARTA051011	Pgls	0.004726845	0.236951552	down
ASMM9PARTA051011	Pgls	0.004726845	0.236951552	down
MM9LINCRNAEXON11018-_P1	mouselincRNA0899	0.010887366	0.479367657	down
mouselincRNA0085+_P1	mouselincRNA0085	0.024133481	0.464372204	down
MM9LINCRNAEXON10562+_P1	mouselincRNA1192	0.016407315	0.473812097	down
ASMM9PARTA013455	Gm13471	0.012231304	0.427353441	down
ASMM9PARTA019503	RP24-252L3.5	0.015589444	0.277631281	down
ASMM9PARTA051419	Kirrel3	0.025322517	0.425542164	down
ASMM9PARTA051419	Kirrel3	0.025322517	0.425542164	down
ASMM9PARTA051419	Kirrel3	0.025322517	0.425542164	down
ASMM9PARTA051419	Kirrel3	0.025322517	0.425542164	down
ASMM9PARTA051419	Kirrel3	0.025322517	0.425542164	down
ASMM9PARTA008003		0.00289244	0.467207151	down
ASMM9PARTA000244		0.026880158	0.292231721	down
ASMM9PARTA009486		0.000385	0.424049366	down
CUST_848_PI426073487	uc.369	0.013055729	0.482278856	down
CUST_508_PI426073487	uc.28	0.000245	0.228862526	down
CUST_508_PI426073487	uc.28	0.000245	0.228862526	down
CUST_508_PI426073487	uc.28	0.000245	0.228862526	down
ASMM9PARTA008203		0.00785283	0.478118318	down
ASMM9PARTA005439		0.016870432	0.450043972	down
humanlincRNA0919+_P1	humanlincRNA0919	0.004129909	0.49452296	down
CUST_76_PI426409190	Mup-ps11	0.00000575	0.399995808	down
CUST_76_PI426409190	Mup-ps11	0.00000575	0.399995808	down
ASMM9PARTA005424		0.009303918	0.344231117	down
ASMM9PARTA017021	Gm13003	0.000126	0.488492342	down
ASMM9PARTA004975		0.003536218	0.478789299	down
ASMM9PARTA045468	5330413P13Rik	0.0000897	0.491909128	down
ASMM9PARTA012454	Gm13230	0.000152	0.470551078	down
ASMM9PARTA000688		0.011740047	0.472755793	down
ASMM9PARTA047600	AK040806	0.004653141	0.435021795	down
mouselincRNA1300+_P1	mouselincRNA1300	0.046768308	0.493239414	down
ASMM9PARTA046216	AK158974	0.00142486	0.327664433	down
ASMM9PARTA047221	AK040557	0.002129852	0.449416994	down
ASMM9PARTA047221	AK040557	0.002129852	0.449416994	down
ASMM9PARTA047221	AK040557	0.002129852	0.449416994	down
ASMM9PARTA004636		0.04727351	0.484398118	down
ASMM9PARTA046625	AK145253	0.007752333	0.496677969	down
ASMM9PARTA014138	Gm15345	0.01325016	0.368988917	down
mouselincRNA1466+_P1	mouselincRNA1466	0.000717	0.421701608	down
ASMM9PARTA011946	Mup-ps10	0.000012	0.303728298	down
ASMM9PARTA019132	9230115E21Rik	0.000000618	0.362249409	down
ASMM9PARTA004530		0.025478927	0.34104423	down
ASMM9PARTA000326		0.009440683	0.396344515	down
ASMM9PARTA019018	5830444B04Rik	0.0000116	0.12745271	down
ASMM9PARTA019018	5830444B04Rik	0.0000116	0.12745271	down
MM9LINCRNAEXON10630-_P1	mouselincRNA1245	0.002806337	0.451422575	down
CUST_161_PI426409190		0.000000901	0.322184317	down
ASMM9PARTA003657		0.044176843	0.471255244	down

ASMM9PARTA008265		0.006280002	0.459559352	down
ASMM9PARTA047612	AK039634	1.68E-08	0.040053398	down
ASMM9PARTA048690	AK021048	0.00000481	0.399356158	down
ASMM9PARTA048690	AK021048	0.00000481	0.399356158	down
ASMM9PARTA048690	AK021048	0.00000481	0.399356158	down
mouselincRNA0995+_P1	mouselincRNA0995	0.044454772	0.457983138	down
ASMM9PARTA005211		0.00152138	0.468623699	down
ASMM9PARTA049041	Apba1	0.00631954	0.312715559	down
ASMM9PARTA003926		6.12E-09	0.023661045	down
ASMM9PARTA049764	Slc22a15	0.000707	0.457808338	down
ASMM9PARTA019394	AC113955.1	0.005920602	0.456519903	down
humanlincRNA0212-_P1	humanlincRNA0212	0.005693027	0.373063335	down
ASMM9PARTA005997		0.025030004	0.478144813	down
ASMM9PARTA050343	Ncf1	0.026508087	0.487943406	down
ASMM9PARTA013275	Gm14589	0.009195147	0.370922454	down
ASMM9PARTA012323	Olfr269-ps1	0.039926402	0.451308098	down
ASMM9PARTA008374		0.005053575	0.411963723	down
ASMM9PARTA000819		0.037536673	0.383416059	down
ASMM9PARTA000984		0.042925965	0.397053577	down
ASMM9PARTA011888	Gm13408	0.00000195	0.347003574	down
ASMM9PARTA008301		0.013398387	0.326432262	down
ASMM9PARTA018654	Ear-ps9	0.000000213	0.30711363	down
ASMM9PARTA008817		0.008489249	0.370480705	down
ASMM9PARTA048567	wtap	0.0000487	0.473886081	down
humanlincRNA0849-_P1	humanlincRNA0849	0.012689612	0.314745463	down
MM9LINCRNAEXON10820+_P1	mouselincRNA0975	0.012246273	0.493949123	down
humanlincRNA1948-_P1	humanlincRNA1948	0.002140137	0.429786527	down
ASMM9PARTA013585	Gm13935	0.0000232	0.200903301	down
ASMM9PARTA015352	Gm14818	0.026708286	0.268350635	down
ASMM9PARTA045609	Gm10649	0.003220451	0.391416043	down
ASMM9PARTA011365	Gm11900	0.04270818	0.381095789	down
ASMM9PARTA051041	Scoc	0.000505	0.056998124	down
ASMM9PARTA051041	Scoc	0.000505	0.056998124	down
ASMM9PARTA013210	Gm13220	0.045839448	0.44551594	down
ASMM9PARTA049926	Gfi1	0.022735307	0.434794613	down
ASMM9PARTA016984	Gm11963	0.001052138	0.371209372	down
ASMM9PARTA046383	AK142161	0.0000302	0.49230047	down
humanlincRNA1634+_P1	humanlincRNA1634	0.033149805	0.334557479	down
ASMM9PARTA009981	Olfr1503-ps1	0.028066704	0.376838397	down
ASMM9PARTA008484		0.034575436	0.291049082	down
ASMM9PARTA010625	Gm15087	0.000746	0.236457958	down
ASMM9PARTA046605	AK143813	0.005582141	0.44550544	down
ASMM9PARTA015097	Gm11775	0.015863145	0.495998286	down
ASMM9PARTA007713		0.026454136	0.477573278	down
MM9LINCRNAEXON11088+_P1	mouselincRNA0819	0.035720468	0.39014535	down
ASMM9PARTA050736	U20367	0.009414643	0.476952055	down
humanlincRNA1715-_P1	humanlincRNA1715	0.016344972	0.385971525	down
ASMM9PARTA002405		0.003196637	0.481768391	down
ASMM9PARTA015348	Gm15271	0.037194923	0.271406687	down
humanlincRNA2014+_P1	humanlincRNA2014	0.044148322	0.43040998	down
ASMM9PARTA012833	Olfr1174-ps	0.026136344	0.283101566	down
ASMM9PARTA045668	Hhatl	0.011752047	0.230318957	down
MM9LINCRNAEXON11908-_P1	mouselincRNA0173	0.024799671	0.493092975	down
ASMM9PARTA016295	Gm15478	0.000244	0.439029648	down
ASMM9PARTA016295	Gm15478	0.000244	0.439029648	down
ASMM9PARTA051072	AK019737	0.00000142	0.437852603	down
ASMM9PARTA051139	NR_001461	0.003234156	0.395349051	down

CUST_21_PI426073487	uc.22	0.018535253	0.38228157	down
ASMM9PARTA010260	Mup-ps2	0.000648	0.300431128	down
ASMM9PARTA013915	Gm11648	0.000414	0.252318364	down
ASMM9PARTA013915	Gm11648	0.000414	0.252318364	down
ASMM9PARTA002438		0.04776144	0.483157072	down
ASMM9PARTA050751	Usp29	0.01259547	0.37211868	down
ASMM9PARTA050751	Usp29	0.01259547	0.37211868	down
ASMM9PARTA049624	BC048720	0.00000275	0.366010775	down
CUST_240_PI426073487	uc.242	0.032857224	0.309875135	down
ASMM9PARTA007848		0.006486828	0.44319332	down
ASMM9PARTA007814		0.015760263	0.419476812	down
ASMM9PARTA019073	Gm15024	0.016301576	0.333184655	down
mouselincRNA1555+_P1	mouselincRNA1555	0.01637528	0.427548817	down
ASMM9PARTA013911	4933439C10Rik	0.004084464	0.452106136	down
ASMM9PARTA013911	4933439C10Rik	0.004084464	0.452106136	down
ASMM9PARTA018537	AC125223.1	0.020755399	0.311302546	down
ASMM9PARTA018537	AC125223.1	0.020755399	0.311302546	down
ASMM9PARTA018537	AC125223.1	0.020755399	0.311302546	down
ASMM9PARTA010967	Gm12392	0.04533273	0.291607898	down
humanlincRNA0338-_P1	humanlincRNA0338	0.000415	0.395706083	down
CUST_306_PI426073487	uc.308	0.03678968	0.385313469	down
CUST_306_PI426073487	uc.308	0.03678968	0.385313469	down
ASMM9PARTA008070		0.03284476	0.364814502	down
ASMM9PARTA047208	AK217959	0.0000211	0.31473209	down
humanlincRNA0539+_P1	humanlincRNA0539	0.006089769	0.407030624	down
ASMM9PARTA006812		0.000361	0.389461484	down
ASMM9PARTA048270	AK213404	0.0347217	0.321092165	down
ASMM9PARTA046268	AK020188	0.000875	0.409055122	down
ASMM9PARTA051177	AK015741	0.002482541	0.41219186	down
ASMM9PARTA047850	AK076852	0.000247	0.256906019	down
humanlincRNA0120-_P1	humanlincRNA0120	0.012510297	0.411163382	down
ASMM9PARTA015249	Gm15638	0.03364441	0.323691188	down
MM9LINCRNAEXON10276-_P1	mouselincRNA1493	0.03576854	0.353309125	down
ASMM9PARTA011830	Mup-ps3	0.00714584	0.448708209	down
ASMM9PARTA048043	AK086168	0.007705907	0.4987566	down
ASMM9PARTA008735		0.0000303	0.489499238	down
MM9LINCRNAEXON11073+_P1	mouselincRNA0818	0.039999366	0.375429491	down
ASMM9PARTA051476	AK043361	0.000058	0.459726425	down
ASMM9PARTA046705	AK083030	0.00000302	0.034131911	down
MM9LINCRNAEXON10944-_P1	mouselincRNA0866	0.00930478	0.450036619	down
MM9LINCRNAEXON10205+_P1	mouselincRNA1474	0.010722918	0.464065871	down
ASMM9PARTA014791	Gm15787	0.01077029	0.285222765	down
ASMM9PARTA014791	Gm15787	0.01077029	0.285222765	down
ASMM9PARTA050201	AK007223	0.002777367	0.473155298	down
ASMM9PARTA004550		0.015151206	0.319327899	down
ASMM9PARTA004550		0.015151206	0.319327899	down
ASMM9PARTA050312	AK006413	0.034992956	0.449767432	down
ASMM9PARTA005614		0.004894454	0.429567196	down
CUST_618_PI426073487	uc.139	0.016790235	0.395208727	down
AW121278_P1	humanlincRNA1024	0.000264	0.410097486	down
ASMM9PARTA003596		0.008902524	0.418853799	down
CUST_176_PI426073487	uc.178	0.01183932	0.268395203	down
ASMM9PARTA015903	4930505O20Rik	0.04516106	0.291150685	down
ASMM9PARTA046565	AK085275	0.008945847	0.470727127	down
ASMM9PARTA018881	Gm11101	0.0000607	0.408859662	down
ASMM9PARTA006438		0.011015652	0.414833552	down
MM9LINCRNAEXON11843+_P1	mouselincRNA0273	0.039404813	0.24381211	down

humanlincRNA2101-_P1	humanlincRNA2101	0.004830487	0.326456313	down
ASMM9PARTA002244		0.006534329	0.472801726	down
ASMM9PARTA046413	AK040222	0.0171797	0.360853058	down
MM9LINCRNAEXON11963+_P1		0.001109784	0.386536605	down
ASMM9PARTA018396	Gm16404	0.01583773	0.39356747	down
ASMM9PARTA019605	BC006965	0.005342174	0.431606454	down
ASMM9PARTA047394	2410018L13Rik	0.000808	0.498760257	down
ASMM9PARTA001725		0.04035243	0.499409897	down
ASMM9PARTA005441		0.010374523	0.311384145	down
ASMM9PARTA010261	Gm10601	0.000123	0.444332769	down
ASMM9PARTA050163	BC064108	0.00000299	0.189867771	down
ASMM9PARTA050163	BC064108	0.00000299	0.189867771	down
CUST_352_PI426073487	uc.354	0.012781641	0.343607212	down
BY666395_P1	humanlincRNA1447	0.009175375	0.495153316	down
ASMM9PARTA047296	AK035085	0.023991171	0.486427221	down
ASMM9PARTA046203	AK138346	0.000126	0.44220152	down
ASMM9PARTA015743	Gm16885	0.019588608	0.4030407	down
ASMM9PARTA015743	Gm16885	0.019588608	0.4030407	down
CA464087_P1	mouselincRNA0285	0.024444764	0.430259552	down
ASMM9PARTA006626		0.00000735	0.218425621	down
ASMM9PARTA050618	AK007376	0.000423	0.271595399	down
ASMM9PARTA009360	Gm8935	0.0000727	0.497841781	down
ASMM9PARTA009936	Mup-ps7	0.0000041	0.323531305	down
ASMM9PARTA012215	Mup-ps19	0.00000108	0.134253327	down
humanlincRNA2077+_P1	humanlincRNA2077	0.009696126	0.463311669	down
MM9LINCRNAEXON10811+_P1	mouselincRNA0961	0.001297182	0.446342531	down
ASMM9PARTA048115	AK077272	0.039455798	0.40453225	down
ASMM9PARTA047918	AK014089	0.0000294	0.386305411	down
ASMM9PARTA010235	Gm13300	0.000089	0.410689458	down
CUST_103_PI426409190	AC087559.5	0.0000727	0.475954765	down
ASMM9PARTA004298		0.00000376	0.218935811	down
MM9LINCRNAEXON10635-_P1	mouselincRNA1245	0.007669432	0.3648854	down
ASMM9PARTA016186	Gm14257	0.045413513	0.387348321	down
ASMM9PARTA012583	Gm15368	0.042070813	0.442027458	down
ASMM9PARTA012583	Gm15368	0.042070813	0.442027458	down
ASMM9PARTA012583	Gm15368	0.042070813	0.442027458	down
ASMM9PARTA012583	Gm15368	0.042070813	0.442027458	down
ASMM9PARTA012583	Gm15368	0.042070813	0.442027458	down
ASMM9PARTA012583	Gm15368	0.042070813	0.442027458	down
ASMM9PARTA009906	A630012P03Rik	0.020752816	0.459634549	down
humanlincRNA1315-_P1	humanlincRNA1315	0.016093252	0.454754166	down
CUST_314_PI426073487	uc.316	0.014083947	0.4444209	down
ASMM9PARTA016224	4930412C18Rik	0.00000115	0.395896862	down
MM9LINCRNAEXON11745+_P1	mouselincRNA0230	0.00775124	0.422867043	down
MM9LINCRNAEXON11042-_P1	mouselincRNA0924	0.032320537	0.453269172	down
ASMM9PARTA014317	Gm14120	0.00607155	0.477571203	down
ASMM9PARTA015931	Mup-ps2	0.002759554	0.295455565	down
ASMM9PARTA051808		0.006495231	0.391673952	down
ASMM9PARTA006938		0.00106378	0.424485513	down
ASMM9PARTA047717	AK138307	0.0000369	0.287666017	down
ASMM9PARTA010666	Gm13890	0.031049656	0.372490587	down
ASMM9PARTA047548	AK016040	0.029687358	0.313830149	down
ASMM9PARTA002086		0.0000126	0.365683861	down
ASMM9PARTA003790		0.007693226	0.47295602	down
ASMM9PARTA048192	AK080258	0.025654526	0.326510632	down
MM9LINCRNAEXON10774-_P1	mouselincRNA1143	0.002725833	0.49690883	down
humanlincRNA1204+_P1	humanlincRNA1204	0.047498424	0.434723902	down
