# Supplementary material for: Microarray Profile of Long Noncoding RNA and Messenger RNA Expression in a Model of Alzheimer’s Disease
Source: Life (Basel). 2020 May 14;10(5):64. doi: 10.3390/life10050064 (PMC7281340; doi:10.3390/life10050064)
Supplement: Supplementary file 1 [file life-10-00064-s001.zip › life-787240-supplemenatry-to be published - PDF/life-787240-supplementary/Table S3.pdf]

life

MDPI

Supplementary

Microarray Profile of Long Noncoding RNA and Messenger RNA Expression in a Model of Alzheimer's Disease

Linlin Wang †, Li Zeng †, Hailun Jiang, Zhuorong Li * and Rui Liu *

Institute of Medicinal Biotechnology, Chinese Academy of Medical Sciences and Peking Union Medical College, Beijing 100050, China; wanglinlin@wfmc.edu.cn (L.W.); zengsheng@imb.pumc.edu.cn (L.Z.); jianghailun@imb.pumc.edu.cn (H.J.)

* Correspondence: lizhourong@imb.pumc.edu.cn (Z.L.); +86-10-8352017; .liurui@imb.pumc.edu.cn (R.L.); Tel.: +86-10-67087731

Table S3. Differently expressed lncRNAs in the brain of 6-month-old APP/PS1 mice compared with age-matched WT mice.

Probe Name	Gene Symbol	*p*-Value	Fold Change	Regulation
ASMM9PARTA045031	Abhd1	0.000000243	3.1196003	up
ASMM9PARTA045031	Abhd1	0.000000243	3.1196003	up
MM9LINCRNAEXON10451+_P1	mouselincRNA1286	0.003538504	2.2654934	up
MM9LINCRNAEXON10103-_P1	mouselincRNA1524	0.006277942	2.1662855	up
ASMM9PARTA045726	4933436C20Rik	0.000129	2.420025	up
ASMM9PARTA017102	A230108P19Rik	0.021290345	2.4069388	up
MM9LINCRNAEXON11250-_P1	mouselincRNA0737	0.004286149	2.6884916	up
ASMM9PARTA010435	Gm11019	0.025719354	2.0346353	up
humanlincRNA1590+_P1	humanlincRNA1590	0.023626897	3.5407078	up
ASMM9PARTA017171	4732418A04Rik	0.000194	4.9253507	up
ASMM9PARTA046759	AK039862	0.001835104	4.3281155	up
ASMM9PARTA018203	NRON.1	0.0000172	3.3017004	up
ASMM9PARTA016545	Gm16706	0.011395447	2.4015005	up
ASMM9PARTA016545	Gm16706	0.011395447	2.4015005	up
ASMM9PARTA008337		0.003564729	2.6720827	up
ASMM9PARTA005648		0.016110636	2.1099603	up
ASMM9PARTA000497		0.036847852	2.0237134	up
ASMM9PARTA010873	Gm15382	0.001595537	2.52695	up
MM9LINCRNAEXON11181-_P1	mouselincRNA0672	0.025258476	2.2143273	up
ASMM9PARTA012176	Gm15719	0.01595798	2.4363487	up
ASMM9PARTA004257		0.001162169	2.5316021	up
mouselincRNA0394-_P1	mouselincRNA0394	0.04695368	2.2102969	up
ASMM9PARTA015256	9130017K11Rik	0.001580209	3.02434	up
ASMM9PARTA015256	9130017K11Rik	0.001580209	3.02434	up

ASMM9PARTA050284	Gak	0.009486467	2.5133553	up
ASMM9PARTA046970	BC006965	0.042283867	2.0452976	up
CA559477_P1	humanlincRNA2085	0.004959819	2.3531377	up
ASMM9PARTA045330	Gm11149	0.000195	2.4911754	up
ASMM9PARTA045330	Gm11149	0.000195	2.4911754	up
ASMM9PARTA045330	Gm11149	0.000195	2.4911754	up
CA465684_P1	mouselincRNA0967	0.015294251	2.8427927	up
ASMM9PARTA046506	mKIAA4192	0.001182393	2.629909	up
ASMM9PARTA014585	Gm8093	0.008273289	2.3559117	up
ASMM9PARTA005223		0.008885149	2.1005719	up
ASMM9PARTA047932	AK135804	0.006776804	2.0336554	up
ASMM9PARTA012883	Gm12706	0.000925	2.0907204	up
ASMM9PARTA003398		0.001732132	2.971151	up
ASMM9PARTA007669		0.024372283	2.0133264	up
ASMM9PARTA008318		0.00395549	2.3784897	up
ASMM9PARTA049385	AK137033	0.002817194	2.1945105	up
CUST_282_PI426073487	uc.284	0.003614473	4.0671477	up
ASMM9PARTA013061	Gm13913	0.002891015	4.452653	up
ASMM9PARTA049079	AK047177	0.026932668	3.2706022	up
ASMM9PARTA017430	A830052D11Rik	0.000927	2.1083608	up
ASMM9PARTA017430	A830052D11Rik	0.000927	2.1083608	up
humanlincRNA1365-_P1	humanlincRNA1365	0.049885552	2.178999	up
ASMM9PARTA016815	Gm16534	0.011682607	2.007548	up
ASMM9PARTA017337	1700021L23Rik	0.005163256	2.999624	up
ASMM9PARTA007917		0.00391314	3.420385	up
ASMM9PARTA000849		0.017323356	3.2378094	up
CUST_714_PI426073487	uc.235	0.002783289	3.806529	up
ASMM9PARTA045466	F930015N05Rik	0.000606	2.0401404	up
MM9LINCRNAEXON11111-_P1	mouselincRNA0828	0.0000201	2.4992845	up
ASMM9PARTA005451		0.003567096	2.315891	up
ASMM9PARTA000911		0.006294135	2.3552628	up
ASMM9PARTA017280	Gm13976	0.01812167	3.2009199	up
ASMM9PARTA011323	Gm12677	0.003474014	2.3821118	up
ASMM9PARTA014414	5430427M07Rik	0.039535355	3.2116146	up
ASMM9PARTA018542	AC164629.8	0.001232109	3.675345	up
humanlincRNA2028-_P1	humanlincRNA2028	0.03381314	2.6891527	up
ASMM9PARTA049439	mKIAA1125	0.019081455	2.6283262	up
ASMM9PARTA006577		0.000000996	2.6155295	up
ASMM9PARTA003621		0.030045127	2.2775762	up
BG082317_P1	mouselincRNA0895	0.030177962	2.0221033	up
ASMM9PARTA007950		0.027898293	2.5250096	up
ASMM9PARTA003250		0.014591098	2.076166	up
ASMM9PARTA003250		0.014591098	2.076166	up
ASMM9PARTA015353	Gm1720	0.009685411	2.0152292	up
ASMM9PARTA048047	Pigz	0.000126	3.1346016	up
ASMM9PARTA011710	Gm13689	0.014496394	2.728053	up
ASMM9PARTA050830	AK081140	0.00000329	2.5226064	up
ASMM9PARTA050830	AK081140	0.00000329	2.5226064	up
ASMM9PARTA050830	AK081140	0.00000329	2.5226064	up

humanlincRNA1576-_P1	humanlincRNA1576	0.000791	2.148372	up
ASMM9PARTA001768		0.037898626	2.1554246	up
humanlincRNA1422+_P1	humanlincRNA1422	0.003613522	3.3776646	up
MM9LINCRNAEXON10275-_P1	mouselincRNA1493	0.012053111	2.185643	up
ASMM9PARTA010627	Gm15626	0.014767407	2.0154865	up
ASMM9PARTA049386	AK158189	0.04751108	2.8660064	up
ASMM9PARTA047940	AK014435	0.000127	7.87863	up
CUST_84_PI426409190	Gm16404	0.0000736	3.7620046	up
ASMM9PARTA008118		0.030992094	2.8354397	up
ASMM9PARTA011622	Gm12377	0.015353539	2.2520926	up
ASMM9PARTA005824		0.004013028	3.8872156	up
ASMM9PARTA008763		0.002697579	3.2295306	up
ASMM9PARTA006453		0.003511864	2.7597766	up
ASMM9PARTA015236	Gm14161	0.028390415	2.4637067	up
MM9LINCRNAEXON10551-_P1	mouselincRNA1185	0.007204602	2.217983	up
ASMM9PARTA010558	Xlr5d-ps	0.024622032	3.307442	up
ASMM9PARTA010441	Gm15560	0.000172	2.100406	up
ASMM9PARTA045751	Gm833	0.001627522	2.0532517	up
ASMM9PARTA015224	BC065397	0.000651	4.5189176	up
ASMM9PARTA015224	BC065397	0.000651	4.5189176	up
ASMM9PARTA015224	BC065397	0.000651	4.5189176	up
ASMM9PARTA015224	BC065397	0.000651	4.5189176	up
ASMM9PARTA015224	BC065397	0.000651	4.5189176	up
ASMM9PARTA015224	BC065397	0.000651	4.5189176	up
ASMM9PARTA015224	BC065397	0.000651	4.5189176	up
ASMM9PARTA015224	BC065397	0.000651	4.5189176	up
AI507909_P1	humanlincRNA1257	0.003566771	2.365197	up
ASMM9PARTA046107	AK039957	0.035611473	2.0791461	up
ASMM9PARTA010032	Gm5301	0.029425412	2.8055193	up
ASMM9PARTA018293	Gm16537	0.000716	2.1571558	up
ASMM9PARTA008417		0.014867369	2.1852226	up
ASMM9PARTA011704	Gm13888	0.009480914	2.3480234	up
ASMM9PARTA011738	Gm15470	0.000384	2.2918737	up
ASMM9PARTA006207		0.027935762	2.6583912	up
ASMM9PARTA048537	NR_002853	0.0000136	5.046747	up
ASMM9PARTA000492		0.000442	3.1650398	up
ASMM9PARTA050019	AK148461	0.007182398	2.8180325	up
ASMM9PARTA005495		0.002728953	2.8448937	up
ASMM9PARTA007865		0.001723707	3.2370198	up
ASMM9PARTA007417		0.006146743	4.9098845	up
ASMM9PARTA013734	Gm13131	0.0000777	2.2333233	up
ASMM9PARTA013190	Gm7386	0.002716446	2.9815123	up
CUST_132_PI426409190	7SK.216	0.0000836	2.469293	up
MM9LINCRNAEXON10332-_P1		0.03909866	2.6996117	up
ASMM9PARTA013040	Gm13577	0.000453	2.3502493	up
ASMM9PARTA019232	Gm14957	0.0000198	2.017383	up
MM9LINCRNAEXON10455-_P1	mouselincRNA1287	0.024758393	2.0509887	up
CUST_184_PI426409190		0.021882309	2.307146	up
BI557075_P1	mouselincRNA0751	0.020051941	2.1689298	up

ASMM9PARTA010114	Gm11041	1.65E-08	4.5114403	up
humanlincRNA1416-_P1	humanlincRNA1416	0.005521476	2.1290872	up
ASMM9PARTA001249		0.001627436	3.3796606	up
ASMM9PARTA011188	Hmgb1-ps4	0.000216	2.3263745	up
BB869594_P1	mouselincRNA1003	0.024265677	3.3511324	up
ASMM9PARTA046284	AK036470	0.0444964	4.350874	up
ASMM9PARTA017913	Gm12440	0.000271	2.851259	up
MM9LINCRNAEXON11222+_P1	mouselincRNA0710	0.009465001	2.1307418	up
ASMM9PARTA007699		0.006026872	2.786616	up
ASMM9PARTA011747	Gm8809	0.0034172	2.5957918	up
BI180507_P1	mouselincRNA1445	0.04222658	3.1212835	up
CX210663_P1	humanlincRNA1977	0.045880754	2.0853064	up
ASMM9PARTA002112		0.001073549	2.2244182	up
ASMM9PARTA013980	2810403D21Rik	0.0170817	2.5690312	up
ASMM9PARTA048349	AK018679	0.000471	3.159507	up
ASMM9PARTA047160	Slc38a6	0.000477	2.5141454	up
ASMM9PARTA047160	Slc38a6	0.000477	2.5141454	up
ASMM9PARTA015335	B230314M03Rik	0.02093286	3.4851682	up
ASMM9PARTA017631	Gm5755	0.024254544	2.2938702	up
MM9LINCRNAEXON11010-_P1	mouselincRNA0889	0.03270752	4.374618	up
humanlincRNA0409-_P1	humanlincRNA0409	0.04526558	2.114595	up
ASMM9PARTA014856	1810059H22Rik	0.024508571	2.0675554	up
ASMM9PARTA015039	Gm15462	0.008491262	2.2994695	up
ASMM9PARTA018383	Gm16551	0.0000014	3.6906974	up
ASMM9PARTA018240	7SK.318	0.009609278	4.277669	up
ASMM9PARTA047628	AK143833	0.00163815	2.0070548	up
MM9LINCRNAEXON10775+_P1	mouselincRNA1143	0.000213	2.9503467	up
ASMM9PARTA050633	AK006412	0.019480785	2.2714992	up
ASMM9PARTA046039	LOC100415784	0.02861781	2.5691552	up
ASMM9PARTA001180		0.04690896	2.4605153	up
ASMM9PARTA008433		0.005605161	3.513392	up
ASMM9PARTA003776		0.001823768	2.083925	up
ASMM9PARTA009988	4930473A02Rik	0.007433488	2.2167606	up
ASMM9PARTA009988	4930473A02Rik	0.007433488	2.2167606	up
ASMM9PARTA009988	4930473A02Rik	0.007433488	2.2167606	up
CA749839_P1	humanlincRNA1455	0.036991995	3.0595503	up
ASMM9PARTA005626		0.02361701	3.3478467	up
ASMM9PARTA013524	Gm16407	0.012301705	2.224433	up
ASMM9PARTA005747		0.000308	2.266706	up
ASMM9PARTA019355	RP23-141L18.6	0.006608017	2.0787408	up
ASMM9PARTA048530	mKIAA0136	0.009078536	2.3973286	up
MM9LINCRNAEXON10991+_P1		0.008344797	2.318909	up
ASMM9PARTA002929		0.04909547	2.9300334	up
ASMM9PARTA046040	4931406H21Rik	0.004570165	2.43949	up
ASMM9PARTA001979		0.001217148	2.6140153	up
ASMM9PARTA005628		0.001928635	2.7167344	up
ASMM9PARTA019050	RP24-252L3.2	0.00021	2.3131325	up
ASMM9PARTA015438	B430212C06Rik	0.037105132	2.1464314	up
ASMM9PARTA005761		0.000184	2.7226276	up

ASMM9PARTA019063	AC125279.2	0.003924265	2.026877	up
mouselincRNA0953+_P1	mouselincRNA0953	0.017413868	2.180299	up
ASMM9PARTA011894	Gm14593	0.000797	2.0784206	up
MM9LINCRNAEXON11384+_P1	mouselincRNA0595	0.023294466	2.2980533	up
ASMM9PARTA017049	Gm2694	0.000232	9.320407	up
ASMM9PARTA016462	Gm14635	0.038758814	2.3224788	up
CUST_909_PI426073487	uc.430	0.00012	8.662708	up
CUST_909_PI426073487	uc.430	0.00012	8.662708	up
CUST_909_PI426073487	uc.430	0.00012	8.662708	up
CUST_909_PI426073487	uc.430	0.00012	8.662708	up
CUST_909_PI426073487	uc.430	0.00012	8.662708	up
ASMM9PARTA019483	AC153382.1	0.03434507	4.1659966	up
MM9LINCRNAEXON11361-_P1	mouselincRNA0586	0.023001742	2.4966457	up
ASMM9PARTA005615		0.01960919	2.7944953	up
MM9LINCRNAEXON10538-_P1	mouselincRNA1178	0.033266254	2.4831288	up
MM9LINCRNAEXON11092+_P1	mouselincRNA0819	0.036621634	2.003259	up
ASMM9PARTA048151	AK085876	0.047738314	3.8536763	up
ASMM9PARTA048151	AK085876	0.047738314	3.8536763	up
ASMM9PARTA048151	AK085876	0.047738314	3.8536763	up
ASMM9PARTA006865		0.000446	4.9645433	up
ASMM9PARTA048808	AK081497	0.00000402	2.214474	up
ASMM9PARTA004660		0.005279298	2.5740352	up
ASMM9PARTA047373	AK141540	0.049087267	2.6281195	up
ASMM9PARTA045220	Tmem181c-ps	0.026408298	2.090798	up
ASMM9PARTA005238		0.002142166	2.8474588	up
ASMM9PARTA010309	Gm3371	0.000688	2.8403668	up
ASMM9PARTA004795		0.013844482	2.466936	up
ASMM9PARTA015594	D430001F17Rik	0.003473096	2.2148507	up
ASMM9PARTA006197		0.03481218	3.0301728	up
ASMM9PARTA001200		0.037559688	2.3540723	up
ASMM9PARTA003874		0.0000143	2.6614656	up
ASMM9PARTA044891	1700108J01Rik	0.012962443	2.6089635	up
ASMM9PARTA046659	4933439F18Rik	0.00000331	2.7995448	up
ASMM9PARTA046659	4933439F18Rik	0.00000331	2.7995448	up
MM9LINCRNAEXON11316+_P1	mouselincRNA0662	0.0158313	3.1894422	up
humanlincRNA1824+_P1	humanlincRNA1824	0.043534677	3.7175477	up
mouselincRNA0757+_P1	mouselincRNA0757	0.04705886	4.155268	up
ASMM9PARTA051750	Smpx	0.000694	11.515533	up
humanlincRNA2404-_P1	humanlincRNA2404	0.016887693	2.9459364	up
ASMM9PARTA049669	AK053689	0.00000821	2.3798423	up
ASMM9PARTA049669	AK053689	0.00000821	2.3798423	up
ASMM9PARTA049669	AK053689	0.00000821	2.3798423	up
ASMM9PARTA049669	AK053689	0.00000821	2.3798423	up
ASMM9PARTA049669	AK053689	0.00000821	2.3798423	up
ASMM9PARTA049669	AK053689	0.00000821	2.3798423	up
CUST_141_PI426073487	uc.143	0.020154553	2.8944547	up
CUST_141_PI426073487	uc.143	0.020154553	2.8944547	up
CUST_141_PI426073487	uc.143	0.020154553	2.8944547	up
CUST_141_PI426073487	uc.143	0.020154553	2.8944547	up

CUST_141_PI426073487	uc.143	0.020154553	2.8944547	up
humanlincRNA0170-_P1	humanlincRNA0170	0.001058076	3.5001497	up
CUST_707_PI426073487	uc.228	0.037611984	2.0780194	up
ASMM9PARTA004940		0.0000236	4.5353403	up
ASMM9PARTA001304		0.023967667	3.30965	up
ASMM9PARTA004865		0.044790927	2.4650948	up
ASMM9PARTA006224		0.002246094	2.7445166	up
ASMM9PARTA006230		0.019308556	2.3876908	up
ASMM9PARTA002107		0.003759661	2.4878573	up
ASMM9PARTA004069		0.00000168	2.2173553	up
ASMM9PARTA013403	Gm13108	0.001053804	3.019906	up
ASMM9PARTA004112		0.000111	2.1247754	up
CUST_820_PI426073487	uc.341	0.034044523	2.0059185	up
ASMM9PARTA003242		0.000814	3.5117667	up
ASMM9PARTA006070		0.0391647	2.7844713	up
ASMM9PARTA018880	RP24-494O2.2	0.00451887	2.3739965	up
ASMM9PARTA003546		0.012069855	2.6409264	up
ASMM9PARTA017501	Gm15179	0.040958084	7.105899	up
ASMM9PARTA049106	AK048878	0.030526496	2.5606627	up
ASMM9PARTA005085		0.002507699	2.7155657	up
ASMM9PARTA004587		0.02057685	3.3519788	up
ASMM9PARTA004417		0.034157258	2.1888185	up
ASMM9PARTA001525		0.024316404	2.3973048	up
ASMM9PARTA014282	Gm15991	0.007740527	2.3824847	up
ASMM9PARTA015617	Gm15133	0.04867951	2.364248	up
ASMM9PARTA049885	ADAM22	0.032752007	4.200696	up
ASMM9PARTA049885	ADAM22	0.032752007	4.200696	up
ASMM9PARTA049885	ADAM22	0.032752007	4.200696	up
ASMM9PARTA008175		0.040986877	2.2441556	up
ASMM9PARTA001618		0.001134463	2.6449451	up
ASMM9PARTA046608	Mif1	0.0000083	2.9739807	up
BG085481_P1	mouselincRNA1465	0.009626612	2.0220177	up
ASMM9PARTA014753	Gm12603	0.000979	3.1681597	up
ASMM9PARTA005164		0.000171	2.4501905	up
MM9LINCRNAEXON10071-_P1	mouselincRNA1506	0.015053556	3.0902917	up
ASMM9PARTA005034		0.002631368	2.4690459	up
humanlincRNA1862+_P1	humanlincRNA1862	0.0310584	3.0636132	up
mouselincRNA0617-_P1	mouselincRNA0617	0.006884662	2.308748	up
MM9LINCRNAEXON11571+_P1	mouselincRNA0391	0.003432522	4.0042496	up
ASMM9PARTA003751		0.00000084	12.310973	up
ASMM9PARTA011562	Gm15039	0.000211	2.456428	up
MM9LINCRNAEXON10841-_P1	mouselincRNA0984	0.027590638	2.7379727	up
ASMM9PARTA003610		0.042254943	3.072782	up
ASMM9PARTA016939	Gm12063	0.028061293	2.6095011	up
mouselincRNA0327+_P1	mouselincRNA0327	0.002864747	4.3478937	up
MM9LINCRNAEXON10848+_P1	mouselincRNA0988	0.027104352	3.9283922	up
humanlincRNA1007-_P1	humanlincRNA1007	0.005941539	2.9665976	up
CUST_124_PI426409190	AC142215.1	0.021953136	2.875312	up
ASMM9PARTA049290	AK139560	0.029100709	2.248473	up

ASMM9PARTA019265	RP23-48M16.7	0.002356861	2.485483	up
MM9LINCRNAEXON11079+_P1	mouselincRNA0819	0.0000116	2.0746212	up
ASMM9PARTA004293		0.000486	2.1103024	up
ASMM9PARTA018549	1810009N23Rik	0.02632637	2.1044378	up
ASMM9PARTA018549	1810009N23Rik	0.02632637	2.1044378	up
ASMM9PARTA018549	1810009N23Rik	0.02632637	2.1044378	up
ASMM9PARTA016660	Gm15342	0.006230607	2.530776	up
ASMM9PARTA016660	Gm15342	0.006230607	2.530776	up
ASMM9PARTA008119		0.008739894	3.5971806	up
ASMM9PARTA008119		0.008739894	3.5971806	up
ASMM9PARTA008119		0.008739894	3.5971806	up
ASMM9PARTA008441		0.008680396	2.9530218	up
ASMM9PARTA008441		0.008680396	2.9530218	up
ASMM9PARTA045636	Ngrn	0.0000224	2.6480951	up
ASMM9PARTA005106		0.030218247	3.7436645	up
ASMM9PARTA005106		0.030218247	3.7436645	up
MM9LINCRNAEXON11076-_P1		0.000243	3.0707293	up
ASMM9PARTA001210		0.006707199	2.4725158	up
ASMM9PARTA001210		0.006707199	2.4725158	up
ASMM9PARTA008966		0.017784009	2.3902009	up
humanlincRNA1751+_P1	humanlincRNA1751	0.005374423	11.642016	up
MM9LINCRNAEXON11077-_P1		0.007299297	2.1697588	up
ASMM9PARTA009943	Gm8062	0.006803574	2.5826466	up
ASMM9PARTA008187		0.03344953	2.8776648	up
ASMM9PARTA017775	1700080N15Rik	0.049553793	2.2832606	up
ASMM9PARTA018736	Gm15509	0.016165158	2.2104762	up
ASMM9PARTA047903	AK039376	0.000748	5.429104	up
MM9LINCRNAEXON12070+_P1	mouselincRNA0095	0.029400904	3.0402145	up
ASMM9PARTA049600	AK080367	0.003822616	2.8285954	up
ASMM9PARTA006133		0.024575898	4.3784523	up
ASMM9PARTA007358		0.0000158	3.1322258	up
ASMM9PARTA009806	Kat2b-ps	0.000168	2.9269757	up
ASMM9PARTA046612	mKIAA0946	0.002403481	2.253309	up
ASMM9PARTA007218		0.004203985	2.2174945	up
ASMM9PARTA005923		0.046916507	2.3488636	up
mouselincRNA0632-_P1	mouselincRNA0632	0.047904536	2.291838	up
ASMM9PARTA005487		0.015877029	2.7783203	up
BU700229_P1	humanlincRNA0480	0.007076173	3.0498855	up
AA612090_P1	humanlincRNA2042	0.014061171	2.833393	up
MM9LINCRNAEXON10912+_P1		0.04281604	2.326849	up
ASMM9PARTA006952		0.000212	2.933043	up
ASMM9PARTA015366	Gm13941	0.004194527	2.2182326	up
ASMM9PARTA048905	AK039403	0.00581306	2.630781	up
ASMM9PARTA016699	Gm11274	0.000149	2.0988553	up
ASMM9PARTA002798		0.016208883	3.5863788	up
ASMM9PARTA004219		0.029434249	2.437581	up
ASMM9PARTA045026	Nctc1	0.048704736	8.675903	up
ASMM9PARTA004020		0.002604622	2.8132827	up
ASMM9PARTA003330		0.000059	2.087925	up

ASMM9PARTA017311	Gm15767	0.01592545	2.0210102	up
ASMM9PARTA048789	AK196959	0.006345866	3.056914	up
ASMM9PARTA046723	AK087691	0.04555099	2.386392	up
ASMM9PARTA008215		0.027094787	2.175607	up
MM9LINCRNAEXON12111-_P1	mouselincRNA0122	0.001980354	2.632564	up
ASMM9PARTA019467	RP24-318H2.3	0.021552607	5.180711	up
MM9LINCRNAEXON10237-_P1	mouselincRNA1492	0.005712733	2.2694352	up
MM9LINCRNAEXON11232+_P1	mouselincRNA0719	0.000351	3.2957702	up
ASMM9PARTA008541		0.010017832	2.381682	up
ASMM9PARTA004196		0.026486952	3.3065698	up
ASMM9PARTA010185	Gm12568	0.0000167	2.1588042	up
ASMM9PARTA011470	Gm13106	0.006857961	2.5908992	up
ASMM9PARTA045533	Gm11213	0.000566	2.0588064	up
ASMM9PARTA008501		0.008320432	3.2261076	up
CUST_596_PI426073487	uc.117	0.039768543	2.2010832	up
ASMM9PARTA046838	AK138505	0.003567821	2.2114522	up
ASMM9PARTA046838	AK138505	0.003567821	2.2114522	up
ASMM9PARTA046838	AK138505	0.003567821	2.2114522	up
CUST_407_PI426073487	uc.409	0.001603269	2.305996	up
humanlincRNA0518+_P1	humanlincRNA0518	0.002420107	9.017064	up
ASMM9PARTA010683	Gm14981	0.029760793	2.2073135	up
humanlincRNA1546-_P1	humanlincRNA1546	0.0000944	2.0835197	up
ASMM9PARTA002361		0.0000749	2.348999	up
AK145365_P1	mouselincRNA1288	0.004732965	2.7883756	up
ASMM9PARTA018655	Gm10075	0.0000383	2.2078483	up
MM9LINCRNAEXON11321+_P1	mouselincRNA0524	0.003361886	2.7437582	up
ASMM9PARTA050637	smarp	0.001898545	3.6767123	up
BU962743_P1	mouselincRNA1083	0.023437398	4.246538	up
ASMM9PARTA017459	Hoxb3os	0.0000226	3.595772	up
ASMM9PARTA017459	Hoxb3os	0.0000226	3.595772	up
ASMM9PARTA017459	Hoxb3os	0.0000226	3.595772	up
ASMM9PARTA002719		0.000633	3.0601602	up
ASMM9PARTA005665		0.033535264	2.1038098	up
BY207821_P1	mouselincRNA0550	0.04907656	2.0571024	up
ASMM9PARTA006951		0.000108	2.0818617	up
ASMM9PARTA014684	2310050B05Rik	0.048039746	3.769259	up
ASMM9PARTA014684	2310050B05Rik	0.048039746	3.769259	up
ASMM9PARTA000801		0.006958491	3.5187576	up
ASMM9PARTA007224		0.006875541	3.3408072	up
ASMM9PARTA013503	Gm14804	0.03480002	2.4201102	up
ASMM9PARTA002839		0.003558714	2.2040365	up
ASMM9PARTA048120	AK017296	0.003308056	2.1203625	up
ASMM9PARTA048120	AK017296	0.003308056	2.1203625	up
ASMM9PARTA045178	Sycp1-ps1	1.14E-08	40.444847	up
ASMM9PARTA045616	Serpina3h	0.000526	2.661606	up
BX636194_P1	mouselincRNA1032	0.0000755	3.3333285	up
ASMM9PARTA005646		0.000167	2.908214	up
ASMM9PARTA005646		0.000167	2.908214	up
CUST_49_PI426409190	AK076905	0.03077829	3.6216407	up

mouselincRNA0735-_P1	mouselincRNA0735	0.026726022	2.2493627	up
ASMM9PARTA048388	C21orf66	0.003340053	2.0076077	up
ASMM9PARTA007593		0.004044956	2.2138622	up
ASMM9PARTA046706	AK082467	0.0000126	5.086711	up
ASMM9PARTA045201	Gm10565	0.049089514	2.3213904	up
ASMM9PARTA050227	BC028660	0.024512043	2.682108	up
ASMM9PARTA006971		0.000262	2.3942513	up
MM9LINCRNAEXON12056-_P1	mouselincRNA0073	6.97E-08	4.2592525	up
ASMM9PARTA017493	Gm15692	0.0000751	2.005298	up
ASMM9PARTA017493	Gm15692	0.0000751	2.005298	up
ASMM9PARTA007175		0.002617595	2.0595605	up
ASMM9PARTA046627	BC023483	0.00000625	2.1630623	up
ASMM9PARTA015968	Gm11521	0.002147284	2.4110267	up
humanlincRNA1010-_P1	humanlincRNA1010	0.009802205	2.9109712	up
BI108441_P1	mouselincRNA0637	0.03589324	2.610662	up
BI108441_P1	mouselincRNA0637	0.03589324	2.610662	up
BI108441_P1	mouselincRNA0637	0.03589324	2.610662	up
BI108441_P1	mouselincRNA0637	0.03589324	2.610662	up
BI108441_P1	mouselincRNA0637	0.03589324	2.610662	up
BI108441_P1	mouselincRNA0637	0.03589324	2.610662	up
BI108441_P1	mouselincRNA0637	0.03589324	2.610662	up
BI108441_P1	mouselincRNA0637	0.03589324	2.610662	up
BI108441_P1	mouselincRNA0637	0.03589324	2.610662	up
ASMM9PARTA007614		0.022839185	2.4202943	up
ASMM9PARTA003563		0.003147569	2.532423	up
ASMM9PARTA002895		0.000319	2.6496823	up
ASMM9PARTA018150	Gm16551	0.000292	3.2764378	up
ASMM9PARTA000137		0.000431	2.885484	up
ASMM9PARTA046058	PAPK-A	0.000000423	2.0264783	up
ASMM9PARTA046058	PAPK-A	0.000000423	2.0264783	up
MM9LINCRNAEXON11978+_P1	mouselincRNA0222	0.001084389	2.481123	up
MM9LINCRNAEXON11855-_P1		0.004649991	2.7688546	up
MM9LINCRNAEXON11855-_P1		0.004649991	2.7688546	up
MM9LINCRNAEXON11855-_P1		0.004649991	2.7688546	up
ASMM9PARTA017917	Gm16143	0.007148983	3.7095916	up
MM9LINCRNAEXON10903-_P1		0.000983	3.012943	up
ASMM9PARTA013657	Gm9225	0.003845907	3.109786	up
ASMM9PARTA012091	Gm12262	0.002530725	3.1234307	up
MM9LINCRNAEXON10679+_P1	mouselincRNA1110	0.018026529	2.8343897	up
ASMM9PARTA048055	AK015937	0.023503464	2.124972	up
humanlincRNA0574+_P1	humanlincRNA0574	0.005158794	4.0270076	up
ASMM9PARTA046956	AK163075	0.000109	2.4392078	up
ASMM9PARTA009216		0.000849	2.533348	up
mouselincRNA1231-_P1	mouselincRNA1231	0.005538898	2.389583	up
MM9LINCRNAEXON11228-_P1	mouselincRNA0717	0.021706568	2.63723	up
ASMM9PARTA004601		0.011715838	3.3561337	up
ASMM9PARTA009446	Mageb16-ps2	0.030589927	2.1051407	up
ASMM9PARTA009446	Mageb16-ps2	0.030589927	2.1051407	up
CO802520_P1	humanlincRNA0879	0.0000066	2.5883179	up

MM9LINCRNAEXON10673+_P1	mouselincRNA1108	0.006572138	2.32608	up
ASMM9PARTA015200	1700081H22Rik	0.013389994	3.0616462	up
ASMM9PARTA004930		0.019904397	2.1912074	up
ASMM9PARTA012816	Gm12006	0.001583721	7.3320594	up
ASMM9PARTA012726	Gm5645	0.038475864	2.8579035	up
MM9LINCRNAEXON10217+_P1	mouselincRNA1479	0.016544439	2.8432105	up
ASMM9PARTA050812	AK009724	0.006954367	2.0454316	up
mouselincRNA1179+_P1	mouselincRNA1179	0.03601607	2.379505	up
ASMM9PARTA010111	Gm10977	0.0000479	2.5837293	up
MM9LINCRNAEXON10620+_P1	mouselincRNA1234	0.030851863	2.3540404	up
CUST_737_PI426073487	uc.258	0.002995235	2.1320958	up
ASMM9PARTA012436	Gm15480	0.043778133	2.0910826	up
ASMM9PARTA047044	AK133925	0.0000815	3.7127237	up
ASMM9PARTA003403		0.000492	2.1165648	up
ASMM9PARTA006650		0.045557506	2.1677904	up
CUST_600_PI426073487	uc.121	0.008882767	3.2375226	up
ASMM9PARTA016823	BC037032	0.020691348	2.032411	up
ASMM9PARTA016400	Gm12204	0.003139618	3.0570884	up
ASMM9PARTA006996		0.00206722	2.9668393	up
ASMM9PARTA002567		0.000494	3.2631228	up
ASMM9PARTA013489	Gm12071	0.027105387	2.742475	up
ASMM9PARTA008655		0.001307275	4.81924	up
ASMM9PARTA008655		0.001307275	4.81924	up
ASMM9PARTA008655		0.001307275	4.81924	up
ASMM9PARTA018423	Ear-ps8	0.008499214	2.6247387	up
ASMM9PARTA003440		0.000623	2.7618341	up
ASMM9PARTA009152		0.000451	2.2848554	up
ASMM9PARTA014524	Gm16144	0.019369109	2.0858865	up
ASMM9PARTA015108	AV039307	0.0000173	3.0902739	up
ASMM9PARTA015108	AV039307	0.0000173	3.0902739	up
ASMM9PARTA015108	AV039307	0.0000173	3.0902739	up
ASMM9PARTA015108	AV039307	0.0000173	3.0902739	up
ASMM9PARTA001481		0.005561674	2.865427	up
MM9LINCRNAEXON11830+_P1	mouselincRNA0272	0.021750178	2.0327263	up
ASMM9PARTA012145	Gm11830	0.001228827	2.2805362	up
ASMM9PARTA009768	Gm8385	0.049983535	2.176528	up
AV570450_P1	humanlincRNA0329	0.0000283	2.0598383	up
ASMM9PARTA006076		0.002344161	2.1693492	up
ASMM9PARTA005435		0.00000402	5.980378	up
ASMM9PARTA013440	Nctc1	0.016879411	4.01852	up
ASMM9PARTA048214	AK170928	0.00462574	3.4809973	up
ASMM9PARTA048214	AK170928	0.00462574	3.4809973	up
ASMM9PARTA048214	AK170928	0.00462574	3.4809973	up
ASMM9PARTA007740		0.000695	2.0377164	up
ASMM9PARTA012692	Gm9436	0.044637505	2.4307625	up
ASMM9PARTA010117	Olfr833-ps1	0.002780625	2.83065	up
ASMM9PARTA012155	Gm9043	0.007666988	2.166605	up
ASMM9PARTA006854		0.003844349	2.5852573	up
MM9LINCRNAEXON11261+_P1	mouselincRNA0611	0.021174934	2.4959385	up

ASMM9PARTA003577		0.024286496	2.25274	up
ASMM9PARTA050677	AK085609	0.02927942	2.7250395	up
ASMM9PARTA002799		0.005829373	3.6962624	up
ASMM9PARTA015078	Gm15050	0.048169628	3.3300498	up
ASMM9PARTA001730		0.002490886	3.3468084	up
ASMM9PARTA051270	AK027968	0.036336716	2.0395389	up
MM9LINCRNAEXON11619+_P1	mouselincRNA0430	0.03901233	2.3357518	up
CUST_72_PI426409190	Gm13155	0.0000926	2.2347572	up
ASMM9PARTA019336	AC132234.1	0.00000426	2.4943562	up
ASMM9PARTA016885	Gm14233	0.036307264	2.569906	up
ASMM9PARTA016885	Gm14233	0.036307264	2.569906	up
ASMM9PARTA002195		0.0179232	2.3497405	up
ASMM9PARTA002066		0.00713263	2.773211	up
CUST_57_PI426073487	uc.59	0.039604124	2.2466028	up
ASMM9PARTA016764	Gm13364	0.02203558	2.316017	up
ASMM9PARTA001716		0.000755	2.2343197	up
ASMM9PARTA006533		0.015066444	2.0008142	up
ASMM9PARTA001870		0.035680737	2.050223	up
ASMM9PARTA013831	Gm7375	0.024381729	2.5395808	up
ASMM9PARTA019160	RP24-230H12.5	0.0000243	2.4092183	up
ASMM9PARTA014536	Gm11532	0.003093464	3.0682335	up
ASMM9PARTA047419	AK031668	0.047120072	2.5715313	up
ASMM9PARTA003674		0.010078838	3.4842553	up
ASMM9PARTA010974	Gm11256	0.005050893	2.0879426	up
ASMM9PARTA012414	Gm12383	0.002204431	2.5984976	up
MM9LINCRNAEXON11454-_P1	mouselincRNA0481	0.006875185	3.946548	up
ASMM9PARTA000626		0.011875407	2.1246278	up
ASMM9PARTA048947	Strbp	0.013653743	2.5091367	up
ASMM9PARTA048947	Strbp	0.013653743	2.5091367	up
ASMM9PARTA048947	Strbp	0.013653743	2.5091367	up
ASMM9PARTA048947	Strbp	0.013653743	2.5091367	up
MM9LINCRNAEXON10665-_P1	mouselincRNA1101	0.000631	2.9414248	up
MM9LINCRNAEXON11728+_P1		0.005330466	5.3235664	up
ASMM9PARTA005235		0.007320527	2.7590816	up
ASMM9PARTA005235		0.007320527	2.7590816	up
ASMM9PARTA005235		0.007320527	2.7590816	up
ASMM9PARTA013034	Gm15379	0.009083724	2.697996	up
ASMM9PARTA015476	Hoxa11as	0.000499	2.3473916	up
CK378914_P1	humanlincRNA1553	0.004313443	3.8119788	up
ASMM9PARTA051052	AK138089	0.040168382	2.1072538	up
ASMM9PARTA017953	1700109F18Rik	0.005890129	2.3121471	up
ASMM9PARTA004458		0.002207072	2.5306082	up
ASMM9PARTA012488	Gm11283	0.008321202	3.794433	up
ASMM9PARTA005058		0.000521	2.9792387	up
ASMM9PARTA003127		0.021392446	2.0916479	up
ASMM9PARTA050768	AK020061	0.04279583	2.9761534	up
ASMM9PARTA049866	AK052888	0.017459173	2.2204773	up
mouselincRNA0238-_P1	mouselincRNA0238	0.006306958	3.0674076	up
ASMM9PARTA047894	myo 10	0.017273339	2.5141213	up

BB791535_P1	humanlincRNA2428	0.0000116	2.735673	up
ASMM9PARTA050876	AK077481	0.009983076	2.242444	up
ASMM9PARTA006154		0.000104	2.0245643	up
ASMM9PARTA014483	Gm12729	0.005225903	2.72301	up
ASMM9PARTA019549	9930038B18Rik	0.043335248	2.403109	up
ASMM9PARTA017474	0610009B14Rik	0.000000919	3.3637536	up
ASMM9PARTA050498	AK079912	0.011036999	3.5525525	up
ASMM9PARTA001256		0.040841885	2.3475182	up
ASMM9PARTA010668	Gm11905	0.049895674	3.3166773	up
ASMM9PARTA050583	AK085986	0.002406458	2.5434663	up
AI503337_P1	mouselincRNA0302	0.009075766	2.4152875	up
ASMM9PARTA005712		0.030666744	2.3799417	up
CF355830_P1	humanlincRNA0960	0.02487194	2.2648835	up
BY568487_P1	humanlincRNA0565	0.02050637	3.4512465	up
ASMM9PARTA044984	Dio3os	0.005266411	3.4866302	up
ASMM9PARTA005919		0.033191428	2.1472256	up
MM9LINCRNAEXON11077+_P1		0.00000508	3.4989035	up
ASMM9PARTA018081	Airn	0.037468906	2.248969	up
ASMM9PARTA016368	Gm15958	0.007254214	2.480366	up
ASMM9PARTA000283		0.017055232	2.1937919	up
ASMM9PARTA004641		0.014735124	2.5504835	up
ASMM9PARTA014456	C230012O17Rik	0.039158992	2.6919045	up
ASMM9PARTA051077	AK215526	0.029262166	2.1506581	up
ASMM9PARTA001427		0.01684309	2.308539	up
ASMM9PARTA001427		0.01684309	2.308539	up
MM9LINCRNAEXON10187+_P1	mouselincRNA1448	0.025601119	2.4678285	up
mouselincRNA0161+_P1	mouselincRNA0161	0.0000579	2.0598936	up
MM9LINCRNAEXON11320+_P1	mouselincRNA0663	0.010808975	2.344869	up
MM9LINCRNAEXON11530+_P1	mouselincRNA0380	0.048439283	2.4950743	up
ASMM9PARTA009332	4930463O16Rik	0.000231	3.7154183	up
BG079320_P1	humanlincRNA2275	0.013950573	2.4456704	up
ASMM9PARTA048065	KIAA0143	0.04088732	2.5226336	up
ASMM9PARTA001680		0.008360054	2.6108751	up
ASMM9PARTA047659	AK076988	0.003413783	2.5410407	up
ASMM9PARTA003081		0.040052835	2.8499064	up
ASMM9PARTA044808	Speer7-ps1	0.001616899	4.6775584	up
ASMM9PARTA005149		0.007181083	2.3194792	up
ASMM9PARTA047063	BC028808	0.027978338	2.3615115	up
ASMM9PARTA013479	Gm16479	0.001211516	2.2726977	up
ASMM9PARTA007855		0.000931	2.3980644	up
ASMM9PARTA009678	Gm5135	0.029795032	4.449114	up
ASMM9PARTA018758	Gm6413	0.000402	2.1950967	up
ASMM9PARTA048894	AK085760	0.002668793	2.4059541	up
MM9LINCRNAEXON10181+_P1	mouselincRNA1444	0.041006327	2.43731	up
ASMM9PARTA001946		0.00000344	2.5476496	up
humanlincRNA0944+_P1	humanlincRNA0944	0.000148	2.6896589	up
ASMM9PARTA049066	AK040009	0.00464691	2.0944953	up
ASMM9PARTA004299		0.011978923	3.120061	up
ASMM9PARTA007731		0.005444197	2.2421808	up

ASMM9PARTA016541	Zfp133-ps	0.003675306	5.749109	up
ASMM9PARTA003726		0.002481517	8.849999	up
ASMM9PARTA003828		0.001181659	2.3281505	up
humanlincRNA0506+_P1	humanlincRNA0506	0.003871118	2.8186488	up
ASMM9PARTA015212	Gm16548	0.03651878	2.3569872	up
ASMM9PARTA015212	Gm16548	0.03651878	2.3569872	up
ASMM9PARTA017761	Gm5432	0.000127	3.140777	up
ASMM9PARTA050128	AK019745	0.001796412	2.8389366	up
humanlincRNA0220+_P1	humanlincRNA0220	0.001739725	2.381016	up
ASMM9PARTA016822	Gm14747	0.019921113	2.4263756	up
ASMM9PARTA015216	Abhd1	0.007528605	2.438796	up
ASMM9PARTA015216	Abhd1	0.007528605	2.438796	up
ASMM9PARTA010177	Gm6973	0.001154182	3.1507869	up
ASMM9PARTA047665	AK148313	0.002132029	2.1279442	up
ASMM9PARTA049540	AK076817	0.004377925	2.7078555	up
ASMM9PARTA017233	4933401P06Rik	0.013323069	2.0364592	up
ASMM9PARTA002192		0.000333	2.180026	up
ASMM9PARTA007514		0.021719767	2.2976751	up
ASMM9PARTA018410	D630014O11Rik	0.023319945	2.791825	up
ASMM9PARTA001246		0.018042874	3.5425942	up
ASMM9PARTA045375	E030003E18Rik	0.00000186	2.176617	up
CA876182_P1	mouselincRNA1216	0.001648179	3.0747747	up
ASMM9PARTA046716	mKIAA0696	0.04783114	2.027925	up
humanlincRNA2366+_P1	humanlincRNA2366	0.00000467	3.7282948	up
ASMM9PARTA003818		0.04744372	2.2373643	up
ASMM9PARTA050913	Zfp787	0.04403898	4.8922796	up
ASMM9PARTA014507	Gm15941	0.023029583	2.4174545	up
ASMM9PARTA014507	Gm15941	0.023029583	2.4174545	up
ASMM9PARTA014507	Gm15941	0.023029583	2.4174545	up
ASMM9PARTA014507	Gm15941	0.023029583	2.4174545	up
ASMM9PARTA051157	AK019792	0.023315659	2.347423	up
ASMM9PARTA005275		0.020657301	2.1455505	up
ASMM9PARTA045163	Asb7	0.02555406	2.444701	up
ASMM9PARTA045163	Asb7	0.02555406	2.444701	up
ASMM9PARTA045163	Asb7	0.02555406	2.444701	up
ASMM9PARTA046159	Ankrd11	0.003794145	2.1204615	up
ASMM9PARTA050279	AK049146	0.017445127	2.9101717	up
MM9LINCRNAEXON10684+_P1	mouselincRNA1112	0.019686526	2.287231	up
ASMM9PARTA046068	Dhx9	0.0000311	6.169505	up
ASMM9PARTA009351		0.007093248	2.2248828	up
MM9LINCRNAEXON11741+_P1		0.010595723	2.4476902	up
ASMM9PARTA046655	AK051928	0.016343374	2.2576847	up
ASMM9PARTA017687	4930522O17Rik	0.002738122	2.2901874	up
humanlincRNA1841+_P1	humanlincRNA1841	0.001601763	3.3601506	up
ASMM9PARTA007563		0.023464495	2.0654676	up
MM9LINCRNAEXON11156-_P1		0.000604	2.5789385	up
ASMM9PARTA015209	Gm13553	0.01703982	2.1824431	up
ASMM9PARTA007824		0.00862385	2.8821995	up
ASMM9PARTA007824		0.00862385	2.8821995	up

ASMM9PARTA007824		0.00862385	2.8821995	up
ASMM9PARTA007824		0.00862385	2.8821995	up
CUST_612_PI426073487	uc.133	0.002941481	2.6404295	up
ASMM9PARTA018864	AC132684.1	0.01411625	6.39298	up
ASMM9PARTA018864	AC132684.1	0.01411625	6.39298	up
ASMM9PARTA016256	E130215H24Rik	0.041712936	2.6932282	up
ASMM9PARTA046571	BC030469	0.009143457	2.0525916	up
ASMM9PARTA015217	AI838599	0.005204718	2.2516434	up
ASMM9PARTA049544	AK145161	0.003340717	2.1015143	up
ASMM9PARTA003824		0.01249576	2.9139717	up
ASMM9PARTA017541	A230087F16Rik	0.0000433	6.9646907	up
ASMM9PARTA003233		0.000275	2.5251708	up
ASMM9PARTA045185	Ralgps2	0.001484566	2.0597565	up
ASMM9PARTA045185	Ralgps2	0.001484566	2.0597565	up
ASMM9PARTA045185	Ralgps2	0.001484566	2.0597565	up
ASMM9PARTA045185	Ralgps2	0.001484566	2.0597565	up
ASMM9PARTA045185	Ralgps2	0.001484566	2.0597565	up
ASMM9PARTA045185	Ralgps2	0.001484566	2.0597565	up
ASMM9PARTA017183	1700086P04Rik	0.012110479	5.7909408	up
ASMM9PARTA047792	Cntn1	0.002157284	4.5487175	up
ASMM9PARTA047792	Cntn1	0.002157284	4.5487175	up
ASMM9PARTA019134	AC124756.1	0.00000321	2.1052601	up
ASMM9PARTA006006		0.005482468	3.2565968	up
ASMM9PARTA001671		0.000203	2.0235047	up
ASMM9PARTA017205	4930511M06Rik	0.009175573	2.41494	up
ASMM9PARTA007010		0.000042	2.2311423	up
ASMM9PARTA051308	AK038653	0.018635966	2.0771594	up
CUST_356_PI426073487	uc.358	0.034310702	2.4101782	up
ASMM9PARTA005120		0.001965555	2.521717	up
ASMM9PARTA047688	apo	0.03793647	2.0667164	up
CUST_196_PI426073487	uc.198	0.00177622	2.047007	up
ASMM9PARTA015795	3110053B16Rik	0.002151803	5.238408	up
ASMM9PARTA006603		0.013116387	3.620633	up
ASMM9PARTA050886	AK042904	0.015276106	3.445386	up
ASMM9PARTA050886	AK042904	0.015276106	3.445386	up
ASMM9PARTA007351		0.001895917	2.0549374	up
ASMM9PARTA007351		0.001895917	2.0549374	up
humanlincRNA1995+_P1	humanlincRNA1995	0.013025064	3.0825474	up
ASMM9PARTA015601	Gm13274	0.024606442	2.4788258	up
ASMM9PARTA015467	Gm15966	0.002925332	2.0849266	up
ASMM9PARTA007580		0.003245979	4.4922867	up
ASMM9PARTA004423		0.009521837	4.258889	up
ASMM9PARTA005347		0.033537924	2.7579272	up
ASMM9PARTA050385	AK080631	0.001908285	2.344752	up
MM9LINCRNAEXON10047+_P1	mouselincRNA1651	0.0000418	2.2476697	up
ASMM9PARTA001176		0.027657757	2.1731987	up
MM9LINCRNAEXON11616+_P1	mouselincRNA0430	0.020025373	2.6535373	up
ASMM9PARTA050165	AK020320	0.014991377	2.2686698	up
ASMM9PARTA050165	AK020320	0.014991377	2.2686698	up

ASMM9PARTA013917	Gm12798	0.006897513	2.0001042	up
ASMM9PARTA002410		0.030456366	2.031518	up
ASMM9PARTA011938	Hmgb1-ps1	0.00000959	2.5220013	up
MM9LINCRNAEXON11078+_P1	mouselincRNA0819	0.0000214	2.3410268	up
ASMM9PARTA012466	Gm4992	0.0441232	2.3853023	up
ASMM9PARTA049238	AK029171	0.019044857	6.588273	up
ASMM9PARTA016811	Gm12300	0.002102381	7.7662644	up
ASMM9PARTA016811	Gm12300	0.002102381	7.7662644	up
ASMM9PARTA016410	D230022J07Rik	0.028153684	2.0599937	up
ASMM9PARTA016410	D230022J07Rik	0.028153684	2.0599937	up
MM9LINCRNAEXON10212-_P1	mouselincRNA1479	0.0000616	2.1968613	up
AA791803_P1	mouselincRNA0980	0.001661601	3.8054485	up
MM9LINCRNAEXON11926+_P1	mouselincRNA0194	0.004795502	2.4484649	up
ASMM9PARTA013353	Gm15079	0.0000113	3.9250944	up
CUST_158_PI426409190		0.026826974	2.3801663	up
MM9LINCRNAEXON11627-_P1		0.002532851	2.3205512	up
MM9LINCRNAEXON10964-_P1		0.014502617	2.076026	up
ASMM9PARTA050126	AK019631	0.0000387	3.73109	up
humanlincRNA0164-_P1	humanlincRNA0164	0.0000945	2.7659109	up
ASMM9PARTA004138		0.001632887	2.006748	up
humanlincRNA1273-_P1	humanlincRNA1273	0.024615398	3.6337423	up
ASMM9PARTA002658		0.03918609	2.1415236	up
ASMM9PARTA004849		0.00000377	2.2213795	up
CK377992_P1	humanlincRNA1344	0.001810144	3.1504905	up
ASMM9PARTA002944		0.042605232	2.2926154	up
ASMM9PARTA001472		0.045620795	2.2508643	up
ASMM9PARTA000683		0.00000935	2.9192622	up
ASMM9PARTA009045		0.02410775	2.426114	up
ASMM9PARTA044906	2410002O22Rik	0.0000193	2.4992902	up
ASMM9PARTA044906	2410002O22Rik	0.0000193	2.4992902	up
ASMM9PARTA044906	2410002O22Rik	0.0000193	2.4992902	up
ASMM9PARTA044906	2410002O22Rik	0.0000193	2.4992902	up
MM9LINCRNAEXON10048-_P1		0.00575058	2.8061473	up
ASMM9PARTA011011	Gm13550	0.00648795	2.5241878	up
ASMM9PARTA002149		0.0000887	2.6577852	up
ASMM9PARTA004214		0.0000706	2.3478658	up
ASMM9PARTA010194	AI314831	0.016917923	2.1526694	up
ASMM9PARTA050991	Isg20l1	0.02310275	3.5411718	up
ASMM9PARTA050991	Isg20l1	0.02310275	3.5411718	up
CUST_365_PI426073487	uc.367	0.0000197	2.4493606	up
ASMM9PARTA014985	1500016L03Rik	0.000565	2.0157654	up
ASMM9PARTA045575	5830416P10Rik	0.001016318	2.147838	up
ASMM9PARTA016669	Gm16211	0.024743207	2.8097625	up
ASMM9PARTA009548	Gm8556	0.000162	2.6073902	up
ASMM9PARTA002598		0.008508823	2.2654462	up
ASMM9PARTA050472	AK080655	0.00000595	2.170994	up
ASMM9PARTA003936		0.000113	2.1957402	up
ASMM9PARTA010960	Gm14987	0.027119735	2.5412476	up
ASMM9PARTA018523	Airn	2.29E-08	33.75424	up

ASMM9PARTA005165		0.041299827	2.4284294	up
ASMM9PARTA017481	Gm13001	0.009409199	2.5680745	up
ASMM9PARTA004710		0.037228215	2.2064583	up
ASMM9PARTA015421	Gm15469	0.001661283	2.1588826	up
ASMM9PARTA009181		0.029672513	2.098135	up
ASMM9PARTA009181		0.029672513	2.098135	up
ASMM9PARTA009181		0.029672513	2.098135	up
ASMM9PARTA009181		0.029672513	2.098135	up
ASMM9PARTA009181		0.029672513	2.098135	up
CUST_421_PI426073487	uc.423	0.00062	2.4833558	up
ASMM9PARTA049260	AK015373	0.024819553	2.0319338	up
ASMM9PARTA045392	Airn	0.000155	2.9775214	up
ASMM9PARTA045392	Airn	0.000155	2.9775214	up
ASMM9PARTA017191	Gm15585	0.009732045	2.138788	up
ASMM9PARTA011140	Gm7199	0.02367411	3.3975704	up
MM9LINCRNAEXON10998-_P1	mouselincRNA0882	0.005738695	4.906354	up
ASMM9PARTA051664	Trip4	0.003507051	2.7870605	up
ASMM9PARTA051664	Trip4	0.003507051	2.7870605	up
ASMM9PARTA051664	Trip4	0.003507051	2.7870605	up
ASMM9PARTA013480	Gm13024	0.0000984	2.2979128	up
ASMM9PARTA019101	Gm13619	0.001322876	2.0551283	up
ASMM9PARTA019101	Gm13619	0.001322876	2.0551283	up
ASMM9PARTA019016	RP24-357O21.3	0.000312	2.2969394	up
ASMM9PARTA012429	Gm12587	0.00000916	2.0209234	up
ASMM9PARTA013144	Gm13081	0.012217936	2.1202137	up
mouselincRNA0436+_P1	mouselincRNA0436	0.014841333	3.0859246	up
ASMM9PARTA005904		0.027762242	2.3599787	up
ASMM9PARTA047035	AK149240	0.0000592	2.4694784	up
ASMM9PARTA018606	RP23-126F14.6	0.0000536	3.9785273	up
humanlincRNA1385-_P1	humanlincRNA1385	0.0000148	3.2987742	up
ASMM9PARTA000607		0.000748	2.084755	up
ASMM9PARTA000607		0.000748	2.084755	up
ASMM9PARTA051782	AK142198	0.000134	2.6232998	up
ASMM9PARTA051782	AK142198	0.000134	2.6232998	up
ASMM9PARTA051782	AK142198	0.000134	2.6232998	up
ASMM9PARTA051782	AK142198	0.000134	2.6232998	up
ASMM9PARTA051782	AK142198	0.000134	2.6232998	up
ASMM9PARTA008632		0.007343965	2.7453694	up
ASMM9PARTA006527		0.006562627	2.8661845	up
ASMM9PARTA049188	AK080416	0.000606	2.6531026	up
ASMM9PARTA012197	Gm12331	0.00000528	2.0659242	up
ASMM9PARTA014186	AV039307	0.0000105	2.5422335	up
ASMM9PARTA014186	AV039307	0.0000105	2.5422335	up
ASMM9PARTA014186	AV039307	0.0000105	2.5422335	up
ASMM9PARTA014186	AV039307	0.0000105	2.5422335	up
ASMM9PARTA015852	Gm13629	0.0000134	2.238708	up
ASMM9PARTA015852	Gm13629	0.0000134	2.238708	up
ASMM9PARTA009653	Airn	0.0000562	2.5570414	up
ASMM9PARTA004251		0.00076	2.1167417	up

ASMM9PARTA018993	RP23-122J17.10	0.034804277	2.7474499	up
ASMM9PARTA006719		0.000258	2.0034661	up
ASMM9PARTA047011	AK163447	0.013146942	2.2898543	up
ASMM9PARTA013866	4930455G09Rik	0.000221	3.3048015	up
ASMM9PARTA017858	Gm2639	0.019559832	2.7024176	up
ASMM9PARTA046645	IL-TIFb	0.00000338	17.057747	up
ASMM9PARTA014593	Gm15169	0.000156	4.1738105	up
ASMM9PARTA009944	Gm10075	0.0000562	2.1748219	up
humanlincRNA1016-_P1	humanlincRNA1016	0.003384101	2.128588	up
ASMM9PARTA010113	Hsp25-ps1	0.00000987	2.3264437	up
ASMM9PARTA049247	AK040741	0.030605968	2.3500462	up
ASMM9PARTA015658	4930517O19Rik	0.037017796	6.714502	up
MM9LINCRNAEXON10743-_P1	mouselincRNA1136	0.013886482	2.4767566	up
ASMM9PARTA007248		0.028717691	2.045148	up
ASMM9PARTA000933		0.032545682	2.2437923	up
ASMM9PARTA018302	Gm4475	0.000195	2.545118	up
AI550063_P1	mouselincRNA0690	0.000679	2.3616488	up
ASMM9PARTA013839	9330185C12Rik	0.026340034	2.731352	up
ASMM9PARTA002853		0.00000256	2.0878062	up
ASMM9PARTA002853		0.00000256	2.0878062	up
ASMM9PARTA002853		0.00000256	2.0878062	up
ASMM9PARTA011165	Gm13858	0.0037146	2.946392	up
ASMM9PARTA002573		0.00000063	3.1626308	up
ASMM9PARTA045380	4930412O13Rik	0.0000187	2.1888402	up
MM9LINCRNAEXON10474+_P1	mouselincRNA1297	0.004138014	5.0234838	up
ASMM9PARTA005229		0.000000177	11.208046	up
ASMM9PARTA048564	AK009785	0.041366693	3.2158453	up
ASMM9PARTA050840	AK050360	0.001704258	2.6885808	up
ASMM9PARTA011266	Gm12311	0.00017	2.0989513	up
ASMM9PARTA016011	Gm10396	0.003340071	3.9637914	up
ASMM9PARTA004792		0.001186573	2.8262455	up
ASMM9PARTA015228	Gm12576	0.032033097	2.9835026	up
ASMM9PARTA017884	B230206L02Rik	0.000035	2.4762156	up
ASMM9PARTA003730		0.003354916	2.3954244	up
ASMM9PARTA047058	Rtn-1A	0.001174494	2.5337267	up
ASMM9PARTA047058	Rtn-1A	0.001174494	2.5337267	up
humanlincRNA0587-_P1	humanlincRNA0587	0.04345736	2.438723	up
ASMM9PARTA009539	Foxl1	0.000535	2.0323555	up
MM9LINCRNAEXON11457+_P1	mouselincRNA0481	0.021859514	2.1053188	up
ASMM9PARTA005729		0.024246696	2.3730893	up
ASMM9PARTA012956	Gm11246	0.000000689	2.4768918	up
ASMM9PARTA011230	Gm14738	0.01458008	3.0252018	up
MM9LINCRNAEXON11100-_P1		0.000261	2.8333008	up
ASMM9PARTA019116	Gm16404	0.013188779	2.1679473	up
ASMM9PARTA045894	Gm2694	0.00000171	2.1997645	up
ASMM9PARTA019156	RP24-391B9.1	0.019646995	2.2116127	up
ASMM9PARTA050441	AK040808	0.021075083	2.0316467	up
ASMM9PARTA050441	AK040808	0.021075083	2.0316467	up
ASMM9PARTA017258	D830026I12Rik	0.034503005	3.0406442	up

humanlincRNA2046+_P1	humanlincRNA2046	0.03543032	2.6785324	up
ASMM9PARTA048888	Gnas	0.0000411	2.7940338	up
ASMM9PARTA048888	Gnas	0.0000411	2.7940338	up
ASMM9PARTA002287		0.038130965	2.4139993	up
ASMM9PARTA010091	Speer4c	0.028170228	3.2082398	up
ASMM9PARTA006843		0.001345258	2.399634	up
ASMM9PARTA003909		0.010097478	3.0227451	up
ASMM9PARTA048456	AK156750	0.025179787	2.1211462	up
ASMM9PARTA048456	AK156750	0.025179787	2.1211462	up
MM9LINCRNAEXON11202-_P1		0.000239	2.1383839	up
ASMM9PARTA051330	Ttc13	0.0000309	2.6243196	up
CUST_475_PI426073487	uc.477	0.001307104	2.459413	up
ASMM9PARTA047428	1700049E17Rik	0.000213	2.248075	up
ASMM9PARTA013513	Gm15556	0.001056074	2.274923	up
ASMM9PARTA013513	Gm15556	0.001056074	2.274923	up
ASMM9PARTA013513	Gm15556	0.001056074	2.274923	up
ASMM9PARTA017655	Gm14033	0.000000593	2.4470801	up
MM9LINCRNAEXON11392-_P1	mouselincRNA0603	0.001932479	2.1581032	up
ASMM9PARTA006188		0.0000404	3.1907637	up
ASMM9PARTA006188		0.0000404	3.1907637	up
mouselincRNA0344+_P1	mouselincRNA0344	0.009298035	2.2420862	up
ASMM9PARTA014556	Gm13723	0.006329532	2.246708	up
ASMM9PARTA009901	5830416P10Rik	0.00000156	2.3955135	up
ASMM9PARTA008907		0.03937839	15.776631	up
MM9LINCRNAEXON10522+_P1	mouselincRNA1351	0.03050216	2.6011755	up
MM9LINCRNAEXON11785-_P1		0.001973419	3.510876	up
ASMM9PARTA046930	AK018772	0.0000305	4.4427795	up
ASMM9PARTA007292		0.025005484	2.4554706	up
ASMM9PARTA007292		0.025005484	2.4554706	up
ASMM9PARTA007292		0.025005484	2.4554706	up
ASMM9PARTA012893	Hmgb1-ps5	0.001829744	2.1575558	up
ASMM9PARTA050302	mKIAA1386	0.012203548	2.085032	up
ASMM9PARTA007306		0.024012117	2.2535694	up
ASMM9PARTA010688	Gm15059	0.0000383	2.3767042	up
ASMM9PARTA046432	AK048993	0.023261486	2.132466	up
mouselincRNA1667-_P1	mouselincRNA1667	0.002626843	2.184947	up
ASMM9PARTA046016	Olfr856-ps1	0.005357082	4.7804413	up
ASMM9PARTA002453		0.045923255	2.0737858	up
ASMM9PARTA018017	Gm16169	0.006929116	3.9912865	up
ASMM9PARTA003722		0.00039	2.1613472	up
ASMM9PARTA006841		0.03256857	2.2611759	up
ASMM9PARTA049457	AK138660	0.0000686	3.2273483	up
ASMM9PARTA047303	Heatr1	0.045637984	2.3177323	up
ASMM9PARTA047303	Heatr1	0.045637984	2.3177323	up
ASMM9PARTA047303	Heatr1	0.045637984	2.3177323	up
ASMM9PARTA007125		0.000000953	3.1511805	up
ASMM9PARTA045563	Airn	0.000227	2.1253428	up
ASMM9PARTA003603		0.000353	2.7487905	up
ASMM9PARTA003671		0.03990971	2.5798256	up

ASMM9PARTA045571	Rmst	0.0000641	2.0183094	up
ASMM9PARTA046136	AK039635	0.001451956	2.1305637	up
ASMM9PARTA046136	AK039635	0.001451956	2.1305637	up
ASMM9PARTA014537	1500016L03Rik	0.0000434	5.442869	up
AV486351_P1	humanlincRNA0937	0.030061444	2.528093	up
MM9LINCRNAEXON10071+_P1	mouselincRNA1506	0.0000148	3.4166107	up
ASMM9PARTA018262	Ear-ps10	0.000686	2.7500083	up
BC156060_P1	mouselincRNA1038	0.0000679	18.584675	up
BC156060_P1	mouselincRNA1038	0.0000679	18.584675	up
BC156060_P1	mouselincRNA1038	0.0000679	18.584675	up
BC156060_P1	mouselincRNA1038	0.0000679	18.584675	up
BC156060_P1	mouselincRNA1038	0.0000679	18.584675	up
ASMM9PARTA004884		0.000000411	2.192306	up
ASMM9PARTA012278	Gm15794	0.016605083	2.245738	up
ASMM9PARTA003161		0.0000969	2.4660194	up
ASMM9PARTA004616		0.012928735	2.2643428	up
ASMM9PARTA014167	Gm13944	0.0000129	17.70349	up
ASMM9PARTA014167	Gm13944	0.0000129	17.70349	up
ASMM9PARTA014167	Gm13944	0.0000129	17.70349	up
ASMM9PARTA047833	AK142028	0.00701822	2.3403025	up
ASMM9PARTA047833	AK142028	0.00701822	2.3403025	up
ASMM9PARTA045545	AI427809	0.004380761	2.6451612	up
humanlincRNA1193-_P1	humanlincRNA1193	0.00000384	3.5577245	up
ASMM9PARTA006594		0.002527592	2.2236533	up
ASMM9PARTA002496		0.005256313	2.9569044	up
ASMM9PARTA002496		0.005256313	2.9569044	up
MM9LINCRNAEXON12014+_P1	mouselincRNA0024	0.005953616	2.0437932	up
ASMM9PARTA018122	Gm6413	0.038960703	2.9755666	up
ASMM9PARTA049015	AK052812	0.001652858	3.9565578	up
ASMM9PARTA048635	2810051F02Rik	0.000329	2.3189695	up
ASMM9PARTA049815	AK042933	0.00000931	3.8712125	up
ASMM9PARTA011317	Hmgb1-ps3	0.00000566	2.1366735	up
ASMM9PARTA051121	Lrrc49	0.0000888	8.12811	up
ASMM9PARTA051121	Lrrc49	0.0000888	8.12811	up
ASMM9PARTA051121	Lrrc49	0.0000888	8.12811	up
ASMM9PARTA049391	Ptpn22	0.005117974	2.18758	up
ASMM9PARTA049809	AK030206	0.00000105	2.8406954	up
ASMM9PARTA049809	AK030206	0.00000105	2.8406954	up
ASMM9PARTA016008	Gm12159	0.003832694	2.296931	up
ASMM9PARTA050695	AK035901	0.000138	2.2840664	up
ASMM9PARTA015540	Gm14271	0.016793156	2.6326547	up
ASMM9PARTA050412	AK006664	0.000278	2.7338235	up
ASMM9PARTA002084		0.00000329	2.6720037	up
MM9LINCRNAEXON11381+_P1	mouselincRNA0588	0.006293111	2.622957	up
ASMM9PARTA002399		0.008305885	2.9394252	up
ASMM9PARTA014232	Gm15169	0.002152088	2.8948379	up
ASMM9PARTA003264		0.028498638	2.1745765	up
ASMM9PARTA007558		0.00000693	3.2392747	up
ASMM9PARTA003147		0.0000461	5.46201	up

CUST_375_PI426073487	uc.377	0.014030896	4.6396375	up
ASMM9PARTA050230	AK131834	0.000225	28.298044	up
CUST_428_PI426073487	uc.430	0.0000202	3.0572083	up
CUST_428_PI426073487	uc.430	0.0000202	3.0572083	up
CUST_428_PI426073487	uc.430	0.0000202	3.0572083	up
CUST_428_PI426073487	uc.430	0.0000202	3.0572083	up
CUST_428_PI426073487	uc.430	0.0000202	3.0572083	up
ASMM9PARTA006828		0.0000243	2.2162519	up
ASMM9PARTA006973		0.001523258	2.5381515	up
ASMM9PARTA006973		0.001523258	2.5381515	up
ASMM9PARTA049559	BC051212	0.004809617	2.3670506	up
CUST_887_PI426073487	uc.408	0.000111	2.1381514	up
ASMM9PARTA007283		0.000206	7.3439097	up
ASMM9PARTA015431	Gm15918	0.000055	2.0622482	up
ASMM9PARTA019374	4930412M03Rik	0.008444748	2.0580752	up
ASMM9PARTA019550	RP23-78F6.7	0.000129	2.3570538	up
MM9LINCRNAEXON12034-_P1	mouselincRNA0038	0.006064827	2.0999901	up
ASMM9PARTA003916		0.00000393	3.192659	up
MM9LINCRNAEXON10204-_P1	mouselincRNA1471	0.000257	2.4786034	up
ASMM9PARTA005240		0.000559	2.7824998	up
ASMM9PARTA048669	AK165766	0.000584	2.213304	up
ASMM9PARTA048669	AK165766	0.000584	2.213304	up
ASMM9PARTA048669	AK165766	0.000584	2.213304	up
mouselincRNA1511+_P1	mouselincRNA1511	0.024254475	2.6243196	up
ASMM9PARTA011219	Gm8812	0.000988	2.7603712	up
ASMM9PARTA001853		0.000969	2.3442802	up
ASMM9PARTA047938	AK082117	0.000005	2.5264413	up
ASMM9PARTA047221	AK040557	0.0000218	3.353819	up
ASMM9PARTA047221	AK040557	0.0000218	3.353819	up
ASMM9PARTA047221	AK040557	0.0000218	3.353819	up
ASMM9PARTA019295	RP23-133I8.1	0.0000299	2.1335657	up
ASMM9PARTA047783	Ugcgl2	0.00000437	3.8473175	up
ASMM9PARTA003032		0.008523298	3.0427992	up
ASMM9PARTA003032		0.008523298	3.0427992	up
ASMM9PARTA003032		0.008523298	3.0427992	up
ASMM9PARTA003716		0.0000245	2.1912527	up
ASMM9PARTA002372		0.001381885	2.600094	up
ASMM9PARTA012055	Gm12118	0.000126	2.197365	up
AA915284_P1	mouselincRNA0740	0.007956133	3.8496263	up
ASMM9PARTA013691	Gm14162	0.000344	2.1395185	up
ASMM9PARTA000845		0.00083	2.6613104	up
ASMM9PARTA018548	Airn	0.00000196	3.6642368	up
ASMM9PARTA018548	Airn	0.00000196	3.6642368	up
ASMM9PARTA014091	4930419G24Rik	0.047696058	3.3682609	up
ASMM9PARTA014091	4930419G24Rik	0.047696058	3.3682609	up
ASMM9PARTA046327	AK009351	0.0000433	3.1334538	up
ASMM9PARTA010097	Gm10361	0.000382	2.2060933	up
ASMM9PARTA000537		0.00000117	5.2932167	up
ASMM9PARTA015283	Gm16677	0.0000674	2.0244634	up

ASMM9PARTA014139	Gm3160	0.00000624	2.0727074	up
ASMM9PARTA014139	Gm3160	0.00000624	2.0727074	up
ASMM9PARTA047832	AK052076	0.008204426	2.5495343	up
ASMM9PARTA015674	Gm13112	0.0000242	4.0338807	up
ASMM9PARTA015674	Gm13112	0.0000242	4.0338807	up
ASMM9PARTA013565	Gm14473	0.002184913	2.1388512	up
ASMM9PARTA003572		0.000103	3.4023733	up
ASMM9PARTA049446	AK031919	0.001955584	2.2646444	up
ASMM9PARTA000684		0.000000751	3.8835692	up
ASMM9PARTA013124	Gm4996	0.0000216	2.2034204	up
ASMM9PARTA046430	AK153590	0.029219106	2.4526875	up
ASMM9PARTA013453	Gm16471	0.000728	2.7894201	up
ASMM9PARTA015970	A730036I17Rik	0.024104962	2.2069778	up
ASMM9PARTA049072	Rasgrp2	0.000000213	2.1650708	up
ASMM9PARTA047252	AK149260	0.00081	2.0493648	up
ASMM9PARTA012045	Gm14788	0.0000751	2.4017513	up
ASMM9PARTA000876		0.049603302	2.062359	up
ASMM9PARTA000876		0.049603302	2.062359	up
ASMM9PARTA000876		0.049603302	2.062359	up
ASMM9PARTA012955	Gm13932	0.000185	2.1489203	up
CUST_499_PI426073487	uc.19	0.008816327	2.7671895	up
ASMM9PARTA045371	5033406O09Rik	0.000309	12.8328495	up
ASMM9PARTA002899		0.0000121	2.5946267	up
ASMM9PARTA002899		0.0000121	2.5946267	up
ASMM9PARTA002899		0.0000121	2.5946267	up
ASMM9PARTA049183	AK036371	0.002618839	2.3823323	up
ASMM9PARTA002490		0.0000806	2.655869	up
ASMM9PARTA046929	AK010044	0.038038343	22.237125	up
ASMM9PARTA051072	AK019737	0.003148946	2.5659835	up
CUST_846_PI426073487	uc.367	0.01564255	2.6767406	up
ASMM9PARTA001331		0.009780455	3.2017717	up
ASMM9PARTA004510		0.038085256	2.077547	up
ASMM9PARTA013646	D230017M19Rik	0.032388743	5.0653653	up
ASMM9PARTA048743	Ppp1r1c	0.000718	2.557851	up
ASMM9PARTA006591		0.019842729	3.4523509	up
ASMM9PARTA048585	C230097I24Rik	0.000818	2.07183	up
MM9LINCRNAEXON11899+_P1	mouselincRNA0173	0.016981503	2.8582718	up
MM9LINCRNAEXON11700+_P1	mouselincRNA0335	0.005123737	2.6914	up
CJ317124_P1	humanlincRNA1977	0.0000198	7.333081	up
MM9LINCRNAEXON12113-_P1		0.0000059	2.6684735	up
ASMM9PARTA019604	Gm14598	0.000246	3.4617174	up
ASMM9PARTA010437	Cd47	0.0000212	4.32716	up
ASMM9PARTA045591	Gm11110	0.036867116	3.6468546	up
ASMM9PARTA007871		0.0000703	2.0995383	up
ASMM9PARTA002148		0.0000115	2.0013807	up
ASMM9PARTA016965	Gm16119	0.03382048	2.3199215	up
ASMM9PARTA004077		0.0000106	2.6248574	up
ASMM9PARTA002181		0.000333	2.3494198	up
ASMM9PARTA008988		0.0000971	2.025747	up

EL605756_P1	humanlincRNA0451	0.000056	3.613005	up
ASMM9PARTA003760		0.00000141	2.4757895	up
ASMM9PARTA016760	Gm2694	0.00019	5.326694	up
ASMM9PARTA045310	Gm11961	0.04806668	2.1525261	up
ASMM9PARTA048972	AK086826	0.0000198	2.6653178	up
ASMM9PARTA049783	Rps6ka1	0.0000646	2.4058597	up
ASMM9PARTA006611		0.013833252	2.5984473	up
ASMM9PARTA000581		0.0000377	2.1457024	up
MM9LINCRNAEXON10767+_P1	mouselincRNA1138	0.000154	2.4317145	up
AV038163_P1	humanlincRNA2267	0.0000274	2.2915192	up
ASMM9PARTA004878		0.018232044	2.2113686	up
ASMM9PARTA003919		0.00000216	2.2708077	up
MM9LINCRNAEXON10396-_P1	mouselincRNA1257	0.0000141	3.2808087	up
ASMM9PARTA006908		0.008430216	4.381537	up
ASMM9PARTA009169		0.0000247	2.889862	up
ASMM9PARTA007450		0.000134	2.3509471	up
ASMM9PARTA019271	AL928696.1	0.005473444	2.1196356	up
ASMM9PARTA008305		0.04622204	2.0878878	up
ASMM9PARTA046796	BC030470	0.0000127	2.2068095	up
ASMM9PARTA018396	Gm16404	0.012278657	2.3413486	up
MM9LINCRNAEXON11795+_P1	mouselincRNA0252	0.0000617	18.041677	up
MM9LINCRNAEXON10103+_P1	mouselincRNA1524	0.0003	5.9844313	up
ASMM9PARTA015949	5330413P13Rik	0.018231718	2.1202192	up
ASMM9PARTA004712		0.003130417	3.137045	up
MM9LINCRNAEXON10539-_P1		0.000000669	9.163587	up
ASMM9PARTA011271	Gm13228	0.000255	2.3399453	up
ASMM9PARTA018144	Gm15051	0.0000882	2.5616744	up
ASMM9PARTA011414	Gm15778	0.0000194	2.1751525	up
ASMM9PARTA003826		0.0000314	5.6208506	up
ASMM9PARTA007146		0.003945055	2.0435443	up
ASMM9PARTA002335		0.000642	2.0657623	up
ASMM9PARTA001043		0.00013	2.2876415	up
MM9LINCRNAEXON11872-_P1	mouselincRNA0141	0.04465102	2.0433455	up
ASMM9PARTA019208	Gm14595	0.043605294	2.922996	up
ASMM9PARTA001365		0.0000174	2.3903117	up
ASMM9PARTA000070		0.0421954	4.916226	up
ASMM9PARTA000070		0.0421954	4.916226	up
ASMM9PARTA012898	Gm5396	0.0000202	2.09585	up
ASMM9PARTA001969		0.000178	5.015228	up
ASMM9PARTA018095	Gm16990	0.000293	3.7959373	up
MM9LINCRNAEXON10492+_P1	mouselincRNA1320	0.010376689	2.7581644	up
ASMM9PARTA013698	Gm13366	0.00309633	2.2090125	up
ASMM9PARTA002671		0.000461	2.0028193	up
ASMM9PARTA014788	B930095G15Rik	5.58E-08	4.029823	up
ASMM9PARTA050069	BC038278	0.03933176	2.8830078	up
ASMM9PARTA048236	AK182695	0.018807136	2.1764898	up
ASMM9PARTA002260		0.000305	3.2376847	up
ASMM9PARTA002260		0.000305	3.2376847	up
ASMM9PARTA004889		0.045090396	2.244647	up

ASMM9PARTA019278	Gm5226	0.0000159	2.3915865	up
ASMM9PARTA017528	Gm13643	4.92E-08	6.467713	up
ASMM9PARTA010788	Gm12261	0.0000106	2.017353	up
ASMM9PARTA014333	1500016L03Rik	0.001133286	6.2949452	up
ASMM9PARTA046928	AK079404	0.008261258	8.877083	up
ASMM9PARTA012597	Gm7958	0.045609962	2.0498455	up
MM9LINCRNAEXON10221-_P1	mouselincRNA1483	0.025543272	2.5155814	up
ASMM9PARTA007101		0.001445409	2.1238625	up
ASMM9PARTA016836	Hoxb3os	0.000883	2.3799367	up
ASMM9PARTA016836	Hoxb3os	0.000883	2.3799367	up
ASMM9PARTA016836	Hoxb3os	0.000883	2.3799367	up
ASMM9PARTA044894	C330024D21Rik	0.0000149	4.848213	up
ASMM9PARTA050456	A530053G22Rik	0.000169	2.6750376	up
ASMM9PARTA014069	A730017L22Rik	0.000206	2.3494983	up
MM9LINCRNAEXON12123+_P1	mouselincRNA0129	0.000291	4.3230715	up
ASMM9PARTA004567		0.0000481	2.9336364	up
CUST_662_PI426073487	uc.183	0.00000438	2.3641312	up
ASMM9PARTA011990	Lamr1-ps1	0.00000736	14.883051	up
ASMM9PARTA015799	5530601H04Rik	0.004917894	2.8705761	up
ASMM9PARTA008317		0.039608467	0.313883934	down
ASMM9PARTA010051	Gm10497	0.000034	0.399063366	down
ASMM9PARTA010051	Gm10497	0.000034	0.399063366	down
ASMM9PARTA010051	Gm10497	0.000034	0.399063366	down
ASMM9PARTA007800		0.0000239	0.101912313	down
ASMM9PARTA001658		0.0000302	0.39969155	down
ASMM9PARTA050620	AK042016	0.000569	0.43446526	down
ASMM9PARTA009163		0.005812168	0.484695803	down
ASMM9PARTA003870		0.016687071	0.486223962	down
ASMM9PARTA009086		0.000203	0.322766163	down
ASMM9PARTA003557		0.000811	0.457844956	down
ASMM9PARTA007767		0.000575	0.3086745	down
ASMM9PARTA050451	TCR-beta chain	0.00000429	0.292112979	down
ASMM9PARTA015180	A530058N18Rik	0.000286	0.124095314	down
ASMM9PARTA047362	BC064451	0.000297	0.335039994	down
ASMM9PARTA003503		0.00000678	0.478635506	down
ASMM9PARTA001783		0.000192	0.251844535	down
ASMM9PARTA049723	AK017111	0.000589	0.281338745	down
ASMM9PARTA051617	AK039014	0.025748411	0.39605576	down
ASMM9PARTA014191	Gm12092	0.009861143	0.450306819	down
CUST_195_PI426073487	uc.197	0.000323	0.435374605	down
ASMM9PARTA050522	TCR-beta chain	0.004011236	0.370055001	down
ASMM9PARTA000135		0.008829746	0.33962861	down
ASMM9PARTA002902		0.024691721	0.213336883	down
ASMM9PARTA045333	3110099E03Rik	0.002467179	0.226432662	down
ASMM9PARTA005664		0.001665762	0.495524301	down
ASMM9PARTA047033	AK043365	0.048594628	0.223587222	down
ASMM9PARTA051451	AK032255	0.011844053	0.413422455	down
ASMM9PARTA018431	RP24-397H7.1	0.001208038	0.205125777	down
ASMM9PARTA050079	11-Sep	0.00000174	0.43810777	down

ASMM9PARTA046323	Ccdc138	0.041328423	0.490821346	down
ASMM9PARTA047203	AK135975	0.0002	0.348169412	down
ASMM9PARTA046371	C130026I21Rik	0.001128692	0.410246346	down
ASMM9PARTA046371	C130026I21Rik	0.001128692	0.410246346	down
ASMM9PARTA004452		0.000032	0.19990009	down
ASMM9PARTA045260	5330426P16Rik	0.000101	0.196487727	down
ASMM9PARTA008971		0.000263	0.289310012	down
ASMM9PARTA011381	Mup-ps14	0.000106	0.205733632	down
ASMM9PARTA050925	AK046300	0.000783	0.444389577	down
ASMM9PARTA050700	TCR-beta chain	0.0000158	0.203070194	down
ASMM9PARTA006630		0.046415683	0.33069869	down
ASMM9PARTA045862	1700086L19Rik	0.0000156	0.343948442	down
ASMM9PARTA019893		0.005497582	0.240715866	down
ASMM9PARTA000020		0.009681764	0.190124261	down
ASMM9PARTA010105	Ear-ps2	0.0000788	0.499098678	down
ASMM9PARTA004261		0.0000512	0.433746426	down
ASMM9PARTA045860	A630072M18Rik	0.039366838	0.426909288	down
ASMM9PARTA046300	AK046727	0.00000411	0.231601142	down
ASMM9PARTA015504	Gm14455	0.000229	0.42260756	down
ASMM9PARTA015504	Gm14455	0.000229	0.42260756	down
ASMM9PARTA015504	Gm14455	0.000229	0.42260756	down
ASMM9PARTA046870	Ppp1r1b	0.00000167	0.229699117	down
ASMM9PARTA000355		0.012110778	0.364568519	down
ASMM9PARTA000355		0.012110778	0.364568519	down
ASMM9PARTA000355		0.012110778	0.364568519	down
ASMM9PARTA051179	AK162599	0.000109	0.079461537	down
ASMM9PARTA001669		0.0000184	0.1581305	down
CUST_171_PI426409190		0.001080269	0.314804705	down
ASMM9PARTA007331		0.003805738	0.456118316	down
ASMM9PARTA014321	Gm11767	0.000545	0.427068124	down
ASMM9PARTA014321	Gm11767	0.000545	0.427068124	down
ASMM9PARTA014321	Gm11767	0.000545	0.427068124	down
ASMM9PARTA047728	AK043659	0.001091563	0.173403731	down
ASMM9PARTA014132	4930500J02Rik	0.000116	0.265754456	down
ASMM9PARTA012474	Gm13754	0.005948891	0.464414732	down
ASMM9PARTA003854		0.000709	0.444267113	down
ASMM9PARTA051038	BC065393	0.000518	0.360109335	down
ASMM9PARTA051038	BC065393	0.000518	0.360109335	down
ASMM9PARTA051038	BC065393	0.000518	0.360109335	down
ASMM9PARTA007216		0.0000121	0.470568748	down
ASMM9PARTA007216		0.0000121	0.470568748	down
ASMM9PARTA047990	AK013627	0.0000104	0.089159903	down
ASMM9PARTA003438		0.0000779	0.433145882	down
ASMM9PARTA000308		0.000934	0.163460223	down
ASMM9PARTA019551		0.013549506	0.243677453	down
ASMM9PARTA007701		0.0000449	0.47207359	down
ASMM9PARTA002484		0.00000037	0.238423621	down
ASMM9PARTA048535	AK122507	0.0000192	0.30549765	down
ASMM9PARTA004828		0.015953597	0.490167846	down

ASMM9PARTA014309	D330050G23Rik	0.014582017	0.314954281	down
ASMM9PARTA018479	Gm16339	0.000073	0.311350659	down
MM9LINCRNAEXON11015-_P1		0.0000429	0.174036673	down
ASMM9PARTA014587	1110050K14Rik	0.024782818	0.382123557	down
ASMM9PARTA000477		0.004741762	0.306028979	down
ASMM9PARTA001867		0.004047871	0.461227275	down
ASMM9PARTA004491		0.0000243	0.156446357	down
ASMM9PARTA019195	Mup-ps4	0.0000187	0.252131711	down
ASMM9PARTA017897	7SK.148	0.000931	0.234949505	down
ASMM9PARTA051087	AK039837	0.012122608	0.328606126	down
ASMM9PARTA017692	Gm7819	0.0000152	0.308506623	down
ASMM9PARTA050523	TCR-beta chain	0.00000233	0.240691034	down
ASMM9PARTA047164	AK144886	0.012889706	0.38436079	down
ASMM9PARTA050701	TCR-beta chain	0.00000773	0.236070205	down
ASMM9PARTA003326		0.007560213	0.452716316	down
ASMM9PARTA050923	AK140288	0.001046875	0.41941541	down
ASMM9PARTA050923	AK140288	0.001046875	0.41941541	down
ASMM9PARTA003714		0.0000738	0.108725114	down
ASMM9PARTA002564		0.000359	0.32874612	down
ASMM9PARTA007801		0.000176	0.142176018	down
ASMM9PARTA011779	Kif22-ps	0.000934	0.492741062	down
ASMM9PARTA048778	AK131873	0.0000588	0.228500137	down
ASMM9PARTA007449		0.002682841	0.304663391	down
ASMM9PARTA047383	AK045624	0.008474385	0.277203351	down
ASMM9PARTA010226	Gm5859	0.001027677	0.380875234	down
ASMM9PARTA006132		0.003083901	0.398229472	down
ASMM9PARTA006132		0.003083901	0.398229472	down
ASMM9PARTA006132		0.003083901	0.398229472	down
ASMM9PARTA048083	AK045744	0.000626	0.327439509	down
MM9LINCRNAEXON11857-_P1	mouselincRNA0281	0.002778734	0.277427133	down
ASMM9PARTA002424		0.002141766	0.457013243	down
ASMM9PARTA049898	AK044041	0.0000226	0.216522109	down
ASMM9PARTA009763	Gm10599	0.000046	0.393542023	down
ASMM9PARTA001917		0.000534	0.368623097	down
ASMM9PARTA002412		0.0000748	0.486951264	down
ASMM9PARTA012178	Mup-ps13	0.000185	0.266223997	down
ASMM9PARTA015630	Dlx1as	0.0000273	0.389636207	down
ASMM9PARTA004156		0.000189	0.467837789	down
ASMM9PARTA001455		0.007929599	0.467930763	down
ASMM9PARTA045969	AI507597	0.002052181	0.478637407	down
ASMM9PARTA003896		0.000334	0.405885552	down
ASMM9PARTA006529		0.000161	0.181765846	down
ASMM9PARTA006529		0.000161	0.181765846	down
ASMM9PARTA000938		0.01583118	0.483355134	down
ASMM9PARTA009931	Gm5096	0.016972536	0.356938688	down
ASMM9PARTA005519		2.04E-09	0.022539075	down
ASMM9PARTA009963	Gm10282	0.002013233	0.443035375	down
ASMM9PARTA018833	Gm16315	0.0000574	0.429691604	down
ASMM9PARTA018833	Gm16315	0.0000574	0.429691604	down

MM9LINCRNAEXON10535-_P1		0.004745931	0.295375826	down
ASMM9PARTA011789	Gm12856	0.00126236	0.278329543	down
ASMM9PARTA049348	AK138412	0.000176	0.430449387	down
ASMM9PARTA009220		0.0000316	0.217465101	down
ASMM9PARTA006695		3.81E-09	0.034571801	down
ASMM9PARTA002356		0.0000479	0.408523867	down
ASMM9PARTA002196		0.00000147	0.133610882	down
ASMM9PARTA002360		0.000108	0.469904165	down
ASMM9PARTA047513	AK044426	0.002303171	0.269723446	down
MM9LINCRNAEXON11962-_P1		0.000193	0.452682706	down
ASMM9PARTA019104	AC104908.1	0.010129376	0.381206955	down
ASMM9PARTA046259	Gm4814	0.001954298	0.392278084	down
ASMM9PARTA013927	Gm16002	0.000132	0.223707571	down
ASMM9PARTA017060	G730013B05Rik	0.010660555	0.436872359	down
ASMM9PARTA047761	Fastkd3	0.000155	0.45856734	down
ASMM9PARTA047761	Fastkd3	0.000155	0.45856734	down
ASMM9PARTA016945	A230056P14Rik	0.000000214	0.047408037	down
ASMM9PARTA005168		0.0000346	0.427127153	down
ASMM9PARTA049309	AK049102	0.0000813	0.461088255	down
ASMM9PARTA012912	Mup-ps22	0.000000457	0.212015872	down
ASMM9PARTA008228		0.006670833	0.474443395	down
ASMM9PARTA008228		0.006670833	0.474443395	down
AK039249_P1	humanlincRNA1989	0.0000781	0.363585171	down
ASMM9PARTA047251	AK086245	0.000101	0.499246487	down
ASMM9PARTA011898	Mup-ps16	0.001047184	0.463997549	down
ASMM9PARTA012332	Mup-ps21	0.001055858	0.11001249	down
ASMM9PARTA045941	D830015G02Rik	0.000000201	0.031819172	down
ASMM9PARTA050591	TCRB	0.00000581	0.310454801	down
ASMM9PARTA003521		0.0000421	0.458899514	down
ASMM9PARTA046086		0.001892755	0.135877055	down
ASMM9PARTA050423	TCR-beta chain	0.000127	0.224978484	down
CUST_840_PI426073487	uc.361	0.002104641	0.358530203	down
ASMM9PARTA000311		0.009939475	0.240465796	down
ASMM9PARTA008547		0.00745637	0.341563068	down
ASMM9PARTA046121		0.015893096	0.481733578	down
ASMM9PARTA050353	Tcrb	0.000233	0.212933677	down
ASMM9PARTA045146	3110070M22Rik	0.001705321	0.358304275	down
ASMM9PARTA045146	3110070M22Rik	0.001705321	0.358304275	down
ASMM9PARTA050521	TCRBVbeta5.1/Jbeta1.5	0.00000341	0.203821074	down
ASMM9PARTA000460		0.000126	0.117057797	down
ASMM9PARTA017466	Dleu2	0.000066	0.405998383	down
ASMM9PARTA003733		0.000304	0.48456416	down
ASMM9PARTA003470		0.002803225	0.493738556	down
ASMM9PARTA012180	Mup-ps17	0.00000365	0.189395812	down
humanlincRNA0848-_P1	humanlincRNA0848	0.014566735	0.463661632	down
ASMM9PARTA045493	D6Ertd474e	0.0000173	0.381655645	down
MM9LINCRNAEXON11287-_P1	mouselincRNA0638	0.0000362	0.194903218	down
ASMM9PARTA009368	Rgsl1	0.000181	0.469703866	down
ASMM9PARTA016956	Mup-ps4	0.000898	0.416643439	down

ASMM9PARTA012907	Gm11299	0.025686609	0.399218267	down
ASMM9PARTA003511		0.00000198	0.393958256	down
ASMM9PARTA009940	Spata5l1	0.000119	0.320462553	down
ASMM9PARTA003754		0.000488	0.177627983	down
ASMM9PARTA002025		0.016783688	0.291783312	down
ASMM9PARTA019683		0.002348356	0.141835027	down
ASMM9PARTA001520		0.0000337	0.22681199	down
ASMM9PARTA009478	Gm5973	0.002758365	0.44622082	down
ASMM9PARTA009478	Gm5973	0.002758365	0.44622082	down
ASMM9PARTA016118	Gm13974	0.000198	0.203421757	down
CUST_212_PI426073487	uc.214	0.01042787	0.293470183	down
ASMM9PARTA017367	Gm15511	0.011003451	0.498328755	down
MM9LINCRNAEXON12015-_P1	mouselincRNA0025	0.00000337	0.142997771	down
ASMM9PARTA007538		0.0001	0.386153121	down
ASMM9PARTA003971		0.000124	0.381781931	down
CJ133294_P1	mouselincRNA0439	0.001212857	0.433873625	down
ASMM9PARTA009322		0.0000873	0.354362026	down
ASMM9PARTA046085		0.024496904	0.240702679	down
ASMM9PARTA044983	Dlx1as	0.00000323	0.413622064	down
ASMM9PARTA009159		0.007852704	0.441887975	down
ASMM9PARTA018023	Gm15997	0.000385	0.210821888	down
ASMM9PARTA001480		0.005839385	0.33914706	down
ASMM9PARTA047897	etoile	0.0000835	0.405742671	down
MM9LINCRNAEXON11045-_P1	mouselincRNA0927	0.001219755	0.482678456	down
ASMM9PARTA007901		0.002469087	0.456291808	down
ASMM9PARTA013751	Mup-ps8	0.001575466	0.368122058	down
MM9LINCRNAEXON10992+_P1	mouselincRNA0879	0.000561	0.373536075	down
ASMM9PARTA011619	Gm14282	0.004663687	0.22783872	down
MM9LINCRNAEXON10572-_P1		0.037459962	0.471489012	down
ASMM9PARTA001264		0.001242206	0.094483612	down
ASMM9PARTA014404	A530058N18Rik	0.000548	0.379554579	down
ASMM9PARTA016341	D030055H07Rik	0.00015	0.293597858	down
ASMM9PARTA016341	D030055H07Rik	0.00015	0.293597858	down
ASMM9PARTA001079		0.006855164	0.459507403	down
ASMM9PARTA002178		0.00000763	0.324288547	down
ASMM9PARTA009305	9930014A18Rik	0.000191	0.43897424	down
ASMM9PARTA045316	4930429B21Rik	0.00000232	0.455881498	down
ASMM9PARTA051561		0.000000799	0.055206192	down
ASMM9PARTA050490	H4	0.000392	0.209170123	down
ASMM9PARTA006592		0.00000867	0.119514172	down
ASMM9PARTA046131	AK046737	0.00000401	0.162668873	down
ASMM9PARTA049218	AK018352	0.0000203	0.48610973	down
ASMM9PARTA049218	AK018352	0.0000203	0.48610973	down
ASMM9PARTA049218	AK018352	0.0000203	0.48610973	down
ASMM9PARTA049218	AK018352	0.0000203	0.48610973	down
ASMM9PARTA049218	AK018352	0.0000203	0.48610973	down
ASMM9PARTA009055		0.00000132	0.213683606	down
ASMM9PARTA002587		0.000038	0.33936088	down
ASMM9PARTA013542	4933400F03Rik	0.000042	0.453508487	down

ASMM9PARTA009176		0.014216131	0.16627155	down
ASMM9PARTA018136	7SK.187	0.011725743	0.485966786	down
ASMM9PARTA000706		0.0000612	0.463444215	down
ASMM9PARTA012440	Mup-ps9	0.001245018	0.1314179	down
ASMM9PARTA045928	1700086L19Rik	0.000212	0.496398184	down
ASMM9PARTA014891	1500017E21Rik	0.001625922	0.425490706	down
ASMM9PARTA014891	1500017E21Rik	0.001625922	0.425490706	down
ASMM9PARTA046584	AK083606	0.000000158	0.177903101	down
CUST_68_PI426073487	uc.70	0.000948	0.415883541	down
ASMM9PARTA007579		0.0000557	0.424466738	down
ASMM9PARTA012206	Mup-ps18	0.000000317	0.211528158	down
MM9LINCRNAEXON10524+_P1		0.000342	0.453116695	down
ASMM9PARTA002713		0.015162923	0.406542207	down
ASMM9PARTA004811		0.000822	0.409488168	down
ASMM9PARTA007225		0.0000417	0.390213081	down
ASMM9PARTA003788		0.00662248	0.498983371	down
ASMM9PARTA001521		0.002458062	0.16738584	down
ASMM9PARTA001685		0.000676	0.397302223	down
MM9LINCRNAEXON11596-_P1		0.033909272	0.430286878	down
ASMM9PARTA048146	AK014704	0.0000422	0.373615595	down
MM9LINCRNAEXON11333+_P1		0.007838967	0.403617592	down
ASMM9PARTA006490		0.0000209	0.452405412	down
ASMM9PARTA005371		0.001548424	0.410563335	down
ASMM9PARTA048305	2610044O15Rik	0.000726	0.453798173	down
ASMM9PARTA017522	A430108G06Rik	0.002335597	0.450952531	down
ASMM9PARTA005200		0.000314	0.488499429	down
ASMM9PARTA001881		0.000191	0.324010105	down
ASMM9PARTA009233		0.0000807	0.476629656	down
ASMM9PARTA000393		0.000568	0.180369637	down
ASMM9PARTA003080		0.002093591	0.485890611	down
ASMM9PARTA001942		0.0000578	0.36479273	down
ASMM9PARTA012988	Gm13508	0.000631	0.196610537	down
ASMM9PARTA002908		0.021026907	0.386527118	down
ASMM9PARTA046338	AK021003	0.000123	0.4893564	down
ASMM9PARTA046147	Gm10790	0.010687304	0.479638138	down
ASMM9PARTA007387		0.00000131	0.314139297	down
ASMM9PARTA006126		0.0000268	0.32936985	down
MM9LINCRNAEXON10292-_P1		0.00000114	0.483198325	down
ASMM9PARTA003798		0.00000677	0.437566808	down
ASMM9PARTA008689		0.0000947	0.402865664	down
ASMM9PARTA046883	AK016591	0.003495385	0.303002721	down
ASMM9PARTA011161	Gm11807	0.0000376	0.202570441	down
ASMM9PARTA016460	Scnm1	0.005445863	0.371627076	down
ASMM9PARTA016460	Scnm1	0.005445863	0.371627076	down
ASMM9PARTA016460	Scnm1	0.005445863	0.371627076	down
ASMM9PARTA005122		0.0000225	0.104280343	down
CUST_88_PI426073487	uc.90	0.029518077	0.444608594	down
ASMM9PARTA047974	2610528E23Rik	0.000274	0.145748085	down
ASMM9PARTA047974	2610528E23Rik	0.000274	0.145748085	down

ASMM9PARTA047974	2610528E23Rik	0.000274	0.145748085	down
ASMM9PARTA045237	Wbscr25	0.04477869	0.460976156	down
ASMM9PARTA047028	AK080999	0.000114	0.494002489	down
MM9LINCRNAEXON11965+_P1		0.0000664	0.42166272	down
ASMM9PARTA000019		0.004348087	0.134884529	down
ASMM9PARTA049400	AK083230	7.22E-08	0.108214837	down
MM9LINCRNAEXON10174-_P1	mouselincRNA1432	0.000142	0.479370115	down
ASMM9PARTA017947	Gm16243	0.001079275	0.44769557	down
ASMM9PARTA017947	Gm16243	0.001079275	0.44769557	down
ASMM9PARTA002081		0.03852829	0.493468939	down
ASMM9PARTA010972	Mup-ps6	0.000168	0.361079957	down
ASMM9PARTA004105		0.001003109	0.144357708	down
ASMM9PARTA004105		0.001003109	0.144357708	down
ASMM9PARTA004105		0.001003109	0.144357708	down
ASMM9PARTA002345		0.00000176	0.396403856	down
ASMM9PARTA010242	Gm13303	0.000944	0.378500147	down
ASMM9PARTA018257	Gm2862	0.0007	0.433041499	down
CUST_930_PI426073487	uc.451	0.000000324	0.040965072	down
MM9LINCRNAEXON11956-_P1		0.01615673	0.238427066	down
ASMM9PARTA051182	AK166159	0.021293415	0.375476658	down
MM9LINCRNAEXON10675-_P1	mouselincRNA1108	0.004215053	0.253028434	down
ASMM9PARTA015024	1700084C06Rik	0.002521763	0.434475737	down
ASMM9PARTA015024	1700084C06Rik	0.002521763	0.434475737	down
ASMM9PARTA015024	1700084C06Rik	0.002521763	0.434475737	down
ASMM9PARTA003886		0.0000322	0.361910956	down
ASMM9PARTA050373	AK044789	0.000577	0.350003498	down
ASMM9PARTA050373	AK044789	0.000577	0.350003498	down
ASMM9PARTA050373	AK044789	0.000577	0.350003498	down
ASMM9PARTA050373	AK044789	0.000577	0.350003498	down
ASMM9PARTA050373	AK044789	0.000577	0.350003498	down
ASMM9PARTA006725		0.000102	0.380803396	down
ASMM9PARTA045641	A530058N18Rik	0.00000564	0.136304592	down
ASMM9PARTA049263	mFLJ00088	0.00000136	0.33473042	down
ASMM9PARTA049263	mFLJ00088	0.00000136	0.33473042	down
ASMM9PARTA049263	mFLJ00088	0.00000136	0.33473042	down
ASMM9PARTA049263	mFLJ00088	0.00000136	0.33473042	down
ASMM9PARTA001729		0.011754142	0.45075794	down
MM9LINCRNAEXON10980-_P1	mouselincRNA0870	0.015735613	0.252876726	down
ASMM9PARTA044962	Gm11346	1.89E-08	0.474909352	down
ASMM9PARTA048874	AK084494	0.030334786	0.36925162	down
ASMM9PARTA017010	B630019A10Rik	0.024288595	0.437337802	down
ASMM9PARTA017112	Spata5l1	0.004330459	0.30310314	down
ASMM9PARTA008695		0.013166565	0.175783101	down
ASMM9PARTA051759	AK006734	0.000167	0.460945005	down
MM9LINCRNAEXON11302-_P1	mouselincRNA0654	0.00000982	0.161370279	down
ASMM9PARTA001799		0.0000919	0.448401538	down
MM9LINCRNAEXON11692+_P1	mouselincRNA0330	0.000122	0.412285175	down
ASMM9PARTA047473	AK139027	0.013969098	0.401531376	down
ASMM9PARTA004722		0.000329	0.427951681	down

ASMM9PARTA013654	Gm11549	0.000525	0.368183036	down
ASMM9PARTA011809	Gm13302	0.001075092	0.351007201	down
ASMM9PARTA047363	AK005679	0.000183	0.417549959	down
ASMM9PARTA001329		0.011712784	0.447161197	down
BX523225_P1	mouselincRNA0093	0.001517506	0.478508004	down
ASMM9PARTA044909	Gprc2a-rs5	0.031269647	0.375628516	down
ASMM9PARTA045845		0.046913672	0.180081217	down
ASMM9PARTA014591	D430036J16Rik	0.0000045	0.476117313	down
ASMM9PARTA001738		0.00000276	0.033384697	down
ASMM9PARTA050739	AK087755	0.0000418	0.490795089	down
ASMM9PARTA050739	AK087755	0.0000418	0.490795089	down
ASMM9PARTA050739	AK087755	0.0000418	0.490795089	down
ASMM9PARTA050739	AK087755	0.0000418	0.490795089	down
ASMM9PARTA050739	AK087755	0.0000418	0.490795089	down
ASMM9PARTA050739	AK087755	0.0000418	0.490795089	down
ASMM9PARTA014773	1700027L20Rik	0.00000293	0.110327114	down
ASMM9PARTA049940	DQ687153	4.11E-08	0.316224852	down
ASMM9PARTA009851	Gm10087	0.00033	0.414774087	down
ASMM9PARTA006426		0.018828645	0.36172493	down
ASMM9PARTA049034	AK046168	0.04949233	0.467118937	down
ASMM9PARTA004304		0.0000215	0.267441069	down
CUST_700_PI426073487	uc.221	0.001269442	0.389230506	down
ASMM9PARTA048873	AK163667	0.00000841	0.076403124	down
ASMM9PARTA049459	AK136882	0.006390123	0.486200062	down
ASMM9PARTA015587	Gm14453	0.031341296	0.46306666	down
ASMM9PARTA050807	AK038589	0.003236465	0.315316943	down
ASMM9PARTA001615		0.000018	0.263614892	down
ASMM9PARTA009100		0.000533	0.485551705	down
ASMM9PARTA015758	Gm11789	0.000655	0.36514085	down
ASMM9PARTA015015	H2-Ea-ps	0.000108	0.02235174	down
ASMM9PARTA014858	A830009L08Rik	0.000000818	0.210256271	down
ASMM9PARTA008461		0.000077	0.452635538	down
ASMM9PARTA050492	TCR-beta chain	0.00000633	0.194287871	down
ASMM9PARTA004677		0.004429004	0.423951118	down
ASMM9PARTA045562	Deaf1	0.04672281	0.280080797	down
ASMM9PARTA045562	Deaf1	0.04672281	0.280080797	down
ASMM9PARTA045562	Deaf1	0.04672281	0.280080797	down
ASMM9PARTA045562	Deaf1	0.04672281	0.280080797	down
ASMM9PARTA004064		0.0000537	0.485527329	down
ASMM9PARTA012505	Gm10601	0.0000483	0.435915268	down
ASMM9PARTA013857	Gm13480	0.00000179	0.447703888	down
ASMM9PARTA017269	A530058N18Rik	0.000000023	0.164670937	down
ASMM9PARTA049498	AK005639	0.003026774	0.452728634	down
ASMM9PARTA002826		0.0000049	0.419513168	down
ASMM9PARTA050491	AK018014	0.000000169	0.212603158	down
ASMM9PARTA017696	1700080G11Rik	0.000113	0.489871803	down
ASMM9PARTA008936		0.000139	0.347466172	down
ASMM9PARTA006929		0.006430599	0.332361464	down
ASMM9PARTA004743		0.0000155	0.149895674	down

ASMM9PARTA008669		0.03349627	0.470678118	down
ASMM9PARTA006044		0.004024913	0.393909808	down
ASMM9PARTA003873		0.023796065	0.365870033	down
ASMM9PARTA005503		1.52E-08	0.461531936	down
ASMM9PARTA015289	Gm13111	0.000227	0.487843691	down
ASMM9PARTA015289	Gm13111	0.000227	0.487843691	down
ASMM9PARTA005555		0.000531	0.226343039	down
ASMM9PARTA019819	RP23-122J17.9	0.0000021	0.171263082	down
ASMM9PARTA002205		0.001589582	0.344948354	down
ASMM9PARTA007853		0.0000013	0.104277896	down
ASMM9PARTA046128	BC049671	0.003969646	0.497564249	down
ASMM9PARTA045134	Gm5468	0.000613	0.492182227	down
ASMM9PARTA009224	1700027L20Rik	0.000000172	0.114644755	down
ASMM9PARTA009087		0.000714	0.428043052	down
ASMM9PARTA004928		0.01257143	0.332508756	down
ASMM9PARTA008950		0.002600775	0.164237531	down
ASMM9PARTA047197	AK046721	0.000443	0.219798891	down
ASMM9PARTA047197	AK046721	0.000443	0.219798891	down
ASMM9PARTA049046	AK077353	0.004440847	0.496541514	down
ASMM9PARTA049978	Mup1	0.0000011	0.335738271	down
ASMM9PARTA005801		0.00318744	0.473007429	down
ASMM9PARTA005801		0.00318744	0.473007429	down
BG808609_P1	humanlincRNA0438	0.001347311	0.430519956	down
ASMM9PARTA002368		0.00000469	0.347485853	down
ASMM9PARTA003797		0.0000378	0.307016399	down
ASMM9PARTA002621		0.000246	0.086780335	down
ASMM9PARTA050421	TCR-beta chain	0.000818	0.313309965	down
ASMM9PARTA050421	TCR-beta chain	0.000818	0.313309965	down
ASMM9PARTA050421	TCR-beta chain	0.000818	0.313309965	down
ASMM9PARTA050421	TCR-beta chain	0.000818	0.313309965	down
ASMM9PARTA050421	TCR-beta chain	0.000818	0.313309965	down
ASMM9PARTA050421	TCR-beta chain	0.000818	0.313309965	down
ASMM9PARTA050421	TCR-beta chain	0.000818	0.313309965	down
ASMM9PARTA050421	TCR-beta chain	0.000818	0.313309965	down
ASMM9PARTA050421	TCR-beta chain	0.000818	0.313309965	down
ASMM9PARTA001674		0.002199233	0.493824405	down
ASMM9PARTA007187		0.00000796	0.328667569	down
ASMM9PARTA047159	AK019435	0.00000313	0.397010274	down
ASMM9PARTA003905		0.00000792	0.293760012	down
ASMM9PARTA008035		0.008928032	0.401773735	down
ASMM9PARTA050559	TCR-beta chain	0.000000291	0.19535728	down
ASMM9PARTA017215	1500002F19Rik	0.0000716	0.288207013	down
ASMM9PARTA008042		0.034899622	0.404423585	down
MM9LINCRNAEXON10875+_P1		0.00000243	0.286688322	down
ASMM9PARTA048242	AK139359	0.000348	0.474836829	down
CUST_160_PI426073487	uc.162	0.00000474	0.22122678	down
ASMM9PARTA005938		0.00000419	0.499296491	down
ASMM9PARTA000690		0.000000723	0.335922399	down
ASMM9PARTA000047		0.000164	0.087415545	down

ASMM9PARTA019614	CT571259.1	0.003513301	0.488276172	down
ASMM9PARTA008846		0.001092117	0.232771044	down
ASMM9PARTA044922	3110039M20Rik	0.0000751	0.433268036	down
ASMM9PARTA002584		0.000000885	0.012418658	down
MM9LINCRNAEXON11907+_P1	mouselincRNA0173	0.0000566	0.389431226	down
ASMM9PARTA045931	Lrch4-sap25	0.023233835	0.486565773	down
ASMM9PARTA045931	Lrch4-sap25	0.023233835	0.486565773	down
ASMM9PARTA045931	Lrch4-sap25	0.023233835	0.486565773	down
ASMM9PARTA009795	Gm13775	0.0000999	0.2427189	down
ASMM9PARTA015665	A330009N23Rik	0.000208	0.431610459	down
ASMM9PARTA009187		0.0000845	0.497806565	down
ASMM9PARTA003810		0.00000731	0.058065097	down
ASMM9PARTA047791	AK159003	0.00028	0.4993024	down
ASMM9PARTA047791	AK159003	0.00028	0.4993024	down
ASMM9PARTA016031	Gm15880	0.0000343	0.236031034	down
ASMM9PARTA018831	Gm15894	0.004041379	0.376846861	down
ASMM9PARTA018831	Gm15894	0.004041379	0.376846861	down
ASMM9PARTA000149		0.005950768	0.166005054	down
ASMM9PARTA013178	Gm13300	0.0000204	0.452667174	down
ASMM9PARTA018948	Gm4876	0.0000541	0.4549947	down
ASMM9PARTA050392	M34473	0.000000682	0.21291629	down
ASMM9PARTA002466		0.000156	0.266984107	down
ASMM9PARTA004363		0.02193925	0.244204776	down
ASMM9PARTA010263	Gm10805	0.007051962	0.367582813	down
ASMM9PARTA014096	Gm13016	0.0000971	0.469752032	down
ASMM9PARTA007162		0.001250507	0.471284139	down
MM9LINCRNAEXON10517+_P1	mouselincRNA1347	0.016411059	0.494846544	down
ASMM9PARTA017777	2900041M22Rik	0.002660395	0.214806191	down
ASMM9PARTA015093	A530058N18Rik	0.00000139	0.30197708	down
ASMM9PARTA004062		0.00000486	0.043599327	down
ASMM9PARTA051011	Pgls	0.010097165	0.408227815	down
ASMM9PARTA051011	Pgls	0.010097165	0.408227815	down
ASMM9PARTA048378	AK016016	0.034725863	0.388578874	down
ASMM9PARTA006892		0.022061676	0.348312137	down
ASMM9PARTA046477	AK193439	0.000549	0.403561055	down
ASMM9PARTA046477	AK193439	0.000549	0.403561055	down
ASMM9PARTA046477	AK193439	0.000549	0.403561055	down
ASMM9PARTA014287	Gm10706	0.005554561	0.431426244	down
ASMM9PARTA012774	Gm15428	0.0000483	0.410379398	down
ASMM9PARTA047276	BC064072	0.001185206	0.429484322	down
ASMM9PARTA011877	Gm13626	0.037082683	0.495645801	down
ASMM9PARTA000547		0.001922868	0.364771639	down
ASMM9PARTA051324	AK038730	0.00019	0.476194603	down
ASMM9PARTA005636		0.0000224	0.409622558	down
ASMM9PARTA004402		0.000442	0.399451058	down
ASMM9PARTA004402		0.000442	0.399451058	down
ASMM9PARTA004402		0.000442	0.399451058	down
AK007918_P1	mouselincRNA0359	0.023077872	0.493832891	down
AK007918_P1	mouselincRNA0359	0.023077872	0.493832891	down

ASMM9PARTA050068	AK021093	0.000935	0.251328112	down
ASMM9PARTA005194		0.000572	0.446717981	down
ASMM9PARTA014192	4930511A02Rik	0.041627042	0.373389013	down
ASMM9PARTA047841	AK086741	0.0000105	0.426285024	down
ASMM9PARTA009136		0.000689	0.100150235	down
CUST_76_PI426409190	Mup-ps11	0.000006	0.220915946	down
CUST_76_PI426409190	Mup-ps11	0.000006	0.220915946	down
ASMM9PARTA017021	Gm13003	0.00011	0.454342487	down
ASMM9PARTA050468	AK080173	0.019126195	0.388137584	down
ASMM9PARTA050468	AK080173	0.019126195	0.388137584	down
ASMM9PARTA050468	AK080173	0.019126195	0.388137584	down
ASMM9PARTA051821		0.009639057	0.380137554	down
ASMM9PARTA045468	5330413P13Rik	0.00000834	0.391804085	down
ASMM9PARTA003547		0.01638323	0.343001694	down
ASMM9PARTA012454	Gm13230	0.000562	0.45358612	down
ASMM9PARTA003479		0.00025	0.084363617	down
ASMM9PARTA050635	AK030803	0.008517969	0.494003904	down
MM9LINCRNAEXON11853-_P1		0.000599	0.442515823	down
CUST_502_PI426073487	uc.22	0.000184	0.417572032	down
ASMM9PARTA046197	Cps1	0.002927542	0.369647818	down
ASMM9PARTA003515		0.000000148	0.162024915	down
ASMM9PARTA003736		0.000256	0.024589549	down
CUST_100_PI426409190	AC087559.13	0.0000025	0.337838637	down
ASMM9PARTA048949	AK170301	0.000153	0.053292166	down
humanlincRNA0127+_P1	humanlincRNA0127	0.0413916	0.398952606	down
CUST_149_PI426073487	uc.151	0.02337389	0.418209896	down
ASMM9PARTA015150	2900093L17Rik	0.000773	0.348367298	down
ASMM9PARTA050674	AK020619	0.000122	0.357938375	down
ASMM9PARTA013545	1700091E21Rik	0.000607	0.419021515	down
ASMM9PARTA002350		0.000724	0.45371672	down
ASMM9PARTA011946	Mup-ps10	0.000000949	0.232516233	down
ASMM9PARTA019132	9230115E21Rik	0.0000229	0.077612894	down
ASMM9PARTA051652	AK136006	0.000122	0.263318547	down
ASMM9PARTA045880	Gm3230	0.006387503	0.475633487	down
ASMM9PARTA009046		0.002047392	0.485047442	down
ASMM9PARTA008624		0.0000888	0.286928156	down
ASMM9PARTA000310		0.002468612	0.140732187	down
MM9LINCRNAEXON10816-_P1		0.0000143	0.449514125	down
ASMM9PARTA005158		0.002328441	0.404611732	down
ASMM9PARTA016484	1810019D21Rik	0.002046348	0.279333978	down
ASMM9PARTA050731	AK014683	0.0000291	0.337652323	down
ASMM9PARTA016427	Gm14285	0.000101	0.412138994	down
ASMM9PARTA015880	BB218582	0.011721686	0.301897109	down
ASMM9PARTA046840	AK179786	0.00148844	0.336422409	down
ASMM9PARTA047612	AK039634	1.78E-08	0.03853192	down
ASMM9PARTA018235	7SK.234	0.000408	0.350196399	down
ASMM9PARTA005211		0.00000217	0.431717601	down
ASMM9PARTA003926		0.00000101	0.026465998	down
ASMM9PARTA051057	AK051019	0.01563291	0.432463682	down

ASMM9PARTA051057	AK051019	0.01563291	0.432463682	down
ASMM9PARTA010293	Gm10593	0.0000588	0.448073843	down
ASMM9PARTA005997		0.0179224	0.389905396	down
AK139572_P1	humanlincRNA1231	0.002131491	0.430025776	down
ASMM9PARTA013275	Gm14589	0.017658768	0.492222394	down
ASMM9PARTA046329	4930418G15Rik	0.000471	0.369242649	down
ASMM9PARTA046329	4930418G15Rik	0.000471	0.369242649	down
ASMM9PARTA017502	1700108N11Rik	0.035066187	0.460275317	down
ASMM9PARTA017502	1700108N11Rik	0.035066187	0.460275317	down
ASMM9PARTA003508		0.000000532	0.385579333	down
CUST_699_PI426073487	uc.220	0.000625	0.286356871	down
ASMM9PARTA049082	AK085201	0.000313	0.37398748	down
ASMM9PARTA011888	Gm13408	0.000006	0.38247128	down
ASMM9PARTA013819	Gm16266	0.00000977	0.437982969	down
ASMM9PARTA007842		0.000117	0.499600569	down
humanlincRNA1948-_P1	humanlincRNA1948	0.024332624	0.388409639	down
ASMM9PARTA013585	Gm13935	0.0000375	0.23309342	down
ASMM9PARTA051041	Scoc	0.000201	0.111485492	down
ASMM9PARTA051041	Scoc	0.000201	0.111485492	down
ASMM9PARTA007011		0.000583	0.467851754	down
ASMM9PARTA046156	A730018C14Rik	0.04429631	0.410015229	down
humanlincRNA1634+_P1	humanlincRNA1634	0.007780833	0.464125748	down
ASMM9PARTA010625	Gm15087	0.00984336	0.260482737	down
ASMM9PARTA002528		0.016655196	0.473858326	down
ASMM9PARTA009357	H2-Ea-ps	0.010612574	0.29308705	down
ASMM9PARTA004103		0.000137	0.01988614	down
ASMM9PARTA007499		0.033189148	0.355846888	down
ASMM9PARTA050300	TCR-beta chain	0.00000369	0.222068373	down
ASMM9PARTA049128	AK043120	0.0000184	0.474642238	down
humanlincRNA1154-_P1	humanlincRNA1154	0.048110716	0.281055036	down
ASMM9PARTA045668	Hhatl	0.014825908	0.346758974	down
ASMM9PARTA050891	AK032343	0.001663768	0.330650818	down
ASMM9PARTA003942		0.000156	0.484500115	down
ASMM9PARTA049167	AK019067	0.000474	0.113237536	down
CUST_21_PI426073487	uc.22	0.016955696	0.372196961	down
mouselincRNA1407+_P1	mouselincRNA1407	0.036058847	0.49301421	down
ASMM9PARTA016225	1700030C12Rik	0.034226015	0.379911164	down
ASMM9PARTA010260	Mup-ps2	0.0000205	0.190253847	down
ASMM9PARTA051175	Wdr17	0.004097767	0.414616217	down
ASMM9PARTA051175	Wdr17	0.004097767	0.414616217	down
ASMM9PARTA013915	Gm11648	0.0000734	0.347020794	down
ASMM9PARTA013915	Gm11648	0.0000734	0.347020794	down
ASMM9PARTA002438		0.001486718	0.236295678	down
ASMM9PARTA012336	Gm5678	0.00082	0.166052411	down
ASMM9PARTA002106		0.023237731	0.447618938	down
ASMM9PARTA007814		0.011178087	0.485568586	down
ASMM9PARTA019073	Gm15024	0.023464035	0.305623005	down
ASMM9PARTA051350	BC058420	0.00000943	0.473367291	down
ASMM9PARTA003360		0.000014	0.435744581	down

ASMM9PARTA004121		0.00000179	0.488226325	down
ASMM9PARTA006718		0.00000383	0.422735922	down
ASMM9PARTA047471	Tc2n	0.000109	0.204568296	down
ASMM9PARTA047471	Tc2n	0.000109	0.204568296	down
ASMM9PARTA003746		0.008565307	0.145315227	down
ASMM9PARTA008070		0.005794246	0.399759089	down
ASMM9PARTA045912		0.00111956	0.123764107	down
ASMM9PARTA047208	AK217959	0.00012	0.2360307	down
ASMM9PARTA013610	A430104N18Rik	0.002459263	0.417326738	down
ASMM9PARTA005442		0.000000642	0.09485516	down
ASMM9PARTA048270	AK213404	0.030380491	0.317101339	down
ASMM9PARTA006348		0.017603844	0.363070844	down
MM9LINCRNAEXON11498+_P1	mouselincRNA0517	0.01326116	0.3968863	down
ASMM9PARTA016121	AI115009	0.015477298	0.357065833	down
ASMM9PARTA006074		0.000473	0.236903501	down
ASMM9PARTA048254	AK151508	0.0000262	0.390559398	down
ASMM9PARTA008059		0.0000579	0.373799636	down
ASMM9PARTA045219	B130006D01Rik	0.00000118	0.118278317	down
ASMM9PARTA004494		0.009845706	0.48784224	down
ASMM9PARTA008863		0.000246	0.337201757	down
ASMM9PARTA003400		0.000369	0.429839473	down
ASMM9PARTA011822	Gm13312	0.00059	0.463913169	down
CUST_836_PI426073487	uc.357	0.010633458	0.389478351	down
ASMM9PARTA047055	AK043502	0.000128	0.365846342	down
ASMM9PARTA002513		0.000335	0.322723914	down
ASMM9PARTA049433	AK144823	0.00014	0.464487903	down
MM9LINCRNAEXON10944-_P1	mouselincRNA0866	0.010125405	0.302818596	down
ASMM9PARTA014930	Gm13383	0.000737	0.472894402	down
ASMM9PARTA005745		0.0000263	0.455922922	down
BB202824_P1	mouselincRNA0958	0.0000508	0.140062594	down
ASMM9PARTA006336		0.005042215	0.265836194	down
ASMM9PARTA005733		0.00000262	0.248457452	down
ASMM9PARTA009012		0.00000209	0.492830208	down
ASMM9PARTA015935	Gm16701	0.001799597	0.451019507	down
ASMM9PARTA000027		0.001059296	0.139040803	down
ASMM9PARTA004550		0.00405342	0.387664335	down
ASMM9PARTA004550		0.00405342	0.387664335	down
ASMM9PARTA006146		0.003528568	0.453201506	down
ASMM9PARTA006146		0.003528568	0.453201506	down
ASMM9PARTA006146		0.003528568	0.453201506	down
ASMM9PARTA044993	A230056P14Rik	0.026065445	0.285091971	down
ASMM9PARTA044993	A230056P14Rik	0.026065445	0.285091971	down
ASMM9PARTA002403		0.024782956	0.434475548	down
ASMM9PARTA050312	AK006413	0.041590855	0.379883093	down
MM9LINCRNAEXON11005+_P1	mouselincRNA0885	0.001336427	0.332339373	down
ASMM9PARTA016561	Gm13589	0.001552763	0.475488971	down
BU698035_P1	mouselincRNA1195	0.01063636	0.375872122	down
ASMM9PARTA014079	Gm13781	0.042472877	0.414111993	down
AK156563_P1	humanlincRNA1111	0.000132	0.444859845	down

AK156563_P1	humanlincRNA1111	0.000132	0.444859845	down
AK156563_P1	humanlincRNA1111	0.000132	0.444859845	down
ASMM9PARTA045066	Dlx6os1	0.00016	0.164637683	down
ASMM9PARTA016030	Gm13601	0.000983	0.454961931	down
ASMM9PARTA016030	Gm13601	0.000983	0.454961931	down
ASMM9PARTA050154	Tcrb	0.0000471	0.192232995	down
ASMM9PARTA003483		0.000000154	0.257359133	down
ASMM9PARTA008823		0.007360342	0.475785832	down
ASMM9PARTA002388		0.008766177	0.340258999	down
ASMM9PARTA007439		0.002319333	0.323349811	down
ASMM9PARTA050813	AK046042	0.0000683	0.390996982	down
ASMM9PARTA050813	AK046042	0.0000683	0.390996982	down
ASMM9PARTA048261	AK018990	0.03202989	0.496868906	down
ASMM9PARTA002960		0.012800043	0.281409825	down
ASMM9PARTA018669	RP24-468F14.2	0.000136	0.487900626	down
ASMM9PARTA045109	9130024F11Rik	0.00000407	0.307762951	down
ASMM9PARTA019857		0.000000857	0.068963708	down
ASMM9PARTA050429	AK190196	0.0000477	0.413691792	down
ASMM9PARTA050429	AK190196	0.0000477	0.413691792	down
ASMM9PARTA050429	AK190196	0.0000477	0.413691792	down
MM9LINCRNAEXON10279-_P1	mouselincRNA1497	0.00000146	0.068619801	down
ASMM9PARTA008115		0.00000509	0.441309694	down
MM9LINCRNAEXON11963+_P1		0.0018236	0.383460358	down
ASMM9PARTA018350	A530040E14Rik	0.000228	0.200950052	down
ASMM9PARTA048519	AK020087	0.00000413	0.398156694	down
ASMM9PARTA047122	AK053157	0.000234	0.318997144	down
ASMM9PARTA049957	AK037159	0.024685148	0.443568328	down
ASMM9PARTA049957	AK037159	0.024685148	0.443568328	down
AK132675_P1	humanlincRNA1194	0.000828	0.489370146	down
ASMM9PARTA010261	Gm10601	0.0000747	0.438413242	down
ASMM9PARTA014711	Fam150a	0.0000946	0.339497649	down
ASMM9PARTA019172	RP24-486L9.1	0.0000255	0.238654793	down
ASMM9PARTA019172	RP24-486L9.1	0.0000255	0.238654793	down
ASMM9PARTA000903		0.027537983	0.395299978	down
ASMM9PARTA007772		0.021485496	0.404072371	down
ASMM9PARTA046203	AK138346	0.000105	0.410905194	down
ASMM9PARTA015743	Gm16885	0.005962122	0.34962524	down
ASMM9PARTA015743	Gm16885	0.005962122	0.34962524	down
ASMM9PARTA005348		0.000142	0.297042379	down
ASMM9PARTA015727	Gm13919	0.026349675	0.386608291	down
ASMM9PARTA000988		0.01956945	0.407450227	down
ASMM9PARTA006626		0.0000373	0.306111173	down
ASMM9PARTA050618	AK007376	0.00576279	0.340373784	down
ASMM9PARTA051621	AK052495	0.0000258	0.417948305	down
ASMM9PARTA049986	DQ266428	0.028635023	0.454645518	down
ASMM9PARTA009936	Mup-ps7	0.0000304	0.202290699	down
ASMM9PARTA046304	AK163333	0.03011973	0.328204643	down
ASMM9PARTA046304	AK163333	0.03011973	0.328204643	down
ASMM9PARTA046304	AK163333	0.03011973	0.328204643	down

ASMM9PARTA047796	AK043267	0.001465006	0.359384389	down
ASMM9PARTA047796	AK043267	0.001465006	0.359384389	down
ASMM9PARTA012215	Mup-ps19	0.0000359	0.128890091	down
ASMM9PARTA050847	Tgfa	0.00000688	0.49883828	down
ASMM9PARTA049404	Myef2	0.0000185	0.411291633	down
ASMM9PARTA049404	Myef2	0.0000185	0.411291633	down
ASMM9PARTA049404	Myef2	0.0000185	0.411291633	down
ASMM9PARTA049404	Myef2	0.0000185	0.411291633	down
ASMM9PARTA000029		0.000856	0.107190591	down
ASMM9PARTA049257	AK139461	0.000188	0.278126262	down
ASMM9PARTA047918	AK014089	0.000124	0.44583559	down
ASMM9PARTA004228		0.004064383	0.277565949	down
ASMM9PARTA010235	Gm13300	0.0000149	0.310438812	down
CUST_103_PI426409190	AC087559.5	0.0000416	0.400792848	down
ASMM9PARTA019341	RP23-143B14.2	0.000709	0.433330837	down
ASMM9PARTA004298		0.00000428	0.222821895	down
CUST_301_PI426073487	uc.303	0.000622	0.375254136	down
ASMM9PARTA012583	Gm15368	0.034053914	0.438150231	down
ASMM9PARTA012583	Gm15368	0.034053914	0.438150231	down
ASMM9PARTA012583	Gm15368	0.034053914	0.438150231	down
ASMM9PARTA012583	Gm15368	0.034053914	0.438150231	down
ASMM9PARTA012583	Gm15368	0.034053914	0.438150231	down
ASMM9PARTA012583	Gm15368	0.034053914	0.438150231	down
ASMM9PARTA009161		0.000309	0.269478927	down
ASMM9PARTA006315		0.027379101	0.435897654	down
ASMM9PARTA011545	H2-M6-ps	0.000312	0.489450865	down
ASMM9PARTA009180		0.0000504	0.355306708	down
ASMM9PARTA002933		0.029462766	0.480671571	down
ASMM9PARTA006662		0.00000565	0.207552245	down
ASMM9PARTA016242	4933400F03Rik	0.00023	0.438004378	down
ASMM9PARTA015861	D430036J16Rik	0.0000789	0.427207203	down
ASMM9PARTA044847	Six3os1	0.023333002	0.483660918	down
ASMM9PARTA050208	AK017143	0.002412934	0.417674655	down
ASMM9PARTA003967		0.000303	0.365362602	down
ASMM9PARTA048273	AK038079	0.0000382	0.437496514	down
ASMM9PARTA050389	Tcrb	0.00000049	0.203147321	down
ASMM9PARTA013859	B130046B21Rik	0.0000039	0.322692026	down
ASMM9PARTA013859	B130046B21Rik	0.0000039	0.322692026	down
ASMM9PARTA011720	Gm13627	0.000801	0.402114705	down
ASMM9PARTA006007		0.023450429	0.447348852	down
ASMM9PARTA002209		0.0000367	0.483487804	down
ASMM9PARTA018824	AC107837.1	0.001857252	0.496943499	down
ASMM9PARTA018824	AC107837.1	0.001857252	0.496943499	down
BE949468_P1	mouselincRNA1019	0.002107645	0.421922698	down
ASMM9PARTA046906	AK007042	0.011831683	0.30875551	down
ASMM9PARTA003111		0.0000783	0.343982315	down
ASMM9PARTA009352		0.00258156	0.185635351	down
ASMM9PARTA019428	Gm10497	0.037693787	0.336343179	down
ASMM9PARTA019428	Gm10497	0.037693787	0.336343179	down

ASMM9PARTA019428	Gm10497	0.037693787	0.336343179	down
ASMM9PARTA007267		0.000167	0.491495168	down
ASMM9PARTA014317	Gm14120	0.002399707	0.349891175	down
ASMM9PARTA000507		0.0000069	0.466609516	down
ASMM9PARTA050514	AK039340	0.001683929	0.323640034	down
ASMM9PARTA000022		0.000296	0.487496851	down
ASMM9PARTA003178		0.001544726	0.445258773	down
CA874578_P1	mouselincRNA0103	0.017573824	0.37807991	down
ASMM9PARTA005659		0.001280571	0.395075573	down
MM9LINCRNAEXON11972+_P1		0.0000152	0.475855383	down
ASMM9PARTA015997	Gm15631	0.00000258	0.497967496	down
ASMM9PARTA015997	Gm15631	0.00000258	0.497967496	down
ASMM9PARTA016971	Gm15535	0.0001	0.366023904	down
ASMM9PARTA046137	AK144829	0.005679592	0.462896422	down
ASMM9PARTA015931	Mup-ps2	0.0000354	0.337265319	down
ASMM9PARTA051808		0.000275	0.130511916	down
ASMM9PARTA050787	AK008222	0.013717309	0.435639265	down
ASMM9PARTA047889	AK136126	0.001251614	0.472807203	down
ASMM9PARTA006222		0.000626	0.350324663	down
ASMM9PARTA006222		0.000626	0.350324663	down
ASMM9PARTA006222		0.000626	0.350324663	down
ASMM9PARTA006222		0.000626	0.350324663	down
ASMM9PARTA008425		0.000306	0.351188617	down
ASMM9PARTA007151		0.003838007	0.472875171	down
ASMM9PARTA016218	Gm12371	0.000317	0.47506428	down
ASMM9PARTA019274	AC157896.1	0.0000114	0.345717092	down
ASMM9PARTA049345	AK142074	0.00000604	0.165453547	down
ASMM9PARTA049168	Ccdc34	0.0000464	0.348566308	down
ASMM9PARTA005438		0.001121801	0.458839925	down
ASMM9PARTA051397	AK018937	0.00064	0.459391239	down
ASMM9PARTA051397	AK018937	0.00064	0.459391239	down
ASMM9PARTA051397	AK018937	0.00064	0.459391239	down
ASMM9PARTA050630	AK131825	0.0000933	0.431225895	down
ASMM9PARTA006619		0.000012	0.479263629	down
ASMM9PARTA019679	Gm3892	0.00000184	0.29196579	down
ASMM9PARTA046463	AK144329	0.000254	0.278920959	down
ASMM9PARTA005334		0.00000147	0.322905843	down
ASMM9PARTA004045		0.0000129	0.421610471	down
ASMM9PARTA005379		0.004583365	0.477264321	down
ASMM9PARTA017530	Gm16129	0.002364924	0.39793578	down
ASMM9PARTA017530	Gm16129	0.002364924	0.39793578	down
ASMM9PARTA017530	Gm16129	0.002364924	0.39793578	down
ASMM9PARTA003790		0.0000859	0.405758772	down
BG794789_P1	humanlincRNA1357	0.000205	0.439104099	down
ASMM9PARTA017182	Gm14820	0.047283363	0.387430244	down
