# Supplementary material for: Microarray Profile of Long Noncoding RNA and Messenger RNA Expression in a Model of Alzheimer’s Disease
Source: Life (Basel). 2020 May 14;10(5):64. doi: 10.3390/life10050064 (PMC7281340; doi:10.3390/life10050064)
Supplement: Supplementary file 1 [file life-10-00064-s001.zip › life-787240-supplemenatry-to be published - PDF/life-787240-supplementary/Table S4.pdf]

life

Supplementary

Microarray Profile of Long Noncoding RNA and Messenger RNA Expression in a Model of Alzheimer's Disease

Linlin Wang [†], Li Zeng [†], Hailun Jiang, Zhuorong Li * and Rui Liu *

Institute of Medicinal Biotechnology, Chinese Academy of Medical Sciences and Peking Union Medical College, Beijing 100050, China; wanglinlin@wfmc.edu.cn (L.W.); zengsheng@imb.pumc.edu.cn (L.Z.); jianghailun@imb.pumc.edu.cn (H.J.)

* Correspondence: lizhourong@imb.pumc.edu.cn (Z.L.); +86-10-8352017; .liurui@imb.pumc.edu.cn (R.L.); Tel.: +86-10-67087731

Table S4. Differently expressed lncRNAs in the brain of 9-month-old.APP/PS1 mice compared with age-matched WT mice.

Probe Name	Gene Symbol	*p*-Value	Fold Change	Regulation
ASMM9PARTA014130	Gm16834	0.0000174	2.4332428	up
ASMM9PARTA006853		0.0000576	89.55974	up
ASMM9PARTA016470	Gm16836	0.0000959	2.4933932	up
MM9LINCRNAEXON10451+_P1	mouselincRNA1286	0.003183203	2.4396448	up
ASMM9PARTA010954	Gm14158	0.000285	2.3584278	up
MM9LINCRNAEXON10103-_P1	mouselincRNA1524	0.00046	2.0816717	up
ASMM9PARTA012889	Gm12983	0.0000247	2.3748605	up
ASMM9PARTA015833	Gm15086	0.020345824	2.7550943	up
MM9LINCRNAEXON10482-_P1		0.005871455	2.0287292	up
ASMM9PARTA014628	Gm12940	0.000724	2.2870247	up
mouselincRNA0155+_P1	mouselincRNA0155	0.040218018	2.235328	up
ASMM9PARTA000080		0.000669	2.143498	up
ASMM9PARTA009048		0.004307814	2.6057537	up
MM9LINCRNAEXON11250-_P1	mouselincRNA0737	0.018313346	2.563409	up
ASMM9PARTA051822		0.000399	2.1336646	up
ASMM9PARTA046301	AK144771	0.000183	2.120443	up
ASMM9PARTA004288		0.000105	2.3698492	up
humanlincRNA1590+_P1	humanlincRNA1590	0.007291022	2.2136614	up
ASMM9PARTA046759	AK039862	0.000135	19.392376	up
ASMM9PARTA016398	4632427E13Rik	0.000518	2.4383655	up
ASMM9PARTA018203	NRON.1	0.000195	2.4120655	up
ASMM9PARTA006958		0.000435	3.0653234	up
ASMM9PARTA016545	Gm16706	0.016001754	3.0476017	up
ASMM9PARTA016545	Gm16706	0.016001754	3.0476017	up
ASMM9PARTA012922	Gm13153	0.0000147	2.4737248	up

ASMM9PARTA012922	Gm13153	0.0000147	2.4737248	up
ASMM9PARTA012922	Gm13153	0.0000147	2.4737248	up
ASMM9PARTA015256	9130017K11Rik	0.0000641	3.2698576	up
ASMM9PARTA015256	9130017K11Rik	0.0000641	3.2698576	up
ASMM9PARTA050547	BC049735	0.019010011	2.0642784	up
ASMM9PARTA018387	Gm16184	0.000389	2.5548847	up
ASMM9PARTA050284	Gak	0.0000833	2.7930596	up
ASMM9PARTA046970	BC006965	0.000548	4.090801	up
ASMM9PARTA011525	Gm13314	0.000141	2.3245313	up
CA465684_P1	mouselincRNA0967	0.02853576	2.0828776	up
ASMM9PARTA013323	Mup-ps15	0.0000818	6.33789	up
ASMM9PARTA011112	Gm13469	0.000409	2.0476577	up
ASMM9PARTA016364	Gm15843	0.00000489	2.5534022	up
ASMM9PARTA014585	Gm8093	0.021374123	2.221592	up
ASMM9PARTA004295		0.004375473	2.6300242	up
ASMM9PARTA009250		0.0000565	2.7566524	up
ASMM9PARTA011368	Gm5759	0.000534	2.4414752	up
ASMM9PARTA002301		0.000698	2.3251016	up
CJ058094_P1	mouselincRNA1482	0.0000258	6.6150665	up
CJ058094_P1	mouselincRNA1482	0.0000258	6.6150665	up
CJ058094_P1	mouselincRNA1482	0.0000258	6.6150665	up
ASMM9PARTA012572	Gm13323	0.001656133	2.1485894	up
ASMM9PARTA012883	Gm12706	0.000126	2.5805876	up
ASMM9PARTA014191	Gm12092	0.000209	3.625209	up
ASMM9PARTA049442	LOC100043381	0.0000269	2.831089	up
ASMM9PARTA049442	LOC100043381	0.0000269	2.831089	up
ASMM9PARTA049442	LOC100043381	0.0000269	2.831089	up
ASMM9PARTA049442	LOC100043381	0.0000269	2.831089	up
ASMM9PARTA049442	LOC100043381	0.0000269	2.831089	up
ASMM9PARTA049442	LOC100043381	0.0000269	2.831089	up
ASMM9PARTA049442	LOC100043381	0.0000269	2.831089	up
ASMM9PARTA049442	LOC100043381	0.0000269	2.831089	up
ASMM9PARTA003398		0.001134059	3.3080199	up
ASMM9PARTA012940	Gm14324	0.00076	2.632217	up
ASMM9PARTA051273	BC039997	0.0000479	4.436292	up
ASMM9PARTA013248	Gm12924	0.00821248	2.148568	up
ASMM9PARTA013248	Gm12924	0.00821248	2.148568	up
ASMM9PARTA049385	AK137033	0.001777993	2.2938242	up
ASMM9PARTA013083	Gm12539	0.00000589	2.224736	up
CUST_282_PI426073487	uc.284	0.004038211	3.4073362	up
ASMM9PARTA009863	Gm7731	0.000881	2.2065191	up
ASMM9PARTA013061	Gm13913	0.0000648	5.3009806	up
ASMM9PARTA000138		0.018494261	2.188609	up
ASMM9PARTA049079	AK047177	0.035374727	2.8497887	up
ASMM9PARTA049464	AK052881	0.006231084	4.531504	up
ASMM9PARTA049464	AK052881	0.006231084	4.531504	up
ASMM9PARTA017430	A830052D11Rik	0.00000708	3.8275795	up
ASMM9PARTA017430	A830052D11Rik	0.00000708	3.8275795	up
ASMM9PARTA008375		0.005777075	3.8994133	up

ASMM9PARTA004844		0.016606377	2.0014207	up
ASMM9PARTA016228	Gm16093	0.0000913	2.3423448	up
ASMM9PARTA016228	Gm16093	0.0000913	2.3423448	up
ASMM9PARTA048616	AK020462	0.014852415	2.2346213	up
BY732216_P1	humanlincRNA0653	0.00000154	2.0202749	up
ASMM9PARTA007917		0.012555691	2.033057	up
ASMM9PARTA000849		0.034926616	4.464988	up
ASMM9PARTA009659	Gm7666	0.000476	2.033739	up
ASMM9PARTA005541		0.013500612	3.8303263	up
ASMM9PARTA016047	G630018N14Rik	0.00683265	2.0304508	up
ASMM9PARTA051010	AK170106	0.000244	3.4403698	up
ASMM9PARTA010969	Gm15399	0.00526151	2.1195905	up
ASMM9PARTA012807	Gm14633	0.000256	2.3599093	up
ASMM9PARTA005182		0.0000912	25.348322	up
MM9LINCRNAEXON11747+_P1	mouselincRNA0230	0.009304563	2.0012047	up
ASMM9PARTA011323	Gm12677	0.00000648	3.3582802	up
ASMM9PARTA014414	5430427M07Rik	0.019764079	2.8032463	up
ASMM9PARTA051477	AK028224	0.000000549	3.2538602	up
humanlincRNA0255+_P1	humanlincRNA0255	0.01382096	2.0040312	up
ASMM9PARTA007950		0.042229388	2.3446157	up
ASMM9PARTA050830	AK081140	0.00000156	3.4868026	up
ASMM9PARTA050830	AK081140	0.00000156	3.4868026	up
ASMM9PARTA050830	AK081140	0.00000156	3.4868026	up
humanlincRNA1422+_P1	humanlincRNA1422	0.009498736	2.1592977	up
ASMM9PARTA047940	AK014435	0.000289	6.456377	up
ASMM9PARTA003773		0.00000327	4.4744334	up
ASMM9PARTA013174	Gm11645	0.013890307	2.1863253	up
ASMM9PARTA010441	Gm15560	0.000165	2.5293121	up
ASMM9PARTA015224	BC065397	0.008927985	3.6267095	up
ASMM9PARTA015224	BC065397	0.008927985	3.6267095	up
ASMM9PARTA015224	BC065397	0.008927985	3.6267095	up
ASMM9PARTA015224	BC065397	0.008927985	3.6267095	up
ASMM9PARTA015224	BC065397	0.008927985	3.6267095	up
ASMM9PARTA015224	BC065397	0.008927985	3.6267095	up
ASMM9PARTA015224	BC065397	0.008927985	3.6267095	up
ASMM9PARTA015224	BC065397	0.008927985	3.6267095	up
AI507909_P1	humanlincRNA1257	0.000895	2.1752374	up
ASMM9PARTA046107	AK039957	0.016099673	2.4726522	up
ASMM9PARTA010032	Gm5301	0.01462416	3.6166127	up
ASMM9PARTA002609		0.010847926	2.2261055	up
ASMM9PARTA015960	9430065F17Rik	0.005798801	3.8265884	up
ASMM9PARTA015960	9430065F17Rik	0.005798801	3.8265884	up
ASMM9PARTA015440	1010001N08Rik	0.038398307	2.7971175	up
ASMM9PARTA011704	Gm13888	0.001015393	2.811255	up
ASMM9PARTA051582	AK019838	0.000227	2.294279	up
MM9LINCRNAEXON10406-_P1	mouselincRNA1260	0.005678954	2.2176535	up
ASMM9PARTA017130	Trim30e-ps1	0.000287	3.6224866	up
ASMM9PARTA011738	Gm15470	0.000173	2.490983	up
ASMM9PARTA016451	2810008D09Rik	0.000000176	2.7259521	up

C80954_P1	humanlincRNA2399	0.003684981	2.344546	up
ASMM9PARTA011242	Gm12230	0.0000993	2.113646	up
ASMM9PARTA011242	Gm12230	0.0000993	2.113646	up
ASMM9PARTA011242	Gm12230	0.0000993	2.113646	up
ASMM9PARTA006207		0.040964875	2.3859847	up
ASMM9PARTA048537	NR_002853	0.000443	2.4265592	up
BI107540_P1	mouselincRNA1064	0.003572188	2.9005456	up
ASMM9PARTA016905	2900053O20Rik	0.000701	2.3093138	up
ASMM9PARTA000492		0.00163919	3.171052	up
CUST_139_PI426073487	uc.141	0.010394	2.5208695	up
ASMM9PARTA050019	AK148461	0.007578794	2.5351596	up
ASMM9PARTA005495		0.002343658	2.558607	up
ASMM9PARTA007417		0.002312835	3.6152418	up
ASMM9PARTA013190	Gm7386	0.003171985	4.6355166	up
ASMM9PARTA011381	Mup-ps14	0.0000522	5.1827497	up
ASMM9PARTA013040	Gm13577	0.0000595	2.7623637	up
ASMM9PARTA048165	Rabl2a	0.003768752	2.053208	up
ASMM9PARTA003806		0.00000252	3.0298662	up
ASMM9PARTA003377		0.003897649	3.1048882	up
MM9LINCRNAEXON10455-_P1	mouselincRNA1287	0.0231683	2.5350296	up
CUST_184_PI426409190		0.021069337	3.335752	up
ASMM9PARTA003640		0.040449712	2.539668	up
ASMM9PARTA002033		0.009132157	2.2649715	up
ASMM9PARTA010114	Gm11041	0.03261728	2.651793	up
ASMM9PARTA010303	Mup-ps4	0.0000907	14.846834	up
ASMM9PARTA011188	Hmgb1-ps4	0.0000992	2.7126071	up
ASMM9PARTA008507		0.026753385	2.4017262	up
ASMM9PARTA014823	A230056P14Rik	0.0000224	2.757427	up
ASMM9PARTA014823	A230056P14Rik	0.0000224	2.757427	up
ASMM9PARTA018579	AC159633.1	0.0000132	2.825753	up
ASMM9PARTA005922		0.006871192	4.436601	up
ASMM9PARTA015378	C920006O11Rik	0.000426	2.0906298	up
ASMM9PARTA013980	2810403D21Rik	0.007480211	3.1815767	up
ASMM9PARTA015335	B230314M03Rik	0.012495741	3.6262882	up
ASMM9PARTA004370		0.000578	2.4249277	up
MM9LINCRNAEXON11226-_P1	mouselincRNA0717	0.010145209	2.037533	up
ASMM9PARTA046618	AK084560	0.025179215	2.0396233	up
MM9LINCRNAEXON11010-_P1	mouselincRNA0889	0.027233997	2.227396	up
mouselincRNA0491-_P1	mouselincRNA0491	0.008828104	2.0486982	up
ASMM9PARTA015039	Gm15462	0.004822339	2.4003394	up
ASMM9PARTA018383	Gm16551	0.00000147	2.4987392	up
ASMM9PARTA018240	7SK.318	0.00000644	2.9303522	up
MM9LINCRNAEXON10775+_P1	mouselincRNA1143	0.003697956	3.6121304	up
ASMM9PARTA051179	AK162599	0.0000507	3.1958008	up
ASMM9PARTA047767	AK043130	0.00000375	2.4398158	up
ASMM9PARTA047767	AK043130	0.00000375	2.4398158	up
ASMM9PARTA010173	Gm7146	0.04551591	2.176919	up
ASMM9PARTA008433		0.0000179	3.665015	up
ASMM9PARTA044951	1700086O06Rik	0.000482	3.9681792	up

CUST_322_PI426073487	uc.324	0.019943956	2.7071564	up
CUST_322_PI426073487	uc.324	0.019943956	2.7071564	up
CA749839_P1	humanlincRNA1455	0.01808123	4.0205665	up
ASMM9PARTA005626		0.000494	2.4046261	up
ASMM9PARTA046629	AK020767	0.015835892	2.3768265	up
ASMM9PARTA013524	Gm16407	0.002360373	19.78195	up
ASMM9PARTA006409		0.0000904	2.274538	up
ASMM9PARTA047151	MAIR-IIa	0.001320717	3.3561897	up
ASMM9PARTA012161	Gm13239	0.0000693	2.5783002	up
ASMM9PARTA019355	RP23-141L18.6	0.000612	2.120292	up
ASMM9PARTA016604	9530034E10Rik	0.005028	2.0953546	up
ASMM9PARTA016604	9530034E10Rik	0.005028	2.0953546	up
MM9LINCRNAEXON10991+_P1		0.001675298	2.293769	up
ASMM9PARTA013682	Gm15886	0.04022039	2.3559144	up
ASMM9PARTA046040	4931406H21Rik	0.003887632	2.6124833	up
ASMM9PARTA010442	Gm10599	0.000000179	13.688693	up
ASMM9PARTA051038	BC065393	0.0000718	2.7037144	up
ASMM9PARTA051038	BC065393	0.0000718	2.7037144	up
ASMM9PARTA051038	BC065393	0.0000718	2.7037144	up
BY365805_P1	humanlincRNA1892	0.002442787	2.1303322	up
ASMM9PARTA019050	RP24-252L3.2	0.00000167	2.5991938	up
ASMM9PARTA014539	Gm16095	0.010736348	2.3576593	up
ASMM9PARTA005761		0.000382	2.3918576	up
CUST_237_PI426073487	uc.239	0.001489799	2.2062602	up
mouselincRNA0953+_P1	mouselincRNA0953	0.015228151	2.0030901	up
ASMM9PARTA011894	Gm14593	0.000402	2.3081677	up
ASMM9PARTA050563	AK039589	0.017318878	3.5861382	up
MM9LINCRNAEXON11384+_P1	mouselincRNA0595	0.031554114	2.3597822	up
ASMM9PARTA001301		0.02248129	2.456022	up
CUST_909_PI426073487	uc.430	0.0000187	22.395819	up
CUST_909_PI426073487	uc.430	0.0000187	22.395819	up
CUST_909_PI426073487	uc.430	0.0000187	22.395819	up
CUST_909_PI426073487	uc.430	0.0000187	22.395819	up
CUST_909_PI426073487	uc.430	0.0000187	22.395819	up
ASMM9PARTA007338		0.002050941	2.5473962	up
ASMM9PARTA015671	Gm13546	0.023608143	2.4103231	up
MM9LINCRNAEXON11327+_P1	mouselincRNA0531	0.032593045	2.2561057	up
ASMM9PARTA005615		0.009516534	2.9427543	up
CN672059_P1	humanlincRNA2025	0.0000922	2.4198492	up
MM9LINCRNAEXON11092+_P1	mouselincRNA0819	0.002204432	2.0520997	up
ASMM9PARTA002874		2.43E-08	48.727898	up
ASMM9PARTA003274		0.000491	2.0557833	up
ASMM9PARTA018348	Gm15717	0.001811937	2.3001482	up
ASMM9PARTA006865		0.000116	6.070025	up
ASMM9PARTA004660		0.00134421	2.2092893	up
ASMM9PARTA045220	Tmem181c-ps	0.041677628	2.1093931	up
ASMM9PARTA009067		0.000263	2.0018008	up
ASMM9PARTA005238		0.00000146	5.201726	up
ASMM9PARTA010309	Gm3371	0.000185	3.9100728	up

ASMM9PARTA012864	Gm9670	0.000165	2.4267838	up
ASMM9PARTA015594	D430001F17Rik	0.001629931	2.1788428	up
ASMM9PARTA011300	Gm9078	0.0000268	2.1964676	up
ASMM9PARTA008851		0.000503	2.3063297	up
ASMM9PARTA019195	Mup-ps4	0.0000158	5.711015	up
CUST_580_PI426073487	uc.101	0.000115	2.40203	up
CUST_580_PI426073487	uc.101	0.000115	2.40203	up
ASMM9PARTA003874		0.0000244	2.2392695	up
ASMM9PARTA045847	AK076931	0.020919668	2.692896	up
ASMM9PARTA009113	3010003L21Rik	0.020108035	2.722407	up
ASMM9PARTA017692	Gm7819	0.0000242	2.9961915	up
MM9LINCRNAEXON10291-_P1	mouselincRNA1500	0.003203733	2.404002	up
ASMM9PARTA005100		0.042936664	3.25303	up
ASMM9PARTA009651	Gm11270	0.0000691	2.6335354	up
ASMM9PARTA046659	4933439F18Rik	0.000433	3.868869	up
ASMM9PARTA046659	4933439F18Rik	0.000433	3.868869	up
ASMM9PARTA049376	Tpd52l2	0.049359888	3.007286	up
MM9LINCRNAEXON11316+_P1	mouselincRNA0662	0.001220781	5.289778	up
ASMM9PARTA006359		0.00000169	17.210634	up
ASMM9PARTA048810	AK042686	0.016800616	2.2249417	up
ASMM9PARTA005198		0.000112	2.312427	up
ASMM9PARTA001166		0.000379	2.2282903	up
ASMM9PARTA016490	Gm15850	0.034122057	2.1715176	up
ASMM9PARTA014438	Pisd-ps2	0.0000848	2.8530047	up
ASMM9PARTA006821		0.000245	2.1837595	up
ASMM9PARTA013474	Gm1947	0.00249382	2.239121	up
ASMM9PARTA050923	AK140288	0.00027	2.330448	up
ASMM9PARTA050923	AK140288	0.00027	2.330448	up
ASMM9PARTA003714		0.00000904	3.1356792	up
ASMM9PARTA048887	BC049717	0.000949	2.0402954	up
CUST_72_PI426073487	uc.74	0.04289264	2.6336536	up
ASMM9PARTA006224		0.002643993	2.2698817	up
MM9LINCRNAEXON10990-_P1		0.000217	2.0491824	up
ASMM9PARTA002107		0.010282259	2.486463	up
ASMM9PARTA045174	F730043M19Rik	0.0000109	2.0907702	up
ASMM9PARTA011834	Gm13013	0.000197	2.7611177	up
ASMM9PARTA003242		0.000013	5.450424	up
ASMM9PARTA007725		0.001178287	2.1477537	up
ASMM9PARTA010226	Gm5859	0.000232	3.999183	up
ASMM9PARTA018880	RP24-494O2.2	0.002038356	2.7605364	up
ASMM9PARTA011084	Gm11212	0.001707489	7.1417847	up
ASMM9PARTA003922		0.002017855	2.0481236	up
ASMM9PARTA003922		0.002017855	2.0481236	up
ASMM9PARTA003922		0.002017855	2.0481236	up
ASMM9PARTA003922		0.002017855	2.0481236	up
ASMM9PARTA003546		0.001226021	5.201623	up
ASMM9PARTA050374	C130026L21Rik	0.000012	2.4183884	up
ASMM9PARTA049104	Caper	0.000114	2.2018623	up
ASMM9PARTA048951	AK017239	0.021493174	2.3300061	up

ASMM9PARTA006532		0.000752	2.1807957	up
ASMM9PARTA009763	Gm10599	0.0000116	3.7107363	up
ASMM9PARTA049106	AK048878	0.043503724	2.885898	up
ASMM9PARTA004587		0.003764876	3.582118	up
ASMM9PARTA014282	Gm15991	0.002124584	3.5229528	up
ASMM9PARTA002934		0.000493	2.1732612	up
ASMM9PARTA045544	4933409K07Rik	0.00027	2.7417903	up
ASMM9PARTA015617	Gm15133	0.018541634	3.162094	up
ASMM9PARTA049885	ADAM22	0.049733885	3.426075	up
ASMM9PARTA049885	ADAM22	0.049733885	3.426075	up
ASMM9PARTA049885	ADAM22	0.049733885	3.426075	up
ASMM9PARTA008175		0.009049292	3.0822744	up
ASMM9PARTA005146		0.003144833	2.3215868	up
ASMM9PARTA001618		0.000106	2.693052	up
ASMM9PARTA012178	Mup-ps13	0.00000369	5.597641	up
MM9LINCRNAEXON10071-_P1	mouselincRNA1506	0.010094375	4.4578896	up
ASMM9PARTA013664	Gm7840	0.001021861	2.5210145	up
MM9LINCRNAEXON11571+_P1	mouselincRNA0391	0.000878	3.9741454	up
ASMM9PARTA011562	Gm15039	0.008076821	2.3185952	up
ASMM9PARTA046079	5430416N02Rik	0.0000735	3.6734188	up
mouselincRNA0327+_P1	mouselincRNA0327	0.002574732	2.9376884	up
MM9LINCRNAEXON10848+_P1	mouselincRNA0988	0.005376498	4.290399	up
ASMM9PARTA004819		0.000032	2.6319525	up
ASMM9PARTA019265	RP23-48M16.7	0.002186676	2.5394754	up
ASMM9PARTA013391	Gm9153	0.000326	2.1752706	up
ASMM9PARTA001271		0.04103195	3.6561942	up
ASMM9PARTA004768		0.002682594	2.5968246	up
ASMM9PARTA006391		0.040181704	2.3498719	up
MM9LINCRNAEXON11855+_P1		0.01677595	2.2109182	up
ASMM9PARTA008119		0.018987644	2.10819	up
ASMM9PARTA008119		0.018987644	2.10819	up
ASMM9PARTA008119		0.018987644	2.10819	up
ASMM9PARTA051336	AK020190	0.000000262	12.0142975	up
ASMM9PARTA008441		0.000295	3.965789	up
ASMM9PARTA008441		0.000295	3.965789	up
ASMM9PARTA049182	AK196015	0.013071364	2.4283123	up
ASMM9PARTA049182	AK196015	0.013071364	2.4283123	up
humanlincRNA1148-_P1	humanlincRNA1148	0.03532807	2.584566	up
ASMM9PARTA005106		0.02291107	3.844378	up
ASMM9PARTA005106		0.02291107	3.844378	up
ASMM9PARTA005643		0.01246422	2.6930146	up
ASMM9PARTA005643		0.01246422	2.6930146	up
MM9LINCRNAEXON11076-_P1		0.0000284	4.9582014	up
ASMM9PARTA016156	Gm12023	0.018250003	2.0630453	up
humanlincRNA1751+_P1	humanlincRNA1751	0.0000187	3.9547136	up
MM9LINCRNAEXON11077-_P1		0.00234579	3.0199275	up
ASMM9PARTA010539	Gm12165	0.001406168	2.3956766	up
ASMM9PARTA009943	Gm8062	0.00000126	3.7414384	up
ASMM9PARTA009158		0.000156	4.5355268	up

ASMM9PARTA017775	1700080N15Rik	0.03840659	2.431088	up
ASMM9PARTA011960	Gm11704	0.0000191	3.0662272	up
ASMM9PARTA011393	Gm11199	0.002621835	2.0320184	up
ASMM9PARTA012289	Gm14442	0.001934686	2.2335358	up
ASMM9PARTA012289	Gm14442	0.001934686	2.2335358	up
ASMM9PARTA047903	AK039376	0.013849491	5.160548	up
BY321897_P1	mouselincRNA1398	0.049968	2.2319455	up
ASMM9PARTA051767	AK042998	0.000649	2.2017314	up
ASMM9PARTA008423		0.001317778	2.607696	up
ASMM9PARTA004278		0.01337667	2.2351344	up
ASMM9PARTA047854	AK040737	0.001505451	2.720816	up
ASMM9PARTA007358		0.00017	2.2416778	up
ASMM9PARTA013273	Gm8178	0.000131	2.2250764	up
AI507368_P1	humanlincRNA2050	0.001653387	2.6645076	up
ASMM9PARTA007105		0.040792417	2.2196145	up
ASMM9PARTA049586	Il7	0.006104276	2.6041915	up
ASMM9PARTA049409	Tgm2	0.006833945	2.5027092	up
ASMM9PARTA007457		0.00000615	3.0624819	up
ASMM9PARTA016699	Gm11274	0.0000411	3.8522258	up
MM9LINCRNAEXON10403-_P1	mouselincRNA1259	0.012309696	2.0408568	up
ASMM9PARTA004883		0.002041745	3.4197848	up
ASMM9PARTA004883		0.002041745	3.4197848	up
ASMM9PARTA001842		0.000295	4.713204	up
ASMM9PARTA007881		0.031380463	2.2529385	up
ASMM9PARTA011798	Gm14901	0.0000952	2.223135	up
ASMM9PARTA011798	Gm14901	0.0000952	2.223135	up
ASMM9PARTA012086	Gm12687	0.001364371	2.0141764	up
ASMM9PARTA002185		0.013646789	2.0110724	up
ASMM9PARTA002185		0.013646789	2.0110724	up
ASMM9PARTA002185		0.013646789	2.0110724	up
ASMM9PARTA008215		0.001599787	2.6774795	up
MM9LINCRNAEXON12111-_P1	mouselincRNA0122	0.000000188	8.041722	up
ASMM9PARTA019467	RP24-318H2.3	0.0241083	3.6438258	up
ASMM9PARTA008826		0.003225998	2.0662498	up
CUST_171_PI426073487	uc.173	0.0000837	2.7726395	up
ASMM9PARTA010185	Gm12568	0.00000273	2.981855	up
ASMM9PARTA046838	AK138505	0.000981	2.5629282	up
ASMM9PARTA046838	AK138505	0.000981	2.5629282	up
ASMM9PARTA046838	AK138505	0.000981	2.5629282	up
ASMM9PARTA000820		0.0000915	16.098686	up
ASMM9PARTA000820		0.0000915	16.098686	up
MM9LINCRNAEXON11562-_P1	mouselincRNA0381	0.010353497	2.0829291	up
CUST_407_PI426073487	uc.409	0.000719	2.3269606	up
humanlincRNA0518+_P1	humanlincRNA0518	0.0000811	3.7236433	up
ASMM9PARTA015498	AI451557	0.000668	2.0574825	up
EL964962_P1	mouselincRNA0001	0.003955408	4.071823	up
humanlincRNA1546-_P1	humanlincRNA1546	0.010746875	2.9871545	up
ASMM9PARTA016744	Gm10863	0.047607485	2.5047936	up
ASMM9PARTA002361		0.0000165	2.0532448	up

AK145365_P1	mouselincRNA1288	0.002516194	3.7512045	up
ASMM9PARTA018655	Gm10075	0.0000143	2.9120884	up
ASMM9PARTA050637	smarp	0.001593024	2.5456998	up
ASMM9PARTA012744	Gm15693	0.000893	2.2510507	up
BY725911_P1	mouselincRNA0668	0.044335973	2.0044153	up
ASMM9PARTA018588	Gm16118	0.000000941	3.7365682	up
ASMM9PARTA006159		0.009355998	3.3225546	up
ASMM9PARTA005665		0.000472	2.183836	up
ASMM9PARTA013927	Gm16002	0.00000463	6.3231006	up
ASMM9PARTA017658	Gm13790	0.042296667	2.1728554	up
ASMM9PARTA016645	Gm14090	0.032816384	2.0021355	up
ASMM9PARTA000801		0.00017	3.4467328	up
ASMM9PARTA050305	AK031300	0.016717825	2.3399742	up
ASMM9PARTA004715		0.000000149	7.717302	up
ASMM9PARTA045616	Serpina3h	0.000614	2.7422664	up
BX636194_P1	mouselincRNA1032	0.013654836	2.5980556	up
ASMM9PARTA005646		0.00820253	2.0123851	up
ASMM9PARTA005646		0.00820253	2.0123851	up
ASMM9PARTA007593		0.005125153	3.678817	up
ASMM9PARTA046706	AK082467	0.000000602	7.714457	up
ASMM9PARTA045201	Gm10565	0.0379293	2.8846593	up
ASMM9PARTA050227	BC028660	0.024266167	3.024105	up
ASMM9PARTA017822	7SK.135	0.000748	3.1937835	up
ASMM9PARTA045165	Gm8234	0.0000421	2.4578252	up
ASMM9PARTA049900	1700028K03Rik	0.001609577	2.0009012	up
ASMM9PARTA049900	1700028K03Rik	0.001609577	2.0009012	up
ASMM9PARTA049900	1700028K03Rik	0.001609577	2.0009012	up
ASMM9PARTA049900	1700028K03Rik	0.001609577	2.0009012	up
ASMM9PARTA049900	1700028K03Rik	0.001609577	2.0009012	up
ASMM9PARTA049900	1700028K03Rik	0.001609577	2.0009012	up
ASMM9PARTA049900	1700028K03Rik	0.001609577	2.0009012	up
MM9LINCRNAEXON12089+_P1	mouselincRNA0102	0.022283986	2.5649867	up
ASMM9PARTA050899	AK166824	0.001238364	2.4209611	up
ASMM9PARTA006971		0.037790198	3.2771153	up
MM9LINCRNAEXON12056-_P1	mouselincRNA0073	0.000368	6.711888	up
ASMM9PARTA017493	Gm15692	0.0000662	2.5697474	up
ASMM9PARTA017493	Gm15692	0.0000662	2.5697474	up
ASMM9PARTA013963	6030471H07Rik	0.026871068	2.4950523	up
MM9LINCRNAEXON11755+_P1		0.0000778	2.4311874	up
BI108441_P1	mouselincRNA0637	0.002089665	3.0207336	up
BI108441_P1	mouselincRNA0637	0.002089665	3.0207336	up
BI108441_P1	mouselincRNA0637	0.002089665	3.0207336	up
BI108441_P1	mouselincRNA0637	0.002089665	3.0207336	up
BI108441_P1	mouselincRNA0637	0.002089665	3.0207336	up
BI108441_P1	mouselincRNA0637	0.002089665	3.0207336	up
BI108441_P1	mouselincRNA0637	0.002089665	3.0207336	up
BI108441_P1	mouselincRNA0637	0.002089665	3.0207336	up
BI108441_P1	mouselincRNA0637	0.002089665	3.0207336	up
CUST_173_PI426409190		0.0000198	3.7454646	up

ASMM9PARTA007295		0.03300677	2.223998	up
ASMM9PARTA002895		0.0000664	3.036509	up
MM9LINCRNAEXON11730-_P1		0.000000323	21.458044	up
CK379655_P1	humanlincRNA1585	0.04898197	2.627792	up
ASMM9PARTA016945	A230056P14Rik	0.0000145	3.817645	up
ASMM9PARTA000137		0.0000181	7.1219735	up
MM9LINCRNAEXON11978+_P1	mouselincRNA0222	0.003712853	2.563598	up
MM9LINCRNAEXON11855-_P1		0.04749484	3.527621	up
MM9LINCRNAEXON11855-_P1		0.04749484	3.527621	up
MM9LINCRNAEXON11855-_P1		0.04749484	3.527621	up
ASMM9PARTA008934		0.015762908	2.701945	up
ASMM9PARTA017917	Gm16143	0.0000255	4.7346373	up
ASMM9PARTA001656		0.012663266	2.0601208	up
MM9LINCRNAEXON10903-_P1		0.000474	2.6986115	up
ASMM9PARTA012091	Gm12262	0.011555783	4.373014	up
ASMM9PARTA046504	Sfi1	0.04870597	2.2429795	up
ASMM9PARTA008616		0.037262544	2.7010329	up
ASMM9PARTA013032	Gm5383	0.001402283	2.0569284	up
ASMM9PARTA015147	Gm8817	0.03249829	2.200502	up
humanlincRNA0574+_P1	humanlincRNA0574	0.017278384	2.6009798	up
ASMM9PARTA009783	4932415M13Rik	0.000425	2.7867901	up
ASMM9PARTA008094		0.021004593	2.81872	up
MM9LINCRNAEXON11219+_P1	mouselincRNA0701	0.004055457	2.1044128	up
ASMM9PARTA047312	AK036683	0.008652229	3.2410846	up
ASMM9PARTA009446	Mageb16-ps2	0.011625608	2.0846617	up
ASMM9PARTA009446	Mageb16-ps2	0.011625608	2.0846617	up
ASMM9PARTA008266		0.03052738	2.151655	up
ASMM9PARTA012816	Gm12006	0.017849	3.1609411	up
ASMM9PARTA012761	Gm15799	0.000206	2.267382	up
ASMM9PARTA012202	Gm5942	0.000996	2.1706877	up
ASMM9PARTA012112	Gm14739	0.031601164	2.220677	up
ASMM9PARTA051219	AK029875	0.000145	3.027687	up
ASMM9PARTA015905	Gm13056	0.000367	6.6384277	up
ASMM9PARTA017308	Gm12472	0.03028674	2.3060696	up
ASMM9PARTA011898	Mup-ps16	0.0000223	2.4326117	up
ASMM9PARTA045091	Snhg3	0.00037	2.0472598	up
ASMM9PARTA051534	AK010793	0.004530279	6.0006886	up
ASMM9PARTA010111	Gm10977	0.0000453	2.647849	up
ASMM9PARTA006234		0.009313879	2.068487	up
ASMM9PARTA012332	Mup-ps21	0.000601	3.2815022	up
ASMM9PARTA011325	Gm12351	0.00076	2.2448652	up
ASMM9PARTA012600	Gm7966	0.0000248	2.6037138	up
ASMM9PARTA006616		0.001158617	2.5371776	up
ASMM9PARTA047369	AK006031	0.002195611	2.3695242	up
ASMM9PARTA016820	AW495222	0.0000794	2.2110105	up
CUST_600_PI426073487	uc.121	0.008126531	4.497435	up
ASMM9PARTA050294	AK035387	0.006486616	2.4051902	up
ASMM9PARTA016823	BC037032	0.02000702	2.1161623	up
ASMM9PARTA008377		0.000531	2.343185	up

ASMM9PARTA016400	Gm12204	0.003325343	3.2655756	up
ASMM9PARTA006996		0.014006976	3.6733685	up
ASMM9PARTA007841		0.001121077	2.401625	up
ASMM9PARTA012724	Gm11307	0.001982323	2.0375216	up
ASMM9PARTA008655		0.023679594	2.3027277	up
ASMM9PARTA008655		0.023679594	2.3027277	up
ASMM9PARTA008655		0.023679594	2.3027277	up
ASMM9PARTA045692	Gm3893	0.0000145	23.297878	up
ASMM9PARTA045692	Gm3893	0.0000145	23.297878	up
ASMM9PARTA045692	Gm3893	0.0000145	23.297878	up
ASMM9PARTA045692	Gm3893	0.0000145	23.297878	up
ASMM9PARTA045692	Gm3893	0.0000145	23.297878	up
ASMM9PARTA045692	Gm3893	0.0000145	23.297878	up
ASMM9PARTA045692	Gm3893	0.0000145	23.297878	up
ASMM9PARTA045692	Gm3893	0.0000145	23.297878	up
ASMM9PARTA045692	Gm3893	0.0000145	23.297878	up
ASMM9PARTA045692	Gm3893	0.0000145	23.297878	up
ASMM9PARTA045692	Gm3893	0.0000145	23.297878	up
ASMM9PARTA045692	Gm3893	0.0000145	23.297878	up
ASMM9PARTA045692	Gm3893	0.0000145	23.297878	up
ASMM9PARTA045692	Gm3893	0.0000145	23.297878	up
ASMM9PARTA045692	Gm3893	0.0000145	23.297878	up
ASMM9PARTA045692	Gm3893	0.0000145	23.297878	up
ASMM9PARTA045692	Gm3893	0.0000145	23.297878	up
ASMM9PARTA045692	Gm3893	0.0000145	23.297878	up
ASMM9PARTA045692	Gm3893	0.0000145	23.297878	up
ASMM9PARTA045692	Gm3893	0.0000145	23.297878	up
ASMM9PARTA045692	Gm3893	0.0000145	23.297878	up
ASMM9PARTA045692	Gm3893	0.0000145	23.297878	up
MM9LINCRNAEXON11831+_P1		0.0000261	2.402831	up
ASMM9PARTA012969	Mup-ps20	0.000206	6.7438145	up
ASMM9PARTA001927		0.000115	2.134282	up
ASMM9PARTA045146	3110070M22Rik	0.000202	5.618087	up
ASMM9PARTA045146	3110070M22Rik	0.000202	5.618087	up
ASMM9PARTA013439	Gm15545	0.027878832	2.5476606	up
ASMM9PARTA001481		0.001773122	4.7225776	up
MM9LINCRNAEXON11830+_P1	mouselincRNA0272	0.012886777	2.2233913	up
ASMM9PARTA012145	Gm11830	0.000018	3.6044834	up
ASMM9PARTA047713	AK166895	0.000122	2.4677193	up
ASMM9PARTA007373		0.000142	2.0476632	up
AV570450_P1	humanlincRNA0329	0.0000147	2.3798816	up
ASMM9PARTA006076		0.000946	2.6552072	up
ASMM9PARTA004840		0.0000211	2.0126963	up
ASMM9PARTA016906	Gm15865	0.006465207	4.2304425	up
ASMM9PARTA046895	slc43a2	0.011298595	2.4693172	up
ASMM9PARTA046895	slc43a2	0.011298595	2.4693172	up
ASMM9PARTA046895	slc43a2	0.011298595	2.4693172	up
ASMM9PARTA005435		6.32E-08	19.430582	up
ASMM9PARTA048214	AK170928	0.000111	5.404855	up

ASMM9PARTA048214	AK170928	0.000111	5.404855	up
ASMM9PARTA048214	AK170928	0.000111	5.404855	up
ASMM9PARTA007740		0.0000155	2.8659556	up
ASMM9PARTA010117	Olfr833-ps1	0.025492877	2.1618166	up
ASMM9PARTA006854		0.001526317	3.472981	up
ASMM9PARTA013449	Gm15052	0.02267584	2.7453294	up
ASMM9PARTA003577		0.04269406	2.631562	up
ASMM9PARTA050677	AK085609	0.041577633	2.2467873	up
ASMM9PARTA012852	Gm14300	0.010992006	2.01897	up
ASMM9PARTA002799		0.000741	2.1187544	up
MM9LINCRNAEXON11234-_P1	mouselincRNA0719	0.025463864	2.4796512	up
ASMM9PARTA003518		0.0000173	3.9191155	up
ASMM9PARTA011435	Gm11877	0.000602	2.0179665	up
ASMM9PARTA015623	Gm11368	0.03503963	2.767399	up
CUST_72_PI426409190	Gm13155	0.00000106	2.655761	up
ASMM9PARTA019336	AC132234.1	0.0000056	2.7873704	up
ASMM9PARTA016885	Gm14233	0.014509914	2.607557	up
ASMM9PARTA016885	Gm14233	0.014509914	2.607557	up
ASMM9PARTA015663	Gm12462	0.021645095	3.114849	up
ASMM9PARTA011869	Gm16106	0.0000682	2.3384438	up
ASMM9PARTA008845		0.043120634	2.8098152	up
ASMM9PARTA018622	Gm16110	0.045055512	2.533396	up
ASMM9PARTA012180	Mup-ps17	0.000424	3.6852767	up
ASMM9PARTA001716		0.0000146	3.3201692	up
ASMM9PARTA006533		0.008149483	2.22305	up
ASMM9PARTA013831	Gm7375	0.008643639	3.4775705	up
ASMM9PARTA019160	RP24-230H12.5	0.0000176	3.2998517	up
ASMM9PARTA014536	Gm11532	0.004175172	2.7584414	up
mouselincRNA1486-_P1	mouselincRNA1486	0.026596203	2.349201	up
ASMM9PARTA008097		0.000362	2.104554	up
ASMM9PARTA008097		0.000362	2.104554	up
ASMM9PARTA003674		0.03726476	2.1515129	up
ASMM9PARTA015858	Gm13194	0.000167	2.112241	up
MM9LINCRNAEXON11454-_P1	mouselincRNA0481	0.010671058	3.3564527	up
ASMM9PARTA048947	Strbp	0.008610019	2.3662748	up
ASMM9PARTA048947	Strbp	0.008610019	2.3662748	up
ASMM9PARTA048947	Strbp	0.008610019	2.3662748	up
ASMM9PARTA048947	Strbp	0.008610019	2.3662748	up
MM9LINCRNAEXON10665-_P1	mouselincRNA1101	0.000109	3.4450989	up
ASMM9PARTA011989	Gm13170	0.0000269	2.4050152	up
ASMM9PARTA011989	Gm13170	0.0000269	2.4050152	up
ASMM9PARTA016026	Gm11861	0.00653889	2.2872052	up
MM9LINCRNAEXON11063+_P1	mouselincRNA0809	0.000421	4.7005215	up
ASMM9PARTA016956	Mup-ps4	0.00000119	11.556606	up
ASMM9PARTA016163	0610005C13Rik	0.000184	4.019154	up
ASMM9PARTA049374	Etohi1	0.0000182	2.2188861	up
ASMM9PARTA049374	Etohi1	0.0000182	2.2188861	up
ASMM9PARTA049374	Etohi1	0.0000182	2.2188861	up
ASMM9PARTA049374	Etohi1	0.0000182	2.2188861	up

ASMM9PARTA049374	Etohi1	0.0000182	2.2188861	up
ASMM9PARTA049374	Etohi1	0.0000182	2.2188861	up
ASMM9PARTA049374	Etohi1	0.0000182	2.2188861	up
ASMM9PARTA013034	Gm15379	0.002158355	2.4215639	up
ASMM9PARTA047583	AK021377	0.0000244	2.4268384	up
ASMM9PARTA047583	AK021377	0.0000244	2.4268384	up
ASMM9PARTA015476	Hoxa11as	0.0000486	2.723337	up
CK378914_P1	humanlincRNA1553	0.039537963	3.2691762	up
CUST_780_PI426073487	uc.301	0.029574085	2.6782415	up
ASMM9PARTA003754		0.00011	3.427992	up
ASMM9PARTA047727	AK013308	0.0000224	2.8708134	up
ASMM9PARTA001697		0.023436017	2.993111	up
ASMM9PARTA010314	Gm12643	0.000466	2.2831063	up
ASMM9PARTA012488	Gm11283	0.008885544	3.546874	up
ASMM9PARTA013211	Gm14983	0.0000664	2.5804033	up
ASMM9PARTA008101		0.00217741	2.4394176	up
ASMM9PARTA046977	Prpf39	0.007360946	2.2625773	up
ASMM9PARTA046977	Prpf39	0.007360946	2.2625773	up
ASMM9PARTA014892	5730405O15Rik	0.000131	2.2418652	up
ASMM9PARTA011821	Gm7219	0.00000905	2.0991886	up
ASMM9PARTA049866	AK052888	0.001548062	2.2319543	up
mouselincRNA0238-_P1	mouselincRNA0238	0.001346171	3.8611655	up
ASMM9PARTA004413		0.000814	2.0993032	up
ASMM9PARTA012701	AA413626	0.00369363	2.0243053	up
ASMM9PARTA050876	AK077481	0.022632372	2.0454216	up
ASMM9PARTA004788		0.0000122	27.265574	up
ASMM9PARTA011129	Gm12741	0.000112	2.0961394	up
ASMM9PARTA006722		0.01094285	2.8356895	up
ASMM9PARTA000388		0.000599	2.3450785	up
ASMM9PARTA015826	Gm15605	0.001950575	2.2574613	up
ASMM9PARTA015826	Gm15605	0.001950575	2.2574613	up
ASMM9PARTA010884	Gm15709	0.002347111	2.385834	up
ASMM9PARTA017044	BC024582	0.001999527	2.8319056	up
ASMM9PARTA005191		0.000000489	19.55925	up
ASMM9PARTA047793	MADP-1	0.000126	2.121295	up
ASMM9PARTA047793	MADP-1	0.000126	2.121295	up
ASMM9PARTA047793	MADP-1	0.000126	2.121295	up
ASMM9PARTA047793	MADP-1	0.000126	2.121295	up
ASMM9PARTA003039		0.0000954	2.2476687	up
MM9LINCRNAEXON10971-_P1		0.0000317	4.04571	up
ASMM9PARTA010668	Gm11905	0.024447275	3.2819102	up
MM9LINCRNAEXON11511+_P1	mouselincRNA0367	0.010972943	4.9441733	up
ASMM9PARTA014765	A530083I20Rik	0.003885604	6.2350335	up
AI503337_P1	mouselincRNA0302	0.015948238	2.035016	up
ASMM9PARTA013309	Gm14460	0.00023	2.424697	up
ASMM9PARTA001201		0.019467967	2.6579392	up
ASMM9PARTA044984	Dio3os	0.016049087	2.2251294	up
MM9LINCRNAEXON11077+_P1		0.00000442	3.0722895	up
ASMM9PARTA016368	Gm15958	0.014653345	2.0924673	up

ASMM9PARTA018263	Gm16570	0.000103	2.2948632	up
ASMM9PARTA000283		0.021953516	2.4303453	up
ASMM9PARTA015559	6720401G13Rik	0.025047805	2.0965323	up
ASMM9PARTA007775		0.010432979	2.8922331	up
ASMM9PARTA051077	AK215526	0.026387833	2.431819	up
ASMM9PARTA001427		0.003310577	3.8885329	up
ASMM9PARTA001427		0.003310577	3.8885329	up
ASMM9PARTA016737	Gm11683	0.039207943	2.1252909	up
ASMM9PARTA016737	Gm11683	0.039207943	2.1252909	up
ASMM9PARTA009332	4930463O16Rik	0.032151803	2.553776	up
ASMM9PARTA017363	Gm11837	0.0000179	2.3324692	up
ASMM9PARTA007597		0.000172	3.4272103	up
MM9LINCRNAEXON10811-_P1	mouselincRNA0961	0.044673372	2.1008406	up
ASMM9PARTA015804	Gm13189	0.007019602	3.6558638	up
ASMM9PARTA044808	Speer7-ps1	0.000416	2.006462	up
ASMM9PARTA048531	NR_003519	0.000141	2.4995687	up
ASMM9PARTA013479	Gm16479	0.000693	2.7296116	up
ASMM9PARTA018563	AC164629.6	0.009281689	2.561227	up
ASMM9PARTA015237	5330411J11Rik	0.006404722	2.828499	up
ASMM9PARTA046658	AK030920	0.000308	2.1788116	up
ASMM9PARTA046658	AK030920	0.000308	2.1788116	up
ASMM9PARTA049776	4933409K07Rik	0.0000754	2.578514	up
ASMM9PARTA047171	AK012841	0.0000144	2.765245	up
ASMM9PARTA047171	AK012841	0.0000144	2.765245	up
CUST_429_PI426073487	uc.431	0.037706435	2.4475033	up
ASMM9PARTA009678	Gm5135	0.028812699	5.794474	up
ASMM9PARTA001946		0.00000109	2.1609287	up
ASMM9PARTA006005		0.003909261	5.623851	up
ASMM9PARTA010979	Gm13458	0.002568956	2.0198169	up
MM9LINCRNAEXON11845+_P1	mouselincRNA0275	0.002253867	5.3943233	up
ASMM9PARTA006974		0.003849258	3.0626094	up
ASMM9PARTA015170	1700042G15Rik	0.04111711	2.9537787	up
ASMM9PARTA044965	Mug-ps1	0.018938622	2.169502	up
ASMM9PARTA004299		0.01829673	2.5046062	up
ASMM9PARTA016541	Zfp133-ps	0.009836554	2.673169	up
ASMM9PARTA048168	AK157795	0.0000922	2.906104	up
ASMM9PARTA048168	AK157795	0.0000922	2.906104	up
ASMM9PARTA003726		0.029743275	2.9296398	up
ASMM9PARTA018124	Gm3470	0.000964	2.5198631	up
ASMM9PARTA015212	Gm16548	0.025674216	3.1395323	up
ASMM9PARTA015212	Gm16548	0.025674216	3.1395323	up
ASMM9PARTA016822	Gm14747	0.000949	2.7512255	up
ASMM9PARTA010177	Gm6973	0.000857	4.095665	up
ASMM9PARTA017321	5330434G04Rik	0.005595785	2.0989368	up
ASMM9PARTA002192		0.000526	2.3362405	up
ASMM9PARTA007514		0.000224	2.1863723	up
ASMM9PARTA018967	AC159205.1	0.002297188	2.7751563	up
ASMM9PARTA004773		0.0000678	3.3029537	up
ASMM9PARTA048470	BC096411	0.017798696	2.4458632	up

DV650143_P1	mouselincRNA1440	0.004753044	2.2532213	up
ASMM9PARTA013751	Mup-ps8	0.00006	8.858889	up
ASMM9PARTA048046	BC098222	0.006436714	2.3191826	up
ASMM9PARTA003818		0.019369354	2.469514	up
ASMM9PARTA017664	4921508A21Rik	0.018799689	2.4309752	up
ASMM9PARTA014507	Gm15941	0.019537766	2.440631	up
ASMM9PARTA014507	Gm15941	0.019537766	2.440631	up
ASMM9PARTA014507	Gm15941	0.019537766	2.440631	up
ASMM9PARTA014507	Gm15941	0.019537766	2.440631	up
mouselincRNA0365-_P1	mouselincRNA0365	0.014068429	3.0238342	up
ASMM9PARTA006236		0.003242792	2.4778924	up
ASMM9PARTA046159	Ankrd11	0.016949203	2.2631643	up
ASMM9PARTA045479	Gm13629	0.000178	2.8826463	up
ASMM9PARTA045479	Gm13629	0.000178	2.8826463	up
MM9LINCRNAEXON11640+_P1	mouselincRNA0450	0.005615351	2.586318	up
ASMM9PARTA009282		0.000456	4.7056136	up
ASMM9PARTA000539		0.000000221	5.009665	up
ASMM9PARTA010158	Gm5532	0.000214	2.0082514	up
ASMM9PARTA015391	C030037D09Rik	0.000000605	17.239882	up
ASMM9PARTA015391	C030037D09Rik	0.000000605	17.239882	up
MM9LINCRNAEXON10854-_P1	mouselincRNA1008	0.045372102	2.189518	up
ASMM9PARTA012588	Gm15224	0.0000362	2.48759	up
ASMM9PARTA050500	AK044653	0.0000138	2.0939777	up
ASMM9PARTA017687	4930522O17Rik	0.003809092	2.0728438	up
ASMM9PARTA010353	Gm14886	0.025164222	4.0286784	up
MM9LINCRNAEXON11156-_P1		0.007178091	2.4582517	up
MM9LINCRNAEXON10120-_P1	mouselincRNA1557	0.029470466	2.294069	up
ASMM9PARTA007824		0.000135	5.0431075	up
ASMM9PARTA007824		0.000135	5.0431075	up
ASMM9PARTA007824		0.000135	5.0431075	up
ASMM9PARTA007824		0.000135	5.0431075	up
ASMM9PARTA006592		0.00000802	2.1025412	up
ASMM9PARTA005318		0.005187639	2.2597253	up
ASMM9PARTA006561		0.000208	3.0838203	up
ASMM9PARTA012053	Gm11469	0.000111	2.0296926	up
ASMM9PARTA013708	Gm13591	0.000228	2.8590877	up
ASMM9PARTA013708	Gm13591	0.000228	2.8590877	up
ASMM9PARTA013708	Gm13591	0.000228	2.8590877	up
ASMM9PARTA049271	BC085231	0.0000535	2.3479714	up
ASMM9PARTA010524	Gm15161	0.023131208	2.8052366	up
ASMM9PARTA049447	Cetn4	0.00011	2.357526	up
ASMM9PARTA017541	A230087F16Rik	0.0000138	14.316176	up
ASMM9PARTA003233		0.000822	2.0655158	up
ASMM9PARTA050716	AK036949	0.001851368	2.0126505	up
ASMM9PARTA012521	Gm12329	0.014312104	2.4452667	up
ASMM9PARTA006006		1.64E-08	15.571929	up
ASMM9PARTA004899		0.0000374	8.867676	up
MM9LINCRNAEXON10979-_P1	mouselincRNA0870	0.00144868	4.3862224	up
ASMM9PARTA001684		0.00000401	2.6117725	up

ASMM9PARTA017205	4930511M06Rik	0.008154828	2.2770941	up
ASMM9PARTA051308	AK038653	0.001074239	3.2058306	up
MM9LINCRNAEXON10035-_P1	mouselincRNA1646	0.001899102	2.2003012	up
ASMM9PARTA046090	AK079804	0.000139	2.1687758	up
MM9LINCRNAEXON12071-_P1	mouselincRNA0095	0.02435267	2.3835924	up
ASMM9PARTA051329	AK076318	0.00848008	2.6944587	up
humanlincRNA2106+_P1	humanlincRNA2106	0.016507933	2.11366	up
CUST_196_PI426073487	uc.198	0.001196752	2.2389858	up
ASMM9PARTA015795	3110053B16Rik	0.000304	17.447605	up
ASMM9PARTA019148	RP23-131B11.2	0.019360963	2.473327	up
ASMM9PARTA007351		0.044408884	2.2335773	up
ASMM9PARTA007351		0.044408884	2.2335773	up
ASMM9PARTA045799	Gm6607	0.000134	2.5401933	up
ASMM9PARTA000082		0.00000713	3.3611763	up
ASMM9PARTA000151		0.0000979	2.4393134	up
ASMM9PARTA004652		0.030589186	2.3324573	up
ASMM9PARTA019226	Gm16759	0.001250253	4.860738	up
ASMM9PARTA002317		0.019190772	4.3123827	up
MM9LINCRNAEXON10047+_P1	mouselincRNA1651	0.04755336	3.226252	up
ASMM9PARTA012440	Mup-ps9	0.0000544	5.0800457	up
ASMM9PARTA045611	Gm8989	0.0000122	2.1922016	up
ASMM9PARTA016310	Gm12868	0.003230158	3.039776	up
MM9LINCRNAEXON11616+_P1	mouselincRNA0430	0.03553728	5.394965	up
ASMM9PARTA046527	AK016383	0.00963778	3.4179327	up
ASMM9PARTA051686	Gprasp1	0.00000601	2.4669201	up
ASMM9PARTA051686	Gprasp1	0.00000601	2.4669201	up
ASMM9PARTA051686	Gprasp1	0.00000601	2.4669201	up
ASMM9PARTA051686	Gprasp1	0.00000601	2.4669201	up
ASMM9PARTA019822	AC152063.3	0.0000015	4.327241	up
ASMM9PARTA018077	Gas5	0.00000113	2.639842	up
ASMM9PARTA010736	Gm11380	0.000326	3.1712265	up
ASMM9PARTA011938	Hmgb1-ps1	0.0000165	2.4225085	up
ASMM9PARTA012466	Gm4992	0.040913377	2.4308543	up
ASMM9PARTA047127	ISG12a	0.000535	2.2497191	up
ASMM9PARTA047127	ISG12a	0.000535	2.2497191	up
ASMM9PARTA047127	ISG12a	0.000535	2.2497191	up
ASMM9PARTA047127	ISG12a	0.000535	2.2497191	up
ASMM9PARTA047127	ISG12a	0.000535	2.2497191	up
ASMM9PARTA012206	Mup-ps18	0.004230593	2.0759668	up
ASMM9PARTA049649	2210012G02Rik	0.005834688	3.7912464	up
ASMM9PARTA049649	2210012G02Rik	0.005834688	3.7912464	up
AA791803_P1	mouselincRNA0980	0.000158	4.209043	up
ASMM9PARTA003122		0.000017	4.562548	up
ASMM9PARTA049855	AK019690	0.0000574	2.2752028	up
ASMM9PARTA019366	AC137708.1	0.0000925	3.10873	up
ASMM9PARTA019568	RP23-97D14.2	0.001179426	3.5288062	up
MM9LINCRNAEXON11627-_P1		0.004710208	2.3805652	up
ASMM9PARTA050126	AK019631	0.00000754	3.6911464	up
ASMM9PARTA005934		0.011721495	2.0523357	up

ASMM9PARTA004622		0.016235739	2.7310739	up
ASMM9PARTA046236	A730008H23Rik	0.000412	6.9144487	up
ASMM9PARTA046236	A730008H23Rik	0.000412	6.9144487	up
ASMM9PARTA014967	6430628N08Rik	0.000497	2.2486057	up
ASMM9PARTA004138		0.001068032	2.472599	up
ASMM9PARTA011360	Gm14466	0.017476412	2.4162133	up
ASMM9PARTA004223		0.00000158	2.1865516	up
ASMM9PARTA002658		0.017831042	2.0186336	up
CK377992_P1	humanlincRNA1344	0.027192576	2.3351774	up
ASMM9PARTA002944		0.035084255	2.1090152	up
ASMM9PARTA002108		0.0000378	2.2845912	up
ASMM9PARTA049481	Etohi1	0.000243	2.1015198	up
ASMM9PARTA049481	Etohi1	0.000243	2.1015198	up
ASMM9PARTA049481	Etohi1	0.000243	2.1015198	up
ASMM9PARTA049481	Etohi1	0.000243	2.1015198	up
ASMM9PARTA049481	Etohi1	0.000243	2.1015198	up
ASMM9PARTA049481	Etohi1	0.000243	2.1015198	up
ASMM9PARTA049481	Etohi1	0.000243	2.1015198	up
ASMM9PARTA049481	Etohi1	0.000243	2.1015198	up
ASMM9PARTA049481	Etohi1	0.000243	2.1015198	up
ASMM9PARTA049481	Etohi1	0.000243	2.1015198	up
ASMM9PARTA049481	Etohi1	0.000243	2.1015198	up
ASMM9PARTA049481	Etohi1	0.000243	2.1015198	up
ASMM9PARTA009045		0.020251706	2.6574764	up
ASMM9PARTA044906	2410002O22Rik	0.00000316	4.917222	up
ASMM9PARTA044906	2410002O22Rik	0.00000316	4.917222	up
ASMM9PARTA044906	2410002O22Rik	0.00000316	4.917222	up
ASMM9PARTA044906	2410002O22Rik	0.00000316	4.917222	up
MM9LINCRNAEXON10048-_P1		0.005635168	2.594016	up
ASMM9PARTA002149		0.0000328	2.0522332	up
ASMM9PARTA002446		0.015533558	2.6028762	up
CUST_365_PI426073487	uc.367	0.0000141	2.2055771	up
ASMM9PARTA015029	Gm16233	0.000738	5.030052	up
ASMM9PARTA000260		0.003679409	2.5219154	up
ASMM9PARTA000260		0.003679409	2.5219154	up
ASMM9PARTA048510	AK161769	0.001542412	2.0662546	up
ASMM9PARTA049539	AK076677	0.0000201	2.4637682	up
ASMM9PARTA011651	Gm11881	0.000721	2.3493764	up
ASMM9PARTA012216	Gm13118	0.0000554	4.0120564	up
ASMM9PARTA009396	Gm16373	0.000826	2.080701	up
ASMM9PARTA045585	Gm839	0.021356562	2.228997	up
ASMM9PARTA016669	Gm16211	0.01741969	3.3444524	up
ASMM9PARTA051758	AK159035	0.001567319	11.803876	up
MM9LINCRNAEXON10124+_P1	mouselincRNA1558	0.001390586	2.6133294	up
ASMM9PARTA050616	AK049852	0.0000644	4.249902	up
ASMM9PARTA009548	Gm8556	0.0000274	3.4742818	up
ASMM9PARTA002598		0.000755	2.546513	up
ASMM9PARTA000813		0.001190985	2.1826582	up
ASMM9PARTA005122		0.000000645	4.7228146	up

CUST_946_PI426073487	uc.467	0.04952777	3.0639296	up
ASMM9PARTA016359	Gm7546	0.041623604	2.7077355	up
ASMM9PARTA013307	Sh3d2c-ps1	0.000058	2.0040734	up
ASMM9PARTA013288	Gm15877	0.003824441	2.5225005	up
ASMM9PARTA010960	Gm14987	0.032874588	2.4844484	up
ASMM9PARTA018523	Airn	0.000000039	30.692284	up
MM9LINCRNAEXON10694-_P1	mouselincRNA1113	0.011640612	2.0307622	up
ASMM9PARTA005165		0.014309723	3.6635392	up
ASMM9PARTA004351		0.0000214	2.6030142	up
ASMM9PARTA015549	A830036E02Rik	0.016751433	4.167666	up
ASMM9PARTA002442		0.00967922	2.2064419	up
ASMM9PARTA009181		0.038325503	2.0786164	up
ASMM9PARTA009181		0.038325503	2.0786164	up
ASMM9PARTA009181		0.038325503	2.0786164	up
ASMM9PARTA009181		0.038325503	2.0786164	up
ASMM9PARTA009181		0.038325503	2.0786164	up
ASMM9PARTA047307	1300014I06Rik	0.002641702	2.3888183	up
ASMM9PARTA045392	Airn	0.0000256	2.243639	up
ASMM9PARTA045392	Airn	0.0000256	2.243639	up
ASMM9PARTA048548	AK079730	0.001127711	2.1647818	up
MM9LINCRNAEXON10998-_P1	mouselincRNA0882	0.015723212	3.3525438	up
ASMM9PARTA051664	Trip4	0.000879	4.542469	up
ASMM9PARTA051664	Trip4	0.000879	4.542469	up
ASMM9PARTA051664	Trip4	0.000879	4.542469	up
ASMM9PARTA002580		0.0000112	2.297891	up
ASMM9PARTA013480	Gm13024	1.51E-08	2.6485834	up
ASMM9PARTA050025	nssr 1	0.00000199	3.2862563	up
ASMM9PARTA050025	nssr 1	0.00000199	3.2862563	up
ASMM9PARTA003955		0.000233	2.1015546	up
ASMM9PARTA049316	AK041310	0.007011698	2.3175473	up
ASMM9PARTA049316	AK041310	0.007011698	2.3175473	up
ASMM9PARTA049316	AK041310	0.007011698	2.3175473	up
BF787814_P1	mouselincRNA0498	0.005410496	2.292607	up
ASMM9PARTA019016	RP24-357O21.3	0.000107	3.0634668	up
mouselincRNA0963-_P1	mouselincRNA0963	0.02520888	2.1265574	up
ASMM9PARTA012429	Gm12587	0.00000843	2.5660658	up
humanlincRNA0055-_P1	humanlincRNA0055	0.002467079	2.166897	up
ASMM9PARTA006085		0.000545	2.6730473	up
ASMM9PARTA006085		0.000545	2.6730473	up
ASMM9PARTA010972	Mup-ps6	0.0000161	4.022564	up
ASMM9PARTA019098	AC121792.3	0.033514142	2.2735584	up
ASMM9PARTA007353		0.042540945	2.275102	up
ASMM9PARTA001194		0.002265764	2.494671	up
MM9LINCRNAEXON12018-_P1	mouselincRNA0031	0.001699114	2.0568318	up
ASMM9PARTA044839	Vmn2r-ps14	0.029846843	4.0783963	up
ASMM9PARTA049975	AK043904	0.0000207	2.6258962	up
ASMM9PARTA049688	AK145113	0.014496135	2.06082	up
ASMM9PARTA018606	RP23-126F14.6	0.0000202	6.4950004	up
ASMM9PARTA004654		0.006798118	2.5093179	up

humanlincRNA1385-_P1	humanlincRNA1385	0.0000319	3.433167	up
ASMM9PARTA013365	Gm11341	0.0000144	2.271584	up
ASMM9PARTA051782	AK142198	0.000049	3.3818111	up
ASMM9PARTA051782	AK142198	0.000049	3.3818111	up
ASMM9PARTA051782	AK142198	0.000049	3.3818111	up
ASMM9PARTA051782	AK142198	0.000049	3.3818111	up
ASMM9PARTA051782	AK142198	0.000049	3.3818111	up
ASMM9PARTA010242	Gm13303	0.000153	4.23593	up
ASMM9PARTA008632		0.007488906	2.1066258	up
ASMM9PARTA006527		0.002830179	2.5977042	up
ASMM9PARTA012197	Gm12331	0.00000216	2.7013195	up
ASMM9PARTA047146	AK087290	0.008651675	2.5153308	up
ASMM9PARTA047146	AK087290	0.008651675	2.5153308	up
AV290481_P1	humanlincRNA2040	0.001539665	2.069698	up
ASMM9PARTA002907		0.01911226	2.2169294	up
ASMM9PARTA009998	4632427E13Rik	0.0000324	2.0722952	up
ASMM9PARTA007196		0.002476982	2.7026877	up
ASMM9PARTA005873		0.000387	2.5644972	up
ASMM9PARTA016340	BC006965	0.002563251	3.8890753	up
ASMM9PARTA047011	AK163447	0.003627984	3.3370185	up
MM9LINCRNAEXON11933-_P1	mouselincRNA0196	0.019213183	2.75035	up
MM9LINCRNAEXON10980-_P1	mouselincRNA0870	0.040502742	2.7829013	up
ASMM9PARTA013866	4930455G09Rik	0.000157	3.1369178	up
ASMM9PARTA047100	AK005722	0.012690729	3.3796914	up
ASMM9PARTA047100	AK005722	0.012690729	3.3796914	up
ASMM9PARTA010799	Gm4919	0.001001884	2.0900185	up
MM9LINCRNAEXON10348-_P1	mouselincRNA1396	0.000156	2.016701	up
ASMM9PARTA003188		0.000343	2.025452	up
ASMM9PARTA003188		0.000343	2.025452	up
ASMM9PARTA046645	IL-TIFb	0.0000524	4.7472396	up
ASMM9PARTA014593	Gm15169	0.000143	3.8509307	up
ASMM9PARTA013056	Gm14143	0.000487	2.3145373	up
ASMM9PARTA009944	Gm10075	0.0000234	2.6519034	up
humanlincRNA1016-_P1	humanlincRNA1016	0.0000528	2.2864692	up
ASMM9PARTA010113	Hsp25-ps1	0.00035	3.3957517	up
ASMM9PARTA049247	AK040741	0.012025277	3.1804085	up
MM9LINCRNAEXON10743-_P1	mouselincRNA1136	0.003954184	3.8251135	up
ASMM9PARTA007248		0.005026355	3.4147952	up
ASMM9PARTA013839	9330185C12Rik	0.026189921	2.1763446	up
ASMM9PARTA047150	AK019365	0.00000601	2.724738	up
ASMM9PARTA012029	Gm13823	0.006309441	2.0960474	up
ASMM9PARTA049465	DQ697944	0.017790703	2.5509765	up
ASMM9PARTA001889		0.0000285	3.873282	up
ASMM9PARTA002853		0.00000522	2.934644	up
ASMM9PARTA002853		0.00000522	2.934644	up
ASMM9PARTA002853		0.00000522	2.934644	up
ASMM9PARTA011809	Gm13302	0.000000181	4.4235177	up
ASMM9PARTA003783		0.000389	2.087983	up
MM9LINCRNAEXON10136+_P1	mouselincRNA1563	0.000027	3.0390553	up

ASMM9PARTA014155	9330111N05Rik	0.000139	11.575967	up
ASMM9PARTA011466	Gm14874	0.000701	2.2405474	up
CUST_910_PI426073487	uc.431	0.04850129	2.2293694	up
ASMM9PARTA048130	Trhr	0.001019991	2.0016923	up
MM9LINCRNAEXON10474+_P1	mouselincRNA1297	0.020328147	2.9579363	up
ASMM9PARTA005229		0.00000227	28.594591	up
ASMM9PARTA048564	AK009785	0.03720002	3.1501374	up
ASMM9PARTA050840	AK050360	0.000423	2.505112	up
ASMM9PARTA010591	Gm12131	0.000121	3.5433466	up
ASMM9PARTA011266	Gm12311	0.000151	2.9004962	up
ASMM9PARTA051813	Cdcp1	0.0000137	2.7459238	up
ASMM9PARTA017061	Gm7809	0.000413	2.185241	up
ASMM9PARTA019500	AC133517.1	0.002038022	3.314145	up
ASMM9PARTA004792		0.005439912	2.7279453	up
ASMM9PARTA015228	Gm12576	0.004371155	4.1432953	up
ASMM9PARTA047058	Rtn-1A	0.006999876	2.5087316	up
ASMM9PARTA047058	Rtn-1A	0.006999876	2.5087316	up
MM9LINCRNAEXON12111+_P1	mouselincRNA0122	0.007135308	2.1534543	up
ASMM9PARTA049940	DQ687153	0.000000968	2.0144653	up
ASMM9PARTA051687	AK208404	0.002799554	2.5060854	up
ASMM9PARTA051687	AK208404	0.002799554	2.5060854	up
ASMM9PARTA051687	AK208404	0.002799554	2.5060854	up
ASMM9PARTA012560	Gm8152	0.00000838	3.2602935	up
MM9LINCRNAEXON11832+_P1		0.0000489	3.6160138	up
ASMM9PARTA016151	Gm15704	0.00006	3.672915	up
ASMM9PARTA011885	Gm15607	0.0000306	2.0043821	up
ASMM9PARTA051133	AK162666	0.00388528	2.1561172	up
ASMM9PARTA011914	Gm12757	0.000186	2.0429502	up
ASMM9PARTA016414	3110053B16Rik	0.00000497	3.0861878	up
ASMM9PARTA009310		0.000121	2.0006466	up
CUST_115_PI426073487	uc.117	0.005279679	2.0733721	up
ASMM9PARTA017746	Gm12406	0.000462	3.0414312	up
MM9LINCRNAEXON11100-_P1		0.00000391	4.5987887	up
ASMM9PARTA051103	AK076954	0.00000596	3.782889	up
ASMM9PARTA003989		0.008304258	2.4503531	up
ASMM9PARTA015857	Gm16098	0.006875705	3.8277466	up
ASMM9PARTA019156	RP24-391B9.1	0.000496	3.6533327	up
ASMM9PARTA002182		0.000104	2.4740412	up
ASMM9PARTA009208		0.0000104	3.987226	up
ASMM9PARTA009208		0.0000104	3.987226	up
ASMM9PARTA050441	AK040808	0.009551179	2.5817962	up
ASMM9PARTA050441	AK040808	0.009551179	2.5817962	up
ASMM9PARTA017258	D830026I12Rik	0.023657888	3.1213667	up
MM9LINCRNAEXON11819+_P1	mouselincRNA0268	0.000732	2.1125832	up
ASMM9PARTA046687	AK040172	0.037222065	2.3282819	up
ASMM9PARTA046687	AK040172	0.037222065	2.3282819	up
ASMM9PARTA002287		0.023207642	2.875669	up
ASMM9PARTA010091	Speer4c	0.016464226	3.7602234	up
ASMM9PARTA015043	Gm15751	0.019191118	2.1902726	up

ASMM9PARTA015043	Gm15751	0.019191118	2.1902726	up
ASMM9PARTA006843		0.0000043	2.4403582	up
ASMM9PARTA003909		0.030186526	2.0060174	up
ASMM9PARTA009504	1700018A04Rik	0.035345774	2.1170304	up
ASMM9PARTA014677	A830036E02Rik	0.000272	72.04179	up
CUST_475_PI426073487	uc.477	0.000165	2.1766112	up
ASMM9PARTA049899	C230055K05Rik	0.000434	3.43939	up
ASMM9PARTA013104	Gm14732	0.000757	2.1510677	up
ASMM9PARTA003355		0.0000737	3.0531328	up
ASMM9PARTA048523	Sil1	0.004610907	5.801971	up
ASMM9PARTA002760		0.04423434	2.1806612	up
ASMM9PARTA047428	1700049E17Rik	0.000203	2.021194	up
ASMM9PARTA012505	Gm10601	0.000000231	8.00714	up
CUST_160_PI426409190		0.010918572	2.4761918	up
mouselincRNA0344+_P1	mouselincRNA0344	0.0000191	2.8268309	up
ASMM9PARTA014359	Gm12648	0.010400829	2.5174477	up
ASMM9PARTA008936		0.000466	2.3162975	up
ASMM9PARTA015458	A230087F16Rik	0.00000393	3.058178	up
ASMM9PARTA007503		0.047567993	2.0747998	up
ASMM9PARTA018983	AL606528.1	0.00035	4.073817	up
ASMM9PARTA011035	Gm12445	0.034812786	2.8441324	up
ASMM9PARTA018865	Gm6490	0.000288	2.0393522	up
MM9LINCRNAEXON10522+_P1	mouselincRNA1351	0.006218692	2.1970863	up
ASMM9PARTA011263	Gm12832	0.005363323	2.55853	up
ASMM9PARTA006044		0.0000579	4.022443	up
MM9LINCRNAEXON11785-_P1		0.025044193	2.106627	up
ASMM9PARTA019007	RP24-284A21.1	0.005916194	2.312445	up
MM9LINCRNAEXON11828+_P1	mouselincRNA0271	0.016319588	2.0236769	up
ASMM9PARTA011632	Gm15484	0.0000113	2.0514188	up
ASMM9PARTA046930	AK018772	0.00000751	2.3355148	up
ASMM9PARTA007292		0.002100656	3.75971	up
ASMM9PARTA007292		0.002100656	3.75971	up
ASMM9PARTA007292		0.002100656	3.75971	up
ASMM9PARTA012573	Gm7571	0.011855153	3.0073943	up
ASMM9PARTA012893	Hmgb1-ps5	0.00000696	2.9856458	up
ASMM9PARTA019819	RP23-122J17.9	0.000017	5.8084054	up
ASMM9PARTA045780	9330020H09Rik	0.00025	3.1872258	up
ASMM9PARTA010608	Gm14448	0.005900046	2.00024	up
ASMM9PARTA007306		0.001683497	2.8407989	up
ASMM9PARTA016175	5830428H23Rik	0.00093	3.1199622	up
ASMM9PARTA002749		0.001053289	5.264673	up
ASMM9PARTA010688	Gm15059	0.0000249	3.045087	up
ASMM9PARTA011117	Zfp389	0.000338	2.0679224	up
ASMM9PARTA005625		0.00000487	4.1940975	up
ASMM9PARTA005625		0.00000487	4.1940975	up
ASMM9PARTA005625		0.00000487	4.1940975	up
ASMM9PARTA016813	D430040D24Rik	0.00000114	9.755448	up
ASMM9PARTA016813	D430040D24Rik	0.00000114	9.755448	up
ASMM9PARTA016813	D430040D24Rik	0.00000114	9.755448	up

ASMM9PARTA046016	Olfr856-ps1	0.002315608	4.6385484	up
ASMM9PARTA018017	Gm16169	0.029165156	2.3030307	up
ASMM9PARTA009000		0.000111	3.9146273	up
ASMM9PARTA049978	Mup1	0.0000106	5.7322636	up
ASMM9PARTA047303	Heatr1	0.042350072	2.6235144	up
ASMM9PARTA047303	Heatr1	0.042350072	2.6235144	up
ASMM9PARTA047303	Heatr1	0.042350072	2.6235144	up
ASMM9PARTA046691	AK158612	0.001038566	9.658383	up
ASMM9PARTA049380	AK039272	0.0000337	2.2423038	up
ASMM9PARTA002955		0.0000219	2.086599	up
ASMM9PARTA003150		0.00000548	2.819932	up
ASMM9PARTA045252	1700086O06Rik	0.0000219	2.1372287	up
ASMM9PARTA003905		0.00000118	2.6994221	up
ASMM9PARTA006118		0.000000159	2.2417	up
ASMM9PARTA006118		0.000000159	2.2417	up
MM9LINCRNAEXON10071+_P1	mouselincRNA1506	0.0000204	2.583075	up
ASMM9PARTA009337	H1f0	0.00014	2.0743325	up
DV645173_P1	humanlincRNA0146	4.32E-08	3.0676453	up
ASMM9PARTA013232	Gm15570	0.000216	2.0401762	up
ASMM9PARTA004884		0.0000649	2.125914	up
ASMM9PARTA012278	Gm15794	0.011537045	2.8566773	up
ASMM9PARTA003161		0.013103615	2.4388435	up
ASMM9PARTA007109		0.006785432	2.0615892	up
ASMM9PARTA047833	AK142028	0.0000204	3.225097	up
ASMM9PARTA047833	AK142028	0.0000204	3.225097	up
ASMM9PARTA010702	Gm9850	0.003652586	2.4966033	up
ASMM9PARTA014203	A230059L01Rik	0.01500843	3.5056098	up
ASMM9PARTA014203	A230059L01Rik	0.01500843	3.5056098	up
ASMM9PARTA045485	BC024582	0.041472863	2.1599445	up
ASMM9PARTA002496		0.004153596	3.3635817	up
ASMM9PARTA002496		0.004153596	3.3635817	up
ASMM9PARTA048866	AK052627	0.000263	2.1936817	up
ASMM9PARTA018767	Gm16558	0.005763229	2.4630747	up
ASMM9PARTA007427		0.000152	15.231653	up
ASMM9PARTA049015	AK052812	0.005637396	2.6731706	up
ASMM9PARTA010939	Gm15264	0.000214	3.7721858	up
ASMM9PARTA001980		0.004348782	3.3548255	up
ASMM9PARTA048619	AK043286	0.000325	2.0171144	up
ASMM9PARTA048619	AK043286	0.000325	2.0171144	up
ASMM9PARTA048619	AK043286	0.000325	2.0171144	up
ASMM9PARTA018883	Gm9946	0.0000694	2.842234	up
ASMM9PARTA003855		0.000229	3.9180405	up
ASMM9PARTA001731		0.002414143	2.0229678	up
ASMM9PARTA011472	Gm12988	0.000000334	2.010097	up
ASMM9PARTA009795	Gm13775	0.0000268	5.2353888	up
ASMM9PARTA011317	Hmgb1-ps3	0.00000706	2.9804971	up
ASMM9PARTA047057	AK015004	0.016565895	2.1351128	up
ASMM9PARTA047057	AK015004	0.016565895	2.1351128	up
ASMM9PARTA051121	Lrrc49	0.00000176	56.598614	up

ASMM9PARTA051121	Lrrc49	0.00000176	56.598614	up
ASMM9PARTA051121	Lrrc49	0.00000176	56.598614	up
ASMM9PARTA051405	BC048761	0.003030621	5.1933436	up
ASMM9PARTA051372	BC048584	0.000533	14.077623	up
ASMM9PARTA049809	AK030206	0.0000469	3.4416368	up
ASMM9PARTA049809	AK030206	0.0000469	3.4416368	up
ASMM9PARTA013829	Gm16881	0.0000861	2.7217197	up
ASMM9PARTA013829	Gm16881	0.0000861	2.7217197	up
ASMM9PARTA013829	Gm16881	0.0000861	2.7217197	up
ASMM9PARTA000678		0.00000402	2.0657392	up
ASMM9PARTA002375		0.00000033	29.815798	up
ASMM9PARTA015917	A930028C08Rik	0.006757761	2.8449636	up
ASMM9PARTA003040		0.002429769	4.1964455	up
ASMM9PARTA015540	Gm14271	0.000123	4.1221213	up
ASMM9PARTA018971	AC165246.1	0.000443	4.2349753	up
ASMM9PARTA047410	AK036683	0.005072215	2.8604343	up
ASMM9PARTA008364		0.000999	2.244141	up
ASMM9PARTA008364		0.000999	2.244141	up
ASMM9PARTA050412	AK006664	0.016164016	2.0065231	up
ASMM9PARTA013178	Gm13300	0.000000132	4.1533117	up
ASMM9PARTA013763	Gm4772	0.011266023	2.366016	up
ASMM9PARTA019456	AC154013.1	0.003402773	6.8527093	up
ASMM9PARTA002539		0.0000821	2.2914972	up
ASMM9PARTA017529	6720416L17Rik	0.00310736	3.1422844	up
ASMM9PARTA013339	Gm15388	0.004796248	2.186649	up
ASMM9PARTA004233		0.000597	4.042444	up
ASMM9PARTA007792		0.001280179	2.9346867	up
ASMM9PARTA012546	Gm12482	0.000147	2.6187327	up
ASMM9PARTA049860	Orai1	0.002793875	2.4832017	up
ASMM9PARTA017284	Gm16762	0.000257	4.5537424	up
humanlincRNA0194+_P1	humanlincRNA0194	0.030545868	2.594462	up
ASMM9PARTA019183	AC102815.2	0.002170969	2.1039898	up
ASMM9PARTA014232	Gm15169	0.01783746	2.7520053	up
ASMM9PARTA004055		2.72E-09	176.79759	up
ASMM9PARTA006158		0.0000859	2.583311	up
ASMM9PARTA015093	A530058N18Rik	0.00000423	2.821805	up
ASMM9PARTA051011	Pgls	0.001753105	2.686602	up
ASMM9PARTA051011	Pgls	0.001753105	2.686602	up
ASMM9PARTA007558		0.000000772	4.0662074	up
ASMM9PARTA003147		0.000771	2.931653	up
CUST_375_PI426073487	uc.377	0.009904888	5.316401	up
ASMM9PARTA047189	AK019332	0.0000202	2.5726964	up
ASMM9PARTA050230	AK131834	0.000197	31.991306	up
ASMM9PARTA015208	Gm12326	0.000504	2.3253443	up
ASMM9PARTA004661		0.000755	2.3564363	up
CUST_428_PI426073487	uc.430	0.000000364	9.030763	up
CUST_428_PI426073487	uc.430	0.000000364	9.030763	up
CUST_428_PI426073487	uc.430	0.000000364	9.030763	up
CUST_428_PI426073487	uc.430	0.000000364	9.030763	up

CUST_428_PI426073487	uc.430	0.000000364	9.030763	up
ASMM9PARTA050850	Mcl	0.011990267	3.4236717	up
ASMM9PARTA050850	Mcl	0.011990267	3.4236717	up
ASMM9PARTA051221	Phkb	0.0000718	2.0668428	up
ASMM9PARTA051221	Phkb	0.0000718	2.0668428	up
ASMM9PARTA004516		0.000144	2.6064804	up
ASMM9PARTA003133		0.01317557	2.1849236	up
ASMM9PARTA006828		0.000000413	2.5142744	up
ASMM9PARTA048587	AK076984	0.0000156	5.2759423	up
ASMM9PARTA014306	3110053B16Rik	0.0000153	2.1579196	up
ASMM9PARTA013266	Gm13214	0.002649052	2.434913	up
ASMM9PARTA010400	9330158H04Rik	0.0000385	5.3170137	up
ASMM9PARTA011807	Gm15403	0.0000573	2.3673801	up
ASMM9PARTA007283		0.001576614	3.1284359	up
ASMM9PARTA003077		0.000142	2.3230376	up
ASMM9PARTA010302	Mup-ps7	0.015276058	3.9666667	up
CUST_954_PI426073487	uc.475	0.000406	2.6264794	up
ASMM9PARTA019550	RP23-78F6.7	0.0000421	2.9541492	up
humanlincRNA1152+_P1	humanlincRNA1152	0.00397527	2.7070966	up
ASMM9PARTA047836	4833420G17Rik	0.000000226	3.4735205	up
ASMM9PARTA047836	4833420G17Rik	0.000000226	3.4735205	up
ASMM9PARTA044959	E230029C05Rik	0.0000424	3.0127733	up
ASMM9PARTA005240		0.000433	3.6069376	up
ASMM9PARTA046418	BC018310	0.0000113	2.1052809	up
ASMM9PARTA051161	AK169831	0.00000542	2.4244323	up
CUST_100_PI426409190	AC087559.13	3.26E-08	2.1758285	up
ASMM9PARTA001853		0.0000135	3.704979	up
ASMM9PARTA047453	AK039140	0.0000558	2.8066964	up
ASMM9PARTA047453	AK039140	0.0000558	2.8066964	up
ASMM9PARTA010001	Gm5792	0.00000517	2.0754125	up
ASMM9PARTA047221	AK040557	0.0000025	7.556741	up
ASMM9PARTA047221	AK040557	0.0000025	7.556741	up
ASMM9PARTA047221	AK040557	0.0000025	7.556741	up
ASMM9PARTA019295	RP23-133I8.1	0.00000159	2.4911675	up
ASMM9PARTA047783	Ugcgl2	0.0000136	5.172222	up
ASMM9PARTA003032		0.014006707	2.4242249	up
ASMM9PARTA003032		0.014006707	2.4242249	up
ASMM9PARTA003032		0.014006707	2.4242249	up
ASMM9PARTA019216	5830444B04Rik	0.012012539	2.1467376	up
ASMM9PARTA019216	5830444B04Rik	0.012012539	2.1467376	up
ASMM9PARTA010393	Gm16221	0.0000371	2.2648113	up
ASMM9PARTA018255	Gm8837	0.000374	3.0742378	up
ASMM9PARTA002372		0.000325	3.9762456	up
ASMM9PARTA019677	RP23-82A21.1	0.022848116	2.836978	up
ASMM9PARTA012510	Gm13121	0.00000484	2.3607845	up
ASMM9PARTA016207	4930458D05Rik	0.010438909	6.2100377	up
ASMM9PARTA044960	BC006965	0.0000162	67.15441	up
ASMM9PARTA016551	Gm16259	0.000094	2.6952586	up
ASMM9PARTA016551	Gm16259	0.000094	2.6952586	up

ASMM9PARTA011946	Mup-ps10	0.000041	4.4082055	up
ASMM9PARTA012055	Gm12118	0.000036	2.516198	up
ASMM9PARTA016684	Gm14261	0.000844	2.701113	up
ASMM9PARTA016684	Gm14261	0.000844	2.701113	up
ASMM9PARTA016684	Gm14261	0.000844	2.701113	up
AA915284_P1	mouselincRNA0740	0.000203	2.3214035	up
ASMM9PARTA011936	Gm13320	0.00000614	2.537862	up
ASMM9PARTA013691	Gm14162	0.0000901	2.7502673	up
ASMM9PARTA018548	Airn	0.0000222	2.430412	up
ASMM9PARTA018548	Airn	0.0000222	2.430412	up
ASMM9PARTA016258	2410004N09Rik	0.0000808	3.2196906	up
ASMM9PARTA044844	Rps4y2	0.000079	2.1135077	up
ASMM9PARTA007420		0.000004	2.7572374	up
ASMM9PARTA007420		0.000004	2.7572374	up
ASMM9PARTA004325		0.000442	2.0255837	up
ASMM9PARTA013969	Gm11681	0.00137149	2.2397988	up
ASMM9PARTA014091	4930419G24Rik	0.026020661	3.9461677	up
ASMM9PARTA014091	4930419G24Rik	0.026020661	3.9461677	up
ASMM9PARTA012968	Gm15566	0.0000464	2.8185575	up
ASMM9PARTA010789	Gm12449	0.000062	2.546853	up
ASMM9PARTA046840	AK179786	0.0000688	2.6710253	up
ASMM9PARTA047612	AK039634	0.00000587	3.522073	up
ASMM9PARTA010447	Gm6088	0.002052184	2.4699576	up
ASMM9PARTA010097	Gm10361	0.00000536	2.9667628	up
ASMM9PARTA000537		0.00000273	5.9810114	up
ASMM9PARTA045288	2610204G22Rik	0.000000141	2.1404438	up
ASMM9PARTA001715		0.000000918	2.5126276	up
ASMM9PARTA003926		0.000024	2.889864	up
ASMM9PARTA012662	Olfr334-ps1	0.003399612	2.0054133	up
ASMM9PARTA050454	AK076606	9.56E-09	445.75082	up
ASMM9PARTA014139	Gm3160	0.000000231	2.1778157	up
ASMM9PARTA014139	Gm3160	0.000000231	2.1778157	up
ASMM9PARTA005558		0.00000673	2.755438	up
ASMM9PARTA018516	Gm16290	0.000409	2.134348	up
MM9LINCRNAEXON11314+_P1	mouselincRNA0661	0.000611	2.0327842	up
ASMM9PARTA015674	Gm13112	0.0000193	5.197551	up
ASMM9PARTA015674	Gm13112	0.0000193	5.197551	up
ASMM9PARTA015409	Gm10565	0.004117666	2.9403174	up
ASMM9PARTA013565	Gm14473	0.0000259	2.5847037	up
ASMM9PARTA003940		0.03034472	2.2634487	up
ASMM9PARTA011002	Gm15310	0.001346174	2.4780962	up
ASMM9PARTA050623	Kox-1	0.004144472	2.7868278	up
ASMM9PARTA007842		0.000208	2.2019105	up
ASMM9PARTA049446	AK031919	0.000234	2.8039787	up
ASMM9PARTA000684		4.03E-08	6.1553197	up
ASMM9PARTA018531	RP23-274C16.2	0.002185957	2.0214283	up
ASMM9PARTA011785	Gm15575	0.003231675	2.075073	up
ASMM9PARTA007743		0.005448009	2.0896385	up
AU018725_P1	humanlincRNA2076	0.033706043	2.0233583	up

ASMM9PARTA018872	RP23-340E10.2	0.000059	2.3255289	up
ASMM9PARTA005051		0.016797869	2.3561113	up
ASMM9PARTA046430	AK153590	0.042234577	2.5575266	up
ASMM9PARTA019300	RP24-246L19.3	0.000135	2.1255887	up
ASMM9PARTA013453	Gm16471	0.000025	3.5457897	up
ASMM9PARTA012045	Gm14788	0.00003	2.640837	up
ASMM9PARTA003533		0.000504	2.0738938	up
ASMM9PARTA012531	Gm13385	0.001866921	2.0577526	up
ASMM9PARTA012955	Gm13932	0.00000918	2.5971808	up
ASMM9PARTA010463	Rpl13-ps5	0.0003	2.5084457	up
ASMM9PARTA045371	5033406O09Rik	0.000151	19.874756	up
ASMM9PARTA002899		0.0000198	3.3824632	up
ASMM9PARTA002899		0.0000198	3.3824632	up
ASMM9PARTA002899		0.0000198	3.3824632	up
ASMM9PARTA011250	Gm11920	0.000406	2.3924294	up
ASMM9PARTA001846		0.000206	2.582115	up
ASMM9PARTA010260	Mup-ps2	0.0000222	5.4068246	up
ASMM9PARTA002438		0.001891725	3.4894876	up
ASMM9PARTA049624	BC048720	0.000154	2.3107316	up
ASMM9PARTA047625	AK153491	0.00000561	2.0996218	up
CUST_846_PI426073487	uc.367	0.016572911	2.6700237	up
ASMM9PARTA046971	AK138212	0.00166566	12.716476	up
ASMM9PARTA014153	Gm12221	0.018201962	2.487967	up
AK138796_P1	humanlincRNA0850	0.000000858	6.973278	up
AK138796_P1	humanlincRNA0850	0.000000858	6.973278	up
AK138796_P1	humanlincRNA0850	0.000000858	6.973278	up
ASMM9PARTA001331		0.008813274	3.2148635	up
ASMM9PARTA002208		0.00017	2.1622796	up
ASMM9PARTA004305		0.00000519	2.4267251	up
ASMM9PARTA013646	D230017M19Rik	0.000366	10.863149	up
ASMM9PARTA048585	C230097I24Rik	0.000121	2.4461868	up
ASMM9PARTA004186		0.031331185	2.460959	up
ASMM9PARTA018972	Gm5859	0.000000223	12.615074	up
ASMM9PARTA018089	RP23-11E12.5	0.00243224	2.2377012	up
ASMM9PARTA011394	Gm11869	0.002306182	2.0773122	up
ASMM9PARTA050419	AK158055	2.53E-09	52.151836	up
MM9LINCRNAEXON11899+_P1	mouselincRNA0173	0.032026485	2.2022645	up
ASMM9PARTA047828	AK035211	0.030093784	3.412731	up
ASMM9PARTA046663	AK053193	0.007637039	2.9658413	up
MM9LINCRNAEXON12113-_P1		0.00000972	2.0957308	up
ASMM9PARTA011830	Mup-ps3	0.0000276	6.961308	up
ASMM9PARTA003927		0.000162	132.02542	up
humanlincRNA0635+_P1	humanlincRNA0635	0.02540335	2.9597874	up
ASMM9PARTA049445	AK080657	0.000114	2.5796616	up
ASMM9PARTA002402		0.000638	2.1015816	up
ASMM9PARTA019681	RP23-273L20.6	0.000205	2.0095043	up
ASMM9PARTA046705	AK083030	0.000000741	3.9425938	up
ASMM9PARTA017565	Gm15545	0.009264852	2.21652	up
ASMM9PARTA017565	Gm15545	0.009264852	2.21652	up

ASMM9PARTA004963		0.0000727	3.7298281	up
ASMM9PARTA050624	BC048663	0.018868716	2.6872802	up
ASMM9PARTA007871		0.0000106	2.9992294	up
ASMM9PARTA002181		0.000000879	4.8205237	up
ASMM9PARTA010492	Gm13378	0.0000721	2.2401564	up
EL605756_P1	humanlincRNA0451	0.000506	3.2630463	up
ASMM9PARTA003760		0.0000485	2.9186254	up
ASMM9PARTA016760	Gm2694	0.004579674	2.3607674	up
ASMM9PARTA019401	9430021M05Rik	0.000000957	5.3567877	up
ASMM9PARTA019105	RP24-84D8.2	0.022724025	2.753335	up
ASMM9PARTA008261		0.011984917	2.3411644	up
ASMM9PARTA045550	A230073K19Rik	0.00000821	2.0862916	up
ASMM9PARTA045550	A230073K19Rik	0.00000821	2.0862916	up
ASMM9PARTA045550	A230073K19Rik	0.00000821	2.0862916	up
ASMM9PARTA013742	Gm11506	0.00053	2.2343495	up
ASMM9PARTA004719		0.006178631	2.0931888	up
BC040767_P1	humanlincRNA1200	1.49E-08	76.40738	up
ASMM9PARTA013516	Gm6044	0.0000643	2.0916908	up
MM9LINCRNAEXON10767+_P1	mouselincRNA1138	0.002600128	2.709299	up
ASMM9PARTA004878		0.0465896	2.0205524	up
ASMM9PARTA003919		0.000000574	2.638879	up
ASMM9PARTA018895	RP23-388P16.10	0.0000918	2.4401617	up
ASMM9PARTA001789		0.0000895	2.039609	up
ASMM9PARTA001789		0.0000895	2.039609	up
ASMM9PARTA048266	AK143195	0.008352266	2.0969896	up
ASMM9PARTA016687	4930430E12Rik	0.000128	2.210298	up
ASMM9PARTA010790	Gm12795	0.000448	2.068478	up
ASMM9PARTA006908		0.022319157	2.7486594	up
ASMM9PARTA048898	BC062254	0.002160315	2.089609	up
MM9LINCRNAEXON10776+_P1	mouselincRNA1143	0.006271291	2.0736318	up
ASMM9PARTA018543	AC160929.1	0.00000864	2.8037739	up
ASMM9PARTA011748	Gm12806	0.002210625	2.262587	up
ASMM9PARTA011748	Gm12806	0.002210625	2.262587	up
ASMM9PARTA013281	Gm12745	0.0000951	2.6216104	up
ASMM9PARTA002778		0.000121	2.8734906	up
ASMM9PARTA051343	Perq1	0.00112595	2.0589914	up
ASMM9PARTA051343	Perq1	0.00112595	2.0589914	up
ASMM9PARTA051343	Perq1	0.00112595	2.0589914	up
ASMM9PARTA051343	Perq1	0.00112595	2.0589914	up
ASMM9PARTA051343	Perq1	0.00112595	2.0589914	up
ASMM9PARTA051343	Perq1	0.00112595	2.0589914	up
ASMM9PARTA051343	Perq1	0.00112595	2.0589914	up
ASMM9PARTA051343	Perq1	0.00112595	2.0589914	up
ASMM9PARTA051343	Perq1	0.00112595	2.0589914	up
ASMM9PARTA051343	Perq1	0.00112595	2.0589914	up
ASMM9PARTA011486	Cbx3-ps3	0.001600628	3.499587	up
ASMM9PARTA046796	BC030470	0.001346888	2.1043758	up
ASMM9PARTA000041		0.005214016	3.1388874	up
MM9LINCRNAEXON11795+_P1	mouselincRNA0252	0.0000133	52.483807	up

MM9LINCRNAEXON10103+_P1	mouselincRNA1524	0.0000585	5.55137	up
ASMM9PARTA015949	5330413P13Rik	0.004337896	2.9964545	up
ASMM9PARTA010261	Gm10601	0.000000999	6.9348526	up
ASMM9PARTA050367	Mcl	0.00077	4.3386984	up
ASMM9PARTA050367	Mcl	0.00077	4.3386984	up
ASMM9PARTA009606	Gm9791	0.000787	2.7296593	up
MM9LINCRNAEXON11138-_P1		0.000105	2.2227647	up
ASMM9PARTA004712		0.000755	3.633781	up
ASMM9PARTA046229	4933407L21Rik	0.0000222	2.043116	up
ASMM9PARTA000415		0.011903576	2.359702	up
ASMM9PARTA046758	Fbxo39	0.001037672	2.0745504	up
ASMM9PARTA006182		0.000485	2.1920044	up
ASMM9PARTA005256		0.000408	2.2233198	up
ASMM9PARTA016109	Gm16682	0.006650837	2.0518725	up
ASMM9PARTA016109	Gm16682	0.006650837	2.0518725	up
ASMM9PARTA005775		0.000362	2.6580997	up
mouselincRNA1564-_P1	mouselincRNA1564	0.00399927	2.8611562	up
ASMM9PARTA012547	Gm12308	0.000138	2.4590018	up
ASMM9PARTA011271	Gm13228	0.000205	2.2385955	up
BU563586_P1	mouselincRNA1020	0.0000177	2.8214636	up
BU563586_P1	mouselincRNA1020	0.0000177	2.8214636	up
BU563586_P1	mouselincRNA1020	0.0000177	2.8214636	up
BU563586_P1	mouselincRNA1020	0.0000177	2.8214636	up
BU563586_P1	mouselincRNA1020	0.0000177	2.8214636	up
BU563586_P1	mouselincRNA1020	0.0000177	2.8214636	up
BU563586_P1	mouselincRNA1020	0.0000177	2.8214636	up
BU563586_P1	mouselincRNA1020	0.0000177	2.8214636	up
BU563586_P1	mouselincRNA1020	0.0000177	2.8214636	up
BU563586_P1	mouselincRNA1020	0.0000177	2.8214636	up
BU563586_P1	mouselincRNA1020	0.0000177	2.8214636	up
BU563586_P1	mouselincRNA1020	0.0000177	2.8214636	up
ASMM9PARTA011414	Gm15778	0.0000358	2.7669818	up
ASMM9PARTA050140	AK050091	0.0447964	2.1253493	up
ASMM9PARTA019398	RP24-376M8.4	0.016682565	2.4897163	up
ASMM9PARTA002398		0.018772587	2.245651	up
ASMM9PARTA050618	AK007376	0.002513846	5.951765	up
ASMM9PARTA003826		0.0000512	2.6118374	up
ASMM9PARTA045625	Hmgb1-rs17	0.0000351	2.2052023	up
ASMM9PARTA009936	Mup-ps7	0.000412	5.7239637	up
MM9LINCRNAEXON11341-_P1		0.001316639	2.0538328	up
ASMM9PARTA001043		0.0000404	2.2346153	up
ASMM9PARTA006890		0.01447322	3.7633927	up
ASMM9PARTA001365		0.0000293	2.2528622	up
ASMM9PARTA002329		0.00000686	8.89867	up
ASMM9PARTA010004	Gm8332	0.000635	2.1689298	up
ASMM9PARTA012094	Gm6461	0.00000557	2.7464592	up
ASMM9PARTA012215	Mup-ps19	0.0000575	10.208999	up
ASMM9PARTA007042		0.00524065	4.28312	up
ASMM9PARTA009756	Usp35	0.003795602	3.0378175	up

ASMM9PARTA012898	Gm5396	0.00000307	2.2883086	up
ASMM9PARTA001969		0.000000517	6.210554	up
CUST_174_PI426073487	uc.176	0.00296069	5.368654	up
ASMM9PARTA018095	Gm16990	0.0000511	7.036365	up
ASMM9PARTA010235	Gm13300	0.0000434	2.9077394	up
MM9LINCRNAEXON11850-_P1	mouselincRNA0276	0.0000261	2.2649379	up
CUST_103_PI426409190	AC087559.5	0.00000257	4.0644464	up
ASMM9PARTA007872		0.013928003	3.1891036	up
MM9LINCRNAEXON10492+_P1	mouselincRNA1320	0.011921521	2.6611245	up
ASMM9PARTA017214	Gm15135	0.029552817	2.2397726	up
MM9LINCRNAEXON10352+_P1		0.000248	2.9232256	up
ASMM9PARTA045124	2610203C22Rik	0.0000389	9.556516	up
ASMM9PARTA045124	2610203C22Rik	0.0000389	9.556516	up
ASMM9PARTA002204		0.0000175	2.1102927	up
ASMM9PARTA013698	Gm13366	0.03154388	2.083002	up
ASMM9PARTA007430		0.004021823	2.304885	up
ASMM9PARTA000642		0.044364646	2.075406	up
ASMM9PARTA012120	Gm5937	0.0000278	2.4145372	up
MM9LINCRNAEXON10420+_P1	mouselincRNA1264	1.18E-08	57.515907	up
ASMM9PARTA014788	B930095G15Rik	0.00000174	5.062689	up
ASMM9PARTA002762		0.039199233	2.2099137	up
ASMM9PARTA009923	Gm12222	0.000288	2.4782395	up
ASMM9PARTA002260		0.0000932	4.927838	up
ASMM9PARTA002260		0.0000932	4.927838	up
ASMM9PARTA048313	AK153988	0.0000588	2.1362422	up
MM9LINCRNAEXON10135+_P1	mouselincRNA1563	0.000368	2.2607794	up
ASMM9PARTA005006		0.04997351	2.363824	up
ASMM9PARTA010427	Gm13714	0.0000576	2.2930443	up
ASMM9PARTA004889		0.015661104	2.5439157	up
ASMM9PARTA045192	Ipw	0.00000968	6.6196465	up
ASMM9PARTA008991		0.004460741	3.7944827	up
ASMM9PARTA017528	Gm13643	0.000000823	12.318098	up
ASMM9PARTA010788	Gm12261	0.00000954	2.4132397	up
ASMM9PARTA002426		0.0000278	2.6535096	up
ASMM9PARTA011432	Gm15036	0.014262523	2.2848454	up
ASMM9PARTA045874	Gm10125	0.00000618	3.9956224	up
ASMM9PARTA014333	1500016L03Rik	0.003832524	3.660633	up
CUST_478_PI426073487	uc.480	0.001674797	2.727503	up
CUST_478_PI426073487	uc.480	0.001674797	2.727503	up
ASMM9PARTA050514	AK039340	0.000267	2.5338933	up
ASMM9PARTA011755	Hmgb1-ps6	0.000044	2.452737	up
ASMM9PARTA003346		0.000652	4.2312984	up
ASMM9PARTA003346		0.000652	4.2312984	up
ASMM9PARTA003370		0.018529471	2.1665783	up
ASMM9PARTA001844		0.000000869	8.687704	up
ASMM9PARTA019220	AC109232.1	0.00000138	6.507343	up
ASMM9PARTA008820		0.0000107	7.4877944	up
ASMM9PARTA015931	Mup-ps2	0.00105642	3.0925615	up
ASMM9PARTA009267	Hspe1-rs1	0.002262701	2.404381	up

ASMM9PARTA013762	Gm12956	0.001371216	2.5402799	up
ASMM9PARTA050763	NR_002895	0.0000468	2.680639	up
ASMM9PARTA018665	Rpsa-ps4	0.0000547	2.817196	up
humanlincRNA1563+_P1	humanlincRNA1563	0.025340265	2.0704727	up
ASMM9PARTA014299	Gm13375	0.000103	2.2424648	up
ASMM9PARTA047295	AK014224	0.001279673	3.3696115	up
ASMM9PARTA008253		0.000447	2.541515	up
ASMM9PARTA008253		0.000447	2.541515	up
CUST_327_PI426073487	uc.329	0.03800932	2.0488782	up
CUST_327_PI426073487	uc.329	0.03800932	2.0488782	up
ASMM9PARTA012250	Gm15387	0.0000136	2.4925725	up
ASMM9PARTA010666	Gm13890	0.000918	4.147316	up
ASMM9PARTA045335	H2-K2	0.000081	2.0002196	up
ASMM9PARTA046244	AK171408	0.000374	4.584018	up
ASMM9PARTA007018		0.015436559	2.6748734	up
ASMM9PARTA019679	Gm3892	0.0000114	2.7301052	up
ASMM9PARTA050456	A530053G22Rik	0.000297	4.455251	up
ASMM9PARTA006915		0.0272825	2.5301056	up
ASMM9PARTA011740	Gm14688	0.0000194	2.489045	up
ASMM9PARTA004567		0.0000241	5.1733522	up
ASMM9PARTA011990	Lamr1-ps1	0.0000345	7.180089	up
ASMM9PARTA010121	Gm14162	0.000251	3.2333097	up
ASMM9PARTA048739	AK039017	0.001882068	4.270442	up
ASMM9PARTA048739	AK039017	0.001882068	4.270442	up
DV653038_P1	mouselincRNA1246	0.000417	2.278675	up
DV653038_P1	mouselincRNA1246	0.000417	2.278675	up
DV653038_P1	mouselincRNA1246	0.000417	2.278675	up
DV653038_P1	mouselincRNA1246	0.000417	2.278675	up
DV653038_P1	mouselincRNA1246	0.000417	2.278675	up
DV653038_P1	mouselincRNA1246	0.000417	2.278675	up
ASMM9PARTA008317		0.00078	0.323063677	down
ASMM9PARTA004702		0.045818124	0.479904572	down
ASMM9PARTA045034	Gm4759	0.011067896	0.275980455	down
ASMM9PARTA004200		0.000538	0.307007483	down
MM9LINCRNAEXON11995-_P1	mouselincRNA0004	0.000337	0.449832802	down
ASMM9PARTA014873	Gm16206	0.017974988	0.383945252	down
ASMM9PARTA046074	F630042J09Rik	0.02230519	0.484179156	down
CA559477_P1	humanlincRNA2085	0.00000106	0.345139247	down
MM9LINCRNAEXON10677-_P1	mouselincRNA1108	0.013267624	0.342984529	down
ASMM9PARTA014450	Gm11508	0.00000196	0.251871925	down
ASMM9PARTA014450	Gm11508	0.00000196	0.251871925	down
ASMM9PARTA049723	AK017111	0.0000171	0.240110278	down
ASMM9PARTA051617	AK039014	0.017692	0.436916719	down
ASMM9PARTA004651		0.034654997	0.253662262	down
MM9LINCRNAEXON10155-_P1		0.00000171	0.426029372	down
ASMM9PARTA000135		0.0000154	0.291929029	down
ASMM9PARTA047204	D12Ertd551e	0.00432953	0.488454045	down
ASMM9PARTA000932		0.003065578	0.257932149	down
ASMM9PARTA008248		0.010611367	0.461417705	down

ASMM9PARTA049912	AK041061	0.0000278	0.497772642	down
MM9LINCRNAEXON10863-_P1	mouselincRNA1009	0.000486	0.323871545	down
MM9LINCRNAEXON11127-_P1	mouselincRNA0742	0.0000269	0.437182264	down
ASMM9PARTA045333	3110099E03Rik	0.005419604	0.470902022	down
ASMM9PARTA016600	Gm13150	0.000212	0.497721481	down
ASMM9PARTA016483	Rgsl1	0.012412198	0.476632723	down
MM9LINCRNAEXON10861-_P1	mouselincRNA1009	0.002390658	0.479455339	down
ASMM9PARTA006658		0.023637507	0.200424676	down
ASMM9PARTA017533	A930038B10Rik	0.024687821	0.496752875	down
ASMM9PARTA047203	AK135975	0.0002	0.270557734	down
ASMM9PARTA049557	AK036227	0.00000873	0.232341306	down
ASMM9PARTA049557	AK036227	0.00000873	0.232341306	down
ASMM9PARTA004048		0.00000203	0.162229882	down
ASMM9PARTA048692	AK053505	0.0000165	0.187414059	down
ASMM9PARTA048692	AK053505	0.0000165	0.187414059	down
ASMM9PARTA047030	BC079904	0.01487201	0.39166645	down
ASMM9PARTA051638	Fxy	0.0000206	0.235417255	down
ASMM9PARTA051638	Fxy	0.0000206	0.235417255	down
ASMM9PARTA013130	Gm15035	0.0000211	0.381320264	down
ASMM9PARTA018906	AC122371.1	0.036727384	0.466418781	down
MM9LINCRNAEXON10461+_P1	mouselincRNA1289	0.012286497	0.357422591	down
ASMM9PARTA013774	Gm14204	0.036426283	0.486297758	down
ASMM9PARTA019893		0.005559176	0.241674203	down
ASMM9PARTA000020		0.008395591	0.181439284	down
ASMM9PARTA010105	Ear-ps2	0.0000621	0.242448711	down
ASMM9PARTA004261		0.0000746	0.466873025	down
ASMM9PARTA048135	AK007154	0.018609809	0.409617206	down
ASMM9PARTA048135	AK007154	0.018609809	0.409617206	down
ASMM9PARTA048135	AK007154	0.018609809	0.409617206	down
ASMM9PARTA048135	AK007154	0.018609809	0.409617206	down
ASMM9PARTA048135	AK007154	0.018609809	0.409617206	down
ASMM9PARTA048135	AK007154	0.018609809	0.409617206	down
ASMM9PARTA046300	AK046727	0.00000244	0.489751701	down
ASMM9PARTA004593		0.000183	0.481923832	down
CUST_6_PI426409190	LOC665622	0.001406992	0.472467745	down
CUST_6_PI426409190	LOC665622	0.001406992	0.472467745	down
CUST_6_PI426409190	LOC665622	0.001406992	0.472467745	down
CUST_6_PI426409190	LOC665622	0.001406992	0.472467745	down
MM9LINCRNAEXON11325+_P1		0.0000351	0.038410349	down
ASMM9PARTA007331		0.02422406	0.46455937	down
ASMM9PARTA008226		0.048964903	0.482049327	down
ASMM9PARTA005747		0.0000366	0.329263396	down
ASMM9PARTA010002	Cecr6	0.00000127	0.433035236	down
MM9LINCRNAEXON11848-_P1	mouselincRNA0275	0.001058863	0.369121727	down
BY346886_P1	mouselincRNA1073	0.005346434	0.469310034	down
ASMM9PARTA000308		0.001435583	0.158079508	down
ASMM9PARTA019551		0.010182098	0.207589213	down
ASMM9PARTA013679	Gm14373	0.000291	0.478385651	down
ASMM9PARTA048535	AK122507	0.0000504	0.370215374	down

ASMM9PARTA004828		0.011135699	0.416525222	down
CUST_492_PI426073487	uc.12	0.0000407	0.354797745	down
ASMM9PARTA004412		0.0000825	0.467161512	down
ASMM9PARTA014587	1110050K14Rik	0.049366653	0.497672064	down
ASMM9PARTA005650		0.0000091	0.464386005	down
BG404676_P1	mouselincRNA1540	0.00304584	0.402538731	down
ASMM9PARTA000477		0.04826826	0.452972036	down
ASMM9PARTA001867		0.000189	0.450420159	down
ASMM9PARTA050182	AB294528	0.0000123	0.345900295	down
ASMM9PARTA048103	AK043982	0.0000444	0.384019814	down
ASMM9PARTA047164	AK144886	0.012499584	0.398548185	down
ASMM9PARTA007801		0.000155	0.15379209	down
ASMM9PARTA014733	H19	0.000101	0.463343613	down
ASMM9PARTA006749		0.017441845	0.431024004	down
ASMM9PARTA006749		0.017441845	0.431024004	down
ASMM9PARTA051651	AK038272	0.0000178	0.409547855	down
ASMM9PARTA051651	AK038272	0.0000178	0.409547855	down
ASMM9PARTA007449		0.020689862	0.358001165	down
ASMM9PARTA006132		0.00105592	0.221923759	down
ASMM9PARTA006132		0.00105592	0.221923759	down
ASMM9PARTA006132		0.00105592	0.221923759	down
ASMM9PARTA048083	AK045744	0.001346885	0.106290687	down
ASMM9PARTA008491		0.00092	0.357186179	down
MM9LINCRNAEXON10200+_P1		0.0000268	0.146556941	down
BI853964_P1	mouselincRNA0749	0.0000928	0.143630711	down
ASMM9PARTA001721		0.00131355	0.466297487	down
ASMM9PARTA001455		0.003796017	0.42183095	down
ASMM9PARTA045969	AI507597	0.0000264	0.4715347	down
ASMM9PARTA011123	Gm13295	0.00000432	0.28997286	down
ASMM9PARTA015982	Gm13010	0.002972326	0.472327735	down
ASMM9PARTA011789	Gm12856	0.007483234	0.470690259	down
ASMM9PARTA009220		0.00000458	0.454223121	down
ASMM9PARTA002356		0.0000147	0.407654679	down
ASMM9PARTA045353	Gm4489	0.031756688	0.386736635	down
CN686219_P1	mouselincRNA0474	0.000863	0.381729699	down
CN686219_P1	mouselincRNA0474	0.000863	0.381729699	down
CN686219_P1	mouselincRNA0474	0.000863	0.381729699	down
CN686219_P1	mouselincRNA0474	0.000863	0.381729699	down
CN686219_P1	mouselincRNA0474	0.000863	0.381729699	down
CN686219_P1	mouselincRNA0474	0.000863	0.381729699	down
CN686219_P1	mouselincRNA0474	0.000863	0.381729699	down
CN686219_P1	mouselincRNA0474	0.000863	0.381729699	down
CN686219_P1	mouselincRNA0474	0.000863	0.381729699	down
CN686219_P1	mouselincRNA0474	0.000863	0.381729699	down
ASMM9PARTA049367	AK040035	0.008649284	0.408644198	down
mouselincRNA1010-_P1	mouselincRNA1010	0.03228873	0.337941675	down
MM9LINCRNAEXON11962-_P1		0.0000372	0.497458485	down
ASMM9PARTA019104	AC104908.1	0.001455817	0.259347776	down
ASMM9PARTA046259	Gm4814	0.000223	0.317045119	down

ASMM9PARTA006436		0.018438153	0.465320154	down
ASMM9PARTA017459	Hoxb3os	0.0000851	0.237138104	down
ASMM9PARTA017459	Hoxb3os	0.0000851	0.237138104	down
ASMM9PARTA017459	Hoxb3os	0.0000851	0.237138104	down
BY084980_P1	mouselincRNA0727	0.0000816	0.478134023	down
ASMM9PARTA001734		0.028122596	0.452975401	down
CF425770_P1	humanlincRNA1138	0.000000676	0.399256377	down
mouselincRNA0582-_P1	mouselincRNA0582	0.006611501	0.45220247	down
ASMM9PARTA003240		0.026461126	0.232529046	down
ASMM9PARTA008736		0.004970821	0.444055787	down
ASMM9PARTA008736		0.004970821	0.444055787	down
ASMM9PARTA008228		0.000502	0.408748894	down
ASMM9PARTA008228		0.000502	0.408748894	down
ASMM9PARTA016063	AI854517	0.0000137	0.484768853	down
ASMM9PARTA045941	D830015G02Rik	0.0000921	0.099167214	down
ASMM9PARTA046086		0.001464829	0.122741555	down
ASMM9PARTA048613	L3mbtl4	0.000106	0.400969062	down
ASMM9PARTA016656	1700015O11Rik	0.00000124	0.228541214	down
ASMM9PARTA000311		0.021721914	0.219778626	down
ASMM9PARTA046121		0.01409216	0.444191749	down
ASMM9PARTA003440		0.0000219	0.289629749	down
ASMM9PARTA007489		0.00000204	0.232432707	down
ASMM9PARTA050521	TCRBVbeta5.1/Jbeta 1.5	0.000332	0.475368202	down
ASMM9PARTA008455		0.001356535	0.432946351	down
ASMM9PARTA006997		0.026933365	0.165626134	down
ASMM9PARTA017466	Dleu2	0.0000231	0.445850797	down
ASMM9PARTA001010		0.000000499	0.486175762	down
ASMM9PARTA014368	2410018L13Rik	0.0000928	0.152342649	down
ASMM9PARTA050350	4631427C17Rik	0.000133	0.482290765	down
ASMM9PARTA050350	4631427C17Rik	0.000133	0.482290765	down
ASMM9PARTA050350	4631427C17Rik	0.000133	0.482290765	down
ASMM9PARTA011079	Gm8688	0.010357689	0.488792477	down
ASMM9PARTA047495	EG218444	0.026615096	0.45159347	down
ASMM9PARTA045493	D6Ertd474e	0.00000335	0.486300431	down
ASMM9PARTA012907	Gm11299	0.000332	0.428477047	down
ASMM9PARTA005833		0.000342	0.426909379	down
ASMM9PARTA005384		0.000324	0.455417185	down
ASMM9PARTA007850		0.00000185	0.304420156	down
ASMM9PARTA019683		0.003445593	0.144617807	down
ASMM9PARTA049031	AK184713	0.0000212	0.272724273	down
ASMM9PARTA005870		0.016775636	0.472426452	down
MM9LINCRNAEXON10601+_P1	mouselincRNA1220	0.000366	0.480150737	down
MM9LINCRNAEXON12015-_P1	mouselincRNA0025	0.000015	0.222110279	down
ASMM9PARTA047748	AK083927	0.001108011	0.369544575	down
CJ133294_P1	mouselincRNA0439	0.019555258	0.413231969	down
ASMM9PARTA048698	Glis3	0.015152084	0.409813156	down
MM9LINCRNAEXON10306-_P1	mouselincRNA1357	0.009085594	0.458682984	down
ASMM9PARTA000786		0.011288716	0.392976041	down

ASMM9PARTA046085		0.011937083	0.178640588	down
ASMM9PARTA003595		0.012592565	0.433856213	down
ASMM9PARTA003595		0.012592565	0.433856213	down
ASMM9PARTA050764	U80893	0.000000389	0.161642364	down
CUST_185_PI426409190		0.000113	0.275998561	down
ASMM9PARTA009159		0.048507366	0.47605258	down
ASMM9PARTA045623	D030047H15Rik	0.03904449	0.383334399	down
ASMM9PARTA045623	D030047H15Rik	0.03904449	0.383334399	down
ASMM9PARTA045623	D030047H15Rik	0.03904449	0.383334399	down
ASMM9PARTA045623	D030047H15Rik	0.03904449	0.383334399	down
ASMM9PARTA049201	Hltf	0.0142338	0.387478208	down
ASMM9PARTA049201	Hltf	0.0142338	0.387478208	down
ASMM9PARTA001382		0.000245	0.492685784	down
ASMM9PARTA017928	7SK.121	0.000224	0.479489248	down
MM9LINCRNAEXON11779-_P1	mouselincRNA0246	0.000892	0.435011557	down
ASMM9PARTA050215	AK040684	0.001922275	0.494779825	down
ASMM9PARTA004517		0.0000742	0.419732868	down
ASMM9PARTA015110	1700016P03Rik	0.00000336	0.318547566	down
CUST_787_PI426073487	uc.308	0.000352	0.296559641	down
CUST_787_PI426073487	uc.308	0.000352	0.296559641	down
ASMM9PARTA011864	Gm13086	0.00000117	0.311332949	down
ASMM9PARTA049722	LOC433791	0.000000541	0.137501915	down
ASMM9PARTA049722	LOC433791	0.000000541	0.137501915	down
ASMM9PARTA049722	LOC433791	0.000000541	0.137501915	down
ASMM9PARTA049722	LOC433791	0.000000541	0.137501915	down
ASMM9PARTA049722	LOC433791	0.000000541	0.137501915	down
ASMM9PARTA049722	LOC433791	0.000000541	0.137501915	down
ASMM9PARTA049722	LOC433791	0.000000541	0.137501915	down
MM9LINCRNAEXON11245+_P1	mouselincRNA0732	0.0000577	0.483223588	down
ASMM9PARTA007901		0.00012	0.381145001	down
MM9LINCRNAEXON10210-_P1	mouselincRNA1477	0.00579754	0.426008501	down
ASMM9PARTA015002	2700023E23Rik	0.00161172	0.443211725	down
MM9LINCRNAEXON11847-_P1	mouselincRNA0275	0.000658	0.470935509	down
ASMM9PARTA049513	AK080176	0.00000908	0.181798402	down
ASMM9PARTA001264		0.023612143	0.410594454	down
ASMM9PARTA008731		0.000272	0.379507794	down
ASMM9PARTA006027		0.005879375	0.497581901	down
ASMM9PARTA000840		0.008521841	0.259091307	down
ASMM9PARTA001040		0.00474639	0.477960204	down
ASMM9PARTA051561		0.000000825	0.05542597	down
ASMM9PARTA050490	H4	0.0000341	0.482699681	down
ASMM9PARTA001112		0.002098108	0.302450963	down
ASMM9PARTA047242	AK019801	0.00219174	0.357155	down
ASMM9PARTA001387		0.02446659	0.416824747	down
ASMM9PARTA000104		0.010006199	0.346658241	down
ASMM9PARTA002587		0.00000629	0.358638945	down
CUST_131_PI426073487	uc.133	0.013562259	0.463886914	down
ASMM9PARTA008980		0.000342	0.441418667	down
ASMM9PARTA005120		0.0000191	0.267256341	down

ASMM9PARTA009176		0.006131728	0.132038825	down
ASMM9PARTA017680	Gm6038	0.015924927	0.491778254	down
ASMM9PARTA045652	Klk1b7-ps	0.001129593	0.399895052	down
AV461233_P1	mouselincRNA1513	0.003009132	0.478287608	down
CUST_718_PI426073487	uc.239	0.00000594	0.435599225	down
ASMM9PARTA019411	AL672241.1	0.000159	0.467324441	down
humanlincRNA1165-_P1	humanlincRNA1165	0.001016832	0.35399819	down
ASMM9PARTA007177		0.03998618	0.386111955	down
MM9LINCRNAEXON10524+_P1		0.0000826	0.020699541	down
MM9LINCRNAEXON11395-_P1		0.000012	0.452577565	down
ASMM9PARTA004207		0.017134914	0.458882394	down
ASMM9PARTA050789	1700073E17Rik	0.004283616	0.431445658	down
MM9LINCRNAEXON11664-_P1	mouselincRNA0308	0.003317551	0.271664519	down
ASMM9PARTA005200		0.00000197	0.420034574	down
humanlincRNA2296-_P1	humanlincRNA2296	0.002244888	0.269267612	down
MM9LINCRNAEXON10678+_P1	mouselincRNA1108	0.021666337	0.482351064	down
ASMM9PARTA050513	Ppfia3	0.0000849	0.42885435	down
MM9LINCRNAEXON11536+_P1	mouselincRNA0380	0.002718529	0.449363477	down
ASMM9PARTA008371		0.008770536	0.421123363	down
ASMM9PARTA008614		0.002326884	0.405194759	down
ASMM9PARTA003080		0.0000525	0.441217634	down
ASMM9PARTA014713	C030005K06Rik	0.015463608	0.305064278	down
ASMM9PARTA014713	C030005K06Rik	0.015463608	0.305064278	down
ASMM9PARTA014713	C030005K06Rik	0.015463608	0.305064278	down
ASMM9PARTA002908		0.001910946	0.383943041	down
MM9LINCRNAEXON10967+_P1		0.000729	0.216779679	down
ASMM9PARTA011719	Gm11862	0.006367177	0.420677582	down
ASMM9PARTA000239		0.000000336	0.03475139	down
ASMM9PARTA000239		0.000000336	0.03475139	down
ASMM9PARTA050235	AK138476	0.0031879	0.490365904	down
ASMM9PARTA049793	BC082597	0.01580295	0.437353678	down
ASMM9PARTA044635	Psg-ps1	0.032420322	0.484841151	down
humanlincRNA2257-_P1	humanlincRNA2257	0.001692752	0.237633527	down
ASMM9PARTA001071		0.00520089	0.321642818	down
ASMM9PARTA050576	AK158295	0.007278598	0.283253886	down
ASMM9PARTA000019		0.00095	0.082972555	down
MM9LINCRNAEXON10652-_P1	mouselincRNA1089	0.03774847	0.489068057	down
MM9LINCRNAEXON10366+_P1		0.028418727	0.491167	down
MM9LINCRNAEXON10174-_P1	mouselincRNA1432	0.000262	0.349179531	down
MM9LINCRNAEXON10365-_P1	mouselincRNA1405	0.032103196	0.444666382	down
ASMM9PARTA047559	AK042724	0.000595	0.491072547	down
humanlincRNA0791+_P1	humanlincRNA0791	0.015497944	0.468182943	down
ASMM9PARTA013573	Gm12929	0.0000251	0.386969289	down
ASMM9PARTA002701		0.000999	0.484212119	down
humanlincRNA0095-_P1	humanlincRNA0095	0.021872127	0.499637213	down
ASMM9PARTA007172		0.03535574	0.368568888	down
CUST_40_PI426073487	uc.42	0.019246697	0.487804212	down
ASMM9PARTA008140		0.042193063	0.429057674	down
ASMM9PARTA008140		0.042193063	0.429057674	down

ASMM9PARTA047980	TCR-V[alpha]new	0.039930906	0.357050955	down
ASMM9PARTA050956	BC021774	0.001269584	0.265815547	down
ASMM9PARTA050956	BC021774	0.001269584	0.265815547	down
ASMM9PARTA050956	BC021774	0.001269584	0.265815547	down
ASMM9PARTA003098		0.0000151	0.459038186	down
ASMM9PARTA015793	Gm13026	0.013562483	0.426883136	down
CUST_103_PI426073487	uc.105	0.04360546	0.300955027	down
CUST_103_PI426073487	uc.105	0.04360546	0.300955027	down
ASMM9PARTA007517		0.03878358	0.457857806	down
ASMM9PARTA005692		0.014156694	0.291304689	down
ASMM9PARTA047498	AK136919	0.002039174	0.476394033	down
ASMM9PARTA001652		0.006812644	0.43673529	down
ASMM9PARTA008294		0.043325778	0.386490607	down
ASMM9PARTA017930	7SK.169	0.035324853	0.232516989	down
ASMM9PARTA018321	Gm16551	0.03162796	0.402431523	down
ASMM9PARTA000071		0.000868	0.477782014	down
ASMM9PARTA001044		0.002646316	0.432725057	down
ASMM9PARTA001044		0.002646316	0.432725057	down
ASMM9PARTA001044		0.002646316	0.432725057	down
ASMM9PARTA001044		0.002646316	0.432725057	down
ASMM9PARTA001044		0.002646316	0.432725057	down
MM9LINCRNAEXON10675-_P1	mouselincRNA1108	0.007772063	0.284974167	down
ASMM9PARTA001873		0.012272421	0.303759381	down
ASMM9PARTA018910	RP24-354E11.1	0.0000019	0.472389986	down
ASMM9PARTA008053		0.009012941	0.423539836	down
ASMM9PARTA015543	Gm16441	0.001360943	0.488982611	down
ASMM9PARTA048438	AK138161	0.024824593	0.359236049	down
ASMM9PARTA048438	AK138161	0.024824593	0.359236049	down
ASMM9PARTA019335	RP24-356D15.2	0.000209	0.478013277	down
ASMM9PARTA017010	B630019A10Rik	0.00064	0.374752336	down
ASMM9PARTA047382	AK006991	0.027023459	0.355345873	down
ASMM9PARTA003369		0.001440315	0.408838583	down
ASMM9PARTA013415	Gm14649	0.03413932	0.236582557	down
ASMM9PARTA002592		0.007200393	0.469146928	down
MM9LINCRNAEXON11367-_P1	mouselincRNA0586	0.000382	0.412672632	down
ASMM9PARTA017429	Gm7076	0.03618517	0.451580113	down
ASMM9PARTA047473	AK139027	0.003025994	0.279618811	down
ASMM9PARTA018457	Gm16556	0.008996647	0.239018321	down
MM9LINCRNAEXON11480-_P1		0.001316092	0.490732613	down
ASMM9PARTA008027		0.004890168	0.430078799	down
ASMM9PARTA008027		0.004890168	0.430078799	down
mouselincRNA0052+_P1	mouselincRNA0052	0.004712214	0.28765785	down
ASMM9PARTA016152	Gm8289	0.008333561	0.34473679	down
ASMM9PARTA008473		0.000726	0.322662279	down
ASMM9PARTA018315	9930104L06Rik	0.003590496	0.477222527	down
ASMM9PARTA018315	9930104L06Rik	0.003590496	0.477222527	down
ASMM9PARTA002911		0.010312071	0.386685191	down
ASMM9PARTA018610	RP23-189E15.2	0.013897288	0.49534794	down
ASMM9PARTA015481	C030037D09Rik	0.00000583	0.023873157	down

ASMM9PARTA015481	C030037D09Rik	0.00000583	0.023873157	down
ASMM9PARTA045845		0.009141183	0.092889931	down
MM9LINCRNAEXON11914+_P1	mouselincRNA0176	0.00000837	0.436005947	down
ASMM9PARTA001738		0.0000028	0.033517602	down
ASMM9PARTA000825		0.001556125	0.298312954	down
ASMM9PARTA006500		0.003987778	0.495960476	down
ASMM9PARTA004842		0.029647207	0.435426718	down
MM9LINCRNAEXON10149+_P1	mouselincRNA1571	0.000667	0.459404872	down
ASMM9PARTA045672	6330407A03Rik	0.000148	0.388389032	down
ASMM9PARTA045672	6330407A03Rik	0.000148	0.388389032	down
ASMM9PARTA014773	1700027L20Rik	0.0000427	0.221144966	down
CUST_932_PI426073487	uc.453	0.002485754	0.43048124	down
ASMM9PARTA046864	Ywhae	0.0000289	0.381040695	down
ASMM9PARTA047397	Slc38a6	0.004971354	0.253074091	down
ASMM9PARTA047397	Slc38a6	0.004971354	0.253074091	down
ASMM9PARTA002040		0.001330059	0.330678241	down
ASMM9PARTA009851	Gm10087	0.001494038	0.485861409	down
ASMM9PARTA006426		0.001173553	0.17018434	down
ASMM9PARTA045115	4933432I09Rik	0.000175	0.347577282	down
CUST_138_PI426409190		0.000614	0.484337424	down
MM9LINCRNAEXON10703-_P1	mouselincRNA1121	0.001971603	0.451543165	down
ASMM9PARTA007243		0.000372	0.496123096	down
ASMM9PARTA019094	AL670236.3	0.000487	0.415589891	down
ASMM9PARTA015161	AI838599	0.001767402	0.441870714	down
ASMM9PARTA017307	Gm15351	0.024890687	0.445217302	down
CUST_700_PI426073487	uc.221	0.003391748	0.395589494	down
ASMM9PARTA005418		0.013872084	0.487288875	down
ASMM9PARTA005418		0.013872084	0.487288875	down
BQ128792_P1	humanlincRNA1677	0.0000354	0.162894739	down
ASMM9PARTA049459	AK136882	0.001072609	0.364516598	down
ASMM9PARTA007323		0.0358005	0.484621506	down
ASMM9PARTA015587	Gm14453	0.003357813	0.473975386	down
ASMM9PARTA008144		0.011306209	0.306258415	down
ASMM9PARTA050807	AK038589	0.001230427	0.393178974	down
ASMM9PARTA001615		0.0000583	0.34569515	down
ASMM9PARTA006877		0.011310122	0.349208198	down
ASMM9PARTA051573	AK048941	0.004146529	0.492462662	down
ASMM9PARTA051573	AK048941	0.004146529	0.492462662	down
ASMM9PARTA051573	AK048941	0.004146529	0.492462662	down
ASMM9PARTA051573	AK048941	0.004146529	0.492462662	down
ASMM9PARTA004356		0.006129209	0.488213715	down
ASMM9PARTA015758	Gm11789	0.00011	0.238154193	down
humanlincRNA2149-_P1	humanlincRNA2149	0.025311101	0.400653337	down
ASMM9PARTA015015	H2-Ea-ps	0.000103	0.021976573	down
ASMM9PARTA014858	A830009L08Rik	0.000015	0.449145097	down
ASMM9PARTA011232	Gm14783	0.00776232	0.451325636	down
ASMM9PARTA015585	Gm14344	0.045442495	0.499157123	down
ASMM9PARTA008461		0.000609	0.224526372	down
ASMM9PARTA051102	AK015772	0.006911601	0.310632005	down

ASMM9PARTA001540		0.014438592	0.47958284	down
humanlincRNA2212-_P1	humanlincRNA2212	0.03744507	0.487002773	down
ASMM9PARTA004677		0.011457424	0.396525485	down
ASMM9PARTA005377		0.011243109	0.426304124	down
ASMM9PARTA005559		0.045271896	0.471589937	down
ASMM9PARTA051330	Ttc13	0.00000314	0.339876581	down
ASMM9PARTA009639	Setd1b	0.002071896	0.353694175	down
ASMM9PARTA045562	Deaf1	0.005822519	0.180606444	down
ASMM9PARTA045562	Deaf1	0.005822519	0.180606444	down
ASMM9PARTA045562	Deaf1	0.005822519	0.180606444	down
ASMM9PARTA045562	Deaf1	0.005822519	0.180606444	down
MM9LINCRNAEXON10699+_P1	mouselincRNA1121	0.0000132	0.491848206	down
MM9LINCRNAEXON12072+_P1	mouselincRNA0095	0.000307	0.380424699	down
ASMM9PARTA049498	AK005639	0.000691	0.399818275	down
ASMM9PARTA017358	Gm13883	0.000385	0.407199001	down
ASMM9PARTA017519	6530402F18Rik	0.022502145	0.493795167	down
ASMM9PARTA050491	AK018014	0.0000515	0.498346337	down
ASMM9PARTA007912		0.015671153	0.186919417	down
MM9LINCRNAEXON11862-_P1	mouselincRNA0283	0.011061511	0.469457737	down
ASMM9PARTA009532	4930515G13Rik	0.010874454	0.391014304	down
humanlincRNA1527+_P1	humanlincRNA1527	0.02555595	0.452410488	down
ASMM9PARTA008669		0.008589326	0.268293167	down
ASMM9PARTA006629		0.000138	0.384235007	down
MM9LINCRNAEXON10128-_P1	mouselincRNA1558	0.000393	0.469900014	down
ASMM9PARTA003873		0.001601955	0.349783769	down
ASMM9PARTA012580	Gm4746	0.005804983	0.316864783	down
ASMM9PARTA012491	Gm8084	0.023236088	0.399023685	down
MM9LINCRNAEXON10572+_P1		0.00000394	0.218752453	down
ASMM9PARTA002100		0.000491	0.387069854	down
ASMM9PARTA006636		0.001580883	0.366134734	down
ASMM9PARTA005279		0.012363432	0.454546529	down
ASMM9PARTA049296	AK042718	0.001576831	0.415977877	down
ASMM9PARTA002205		0.000413	0.303456273	down
MM9LINCRNAEXON10133-_P1		0.000366	0.079783379	down
ASMM9PARTA048644	mKIAA0007	0.000323	0.499595977	down
ASMM9PARTA048647	AK053722	0.020272028	0.489608788	down
ASMM9PARTA005267		0.018825293	0.473144888	down
ASMM9PARTA011388	Gm11566	0.0000186	0.423416956	down
humanlincRNA1524-_P1	humanlincRNA1524	0.017975576	0.318420096	down
ASMM9PARTA045134	Gm5468	0.000463	0.451035578	down
mouselincRNA0052-_P1	mouselincRNA0052	0.00000179	0.476686684	down
mouselincRNA0166-_P1	mouselincRNA0166	0.045095038	0.295663043	down
ASMM9PARTA009224	1700027L20Rik	0.00000471	0.240607988	down
ASMM9PARTA011640	Gm14631	0.000338	0.374404145	down
ASMM9PARTA009329		0.013398703	0.452567775	down
ASMM9PARTA008950		0.002322083	0.154454801	down
humanlincRNA0588+_P1	humanlincRNA0588	0.016839711	0.346574225	down
BG808609_P1	humanlincRNA0438	0.003797978	0.489305135	down
mouselincRNA1656+_P1	mouselincRNA1656	0.026321959	0.456037526	down

ASMM9PARTA010572	Gm12378	0.010290162	0.482933283	down
ASMM9PARTA004342		0.003810819	0.457666721	down
ASMM9PARTA005983		0.023021948	0.331175636	down
ASMM9PARTA017566	Gm15344	0.0003	0.405236071	down
CF586746_P1	humanlincRNA0707	0.012940467	0.469537796	down
CF586746_P1	humanlincRNA0707	0.012940467	0.469537796	down
CF586746_P1	humanlincRNA0707	0.012940467	0.469537796	down
CF586746_P1	humanlincRNA0707	0.012940467	0.469537796	down
CF586746_P1	humanlincRNA0707	0.012940467	0.469537796	down
CF586746_P1	humanlincRNA0707	0.012940467	0.469537796	down
CF586746_P1	humanlincRNA0707	0.012940467	0.469537796	down
CF586746_P1	humanlincRNA0707	0.012940467	0.469537796	down
CF586746_P1	humanlincRNA0707	0.012940467	0.469537796	down
CF586746_P1	humanlincRNA0707	0.012940467	0.469537796	down
mouselincRNA0750-_P1	mouselincRNA0750	0.000155	0.330458433	down
MM9LINCRNAEXON10551+_P1	mouselincRNA1185	0.0000325	0.300137145	down
ASMM9PARTA004866		0.006055214	0.350850381	down
ASMM9PARTA004866		0.006055214	0.350850381	down
ASMM9PARTA050339	BC100494	0.000666	0.349061778	down
ASMM9PARTA047110	BC027544	0.000135	0.464182215	down
ASMM9PARTA047110	BC027544	0.000135	0.464182215	down
ASMM9PARTA005774		0.000115	0.445953949	down
ASMM9PARTA003686		0.006890012	0.404503843	down
ASMM9PARTA001544		0.0000822	0.276324961	down
ASMM9PARTA018908	AC124108.1	0.0000879	0.289037139	down
ASMM9PARTA048969	Ahnak	0.028780313	0.499330473	down
ASMM9PARTA048969	Ahnak	0.028780313	0.499330473	down
ASMM9PARTA008853		0.00046	0.302225029	down
ASMM9PARTA009320		0.011067352	0.414752583	down
ASMM9PARTA047731	CSA	0.004093475	0.492028353	down
ASMM9PARTA047731	CSA	0.004093475	0.492028353	down
ASMM9PARTA000534		0.0000225	0.277677212	down
ASMM9PARTA050559	TCR-beta chain	0.000000571	0.486543591	down
ASMM9PARTA045691	C030023E24Rik	0.000046	0.422710243	down
ASMM9PARTA015780	Gm16899	0.00000338	0.47927787	down
ASMM9PARTA048522	AK008384	0.0000548	0.498469748	down
ASMM9PARTA048468	Adamts10	0.043763135	0.447672061	down
ASMM9PARTA011523	Olfr1315-ps1	0.000994	0.21948924	down
ASMM9PARTA002507		0.0000234	0.450117931	down
ASMM9PARTA002507		0.0000234	0.450117931	down
ASMM9PARTA002507		0.0000234	0.450117931	down
ASMM9PARTA007137		0.000879	0.344811107	down
mouselincRNA0499+_P1	mouselincRNA0499	0.0000318	0.454865514	down
ASMM9PARTA017215	1500002F19Rik	0.000205	0.346175139	down
ASMM9PARTA018399	AC102317.1	0.003320135	0.46534367	down
BC156060_P1	mouselincRNA1038	0.000133	0.47322247	down
BC156060_P1	mouselincRNA1038	0.000133	0.47322247	down
BC156060_P1	mouselincRNA1038	0.000133	0.47322247	down
BC156060_P1	mouselincRNA1038	0.000133	0.47322247	down

BC156060_P1	mouselincRNA1038	0.000133	0.47322247	down
MM9LINCRNAEXON11766+_P1	mouselincRNA0239	0.000215	0.297064465	down
ASMM9PARTA000466		0.036914453	0.290658554	down
MM9LINCRNAEXON10104+_P1		0.000593	0.363998774	down
ASMM9PARTA015299	Gm12940	0.00000414	0.495207432	down
ASMM9PARTA045068	4930558C23Rik	0.013556981	0.39604867	down
MM9LINCRNAEXON10083+_P1	mouselincRNA1506	0.0000198	0.460631443	down
MM9LINCRNAEXON11272+_P1	mouselincRNA0626	0.003659791	0.480359309	down
ASMM9PARTA010963	Gm12941	0.000181	0.359188564	down
ASMM9PARTA006968		0.016136985	0.302468756	down
ASMM9PARTA017569	Gm12081	0.000749	0.115529178	down
ASMM9PARTA011163	Gpr144	0.009776521	0.416087715	down
mouselincRNA0570-_P1	mouselincRNA0570	0.00000246	0.48612561	down
ASMM9PARTA008696		0.000174	0.399640164	down
ASMM9PARTA014023	Gm13147	0.000285	0.474448865	down
ASMM9PARTA045902	Gm16287	0.0391789	0.401970378	down
ASMM9PARTA007823		0.0000734	0.324226376	down
ASMM9PARTA014881	Mir17hg	0.000000729	0.178390521	down
ASMM9PARTA017438	Gm11940	0.0000252	0.33986243	down
ASMM9PARTA003703		0.000145	0.393924114	down
ASMM9PARTA012978	Gm15188	0.001961787	0.343265032	down
humanlincRNA0219+_P1	humanlincRNA0219	0.000137	0.338530648	down
ASMM9PARTA048267	Tcte2	0.000386	0.414836202	down
ASMM9PARTA048267	Tcte2	0.000386	0.414836202	down
ASMM9PARTA000047		0.000103	0.077911547	down
ASMM9PARTA019614	CT571259.1	0.005521244	0.384955271	down
MM9LINCRNAEXON11266+_P1	mouselincRNA0611	0.000302	0.482072449	down
CUST_417_PI426073487	uc.419	0.003995812	0.361092135	down
CUST_417_PI426073487	uc.419	0.003995812	0.361092135	down
ASMM9PARTA003255		0.00000375	0.451977973	down
ASMM9PARTA046888	AK140919	0.04342912	0.412120123	down
ASMM9PARTA046888	AK140919	0.04342912	0.412120123	down
ASMM9PARTA046888	AK140919	0.04342912	0.412120123	down
ASMM9PARTA046888	AK140919	0.04342912	0.412120123	down
MM9LINCRNAEXON11907+_P1	mouselincRNA0173	0.0000184	0.359910271	down
CUST_338_PI426073487	uc.340	0.008479083	0.447244973	down
ASMM9PARTA008362		0.045565672	0.464115323	down
ASMM9PARTA045931	Lrch4-sap25	0.010437384	0.392545252	down
ASMM9PARTA045931	Lrch4-sap25	0.010437384	0.392545252	down
ASMM9PARTA045931	Lrch4-sap25	0.010437384	0.392545252	down
CUST_377_PI426073487	uc.379	0.000755	0.253667126	down
ASMM9PARTA012487	Gm14054	0.003452617	0.144087139	down
ASMM9PARTA014754	Gm6277	0.000528	0.161877714	down
ASMM9PARTA014754	Gm6277	0.000528	0.161877714	down
ASMM9PARTA009187		0.000573	0.459405379	down
MM9LINCRNAEXON11776-_P1	mouselincRNA0243	0.011599883	0.444094754	down
ASMM9PARTA002906		0.0000429	0.410777537	down
humanlincRNA0034-_P1	humanlincRNA0034	0.013223419	0.492998388	down
ASMM9PARTA008341		0.037641805	0.318367838	down

ASMM9PARTA000224		0.034341156	0.323338969	down
ASMM9PARTA000224		0.034341156	0.323338969	down
ASMM9PARTA002055		0.000447	0.151165501	down
ASMM9PARTA003639		0.00019	0.404253229	down
humanlincRNA1082+_P1	humanlincRNA1082	0.004578662	0.38300526	down
ASMM9PARTA000149		0.010409152	0.19913694	down
ASMM9PARTA049191	A230067G21Rik	0.010616894	0.384798786	down
CUST_179_PI426073487	uc.181	0.018138608	0.397397302	down
ASMM9PARTA050392	M34473	0.000000629	0.122071102	down
ASMM9PARTA002466		0.00036	0.437972956	down
ASMM9PARTA004363		0.026345985	0.204381907	down
ASMM9PARTA011773	Gm12135	0.04528992	0.481581089	down
ASMM9PARTA002294		0.0000646	0.320819697	down
mouselincRNA0464-_P1	mouselincRNA0464	0.004958587	0.442761753	down
ASMM9PARTA006892		0.023778234	0.417326651	down
ASMM9PARTA012774	Gm15428	0.000108	0.491206302	down
ASMM9PARTA000934		0.00000437	0.412450614	down
ASMM9PARTA047992	AK043393	0.000142	0.406322442	down
ASMM9PARTA007256		0.010065827	0.452734926	down
ASMM9PARTA009861	Gm14164	0.005539357	0.4140521	down
ASMM9PARTA048582	BC030050	0.000128	0.368828068	down
ASMM9PARTA048582	BC030050	0.000128	0.368828068	down
ASMM9PARTA008024		0.026894582	0.300904395	down
ASMM9PARTA045716	1600029O15Rik	0.02185933	0.445340628	down
ASMM9PARTA045716	1600029O15Rik	0.02185933	0.445340628	down
ASMM9PARTA045716	1600029O15Rik	0.02185933	0.445340628	down
ASMM9PARTA045716	1600029O15Rik	0.02185933	0.445340628	down
ASMM9PARTA045716	1600029O15Rik	0.02185933	0.445340628	down
ASMM9PARTA005021		0.034613617	0.427891875	down
ASMM9PARTA005021		0.034613617	0.427891875	down
MM9LINCRNAEXON10013+_P1	mouselincRNA1614	0.008607643	0.329376056	down
ASMM9PARTA005194		0.00000746	0.486538407	down
ASMM9PARTA014192	4930511A02Rik	0.008606721	0.395560654	down
ASMM9PARTA047841	AK086741	0.000021	0.437520383	down
ASMM9PARTA009136		0.000695	0.100548957	down
MM9LINCRNAEXON10204-_P1	mouselincRNA1471	0.000153	0.161505385	down
ASMM9PARTA005458		0.000218	0.482747404	down
ASMM9PARTA051821		0.012466883	0.279174075	down
ASMM9PARTA012454	Gm13230	0.000607	0.049574723	down
ASMM9PARTA003479		0.001283588	0.44298166	down
ASMM9PARTA007030		0.000224	0.476168708	down
ASMM9PARTA048949	AK170301	0.000116	0.058740084	down
CUST_149_PI426073487	uc.151	0.01611484	0.395704438	down
ASMM9PARTA014138	Gm15345	0.020396754	0.465242652	down
CUST_30_PI426073487	uc.31	0.011659533	0.49755452	down
MM9LINCRNAEXON11324+_P1		0.005612009	0.479999846	down
ASMM9PARTA049352	BC038520	0.000749	0.488195077	down
ASMM9PARTA019132	9230115E21Rik	0.0000029	0.05388922	down
ASMM9PARTA051652	AK136006	0.001508328	0.397145319	down

ASMM9PARTA016802	1700029M20Rik	0.030142656	0.445221544	down
ASMM9PARTA046610	BC065048	0.00000204	0.407713449	down
ASMM9PARTA000310		0.002698028	0.128770016	down
MM9LINCRNAEXON10816-_P1		0.00000897	0.490901991	down
BY300155_P1	humanlincRNA1943	0.004361909	0.476623137	down
CUST_161_PI426409190		0.0000796	0.335716856	down
ASMM9PARTA050748	AK014378	0.020210154	0.400280356	down
ASMM9PARTA015195	0610005C13Rik	0.001260763	0.458477609	down
ASMM9PARTA046987	AK172386	0.00000967	0.488294077	down
ASMM9PARTA046987	AK172386	0.00000967	0.488294077	down
ASMM9PARTA018235	7SK.234	0.000729	0.442379417	down
ASMM9PARTA005211		0.00000119	0.127643116	down
ASMM9PARTA019009	AC154675.1	0.011399932	0.427618328	down
ASMM9PARTA006534		0.0000234	0.494633963	down
ASMM9PARTA047508	mKIAA4205	0.000141	0.472979598	down
ASMM9PARTA047508	mKIAA4205	0.000141	0.472979598	down
CUST_699_PI426073487	uc.220	0.003879945	0.346881399	down
ASMM9PARTA006060		0.006457548	0.379811298	down
ASMM9PARTA015401	4930583K01Rik	0.000534	0.395203775	down
ASMM9PARTA009296	Gm8615	0.00000297	0.382410845	down
ASMM9PARTA018654	Ear-ps9	0.00594653	0.264196325	down
ASMM9PARTA048567	wtap	0.000068	0.358985736	down
humanlincRNA0849-_P1	humanlincRNA0849	0.008020902	0.344298257	down
ASMM9PARTA051806	Smcy	0.000225	0.465576268	down
CUST_223_PI426073487	uc.225	0.020562151	0.49322112	down
CUST_223_PI426073487	uc.225	0.020562151	0.49322112	down
ASMM9PARTA014337	Gm16867	0.00000229	0.349896831	down
ASMM9PARTA014337	Gm16867	0.00000229	0.349896831	down
ASMM9PARTA014337	Gm16867	0.00000229	0.349896831	down
ASMM9PARTA014337	Gm16867	0.00000229	0.349896831	down
ASMM9PARTA045609	Gm10649	0.005296455	0.431069136	down
ASMM9PARTA050130	AK152437	0.000239	0.474923787	down
ASMM9PARTA015586	Gm16295	0.000153	0.457485715	down
mouselincRNA0963+_P1	mouselincRNA0963	0.002121049	0.30314106	down
ASMM9PARTA014230	C030037D09Rik	0.0000309	0.183574567	down
CF545245_P1	humanlincRNA1294	0.003085945	0.375109673	down
ASMM9PARTA010625	Gm15087	0.001002297	0.23369766	down
ASMM9PARTA007684		0.012269583	0.467972697	down
ASMM9PARTA015518	Gm15606	0.001584072	0.425599975	down
ASMM9PARTA006932		0.000701	0.443333156	down
ASMM9PARTA009357	H2-Ea-ps	0.000611	0.309340091	down
ASMM9PARTA016911	Gm15246	0.002087223	0.496734468	down
ASMM9PARTA015172	Gm13257	0.03641189	0.449612046	down
ASMM9PARTA050692	Mrmt1	0.0000492	0.438261586	down
ASMM9PARTA005609		0.010279777	0.417379655	down
ASMM9PARTA014533	Gm16731	0.006665989	0.329612427	down
ASMM9PARTA014533	Gm16731	0.006665989	0.329612427	down
ASMM9PARTA014533	Gm16731	0.006665989	0.329612427	down
ASMM9PARTA018464	Gm12759	0.00000803	0.489419317	down

mouselincRNA1170-_P1	mouselincRNA1170	0.024690907	0.42585912	down
MM9LINCRNAEXON10352-_P1		0.0000558	0.440884255	down
ASMM9PARTA045668	Hhatl	0.00600032	0.211631254	down
ASMM9PARTA048570	AK018977	0.0000245	0.492678599	down
mouselincRNA1407+_P1	mouselincRNA1407	0.03384627	0.450267463	down
ASMM9PARTA013915	Gm11648	0.0000569	0.139955827	down
ASMM9PARTA013915	Gm11648	0.0000569	0.139955827	down
CUST_380_PI426073487	uc.382	0.013810853	0.362283321	down
MM9LINCRNAEXON11889+_P1	mouselincRNA0159	0.000143	0.273824123	down
ASMM9PARTA002161		0.000000357	0.160043378	down
ASMM9PARTA012184	Xlr3e-ps	0.0000157	0.453576862	down
mouselincRNA1555+_P1	mouselincRNA1555	0.017862892	0.378975385	down
ASMM9PARTA013285	Gm14848	0.001688507	0.447741874	down
ASMM9PARTA003746		0.000406	0.360871055	down
ASMM9PARTA045912		0.000365	0.076809517	down
ASMM9PARTA045222	AI450353	0.000000561	0.125600174	down
ASMM9PARTA014504	8430419K02Rik	0.04490643	0.385025629	down
AA797690_P1	mouselincRNA1236	0.043949436	0.336325736	down
MM9LINCRNAEXON10076-_P1	mouselincRNA1506	0.007722864	0.483262447	down
ASMM9PARTA009966	Olfr663	0.000616	0.479172067	down
ASMM9PARTA006074		0.00049	0.174306552	down
MM9LINCRNAEXON10276-_P1	mouselincRNA1493	0.044340193	0.400187544	down
ASMM9PARTA013872	Gm11985	0.008604638	0.359987141	down
ASMM9PARTA004494		0.006725606	0.441619902	down
ASMM9PARTA001713		0.001527407	0.486180442	down
ASMM9PARTA047429	AK035761	0.001191142	0.395658002	down
MM9LINCRNAEXON11073+_P1	mouselincRNA0818	0.037518453	0.41061772	down
ASMM9PARTA007253		0.00596165	0.468153178	down
MM9LINCRNAEXON12078-_P1	mouselincRNA0097	0.0000717	0.406551612	down
ASMM9PARTA013810	1500026H17Rik	0.000101	0.381765141	down
ASMM9PARTA051476	AK043361	0.0000163	0.159011736	down
ASMM9PARTA009937	1700026J12Rik	0.014855156	0.353994782	down
CUST_416_PI426073487	uc.418	0.0000185	0.405813358	down
CUST_416_PI426073487	uc.418	0.0000185	0.405813358	down
BB202824_P1	mouselincRNA0958	0.00022	0.301847363	down
ASMM9PARTA009842	Fzd10	0.000555	0.483549501	down
ASMM9PARTA046419	AK030763	0.000375	0.488566756	down
ASMM9PARTA006336		0.009015284	0.342247395	down
ASMM9PARTA014441	Gm15619	0.01389206	0.480857496	down
ASMM9PARTA009012		0.0000313	0.37537708	down
ASMM9PARTA009972	Gm807	0.01793847	0.381970576	down
ASMM9PARTA000027		0.001488198	0.153417263	down
ASMM9PARTA046533	Spatial	0.000803	0.274039378	down
ASMM9PARTA046533	Spatial	0.000803	0.274039378	down
ASMM9PARTA046533	Spatial	0.000803	0.274039378	down
ASMM9PARTA046533	Spatial	0.000803	0.274039378	down
ASMM9PARTA046533	Spatial	0.000803	0.274039378	down
ASMM9PARTA046533	Spatial	0.000803	0.274039378	down
mouselincRNA1284-_P1	mouselincRNA1284	0.023888398	0.43662063	down

ASMM9PARTA002943		0.000181	0.45819313	down
ASMM9PARTA002729		0.004080148	0.438781549	down
mouselincRNA0362+_P1	mouselincRNA0362	0.001024091	0.4650308	down
ASMM9PARTA018204	AU015336	0.003257209	0.396053282	down
ASMM9PARTA003994		0.004302118	0.250503763	down
ASMM9PARTA046088	AK038933	0.014618578	0.489841544	down
ASMM9PARTA006885		0.0000131	0.474909036	down
ASMM9PARTA045510	AA465934	8.71E-08	0.032668283	down
ASMM9PARTA015947	Gm12766	0.01046971	0.457966674	down
humanlincRNA0288-_P1	humanlincRNA0288	0.041815788	0.499016937	down
ASMM9PARTA016697	4930412C18Rik	0.000109	0.361271441	down
MM9LINCRNAEXON10592-_P1	mouselincRNA1215	0.003916962	0.473027588	down
ASMM9PARTA010513	Gm14756	0.001945664	0.481852937	down
humanlincRNA2300+_P1	humanlincRNA2300	0.004077318	0.374071461	down
ASMM9PARTA015118	E130218I03Rik	0.0000955	0.471291935	down
ASMM9PARTA008653		0.02055635	0.42165687	down
AA762819_P1	mouselincRNA0620	0.0000452	0.495304391	down
ASMM9PARTA019857		0.0000024	0.052299948	down
ASMM9PARTA000659		0.00000142	0.127091933	down
ASMM9PARTA016129	Gm12052	0.000946	0.496626121	down
ASMM9PARTA008115		0.000066	0.4596002	down
ASMM9PARTA016334	4930426D05Rik	0.000442	0.417698731	down
ASMM9PARTA011020	Olfr587-ps1	0.0000173	0.452106443	down
ASMM9PARTA048519	AK020087	0.000022	0.206085359	down
ASMM9PARTA019605	BC006965	0.0000749	0.085877431	down
ASMM9PARTA049719	Ubxd3	0.001560129	0.455276546	down
ASMM9PARTA049719	Ubxd3	0.001560129	0.455276546	down
ASMM9PARTA049957	AK037159	0.007303125	0.468762334	down
ASMM9PARTA049957	AK037159	0.007303125	0.468762334	down
ASMM9PARTA045865	Rmi1	0.000027	0.46352821	down
ASMM9PARTA045865	Rmi1	0.000027	0.46352821	down
ASMM9PARTA045865	Rmi1	0.000027	0.46352821	down
ASMM9PARTA015580	Gm16194	0.001408998	0.490612618	down
ASMM9PARTA019172	RP24-486L9.1	0.0000199	0.125848455	down
ASMM9PARTA019172	RP24-486L9.1	0.0000199	0.125848455	down
humanlincRNA1992+_P1	humanlincRNA1992	0.01292699	0.485081418	down
ASMM9PARTA007772		0.01841998	0.393336378	down
ASMM9PARTA050826	Flt3l	0.0000534	0.499682452	down
ASMM9PARTA000568		0.000054	0.389428663	down
MM9LINCRNAEXON10678-_P1	mouselincRNA1108	0.000864	0.383320469	down
ASMM9PARTA049468	BC055065	0.003232447	0.498902589	down
ASMM9PARTA015743	Gm16885	0.013186666	0.315818592	down
ASMM9PARTA015743	Gm16885	0.013186666	0.315818592	down
ASMM9PARTA002184		0.00000148	0.189728634	down
ASMM9PARTA012007	Gm13105	0.0000663	0.389486346	down
ASMM9PARTA046745	AK043075	0.0000294	0.418773797	down
ASMM9PARTA009360	Gm8935	0.00000596	0.167253224	down
ASMM9PARTA005358		0.000017	0.497060459	down
ASMM9PARTA008328		0.000827	0.408987484	down

ASMM9PARTA049425	AK047534	0.000586	0.180410368	down
ASMM9PARTA017505	Gprc2a-rs5	0.000132	0.496382735	down
ASMM9PARTA017371	Gm13236	0.0000567	0.43896422	down
ASMM9PARTA011045	Gm13606	0.000329	0.447446312	down
ASMM9PARTA047769	AK015236	0.007197172	0.350960377	down
ASMM9PARTA047769	AK015236	0.007197172	0.350960377	down
ASMM9PARTA018492	Gm16311	0.010534297	0.492585116	down
ASMM9PARTA046151	AY512931	0.000382	0.476806237	down
ASMM9PARTA002462		0.004421781	0.349586862	down
ASMM9PARTA000029		0.001741061	0.125177591	down
MM9LINCRNAEXON11761-_P1	mouselincRNA0237	0.0000212	0.302590035	down
ASMM9PARTA047918	AK014089	0.000016	0.299162316	down
ASMM9PARTA007575		0.007384884	0.486187179	down
ASMM9PARTA007575		0.007384884	0.486187179	down
ASMM9PARTA044915	4930471G03Rik	0.00022	0.433259701	down
ASMM9PARTA050475	Atg16l2	0.003020605	0.283329277	down
ASMM9PARTA006522		0.003308344	0.476319082	down
ASMM9PARTA045413	9530009M10Rik	0.004130147	0.347505318	down
ASMM9PARTA047065	AK006051	0.000000196	0.241939091	down
CUST_301_PI426073487	uc.303	0.001177246	0.419145669	down
CK382063_P1	humanlincRNA0287	0.003280742	0.44224689	down
ASMM9PARTA007214		0.00000192	0.372823772	down
MM9LINCRNAEXON10630+_P1	mouselincRNA1245	0.000102	0.300534916	down
ASMM9PARTA009780	Gm12569	0.001947491	0.392606836	down
CO044174_P1	mouselincRNA1066	0.000049	0.472173761	down
ASMM9PARTA000444		0.029128164	0.362629572	down
ASMM9PARTA017595	Gm11846	0.00000216	0.291409892	down
ASMM9PARTA006315		0.008131351	0.463956345	down
ASMM9PARTA001580		0.00519406	0.473822425	down
ASMM9PARTA016844	Gm15444	0.012283576	0.314743432	down
ASMM9PARTA016844	Gm15444	0.012283576	0.314743432	down
ASMM9PARTA048638	Zbtb9	0.019881556	0.427230511	down
ASMM9PARTA048638	Zbtb9	0.019881556	0.427230511	down
ASMM9PARTA048638	Zbtb9	0.019881556	0.427230511	down
ASMM9PARTA016224	4930412C18Rik	0.000000376	0.10551603	down
ASMM9PARTA003967		0.0000447	0.498906696	down
ASMM9PARTA011720	Gm13627	0.000157	0.428877194	down
ASMM9PARTA015647	1700125H03Rik	0.03708068	0.400345033	down
BE949468_P1	mouselincRNA1019	0.006572181	0.343576447	down
ASMM9PARTA009841	D130007C19Rik	0.0000867	0.435406869	down
ASMM9PARTA003111		0.000645	0.265245664	down
ASMM9PARTA000385		0.000192	0.357868589	down
ASMM9PARTA009352		0.001883952	0.135158404	down
ASMM9PARTA007116		0.00000436	0.387407595	down
ASMM9PARTA017353	Gm6117	0.007448608	0.461503245	down
ASMM9PARTA047039	AK166453	0.000000363	0.029903844	down
ASMM9PARTA007128		0.011875288	0.337378206	down
ASMM9PARTA014317	Gm14120	0.006682273	0.414347237	down
MM9LINCRNAEXON10341+_P1		0.000101	0.488959227	down

ASMM9PARTA014298	Gm14396	0.001661232	0.466454243	down
ASMM9PARTA007777		0.0000134	0.349362504	down
MM9LINCRNAEXON10138-_P1	mouselincRNA1567	0.00000805	0.286963067	down
ASMM9PARTA004565		0.0000104	0.031065693	down
ASMM9PARTA051808		0.000412	0.138451317	down
ASMM9PARTA006624		0.008812039	0.407491819	down
ASMM9PARTA047889	AK136126	0.00000144	0.071002299	down
ASMM9PARTA005094		0.001441168	0.492823747	down
MM9LINCRNAEXON11717+_P1	mouselincRNA0341	0.0000474	0.407132522	down
ASMM9PARTA019274	AC157896.1	0.0000219	0.441511591	down
CD563094_P1	mouselincRNA0148	0.001358144	0.172894398	down
MM9LINCRNAEXON10349-_P1		0.000332	0.420470859	down
ASMM9PARTA005587		0.015107151	0.432195262	down
ASMM9PARTA047548	AK016040	0.000246	0.421582494	down
AI558076_P1	mouselincRNA0528	0.017239751	0.425502926	down
ASMM9PARTA016836	Hoxb3os	0.001191853	0.440898776	down
ASMM9PARTA016836	Hoxb3os	0.001191853	0.440898776	down
ASMM9PARTA016836	Hoxb3os	0.001191853	0.440898776	down
ASMM9PARTA014069	A730017L22Rik	0.000129	0.159517135	down
ASMM9PARTA004045		0.000324	0.467248737	down
ASMM9PARTA017530	Gm16129	0.002298507	0.383433995	down
ASMM9PARTA017530	Gm16129	0.002298507	0.383433995	down
ASMM9PARTA017530	Gm16129	0.002298507	0.383433995	down
ASMM9PARTA048192	AK080258	0.008977083	0.465652104	down
ASMM9PARTA049060	mKIAA0137	0.017651971	0.42077832	down
ASMM9PARTA005909		0.016901856	0.469447401	down
ASMM9PARTA017182	Gm14820	0.019883476	0.279931869	down
