# Supplementary material for: Microarray Profile of Long Noncoding RNA and Messenger RNA Expression in a Model of Alzheimer’s Disease
Source: Life (Basel). 2020 May 14;10(5):64. doi: 10.3390/life10050064 (PMC7281340; doi:10.3390/life10050064)
Supplement: Supplementary file 1 [file life-10-00064-s001.zip › life-787240-supplemenatry-to be published - PDF/life-787240-supplementary/Table S5.pdf]

life

MDPI

Supplementary

Microarray Profile of Long Noncoding RNA and Messenger RNA Expression in a Model of Alzheimer's Disease

Linlin Wang †, Li Zeng †, Hailun Jiang, Zhuorong Li * and Rui Liu *

Institute of Medicinal Biotechnology, Chinese Academy of Medical Sciences and Peking Union Medical College, Beijing 100050, China; wanglinlin@wfmc.edu.cn (L.W.); zengsheng@imb.pumc.edu.cn (L.Z.); jianghailun@imb.pumc.edu.cn (H.J.)

* Correspondence: lizhourong@imb.pumc.edu.cn (Z.L.); +86-10-8352017; .liurui@imb.pumc.edu.cn (R.L.); Tel.: +86-10-67087731

Table S5. Differently expressed mRNAs in the brain of 1-month-old APP/PS1 mice compared with age-matched WT mice.

Probe Name	Symbol	*p*-Value	Fold Change	Regulation
ASMM9PARTA045052	Olfr220	0.003751036	2.4421592	up
ASMM9PARTA026649	Gm4858	0.041884437	2.1697762	up
ASMM9PARTA025747	Tshb	0.00000521	7.7614822	up
ASMM9PARTA036436	2210009G21Rik	0.013442672	2.3971777	up
ASMM9PARTA037561	Dbr1	0.000113	2.0642335	up
ASMM9PARTA044292	Zhx2	0.002187236	2.854121	up
ASMM9PARTA025730	Ephb1	0.000717	2.0853477	up
ASMM9PARTA024895	4930579F01Rik	0.03686085	2.7898862	up
ASMM9PARTA026372	Rbm20	0.002392128	2.387044	up
ASMM9PARTA030304	Mycs	0.003797783	3.1414168	up
ASMM9PARTA028893	Hnf1b	0.035493784	3.080625	up
ASMM9PARTA036289	Ceacam13	0.011300727	2.136738	up
ASMM9PARTA028604	Arhgef2	0.02494856	2.0964904	up
ASMM9PARTA039428	Gucy2c	0.018975068	2.5197103	up
ASMM9PARTA033070	Mknk2	0.010417876	2.7635362	up
ASMM9PARTA037710	Fam81a	0.0000496	2.2953494	up
ASMM9PARTA026869	Dnmt1	0.014693277	8.319181	up
ASMM9PARTA037963	Prss29	0.000323	4.2877235	up
ASMM9PARTA035878	Mapk6	0.000305	2.8842177	up
ASMM9PARTA028991	Slc4a3	0.000805	3.1703966	up
ASMM9PARTA022668	Gm5634	0.0000154	2.6224368	up
ASMM9PARTA021305	G630016D24Rik	0.032856867	4.5807657	up
ASMM9PARTA024014	Cdv3	0.000221	2.0749805	up
ASMM9PARTA034560	Kctd20	0.04966612	2.500376	up
ASMM9PARTA044936	Olfr774	0.041685455	2.1023014	up
ASMM9PARTA025718	Cdr1	0.000874	2.3227308	up
ASMM9PARTA036648	Pear1	0.010539373	4.361214	up
ASMM9PARTA028213	Klkb1	0.015042135	4.822848	up
ASMM9PARTA027615	Cdh15	0.019219782	2.8894737	up
ASMM9PARTA024198	Lpp	0.000184	3.1280704	up
ASMM9PARTA034297	Baiap2l1	0.000608	9.315259	up

ASMM9PARTA043897	Vps39	0.004893989	2.3448107	up
ASMM9PARTA036860	4930432K09Rik	0.046586692	2.4758584	up
ASMM9PARTA033615	Bhmt2	0.012276932	6.0526295	up
ASMM9PARTA030930	Aplnr	0.000451	2.1038837	up
ASMM9PARTA042888	Vsig8	0.00026	2.3886468	up
ASMM9PARTA022787	Gm5640	0.021163333	2.170801	up
ASMM9PARTA019730	Penk	0.0000101	2.0385914	up
ASMM9PARTA021143	Defb23	0.019433258	3.2346437	up
ASMM9PARTA029828	Cdh5	0.001553116	2.7611332	up
ASMM9PARTA027701	Gja8	0.047042444	5.3950458	up
ASMM9PARTA044055	Abhd16b	0.012498511	2.5325136	up
ASMM9PARTA027992	Gif	0.020529864	2.3620298	up
ASMM9PARTA024307	Hsd3b3	0.010374174	2.7383063	up
ASMM9PARTA041340	Phc3	0.042536356	2.196776	up
ASMM9PARTA023813	Tmem150b	0.006467491	3.4289734	up
ASMM9PARTA027844	Gria1	0.00000206	2.023898	up
ASMM9PARTA032924	Apbh	0.046796106	2.0778801	up
ASMM9PARTA034865	Ssr1	0.000238	2.055347	up
ASMM9PARTA026193	Tmem11	0.011518378	2.1342025	up
ASMM9PARTA021564	Shroom4	0.001165245	2.3926134	up
ASMM9PARTA022070	Olfr455	0.00052	3.1390016	up
ASMM9PARTA021097	Trpm3	0.001609749	3.9606633	up
ASMM9PARTA029691	Cd44	0.04855457	2.633932	up
ASMM9PARTA035866	1700030J22Rik	0.026191039	2.0290802	up
ASMM9PARTA042588	Fbxw16	0.000124	2.104547	up
ASMM9PARTA025516	Slc17a3	0.005393438	3.531697	up
ASMM9PARTA023315	Gatad2a	0.01264692	2.7757745	up
ASMM9PARTA040420	Olfr361	0.0351323	3.0229704	up
ASMM9PARTA030912	Slc22a3	0.009055254	2.4426003	up
ASMM9PARTA031792	Ar	0.0457471	2.6268723	up
ASMM9PARTA042087	Fry	0.00804436	2.0477142	up
ASMM9PARTA043179	Fam163a	0.006152224	2.2731571	up
ASMM9PARTA019635	Whamm	0.00001	2.3447661	up
ASMM9PARTA035507	Otop3	0.019065587	2.7423575	up
ASMM9PARTA041596	Pskh1	0.031563457	2.012173	up
ASMM9PARTA031008	Procr	0.005771449	2.3010159	up
ASMM9PARTA034391	1700011A15Rik	0.014602835	2.8966568	up
ASMM9PARTA042058	Mon1b	0.015270972	2.3139317	up
ASMM9PARTA043018	Ust	0.002058796	2.2558486	up
ASMM9PARTA036930	Fam65b	0.010708857	2.933867	up
ASMM9PARTA031518	Spnb1	0.000164	2.6206775	up
ASMM9PARTA020586	Defb46	0.007054768	2.1755826	up
ASMM9PARTA042206	Stk36	0.000698	3.3935292	up
ASMM9PARTA040424	Olfr197	0.015071325	2.1973288	up
ASMM9PARTA022169	Mki67	0.003529784	3.030028	up
ASMM9PARTA044228	Ece1	0.001867219	2.488216	up
ASMM9PARTA023951	Grm5	0.00349157	2.1228971	up
ASMM9PARTA029461	Vmn2r42	0.009567143	3.1388469	up
ASMM9PARTA037234	Pcdh1	0.000857	3.1323411	up
ASMM9PARTA035876	Pmvk	0.010726194	2.721194	up
ASMM9PARTA038992	Vmn1r16	0.000164	2.4647965	up
ASMM9PARTA043919	A430033K04Rik	0.0000361	2.0145612	up
ASMM9PARTA043036	Klhl18	0.011628633	3.9553802	up
ASMM9PARTA024704	Islr2	0.00321518	2.120134	up
ASMM9PARTA020539	Ffar3	0.006652573	2.678523	up
ASMM9PARTA037652	Nfkbiz	0.03210981	2.0775528	up
ASMM9PARTA038866	1110008J03Rik	0.001094808	2.7508729	up

ASMM9PARTA036549	Mbd3l1	0.028641967	3.1825857	up
ASMM9PARTA041772	Galnt5	0.001775296	2.5194244	up
ASMM9PARTA019955	Lrrc52	0.002518788	3.0269392	up
ASMM9PARTA020417	Gm5591	0.007129067	4.217878	up
ASMM9PARTA027610	Capn5	0.010467581	2.154866	up
ASMM9PARTA021767	Rorb	0.010541505	2.6885617	up
ASMM9PARTA030638	Apcs	0.004201958	3.175886	up
ASMM9PARTA023026	Kng2	0.003901125	2.04617	up
ASMM9PARTA028084	Gabrr2	0.000542	3.4749467	up
ASMM9PARTA037250	1600029D21Rik	0.041963648	2.3025756	up
ASMM9PARTA037304	Ep400	0.000563	2.3140547	up
ASMM9PARTA030219	Hoxc13	0.004009119	2.9702234	up
ASMM9PARTA027534	Slc7a2	0.000575	2.1334062	up
ASMM9PARTA033439	Tsga8	0.000414	2.9582245	up
ASMM9PARTA019763	Nlrp14	0.045316145	2.1160905	up
ASMM9PARTA030257	Klra8	0.023608824	2.1983066	up
ASMM9PARTA031864	Ltbp2	0.003326668	2.25256	up
ASMM9PARTA025754	Hnrnpf	0.034013495	2.1414032	up
ASMM9PARTA044202	Tnfsf18	0.006154811	2.7685206	up
ASMM9PARTA027062	Dcun1d4	0.001249509	2.9976602	up
ASMM9PARTA039606	Fcrla	0.014220423	2.220666	up
ASMM9PARTA033437	Il21	0.02809222	2.7552145	up
ASMM9PARTA044252	BC049730	0.011351231	2.406666	up
ASMM9PARTA033390	Rqcd1	0.022882305	2.7984204	up
ASMM9PARTA033704	Sync	0.004800494	3.3473167	up
ASMM9PARTA040166	Olfr122	0.003276278	3.2400842	up
ASMM9PARTA035380	Ccdc80	0.003335427	2.1923726	up
ASMM9PARTA029737	Dlx5	0.000591	2.1020262	up
ASMM9PARTA040757	Olfr1214	0.018534712	2.7296355	up
ASMM9PARTA044593	Pglyrp3	0.0314343	2.3026562	up
ASMM9PARTA044605	Ippk	0.000586	2.0034702	up
ASMM9PARTA022495	Ptprq	0.034451496	2.2360857	up
ASMM9PARTA024087	4930451C15Rik	0.00000216	2.8518217	up
ASMM9PARTA032256	Chst2	0.00000208	3.0054355	up
ASMM9PARTA031482	Slco1a1	0.000185	2.5802124	up
ASMM9PARTA043529	Agap1	0.006012578	2.037107	up
ASMM9PARTA024965	Ush1c	0.006916838	2.1530454	up
ASMM9PARTA041952	Tstd2	0.02403953	2.0026326	up
ASMM9PARTA035193	Rnf151	0.001736181	3.0927174	up
ASMM9PARTA033142	G6pc2	0.039489966	2.233592	up
ASMM9PARTA024887	Ccdc90b	0.004433446	3.0351093	up
ASMM9PARTA021918	Gm5347	0.013306319	2.410623	up
ASMM9PARTA033834	2010106G01Rik	0.000989	3.5657985	up
ASMM9PARTA040339	Olfr196	0.008369318	2.762678	up
ASMM9PARTA035587	Lce1d	0.047274437	2.7813826	up
ASMM9PARTA024383	Fam5c	0.031252425	3.781462	up
ASMM9PARTA025288	Rtdr1	0.002890317	2.5196795	up
ASMM9PARTA039817	Ankrd40	0.00000341	2.186199	up
ASMM9PARTA023477	Slc14a2	0.003879792	2.6556864	up
ASMM9PARTA037995	Clec2h	0.002186674	2.524383	up
ASMM9PARTA044601	Abr	0.029681418	2.072508	up
ASMM9PARTA044144	Pou2f1	0.000792	2.0017056	up
ASMM9PARTA040966	Cxcl17	0.009809971	3.2530086	up
ASMM9PARTA043668	Edc4	0.033970255	3.3108282	up
ASMM9PARTA030102	Heph	0.003666807	2.6783116	up
ASMM9PARTA021695	Glyctk	0.007635023	2.0406108	up
ASMM9PARTA037407	2310079F23Rik	0.000254	3.4571393	up

ASMM9PARTA042579	Tmem169	0.001039684	2.6135087	up
ASMM9PARTA043176	Klhl23	0.001150392	2.3827937	up
ASMM9PARTA024222	Cntfr	0.0000176	6.269727	up
ASMM9PARTA035572	Klk5	0.021718172	2.1237462	up
ASMM9PARTA021180	Ceacam1	0.005726648	3.3772664	up
ASMM9PARTA022678	Prrt4	0.013258315	2.336178	up
ASMM9PARTA030244	Gsr	0.0000254	7.3731484	up
ASMM9PARTA030760	Prl	9.22E-08	3.846125	up
ASMM9PARTA043610	Nlrx1	0.04522601	2.5623264	up
ASMM9PARTA036875	Abcb8	0.020251606	2.6136885	up
ASMM9PARTA030387	Igf2r	0.038781494	2.597996	up
ASMM9PARTA020624	Gal3st3	0.004824128	2.512867	up
ASMM9PARTA034491	D14Ertd449e	0.000000137	3.4564073	up
ASMM9PARTA019864	Olfr279	0.030927038	2.2184315	up
ASMM9PARTA020982	Gm136	0.000586	2.9435625	up
ASMM9PARTA041163	Ccdc137	0.036690895	2.0706782	up
ASMM9PARTA029660	Colq	0.018523209	2.5883741	up
ASMM9PARTA033694	Nup98	0.019515885	2.3826878	up
ASMM9PARTA034058	Osbpl5	0.036261357	2.0437946	up
ASMM9PARTA028599	Raet1c	0.04866519	2.7203033	up
ASMM9PARTA033905	Ndc80	0.015883194	2.1719277	up
ASMM9PARTA030586	Per2	0.000529	2.267821	up
ASMM9PARTA024773	Myef2	0.000000148	3.5772727	up
ASMM9PARTA037145	Gsc2	0.004948864	3.1132085	up
ASMM9PARTA026158	Gtf2e2	0.01296082	2.0679715	up
ASMM9PARTA040825	Olfr109	0.048210464	3.3950684	up
ASMM9PARTA020210	2700007P21Rik	0.000301	7.393998	up
ASMM9PARTA027898	Klra5	0.006089612	2.4630258	up
ASMM9PARTA043849	Duxbl	0.000972	3.9733903	up
ASMM9PARTA036484	Pi4k2b	0.032137237	3.231536	up
ASMM9PARTA025140	Rrp1b	0.005512097	2.736371	up
ASMM9PARTA037729	Apobec1	0.005486235	3.2105725	up
ASMM9PARTA031745	Fbxo6	0.005948691	3.0164595	up
ASMM9PARTA041228	9930023K05Rik	0.0292919	2.9537826	up
ASMM9PARTA037840	Akap12	0.00016	2.0194292	up
ASMM9PARTA038935	Ehd4	0.020563837	2.1692934	up
ASMM9PARTA037836	F13b	0.024273245	2.8877404	up
ASMM9PARTA029972	Lhx8	0.0000145	2.2731035	up
ASMM9PARTA027224	Taok3	0.000000362	2.1145806	up
ASMM9PARTA031132	Cd70	0.00155445	2.1202483	up
ASMM9PARTA020973	Skil	0.005644537	2.1603162	up
ASMM9PARTA042613	Pcnxl2	0.007640276	2.8097208	up
ASMM9PARTA024808	Ttc15	0.000000162	46.72725	up
ASMM9PARTA025859	Frem3	0.002473361	3.018657	up
ASMM9PARTA021040	Trpm3	0.002530899	11.01106	up
ASMM9PARTA042786	Mrgprb2	0.001131448	3.4861302	up
ASMM9PARTA044237	Tspan15	0.000944	2.3455203	up
ASMM9PARTA020181	Phactr1	0.000028	2.0741315	up
ASMM9PARTA037352	Bicd2	0.010708182	2.6992705	up
ASMM9PARTA022560	Ncam1	0.000199	2.0635138	up
ASMM9PARTA032065	Dlc1	0.00000839	4.717971	up
ASMM9PARTA020201	Thoc7	0.0000549	4.0574574	up
ASMM9PARTA034441	4933411K16Rik	0.020354683	2.902926	up
ASMM9PARTA035504	Spaca4	0.02384921	2.2065644	up
ASMM9PARTA027033	Kcnmb3	0.01989948	2.4002414	up
ASMM9PARTA037326	2610028H24Rik	0.00244168	3.2170994	up
ASMM9PARTA037842	Bglap-rs1	0.0000481	2.9825518	up

ASMM9PARTA039075	Cyyr1	0.005379924	2.5244765	up
ASMM9PARTA031408	Cdca3	0.0000225	2.082289	up
ASMM9PARTA021003	Trpm3	0.0000857	7.781857	up
ASMM9PARTA030509	Myl4	0.0000416	2.0461586	up
ASMM9PARTA020138	Adcy5	0.000197	2.2494032	up
ASMM9PARTA044588	Tas2r117	0.043061458	2.1725197	up
ASMM9PARTA042118	Vwa2	0.020250231	4.088876	up
ASMM9PARTA026891	Gm5741	0.000000244	4.2060094	up
ASMM9PARTA043087	Igsf1	0.0485156	2.5066066	up
ASMM9PARTA022556	Pcdh9	0.000000213	4.2718415	up
ASMM9PARTA026365	Arpc4	0.042895436	2.9742742	up
ASMM9PARTA035697	Prss32	0.001030298	2.2232764	up
ASMM9PARTA023985	Ddx4	0.009475864	2.7534766	up
ASMM9PARTA029020	Reg2	0.028020782	2.1968572	up
ASMM9PARTA044758	Gm12597	0.048003875	2.2052891	up
ASMM9PARTA041379	Cpne2	0.002771823	2.0274973	up
ASMM9PARTA041162	Asb16	0.02493451	2.3192282	up
ASMM9PARTA036401	Ptar1	0.008298238	2.2376778	up
ASMM9PARTA021422	Sel1l	0.0000113	2.8668406	up
ASMM9PARTA041081	Olfr711	0.021222772	2.5598984	up
ASMM9PARTA029782	Dffa	0.000119	2.0975533	up
ASMM9PARTA029581	Cga	0.000258	2.755183	up
ASMM9PARTA025221	Mapk8ip3	0.000253	2.0993261	up
ASMM9PARTA038444	Cysltr2	0.028974479	2.8222558	up
ASMM9PARTA024458	Casz1	0.000271	3.9513557	up
ASMM9PARTA027171	App	0.0000505	2.1252465	up
ASMM9PARTA041048	Olfr1390	0.04732841	2.7029982	up
ASMM9PARTA019935	Txlna	0.0000531	3.7452648	up
ASMM9PARTA043988	Actl9	0.0391684	2.271989	up
ASMM9PARTA030282	Hp1bp3	0.003339073	2.1426358	up
ASMM9PARTA033144	Accn5	0.004842273	2.3114023	up
ASMM9PARTA027749	Gh	0.00000055	13.226465	up
ASMM9PARTA041214	Adam1b	0.026432866	2.1164045	up
ASMM9PARTA022335	Cep152	0.0222133	2.6376128	up
ASMM9PARTA021478	Kcnh1	0.004350807	2.7828128	up
ASMM9PARTA020625	Ttbk2	0.04167986	2.1556003	up
ASMM9PARTA041481	Zswim4	0.0000592	2.4755757	up
ASMM9PARTA044533	Zfp933	0.000000125	8.12096	up
ASMM9PARTA029751	Alox5	0.00419579	2.0628865	up
ASMM9PARTA031603	Pde10a	0.0000936	2.3514514	up
ASMM9PARTA022770	4933407P14Rik	0.003295882	2.4269924	up
ASMM9PARTA023801	8430427H17Rik	0.018969456	2.158623	up
ASMM9PARTA027306	App	0.00001	2.2057827	up
ASMM9PARTA035416	Galntl5	0.010521207	2.3042307	up
ASMM9PARTA026471	Lrtm2	0.031467587	2.209503	up
ASMM9PARTA022180	Dscaml1	0.02670769	2.0475645	up
ASMM9PARTA038806	Vmn1r24	0.021826038	2.3893595	up
ASMM9PARTA044769	Tigd4	0.001872062	5.801745	up
ASMM9PARTA022310	Mfhas1	0.00000897	2.2740405	up
ASMM9PARTA023780	Bcl2l15	0.000124	2.7243848	up
ASMM9PARTA035366	1700029I01Rik	0.000387	3.0644279	up
ASMM9PARTA030019	Gast	0.001739646	2.949806	up
ASMM9PARTA042803	Auts2	0.006495534	2.8973548	up
ASMM9PARTA023424	Fcrl5	0.000142	4.1270227	up
ASMM9PARTA028168	Inhba	0.03436925	2.6375701	up
ASMM9PARTA029724	Cacna2d3	0.0000631	2.134954	up
ASMM9PARTA027494	Bglap	0.00000986	2.8016675	up

ASMM9PARTA021768	Slc7a2	0.0004	2.269368	up
ASMM9PARTA026984	App	0.00000158	2.0835586	up
ASMM9PARTA044577	Dand5	0.032071725	2.0551238	up
ASMM9PARTA025083	Macc1	0.000177	3.927157	up
ASMM9PARTA044835	Acsm3	0.001409163	6.756055	up
ASMM9PARTA035080	D14Ertd449e	0.00000825	3.3127344	up
CUST_262_PI426409190	Gm4133	0.02526818	2.0456967	up
ASMM9PARTA020878	Pak6	0.000299	2.579196	up
ASMM9PARTA041636	Nup214	0.019956734	2.0582464	up
ASMM9PARTA026835	Krtap22-2	0.011331555	3.007457	up
ASMM9PARTA027539	Cacng1	0.00000412	2.0118864	up
ASMM9PARTA031494	Scn11a	0.004698082	2.6589656	up
ASMM9PARTA035781	Prdm16	0.024131129	3.3408496	up
ASMM9PARTA025891	Lekr1	0.017828165	5.922202	up
ASMM9PARTA044591	Plac9	0.00000246	2.779668	up
ASMM9PARTA041881	Ttc34	0.001574245	2.8212528	up
ASMM9PARTA025131	Prl	0.000000173	3.448135	up
ASMM9PARTA030978	Rlim	0.0000422	2.0607562	up
ASMM9PARTA032643	Pdyn	0.000359	2.0603468	up
ASMM9PARTA020773	Bglap2	0.000224	2.5209253	up
ASMM9PARTA032408	Cyp11a1	0.000219	2.8131297	up
ASMM9PARTA028968	Tnnc1	0.0000042	3.4523382	up
ASMM9PARTA021777	A4gnt	0.0227427	2.0165484	up
ASMM9PARTA035066	Ooep	0.00222082	2.3876872	up
ASMM9PARTA025377	Erlin1	0.02012964	2.2184336	up
ASMM9PARTA022516	Neto2	0.0000164	2.3385117	up
ASMM9PARTA027293	Spp1	0.000000766	0.270835365	down
ASMM9PARTA023986	Zfat	0.010961509	0.468865788	down
ASMM9PARTA033120	Cbln3	0.00000213	0.13374038	down
ASMM9PARTA039095	Dbh	0.001267741	0.487637987	down
ASMM9PARTA031729	Ccl5	0.00069	0.283192657	down
ASMM9PARTA027822	Gch1	0.0000267	0.427868825	down
ASMM9PARTA035334	Cthrc1	0.000206	0.386814544	down
ASMM9PARTA021588	Gm13152	0.0000397	0.428907931	down
ASMM9PARTA024173	Tph1	0.04144048	0.375018502	down
ASMM9PARTA021442	Gm13051	0.0000463	0.340581405	down
ASMM9PARTA030360	Masp2	0.000532	0.413983176	down
ASMM9PARTA027888	Hbb-b1	0.002115642	0.438872673	down
ASMM9PARTA028818	Th	0.000000812	0.179842992	down
ASMM9PARTA029194	Alox12b	0.00000407	0.493526414	down
ASMM9PARTA029086	Ret	0.007033648	0.37521681	down
ASMM9PARTA035300	Hddc3	0.00000245	0.471653484	down
ASMM9PARTA024752	Gm3604	0.000567	0.453091381	down
ASMM9PARTA025656	Dcaf17	0.0000438	0.464496813	down
ASMM9PARTA037112	Rpl18a	3.13E-09	0.064899366	down
ASMM9PARTA027419	Agt	0.00000641	0.273542519	down
ASMM9PARTA040089	Tspan8	0.000252	0.497196977	down
ASMM9PARTA029936	Cyp2d9	0.0000257	0.314010883	down
ASMM9PARTA027054	Spp1	0.00000724	0.202091158	down
ASMM9PARTA044859	H2-Ab1	0.0000141	0.421966211	down
ASMM9PARTA029513	Cd3g	0.000299	0.208552809	down
ASMM9PARTA029838	Cyp2d10	0.002076154	0.376422307	down
ASMM9PARTA020724	Lce3f	0.004041684	0.300932856	down
ASMM9PARTA023568	Mup10	0.000000858	0.40693747	down
ASMM9PARTA024716	Nek3	0.007895443	0.400336507	down
ASMM9PARTA028744	Serpina1c	0.0000854	0.414697201	down
ASMM9PARTA029778	Slc6a3	0.0000363	0.319221478	down

ASMM9PARTA038671	D17H6S56E-3	0.007374279	0.449391082	down
ASMM9PARTA030588	Pitx1	0.001171521	0.28862846	down
ASMM9PARTA035824	Cd209b	0.000308	0.390840634	down
ASMM9PARTA036237	Ubiad1	0.002128646	0.461540776	down
ASMM9PARTA033715	Foxb1	0.00000363	0.154075625	down
ASMM9PARTA027255	Ambp	0.0000222	0.472511814	down
ASMM9PARTA032497	Fabp1	0.000212	0.360369723	down
ASMM9PARTA032626	Cyp3a25	0.0191466	0.436343561	down
ASMM9PARTA029223	Apoa1	0.0000916	0.331900343	down
ASMM9PARTA023701	Pcp2	0.000104	0.197793753	down
ASMM9PARTA039382	Klhdc8a	0.000243	0.493643233	down
ASMM9PARTA032773	Scand1	0.044511314	0.444243488	down
ASMM9PARTA029910	Epm2a	0.00000628	0.043398766	down
ASMM9PARTA030400	Mab21l1	0.0000593	0.279704648	down
ASMM9PARTA043205	Mslnl	0.000119	0.393636876	down
ASMM9PARTA028385	Hsd3b5	0.0000164	0.256714015	down
ASMM9PARTA039277	Cabp7	0.019661415	0.221170679	down
ASMM9PARTA026754	Fau	0.0000286	0.364176895	down
ASMM9PARTA032388	Hbb-b2	0.0000465	0.477889099	down
ASMM9PARTA042788	Eda2r	0.002271703	0.495996023	down
ASMM9PARTA029855	Gsta3	0.0294203	0.457525819	down
ASMM9PARTA028854	Serpina1b	0.000214	0.408362146	down
ASMM9PARTA024525	Rdh7	0.003904659	0.496537643	down
ASMM9PARTA026597	Kirrel3	0.001898905	0.486393909	down
ASMM9PARTA030315	Slc6a4	0.000006	0.328893815	down
ASMM9PARTA027693	F2rl1	0.049067512	0.473549154	down
ASMM9PARTA041954	Rln3	0.0000998	0.26294437	down
ASMM9PARTA040898	Olfr167	0.004060974	0.360103214	down
ASMM9PARTA034098	Emid2	0.0000673	0.433712847	down
ASMM9PARTA044396	Cd300lh	0.000000384	0.22127017	down
CUST_305_PI426409190	Ccl28	0.000896	0.364664825	down
ASMM9PARTA021492	Gbp11	0.000191	0.485064077	down
ASMM9PARTA042165	Slc24a5	0.000026	0.289132192	down
ASMM9PARTA044541	Xkr8	0.00000112	0.476201361	down
ASMM9PARTA022794	Rbm8a	0.00000015	0.138931236	down
ASMM9PARTA029884	Gata6	0.000221	0.346125713	down
ASMM9PARTA024749	Myef2	0.0000299	0.077665451	down
ASMM9PARTA027936	Inadl	0.040855188	0.486943914	down
ASMM9PARTA036550	2410076I21Rik	0.00018	0.285626574	down
ASMM9PARTA040145	Olfr360	0.012411629	0.342445277	down
ASMM9PARTA027393	Alx4	0.003925964	0.357112605	down
ASMM9PARTA042802	C730048C13Rik	0.0000349	0.197717588	down
ASMM9PARTA036982	Trim14	0.000158	0.202457818	down
ASMM9PARTA021026	Mef2a	0.01771908	0.499932884	down
ASMM9PARTA029776	Dao	0.0000532	0.092268934	down
ASMM9PARTA029472	Alb	0.000132	0.291191922	down
ASMM9PARTA038655	Ugt2b38	0.000787	0.349204503	down
ASMM9PARTA023296	Gm3448	0.0000146	0.310497803	down
ASMM9PARTA031869	Ccl7	0.001778032	0.337732639	down
ASMM9PARTA031126	Sphk1	0.000013	0.45064175	down
ASMM9PARTA020376	Gm5622	0.000775	0.294413013	down
ASMM9PARTA042036	Agtr1b	0.015565972	0.252611166	down
ASMM9PARTA042858	Gpr81	0.0000645	0.364822501	down
ASMM9PARTA035197	Fibin	0.000000527	0.461992885	down
ASMM9PARTA028012	Mpzl2	0.000413	0.435237223	down
ASMM9PARTA032788	Ear5	0.006115099	0.362974193	down
ASMM9PARTA033515	H2-Q8	0.000101	0.248923871	down

ASMM9PARTA036465	Cda	0.00000179	0.376086905	down
ASMM9PARTA033683	Ms4a4b	0.006376007	0.369090379	down
ASMM9PARTA037619	Tlr1	0.0000658	0.483324811	down
ASMM9PARTA027086	Spp1	0.0000364	0.302828051	down
ASMM9PARTA020037	Arhgef11	0.011509744	0.191757371	down
ASMM9PARTA027011	Mup15	0.000000154	0.407793754	down
ASMM9PARTA026146	Slc10a1	0.001751838	0.240444148	down
ASMM9PARTA036813	1700011I03Rik	0.046159588	0.389570997	down
ASMM9PARTA020699	Tmem171	0.023241505	0.445388272	down
ASMM9PARTA033750	Scel	0.01893564	0.439891934	down
ASMM9PARTA038738	Pou4f2	0.000000782	0.021485966	down
ASMM9PARTA037570	Abcb5	0.011824286	0.481956559	down
ASMM9PARTA029488	Cyp8b1	0.000753	0.434492972	down
ASMM9PARTA039381	Ankrd33	0.006048001	0.481616227	down
ASMM9PARTA030083	Neurog1	0.005889766	0.458822073	down
ASMM9PARTA028004	Hpd	0.001849783	0.402435783	down
ASMM9PARTA044070	Exoc8	0.0000118	0.44342506	down
ASMM9PARTA031095	Sox14	0.0000014	0.120169364	down
ASMM9PARTA035055	Slc47a1	0.0000425	0.394506808	down
ASMM9PARTA043102	Vsig4	0.018527258	0.490885339	down
ASMM9PARTA024271	Pax3	0.012761468	0.404511272	down
ASMM9PARTA042808	Gchfr	0.00000138	0.153785482	down
ASMM9PARTA036957	Manf	0.000811	0.399164692	down
ASMM9PARTA020936	Calca	0.0000178	0.411528917	down
ASMM9PARTA027947	Epyc	0.001802313	0.329099934	down
ASMM9PARTA020324	Skap1	0.00133955	0.243848436	down
ASMM9PARTA030022	H2-Q10	0.0000253	0.365611704	down
ASMM9PARTA041084	Olfr555	0.017819004	0.457085186	down
ASMM9PARTA024098	Emp3	0.048557542	0.492098425	down
ASMM9PARTA044861	Olfr1349	0.009783274	0.472811115	down
ASMM9PARTA031618	Cd3d	0.003372742	0.277884285	down
ASMM9PARTA026103	Myt1	0.003210547	0.41371868	down
ASMM9PARTA025046	Gm8267	0.003713606	0.277760695	down
ASMM9PARTA032045	Uncx	0.000868	0.111744916	down
ASMM9PARTA020218	Epb4.1l1	0.016178584	0.378831172	down
ASMM9PARTA033840	Pla2g12b	0.00000344	0.255397507	down
ASMM9PARTA042468	Tmem132c	0.038441814	0.472217776	down
ASMM9PARTA031158	Saa3	0.000866	0.455177448	down
ASMM9PARTA034658	Med18	0.010337146	0.458610453	down
ASMM9PARTA037474	Tmem210	0.006449624	0.421148352	down
ASMM9PARTA038575	Lypd3	0.003519286	0.398857513	down
ASMM9PARTA040003	Olfr1325	0.000744	0.407930795	down
ASMM9PARTA038599	Cdhr1	0.000257	0.411554203	down
ASMM9PARTA044750	Serpinb3c	0.000307	0.377743875	down
ASMM9PARTA041741	Tph2	7.79E-08	0.074974417	down
ASMM9PARTA024986	Clec4d	0.011190993	0.417291561	down
ASMM9PARTA029274	Thrb	0.042430427	0.269124158	down
ASMM9PARTA035865	Krtap4-13	0.000036	0.24358465	down
ASMM9PARTA044580	Trim56	0.0000296	0.491315097	down
ASMM9PARTA023546	Serac1	0.002654136	0.458561558	down
ASMM9PARTA040990	Rgs13	0.01656337	0.436844133	down
ASMM9PARTA041433	Skor1	0.012819057	0.456959029	down
ASMM9PARTA038106	Pth2	0.00000367	0.131878955	down
ASMM9PARTA023902	Sema6b	0.000808	0.482857497	down
ASMM9PARTA040340	Olfr1238	0.017646741	0.35896544	down
ASMM9PARTA020537	Eif4e1b	0.001858372	0.357191958	down
ASMM9PARTA033706	Rsc1a1	0.008604575	0.478494564	down

ASMM9PARTA031047	Rhag	0.014100929	0.382919493	down
ASMM9PARTA039138	Il23r	0.000192	0.393423424	down
ASMM9PARTA025955	Vmn1r159	0.000553	0.448809868	down
ASMM9PARTA025833	Ankrd55	0.00000145	0.213764757	down
ASMM9PARTA037766	Cxcr6	0.01597911	0.33174342	down
ASMM9PARTA029743	Epha2	0.006307291	0.435350078	down
ASMM9PARTA033257	Doc2g	0.000000134	0.375884612	down
ASMM9PARTA031013	Reg3a	0.001495296	0.480919981	down
ASMM9PARTA031999	Bhmt	0.0000367	0.464683259	down
ASMM9PARTA021799	Pitx2	0.001764554	0.386329574	down
ASMM9PARTA027018	Bcl2a1c	0.001010992	0.463364288	down
ASMM9PARTA027835	Cyp2f2	0.0000198	0.429006466	down
ASMM9PARTA031281	Sgcg	0.01973625	0.424984445	down
ASMM9PARTA041416	BC030307	0.000474	0.47952353	down
ASMM9PARTA037532	Akr1c21	0.001751824	0.421506652	down
ASMM9PARTA028812	Serpina1d	0.000353	0.401028333	down
ASMM9PARTA030222	Ifna4	0.000351	0.41213665	down
ASMM9PARTA028978	Prox1	0.000362	0.460240788	down
ASMM9PARTA028931	Spp1	0.0000299	0.283136883	down
ASMM9PARTA039158	Vmn1r75	0.0000873	0.342131613	down
ASMM9PARTA027757	Chad	0.000333	0.42549563	down
ASMM9PARTA030085	Erf	0.018492376	0.499463826	down
ASMM9PARTA026853	9830107B12Rik	0.022867063	0.417142626	down
ASMM9PARTA030350	Foxa2	0.00000139	0.080439357	down
ASMM9PARTA023189	Zfp385b	0.047261067	0.392977492	down
ASMM9PARTA022734	Gm11428	0.0000156	0.447403592	down
ASMM9PARTA044230	BC048679	0.004698515	0.38887786	down
ASMM9PARTA019880	Olfr111	0.000821	0.456762413	down
ASMM9PARTA019871	Dnmt3b	0.000092	0.449614613	down
ASMM9PARTA027981	Evx1	0.0000138	0.052216071	down
ASMM9PARTA021692	Mup3	8.37E-08	0.308499475	down
ASMM9PARTA030391	Irx2	0.000000204	0.031454856	down
ASMM9PARTA044724	Olfr741	0.005040333	0.253448021	down
ASMM9PARTA024658	Birc7	0.00000366	0.340921333	down
ASMM9PARTA028475	Pah	0.000402	0.346199395	down
ASMM9PARTA022918	Dnahc9	0.008782837	0.32455683	down
ASMM9PARTA033277	Cxcl10	0.004446081	0.471386309	down
ASMM9PARTA038372	Cd209a	0.000857	0.439088714	down
ASMM9PARTA020925	Shisa3	0.0000191	0.323512727	down
ASMM9PARTA028663	Prg2	0.0000244	0.314351926	down
ASMM9PARTA023422	Arhgef16	0.0000113	0.322846181	down
ASMM9PARTA041247	Lrrc45	0.027927503	0.459389719	down
ASMM9PARTA028666	Prl7a1	0.00000581	0.298754118	down
ASMM9PARTA039640	Chi3l4	0.00031	0.476187982	down
ASMM9PARTA031052	Sncg	0.00000178	0.317175926	down
ASMM9PARTA030826	Otp	0.00000451	0.332344873	down
ASMM9PARTA023255	Col2a1	0.0000613	0.498609304	down
ASMM9PARTA030228	Kap	0.0000321	0.155361164	down
ASMM9PARTA037978	Msr1	0.000596	0.457828333	down
ASMM9PARTA044308	Mrvi1	0.000125	0.493701358	down
ASMM9PARTA023537	Catsper4	0.003237183	0.321849911	down
ASMM9PARTA027144	Mup17	0.00000132	0.498565279	down
ASMM9PARTA029207	Tlx2	0.008945637	0.321060826	down
ASMM9PARTA031089	Sema6c	0.016469039	0.461748522	down
ASMM9PARTA031582	Foxc2	0.000000216	0.301975995	down
ASMM9PARTA020509	Vwde	0.003051164	0.391422002	down
CUST_216_PI426409190	Tcstv1	0.000509	0.49688404	down

ASMM9PARTA040880	Hrh4	0.001494183	0.328859139	down
ASMM9PARTA032589	Tbx21	0.000366	0.223504727	down
ASMM9PARTA032874	Rbck1	0.001264961	0.392780537	down
ASMM9PARTA030041	Ldb1	0.03084223	0.425370745	down
ASMM9PARTA044389	Gse1	0.047766864	0.493533648	down
ASMM9PARTA025799	Tmem40	0.000768	0.387595472	down
ASMM9PARTA020142	Gm13154	0.00000258	0.45213455	down
ASMM9PARTA028465	Mgp	0.000959	0.470358568	down
ASMM9PARTA034414	Car13	0.000197	0.49643855	down
ASMM9PARTA025047	1700024P16Rik	0.0000271	0.434459409	down
ASMM9PARTA028583	Mup2	0.0000741	0.371776097	down
ASMM9PARTA043495	Hist1h2ac	0.000699	0.151374328	down
ASMM9PARTA022033	Arfip1	0.0000288	0.420924174	down
ASMM9PARTA031215	Zscan21	0.00057	0.460670551	down
ASMM9PARTA031086	Scnn1g	0.000116	0.463856101	down
ASMM9PARTA038143	Lmo1	0.001261006	0.491758762	down
ASMM9PARTA025291	Prph	0.000385	0.443206284	down
ASMM9PARTA020245	Ear12	0.0000513	0.347774919	down
ASMM9PARTA026757	Gm6537	0.0000844	0.385755634	down
ASMM9PARTA032028	Chrna4	0.0000397	0.444166377	down
ASMM9PARTA028822	Ugt2b5	0.000019	0.434144039	down
ASMM9PARTA040779	Zc3h12a	0.012332202	0.425404348	down
ASMM9PARTA030539	Anxa1	0.000124	0.475038553	down
ASMM9PARTA029843	En2	0.000000526	0.152041445	down
ASMM9PARTA020689	C1ql4	0.000826	0.110243908	down
ASMM9PARTA027052	Mup14	0.000114	0.366819086	down
ASMM9PARTA026834	Kif17	0.000303	0.495580979	down
ASMM9PARTA027745	Gabra6	0.02647982	0.297270374	down
ASMM9PARTA044638	Dgkq	0.000725	0.416558014	down
ASMM9PARTA020203	Gm52	0.001634004	0.378973202	down
ASMM9PARTA021544	Mup2	0.000031	0.482484021	down
ASMM9PARTA037771	Tcfap4	0.03489789	0.365331942	down
ASMM9PARTA021929	Myst3	0.035654817	0.449276261	down
ASMM9PARTA027159	Car3	0.000916	0.472998189	down
ASMM9PARTA028274	Foxa1	0.0000641	0.04409557	down
ASMM9PARTA044658	C1ql2	0.000000539	0.291101468	down
ASMM9PARTA032061	Casp9	0.000298	0.391780231	down
ASMM9PARTA025578	Ucma	0.015660925	0.392897004	down
ASMM9PARTA032995	Sec1	0.000187	0.47915595	down
ASMM9PARTA037618	Akr1c6	0.000444	0.374077842	down
ASMM9PARTA019886	Mup21	0.002456202	0.374074498	down
ASMM9PARTA027253	Gpr182	0.000607	0.382188459	down
ASMM9PARTA029960	Irx1	0.00000197	0.101702898	down
ASMM9PARTA038073	Bbox1	0.002758299	0.352427876	down
ASMM9PARTA038178	Tshz2	0.0000161	0.496786808	down
ASMM9PARTA035483	Cd209g	0.000181	0.430250685	down
ASMM9PARTA030847	Slc34a1	0.028431648	0.457368876	down
ASMM9PARTA029298	Ch25h	0.0000136	0.326547108	down
ASMM9PARTA028551	Tnfrsf11b	0.019162202	0.411245255	down
ASMM9PARTA023494	Wtap	0.000156	0.054112785	down
ASMM9PARTA039049	Fut10	0.0000154	0.45058989	down
ASMM9PARTA036766	Dmgdh	0.008427964	0.448133821	down
ASMM9PARTA028990	Slc22a1	0.018043306	0.486755339	down
ASMM9PARTA042640	Mael	0.0000309	0.306909876	down
ASMM9PARTA027349	LOC100048884	0.00000302	0.461396479	down
ASMM9PARTA037449	Sucnr1	0.002563587	0.389406356	down
ASMM9PARTA030926	Xdh	0.00000699	0.288572456	down

ASMM9PARTA031728	S100a8	0.00055	0.468861941	down
ASMM9PARTA020403	Mup20	0.0000229	0.276195118	down
ASMM9PARTA032320	Vpreb2	0.04752366	0.292196746	down
ASMM9PARTA025706	Trim45	0.017479895	0.426420794	down
ASMM9PARTA029800	Cd83	0.007302238	0.488898102	down
ASMM9PARTA031250	Plscr1	0.00000565	0.433983607	down
ASMM9PARTA023085	Krtap17-1	0.041328	0.461730613	down
ASMM9PARTA027595	Foxd4	0.000648	0.206476166	down
ASMM9PARTA036347	Dhrs2	0.0000551	0.360204804	down
ASMM9PARTA030053	Fcnb	0.044042222	0.409078298	down
ASMM9PARTA039113	BC021614	0.01366014	0.443285401	down
ASMM9PARTA029700	Usp17l5	0.033942975	0.311348041	down
ASMM9PARTA022964	Phldb3	0.002448608	0.462279239	down
ASMM9PARTA029143	Tjp1	0.027230859	0.378267546	down
ASMM9PARTA022115	Ankrd44	0.009138472	0.492206404	down
ASMM9PARTA030909	S100a5	0.0000843	0.011423776	down
ASMM9PARTA023096	Vmn2r5	0.008717687	0.480057453	down
ASMM9PARTA033209	Kcne4	0.00011	0.454853825	down
ASMM9PARTA042746	H2-M10.5	0.036926385	0.375847258	down
ASMM9PARTA032569	Il22	0.000457	0.351017538	down
ASMM9PARTA031128	Tal1	0.0000107	0.225453776	down
ASMM9PARTA025388	Prph	0.000134	0.435001944	down
ASMM9PARTA021303	Gm904	0.009609754	0.370477631	down
ASMM9PARTA032496	Ear7	0.001011707	0.297193451	down
ASMM9PARTA021266	Il20rb	0.000132	0.454482942	down
ASMM9PARTA027690	Ear2	0.0000152	0.341822378	down
ASMM9PARTA038827	Fgg	0.000798	0.49645038	down
ASMM9PARTA037765	Mesdc1	0.0000112	0.366369077	down
ASMM9PARTA038122	Fkbp6	0.0222166	0.478177074	down
ASMM9PARTA043136	Rassf4	0.04122614	0.484676234	down
ASMM9PARTA036809	4930503E14Rik	0.000062	0.315516804	down
ASMM9PARTA036389	Ppm1j	0.000206	0.349679486	down
ASMM9PARTA027972	Gzmk	0.0000815	0.291374553	down
ASMM9PARTA030501	Klk1b26	0.003923854	0.423787408	down
ASMM9PARTA026905	Beta-s	0.0000326	0.445792422	down
ASMM9PARTA034826	1300014I06Rik	0.0000687	0.04382009	down
ASMM9PARTA038740	Zfp192	0.013429289	0.419474823	down
ASMM9PARTA024981	Mup1	0.0000115	0.366565784	down
ASMM9PARTA021726	C030030A07Rik	0.000001	0.16708227	down
ASMM9PARTA038130	Ear10	0.000524	0.341459687	down
ASMM9PARTA044351	Tanc1	0.004578317	0.480016344	down
ASMM9PARTA032436	Irx4	0.000000355	0.084356145	down
ASMM9PARTA028279	Irx3	0.000000254	0.082131382	down
ASMM9PARTA038363	Slc17a6	0.00000402	0.368558876	down
ASMM9PARTA043669	Retnlg	0.001435137	0.456194681	down
ASMM9PARTA035887	Cage1	0.001942557	0.232231482	down
ASMM9PARTA031936	Shox2	0.0000877	0.215286412	down
ASMM9PARTA033853	Pde6h	0.001855051	0.389529706	down
ASMM9PARTA032344	Agxt	0.006130318	0.497397344	down
ASMM9PARTA043644	Lhfpl1	0.014803316	0.465158814	down
ASMM9PARTA029204	Serpina1a	0.00000126	0.435796879	down
ASMM9PARTA028237	Hoxb6	0.000883	0.466255091	down
ASMM9PARTA021102	Slc38a6	0.0000172	0.38502895	down
ASMM9PARTA039245	Ccdc21	0.0000227	0.170697139	down
ASMM9PARTA034600	Camk2n1	0.00000129	0.210967349	down
ASMM9PARTA038898	Il33	0.0000193	0.312992435	down
ASMM9PARTA039789	Cyp2c70	0.009758089	0.395938871	down

ASMM9PARTA028541	Foxd2	0.0000134	0.422191108	down
ASMM9PARTA042427	Slc25a41	0.029000202	0.331379739	down
ASMM9PARTA028427	Klra2	0.000152	0.291645437	down
ASMM9PARTA022135	Cytl1	0.0000208	0.385958282	down
ASMM9PARTA029321	Wnt6	0.0000202	0.38973113	down
ASMM9PARTA031372	Cep110	0.003978495	0.499116489	down
ASMM9PARTA033242	Ucn	0.000032	0.25719136	down
ASMM9PARTA029892	H2-Q1	0.00000364	0.349764329	down
ASMM9PARTA030248	Hand2	0.008195564	0.473641563	down
ASMM9PARTA021920	Fam65c	0.004216134	0.467311207	down
ASMM9PARTA030563	Pax7	0.000357	0.163592866	down
ASMM9PARTA044022	Spink8	0.0000248	0.377640923	down
ASMM9PARTA028217	Lhx5	0.029400224	0.426123737	down
ASMM9PARTA022057	Ccdc90a	0.0000931	0.470118427	down
ASMM9PARTA022848	Gm10375	0.011573165	0.391472124	down
ASMM9PARTA041875	Lmln	0.023934191	0.466228809	down
ASMM9PARTA029669	Lefty1	0.0000246	0.296821252	down
ASMM9PARTA030278	Foxd3	7.76E-08	0.118712285	down
ASMM9PARTA033719	Apoc3	0.0000816	0.472522889	down
ASMM9PARTA031523	Rax	0.007013034	0.308461497	down
