# Supplementary material for: Microarray Profile of Long Noncoding RNA and Messenger RNA Expression in a Model of Alzheimer’s Disease
Source: Life (Basel). 2020 May 14;10(5):64. doi: 10.3390/life10050064 (PMC7281340; doi:10.3390/life10050064)
Supplement: Supplementary file 1 [file life-10-00064-s001.zip › life-787240-supplemenatry-to be published - PDF/life-787240-supplementary/Table S6.pdf]

life

Supplementary

Microarray Profile of Long Noncoding RNA and Messenger RNA Expression in a Model of Alzheimer's Disease

Linlin Wang †, Li Zeng †, Hailun Jiang, Zhuorong Li * and Rui Liu *

Institute of Medicinal Biotechnology, Chinese Academy of Medical Sciences and Peking Union Medical College, Beijing 100050, China; wanglinlin@wfmc.edu.cn (L.W.); zengsheng@imb.pumc.edu.cn (L.Z.); jianghailun@imb.pumc.edu.cn (H.J.)

* Correspondence: lizhourong@imb.pumc.edu.cn (Z.L.); +86-10-8352017; .liurui@imb.pumc.edu.cn (R.L.); Tel.: +86-10-67087731

Table S6. Differently expressed mRNAs in the brain of 3-month-old APP/PS1 mice compared with age-matched WT mice.

Probe Name	Symbol	*p*-Value	Fold Change	Regulation
ASMM9PARTA034335	Rexo1	0.0000776	2.0131881	up
ASMM9PARTA020859	Golm1	0.000497	2.124896	up
ASMM9PARTA026649	Gm4858	0.035422985	2.2425082	up
ASMM9PARTA025747	Tshb	0.000261	7.1079755	up
ASMM9PARTA033120	Cbln3	0.000000332	31.41241	up
ASMM9PARTA044292	Zhx2	0.004505109	3.8538814	up
ASMM9PARTA039095	Dbh	0.00000468	10.825143	up
ASMM9PARTA030304	Mycs	0.014476405	2.687255	up
ASMM9PARTA042374	Mpzl3	0.00131254	2.577886	up
ASMM9PARTA028562	Pten	0.001236823	2.0602944	up
ASMM9PARTA027356	Rapgef4	0.000289	2.3592978	up
ASMM9PARTA030055	Gpd1	0.0000947	2.3032591	up
ASMM9PARTA043927	Synm	0.023921505	2.5987158	up
ASMM9PARTA033070	Mknk2	0.020674184	2.598206	up
ASMM9PARTA026869	Dnmt1	0.028534383	2.0691738	up
ASMM9PARTA044461	Rsl24d1	0.00000256	3.8246887	up
ASMM9PARTA044865	Lpcat4	0.0000565	2.7018802	up
ASMM9PARTA025964	Atp2b4	0.00000577	2.1404495	up
ASMM9PARTA021472	Serpinb6e	0.019518634	2.115108	up
ASMM9PARTA031219	Klk7	0.03606632	2.0841243	up
ASMM9PARTA022668	Gm5634	0.005937275	2.631999	up
ASMM9PARTA025718	Cdr1	0.000378	4.950154	up
ASMM9PARTA043488	Slfn8	0.01830712	2.6219857	up
ASMM9PARTA022401	Crygc	0.002933367	2.2056231	up
ASMM9PARTA027615	Cdh15	0.026808225	2.6264644	up
ASMM9PARTA034297	Baiap2l1	0.009151345	2.9503748	up
ASMM9PARTA043897	Vps39	0.002854362	2.7100213	up
ASMM9PARTA020618	Nxf3	0.020358928	3.0989316	up
ASMM9PARTA044055	Abhd16b	0.020655252	2.42075	up

ASMM9PARTA033443	B4galt1	0.009779065	2.0191495	up
ASMM9PARTA041340	Phc3	0.011523894	3.0563898	up
ASMM9PARTA029291	S100g	0.000883	4.2812963	up
ASMM9PARTA026193	Tmem11	0.00292268	2.7385876	up
ASMM9PARTA035974	Dzip3	0.000309	2.084709	up
ASMM9PARTA029247	Srpk2	0.001399339	2.0067003	up
ASMM9PARTA043891	Nxph4	0.000252	3.0350542	up
ASMM9PARTA022309	Tsc22d2	0.007475972	5.3817773	up
ASMM9PARTA028723	Pou3f2	0.000298	2.6886995	up
ASMM9PARTA024120	Bcl2l15	0.000000657	9.992126	up
ASMM9PARTA021097	Trpm3	0.010695213	3.1141548	up
ASMM9PARTA029691	Cd44	0.005121916	2.274645	up
ASMM9PARTA023315	Gatad2a	0.011054924	2.5039454	up
ASMM9PARTA031792	Ar	0.016093018	3.4225528	up
ASMM9PARTA042087	Fry	0.018274065	2.550512	up
ASMM9PARTA039655	Cald1	0.018220231	2.2480915	up
ASMM9PARTA035507	Otop3	0.000654	2.1003282	up
ASMM9PARTA024171	Homer3	0.0000226	2.4348607	up
ASMM9PARTA034391	1700011A15Rik	0.005382269	3.5566006	up
ASMM9PARTA027268	Ceacam10	0.000935	2.1249924	up
ASMM9PARTA042058	Mon1b	0.04101522	2.0168517	up
ASMM9PARTA031518	Spnb1	0.000387	3.5507712	up
ASMM9PARTA030135	Elavl3	0.00000108	3.0500207	up
ASMM9PARTA044228	Ece1	0.000386	3.2694302	up
ASMM9PARTA030907	Rgs16	0.014481483	2.2052946	up
ASMM9PARTA023951	Grm5	0.0000307	2.764177	up
ASMM9PARTA029461	Vmn2r42	0.024426727	2.5667095	up
ASMM9PARTA037234	Pcdh1	0.001407658	2.9612257	up
ASMM9PARTA022034	Upf2	0.001445179	2.0416608	up
ASMM9PARTA044023	E030010A14Rik	0.000089	3.3643317	up
ASMM9PARTA028727	Psen1	0.014514961	2.0423884	up
ASMM9PARTA027704	Grid2	0.0000396	2.4946718	up
ASMM9PARTA038866	1110008J03Rik	0.000974	2.5506053	up
ASMM9PARTA019955	Lrrc52	0.000000984	2.2423987	up
ASMM9PARTA030353	Klk1b1	0.00000725	3.0717392	up
ASMM9PARTA032069	Gpc3	0.000463	5.734672	up
ASMM9PARTA023378	Hmg1l1	0.0000139	2.006031	up
ASMM9PARTA028078	Lif	0.040207397	2.4267874	up
ASMM9PARTA030219	Hoxc13	0.022291115	2.220715	up
ASMM9PARTA033439	Tsga8	0.024242368	2.0121956	up
ASMM9PARTA033035	Olfr749	0.034461096	2.197205	up
ASMM9PARTA033204	Angptl4	0.000976	2.0785153	up
ASMM9PARTA027577	Cdh16	0.0000298	2.500842	up
ASMM9PARTA031864	Ltbp2	0.003603911	2.351251	up
ASMM9PARTA042776	Gpr123	0.0000174	2.700068	up
ASMM9PARTA033437	Il21	0.036489565	2.444443	up
ASMM9PARTA038785	Krt25	0.00000162	7.0936074	up
ASMM9PARTA033390	Rqcd1	0.004415415	5.4435105	up
ASMM9PARTA033704	Sync	0.001493227	4.5090194	up
ASMM9PARTA031529	Zfp59	0.043619093	2.1645434	up
ASMM9PARTA040166	Olfr122	0.001698892	2.2567854	up
ASMM9PARTA037334	Mccc2	0.000131	3.4796681	up
ASMM9PARTA029737	Dlx5	0.00000128	3.2918766	up
ASMM9PARTA030734	Ccl12	0.0000128	2.8336325	up
ASMM9PARTA039194	Vmn1r203	0.04366745	2.4464188	up
ASMM9PARTA039964	Olfr284	0.013073212	2.4551265	up
ASMM9PARTA031288	Map3k1	0.01629606	2.120114	up

ASMM9PARTA038671	D17H6S56E-3	0.001084572	2.7253125	up
ASMM9PARTA027377	Cebpd	0.0000837	2.2806914	up
ASMM9PARTA027016	Apba2	0.000041	2.1796477	up
ASMM9PARTA024887	Ccdc90b	0.001811773	4.9244823	up
ASMM9PARTA021373	Akap2	0.0000136	2.826316	up
ASMM9PARTA033834	2010106G01Rik	0.000412	3.6309698	up
ASMM9PARTA023274	Mettl8	0.00927067	2.0223105	up
ASMM9PARTA038903	Lbr	0.007786574	3.6255786	up
ASMM9PARTA041614	Itpk1	0.001825231	2.00979	up
ASMM9PARTA040339	Olfr196	0.000934	2.6193984	up
ASMM9PARTA035587	Lce1d	0.02206017	2.868261	up
ASMM9PARTA032481	Cbln1	0.00000695	6.124395	up
ASMM9PARTA033582	Slc18a3	0.000162	2.2332523	up
ASMM9PARTA023587	Schip1	0.001891412	3.0521924	up
ASMM9PARTA023477	Slc14a2	0.004263157	2.3148105	up
ASMM9PARTA035767	Zfp715	0.000929	2.572336	up
ASMM9PARTA043312	Apol10b	0.018448599	2.277396	up
ASMM9PARTA042964	Ep300	0.000918	2.258293	up
ASMM9PARTA029841	Eif4ebp2	0.002615104	2.7991123	up
ASMM9PARTA037995	Clec2h	0.01248099	2.3497608	up
ASMM9PARTA023701	Pcp2	0.000000619	25.212336	up
ASMM9PARTA040966	Cxcl17	0.007967323	3.5091264	up
ASMM9PARTA043668	Edc4	0.006350893	2.4462724	up
ASMM9PARTA031730	Sp1	0.000372	2.3515835	up
ASMM9PARTA036365	Zfp618	0.000288	3.2602153	up
ASMM9PARTA031057	Ubtf	0.0000546	2.523026	up
ASMM9PARTA037939	Prl2c2	0.001083014	3.109749	up
ASMM9PARTA022248	C2cd4b	0.000107	2.0825863	up
ASMM9PARTA041564	Spata2	0.008401684	2.6905072	up
ASMM9PARTA037407	2310079F23Rik	0.000124	2.1839232	up
ASMM9PARTA028440	Mybl1	0.023467902	2.028853	up
ASMM9PARTA030400	Mab21l1	0.0000331	3.0003905	up
ASMM9PARTA038572	Tspan1	0.034594834	2.594937	up
ASMM9PARTA042579	Tmem169	0.00093	2.6716704	up
ASMM9PARTA037599	Trdn	0.0000921	2.4758415	up
ASMM9PARTA024222	Cntfr	0.000000512	21.353983	up
ASMM9PARTA021485	Elovl1	0.006396808	2.1231952	up
ASMM9PARTA029305	Emp1	0.023290558	2.564442	up
ASMM9PARTA021180	Ceacam1	0.026830751	2.2394369	up
ASMM9PARTA027678	Bmp5	0.044724654	2.2822754	up
ASMM9PARTA034817	4921517D21Rik	0.001154023	2.7680027	up
ASMM9PARTA025907	Phc3	0.038478695	2.958142	up
ASMM9PARTA038817	Chodl	0.0000495	2.328985	up
ASMM9PARTA043857	Uts2d	0.000000739	7.9098063	up
ASMM9PARTA037047	4931428F04Rik	0.031373866	2.776208	up
ASMM9PARTA028903	Phox2a	0.000243	4.1923914	up
ASMM9PARTA042512	9230105E10Rik	0.02026018	2.2860644	up
ASMM9PARTA030005	Hipk1	0.00000818	3.7615056	up
ASMM9PARTA022678	Prrt4	0.01517127	2.4829762	up
ASMM9PARTA022707	Krtap9-5	0.000147	4.1937265	up
ASMM9PARTA030244	Gsr	0.0000176	8.693484	up
ASMM9PARTA030760	Prl	0.0000032	3.077553	up
ASMM9PARTA023344	Keap1	0.010525669	2.0258892	up
ASMM9PARTA030241	Gnai1	0.00000248	2.1913018	up
ASMM9PARTA043415	Prpf39	0.000218	3.234354	up
ASMM9PARTA029529	Dnase1	0.00255611	2.0792966	up
ASMM9PARTA040116	Olfr694	0.017981159	2.7239904	up

ASMM9PARTA020086	Whrn	0.004930127	2.1689317	up
ASMM9PARTA020305	5730409E04Rik	0.00921163	2.3247058	up
ASMM9PARTA042538	Grik4	0.025485823	2.4263203	up
ASMM9PARTA020624	Gal3st3	0.000868	4.257045	up
ASMM9PARTA022377	Slc4a11	0.006145392	2.10843	up
ASMM9PARTA038269	Clec2d	0.0000681	2.1642687	up
ASMM9PARTA019864	Olfr279	0.021622322	2.0405638	up
ASMM9PARTA021401	Sco1	0.011102481	2.7820432	up
ASMM9PARTA023023	D3Ertd254e	0.04715396	2.0502346	up
ASMM9PARTA026309	Rbms3	0.003286623	2.7078137	up
ASMM9PARTA041163	Ccdc137	0.007228469	3.271304	up
ASMM9PARTA041255	A630007B06Rik	0.003440239	2.083166	up
ASMM9PARTA021014	Zfc3h1	0.002089937	2.2518246	up
ASMM9PARTA023270	Wdr36	0.00000589	2.239158	up
ASMM9PARTA041305	Cpne5	0.0000197	2.3959188	up
ASMM9PARTA033677	Stk25	0.00000142	2.2109196	up
ASMM9PARTA041954	Rln3	0.0000113	11.949367	up
ASMM9PARTA031964	Tmod4	0.003189421	2.0860846	up
ASMM9PARTA024773	Myef2	0.000247	2.0085769	up
ASMM9PARTA029755	Azi1	0.039897278	2.3221018	up
ASMM9PARTA021805	Sema3b	0.017367177	2.0740938	up
ASMM9PARTA031919	Atp2a3	0.012361143	2.219233	up
ASMM9PARTA042749	Actbl2	0.018392757	2.1176643	up
ASMM9PARTA020588	Chka	0.00000246	2.159946	up
ASMM9PARTA040025	2610301G19Rik	0.000672	2.2444727	up
ASMM9PARTA031110	Ywhaz	0.00000022	4.974651	up
ASMM9PARTA039855	Fam199x	0.003728605	3.1842735	up
ASMM9PARTA038885	Pcdha12	0.000652	2.2069955	up
ASMM9PARTA020210	2700007P21Rik	0.000505	6.5304055	up
ASMM9PARTA033314	Jmy	0.005640682	2.384646	up
ASMM9PARTA030680	Neurod1	0.00000557	3.6622305	up
ASMM9PARTA040122	Cacnb4	0.0000269	2.2965052	up
ASMM9PARTA033174	Ntn4	0.003987917	2.1934178	up
ASMM9PARTA020841	Plekhg1	0.006748208	2.8092928	up
ASMM9PARTA033354	Rhbg	0.022154806	3.0685782	up
ASMM9PARTA041859	Larp4b	0.004059287	2.7204006	up
ASMM9PARTA043849	Duxbl	0.001021026	2.7272217	up
ASMM9PARTA039802	Il17re	0.0000526	2.1716788	up
ASMM9PARTA044087	4931429L15Rik	0.04735245	2.1686256	up
ASMM9PARTA030614	Oc90	0.00645964	4.360575	up
ASMM9PARTA021995	Cep76	0.0000501	2.040232	up
ASMM9PARTA025140	Rrp1b	0.023219869	2.0569618	up
ASMM9PARTA037729	Apobec1	0.012179105	2.7671332	up
ASMM9PARTA031745	Fbxo6	0.031565513	2.1618094	up
ASMM9PARTA037840	Akap12	0.0000175	7.435588	up
ASMM9PARTA020908	Dnajb14	0.007936576	2.1309862	up
ASMM9PARTA025186	Krtap20-2	0.019794378	2.4835892	up
ASMM9PARTA020953	Ralgapa2	0.0000133	2.5162442	up
ASMM9PARTA019915	Zfyve26	0.009721629	2.249106	up
ASMM9PARTA042120	Frmd3	0.000357	2.1188087	up
ASMM9PARTA036961	1700001C19Rik	0.003972282	2.1194308	up
ASMM9PARTA038935	Ehd4	0.000731	3.6810253	up
ASMM9PARTA042571	Ankrd34b	0.009604597	2.5402524	up
ASMM9PARTA037836	F13b	0.006615834	2.5302463	up
ASMM9PARTA021982	Gpr155	0.016467305	2.3568723	up
ASMM9PARTA042755	Cnpy1	6.15E-09	9.576895	up
ASMM9PARTA019700	Amica1	0.018433895	3.9024458	up

ASMM9PARTA027224	Taok3	0.01789806	2.4219186	up
ASMM9PARTA031132	Cd70	0.00000529	7.2947025	up
ASMM9PARTA036338	Dzip1l	0.013755283	2.3049047	up
ASMM9PARTA042613	Pcnxl2	0.003755847	2.701153	up
ASMM9PARTA030911	St3gal5	0.007328525	2.544103	up
ASMM9PARTA024346	Zkscan3	0.04097148	3.0041509	up
ASMM9PARTA042382	Clcn3	0.0000302	2.0260973	up
ASMM9PARTA024808	Ttc15	4.86E-08	104.09621	up
ASMM9PARTA022470	Acin1	0.0000727	3.3000937	up
ASMM9PARTA029776	Dao	0.000003	7.0075417	up
ASMM9PARTA039500	Hnrnpul1	0.0000777	2.402664	up
ASMM9PARTA038655	Ugt2b38	0.033007827	2.453221	up
ASMM9PARTA021040	Trpm3	0.004312711	8.494691	up
ASMM9PARTA029136	Svs5	0.00000243	5.0985413	up
ASMM9PARTA042786	Mrgprb2	0.017246226	3.2687197	up
ASMM9PARTA044237	Tspan15	0.0000828	3.296772	up
ASMM9PARTA037352	Bicd2	0.001428681	2.7240407	up
ASMM9PARTA023156	Steap2	0.020860549	2.079492	up
ASMM9PARTA022560	Ncam1	0.000208	2.2651258	up
ASMM9PARTA037824	1700061G19Rik	0.02579874	2.0191689	up
ASMM9PARTA031043	Wdr1	0.00000761	2.3161047	up
ASMM9PARTA032065	Dlc1	0.000188	4.88514	up
ASMM9PARTA034262	Srsf9	0.0000446	3.1864085	up
ASMM9PARTA037902	Senp3	0.022381496	2.1704063	up
ASMM9PARTA027033	Kcnmb3	0.005849643	3.108935	up
ASMM9PARTA039089	Triobp	0.029576972	2.9908319	up
ASMM9PARTA042748	Zfp367	0.00054	2.1110482	up
ASMM9PARTA042303	Rab8b	0.012267113	2.140596	up
ASMM9PARTA031678	Figla	0.04417995	2.6710413	up
ASMM9PARTA035267	Ribc2	0.017934376	2.3886616	up
ASMM9PARTA025590	Bcas3	0.025509913	2.4655817	up
ASMM9PARTA020151	Taar2	0.002315064	3.5115612	up
ASMM9PARTA038388	Scgb3a1	0.025819426	2.0246587	up
ASMM9PARTA025390	Chn2	0.00000163	2.5671134	up
ASMM9PARTA020325	Olfr288	0.000053	3.2774408	up
ASMM9PARTA039455	Galnt7	0.0000329	6.361879	up
ASMM9PARTA028888	Slc1a6	0.00000428	2.3305912	up
ASMM9PARTA029080	Zic1	0.000000692	2.1378815	up
ASMM9PARTA032766	Slc5a1	0.006583559	2.0172143	up
ASMM9PARTA038418	Pip4k2c	0.01396793	2.3580341	up
ASMM9PARTA029599	Brd2	0.004400514	2.2205582	up
ASMM9PARTA044093	Kcnv2	0.01415794	3.1431258	up
ASMM9PARTA026094	Plscr3	0.005433633	2.009998	up
ASMM9PARTA020744	Ccdc141	0.000738	3.8202212	up
ASMM9PARTA042368	Ccdc88a	0.000716	2.8191738	up
ASMM9PARTA039075	Cyyr1	0.003206401	3.874123	up
ASMM9PARTA043130	Exoc3l	0.003598359	3.419214	up
ASMM9PARTA020725	Crls1	0.00000938	2.3094146	up
ASMM9PARTA022705	Gm436	0.000322	5.842333	up
ASMM9PARTA020793	Dusp27	0.035250545	2.0139375	up
ASMM9PARTA033406	Sap30	0.00000588	2.3725853	up
ASMM9PARTA019738	Rhox8	0.00616448	2.5611932	up
ASMM9PARTA022123	Ash2l	0.042711552	4.4007535	up
ASMM9PARTA021003	Trpm3	0.000169	5.5811014	up
ASMM9PARTA028835	Ppl	0.001390428	2.8787844	up
ASMM9PARTA040971	Olfr178	0.033310827	2.611279	up
ASMM9PARTA029802	Cdkn1b	0.044495378	2.1857147	up

ASMM9PARTA024271	Pax3	0.001955256	2.8865151	up
ASMM9PARTA024457	Fas	0.0000141	2.75859	up
ASMM9PARTA020936	Calca	0.0000145	2.3954597	up
ASMM9PARTA030813	Stat5b	0.00499773	2.0365803	up
ASMM9PARTA024760	Atp2a3	0.000252	3.25739	up
ASMM9PARTA026365	Arpc4	0.028725602	3.921665	up
ASMM9PARTA029490	Dag1	0.013240523	2.0206296	up
ASMM9PARTA042277	St18	0.00024	2.536438	up
ASMM9PARTA025384	Puf60	0.00000617	2.2255862	up
ASMM9PARTA032045	Uncx	0.0000432	2.0601583	up
ASMM9PARTA030329	Zbtb7a	0.00000311	2.444849	up
ASMM9PARTA041502	Snx30	0.000237	2.8903682	up
ASMM9PARTA023018	Gabra6	0.000000593	45.63106	up
ASMM9PARTA030388	Igfbp5	0.04744752	2.3861468	up
ASMM9PARTA021744	Xlr5a	0.027561773	3.5007346	up
ASMM9PARTA031158	Saa3	0.000092	3.4086795	up
ASMM9PARTA043447	Grm3	0.0000094	2.6368442	up
ASMM9PARTA028683	Pcp2	0.000574	2.5662684	up
ASMM9PARTA045008	Dpp6	0.0000211	2.1643553	up
ASMM9PARTA041081	Olfr711	0.030779494	2.0663273	up
ASMM9PARTA026355	Tfdp2	0.041753415	3.2512505	up
ASMM9PARTA033151	Rbfox1	0.000000545	8.581732	up
ASMM9PARTA035464	Srrm4	0.0000115	2.044778	up
ASMM9PARTA029782	Dffa	0.000151	2.584579	up
ASMM9PARTA038007	Sf3b1	0.000002	2.5554943	up
ASMM9PARTA044527	Tex2	0.000131	2.2574747	up
ASMM9PARTA029581	Cga	0.001247127	2.0177164	up
ASMM9PARTA033642	Clstn1	0.000517	2.3315802	up
ASMM9PARTA020822	Gm694	0.0000146	2.0105753	up
ASMM9PARTA030500	Klk1b11	0.000000237	2.9923458	up
ASMM9PARTA032269	Ddc	0.002748172	2.1244123	up
ASMM9PARTA042600	Srrm2	0.000000588	2.1800425	up
ASMM9PARTA039943	Olah	0.011828708	2.0999353	up
ASMM9PARTA038207	Calcb	0.00598834	2.010279	up
ASMM9PARTA030188	Mecp2	0.000000383	2.2802384	up
ASMM9PARTA041004	2210018M11Rik	0.037067667	2.0723267	up
ASMM9PARTA030775	Sftpc	0.01568264	2.4331853	up
ASMM9PARTA024458	Casz1	0.005312582	2.271022	up
ASMM9PARTA027171	App	0.0000321	2.267833	up
ASMM9PARTA038850	Gldc	0.019490713	2.4809875	up
ASMM9PARTA038170	Rims1	0.000779	2.1298378	up
ASMM9PARTA026421	Plch1	0.006341245	2.4133396	up
ASMM9PARTA029664	Cyp2b10	0.043919038	2.1898785	up
ASMM9PARTA026908	Fxyd2	0.015080172	3.4752994	up
ASMM9PARTA019935	Txlna	0.00000348	6.327588	up
ASMM9PARTA027734	Ebf1	0.006859016	2.664445	up
ASMM9PARTA043988	Actl9	0.04139674	2.1593118	up
ASMM9PARTA044488	Ostn	0.0000927	2.5186367	up
ASMM9PARTA030282	Hp1bp3	0.000633	3.4439776	up
ASMM9PARTA033144	Accn5	0.005091173	2.6091871	up
ASMM9PARTA045012	Olfr1321	0.020510351	2.1040514	up
ASMM9PARTA026178	Rbms3	0.000528	2.580435	up
ASMM9PARTA027749	Gh	0.000000477	6.586476	up
ASMM9PARTA020962	Akap2	0.00000119	2.3027155	up
ASMM9PARTA030450	Gsbs	0.0000045	3.9371283	up
ASMM9PARTA036674	Psca	0.04340225	2.665277	up
ASMM9PARTA025585	Slc38a1	0.01696703	2.1143532	up

ASMM9PARTA031638	Uts2	0.0000771	11.368202	up
ASMM9PARTA023362	Qrich1	0.006923941	2.5978963	up
ASMM9PARTA023233	Skor2	0.000428	4.101804	up
ASMM9PARTA033226	Sdf2l1	8.46E-08	3.1844397	up
ASMM9PARTA037810	Usp26	0.004630403	2.0790303	up
ASMM9PARTA035707	4921528I01Rik	0.02707344	2.1914601	up
ASMM9PARTA027331	Btg1	0.020830113	2.2247374	up
ASMM9PARTA027827	Cd38	0.001257424	2.0815308	up
ASMM9PARTA024004	Dedd	0.000289	6.3812113	up
ASMM9PARTA029430	Akap2	0.000023	2.630236	up
ASMM9PARTA027630	Gbas	0.000265	2.0316691	up
ASMM9PARTA022642	Scn2a1	0.0000421	2.0201728	up
ASMM9PARTA041481	Zswim4	0.0000687	2.4257221	up
ASMM9PARTA044533	Zfp933	0.00000407	6.508013	up
ASMM9PARTA022278	Trio	0.000261	2.1059213	up
ASMM9PARTA029751	Alox5	0.005694531	2.5102727	up
ASMM9PARTA033028	Miox	0.0000209	2.7134473	up
ASMM9PARTA037987	Bcl3	0.000000928	2.3982546	up
ASMM9PARTA027912	Dcn	0.03230018	2.1511126	up
ASMM9PARTA042372	6430573F11Rik	0.0000004	5.1928716	up
ASMM9PARTA022716	Cep170	0.019999761	2.7042885	up
ASMM9PARTA043521	Gprc5a	0.0000665	2.73489	up
ASMM9PARTA030995	Sdc4	0.0000116	2.133687	up
ASMM9PARTA023518	Cry2	0.001406526	2.1740687	up
ASMM9PARTA038488	Dpf3	0.000276	2.008091	up
ASMM9PARTA037101	Pibf1	0.003939726	2.0952516	up
ASMM9PARTA042594	Hnrnpl	0.000000979	6.9169126	up
ASMM9PARTA030942	Prnp	0.00000222	2.304952	up
ASMM9PARTA032730	Ppp4c	0.0000744	2.2432292	up
ASMM9PARTA023801	8430427H17Rik	0.002993711	2.563201	up
ASMM9PARTA041201	Rbm45	0.0000085	2.9006667	up
ASMM9PARTA043858	Nhlh2	0.013809101	2.8469856	up
ASMM9PARTA042037	Aqp6	0.0000062	7.5544186	up
ASMM9PARTA033259	Tlx1	0.000000911	8.637319	up
ASMM9PARTA025291	Prph	0.0000025	3.4916854	up
ASMM9PARTA029843	En2	0.00000393	3.2560978	up
ASMM9PARTA020162	Olfr287	0.0000831	2.5563526	up
ASMM9PARTA027745	Gabra6	0.000287	40.717136	up
ASMM9PARTA028216	Lcn2	0.00000166	5.038747	up
ASMM9PARTA036680	Gcap14	0.0000229	2.0007617	up
ASMM9PARTA023569	Rhbdd1	0.044193663	2.053171	up
ASMM9PARTA033589	Eral1	0.000145	2.0973113	up
ASMM9PARTA028676	Rbbp4	0.000014	2.3795025	up
ASMM9PARTA027065	Kcnip1	0.000153	2.501945	up
ASMM9PARTA033269	Klk1b27	0.000233	2.1436794	up
ASMM9PARTA021113	Rbfox3	0.000019	2.5786054	up
ASMM9PARTA032825	Nmu	0.003168894	2.6354065	up
ASMM9PARTA024585	Ptprr	0.006181602	5.7722845	up
ASMM9PARTA022268	4930420K17Rik	0.00000348	2.3743002	up
ASMM9PARTA024350	Lgals3	0.002607916	2.1792047	up
ASMM9PARTA039119	Otub1	0.0000673	3.2754765	up
ASMM9PARTA028752	Olfr15	0.021944268	2.1585197	up
ASMM9PARTA022310	Mfhas1	0.00000266	3.0768075	up
ASMM9PARTA023780	Bcl2l15	0.00000581	6.381978	up
ASMM9PARTA031902	Eif2ak4	0.04231555	2.1145937	up
ASMM9PARTA034497	Arl4d	0.0000233	2.2362335	up
ASMM9PARTA023424	Fcrl5	0.000000589	4.7957745	up

ASMM9PARTA027514	Epx	0.002280834	2.2140546	up
ASMM9PARTA029298	Ch25h	0.00000248	2.6407201	up
ASMM9PARTA025339	Taok2	0.00000441	2.3447692	up
ASMM9PARTA028168	Inhba	0.007865816	4.527508	up
ASMM9PARTA037704	Gigyf1	0.00000187	2.053103	up
ASMM9PARTA020076	Mreg	0.027675852	2.262513	up
ASMM9PARTA023622	Ucma	0.00095	2.022368	up
ASMM9PARTA036721	Ms4a6c	0.00000163	2.016897	up
ASMM9PARTA032636	Zfp326	0.00479378	2.0007935	up
ASMM9PARTA019691	Pcdhac2	0.00000802	2.049352	up
ASMM9PARTA019829	Limch1	0.016721118	2.5045152	up
ASMM9PARTA036204	Cul5	0.007891292	2.0390193	up
ASMM9PARTA020277	Macrod2	0.000331	2.1779914	up
ASMM9PARTA030926	Xdh	0.00000665	2.641078	up
ASMM9PARTA024013	Megf11	0.0000164	2.6995435	up
ASMM9PARTA033890	4930455C21Rik	0.000577	2.4747503	up
ASMM9PARTA029947	Gal	0.0000802	2.4134696	up
ASMM9PARTA033482	Ankrd36	0.017776307	2.115237	up
ASMM9PARTA042242	Clcn3	0.000104	2.057571	up
ASMM9PARTA041245	Nrn1	0.0000133	3.3465772	up
ASMM9PARTA021504	Prl2c1	0.015831161	2.827291	up
ASMM9PARTA043734	Prl2c5	0.00000574	4.5089	up
ASMM9PARTA031598	Schip1	0.00000488	5.9925	up
ASMM9PARTA026984	App	0.00000141	2.2700558	up
ASMM9PARTA032569	Il22	0.00000113	28.121023	up
ASMM9PARTA025388	Prph	0.0000529	2.354521	up
ASMM9PARTA023048	Lyn	0.00415865	3.4413486	up
ASMM9PARTA044577	Dand5	0.01038326	2.4686186	up
ASMM9PARTA028172	Klk1b5	0.000492	2.18727	up
ASMM9PARTA041787	Zfp609	0.027383152	2.2025447	up
ASMM9PARTA042824	Rab11fip4	0.005243877	2.2061822	up
ASMM9PARTA025216	Atp2a3	0.020483285	2.2597837	up
ASMM9PARTA024523	Celf4	0.027295299	2.2297	up
ASMM9PARTA022018	Paip1	0.000975	2.0925379	up
ASMM9PARTA021266	Il20rb	7.98E-08	15.495012	up
ASMM9PARTA042557	Lingo4	0.00009	2.0992484	up
ASMM9PARTA044835	Acsm3	0.011098072	3.2589226	up
ASMM9PARTA043837	Elfn2	0.0000177	2.1600103	up
ASMM9PARTA043268	BC048502	0.001429616	3.2668848	up
ASMM9PARTA035845	Rpf1	0.004638776	2.01361	up
ASMM9PARTA040101	Als2	0.00000759	2.0729184	up
ASMM9PARTA030501	Klk1b26	0.0000112	3.3850658	up
ASMM9PARTA024192	Kif2a	0.006471478	2.356188	up
ASMM9PARTA030989	Stat3	0.0000341	2.0919695	up
ASMM9PARTA032843	Amac1	0.001432844	3.1314535	up
ASMM9PARTA041636	Nup214	0.001879399	2.6814542	up
ASMM9PARTA035781	Prdm16	0.010508808	4.65794	up
ASMM9PARTA030182	Lig1	0.001845976	3.0934188	up
ASMM9PARTA032436	Irx4	0.001233913	2.1811604	up
ASMM9PARTA044591	Plac9	0.000114	2.62367	up
ASMM9PARTA042086	Pcyox1l	0.00006	2.168047	up
ASMM9PARTA039937	Ces2c	0.021259435	2.812997	up
ASMM9PARTA036767	Rnf6	0.000387	2.0789928	up
ASMM9PARTA027019	Calm3	0.000432	2.041094	up
ASMM9PARTA025131	Prl	0.00000679	2.659923	up
ASMM9PARTA019948	Olfr1415	0.0000343	2.0981004	up
ASMM9PARTA019879	Ypel4	0.00000789	2.118269	up

ASMM9PARTA032130	Dmp1	0.00000327	3.0958586	up
ASMM9PARTA037877	Tsga14	0.022264006	2.6934185	up
ASMM9PARTA032235	Prok2	0.001968308	2.548295	up
ASMM9PARTA029091	Sfrp2	0.000742	2.5556793	up
ASMM9PARTA022380	Irs2	0.0000298	2.5150023	up
ASMM9PARTA030109	Hrc	0.000366	5.5128555	up
ASMM9PARTA028348	Hmx3	0.021100137	2.0058124	up
ASMM9PARTA030499	Kcnj12	0.0000302	2.0685737	up
ASMM9PARTA043489	4933402J07Rik	0.0000423	3.640099	up
ASMM9PARTA041434	Nphp3	0.000332	2.961486	up
ASMM9PARTA041883	AI118078	0.020297442	2.475887	up
ASMM9PARTA020861	Alpk2	0.0000717	2.3543413	up
ASMM9PARTA042122	Man2a2	0.000695	3.2351592	up
ASMM9PARTA040038	Olfr657	0.024216658	2.526638	up
ASMM9PARTA028968	Tnnc1	0.0000556	2.721746	up
ASMM9PARTA020569	Thoc2	0.009915572	3.643276	up
ASMM9PARTA027376	Cdkn1a	0.00000449	3.9448357	up
ASMM9PARTA029974	Dlx3	0.01115084	2.501846	up
ASMM9PARTA027262	Car8	0.000000896	10.347539	up
ASMM9PARTA032218	Atoh7	0.001012383	2.1158974	up
ASMM9PARTA026717	Rhox3b	0.002659484	3.030071	up
ASMM9PARTA033272	Vav3	0.004049175	2.184858	up
ASMM9PARTA039324	Afm	0.039152432	2.4595587	up
ASMM9PARTA024680	Reg3d	0.03834498	3.995596	up
ASMM9PARTA041791	Cbln2	0.000367	2.1172154	up
ASMM9PARTA020649	Zbtb16	0.002291162	2.6081762	up
ASMM9PARTA041303	Olfm3	0.0000532	2.670419	up
ASMM9PARTA030114	Il16	0.00000909	2.228048	up
ASMM9PARTA022516	Neto2	0.0000507	2.480403	up
ASMM9PARTA020433	Olfr304	0.015975207	2.2241602	up
ASMM9PARTA038890	Dnajc17	0.04247645	2.2162783	up
ASMM9PARTA028957	Sox11	0.00000391	2.1375854	up
ASMM9PARTA024209	Ttll3	0.0000696	2.4078317	up
ASMM9PARTA042973	Slc9a4	0.03867477	0.376517563	down
ASMM9PARTA044432	4933407P14Rik	0.000379	0.448516093	down
ASMM9PARTA023108	Zfp703	0.03617445	0.33909248	down
ASMM9PARTA025664	Gm6904	0.000615	0.479088414	down
ASMM9PARTA041768	Gbp9	0.000352	0.490601113	down
ASMM9PARTA031904	Skp2	0.02077741	0.445450728	down
ASMM9PARTA039399	Ccdc136	0.039321084	0.392621018	down
ASMM9PARTA022725	Kng2	0.000044	0.433482876	down
ASMM9PARTA028818	Th	0.0000139	0.40541319	down
ASMM9PARTA022035	Kcna10	0.002918199	0.398040241	down
ASMM9PARTA043810	Krtap13-1	0.03193396	0.488030682	down
ASMM9PARTA031995	Postn	0.001804427	0.396111657	down
ASMM9PARTA037112	Rpl18a	3.76E-09	0.066564021	down
ASMM9PARTA021278	Pm20d2	0.011180944	0.494348704	down
ASMM9PARTA029936	Cyp2d9	0.0000192	0.318785199	down
ASMM9PARTA036396	F11	0.021282494	0.464336367	down
ASMM9PARTA029513	Cd3g	0.049232278	0.387342874	down
ASMM9PARTA029838	Cyp2d10	0.000412	0.447776258	down
ASMM9PARTA020724	Lce3f	0.000416	0.243116207	down
ASMM9PARTA023568	Mup10	0.000000135	0.209730382	down
ASMM9PARTA039210	Pth2r	0.029752199	0.322729163	down
ASMM9PARTA028744	Serpina1c	0.00000528	0.236018093	down
ASMM9PARTA029778	Slc6a3	0.00000648	0.350588173	down
ASMM9PARTA034613	Mcm8	0.002277384	0.465156239	down

ASMM9PARTA033715	Foxb1	0.0000722	0.370373402	down
ASMM9PARTA032497	Fabp1	0.0000189	0.255029175	down
ASMM9PARTA042775	Myh8	0.01402403	0.331372086	down
ASMM9PARTA022182	Mup6	0.000305	0.287943503	down
ASMM9PARTA029223	Apoa1	0.000101	0.076311394	down
ASMM9PARTA037527	1700110M21Rik	0.017526075	0.390415975	down
ASMM9PARTA022540	Ttll2	0.015117502	0.437657524	down
ASMM9PARTA028164	Ifit1	0.0000408	0.405080225	down
ASMM9PARTA032773	Scand1	0.001195885	0.285031456	down
ASMM9PARTA022679	Gm15104	0.024647413	0.410321473	down
ASMM9PARTA028385	Hsd3b5	0.000127	0.116294797	down
ASMM9PARTA031062	Tnnt1	0.00000124	0.460475754	down
ASMM9PARTA029830	Chi3l3	0.003989127	0.461662251	down
ASMM9PARTA026369	Fam47e	0.01762749	0.279349319	down
ASMM9PARTA028854	Serpina1b	0.00000759	0.152172108	down
ASMM9PARTA024525	Rdh7	0.000109	0.416357469	down
ASMM9PARTA028615	Mup5	0.000575	0.324830927	down
ASMM9PARTA027299	Alox12	0.003108772	0.47511362	down
ASMM9PARTA040898	Olfr167	0.000482	0.201708674	down
CUST_305_PI426409190	Ccl28	0.002444553	0.3063397	down
ASMM9PARTA021492	Gbp11	0.000042	0.373694033	down
ASMM9PARTA023640	Igsf9b	0.001457356	0.411119635	down
ASMM9PARTA032166	Ahcy	0.000000561	0.493928873	down
ASMM9PARTA022794	Rbm8a	4.41E-08	0.136787083	down
ASMM9PARTA029884	Gata6	0.002253278	0.489105948	down
ASMM9PARTA040145	Olfr360	0.001774364	0.351478653	down
ASMM9PARTA027393	Alx4	0.011402214	0.465946097	down
ASMM9PARTA029029	Serpina1e	0.0000163	0.216747893	down
ASMM9PARTA043514	Fibcd1	0.000000714	0.464382016	down
ASMM9PARTA036982	Trim14	0.001675722	0.26899079	down
ASMM9PARTA023802	LOC100048885	0.00000561	0.472990672	down
ASMM9PARTA029472	Alb	0.0000443	0.10484613	down
ASMM9PARTA043606	Slc25a31	0.002886053	0.491543293	down
ASMM9PARTA023296	Gm3448	0.00000256	0.204340394	down
ASMM9PARTA021801	Lhx9	0.002228591	0.42917895	down
ASMM9PARTA036700	Cep97	0.003294515	0.498130963	down
ASMM9PARTA028759	Serpinf2	0.001516082	0.445709566	down
ASMM9PARTA021558	Lce3a	0.046632025	0.418816031	down
ASMM9PARTA020879	Gpr55	0.034335684	0.496669335	down
ASMM9PARTA027011	Mup15	0.000000216	0.187041643	down
ASMM9PARTA026146	Slc10a1	0.001654563	0.149810162	down
ASMM9PARTA035444	Pnlip	0.001649244	0.468657587	down
ASMM9PARTA038738	Pou4f2	0.00000366	0.265904775	down
ASMM9PARTA031095	Sox14	0.0000334	0.336051542	down
ASMM9PARTA028119	Orm1	0.000292	0.341020643	down
ASMM9PARTA043538	Creb3l2	0.000751	0.439219798	down
ASMM9PARTA027947	Epyc	0.001967873	0.337541269	down
ASMM9PARTA020324	Skap1	0.000839	0.265300947	down
ASMM9PARTA031037	Try4	0.016354358	0.354634728	down
ASMM9PARTA029399	Adora3	0.015459116	0.321788754	down
ASMM9PARTA030022	H2-Q10	0.0000472	0.390551772	down
ASMM9PARTA025627	Vmn1r168	0.0000566	0.433849963	down
ASMM9PARTA042003	Adhfe1	0.000239	0.497323851	down
ASMM9PARTA031618	Cd3d	0.037102643	0.344743148	down
ASMM9PARTA019833	Try5	0.03331522	0.203805165	down
ASMM9PARTA025046	Gm8267	0.00369292	0.453755096	down
ASMM9PARTA040316	Olfr849	0.047231596	0.31355409	down

ASMM9PARTA031514	Phtf1	0.00000299	0.414216078	down
ASMM9PARTA036263	Veph1	0.0000846	0.497557738	down
ASMM9PARTA028234	Hhex	0.0000461	0.385337552	down
ASMM9PARTA030281	Hoxc6	0.039953146	0.434038892	down
ASMM9PARTA044750	Serpinb3c	0.04825284	0.400899699	down
ASMM9PARTA034590	Fam187a	0.0000792	0.2252953	down
ASMM9PARTA023758	Samd3	0.00218318	0.438376726	down
ASMM9PARTA027261	C4bp	0.000519	0.496951377	down
ASMM9PARTA029274	Thrb	0.012458769	0.158537935	down
ASMM9PARTA023546	Serac1	0.031806257	0.267892299	down
ASMM9PARTA040990	Rgs13	0.007759217	0.246271062	down
ASMM9PARTA037288	Ocel1	0.04141541	0.431192779	down
ASMM9PARTA040340	Olfr1238	0.047306612	0.455096204	down
ASMM9PARTA041362	Pus7l	0.025421258	0.435781875	down
ASMM9PARTA031999	Bhmt	0.00000961	0.381095615	down
ASMM9PARTA041165	Mmp21	0.001219689	0.288006223	down
ASMM9PARTA024174	Utp14b	0.001987715	0.356708005	down
ASMM9PARTA028812	Serpina1d	0.0000631	0.1650119	down
ASMM9PARTA020150	Mcoln2	0.002898456	0.402482707	down
ASMM9PARTA030350	Foxa2	0.00000145	0.082502601	down
ASMM9PARTA021692	Mup3	9.91E-08	0.299104064	down
ASMM9PARTA028663	Prg2	0.000000599	0.22314286	down
ASMM9PARTA037956	Ear11	0.003546286	0.306079204	down
ASMM9PARTA035348	Iqcf3	0.0000254	0.239425417	down
ASMM9PARTA028873	Psp	0.010985192	0.365431603	down
ASMM9PARTA024259	Runx2	0.047490794	0.399721043	down
ASMM9PARTA043655	Aldh8a1	0.04789538	0.443317079	down
ASMM9PARTA019794	Zfp457	0.0000181	0.487699226	down
ASMM9PARTA034514	4921530L21Rik	0.000753	0.33422413	down
ASMM9PARTA026360	Zdhhc24	0.005325407	0.440434339	down
ASMM9PARTA025047	1700024P16Rik	0.0000715	0.276017994	down
ASMM9PARTA042990	Mogat2	0.002202216	0.42248706	down
ASMM9PARTA028583	Mup2	0.000000994	0.185906063	down
ASMM9PARTA042987	Myo1d	0.046635848	0.411749181	down
ASMM9PARTA022033	Arfip1	0.000000249	0.38165011	down
ASMM9PARTA027860	Il7r	0.006350752	0.458992782	down
ASMM9PARTA020245	Ear12	0.0000858	0.380890974	down
ASMM9PARTA027403	Cfc1	0.04367976	0.489494206	down
ASMM9PARTA040779	Zc3h12a	0.047002077	0.417886774	down
ASMM9PARTA027052	Mup14	0.000000437	0.163261681	down
ASMM9PARTA027050	4931406C07Rik	0.002749908	0.499306389	down
ASMM9PARTA022613	Gm10922	0.04994502	0.360763076	down
ASMM9PARTA027313	C1qtnf1	0.008685808	0.400488147	down
ASMM9PARTA038186	Cacng6	0.003250514	0.370132005	down
ASMM9PARTA037382	Pbp2	1.58E-09	0.094225015	down
ASMM9PARTA029641	Crisp3	0.001753711	0.343192322	down
ASMM9PARTA021544	Mup2	0.0000151	0.346992123	down
ASMM9PARTA035271	Plxnd1	0.000229	0.382752118	down
ASMM9PARTA021108	Gm6878	0.000862	0.410802327	down
ASMM9PARTA043806	1700067K01Rik	0.000813	0.424293231	down
ASMM9PARTA041493	Igsf11	0.004404892	0.351616698	down
ASMM9PARTA020098	Olfr832	0.013129888	0.463510936	down
ASMM9PARTA026076	Exog	0.003181372	0.411773951	down
ASMM9PARTA028274	Foxa1	0.0000121	0.097204212	down
ASMM9PARTA022904	Vmn2r72-ps	0.014047643	0.353126485	down
ASMM9PARTA044658	C1ql2	0.00000198	0.430703546	down
ASMM9PARTA025240	Zfp605	0.0000662	0.352834297	down

ASMM9PARTA027849	Hcfc1	0.010743204	0.491653204	down
ASMM9PARTA032061	Casp9	0.000454	0.452595386	down
ASMM9PARTA030808	Slfn3	0.033985566	0.399375217	down
ASMM9PARTA025399	Ick	0.015424635	0.391674136	down
ASMM9PARTA040706	Olfr399	0.002206543	0.364324526	down
ASMM9PARTA032995	Sec1	0.000348	0.477181651	down
ASMM9PARTA042840	Casc1	0.029330386	0.412621157	down
ASMM9PARTA021981	Tmpo	0.003964028	0.372571426	down
ASMM9PARTA031165	Snai2	0.012039218	0.323816486	down
ASMM9PARTA037618	Akr1c6	0.002201672	0.324927038	down
ASMM9PARTA019923	Olfr93	0.018084869	0.496448901	down
ASMM9PARTA019886	Mup21	0.000295	0.373076599	down
ASMM9PARTA040373	Olfr539	0.024491915	0.29818416	down
ASMM9PARTA039238	Defb15	0.03907171	0.43839704	down
ASMM9PARTA041571	Mtrr	0.04318515	0.413983022	down
ASMM9PARTA021890	Gm5840	0.0000015	0.434904514	down
ASMM9PARTA022363	Slc43a1	0.009281529	0.467371248	down
ASMM9PARTA029412	Casq1	0.003831348	0.372077573	down
ASMM9PARTA044103	Tcte3	0.0000177	0.499021967	down
ASMM9PARTA038973	Krba1	0.043957543	0.489913418	down
ASMM9PARTA023494	Wtap	2.51E-09	0.03257547	down
ASMM9PARTA028182	Gbp4	0.000146	0.466836888	down
ASMM9PARTA044258	Ugt1a1	0.01841505	0.497798883	down
ASMM9PARTA028739	Cxcl5	0.000019	0.439534937	down
ASMM9PARTA028990	Slc22a1	0.001466898	0.407867037	down
ASMM9PARTA020230	Olfr735	0.00312468	0.339033515	down
ASMM9PARTA042640	Mael	0.0000535	0.284174829	down
ASMM9PARTA037449	Sucnr1	0.005230612	0.398387757	down
ASMM9PARTA037317	Slamf9	0.00411922	0.458642845	down
ASMM9PARTA032949	Fgf21	0.03266398	0.338274303	down
ASMM9PARTA039642	Oas1f	0.003327265	0.388701338	down
ASMM9PARTA020403	Mup20	0.00000871	0.179218166	down
ASMM9PARTA025706	Trim45	0.001484939	0.294947177	down
ASMM9PARTA033936	Lima1	0.021833094	0.326472386	down
ASMM9PARTA027638	H2-Oa	0.020186478	0.331940114	down
ASMM9PARTA024623	Ptprr	0.04364813	0.291228404	down
ASMM9PARTA026872	Olfr612	0.04388193	0.480877197	down
ASMM9PARTA039413	Cdca7l	0.019246167	0.240967032	down
ASMM9PARTA040040	Olfr1128	0.001876537	0.469298537	down
ASMM9PARTA029837	Cyp1a2	0.005328262	0.225261774	down
ASMM9PARTA029700	Usp17l5	0.014676004	0.228703564	down
ASMM9PARTA029143	Tjp1	0.030617232	0.45184729	down
ASMM9PARTA021821	Med1	0.0000362	0.035075464	down
ASMM9PARTA030909	S100a5	0.0000222	0.383845081	down
ASMM9PARTA033209	Kcne4	0.0000232	0.452225435	down
ASMM9PARTA040512	Olfr143	0.035938114	0.45752	down
ASMM9PARTA035615	Krt5	0.034227073	0.198899541	down
ASMM9PARTA042746	H2-M10.5	0.031519182	0.32006893	down
ASMM9PARTA031976	Sgk2	0.002140392	0.420618606	down
ASMM9PARTA034177	Aptx	0.007547147	0.383610399	down
ASMM9PARTA032496	Ear7	0.001177595	0.16413272	down
ASMM9PARTA030415	Pdcd11	0.027891582	0.292071327	down
ASMM9PARTA027690	Ear2	0.000011	0.344345182	down
ASMM9PARTA038827	Fgg	0.00000164	0.235870235	down
ASMM9PARTA038122	Fkbp6	0.000815	0.40510671	down
ASMM9PARTA021314	Timm8a2	0.021009866	0.385281507	down
ASMM9PARTA021733	Cabyr	0.03140675	0.415820576	down

ASMM9PARTA024983	Cd97	0.020184014	0.480554968	down
ASMM9PARTA028309	Hlx	0.004340222	0.289374501	down
ASMM9PARTA020568	Dpcr1	0.00650527	0.406224992	down
ASMM9PARTA034826	1300014I06Rik	0.000457	0.348980405	down
ASMM9PARTA019555	Tas2r144	0.003786296	0.482533913	down
ASMM9PARTA033359	Bcmo1	0.007272051	0.476240073	down
ASMM9PARTA024534	Cox7a2l	0.005117261	0.454848404	down
ASMM9PARTA024981	Mup1	0.00000655	0.196297407	down
ASMM9PARTA021726	C030030A07Rik	0.000000743	0.015542968	down
ASMM9PARTA038214	Ftcd	0.001814448	0.375157238	down
ASMM9PARTA038130	Ear10	0.0000932	0.420623507	down
ASMM9PARTA029005	Uox	0.004563299	0.368850256	down
ASMM9PARTA032311	Sult1d1	0.044592243	0.467944448	down
ASMM9PARTA035887	Cage1	0.030652452	0.299472748	down
ASMM9PARTA033853	Pde6h	0.0000885	0.21165468	down
ASMM9PARTA032344	Agxt	0.000188	0.36290538	down
ASMM9PARTA029204	Serpina1a	0.000000696	0.210762947	down
ASMM9PARTA039245	Ccdc21	0.002202333	0.233532006	down
ASMM9PARTA037688	Fcrls	0.00000356	0.436056348	down
ASMM9PARTA038898	Il33	0.0000442	0.481873927	down
ASMM9PARTA039789	Cyp2c70	0.00587522	0.375815407	down
ASMM9PARTA040805	Prss44	0.005634246	0.454226567	down
ASMM9PARTA028427	Klra2	0.001272043	0.339439372	down
ASMM9PARTA043975	Ccdc67	0.04441505	0.393162464	down
ASMM9PARTA033242	Ucn	0.000166	0.442268894	down
ASMM9PARTA027943	Cyp2a5	0.003612638	0.385093389	down
ASMM9PARTA037198	Slxl1	0.001041755	0.325590498	down
ASMM9PARTA040203	Olfr1123	0.028200556	0.389539038	down
ASMM9PARTA035442	1700113I22Rik	0.020701	0.49252917	down
ASMM9PARTA030563	Pax7	0.001276516	0.486153616	down
ASMM9PARTA022057	Ccdc90a	0.0000444	0.384906655	down
ASMM9PARTA041030	Cpz	0.001890815	0.431117027	down
ASMM9PARTA040989	Acat3	0.000022	0.43168495	down
ASMM9PARTA029669	Lefty1	0.000108	0.480737497	down
CUST_292_PI426409190	Gm13298	0.0000235	0.481452407	down
ASMM9PARTA026243	Dnase1l1	0.006113933	0.492197004	down
ASMM9PARTA029989	Gbx2	0.00000594	0.287773618	down
ASMM9PARTA023550	Slc9a5	0.03002272	0.46258204	down
