# Supplementary material for: Microarray Profile of Long Noncoding RNA and Messenger RNA Expression in a Model of Alzheimer’s Disease
Source: Life (Basel). 2020 May 14;10(5):64. doi: 10.3390/life10050064 (PMC7281340; doi:10.3390/life10050064)
Supplement: Supplementary file 1 [file life-10-00064-s001.zip › life-787240-supplemenatry-to be published - PDF/life-787240-supplementary/Table S7.pdf]

life

Supplementary

Microarray Profile of Long Noncoding RNA and Messenger RNA Expression in a Model of Alzheimer's Disease

Linlin Wang †, Li Zeng †, Hailun Jiang, Zhuorong Li * and Rui Liu *

Institute of Medicinal Biotechnology, Chinese Academy of Medical Sciences and Peking Union Medical College, Beijing 100050, China; wanglinlin@wfmc.edu.cn (L.W.); zengsheng@imb.pumc.edu.cn (L.Z.); jianghailun@imb.pumc.edu.cn (H.J.)

* Correspondence: lizhourong@imb.pumc.edu.cn (Z.L.); +86-10-8352017; .liurui@imb.pumc.edu.cn (R.L.); Tel.: +86-10-67087731

Table S7. Differently expressed mRNAs in the brain of 6-month-old APP/PS1 mice compared with age-matched WT mice.

Probe Name	Symbol	*p*-Value	Fold Change	Regulation
ASMM9PARTA027293	Spp1	0.0000451	2.1507535	up
ASMM9PARTA042739	Ghsr	0.0000934	2.5166237	up
ASMM9PARTA025747	Tshb	0.002031273	2.2207026	up
ASMM9PARTA031791	Cfd	0.033389803	22.0273	up
ASMM9PARTA023112	Gm514	0.04049753	2.0624256	up
ASMM9PARTA033120	Cbln3	0.000000297	28.075712	up
ASMM9PARTA044292	Zhx2	0.000745	5.022098	up
ASMM9PARTA039095	Dbh	0.0000021	16.80503	up
ASMM9PARTA030304	Mycs	0.025340643	3.0412478	up
ASMM9PARTA030370	Myl3	0.022885038	15.371072	up
ASMM9PARTA037203	Arhgap24	0.001944784	2.2457936	up
ASMM9PARTA033464	Nrip2	0.0000124	2.1364672	up
ASMM9PARTA026780	Tirap	0.036988758	2.412029	up
ASMM9PARTA020996	Gm5615	0.00276148	2.299788	up
ASMM9PARTA033070	Mknk2	0.008370707	3.2609756	up
ASMM9PARTA026869	Dnmt1	0.001004816	3.3003516	up
ASMM9PARTA044461	Rsl24d1	0.0000209	3.105103	up
ASMM9PARTA023331	Arhgap17	0.027770417	2.0804007	up
ASMM9PARTA019947	Gjb6	0.000141	2.1275125	up
ASMM9PARTA021472	Serpinb6e	0.011722688	2.882823	up
ASMM9PARTA034385	Smpx	0.016476726	9.117216	up
ASMM9PARTA042177	Zfp934	0.000556	2.2483299	up
ASMM9PARTA022334	Kbtbd10	0.018513989	3.2591946	up
ASMM9PARTA022668	Gm5634	0.000856	3.7699485	up
ASMM9PARTA021305	G630016D24Rik	0.020363238	2.336865	up
ASMM9PARTA028762	Pipox	0.016579866	2.05768	up
ASMM9PARTA032059	Bag3	0.000000632	2.1400392	up
ASMM9PARTA034560	Kctd20	0.04951716	3.5796947	up
ASMM9PARTA025718	Cdr1	0.0000362	3.79277	up
ASMM9PARTA036648	Pear1	0.032610297	2.8006957	up

ASMM9PARTA027615	Cdh15	0.010074805	3.2024999	up
ASMM9PARTA025697	5-Mar	0.03398695	2.4036894	up
ASMM9PARTA024198	Lpp	0.001036903	2.7760649	up
ASMM9PARTA034297	Baiap2l1	0.000347	13.128269	up
ASMM9PARTA027888	Hbb-b1	2.17E-08	2.8186767	up
ASMM9PARTA043897	Vps39	0.00000818	6.309617	up
ASMM9PARTA038774	Kctd9	0.00019	2.0035682	up
ASMM9PARTA030930	Aplnr	0.0000104	3.750766	up
ASMM9PARTA023161	Vmn2r79	0.021861287	2.1963494	up
ASMM9PARTA029828	Cdh5	0.013344823	2.5924551	up
ASMM9PARTA044055	Abhd16b	0.01355558	2.592133	up
ASMM9PARTA024307	Hsd3b3	0.015399736	2.4931066	up
ASMM9PARTA033640	Stk32b	0.004813986	2.3867688	up
ASMM9PARTA041340	Phc3	0.008379956	3.2680974	up
ASMM9PARTA032924	Apbh	0.00733979	2.0741892	up
ASMM9PARTA034865	Ssr1	0.002536808	2.176073	up
ASMM9PARTA026193	Tmem11	0.011889421	2.635801	up
ASMM9PARTA029560	Cnn1	0.005160857	2.4631698	up
ASMM9PARTA036950	Eaf1	0.048665036	2.7194755	up
ASMM9PARTA022309	Tsc22d2	0.012616459	3.9405763	up
ASMM9PARTA028059	Gstm6	0.0000459	2.2506855	up
ASMM9PARTA021642	Hsf5	0.043553278	2.1462085	up
ASMM9PARTA028818	Th	0.00000893	2.2970579	up
ASMM9PARTA022070	Olfr455	0.006079886	2.072328	up
ASMM9PARTA041981	Phtf2	0.001884467	2.289164	up
ASMM9PARTA020187	Whrn	0.002399045	2.3698502	up
ASMM9PARTA043696	Zfp869	0.000164	2.5112703	up
ASMM9PARTA024120	Bcl2l15	0.00000181	6.9800644	up
ASMM9PARTA044674	Casc4	0.0000467	2.5319176	up
ASMM9PARTA021097	Trpm3	0.000691	5.5299506	up
ASMM9PARTA025695	R3hdm2	0.00489909	2.326392	up
ASMM9PARTA023315	Gatad2a	0.009272239	2.7316368	up
ASMM9PARTA031792	Ar	0.028068736	2.9195232	up
ASMM9PARTA042087	Fry	0.000238	2.2708285	up
ASMM9PARTA039655	Cald1	0.005097217	2.0567584	up
ASMM9PARTA039607	Oas1e	0.000819	3.174069	up
ASMM9PARTA043179	Fam163a	0.000347	3.2224464	up
ASMM9PARTA041975	Slc4a9	0.000625	2.2972817	up
ASMM9PARTA019635	Whamm	0.0000878	2.9223146	up
ASMM9PARTA029086	Ret	0.000284	2.0627956	up
ASMM9PARTA028813	Serpina3g	0.00000112	2.6244316	up
ASMM9PARTA024171	Homer3	0.000016	3.038775	up
ASMM9PARTA034391	1700011A15Rik	0.049442526	2.0497057	up
ASMM9PARTA042058	Mon1b	0.002280486	2.9871006	up
ASMM9PARTA031518	Spnb1	0.000582	5.295755	up
ASMM9PARTA020586	Defb46	0.010696249	2.0395687	up
ASMM9PARTA025868	Arhgap39	0.000537	2.4710615	up
ASMM9PARTA040424	Olfr197	0.0233421	2.075901	up
ASMM9PARTA044228	Ece1	0.004784548	2.6239338	up
ASMM9PARTA030907	Rgs16	0.0000114	4.361314	up
ASMM9PARTA021052	Tmem211	0.03917742	2.1583846	up
ASMM9PARTA029461	Vmn2r42	0.015054045	2.8441594	up
ASMM9PARTA037016	Tc2n	0.006119962	2.2615747	up
ASMM9PARTA035876	Pmvk	0.015881788	2.3479319	up
ASMM9PARTA030121	Eomes	0.0000614	3.354912	up
ASMM9PARTA025469	Amigo2	0.000802	2.2212565	up
ASMM9PARTA028727	Psen1	0.018458707	2.4210668	up

ASMM9PARTA043919	A430033K04Rik	0.00000451	3.1883342	up
ASMM9PARTA027704	Grid2	0.000857	2.8446321	up
ASMM9PARTA024666	Cntn1	0.000497	2.1110127	up
ASMM9PARTA027419	Agt	0.0000171	2.123765	up
ASMM9PARTA038866	1110008J03Rik	0.001368486	2.324984	up
ASMM9PARTA028143	Kera	0.021689761	2.130889	up
ASMM9PARTA029077	Vamp1	0.0000275	3.1666138	up
ASMM9PARTA027054	Spp1	0.00000948	2.4645803	up
ASMM9PARTA019955	Lrrc52	0.002044983	3.0441134	up
ASMM9PARTA020417	Gm5591	0.001059869	2.8320599	up
ASMM9PARTA027610	Capn5	0.016212257	2.0247874	up
ASMM9PARTA030353	Klk1b1	0.0000054	2.7851822	up
ASMM9PARTA021767	Rorb	0.015804261	2.4403055	up
ASMM9PARTA028084	Gabrr2	0.008975505	2.8294191	up
ASMM9PARTA032069	Gpc3	0.000525	5.630015	up
ASMM9PARTA030219	Hoxc13	0.006774094	2.6225698	up
ASMM9PARTA019763	Nlrp14	0.025239782	2.941392	up
ASMM9PARTA022005	Flnc	0.02564893	3.4128568	up
ASMM9PARTA043948	6330503K22Rik	0.009590808	2.168929	up
ASMM9PARTA031864	Ltbp2	0.005506281	2.084135	up
ASMM9PARTA034100	Pcbd1	0.000897	2.2127445	up
ASMM9PARTA027062	Dcun1d4	0.000164	2.0541759	up
ASMM9PARTA027366	Atoh1	0.025531106	2.0214107	up
ASMM9PARTA033437	Il21	0.017870067	3.065876	up
ASMM9PARTA038785	Krt25	0.00000787	5.745	up
ASMM9PARTA033390	Rqcd1	0.003801986	5.380088	up
ASMM9PARTA025231	Tnnt3	0.002261184	92.10396	up
ASMM9PARTA029574	Apob	0.00023	2.2258976	up
ASMM9PARTA033704	Sync	0.005422793	4.0052185	up
ASMM9PARTA039024	Atp12a	0.03470405	3.4817407	up
ASMM9PARTA031529	Zfp59	0.03831559	2.48568	up
ASMM9PARTA029209	Prss2	0.048823357	3.38558	up
ASMM9PARTA040166	Olfr122	0.001975795	3.254862	up
ASMM9PARTA037334	Mccc2	0.0000368	3.304477	up
ASMM9PARTA040757	Olfr1214	0.007098079	3.1042252	up
ASMM9PARTA043067	Ano5	0.031087117	2.5282829	up
ASMM9PARTA029895	Hspa1b	0.00000369	4.155569	up
ASMM9PARTA028754	P2ry1	0.0000979	2.2626753	up
ASMM9PARTA029778	Slc6a3	0.00000547	2.981036	up
ASMM9PARTA038671	D17H6S56E-3	0.001586099	2.706511	up
ASMM9PARTA039871	Ppp4r1	0.006232996	3.2765002	up
ASMM9PARTA040991	Clrn1	0.006462216	2.375496	up
ASMM9PARTA041748	Itih5	0.015554895	2.0738025	up
ASMM9PARTA033142	G6pc2	0.045349248	2.1340294	up
ASMM9PARTA038384	Vmn1r53	0.000728	2.467902	up
ASMM9PARTA037367	Trim42	0.022432802	2.4971278	up
ASMM9PARTA024887	Ccdc90b	0.00047	10.313978	up
ASMM9PARTA029709	Fut4	0.0000312	2.0945861	up
ASMM9PARTA021373	Akap2	0.0000253	2.9824905	up
ASMM9PARTA025157	Cttnbp2nl	0.027286498	2.3385003	up
ASMM9PARTA043478	Mapkapk3	0.026506413	2.2498312	up
ASMM9PARTA043280	Slc15a5	0.046162724	4.608725	up
ASMM9PARTA033834	2010106G01Rik	0.000591	4.5927052	up
ASMM9PARTA043838	Inpp5a	0.0000157	2.3635688	up
ASMM9PARTA033715	Foxb1	0.000159	2.247318	up
ASMM9PARTA038903	Lbr	0.033360366	2.5546308	up
ASMM9PARTA021491	Edem3	0.035057377	2.1087804	up

ASMM9PARTA041614	Itpk1	0.000689	2.6587727	up
ASMM9PARTA040339	Olfr196	0.000354	2.888675	up
ASMM9PARTA032481	Cbln1	6.21E-08	7.6642175	up
ASMM9PARTA043545	Zfp750	0.00000792	3.6266682	up
ASMM9PARTA023587	Schip1	0.007595373	2.278519	up
ASMM9PARTA025288	Rtdr1	0.001911789	2.317909	up
ASMM9PARTA039817	Ankrd40	0.0000125	3.1449397	up
ASMM9PARTA023477	Slc14a2	0.032083437	2.1979492	up
ASMM9PARTA035767	Zfp715	0.001757891	2.461495	up
ASMM9PARTA043312	Apol10b	0.01654708	3.3964062	up
ASMM9PARTA042964	Ep300	0.017471774	2.0880883	up
ASMM9PARTA037995	Clec2h	0.001158288	3.1110222	up
ASMM9PARTA024254	C530028O21Rik	0.000695	2.6598995	up
ASMM9PARTA023701	Pcp2	0.00000446	24.346376	up
ASMM9PARTA037527	1700110M21Rik	0.0000836	2.4209843	up
ASMM9PARTA042763	Ankar	0.0000426	2.1058571	up
ASMM9PARTA040966	Cxcl17	0.008595878	3.92512	up
ASMM9PARTA036365	Zfp618	0.002117588	3.4052744	up
ASMM9PARTA035190	Art4	0.012851267	2.3664777	up
ASMM9PARTA037939	Prl2c2	0.001795423	3.205593	up
ASMM9PARTA030102	Heph	0.008351945	2.4032056	up
ASMM9PARTA038954	H1foo	1.58E-08	3.816673	up
ASMM9PARTA041564	Spata2	0.015378239	2.4230084	up
ASMM9PARTA026523	Gm4340	0.03074876	2.1404755	up
ASMM9PARTA028498	Il13ra2	0.000131	2.8751483	up
ASMM9PARTA026945	Dpp6	0.001361765	2.268861	up
ASMM9PARTA024850	A630055G03Rik	0.0155094	2.0040278	up
ASMM9PARTA037407	2310079F23Rik	0.000922	2.5119534	up
ASMM9PARTA041027	Fcrl1	0.03209504	2.072643	up
ASMM9PARTA028440	Mybl1	0.015376825	2.0492637	up
ASMM9PARTA030885	Tagln	0.000107	2.238162	up
ASMM9PARTA030400	Mab21l1	0.0000142	4.5367174	up
ASMM9PARTA025147	1110017F19Rik	0.04037341	2.7562046	up
ASMM9PARTA038572	Tspan1	0.016547572	2.2429266	up
ASMM9PARTA042451	Gimap9	0.02050271	3.6060855	up
ASMM9PARTA042579	Tmem169	0.002666349	2.661957	up
ASMM9PARTA021047	Lrrc30	0.019311016	101.44545	up
ASMM9PARTA037599	Trdn	0.01988945	9.928261	up
ASMM9PARTA030271	Tsc22d3	0.000208	2.513357	up
ASMM9PARTA043176	Klhl23	0.036333885	2.1211534	up
ASMM9PARTA024222	Cntfr	0.000000248	20.435862	up
ASMM9PARTA043586	Ldlrad3	0.04709643	2.1698556	up
ASMM9PARTA029305	Emp1	0.026493885	2.3396127	up
ASMM9PARTA021443	Dach1	0.004493969	2.305273	up
ASMM9PARTA028099	Lrig1	0.0000134	2.8132274	up
ASMM9PARTA021180	Ceacam1	0.009496469	2.860777	up
ASMM9PARTA027678	Bmp5	0.026984278	2.2557604	up
ASMM9PARTA034817	4921517D21Rik	0.005037657	3.212635	up
ASMM9PARTA033917	Aox3	0.002923544	2.3182437	up
ASMM9PARTA019807	Pabpn1l	0.005487891	2.8395882	up
ASMM9PARTA041849	Fam70a	0.0000955	2.0372658	up
ASMM9PARTA025907	Phc3	0.04853418	3.0800793	up
ASMM9PARTA023833	Tspan32	0.029588483	2.9447546	up
ASMM9PARTA043857	Uts2d	0.000366	4.222785	up
ASMM9PARTA028903	Phox2a	0.000256	4.831777	up
ASMM9PARTA042512	9230105E10Rik	0.000692	2.464564	up
ASMM9PARTA033690	Ddr2	0.000069	2.4158735	up

ASMM9PARTA030005	Hipk1	0.00000171	2.8245034	up
ASMM9PARTA022678	Prrt4	0.006915411	2.8614578	up
ASMM9PARTA022707	Krtap9-5	0.00031	3.3291621	up
ASMM9PARTA030244	Gsr	0.0000322	6.854373	up
ASMM9PARTA043610	Nlrx1	0.010251218	2.880651	up
ASMM9PARTA031062	Tnnt1	0.00000211	2.1378279	up
ASMM9PARTA043415	Prpf39	0.00000481	3.5499609	up
ASMM9PARTA044030	Igsf1	0.0000524	2.6186712	up
ASMM9PARTA029529	Dnase1	0.001909962	2.8052783	up
ASMM9PARTA040116	Olfr694	0.021078102	3.3556395	up
ASMM9PARTA020086	Whrn	0.023081334	2.1139889	up
ASMM9PARTA024537	Cd36	0.00013	2.2969499	up
ASMM9PARTA022826	Slc2a9	0.02643714	2.0945556	up
ASMM9PARTA029985	Fmn1	0.002995136	2.1423795	up
CUST_241_PI426409190	Ssty2	0.000606	2.6810532	up
ASMM9PARTA042538	Grik4	0.006821831	2.837808	up
ASMM9PARTA032388	Hbb-b2	0.0000234	2.3997326	up
ASMM9PARTA031278	Mab21l2	0.029376728	3.4686258	up
ASMM9PARTA020624	Gal3st3	0.006092818	2.3928666	up
ASMM9PARTA021175	Ldb3	0.002290151	2.0798974	up
ASMM9PARTA041188	Dio3	0.002794048	2.2760143	up
ASMM9PARTA029354	Vipr2	0.013459918	3.065879	up
ASMM9PARTA036776	Tmc1	0.001881636	5.8655567	up
ASMM9PARTA019864	Olfr279	0.000771	2.7403243	up
ASMM9PARTA022177	Lhfpl3	0.0000118	2.015227	up
ASMM9PARTA021401	Sco1	0.028912717	2.1166487	up
ASMM9PARTA020982	Gm136	0.002576352	2.1638193	up
ASMM9PARTA026309	Rbms3	0.03979875	2.1052928	up
ASMM9PARTA036032	Pdgfd	0.02692101	3.0033252	up
ASMM9PARTA041163	Ccdc137	0.014191769	2.763659	up
ASMM9PARTA041104	Nat10	0.0401323	3.242169	up
ASMM9PARTA038580	Dst	0.045145154	2.7075315	up
ASMM9PARTA021775	Spna2	0.000147	2.0539868	up
ASMM9PARTA031584	Hoxd9	0.006060285	8.735599	up
ASMM9PARTA030062	Hspa1a	0.0000067	5.140137	up
ASMM9PARTA021070	Lipt1	0.024270058	2.5310833	up
ASMM9PARTA041954	Rln3	0.0000116	16.755537	up
ASMM9PARTA033694	Nup98	0.001093553	2.0166821	up
ASMM9PARTA031964	Tmod4	0.008487557	5.95723	up
ASMM9PARTA034058	Osbpl5	0.039886918	2.164772	up
ASMM9PARTA033067	Nova1	0.00000572	2.9075933	up
ASMM9PARTA027370	Calb2	0.000132	2.6458483	up
ASMM9PARTA030586	Per2	0.000386	2.510641	up
ASMM9PARTA024773	Myef2	0.00000144	3.3670971	up
ASMM9PARTA039294	Chrna3	0.00000842	2.247737	up
ASMM9PARTA037392	Flywch2	0.002874707	2.0012379	up
ASMM9PARTA037145	Gsc2	0.002820018	2.5668445	up
ASMM9PARTA036517	Vgll3	0.000586	3.5175169	up
ASMM9PARTA031919	Atp2a3	0.009953384	2.2323298	up
ASMM9PARTA021470	Ifi203	0.009155741	2.3724337	up
ASMM9PARTA042749	Actbl2	0.017515803	2.4555907	up
ASMM9PARTA031110	Ywhaz	0.00000757	2.839082	up
ASMM9PARTA026158	Gtf2e2	0.00518237	3.1096683	up
ASMM9PARTA020210	2700007P21Rik	0.00015	13.020236	up
ASMM9PARTA033314	Jmy	0.007809873	2.534826	up
ASMM9PARTA030680	Neurod1	0.00000489	3.9375358	up
ASMM9PARTA040122	Cacnb4	0.000000403	2.448683	up

ASMM9PARTA044544	Mrgprb4	0.0000313	2.1738114	up
ASMM9PARTA022634	Dusp5	0.0000134	2.0263927	up
ASMM9PARTA030432	Lmx1b	0.007112836	2.2152882	up
ASMM9PARTA022535	Hba-a2	0.000392	2.4255915	up
ASMM9PARTA021995	Cep76	0.000218	2.3323243	up
ASMM9PARTA041320	Acsf2	0.0000591	2.1580765	up
ASMM9PARTA036484	Pi4k2b	0.007517041	2.150202	up
ASMM9PARTA039203	Cys1	0.000255	2.5070796	up
ASMM9PARTA025140	Rrp1b	0.004429032	2.774386	up
ASMM9PARTA037729	Apobec1	0.004225649	4.623898	up
ASMM9PARTA037840	Akap12	0.00000167	6.0745997	up
CUST_243_PI426409190	B020031M17Rik	0.001004077	3.1337562	up
ASMM9PARTA036824	Arhgap8	0.004597947	2.661679	up
ASMM9PARTA027106	Me1	0.045341842	2.7356696	up
ASMM9PARTA020953	Ralgapa2	0.02107708	2.4623852	up
ASMM9PARTA019915	Zfyve26	0.030769322	6.8301525	up
ASMM9PARTA019599	Zfp619	0.013915096	2.6102865	up
ASMM9PARTA029961	Itih2	0.00774049	2.746205	up
ASMM9PARTA035548	2610034M16Rik	0.029662553	2.0447943	up
ASMM9PARTA025965	Galnt7	0.0000225	2.3616095	up
ASMM9PARTA036961	1700001C19Rik	0.000939	2.3691561	up
ASMM9PARTA038935	Ehd4	0.000582	4.431277	up
ASMM9PARTA042571	Ankrd34b	0.002932512	3.3556213	up
ASMM9PARTA037836	F13b	0.012693805	2.3770068	up
ASMM9PARTA021982	Gpr155	0.012926245	3.0703354	up
ASMM9PARTA042755	Cnpy1	0.00000572	9.815484	up
ASMM9PARTA019700	Amica1	0.01967706	3.7898314	up
ASMM9PARTA031132	Cd70	0.000000368	10.944201	up
ASMM9PARTA044412	Olfr645	0.000515	3.6101472	up
ASMM9PARTA028102	Kcnc3	0.000459	2.5806677	up
ASMM9PARTA038930	Vmn1r231	0.02228777	2.5191486	up
ASMM9PARTA024346	Zkscan3	0.03204355	3.2180145	up
ASMM9PARTA026910	Nkx3-2	0.006124512	2.7580554	up
ASMM9PARTA027355	Armcx4	0.03317694	2.5112898	up
ASMM9PARTA024808	Ttc15	1.66E-08	103.32905	up
ASMM9PARTA022470	Acin1	0.000398	2.6483488	up
ASMM9PARTA019728	A430105I19Rik	0.0000744	2.4022124	up
ASMM9PARTA036836	Psd2	0.003252846	3.6852026	up
ASMM9PARTA025234	Psg18	0.007264738	2.285354	up
ASMM9PARTA029776	Dao	0.000003	6.381328	up
ASMM9PARTA039500	Hnrnpul1	0.002161555	2.5496287	up
ASMM9PARTA040206	Olfr1305	0.014845275	2.002494	up
ASMM9PARTA020089	Ppp1r13l	0.000722	2.2169778	up
ASMM9PARTA021040	Trpm3	0.001506105	15.397922	up
ASMM9PARTA029136	Svs5	0.001207155	3.3846748	up
ASMM9PARTA020866	Camk2d	0.0000433	2.1424425	up
ASMM9PARTA042786	Mrgprb2	0.001318275	3.5893843	up
ASMM9PARTA039742	Hmx2	0.011951079	2.2070355	up
ASMM9PARTA044237	Tspan15	0.0000517	3.375963	up
ASMM9PARTA037515	Kcnip4	0.000041	3.956419	up
ASMM9PARTA040624	Olfr907	0.00016	2.785742	up
ASMM9PARTA037352	Bicd2	0.000739	3.8382185	up
ASMM9PARTA037850	Kel	0.011615739	2.8589933	up
ASMM9PARTA020376	Gm5622	0.000459	2.199031	up
ASMM9PARTA024400	Pex19	0.046047475	2.1324735	up
ASMM9PARTA037824	1700061G19Rik	0.027703602	2.002491	up
ASMM9PARTA042805	Casc4	0.000722	2.038495	up

ASMM9PARTA031043	Wdr1	0.00000441	2.1056302	up
ASMM9PARTA032065	Dlc1	0.0000614	3.96439	up
ASMM9PARTA034262	Srsf9	0.000102	2.5463078	up
ASMM9PARTA030830	Pcsk6	0.003532501	2.5390463	up
ASMM9PARTA027033	Kcnmb3	0.005199567	3.2952516	up
ASMM9PARTA041105	Mmrn2	0.000667	2.00408	up
ASMM9PARTA039089	Triobp	0.038257845	2.5824273	up
ASMM9PARTA044550	Rasgrp3	0.017140893	2.763976	up
ASMM9PARTA031087	Serpinf1	0.000174	2.261003	up
ASMM9PARTA025317	Mpp6	0.0000592	2.136795	up
ASMM9PARTA026812	Plch1	0.007054912	2.9347374	up
ASMM9PARTA042748	Zfp367	0.000671	2.114461	up
ASMM9PARTA026781	Asph	0.000643	2.092841	up
ASMM9PARTA042834	Sp8	0.049545813	2.1542397	up
ASMM9PARTA034340	Plekhf1	0.000006	3.0567439	up
ASMM9PARTA025390	Chn2	0.00000017	3.674339	up
ASMM9PARTA020325	Olfr288	0.000044	6.0403857	up
ASMM9PARTA044568	B4galnt3	0.00214913	2.0200653	up
ASMM9PARTA039455	Galnt7	0.00000339	5.971164	up
ASMM9PARTA035313	Fam134c	0.0000552	2.0044026	up
ASMM9PARTA028888	Slc1a6	0.00000017	4.558792	up
ASMM9PARTA028251	Mfap2	0.021973915	2.2584455	up
ASMM9PARTA029080	Zic1	2.75E-08	2.7256691	up
ASMM9PARTA027863	Cntn1	0.006698464	2.056628	up
ASMM9PARTA032766	Slc5a1	0.000437	2.324745	up
ASMM9PARTA034780	Lipf	0.010790499	2.430978	up
ASMM9PARTA036813	1700011I03Rik	0.027928095	2.7580304	up
ASMM9PARTA020744	Ccdc141	0.000279	6.163243	up
ASMM9PARTA042368	Ccdc88a	0.00791295	3.2940211	up
ASMM9PARTA043130	Exoc3l	0.00936924	3.0666118	up
ASMM9PARTA039338	Tmprss11d	0.001684542	4.3812766	up
ASMM9PARTA029319	Wnt3	0.000000967	2.064447	up
ASMM9PARTA022542	Atad2b	0.003796604	2.3051326	up
ASMM9PARTA020793	Dusp27	0.021160334	7.7792406	up
ASMM9PARTA038129	Klhl1	0.000171	2.3436751	up
ASMM9PARTA033406	Sap30	0.0000341	2.1461208	up
ASMM9PARTA040840	Olfr1047	0.010189298	3.2805078	up
ASMM9PARTA025731	Gm2799	0.03302557	6.0889807	up
ASMM9PARTA028000	Hba-a1	0.000000371	2.2257965	up
ASMM9PARTA042773	Itgb8	0.004099923	2.4200108	up
ASMM9PARTA025232	Tnnt3	0.00263361	42.93324	up
ASMM9PARTA024050	Sgca	0.046804093	7.6466875	up
ASMM9PARTA021003	Trpm3	0.000075	9.5873165	up
ASMM9PARTA031395	Slc27a2	0.00000132	2.1188936	up
ASMM9PARTA043498	Reps2	0.002706006	2.2106931	up
ASMM9PARTA020326	Olfr1386	0.028507153	2.1641836	up
ASMM9PARTA031095	Sox14	0.0000881	2.1730487	up
ASMM9PARTA029686	Pdia4	0.000297	2.0785758	up
ASMM9PARTA043171	Tmem69	0.004710449	3.7459686	up
ASMM9PARTA028835	Ppl	0.002112785	2.651658	up
ASMM9PARTA027815	Fbn1	0.0000735	2.8485188	up
ASMM9PARTA035509	2310057N15Rik	0.002414743	9.600296	up
ASMM9PARTA037605	Efcab6	0.029474366	2.6773224	up
ASMM9PARTA040822	Olfr902	0.003848341	3.0751271	up
ASMM9PARTA024271	Pax3	0.003083293	3.184304	up
ASMM9PARTA042808	Gchfr	0.0000145	2.2406266	up
ASMM9PARTA032063	Sufu	0.0074365	2.2831154	up

ASMM9PARTA034146	Krtap3-3	0.026812445	2.436852	up
ASMM9PARTA043245	Gm14461	0.015765708	2.2798564	up
ASMM9PARTA020936	Calca	0.00000375	3.413624	up
ASMM9PARTA034762	Ceacam14	0.000837	2.1184804	up
ASMM9PARTA020263	Olfr1211	0.023600569	3.029151	up
ASMM9PARTA031037	Try4	0.006781471	2.6001742	up
ASMM9PARTA026515	Shisa9	0.001662943	2.5787144	up
ASMM9PARTA039612	Duoxa1	0.005727533	2.359136	up
ASMM9PARTA024549	Smek1	0.004446384	2.2665513	up
ASMM9PARTA020552	Serinc4	0.03796334	2.4404204	up
ASMM9PARTA020828	Cpxcr1	0.00123529	2.0561805	up
ASMM9PARTA041953	Serpinb1c	0.002610939	3.0740879	up
ASMM9PARTA025627	Vmn1r168	0.0000289	2.517856	up
ASMM9PARTA029772	Cox7a1	0.003111761	2.4177475	up
ASMM9PARTA024760	Atp2a3	0.00000317	5.0496964	up
ASMM9PARTA026365	Arpc4	0.0000912	2.3872566	up
ASMM9PARTA022337	Arhgap36	0.004429061	2.2168605	up
ASMM9PARTA029144	Tnni2	0.012855267	57.733353	up
ASMM9PARTA042277	St18	0.000272	2.485741	up
ASMM9PARTA025384	Puf60	0.012556245	2.1604857	up
ASMM9PARTA032045	Uncx	0.00000569	4.4167857	up
ASMM9PARTA028786	Tnnc2	0.004108564	36.2881	up
ASMM9PARTA043974	Opn5	0.0000622	2.4719467	up
ASMM9PARTA044790	Vmn1r177	0.001943354	4.166602	up
ASMM9PARTA041502	Snx30	0.000566	2.3903387	up
ASMM9PARTA034030	Ankrd22	0.002593705	2.305333	up
ASMM9PARTA023018	Gabra6	0.00000044	68.853714	up
ASMM9PARTA030388	Igfbp5	0.046108607	2.194429	up
ASMM9PARTA032853	Fam48a	0.014244651	2.0026085	up
ASMM9PARTA029959	Irs4	0.013977591	2.212249	up
ASMM9PARTA019811	Mxi1	0.001022708	2.040559	up
ASMM9PARTA031308	Clec3b	0.001239764	2.016329	up
ASMM9PARTA028683	Pcp2	0.000198	3.2875087	up
ASMM9PARTA031384	Zfp9	0.01831999	2.0299964	up
ASMM9PARTA041081	Olfr711	0.031449612	2.7025623	up
ASMM9PARTA042591	Cyb5r2	0.004445947	2.1296048	up
ASMM9PARTA026501	Magi2	0.02515099	2.2347913	up
ASMM9PARTA035464	Srrm4	0.004873351	2.1525927	up
ASMM9PARTA030405	Myoc	1.78E-08	2.0539494	up
ASMM9PARTA027336	Cav3	0.034468632	11.514316	up
ASMM9PARTA034118	1700020D05Rik	0.000278	2.1767392	up
ASMM9PARTA020564	Yeats2	0.01473221	2.754269	up
ASMM9PARTA030500	Klk1b11	0.00000108	2.7794347	up
ASMM9PARTA037389	Calr3	0.00202343	2.6592324	up
ASMM9PARTA022994	Gm6251	0.000407	5.2407193	up
ASMM9PARTA029402	Apobec2	0.014304318	8.075904	up
ASMM9PARTA038106	Pth2	0.0000485	3.4290369	up
ASMM9PARTA028920	S100b	0.000000832	2.0906541	up
ASMM9PARTA023496	Stxbp5l	0.000317	2.0372517	up
ASMM9PARTA037984	E2f6	0.0000789	2.73142	up
ASMM9PARTA020160	Olfr1229	0.019650666	2.1758113	up
ASMM9PARTA041048	Olfr1390	0.022678364	2.140308	up
ASMM9PARTA026421	Plch1	0.020583244	2.1288514	up
ASMM9PARTA027734	Ebf1	0.004148378	3.2014337	up
ASMM9PARTA021187	Rrn3	0.000136	3.4482841	up
ASMM9PARTA044488	Ostn	0.000935	5.9937854	up
ASMM9PARTA039435	Mrgprf	0.000179	3.5290005	up

ASMM9PARTA032757	Cxcl11	0.018971197	3.7834735	up
ASMM9PARTA024516	Sgk1	0.00000765	2.107928	up
ASMM9PARTA030282	Hp1bp3	0.000578	3.1041622	up
ASMM9PARTA033144	Accn5	0.000304	4.12643	up
ASMM9PARTA027834	Csf2rb2	0.04952057	2.3011076	up
ASMM9PARTA026178	Rbms3	0.000576	2.7565129	up
ASMM9PARTA020962	Akap2	0.00000683	2.7353158	up
CUST_307_PI426409190	Amy2a5	0.0000228	2.060406	up
ASMM9PARTA030450	Gsbs	0.00000044	5.4431267	up
ASMM9PARTA041214	Adam1b	0.008788805	2.3065994	up
ASMM9PARTA022749	Pnmal2	0.014129776	2.0180314	up
ASMM9PARTA025585	Slc38a1	0.001739261	2.4397264	up
ASMM9PARTA031638	Uts2	0.000124	8.977784	up
ASMM9PARTA022137	Mtap7d2	0.00000829	2.4873993	up
ASMM9PARTA030350	Foxa2	0.0000796	3.0492747	up
ASMM9PARTA023933	Opn4	0.000193	2.4023864	up
ASMM9PARTA026822	Elavl2	0.000835	2.171586	up
ASMM9PARTA023233	Skor2	0.000503	5.272236	up
ASMM9PARTA033226	Sdf2l1	0.000000119	2.9944234	up
ASMM9PARTA027981	Evx1	6.88E-08	3.2314925	up
ASMM9PARTA022335	Cep152	0.041447245	2.2245932	up
ASMM9PARTA028696	Resp18	0.000000906	2.283674	up
ASMM9PARTA035707	4921528I01Rik	0.023241224	2.213509	up
ASMM9PARTA024658	Birc7	0.0000156	2.0662322	up
ASMM9PARTA027331	Btg1	0.03314706	2.0956974	up
ASMM9PARTA027827	Cd38	0.001392794	2.2292066	up
ASMM9PARTA024004	Dedd	0.010933708	2.241811	up
ASMM9PARTA026887	Pdlim5	0.004873829	2.0783958	up
ASMM9PARTA029430	Akap2	0.0000198	2.804051	up
ASMM9PARTA038153	Myoz3	0.003786849	12.108096	up
ASMM9PARTA033675	Dact1	0.03994828	2.0742466	up
ASMM9PARTA027750	Cbr2	0.000192	2.0349858	up
ASMM9PARTA024571	Pde1c	0.005904309	2.1231558	up
ASMM9PARTA031052	Sncg	0.00000623	2.9424489	up
ASMM9PARTA041481	Zswim4	0.000244	2.398425	up
ASMM9PARTA031863	Hspb1	0.00000259	2.7280755	up
ASMM9PARTA044533	Zfp933	0.00000182	6.0847216	up
ASMM9PARTA031244	Tcea3	0.03166103	2.7796075	up
ASMM9PARTA030984	Slc4a1	0.000308	2.2548468	up
ASMM9PARTA029751	Alox5	0.000573	3.5853097	up
ASMM9PARTA034897	Ttc23	0.035309765	2.9228115	up
ASMM9PARTA027912	Dcn	0.004250892	4.1296043	up
ASMM9PARTA033313	Abhd1	0.000226	2.9045417	up
ASMM9PARTA029678	Ahrr	0.033838246	2.2988186	up
ASMM9PARTA042372	6430573F11Rik	0.0000429	2.9362195	up
ASMM9PARTA028062	Hsd17b2	0.000178	2.4844553	up
ASMM9PARTA021755	Ret	0.004257571	2.1840377	up
ASMM9PARTA037719	Armcx1	0.000058	2.0988758	up
ASMM9PARTA019859	Zfp456	0.006440431	2.3468914	up
ASMM9PARTA027681	Ckm	0.041310553	63.597164	up
ASMM9PARTA031831	Pvalb	0.00000581	2.5284045	up
ASMM9PARTA022286	Agmat	0.0000157	2.365273	up
ASMM9PARTA033219	Fign	0.000223	2.0633774	up
ASMM9PARTA035573	Fitm1	0.020627724	15.344873	up
ASMM9PARTA038028	Pdxdc1	0.00000421	2.9084237	up
ASMM9PARTA032586	Hebp2	0.003912168	2.2569003	up
ASMM9PARTA030995	Sdc4	0.0000285	2.1772516	up

ASMM9PARTA039169	Onecut3	0.000958	2.4656615	up
ASMM9PARTA026574	Gm6588	0.00493508	2.4860842	up
ASMM9PARTA033369	Trp53inp1	0.025053442	2.1810691	up
ASMM9PARTA038488	Dpf3	0.00015	2.3491356	up
ASMM9PARTA027810	Eps8	0.000509	2.0840042	up
ASMM9PARTA032827	Pigp	0.002434658	2.1606565	up
ASMM9PARTA022770	4933407P14Rik	0.001538146	2.010531	up
ASMM9PARTA042594	Hnrnpl	0.00000534	3.3814728	up
ASMM9PARTA033117	Dpt	0.015191129	2.2584329	up
ASMM9PARTA030942	Prnp	0.00000509	2.3321254	up
ASMM9PARTA043858	Nhlh2	0.001418031	7.3683906	up
ASMM9PARTA042037	Aqp6	0.0000084	6.8246646	up
ASMM9PARTA026534	Cuedc1	0.00000967	2.1203527	up
ASMM9PARTA033259	Tlx1	0.0000148	2.7205691	up
ASMM9PARTA025291	Prph	0.00017	2.9519014	up
ASMM9PARTA023927	B3gnt5	0.03585292	2.1002202	up
ASMM9PARTA032039	Mylpf	0.019202126	19.031195	up
ASMM9PARTA025067	Tnnt3	0.024728868	20.21389	up
ASMM9PARTA029843	En2	0.000000626	4.271328	up
ASMM9PARTA020689	C1ql4	0.00000964	3.728463	up
ASMM9PARTA020162	Olfr287	0.0000117	4.7746563	up
ASMM9PARTA021792	Unc13c	0.000214	2.8390257	up
ASMM9PARTA027745	Gabra6	0.000163	71.481064	up
ASMM9PARTA038750	1110032A04Rik	0.0000627	2.1570268	up
ASMM9PARTA031536	Nr0b2	0.0000102	2.038954	up
ASMM9PARTA029133	Sparc	0.0000436	2.396882	up
ASMM9PARTA038588	Ccdc117	0.000376	2.16176	up
ASMM9PARTA024273	Heph	0.000839	2.975967	up
ASMM9PARTA031961	Hpcal1	0.0000166	2.2622392	up
ASMM9PARTA028676	Rbbp4	0.0000256	2.3407652	up
ASMM9PARTA027065	Kcnip1	0.0000282	4.726039	up
ASMM9PARTA022180	Dscaml1	0.026648734	2.155983	up
ASMM9PARTA032825	Nmu	0.006328255	2.2326236	up
ASMM9PARTA028274	Foxa1	6.42E-08	2.8207698	up
ASMM9PARTA024585	Ptprr	0.00406898	7.350789	up
ASMM9PARTA022268	4930420K17Rik	0.000375	2.006079	up
ASMM9PARTA044658	C1ql2	0.0000121	2.2450745	up
ASMM9PARTA044769	Tigd4	0.000112	5.815109	up
ASMM9PARTA024350	Lgals3	0.001434588	2.5295136	up
ASMM9PARTA028740	Scg5	0.0000977	2.3858893	up
ASMM9PARTA030689	Hspa4l	0.0000151	2.0125673	up
ASMM9PARTA039119	Otub1	0.000301	2.2946272	up
ASMM9PARTA027647	Cdh2	0.00000207	2.0419476	up
ASMM9PARTA022310	Mfhas1	0.0000667	2.2076235	up
ASMM9PARTA023780	Bcl2l15	0.00000555	6.2908607	up
ASMM9PARTA035366	1700029I01Rik	0.004849663	2.420302	up
ASMM9PARTA032822	Elovl2	0.005735204	2.4472451	up
ASMM9PARTA031902	Eif2ak4	0.00000387	3.9036727	up
ASMM9PARTA043954	Pla2g12a	0.00188179	2.3330312	up
ASMM9PARTA029960	Irx1	0.0000907	2.4034426	up
ASMM9PARTA036814	Ascc2	0.034301106	2.198609	up
ASMM9PARTA029329	Nr2f2	0.0000198	4.012704	up
ASMM9PARTA023424	Fcrl5	0.000212	3.8824918	up
ASMM9PARTA031116	Mmp23	0.000015	2.244075	up
ASMM9PARTA020844	Gpr101	0.0000695	2.1989505	up
ASMM9PARTA024036	Inpp5a	0.000493	2.1914525	up
ASMM9PARTA023497	Stxbp5l	0.0000586	2.9507787	up

ASMM9PARTA036324	Rpap3	0.000549	2.2583456	up
ASMM9PARTA043472	Luzp2	0.0000599	2.5671635	up
ASMM9PARTA023377	Kctd9	0.000752	2.0294223	up
ASMM9PARTA044231	Zfp748	0.0000707	2.4149058	up
ASMM9PARTA028311	Htr2c	0.003728641	2.1024995	up
ASMM9PARTA035109	Prorsd1	0.00000241	2.8383548	up
ASMM9PARTA031374	Aldh1a1	0.000068	2.2125616	up
ASMM9PARTA029965	Kcnk7	0.00088	2.6617348	up
ASMM9PARTA030926	Xdh	0.000617	2.58435	up
ASMM9PARTA024013	Megf11	0.0000309	2.2931662	up
ASMM9PARTA042250	Nek1	0.00098	2.1580672	up
ASMM9PARTA033962	Popdc3	0.00000205	2.853729	up
ASMM9PARTA029000	Tnfsf9	0.0000101	2.390563	up
ASMM9PARTA029038	Tll1	0.003162227	3.25447	up
ASMM9PARTA036347	Dhrs2	0.000478	2.3309717	up
ASMM9PARTA041756	4932438A13Rik	0.010479278	2.073594	up
ASMM9PARTA028318	Itih3	0.000169	2.378268	up
ASMM9PARTA021504	Prl2c1	0.023716394	2.8436213	up
ASMM9PARTA019912	Inadl	0.0000607	2.3539095	up
ASMM9PARTA021703	Zscan21	0.001603618	2.1092527	up
ASMM9PARTA043734	Prl2c5	0.00019	3.9286435	up
ASMM9PARTA034289	Fam107b	0.0000527	2.6628532	up
ASMM9PARTA036473	3110007F17Rik	0.000112	2.1604674	up
ASMM9PARTA020425	Ldoc1	0.0000218	2.3029172	up
ASMM9PARTA031598	Schip1	0.0000466	2.9829657	up
ASMM9PARTA026984	App	0.000228	2.0537932	up
ASMM9PARTA021618	Scoc	0.001918294	2.12381	up
ASMM9PARTA030969	Tnnt3	0.014237377	105.348335	up
ASMM9PARTA029543	Aqp4	0.000000065	2.2935846	up
ASMM9PARTA032569	Il22	0.000000781	24.978449	up
ASMM9PARTA031128	Tal1	0.0000101	2.477537	up
ASMM9PARTA025388	Prph	0.000312	2.0239348	up
ASMM9PARTA044577	Dand5	0.018277878	2.1043851	up
ASMM9PARTA041787	Zfp609	0.01374517	2.3819363	up
ASMM9PARTA024523	Celf4	0.000777	9.330751	up
ASMM9PARTA037989	Olig3	0.000465	2.427537	up
ASMM9PARTA021266	Il20rb	0.00000137	11.799635	up
ASMM9PARTA042557	Lingo4	0.000318	2.3799798	up
ASMM9PARTA038320	Timp4	0.0000368	2.0005925	up
ASMM9PARTA030189	Meis1	0.001373908	2.7708988	up
ASMM9PARTA025685	Ccdc85a	0.0000166	2.1329918	up
ASMM9PARTA041138	E430025E21Rik	0.00686088	2.171286	up
ASMM9PARTA036809	4930503E14Rik	0.000118	2.1801968	up
ASMM9PARTA033172	Myl1	0.022617685	4.9668155	up
ASMM9PARTA031682	Anxa4	0.000457	2.016557	up
ASMM9PARTA031049	Sgk1	0.000272	2.2176924	up
ASMM9PARTA030501	Klk1b26	0.003440333	3.764769	up
ASMM9PARTA026905	Beta-s	0.00000967	2.5352697	up
ASMM9PARTA020878	Pak6	0.000121	2.8478758	up
ASMM9PARTA037349	Hyls1	0.009235193	2.3268843	up
ASMM9PARTA022660	Cartpt	0.000106	2.1583462	up
ASMM9PARTA021034	Shisa6	0.000236	2.0915284	up
ASMM9PARTA034233	Fabp4	0.025578842	14.826364	up
ASMM9PARTA030929	Adam23	0.001391964	3.0830736	up
ASMM9PARTA033326	Cryba2	0.0000726	2.0248675	up
ASMM9PARTA041636	Nup214	0.0000521	3.3132715	up
ASMM9PARTA031782	Plcb4	0.00004	2.9125206	up

ASMM9PARTA043526	Eif5	0.000889	2.544023	up
ASMM9PARTA031477	Cartpt	0.00000557	2.2517643	up
ASMM9PARTA027539	Cacng1	0.005914503	3.1773465	up
ASMM9PARTA035781	Prdm16	0.03654214	2.9617462	up
ASMM9PARTA028020	Fgf7	0.003777369	2.6119907	up
ASMM9PARTA030182	Lig1	0.04229518	2.249699	up
ASMM9PARTA032436	Irx4	0.00000854	3.3709266	up
ASMM9PARTA023174	Tmem87a	0.0105444	4.580938	up
ASMM9PARTA028279	Irx3	0.00000704	2.7918754	up
ASMM9PARTA038363	Slc17a6	0.00000287	2.9070206	up
ASMM9PARTA044591	Plac9	0.0000113	2.8673162	up
ASMM9PARTA031936	Shox2	0.000354	2.7292905	up
ASMM9PARTA027617	Cdr2	0.0000347	2.072941	up
ASMM9PARTA033919	Camk2d	0.0000853	2.371512	up
ASMM9PARTA039937	Ces2c	0.008924363	2.5273514	up
ASMM9PARTA029191	Avil	0.000164	2.6097085	up
ASMM9PARTA036767	Rnf6	0.000818	2.313316	up
ASMM9PARTA019879	Ypel4	0.000747	2.0703413	up
ASMM9PARTA024554	Slc25a2	0.000607	2.7162147	up
ASMM9PARTA032130	Dmp1	0.000000577	3.5742388	up
ASMM9PARTA032689	Rbm38	0.000169	2.0112605	up
ASMM9PARTA029091	Sfrp2	0.001410236	2.3574317	up
ASMM9PARTA025104	Tnnt3	0.028151358	9.886942	up
ASMM9PARTA027024	Dcn	0.0000282	2.4121919	up
ASMM9PARTA025333	Tnnt3	0.009303493	61.182983	up
ASMM9PARTA041434	Nphp3	0.000469	2.7009454	up
ASMM9PARTA022135	Cytl1	0.000051	2.4156404	up
ASMM9PARTA034700	Gprasp1	0.000421	2.3494184	up
ASMM9PARTA025392	Tnnt3	0.047394775	8.658881	up
ASMM9PARTA020861	Alpk2	0.015779583	4.2894406	up
ASMM9PARTA040038	Olfr657	0.009732459	3.1989865	up
ASMM9PARTA020569	Thoc2	0.037124027	2.383962	up
ASMM9PARTA033242	Ucn	0.00000919	3.856744	up
ASMM9PARTA029404	Art1	0.02242654	13.814318	up
ASMM9PARTA041066	AI428936	0.000901	2.3417852	up
ASMM9PARTA023235	D0H4S114	0.0000371	2.058481	up
ASMM9PARTA029974	Dlx3	0.001408583	2.5063183	up
ASMM9PARTA027262	Car8	2.08E-08	12.755496	up
ASMM9PARTA020002	Mtus1	0.014918658	2.256344	up
ASMM9PARTA031212	Xirp1	0.013895262	2.7617493	up
ASMM9PARTA042475	A2m	0.000128	2.4170778	up
ASMM9PARTA029210	Ttf1	0.006709633	2.6899865	up
ASMM9PARTA028188	Nefm	0.0000484	2.4131057	up
ASMM9PARTA030563	Pax7	0.0000651	2.627603	up
ASMM9PARTA026717	Rhox3b	0.000352	2.4762418	up
ASMM9PARTA028658	Prkaca	0.0000232	2.1011724	up
ASMM9PARTA025002	Tnnt3	0.027619606	23.66482	up
ASMM9PARTA042019	Lsm12	0.000132	2.1977196	up
ASMM9PARTA041303	Olfm3	0.0000484	2.3936164	up
ASMM9PARTA033634	4930550L24Rik	0.00561765	3.5129473	up
ASMM9PARTA030114	Il16	0.0000637	2.9721432	up
ASMM9PARTA020433	Olfr304	0.015489637	2.0538113	up
ASMM9PARTA039495	Spata20	0.000403	2.4811966	up
ASMM9PARTA029989	Gbx2	0.00006	2.338181	up
ASMM9PARTA026767	9430070O13Rik	0.022252211	0.452086351	down
ASMM9PARTA020476	Osm	0.000958	0.488652511	down
ASMM9PARTA039369	Cd300ld	0.025881946	0.344883362	down

ASMM9PARTA023986	Zfat	0.002198375	0.435569778	down
ASMM9PARTA031340	Cd2	0.0000567	0.172296148	down
ASMM9PARTA032887	Mos	0.024729842	0.411311392	down
ASMM9PARTA024347	Atp6ap1l	0.000571	0.410128955	down
ASMM9PARTA037071	1190003J15Rik	0.000187	0.417253081	down
ASMM9PARTA041835	D630002G06Rik	0.036431365	0.427162257	down
ASMM9PARTA028532	Junb	0.000376	0.493952344	down
ASMM9PARTA043124	Fbxw13	0.000131	0.209202071	down
ASMM9PARTA025664	Gm6904	0.001071501	0.442934079	down
ASMM9PARTA036192	5730508B09Rik	0.000557	0.476970982	down
ASMM9PARTA029251	Tac1	0.0000173	0.43290598	down
ASMM9PARTA043417	Cpa6	0.017711971	0.430763622	down
ASMM9PARTA028804	Saa1	0.001231277	0.397798899	down
ASMM9PARTA033542	Dpys	0.000167	0.327775614	down
ASMM9PARTA031535	Mgll	0.000297	0.400421965	down
ASMM9PARTA030821	Tsks	0.02191947	0.461074691	down
ASMM9PARTA039471	Rin1	7.47E-08	0.351150166	down
ASMM9PARTA023531	9030025P20Rik	0.001066975	0.478101585	down
ASMM9PARTA035576	Asb11	0.000702	0.265205519	down
ASMM9PARTA023040	Synpo	0.00000406	0.369699366	down
ASMM9PARTA029903	Dgcr6	0.007959221	0.330797243	down
ASMM9PARTA043958	Synrg	0.00015	0.416018129	down
ASMM9PARTA044426	Dlx5	0.00000106	0.233779791	down
ASMM9PARTA024385	Slc15a2	9.72E-08	0.04962425	down
ASMM9PARTA036811	Spag16	0.000984	0.465421942	down
ASMM9PARTA022725	Kng2	0.00000727	0.238688891	down
ASMM9PARTA024109	Mup13	0.000000693	0.263233886	down
ASMM9PARTA037199	Rasd2	0.0000382	0.341615225	down
ASMM9PARTA031321	Slc30a3	0.00000746	0.482242319	down
ASMM9PARTA038435	Gsx2	0.000181	0.334285602	down
ASMM9PARTA035300	Hddc3	0.000000454	0.279102491	down
ASMM9PARTA022035	Kcna10	0.000681	0.375963397	down
ASMM9PARTA042546	4931440F15Rik	0.00001	0.323320632	down
ASMM9PARTA023907	Mup19	0.00000847	0.248319522	down
ASMM9PARTA034527	Sostdc1	0.0000296	0.167226745	down
ASMM9PARTA024076	Mup8	0.00000531	0.246651192	down
ASMM9PARTA025656	Dcaf17	0.000226	0.433147439	down
ASMM9PARTA037112	Rpl18a	4.79E-09	0.064035491	down
ASMM9PARTA042154	E130012A19Rik	0.000139	0.488572103	down
ASMM9PARTA038544	2610018G03Rik	0.000645	0.37081792	down
ASMM9PARTA039206	Rab40b	0.0000351	0.411476965	down
ASMM9PARTA020539	Ffar3	0.000978	0.28482073	down
ASMM9PARTA029936	Cyp2d9	0.000167	0.261223548	down
ASMM9PARTA035292	Ino80	0.015004523	0.394612357	down
ASMM9PARTA024121	Mpped2	0.005606	0.389266596	down
ASMM9PARTA022139	Cntnap3	0.003140523	0.40026999	down
ASMM9PARTA036900	Rap2b	0.000482	0.481283603	down
ASMM9PARTA033332	Trpv4	0.00000722	0.399645594	down
ASMM9PARTA024486	Nkx2-1	0.0000648	0.206837489	down
ASMM9PARTA035363	2310046A06Rik	0.000237	0.381426265	down
ASMM9PARTA043876	Slc17a7	0.000129	0.443483249	down
ASMM9PARTA029513	Cd3g	0.005643459	0.395684616	down
ASMM9PARTA044496	Gpr6	0.000000686	0.305557635	down
ASMM9PARTA029838	Cyp2d10	0.000952	0.327348851	down
ASMM9PARTA020724	Lce3f	0.002857118	0.334797178	down
ASMM9PARTA034327	Lrrk2	0.009622876	0.498917076	down
ASMM9PARTA038001	Rem2	0.00000312	0.479569477	down

ASMM9PARTA023568	Mup10	0.0000008	0.191509154	down
ASMM9PARTA024564	Meis2	0.020248394	0.373741673	down
ASMM9PARTA038104	Prss1	0.005254163	0.499804402	down
ASMM9PARTA029737	Dlx5	0.001092952	0.440921715	down
ASMM9PARTA024716	Nek3	0.0260986	0.485719176	down
ASMM9PARTA028744	Serpina1c	0.00000844	0.154268613	down
ASMM9PARTA037934	Cpn1	0.002054692	0.496724697	down
ASMM9PARTA028840	Rcvrn	0.00797785	0.356382909	down
ASMM9PARTA029422	Wnt10a	0.0000369	0.371206616	down
ASMM9PARTA033946	Npy	0.00000139	0.243790295	down
ASMM9PARTA025050	Synpr	0.0000555	0.296299896	down
ASMM9PARTA034613	Mcm8	0.000382	0.43542268	down
ASMM9PARTA041882	Dlgap2	0.001340083	0.493811676	down
ASMM9PARTA029474	Areg	0.000609	0.415910005	down
ASMM9PARTA029528	Dlx2	0.0000111	0.206601569	down
ASMM9PARTA025637	Tpm1	0.000122	0.47664256	down
ASMM9PARTA035470	1700003M02Rik	0.00000899	0.468852356	down
ASMM9PARTA027255	Ambp	0.00000473	0.192091552	down
ASMM9PARTA032497	Fabp1	0.000963	0.464768783	down
ASMM9PARTA042775	Myh8	0.043965533	0.430939379	down
ASMM9PARTA032626	Cyp3a25	0.004287357	0.459424332	down
ASMM9PARTA029223	Apoa1	0.000442	0.077908305	down
ASMM9PARTA041355	Sbsn	0.000112	0.177990322	down
ASMM9PARTA038644	Spata4	0.006757452	0.264950038	down
ASMM9PARTA029819	Fhl2	0.000000333	0.441765305	down
ASMM9PARTA022248	C2cd4b	0.0000194	0.487783891	down
ASMM9PARTA024911	Mup1	0.00000243	0.365613642	down
ASMM9PARTA034985	Kcnv1	0.000206	0.395460321	down
ASMM9PARTA039407	Ell3	0.003593214	0.318624866	down
ASMM9PARTA039643	Rasl10a	0.0000109	0.421555836	down
ASMM9PARTA037343	4930447C04Rik	0.000196	0.493375375	down
ASMM9PARTA037347	Prss23	0.00000158	0.376093992	down
ASMM9PARTA029481	Cnr2	0.021912646	0.428702636	down
ASMM9PARTA025621	BC005561	0.0000237	0.425649775	down
ASMM9PARTA033859	Scube1	0.002698337	0.37310384	down
ASMM9PARTA021788	Thg1l	0.001822639	0.438995554	down
ASMM9PARTA025009	Ptk2b	0.0000133	0.376373429	down
ASMM9PARTA039637	Neto1	0.0000194	0.449146429	down
ASMM9PARTA028385	Hsd3b5	0.0000271	0.112043537	down
ASMM9PARTA037298	Spata2L	0.00000734	0.497100512	down
ASMM9PARTA025133	Crb2	0.0000846	0.479205041	down
ASMM9PARTA037841	Gimap3	0.002224498	0.468160323	down
ASMM9PARTA027570	Cxcr5	0.000216	0.342926367	down
ASMM9PARTA039734	Dmrt2	0.000141	0.424886539	down
ASMM9PARTA036425	Mlf1ip	0.004737627	0.336244878	down
ASMM9PARTA041099	Crygn	0.001326628	0.411536521	down
ASMM9PARTA036647	Cnih3	0.003571751	0.418752104	down
ASMM9PARTA028678	Nppa	0.002488728	0.246553977	down
ASMM9PARTA024746	Myo1b	0.000526	0.45430975	down
ASMM9PARTA025197	Kalrn	0.0000105	0.380450751	down
ASMM9PARTA029830	Chi3l3	0.000372	0.373540135	down
ASMM9PARTA037053	Rasgef1c	0.00000589	0.361700255	down
ASMM9PARTA019493	Tmem158	0.0000107	0.327977612	down
ASMM9PARTA028145	Lbp	0.00000391	0.423863211	down
ASMM9PARTA026369	Fam47e	0.031084172	0.400323253	down
ASMM9PARTA037196	Spata16	0.004009562	0.291892652	down
ASMM9PARTA028854	Serpina1b	0.0000685	0.171326666	down

ASMM9PARTA042935	Ptpn7	0.0000981	0.295138845	down
ASMM9PARTA033099	Stap1	0.000468	0.318098473	down
ASMM9PARTA031779	Cldn5	0.000193	0.498873544	down
ASMM9PARTA024525	Rdh7	0.0000564	0.263748581	down
ASMM9PARTA024745	Palm	0.006232396	0.476590903	down
ASMM9PARTA028615	Mup5	0.000199	0.219583367	down
ASMM9PARTA038804	Kcne2	0.00000038	0.040648903	down
ASMM9PARTA020183	Kcnq2	0.000978	0.356295599	down
ASMM9PARTA027956	Folr1	0.00000886	0.193353138	down
CUST_250_PI426409190	Gm3286	0.001867091	0.499941007	down
ASMM9PARTA040898	Olfr167	0.005143871	0.254419457	down
ASMM9PARTA037379	Slc35d3	0.0000228	0.430735753	down
ASMM9PARTA028599	Raet1c	0.000209	0.350996125	down
ASMM9PARTA022992	Gsg1l	0.000677	0.288710023	down
ASMM9PARTA034098	Emid2	0.000461	0.445201088	down
ASMM9PARTA025229	Lrriq1	0.000754	0.407617524	down
ASMM9PARTA028533	Kcnj4	0.000000832	0.28194335	down
ASMM9PARTA029564	Dpp4	0.0000212	0.374137072	down
CUST_305_PI426409190	Ccl28	0.000161	0.215191546	down
ASMM9PARTA042165	Slc24a5	0.000703	0.432185045	down
ASMM9PARTA043055	Cntln	0.01939386	0.445860517	down
ASMM9PARTA039135	Wnt9a	0.00000649	0.3313074	down
ASMM9PARTA029840	Efnb2	0.000642	0.466073746	down
ASMM9PARTA030751	Pcdh10	0.0000577	0.44293347	down
ASMM9PARTA033039	Fbxo3	0.0000246	0.232778987	down
ASMM9PARTA044005	Fam120c	0.002607773	0.432206601	down
ASMM9PARTA020791	Frmpd4	0.019704299	0.464463438	down
ASMM9PARTA044281	Trat1	0.002789541	0.38111133	down
ASMM9PARTA042886	Clec12a	0.000069	0.412363686	down
ASMM9PARTA022794	Rbm8a	5.09E-08	0.173634654	down
ASMM9PARTA024749	Myef2	0.0000605	0.087595044	down
ASMM9PARTA021427	Pdxdc1	7.17E-08	0.066147392	down
ASMM9PARTA040145	Olfr360	0.043136913	0.45242719	down
ASMM9PARTA030600	Rgs9	4.13E-08	0.157634971	down
ASMM9PARTA027393	Alx4	0.019655824	0.488700631	down
ASMM9PARTA030794	Ccl21a	0.000125	0.373032941	down
ASMM9PARTA029972	Lhx8	0.000451	0.273172216	down
ASMM9PARTA029029	Serpina1e	0.00000637	0.111211498	down
ASMM9PARTA043911	Clec18a	0.000204	0.468546541	down
ASMM9PARTA041884	Mgat5b	0.001188887	0.410534614	down
ASMM9PARTA037497	Dtl	0.000063	0.33030742	down
ASMM9PARTA042802	C730048C13Rik	0.001696692	0.228537302	down
ASMM9PARTA036144	2010300C02Rik	0.00000897	0.495064749	down
ASMM9PARTA022198	Ccno	0.0000204	0.44022172	down
ASMM9PARTA036982	Trim14	0.006627751	0.271392603	down
ASMM9PARTA023802	LOC100048885	0.000912	0.440673882	down
ASMM9PARTA036624	Mei1	0.000481	0.192106904	down
ASMM9PARTA029472	Alb	0.0000183	0.128460975	down
ASMM9PARTA023296	Gm3448	0.0000221	0.204595836	down
ASMM9PARTA041022	Homer1	0.000195	0.4672583	down
ASMM9PARTA025371	Fbxl16	0.0000458	0.421689765	down
ASMM9PARTA035719	Lyzl4	0.000707	0.410946902	down
ASMM9PARTA043635	Ppip5k1	0.000437	0.235961211	down
ASMM9PARTA041980	Dclk3	0.0000132	0.443511533	down
ASMM9PARTA030793	Prkcc	0.00039	0.481780623	down
ASMM9PARTA029622	Clca1	0.0000439	0.179811162	down
ASMM9PARTA022966	Gm10334	0.0000355	0.344562629	down

ASMM9PARTA030118	Krt10	0.016283398	0.414397839	down
ASMM9PARTA028483	Pomc	0.000386	0.438948825	down
ASMM9PARTA020879	Gpr55	0.004020004	0.428410303	down
ASMM9PARTA023887	Plcb1	0.00000484	0.39348403	down
ASMM9PARTA022657	Wdr86	0.000084	0.321698258	down
ASMM9PARTA025643	Ankrd33b	0.030486941	0.479541949	down
ASMM9PARTA037277	6330527O06Rik	0.000389	0.35357512	down
ASMM9PARTA032248	Vpreb1	0.001076197	0.438437846	down
ASMM9PARTA022342	Htra4	0.002256475	0.429942355	down
ASMM9PARTA022506	Gm1337	9.39E-08	0.115969021	down
ASMM9PARTA038497	Slco1a5	0.00082	0.35039958	down
ASMM9PARTA024512	Tmem116	0.008582863	0.462072276	down
ASMM9PARTA022895	Gm7244	0.000237	0.186893122	down
ASMM9PARTA022009	Ccl27a	0.00000109	0.464213503	down
ASMM9PARTA025647	Acnat1	0.0000709	0.42080132	down
ASMM9PARTA039741	Kcna5	0.000000857	0.379502033	down
ASMM9PARTA033683	Ms4a4b	0.00680673	0.435526905	down
ASMM9PARTA037619	Tlr1	0.000246	0.300632775	down
ASMM9PARTA042570	Mylk3	0.00083	0.27851631	down
ASMM9PARTA027011	Mup15	0.00000139	0.219367231	down
ASMM9PARTA026146	Slc10a1	0.000169	0.191183533	down
ASMM9PARTA035444	Pnlip	0.00023	0.264036413	down
ASMM9PARTA020699	Tmem171	0.014505961	0.328212109	down
ASMM9PARTA033794	Ypel1	0.000274	0.460009045	down
ASMM9PARTA020062	Ddn	0.00000132	0.409100289	down
ASMM9PARTA033750	Scel	0.006426611	0.272576786	down
ASMM9PARTA029488	Cyp8b1	0.0000753	0.246691059	down
ASMM9PARTA025601	Mup11	0.00000147	0.242243309	down
ASMM9PARTA030083	Neurog1	0.003499295	0.449113629	down
ASMM9PARTA029588	Drd1a	0.000000133	0.083367695	down
ASMM9PARTA028004	Hpd	0.000891	0.278931384	down
ASMM9PARTA040029	Itpka	0.0000912	0.396902305	down
ASMM9PARTA044070	Exoc8	0.00000456	0.32967305	down
ASMM9PARTA030270	Gdnf	0.001346218	0.435908256	down
ASMM9PARTA039331	Gpx6	0.009131108	0.207561089	down
ASMM9PARTA043423	Pacsin1	0.003091733	0.351882094	down
ASMM9PARTA037799	Clca2	0.000532	0.462275264	down
ASMM9PARTA038744	Fam64a	0.000234	0.403512512	down
ASMM9PARTA020109	Kcnq2	0.00000479	0.428725243	down
ASMM9PARTA030509	Myl4	0.00000374	0.209806173	down
ASMM9PARTA020324	Skap1	0.000184	0.352408476	down
ASMM9PARTA030022	H2-Q10	0.000502	0.363219177	down
ASMM9PARTA043826	Cwh43	0.007104415	0.447181393	down
ASMM9PARTA025525	Nfatc1	0.012698671	0.417379237	down
ASMM9PARTA038893	Defb9	0.0000283	0.266064694	down
ASMM9PARTA027895	Htr1f	0.001730357	0.290470221	down
ASMM9PARTA031618	Cd3d	0.018658267	0.332891808	down
ASMM9PARTA043662	Vmac	0.01991689	0.413505161	down
ASMM9PARTA036543	Ttc9b	0.0000502	0.408683778	down
ASMM9PARTA026120	Gpr19	0.00033	0.45289451	down
ASMM9PARTA041000	Arhgap15	0.000237	0.423609682	down
ASMM9PARTA028464	Mesp2	0.00231803	0.491156771	down
ASMM9PARTA022663	Stfa1	0.001452552	0.359268766	down
ASMM9PARTA029526	Dazl	0.0000578	0.471517691	down
ASMM9PARTA030528	Ids	0.01330641	0.456687568	down
ASMM9PARTA042944	Sh3pxd2b	0.017831616	0.38603245	down
ASMM9PARTA021408	Trpm3	0.00089	0.328878174	down

ASMM9PARTA019942	Tmem232	0.000842	0.468943293	down
ASMM9PARTA022042	Hs3st2	0.000012	0.335948367	down
ASMM9PARTA024855	C2cd4a	0.001719892	0.473813287	down
ASMM9PARTA040559	Olfr641	0.006276009	0.482602703	down
ASMM9PARTA033543	Folr4	0.024941446	0.363060813	down
ASMM9PARTA043314	Spata21	0.0000566	0.416377978	down
ASMM9PARTA033641	Gpr88	0.000000817	0.064518789	down
ASMM9PARTA033394	Kcnmb4	0.0000262	0.454633137	down
ASMM9PARTA038599	Cdhr1	0.001211736	0.447039938	down
ASMM9PARTA038797	Mbtd1	0.00000799	0.289010767	down
ASMM9PARTA043103	4933400C05Rik	0.000329	0.499477871	down
ASMM9PARTA027245	Mup12	8.94E-09	0.258872901	down
ASMM9PARTA032318	Syt5	0.000793	0.408389146	down
ASMM9PARTA034590	Fam187a	0.000324	0.334695399	down
ASMM9PARTA028286	Ltb	0.011367448	0.461843847	down
ASMM9PARTA035746	Steap1	0.0000137	0.173353278	down
ASMM9PARTA023758	Samd3	0.000278	0.453253579	down
ASMM9PARTA029274	Thrb	0.018455353	0.202143318	down
ASMM9PARTA035865	Krtap4-13	0.0000392	0.435048594	down
ASMM9PARTA023546	Serac1	0.005735342	0.370959	down
ASMM9PARTA037291	Tmem116	0.010050296	0.38057123	down
ASMM9PARTA040990	Rgs13	0.001450367	0.19450609	down
ASMM9PARTA025728	Gm7455	0.001019644	0.419449944	down
ASMM9PARTA037288	Ocel1	0.000356	0.385815062	down
ASMM9PARTA033122	Spib	0.005946528	0.446119634	down
ASMM9PARTA040340	Olfr1238	0.007549198	0.288090099	down
ASMM9PARTA041362	Pus7l	0.034232195	0.472554886	down
ASMM9PARTA042804	Kif6	0.003215302	0.476097796	down
ASMM9PARTA033257	Doc2g	0.000000305	0.400275117	down
ASMM9PARTA030637	Saa2	0.000265	0.429018411	down
ASMM9PARTA033004	Cldn9	0.0000199	0.444997032	down
ASMM9PARTA029272	Tbr1	0.01312867	0.237979591	down
ASMM9PARTA030000	Hal	0.04055374	0.454654344	down
ASMM9PARTA031999	Bhmt	0.000000616	0.235375862	down
ASMM9PARTA021231	Iqgap3	0.00160808	0.401064184	down
ASMM9PARTA023055	Calml4	0.000327	0.39455598	down
ASMM9PARTA024843	Lmo4	0.000306	0.435187579	down
ASMM9PARTA024174	Utp14b	0.009906592	0.442702107	down
ASMM9PARTA028812	Serpina1d	0.0000055	0.164241261	down
ASMM9PARTA041388	Zdhhc8	0.003374993	0.428042924	down
ASMM9PARTA024343	Cnih3	0.0000244	0.483315233	down
ASMM9PARTA023189	Zfp385b	0.047750082	0.424597403	down
ASMM9PARTA042712	6530418L21Rik	0.000197	0.48622914	down
ASMM9PARTA019903	Ces5a	0.010479536	0.499057106	down
ASMM9PARTA034801	Fam132a	0.0000485	0.470912001	down
ASMM9PARTA024138	Mup7	0.000000384	0.240719841	down
ASMM9PARTA020035	Gm5409	0.000341	0.262294965	down
ASMM9PARTA021692	Mup3	0.00000519	0.20712954	down
ASMM9PARTA044724	Olfr741	0.039275255	0.340936827	down
ASMM9PARTA034885	4930549C01Rik	0.001364725	0.48323029	down
ASMM9PARTA029754	Avp	0.000159	0.238787181	down
ASMM9PARTA038807	Vmn1r4	0.009277049	0.363759076	down
ASMM9PARTA036034	Lrrc34	0.004095026	0.485777267	down
ASMM9PARTA028663	Prg2	0.00021	0.303465298	down
ASMM9PARTA023422	Arhgef16	0.003660981	0.491400347	down
ASMM9PARTA044240	Raet1e	0.000147	0.408908213	down
ASMM9PARTA036074	Serpina9	0.00000128	0.139742879	down

ASMM9PARTA028515	Lgals7	0.000832	0.411405205	down
ASMM9PARTA027144	Mup17	9.18E-08	0.237956385	down
ASMM9PARTA028014	F5	0.001756891	0.094705543	down
ASMM9PARTA031603	Pde10a	0.000667	0.336120139	down
ASMM9PARTA032589	Tbx21	0.008143852	0.407656507	down
ASMM9PARTA026982	Adrb1	0.0000497	0.493028503	down
ASMM9PARTA029518	Cldn3	0.0000986	0.474748462	down
ASMM9PARTA035348	Iqcf3	0.00000504	0.251657903	down
ASMM9PARTA026871	Ccl27b	0.000918	0.463315855	down
ASMM9PARTA043728	Me3	0.00000145	0.382131121	down
ASMM9PARTA032630	Copz2	0.000618	0.360385464	down
ASMM9PARTA028873	Psp	0.01353761	0.460102087	down
ASMM9PARTA022196	Efcab3	0.000194	0.308778781	down
ASMM9PARTA035371	Pnma1	0.000145	0.493553841	down
ASMM9PARTA021245	Baiap2	0.001763001	0.446760051	down
ASMM9PARTA043655	Aldh8a1	0.00626015	0.477983689	down
ASMM9PARTA038944	Upb1	0.000268	0.310357447	down
ASMM9PARTA032151	Igfbpl1	0.001696612	0.356494661	down
ASMM9PARTA019794	Zfp457	0.00000806	0.419589598	down
ASMM9PARTA025617	Ptp4a3	0.00012	0.464946887	down
ASMM9PARTA025047	1700024P16Rik	0.0000138	0.365314819	down
ASMM9PARTA029071	Tgm3	0.004360132	0.25541814	down
ASMM9PARTA028583	Mup2	0.000000304	0.200158125	down
ASMM9PARTA043495	Hist1h2ac	0.000478	0.247768443	down
ASMM9PARTA038077	Kcnh7	0.000887	0.482377798	down
ASMM9PARTA036492	Zswim2	0.002380232	0.381945337	down
ASMM9PARTA031215	Zscan21	0.001165335	0.391873761	down
ASMM9PARTA021779	Nme2	0.0000443	0.391396357	down
ASMM9PARTA039721	Hkdc1	0.0000017	0.209026998	down
ASMM9PARTA037178	1700027A23Rik	0.035409767	0.488726712	down
ASMM9PARTA040779	Zc3h12a	0.0000529	0.458695208	down
ASMM9PARTA030354	Krt18	0.00358591	0.211399038	down
ASMM9PARTA032678	Extl3	0.008541565	0.481504451	down
ASMM9PARTA037818	Cecr6	0.00000795	0.319504385	down
ASMM9PARTA032072	Rgs14	0.00000326	0.256738249	down
ASMM9PARTA027052	Mup14	0.000000115	0.198829254	down
ASMM9PARTA026834	Kif17	0.0000107	0.20289481	down
ASMM9PARTA038186	Cacng6	0.011634019	0.270069565	down
ASMM9PARTA037382	Pbp2	3.65E-08	0.092656036	down
ASMM9PARTA027458	Arx	0.00000244	0.331524283	down
ASMM9PARTA024920	Hsd17b13	0.006204337	0.436534116	down
ASMM9PARTA021544	Mup2	0.000000852	0.243681402	down
ASMM9PARTA036026	Mettl7b	0.015865378	0.416268783	down
ASMM9PARTA033165	Aurkc	0.002146789	0.460917131	down
ASMM9PARTA041403	Ptk2b	0.0000207	0.465016484	down
ASMM9PARTA021757	Arl4a	0.004045966	0.492928157	down
ASMM9PARTA026647	Arpp21	0.00000417	0.42627672	down
ASMM9PARTA036056	Ankrd33b	0.034586582	0.326527254	down
ASMM9PARTA023928	Prdm12	0.00000133	0.040939831	down
ASMM9PARTA032107	Stx1a	0.00000303	0.414641007	down
ASMM9PARTA019931	Gp1bb	0.0000432	0.387638142	down
ASMM9PARTA027327	Hrk	0.0000198	0.466530278	down
ASMM9PARTA043801	Fgb	0.002688705	0.318784132	down
ASMM9PARTA030247	H2-Ea-ps	0.0000609	0.211806537	down
ASMM9PARTA029902	Cyp21a1	0.001671015	0.486479281	down
ASMM9PARTA025240	Zfp605	0.0000085	0.342701349	down
ASMM9PARTA040940	Olfr520	0.011228737	0.334146334	down

ASMM9PARTA027124	Alx3	0.00000651	0.305562583	down
ASMM9PARTA033533	Pvrl3	0.000378	0.396664527	down
ASMM9PARTA029980	Celf2	0.0000208	0.478995726	down
ASMM9PARTA031893	Prss30	0.007131813	0.384441794	down
ASMM9PARTA029562	Col6a1	0.0000977	0.366510526	down
ASMM9PARTA042840	Casc1	0.01858521	0.483804172	down
ASMM9PARTA037618	Akr1c6	0.000099	0.360744635	down
ASMM9PARTA022728	Kif19a	0.018955346	0.409161184	down
ASMM9PARTA019886	Mup21	0.011602221	0.417094675	down
ASMM9PARTA030904	Ptger3	0.0000484	0.491593699	down
ASMM9PARTA040373	Olfr539	0.03280855	0.367703458	down
ASMM9PARTA031468	Ahsg	0.000318	0.463748673	down
ASMM9PARTA043607	Jakmip1	0.000576	0.427614909	down
ASMM9PARTA029867	Dlx1	0.000966	0.296238497	down
ASMM9PARTA044501	Tas2r135	0.006770704	0.386129189	down
ASMM9PARTA029376	Cst7	0.008895044	0.446155003	down
ASMM9PARTA043518	Lingo1	0.00000721	0.477384232	down
ASMM9PARTA041595	Fam132b	0.000235	0.35978574	down
ASMM9PARTA031347	Wfs1	0.00000463	0.42589563	down
ASMM9PARTA029787	Adam18	0.000525	0.249828905	down
ASMM9PARTA044787	Kcnf1	0.000000429	0.428665606	down
ASMM9PARTA032969	Extl1	0.000000461	0.354690124	down
ASMM9PARTA028803	Rxrg	0.000791	0.339307406	down
ASMM9PARTA031895	Dkkl1	0.0000125	0.209419917	down
ASMM9PARTA029412	Casq1	0.006500153	0.408109212	down
ASMM9PARTA044103	Tcte3	0.000712	0.4682233	down
ASMM9PARTA029639	Emx1	0.0000499	0.415882831	down
ASMM9PARTA041662	Shisa7	0.0000156	0.40882212	down
ASMM9PARTA043698	Kcns2	0.000136	0.472383872	down
ASMM9PARTA031080	Sh3bp2	0.001149456	0.470393968	down
ASMM9PARTA023494	Wtap	3.03E-09	0.031338052	down
ASMM9PARTA019803	Sp9	0.000008	0.369539113	down
ASMM9PARTA022493	Chrna10	0.000196	0.068845726	down
ASMM9PARTA028739	Cxcl5	0.000186	0.298476074	down
ASMM9PARTA034114	Lrrc57	0.018392675	0.440025338	down
ASMM9PARTA029853	Gnal	0.000593	0.386547976	down
ASMM9PARTA027417	Adh1	0.00000265	0.498736377	down
ASMM9PARTA027349	LOC100048884	0.000000371	0.235953488	down
ASMM9PARTA024051	Slc6a18	0.04500027	0.47534007	down
ASMM9PARTA035886	Otud1	0.0000946	0.485737296	down
ASMM9PARTA039876	Gimap7	0.045070495	0.493111625	down
ASMM9PARTA020403	Mup20	0.00000165	0.146485698	down
ASMM9PARTA032320	Vpreb2	0.032925587	0.246151998	down
ASMM9PARTA031630	Oprd1	0.002697941	0.32093487	down
ASMM9PARTA031920	Matn2	0.000139	0.356933363	down
ASMM9PARTA040156	Sord	0.000456	0.416700003	down
ASMM9PARTA021430	Cypt8	0.033588585	0.25303629	down
ASMM9PARTA037044	Sash3	0.006988749	0.419684231	down
ASMM9PARTA023313	Lrrc10b	0.0000606	0.145081104	down
ASMM9PARTA043309	Cnksr2	0.000000854	0.409904271	down
ASMM9PARTA030452	Ptpru	0.048888586	0.178223034	down
ASMM9PARTA023064	Kif21a	0.000337	0.473850849	down
ASMM9PARTA029861	Nr4a1	0.00000712	0.495284397	down
ASMM9PARTA027638	H2-Oa	0.001764614	0.339601229	down
ASMM9PARTA032080	Ttr	2.53E-08	0.023502838	down
ASMM9PARTA042398	Sp110	0.005624052	0.333696685	down
ASMM9PARTA020753	Tmem90a	0.0000035	0.412804553	down

ASMM9PARTA024030	Spdya	0.022558821	0.488107576	down
ASMM9PARTA039413	Cdca7l	0.024608998	0.318074302	down
ASMM9PARTA038922	Akr1c18	0.000137	0.321200385	down
ASMM9PARTA026977	1700027A23Rik	0.009445014	0.421691347	down
ASMM9PARTA029837	Cyp1a2	0.033267375	0.318228774	down
ASMM9PARTA029700	Usp17l5	0.020234888	0.255496888	down
ASMM9PARTA042085	Dact2	0.000483	0.441092397	down
ASMM9PARTA043232	Sntn	0.0000823	0.45459484	down
ASMM9PARTA044515	Cd200r4	0.000637	0.45578357	down
ASMM9PARTA030909	S100a5	1.78E-09	0.006571571	down
ASMM9PARTA021134	Gm4925	0.030946357	0.413789981	down
ASMM9PARTA038735	Calml4	0.0000365	0.388611567	down
ASMM9PARTA030666	Serpinb8	0.0000342	0.248758286	down
ASMM9PARTA032454	Nptx2	0.00000167	0.471727374	down
ASMM9PARTA021303	Gm904	0.031213142	0.284344363	down
ASMM9PARTA026985	Aqp1	0.00000908	0.2612922	down
ASMM9PARTA032496	Ear7	0.000302	0.162439971	down
ASMM9PARTA036763	Arpp21	0.0000241	0.233984547	down
ASMM9PARTA024104	Ano3	0.00000844	0.333681808	down
ASMM9PARTA037707	Actn2	0.001058476	0.311369864	down
ASMM9PARTA031112	Hcst	0.004789569	0.481713598	down
ASMM9PARTA039330	Ifi27l2b	0.027126346	0.227597934	down
ASMM9PARTA042899	C130050O18Rik	0.0044037	0.371811567	down
ASMM9PARTA041681	Clic6	0.000163	0.147663244	down
ASMM9PARTA039062	Syt17	0.000723	0.230432512	down
ASMM9PARTA027690	Ear2	0.000088	0.497211588	down
ASMM9PARTA038827	Fgg	0.000139	0.119546112	down
ASMM9PARTA028365	Lmo2	0.000000168	0.443729704	down
ASMM9PARTA041439	Zbbx	0.002242688	0.421385801	down
ASMM9PARTA037765	Mesdc1	0.000000474	0.1469304	down
ASMM9PARTA021098	Trpm3	0.0000562	0.260835226	down
ASMM9PARTA033518	Chia	0.000694	0.30522827	down
ASMM9PARTA044316	Phactr1	0.002261274	0.295423244	down
ASMM9PARTA031019	Ccl3	0.002990186	0.46892658	down
ASMM9PARTA034000	1500015O10Rik	0.00000964	0.395420321	down
ASMM9PARTA031245	Tcf21	0.03712478	0.379334465	down
ASMM9PARTA032373	Gpr56	0.03331314	0.478418059	down
ASMM9PARTA037945	Ocm	0.00009	0.24254921	down
ASMM9PARTA028493	Icam5	0.0000405	0.397271097	down
ASMM9PARTA039576	Tmem184a	0.000617	0.334750219	down
ASMM9PARTA028309	Hlx	0.027598886	0.333741076	down
ASMM9PARTA038969	Olfml3	0.0000386	0.471053969	down
ASMM9PARTA043294	3110035E14Rik	0.0000299	0.486688628	down
ASMM9PARTA044092	Zfp811	0.000501	0.454742275	down
ASMM9PARTA042997	4833427G06Rik	0.000173	0.395600714	down
ASMM9PARTA034826	1300014I06Rik	0.00000409	0.075304193	down
ASMM9PARTA039480	Rgs9bp	0.011031129	0.474324574	down
ASMM9PARTA033105	Slc16a8	0.00000483	0.122152876	down
ASMM9PARTA024981	Mup1	0.00000201	0.192426738	down
ASMM9PARTA035663	2010001M09Rik	0.000167	0.445064429	down
ASMM9PARTA024133	Gpr52	0.000442	0.294673896	down
ASMM9PARTA033199	Arhgap31	0.042125855	0.434491499	down
ASMM9PARTA028030	Grin2b	0.0000158	0.480857658	down
ASMM9PARTA028098	Cd79b	0.0000194	0.232821244	down
ASMM9PARTA021726	C030030A07Rik	0.00000033	0.047905447	down
ASMM9PARTA038214	Ftcd	0.001861306	0.371297376	down
ASMM9PARTA021267	Lrrc8b	0.0000105	0.434414301	down

ASMM9PARTA029391	Ezr	0.00000115	0.494731114	down
ASMM9PARTA020808	Rprml	0.00000241	0.383029187	down
ASMM9PARTA039168	Defb11	7.53E-08	0.010289556	down
ASMM9PARTA031186	Prss3	0.0000034	0.200648335	down
ASMM9PARTA031616	Adra1d	0.0000146	0.379005376	down
ASMM9PARTA029005	Uox	0.003978182	0.354838989	down
ASMM9PARTA029095	St8sia3	0.0000291	0.422561077	down
ASMM9PARTA035887	Cage1	0.004676535	0.251412688	down
ASMM9PARTA030596	Rasgrp1	0.0000263	0.403056896	down
ASMM9PARTA024629	Lrrc57	0.001721961	0.419265203	down
ASMM9PARTA033853	Pde6h	0.000234	0.207052485	down
ASMM9PARTA032344	Agxt	0.000216	0.360265886	down
ASMM9PARTA022993	Cldn20	0.004639955	0.493985846	down
ASMM9PARTA029204	Serpina1a	0.00000194	0.151836845	down
ASMM9PARTA022341	6330545A04Rik	0.000197	0.473756743	down
ASMM9PARTA037816	Arpp21	0.005381466	0.450126501	down
ASMM9PARTA029649	Neurod6	0.000112	0.407858204	down
ASMM9PARTA039879	Cplx3	0.046604663	0.410838647	down
ASMM9PARTA021132	Gm5464	0.042043246	0.282848241	down
ASMM9PARTA022526	Neurl1b	0.0000148	0.385882891	down
ASMM9PARTA042283	Tnks	0.000736	0.406309004	down
ASMM9PARTA031376	Crabp1	0.0000437	0.298028642	down
ASMM9PARTA021102	Slc38a6	0.000815	0.47376489	down
ASMM9PARTA023217	Rad51ap2	0.000554	0.42084237	down
ASMM9PARTA039245	Ccdc21	0.0000508	0.184061127	down
ASMM9PARTA037688	Fcrls	0.00000577	0.314456351	down
ASMM9PARTA030183	Lmo4	0.00000263	0.444368171	down
ASMM9PARTA040044	Camkv	0.0000616	0.431609603	down
ASMM9PARTA025273	Efemp2	0.00045	0.490483733	down
ASMM9PARTA034600	Camk2n1	0.00000024	0.089560641	down
ASMM9PARTA032643	Pdyn	0.0000172	0.206859032	down
ASMM9PARTA037977	Krt8	0.010386748	0.453967678	down
ASMM9PARTA039789	Cyp2c70	0.005116757	0.361539539	down
ASMM9PARTA030522	Pou2f3	0.004801637	0.477644312	down
ASMM9PARTA019655	Cyp2c44	0.0000653	0.315705396	down
ASMM9PARTA028417	Prss12	0.000944	0.362782585	down
ASMM9PARTA028427	Klra2	0.000753	0.335705563	down
ASMM9PARTA033027	Hspb3	0.00611546	0.341625612	down
ASMM9PARTA037597	Aldh1a3	0.00646126	0.409539569	down
ASMM9PARTA044604	Irgc1	0.000239	0.230403279	down
ASMM9PARTA034368	Klhl10	0.019284684	0.392400366	down
ASMM9PARTA027435	Cort	0.00000644	0.318111588	down
ASMM9PARTA041911	Rspo2	0.001337609	0.455847999	down
ASMM9PARTA035258	Rasl11b	0.0000154	0.4323772	down
ASMM9PARTA035862	Cst12	0.048235975	0.380437768	down
ASMM9PARTA036919	4930563D23Rik	0.000732	0.347832693	down
ASMM9PARTA041635	Tmem132d	0.023052653	0.367791336	down
ASMM9PARTA041806	St6gal2	0.046759516	0.408202919	down
ASMM9PARTA040011	Kcnip2	0.000667	0.463640049	down
ASMM9PARTA036385	Amdhd1	0.00809693	0.351826928	down
ASMM9PARTA022906	Apold1	0.000171	0.463313773	down
ASMM9PARTA033534	Pvrl3	0.029569859	0.349675524	down
ASMM9PARTA042913	Dmrt3	0.00000511	0.432159028	down
ASMM9PARTA036300	Pebp4	0.012370867	0.414217948	down
ASMM9PARTA029190	Adora2a	7.49E-08	0.080057033	down
ASMM9PARTA034317	Eid3	0.030599816	0.425848275	down
ASMM9PARTA027162	Cckbr	0.000202	0.361715915	down

ASMM9PARTA027287	Ccl27b	0.00000899	0.407475795	down
ASMM9PARTA022057	Ccdc90a	0.000238	0.334822467	down
ASMM9PARTA043502	Rnf24	0.026811577	0.366931973	down
ASMM9PARTA022859	Akap5	0.0000251	0.352882332	down
ASMM9PARTA039800	Lrrc67	0.000721	0.445708772	down
ASMM9PARTA032665	Ngef	0.000044	0.374574343	down
ASMM9PARTA027477	Cr2	0.001127469	0.150615847	down
ASMM9PARTA029467	Zfp185	0.009606877	0.462801004	down
ASMM9PARTA040989	Acat3	0.00000378	0.471443045	down
ASMM9PARTA032247	Spint1	0.000117	0.151463906	down
ASMM9PARTA029905	Dlx6	0.006954233	0.12914653	down
ASMM9PARTA023153	Dclk1	0.00000439	0.474714724	down
ASMM9PARTA042339	Fam171b	0.0000253	0.27883114	down
ASMM9PARTA020570	Gm1060	0.000258	0.463459422	down
ASMM9PARTA029811	Dsg1a	0.000413	0.228038782	down
ASMM9PARTA040858	Neurl3	0.003668207	0.415740449	down
CUST_292_PI426409190	Gm13298	0.0000412	0.383049449	down
ASMM9PARTA033719	Apoc3	0.0000179	0.371161976	down
ASMM9PARTA026413	Gm13476	0.025168736	0.493887403	down
ASMM9PARTA033799	Rprm	0.00000914	0.430396161	down
ASMM9PARTA035223	1110059M19Rik	0.00000468	0.077540015	down
