# Supplementary material for: Microarray Profile of Long Noncoding RNA and Messenger RNA Expression in a Model of Alzheimer’s Disease
Source: Life (Basel). 2020 May 14;10(5):64. doi: 10.3390/life10050064 (PMC7281340; doi:10.3390/life10050064)
Supplement: Supplementary file 1 [file life-10-00064-s001.zip › life-787240-supplemenatry-to be published - PDF/life-787240-supplementary/Table S8.pdf]

life

Supplementary

Microarray Profile of Long Noncoding RNA and Messenger RNA Expression in a Model of Alzheimer's Disease

Linlin Wang †, Li Zeng †, Hailun Jiang, Zhuorong Li * and Rui Liu *

Institute of Medicinal Biotechnology, Chinese Academy of Medical Sciences and Peking Union Medical College, Beijing 100050, China; wanglinlin@wfmc.edu.cn (L.W.); zengsheng@imb.pumc.edu.cn (L.Z.); jianghailun@imb.pumc.edu.cn (H.J.)

* Correspondence: lizhourong@imb.pumc.edu.cn (Z.L.); +86-10-8352017; .liurui@imb.pumc.edu.cn (R.L.); Tel.: +86-10-67087731

Table S8. Differently expressed mRNAs in the brain of 9-month-old APP/PS1 mice compared with age-matched WT mice.

Probe Name	Symbol	*p*-Value	Fold Change	Regulation
ASMM9PARTA038194	Cml2	0.045779776	3.6246243	up
ASMM9PARTA027886	Gsta1	0.00000359	53.68711	up
ASMM9PARTA045052	Olfr220	0.012035505	2.4770977	up
ASMM9PARTA027293	Spp1	0.000272	4.6906066	up
ASMM9PARTA025747	Tshb	0.00000154	15.374761	up
ASMM9PARTA033934	Asb4	0.011208599	2.3513675	up
ASMM9PARTA037561	Dbr1	0.0000156	2.284718	up
ASMM9PARTA031760	Cpt1a	0.02994442	2.0144393	up
ASMM9PARTA028605	Lgals1	0.000781	2.9727602	up
ASMM9PARTA032887	Mos	0.031983916	2.0967693	up
ASMM9PARTA032449	Rbms2	0.000788	2.1443632	up
ASMM9PARTA044292	Zhx2	0.004828548	3.0402644	up
ASMM9PARTA038784	Cyp2a12	0.003379141	2.8495603	up
ASMM9PARTA044232	C1qtnf9	0.003571573	2.2076929	up
ASMM9PARTA037203	Arhgap24	0.003013721	2.4376557	up
ASMM9PARTA031750	Slc27a1	0.00000665	4.0185995	up
ASMM9PARTA038638	Pygl	0.00000535	3.7015345	up
ASMM9PARTA032573	Rdh7	0.014953258	3.7964184	up
ASMM9PARTA025350	Tpm1	0.000949	2.423386	up
ASMM9PARTA025501	Tpm1	0.01789245	2.1569705	up
ASMM9PARTA028756	Pax1	0.011056793	2.3572633	up
ASMM9PARTA027822	Gch1	0.0000671	2.8479276	up
ASMM9PARTA026869	Dnmt1	0.002123956	3.2389054	up
ASMM9PARTA044461	Rsl24d1	0.00000228	4.489637	up
ASMM9PARTA036319	1700001F09Rik	0.000445	2.8995903	up
ASMM9PARTA035878	Mapk6	0.029392151	4.8868704	up
ASMM9PARTA022999	Kng1	0.000029	6.634968	up
ASMM9PARTA028991	Slc4a3	0.005388057	2.2340128	up

ASMM9PARTA031296	Azgp1	0.0000758	53.193203	up
ASMM9PARTA028762	Pipox	0.008351406	2.0164452	up
ASMM9PARTA028034	Cfb	0.0000159	22.322893	up
ASMM9PARTA032662	Ncoa4	0.00755903	2.5767996	up
ASMM9PARTA025888	Abca6	0.0000352	3.1062806	up
ASMM9PARTA025718	Cdr1	0.0000644	2.13334	up
ASMM9PARTA042349	Qrich1	0.026870431	4.2185373	up
ASMM9PARTA043488	Slfn8	0.003016642	4.9476	up
ASMM9PARTA023561	Epb4.1l5	0.001206713	2.00441	up
ASMM9PARTA044235	Slc23a3	0.0000338	3.5199783	up
ASMM9PARTA028804	Saa1	6.62E-08	33.61684	up
ASMM9PARTA037501	Mrap	0.002060056	5.355639	up
ASMM9PARTA038270	Gpnmb	0.029276676	2.5159812	up
ASMM9PARTA034297	Baiap2l1	0.014289469	2.5955942	up
ASMM9PARTA033992	Tnfrsf22	0.001479952	2.4460945	up
ASMM9PARTA020618	Nxf3	0.026787411	2.1621056	up
ASMM9PARTA021063	Trpm3	0.000681	2.2152126	up
ASMM9PARTA028895	Trh	0.00000126	3.0509381	up
ASMM9PARTA036284	Anks4b	0.014252404	2.8966143	up
ASMM9PARTA027698	Gc	0.00000403	109.52732	up
ASMM9PARTA033615	Bhmt2	0.005609026	4.055277	up
ASMM9PARTA024997	Nr1h4	0.029813219	2.263736	up
ASMM9PARTA023618	Krtap11-1	0.002912072	2.4261823	up
ASMM9PARTA043364	Reep3	0.00039	2.1506965	up
ASMM9PARTA021502	Raph1	0.004872218	6.241117	up
ASMM9PARTA033443	B4galt1	0.01023507	2.0386043	up
ASMM9PARTA033640	Stk32b	0.036292892	2.0242531	up
ASMM9PARTA028934	Tac2	0.00000103	2.00584	up
ASMM9PARTA041340	Phc3	0.032855276	2.1730123	up
ASMM9PARTA043987	1100001G20Rik	0.000982	5.176354	up
ASMM9PARTA026193	Tmem11	0.006078165	2.4421546	up
ASMM9PARTA035974	Dzip3	0.000312	2.655217	up
ASMM9PARTA032094	Tmprss2	0.005929107	8.4456625	up
ASMM9PARTA029814	F7	0.019000951	2.0754564	up
ASMM9PARTA043891	Nxph4	0.0000255	6.4234366	up
ASMM9PARTA022309	Tsc22d2	0.018254824	3.8422744	up
ASMM9PARTA021564	Shroom4	0.001518185	2.189238	up
ASMM9PARTA028059	Gstm6	0.00000522	3.972863	up
ASMM9PARTA027522	Fst	0.001207298	3.5836024	up
ASMM9PARTA021642	Hsf5	0.002550301	2.1091907	up
ASMM9PARTA022725	Kng2	0.0000492	4.140855	up
ASMM9PARTA028723	Pou3f2	0.000556	2.2296135	up
ASMM9PARTA029984	Flt3	0.000338	2.029359	up
ASMM9PARTA031617	Adrb3	0.0000642	2.5341303	up
ASMM9PARTA027085	H2-Q9	0.0000617	2.3414183	up
ASMM9PARTA021097	Trpm3	0.002624987	3.5190523	up
ASMM9PARTA039476	Tm4sf4	0.000168	687.16095	up
ASMM9PARTA039253	Aldob	0.036676973	4.116622	up
ASMM9PARTA038680	Errfi1	0.044672493	3.0001395	up
ASMM9PARTA025695	R3hdm2	0.019590486	2.4934993	up
ASMM9PARTA034324	Metap1d	0.0000396	3.1245039	up
ASMM9PARTA031792	Ar	0.043859076	2.5082378	up
ASMM9PARTA022331	1110020G09Rik	0.0000636	2.6740084	up
ASMM9PARTA039655	Cald1	0.001165898	3.7672837	up
ASMM9PARTA019635	Whamm	0.0000117	3.1854694	up
ASMM9PARTA032279	Timm23	0.002484317	2.1506739	up
ASMM9PARTA022946	Mpzl3	0.005419126	2.5725136	up

ASMM9PARTA035507	Otop3	0.002396526	2.7736433	up
ASMM9PARTA031008	Procr	0.007059929	8.744379	up
ASMM9PARTA041619	F11r	0.0000247	2.3263345	up
ASMM9PARTA024171	Homer3	0.0000305	2.0901227	up
ASMM9PARTA041488	Ugt2b34	0.010387068	2.4133658	up
ASMM9PARTA034391	1700011A15Rik	0.00779953	3.1635954	up
ASMM9PARTA031689	Gfpt1	0.010807246	3.7557623	up
ASMM9PARTA031518	Spnb1	0.002384956	2.6896532	up
ASMM9PARTA030892	Vill	0.002116629	2.0621455	up
ASMM9PARTA025868	Arhgap39	0.000172	2.7099278	up
ASMM9PARTA042206	Stk36	0.000547	3.4348116	up
ASMM9PARTA041997	Slc31a1	0.002809444	2.05081	up
ASMM9PARTA030907	Rgs16	0.003411418	2.0144656	up
ASMM9PARTA036949	Lonrf3	0.003271336	2.037203	up
ASMM9PARTA032904	Ikbke	0.0311579	3.4544601	up
ASMM9PARTA029790	Atf2	0.001007943	2.209244	up
ASMM9PARTA036225	Fam75d3	0.0330409	2.1716456	up
ASMM9PARTA037234	Pcdh1	0.026136031	2.3191028	up
ASMM9PARTA028708	Snap23	0.000199	2.0343738	up
ASMM9PARTA025656	Dcaf17	0.0000296	2.4647758	up
ASMM9PARTA037112	Rpl18a	0.0000166	3.3978186	up
ASMM9PARTA028727	Psen1	0.000303	2.2164915	up
ASMM9PARTA027887	Hadh	0.000165	2.0298033	up
ASMM9PARTA027419	Agt	0.0000601	2.1952047	up
ASMM9PARTA037652	Nfkbiz	0.010048649	2.466104	up
ASMM9PARTA029936	Cyp2d9	0.031257126	2.7189524	up
ASMM9PARTA027054	Spp1	0.000561	3.3399868	up
ASMM9PARTA022841	Tmem235	0.0000452	2.6219156	up
ASMM9PARTA020417	Gm5591	0.00000399	4.1472974	up
ASMM9PARTA027759	Cyp3a16	0.016960327	3.7645054	up
ASMM9PARTA023878	Usp51	0.0006	2.1780007	up
ASMM9PARTA027122	Afp	0.038003333	15.9037895	up
ASMM9PARTA030638	Apcs	0.008810872	12.0482855	up
ASMM9PARTA023026	Kng2	0.001326915	2.5115268	up
ASMM9PARTA030003	Hfe	0.006667319	2.0103436	up
ASMM9PARTA023882	Cfb	0.00033	9.183315	up
ASMM9PARTA032069	Gpc3	0.000624	5.140846	up
ASMM9PARTA037250	1600029D21Rik	0.002240232	22.936808	up
ASMM9PARTA027411	Csn3	0.00000257	7.2258677	up
ASMM9PARTA036396	F11	0.025590647	2.6305017	up
ASMM9PARTA027534	Slc7a2	0.005787695	8.339105	up
ASMM9PARTA034257	Chmp4c	0.00062	16.92601	up
ASMM9PARTA022005	Flnc	0.011085186	2.4486353	up
ASMM9PARTA024486	Nkx2-1	0.0000198	2.621146	up
ASMM9PARTA034100	Pcbd1	0.002003013	2.088299	up
ASMM9PARTA029514	Cd8b1	0.000255	2.1830075	up
ASMM9PARTA029838	Cyp2d10	0.000208	19.47933	up
ASMM9PARTA039606	Fcrla	0.04281942	2.247378	up
ASMM9PARTA024433	Ccdc48	0.000237	2.2192373	up
ASMM9PARTA040876	Olfr550	0.001201631	2.763322	up
ASMM9PARTA033437	Il21	0.029619403	2.6832252	up
ASMM9PARTA034429	Cyb5b	0.000114	2.2067766	up
ASMM9PARTA028611	Cxcl9	0.014023554	3.9382093	up
ASMM9PARTA033390	Rqcd1	0.005982852	4.5682845	up
ASMM9PARTA033704	Sync	0.003430105	3.6855893	up
ASMM9PARTA039024	Atp12a	0.047899127	2.889323	up
ASMM9PARTA031529	Zfp59	0.037735634	2.4037533	up

ASMM9PARTA035126	Srek1ip1	0.000342	2.6132863	up
ASMM9PARTA037334	Mccc2	0.0000311	5.0420737	up
ASMM9PARTA029737	Dlx5	0.0000601	2.6381536	up
ASMM9PARTA028744	Serpina1c	0.0000543	7.8481054	up
ASMM9PARTA030734	Ccl12	0.00000878	2.3583927	up
ASMM9PARTA037934	Cpn1	0.000176	6.4089227	up
ASMM9PARTA027191	Plin2	0.000143	4.8682632	up
ASMM9PARTA030825	Orm2	0.0000256	23.21154	up
ASMM9PARTA031288	Map3k1	0.033357356	3.0881505	up
ASMM9PARTA032256	Chst2	7.06E-08	4.2964516	up
ASMM9PARTA040891	Rnf183	0.032298982	2.6582546	up
ASMM9PARTA042023	Pon3	0.000197	3.0224338	up
ASMM9PARTA029891	H2-D1	0.000545	3.2681434	up
ASMM9PARTA032787	Fbp1	0.03642512	5.903397	up
ASMM9PARTA031329	Nt5e	0.028728137	2.2291462	up
ASMM9PARTA027587	Efnb3	0.000045	2.0903556	up
ASMM9PARTA037256	Spcs3	0.001843464	2.2931519	up
ASMM9PARTA021622	Hdhd2	0.000244	2.0585213	up
ASMM9PARTA024887	Ccdc90b	0.0000674	18.06837	up
ASMM9PARTA021373	Akap2	0.000276	2.1533675	up
ASMM9PARTA037997	Steap4	0.047949597	2.9620726	up
ASMM9PARTA043478	Mapkapk3	0.008205338	2.4605498	up
ASMM9PARTA044234	Fam25c	0.000649	13.791984	up
ASMM9PARTA021491	Edem3	0.040823113	2.038919	up
ASMM9PARTA041614	Itpk1	0.0000997	2.5102594	up
ASMM9PARTA040339	Olfr196	0.0369579	2.487124	up
ASMM9PARTA023587	Schip1	0.00197141	3.480949	up
ASMM9PARTA024383	Fam5c	0.04707206	3.1735277	up
ASMM9PARTA025288	Rtdr1	0.001840493	2.9028354	up
ASMM9PARTA020524	Ate1	0.006402261	2.2398763	up
ASMM9PARTA027255	Ambp	0.0000111	17.787172	up
ASMM9PARTA025569	5-Mar	0.000245	3.5646636	up
ASMM9PARTA027581	Cp	0.0000168	3.564718	up
ASMM9PARTA027428	Anxa2	0.001247452	5.3888893	up
ASMM9PARTA043134	Serpina7	0.004401589	13.657775	up
ASMM9PARTA035767	Zfp715	0.004082636	2.024345	up
ASMM9PARTA043312	Apol10b	0.015021765	3.599081	up
ASMM9PARTA043420	Hbp1	8.71E-08	2.706979	up
ASMM9PARTA022182	Mup6	0.00000886	26.435205	up
ASMM9PARTA026350	Rreb1	0.000111	2.0152	up
ASMM9PARTA037995	Clec2h	0.003319784	2.6663601	up
ASMM9PARTA029223	Apoa1	0.00000898	24.06658	up
ASMM9PARTA044144	Pou2f1	0.004604596	2.1384192	up
ASMM9PARTA042763	Ankar	0.0000578	2.013105	up
ASMM9PARTA028178	Cd180	0.000215	6.608197	up
ASMM9PARTA040966	Cxcl17	0.006841142	3.6941843	up
ASMM9PARTA043668	Edc4	0.00000815	2.2583656	up
ASMM9PARTA041135	Pilra	0.001711208	2.1585486	up
ASMM9PARTA044321	Gm5148	0.000824	2.5767224	up
ASMM9PARTA032223	Hpx	0.00000117	53.584538	up
ASMM9PARTA024911	Mup1	0.0000029	3.0849967	up
ASMM9PARTA032294	Gnpnat1	0.002818301	2.1584404	up
ASMM9PARTA021695	Glyctk	0.031817373	2.1775353	up
ASMM9PARTA037407	2310079F23Rik	0.001868273	2.1136265	up
CUST_268_PI426409190	Gm13304	5.44E-08	24.152235	up
ASMM9PARTA032749	Apom	0.000485	12.258493	up
ASMM9PARTA042579	Tmem169	0.002541478	2.9339955	up

ASMM9PARTA027544	Cd36	0.000634	2.5803492	up
ASMM9PARTA025984	Hmga1	0.001654858	2.4464333	up
ASMM9PARTA043176	Klhl23	0.002474109	2.0706751	up
ASMM9PARTA039314	Serpina10	0.003007356	5.7853756	up
ASMM9PARTA028891	Hnf1a	0.000671	9.203837	up
ASMM9PARTA041532	Snx8	0.0000686	2.0165017	up
ASMM9PARTA028099	Lrig1	0.018317364	2.1021	up
ASMM9PARTA026253	Gm7120	0.000000739	7.5769663	up
ASMM9PARTA027785	Cxcl1	0.015482241	8.986367	up
ASMM9PARTA026661	Gm14326	0.015607159	2.0226023	up
ASMM9PARTA037052	Mau2	0.0000168	2.4766018	up
ASMM9PARTA021180	Ceacam1	0.008224509	3.290531	up
ASMM9PARTA027498	Cd63	0.000128	2.9222653	up
ASMM9PARTA035554	C8g	0.003914604	2.1211708	up
ASMM9PARTA038624	Rpp25	0.000175	2.132115	up
ASMM9PARTA041452	Slain2	0.000889	2.1441817	up
ASMM9PARTA035965	Caskin1	0.025584867	2.8046715	up
ASMM9PARTA025907	Phc3	0.03603932	3.0776289	up
ASMM9PARTA028313	Igfals	0.002827816	10.235139	up
ASMM9PARTA021630	Sytl2	0.00118439	2.2405682	up
ASMM9PARTA024037	Gm765	0.0000164	2.1508787	up
ASMM9PARTA029833	Ctsl	0.0000281	3.1652515	up
ASMM9PARTA042512	9230105E10Rik	0.00000471	4.2053246	up
ASMM9PARTA023458	Igf1	0.00091	3.517021	up
ASMM9PARTA022678	Prrt4	0.017552514	2.3765056	up
ASMM9PARTA019633	Dnmt3b	0.036239456	2.844782	up
ASMM9PARTA030386	Igf1	0.00014	5.502942	up
ASMM9PARTA022707	Krtap9-5	0.003763374	3.8013742	up
ASMM9PARTA030244	Gsr	0.000236	3.698937	up
ASMM9PARTA030760	Prl	0.0000171	2.1874514	up
ASMM9PARTA022155	Tmpo	0.001669535	2.4939332	up
ASMM9PARTA036875	Abcb8	0.039731324	2.4103553	up
ASMM9PARTA021527	Peg10	0.0000274	2.0524564	up
ASMM9PARTA024659	Irf9	0.00309557	2.384155	up
ASMM9PARTA043415	Prpf39	0.00000306	4.11868	up
ASMM9PARTA025500	Rnf41	0.002577583	2.1178644	up
ASMM9PARTA020382	Pcnp	0.000307	2.0917192	up
ASMM9PARTA040116	Olfr694	0.044646926	2.5641098	up
ASMM9PARTA034887	Dnajc19	0.0000341	2.0957391	up
ASMM9PARTA032070	S100a11	0.000862	2.1032557	up
ASMM9PARTA024537	Cd36	0.000211	3.6749263	up
ASMM9PARTA020156	Pde1a	0.0000632	2.2359629	up
ASMM9PARTA021444	Stk35	0.000122	2.4849546	up
ASMM9PARTA041600	Vps41	0.000227	2.1319923	up
ASMM9PARTA025980	Hnrnpf	0.04025108	2.8693454	up
CUST_241_PI426409190	Ssty2	0.000249	3.71356	up
ASMM9PARTA042538	Grik4	0.012636185	2.6928651	up
ASMM9PARTA031447	Psmb9	0.0000563	2.0374448	up
ASMM9PARTA020624	Gal3st3	0.000508	5.6365447	up
ASMM9PARTA043082	Cdkl2	0.0000666	3.13522	up
ASMM9PARTA036776	Tmc1	0.005640278	3.0431974	up
ASMM9PARTA025115	Ctxn2	0.000497	2.003892	up
ASMM9PARTA021401	Sco1	0.00970117	2.8475747	up
ASMM9PARTA028854	Serpina1b	0.0000607	12.649457	up
ASMM9PARTA026748	Igsf5	0.001061872	9.769573	up
ASMM9PARTA021463	Krt40	0.006774337	2.318333	up
ASMM9PARTA038940	Rbm28	0.00000617	2.4362783	up

ASMM9PARTA038580	Dst	0.029456314	2.5313416	up
ASMM9PARTA021014	Zfc3h1	0.012504224	2.00228	up
ASMM9PARTA030642	Marco	0.000066	16.976564	up
ASMM9PARTA028615	Mup5	0.0000247	6.272828	up
ASMM9PARTA021230	Gm98	0.007039565	2.4478495	up
ASMM9PARTA026155	Tcirg1	0.0000475	2.1868963	up
ASMM9PARTA041970	Slc25a24	0.0000115	2.83283	up
ASMM9PARTA023544	Rbfox2	0.005439616	3.4373653	up
ASMM9PARTA043938	Tanc2	0.012043234	2.2852807	up
ASMM9PARTA021070	Lipt1	0.012651081	2.350895	up
ASMM9PARTA024527	Cep97	0.027278522	2.0036643	up
ASMM9PARTA034143	Fam134b	0.029609755	2.9723966	up
ASMM9PARTA041954	Rln3	0.003609926	2.5326767	up
ASMM9PARTA033694	Nup98	0.011752876	2.6012425	up
ASMM9PARTA022049	Prdm13	0.00000334	4.24886	up
ASMM9PARTA028599	Raet1c	0.000166	5.084378	up
ASMM9PARTA031227	Slc7a11	0.000507	2.1157584	up
ASMM9PARTA033087	Cpb2	0.0000626	278.82602	up
ASMM9PARTA037145	Gsc2	0.03481157	2.2349374	up
ASMM9PARTA036517	Vgll3	0.001370002	2.1974564	up
ASMM9PARTA031919	Atp2a3	0.002671693	2.3173857	up
ASMM9PARTA029618	Cd2ap	0.005638016	2.4518116	up
ASMM9PARTA044388	Hjurp	0.0000229	2.2545362	up
ASMM9PARTA027386	Pzp	0.001538771	8.373349	up
ASMM9PARTA031110	Ywhaz	0.000000437	3.3895173	up
CUST_305_PI426409190	Ccl28	0.000000213	8.759215	up
ASMM9PARTA043745	Ccdc83	0.035488527	2.639819	up
ASMM9PARTA040825	Olfr109	0.022216693	4.8845677	up
ASMM9PARTA036313	Cpne3	0.000366	2.4124289	up
ASMM9PARTA027898	Klra5	0.000232	2.7668018	up
ASMM9PARTA041859	Larp4b	0.009818823	2.1518023	up
ASMM9PARTA030185	Ly6d	0.007449973	7.8545566	up
ASMM9PARTA037143	Klhl24	0.027897537	2.24306	up
ASMM9PARTA030432	Lmx1b	0.01476521	2.1249783	up
ASMM9PARTA038161	Rbfox2	0.03709465	2.0636907	up
ASMM9PARTA038245	Lpo	0.002868947	2.379003	up
ASMM9PARTA025140	Rrp1b	0.009842187	2.2809372	up
ASMM9PARTA033455	Pard6b	0.008547243	2.0140612	up
ASMM9PARTA037729	Apobec1	0.003064843	5.4989486	up
ASMM9PARTA023816	H2-Gs10	0.0000301	2.0221434	up
ASMM9PARTA031745	Fbxo6	0.010012584	2.7544951	up
ASMM9PARTA028203	Hpgd	0.002606208	2.032682	up
ASMM9PARTA025253	1700016H13Rik	0.002268186	2.1239724	up
ASMM9PARTA029884	Gata6	0.002283217	12.734244	up
ASMM9PARTA037840	Akap12	0.00000519	5.0415626	up
CUST_243_PI426409190	B020031M17Rik	0.0000136	3.8238912	up
ASMM9PARTA042096	H6pd	0.00093	5.7167263	up
ASMM9PARTA033540	Wbp11	0.0000415	2.2315981	up
ASMM9PARTA038889	Rbm47	0.02987905	2.7269614	up
ASMM9PARTA029961	Itih2	0.002951558	5.789016	up
ASMM9PARTA042120	Frmd3	0.000175	2.8166726	up
ASMM9PARTA030794	Ccl21a	7.38E-08	21.355742	up
ASMM9PARTA031691	Gzmb	0.031188648	2.174022	up
ASMM9PARTA036488	Crct1	0.001768091	16.470968	up
ASMM9PARTA036961	1700001C19Rik	0.000661	2.587373	up
ASMM9PARTA038935	Ehd4	0.003135653	3.1974306	up
ASMM9PARTA035168	Agpat2	0.0000843	2.6123466	up

ASMM9PARTA037836	F13b	0.0000455	3.2635543	up
ASMM9PARTA029721	Bst1	0.013171109	2.570811	up
ASMM9PARTA021982	Gpr155	0.005910656	3.7375243	up
ASMM9PARTA029029	Serpina1e	0.0000239	2.6646607	up
ASMM9PARTA027340	Cfi	0.000276	2.6662219	up
ASMM9PARTA029478	Runx2	0.005437158	2.1712294	up
ASMM9PARTA031132	Cd70	0.005175323	2.153527	up
ASMM9PARTA020973	Skil	0.03163705	2.6564832	up
ASMM9PARTA041397	Cdkn2aip	0.000844	2.015926	up
ASMM9PARTA028102	Kcnc3	0.001163963	2.1579874	up
ASMM9PARTA028356	Impact	0.000939	2.204607	up
ASMM9PARTA033903	Cblc	0.000282	12.427941	up
ASMM9PARTA040851	Nmur2	0.0000694	2.631134	up
ASMM9PARTA029246	Sprr1a	0.00000525	7.3599496	up
ASMM9PARTA038930	Vmn1r231	0.001137159	2.4766347	up
ASMM9PARTA038354	Pawr	0.0000561	3.0350862	up
ASMM9PARTA023176	Cacna2d1	0.002817812	2.2370334	up
ASMM9PARTA024808	Ttc15	5.77E-10	160.13786	up
ASMM9PARTA022896	Kng1	0.0000304	17.701557	up
ASMM9PARTA023802	LOC100048885	0.0000176	11.626115	up
ASMM9PARTA036499	Ugt2a3	0.002001194	5.840185	up
ASMM9PARTA025859	Frem3	0.000902	4.157524	up
ASMM9PARTA025234	Psg18	0.000667	2.426835	up
ASMM9PARTA039500	Hnrnpul1	0.019008452	3.767613	up
ASMM9PARTA029472	Alb	0.00000482	16.24476	up
ASMM9PARTA025786	Vmn1r39	0.009413214	2.614772	up
ASMM9PARTA021040	Trpm3	0.000545	34.841045	up
ASMM9PARTA029136	Svs5	0.000735	2.8063147	up
ASMM9PARTA042786	Mrgprb2	0.000723	4.66047	up
ASMM9PARTA039742	Hmx2	0.002073214	3.567825	up
ASMM9PARTA030647	2-Sep	0.02096455	2.5368752	up
ASMM9PARTA038515	Cyp4v3	0.02671918	2.1643274	up
ASMM9PARTA035481	Tlcd2	0.003430681	2.745155	up
ASMM9PARTA044237	Tspan15	0.0000273	3.978303	up
ASMM9PARTA027983	Akr1b8	0.000587	2.9254284	up
ASMM9PARTA039743	Soat2	0.003598295	2.1649642	up
ASMM9PARTA037352	Bicd2	0.004271484	2.3764355	up
ASMM9PARTA038017	Aff4	0.036303855	2.5694952	up
ASMM9PARTA032278	Ybx2	0.006259136	2.138803	up
ASMM9PARTA031043	Wdr1	0.000161	3.7481172	up
ASMM9PARTA027656	Cryga	0.000254	2.5153656	up
ASMM9PARTA043624	Siglech	0.00000739	2.0416312	up
ASMM9PARTA034441	4933411K16Rik	0.007996665	2.0351696	up
ASMM9PARTA034262	Srsf9	0.000311	2.7478414	up
ASMM9PARTA030830	Pcsk6	0.008051564	2.927615	up
ASMM9PARTA027908	Ctla2b	0.006914449	2.3779097	up
ASMM9PARTA027033	Kcnmb3	0.013990067	2.9422908	up
ASMM9PARTA044550	Rasgrp3	0.027272016	2.5414963	up
ASMM9PARTA025542	Nsun6	0.00000941	3.2402625	up
ASMM9PARTA031087	Serpinf1	0.0000176	3.9197984	up
ASMM9PARTA042748	Zfp367	0.000221	2.131845	up
ASMM9PARTA026912	Bmp8b	0.000354	5.935956	up
ASMM9PARTA028483	Pomc	0.000488	2.472528	up
ASMM9PARTA029559	Cldn4	0.00615511	16.009712	up
ASMM9PARTA033412	Lrp10	0.0000893	2.3119104	up
ASMM9PARTA029114	Phlda2	0.007752576	2.7411995	up
ASMM9PARTA031492	Creg1	0.001135305	2.5291033	up

ASMM9PARTA029385	Tuba1c	0.013628778	3.1992228	up
ASMM9PARTA030216	Hemt1	0.000238	4.223135	up
ASMM9PARTA044740	A530032D15Rik	0.000704	2.4376726	up
ASMM9PARTA041728	Lnpep	0.010949393	2.5059986	up
ASMM9PARTA032336	Hgfac	0.00000172	40.631905	up
ASMM9PARTA033295	Gmfg	0.005694846	2.247543	up
ASMM9PARTA020325	Olfr288	0.0000361	4.521481	up
ASMM9PARTA033515	H2-Q8	0.000229	5.4798155	up
ASMM9PARTA037842	Bglap-rs1	0.000291	2.2649064	up
ASMM9PARTA039455	Galnt7	0.000368	3.3095207	up
ASMM9PARTA039696	Stap2	0.000221	3.4181423	up
ASMM9PARTA036465	Cda	0.000000168	2.100513	up
ASMM9PARTA032473	Lgals8	0.03650354	2.1091764	up
ASMM9PARTA037972	Gpr84	0.0000824	2.4234054	up
ASMM9PARTA044719	Cxcl3	0.004220351	14.844567	up
ASMM9PARTA038294	Edaradd	0.000585	3.18391	up
ASMM9PARTA027011	Mup15	0.0000247	3.8392856	up
ASMM9PARTA042368	Ccdc88a	0.000924	2.525976	up
ASMM9PARTA034174	Cks2	0.00848776	3.1781802	up
ASMM9PARTA028364	Gzmm	0.01956766	2.566282	up
ASMM9PARTA027778	Fzd8	0.001038831	2.8540072	up
ASMM9PARTA019738	Rhox8	0.006942352	2.4696825	up
ASMM9PARTA021003	Trpm3	0.0000333	15.922824	up
ASMM9PARTA041572	A730008H23Rik	0.00034	3.3139248	up
ASMM9PARTA030554	Nnmt	0.000104	12.062769	up
ASMM9PARTA027525	Gcgr	0.027601104	2.6900043	up
ASMM9PARTA020765	BC026782	0.001747812	2.427853	up
ASMM9PARTA042620	Esrp2	0.0000256	4.0884886	up
ASMM9PARTA039331	Gpx6	0.000272	2.9223735	up
ASMM9PARTA028997	Tff1	0.0000742	8.1746235	up
ASMM9PARTA029555	Cdk6	0.021065174	6.506502	up
ASMM9PARTA024293	Nr6a1	0.033313368	2.6152036	up
ASMM9PARTA029942	F2	0.00000138	15.673193	up
ASMM9PARTA022450	0610010O12Rik	0.001779541	2.0968406	up
ASMM9PARTA028119	Orm1	0.00000196	113.098274	up
ASMM9PARTA021399	Rhox4d	0.00000879	2.050915	up
ASMM9PARTA028672	Raet1a	0.001972397	3.3672519	up
ASMM9PARTA030693	Plaur	0.020061687	2.7258368	up
ASMM9PARTA025875	Slc38a10	0.000048	2.1809382	up
ASMM9PARTA038789	Stard4	0.001689114	2.138523	up
ASMM9PARTA034146	Krtap3-3	0.024097081	2.319153	up
ASMM9PARTA043245	Gm14461	0.031990055	2.5192304	up
ASMM9PARTA030967	Thoc4	0.000000611	2.2027664	up
ASMM9PARTA032400	Ddx21	0.015749132	2.2053607	up
ASMM9PARTA037852	Loxl2	0.001140768	14.573694	up
ASMM9PARTA030813	Stat5b	0.001490296	2.526246	up
ASMM9PARTA034762	Ceacam14	0.008290645	2.357579	up
ASMM9PARTA038095	Glrx	0.00000117	2.4497848	up
ASMM9PARTA023707	Apobec1	0.00195491	3.3674955	up
ASMM9PARTA034935	2010011I20Rik	0.000356	2.3976474	up
ASMM9PARTA026261	Ankrd55	0.0000171	3.3367715	up
ASMM9PARTA020206	Cdhr3	0.001257422	3.440596	up
ASMM9PARTA034294	Slc16a9	0.00016	4.3543863	up
ASMM9PARTA039612	Duoxa1	0.001844626	2.374134	up
ASMM9PARTA030022	H2-Q10	0.004063776	2.001443	up
ASMM9PARTA020828	Cpxcr1	0.01748431	2.6426196	up
ASMM9PARTA043087	Igsf1	0.047533356	2.4877734	up

ASMM9PARTA034026	Trim12a	0.000145	2.1414542	up
ASMM9PARTA019664	BC057022	0.000014	2.137938	up
ASMM9PARTA023147	Snx12	0.0000581	2.3290365	up
ASMM9PARTA023267	Aqp8	0.010882038	4.8572707	up
ASMM9PARTA025627	Vmn1r168	0.000123	3.3357494	up
ASMM9PARTA027216	Trp53inp1	0.02127502	5.8145533	up
ASMM9PARTA030745	Serpina3k	0.000191	16.174522	up
ASMM9PARTA027319	Nr0b1	0.002982293	2.3163462	up
ASMM9PARTA026365	Arpc4	0.001644487	2.3961735	up
ASMM9PARTA043807	6030498E09Rik	0.027906979	2.1940942	up
ASMM9PARTA025384	Puf60	0.00000241	2.672495	up
ASMM9PARTA037434	Rpe65	0.00036	2.299696	up
ASMM9PARTA044790	Vmn1r177	0.00098	5.449634	up
ASMM9PARTA030329	Zbtb7a	0.0000576	2.0259032	up
ASMM9PARTA041502	Snx30	0.002051058	2.5296726	up
ASMM9PARTA030475	Nfkbil1	0.002694815	2.279042	up
ASMM9PARTA033840	Pla2g12b	0.006538657	4.1356807	up
ASMM9PARTA023730	Atp13a3	0.000125	6.806229	up
ASMM9PARTA032853	Fam48a	0.048817344	6.4496555	up
ASMM9PARTA041379	Cpne2	0.002825001	2.0892766	up
ASMM9PARTA028173	Serpina3c	0.001344137	2.8078647	up
ASMM9PARTA031158	Saa3	5.79E-08	414.05643	up
ASMM9PARTA034766	Lipt2	0.000871	2.0137596	up
ASMM9PARTA027739	F9	0.003993888	8.055491	up
ASMM9PARTA031384	Zfp9	0.006402783	2.2850375	up
ASMM9PARTA032047	Tnfrsf12a	0.0000175	5.7876096	up
ASMM9PARTA021422	Sel1l	0.00000143	6.379078	up
ASMM9PARTA041081	Olfr711	0.011204711	3.0466328	up
ASMM9PARTA026501	Magi2	0.010340264	2.3900032	up
ASMM9PARTA035464	Srrm4	0.0000683	2.5102985	up
ASMM9PARTA039467	Slc44a3	0.00103898	3.9473743	up
ASMM9PARTA029782	Dffa	0.0000269	2.1218286	up
ASMM9PARTA023696	Mup9	0.002020386	6.029093	up
ASMM9PARTA029581	Cga	0.0000947	3.5618737	up
ASMM9PARTA037740	Slco2a1	0.00000761	8.408813	up
ASMM9PARTA038658	Chpf2	0.016918479	2.398154	up
ASMM9PARTA042044	Sc5d	0.00000077	6.154465	up
ASMM9PARTA022193	Gm6484	0.005445675	4.9623146	up
ASMM9PARTA027261	C4bp	0.000049	11.261244	up
ASMM9PARTA040198	Actr2	0.001118875	2.373445	up
ASMM9PARTA027362	Apoa4	0.00002	41.79531	up
ASMM9PARTA028386	Hsp90ab1	0.0000168	3.3226452	up
ASMM9PARTA024967	Ces2e	0.00000226	8.396423	up
ASMM9PARTA032331	Rplp1	0.0000597	2.2746043	up
ASMM9PARTA032428	Itih4	0.004918584	2.2723079	up
ASMM9PARTA037288	Ocel1	0.00017	3.6030266	up
ASMM9PARTA027851	Mst1	0.00021	9.569627	up
ASMM9PARTA031235	Slc10a2	0.019884001	3.6096091	up
ASMM9PARTA037984	E2f6	0.0000319	2.7200866	up
ASMM9PARTA022138	Adamts14	0.000305	2.076006	up
ASMM9PARTA030591	Plunc	0.00000875	29.511639	up
ASMM9PARTA026575	Gm7849	0.001405813	3.4973295	up
ASMM9PARTA024458	Casz1	0.000623	3.6816106	up
ASMM9PARTA031164	Slfn2	0.000862	2.1003532	up
ASMM9PARTA033950	Rab27a	0.000398	2.8668993	up
ASMM9PARTA031317	Csda	0.005746611	2.2715404	up
ASMM9PARTA030460	Uhmk1	0.003656815	2.0136213	up

ASMM9PARTA026421	Plch1	0.001441778	2.107422	up
ASMM9PARTA035005	Serpina12	0.002748228	4.7023416	up
ASMM9PARTA034001	Tnfrsf23	0.003493739	3.771012	up
ASMM9PARTA030637	Saa2	0.00000458	28.281317	up
ASMM9PARTA031579	Apoa2	0.001091969	2.5937545	up
ASMM9PARTA021979	Repin1	0.04352855	2.046069	up
ASMM9PARTA030104	Hmox1	0.000665	2.5138047	up
ASMM9PARTA030950	Rps7	0.000113	2.2714653	up
ASMM9PARTA031533	Gdf15	0.020617852	2.7145615	up
ASMM9PARTA035631	Snrpf	0.0000829	2.1035519	up
ASMM9PARTA031300	Lyz1	0.0000213	2.1884444	up
ASMM9PARTA026178	Rbms3	0.000689	2.3543537	up
ASMM9PARTA027749	Gh	0.000000589	8.723904	up
ASMM9PARTA026204	Wdr33	0.003226724	7.2382317	up
ASMM9PARTA043014	Lamp3	0.00095	3.015213	up
ASMM9PARTA028812	Serpina1d	0.00000629	9.349753	up
ASMM9PARTA031026	Spink4	0.006535773	2.481017	up
ASMM9PARTA022749	Pnmal2	0.016980287	2.04741	up
ASMM9PARTA038894	Ces3b	0.027511071	2.1117733	up
ASMM9PARTA028931	Spp1	0.000742	3.3085916	up
ASMM9PARTA025573	Arhgap8	0.000123	2.0207765	up
ASMM9PARTA030350	Foxa2	0.0232851	2.0586498	up
ASMM9PARTA023632	Qser1	0.002058371	2.2529674	up
ASMM9PARTA022734	Gm11428	0.000249	5.7920837	up
ASMM9PARTA042630	Micall1	0.0000493	2.5832434	up
ASMM9PARTA037470	Pmch	1.21E-08	258.39825	up
ASMM9PARTA031956	Nox4	0.030720923	2.16569	up
ASMM9PARTA033857	Rps25	0.0000367	2.2141914	up
ASMM9PARTA037035	Lyrm7	0.0000234	2.60048	up
ASMM9PARTA031590	Abcc2	0.004844309	4.305907	up
ASMM9PARTA028696	Resp18	0.00000723	2.428444	up
ASMM9PARTA035707	4921528I01Rik	0.03904229	2.5018775	up
ASMM9PARTA024658	Birc7	0.0000357	2.0268214	up
ASMM9PARTA027754	Cd1d2	0.00575796	2.294848	up
ASMM9PARTA031905	Tcf20	0.008389055	2.2062988	up
ASMM9PARTA042837	4833442J19Rik	0.03905804	2.5352	up
ASMM9PARTA029754	Avp	2.08E-08	29.416008	up
ASMM9PARTA024541	Wdsub1	0.000081	3.6814578	up
ASMM9PARTA033277	Cxcl10	0.000228	3.2498384	up
ASMM9PARTA024004	Dedd	0.002877288	3.3849053	up
ASMM9PARTA036649	Fezf1	0.011198795	5.4864078	up
ASMM9PARTA029430	Akap2	0.0000106	2.3201954	up
ASMM9PARTA039911	Habp2	0.003987792	3.558833	up
ASMM9PARTA031558	C9	0.0000264	6.125046	up
ASMM9PARTA020625	Ttbk2	0.03833984	2.1436286	up
ASMM9PARTA030011	E2f3	0.001955219	2.407097	up
ASMM9PARTA023663	Ndufaf2	0.0000478	2.03734	up
ASMM9PARTA044240	Raet1e	0.00000734	11.305048	up
ASMM9PARTA044533	Zfp933	0.000000143	9.04388	up
ASMM9PARTA029751	Alox5	0.001309168	3.1652672	up
ASMM9PARTA025739	Vill	0.00057	4.4524846	up
ASMM9PARTA034685	Plin3	0.003518721	2.2826416	up
ASMM9PARTA037987	Bcl3	0.000916	2.3514645	up
ASMM9PARTA027409	Crp	0.0000745	4.7335916	up
ASMM9PARTA027912	Dcn	0.015172899	2.6361022	up
ASMM9PARTA042381	Gm5077	0.000641	13.011049	up
ASMM9PARTA035367	Ifi35	0.0000982	2.0831928	up

ASMM9PARTA035686	Slc38a4	0.001025338	38.25997	up
ASMM9PARTA028062	Hsd17b2	0.0000111	11.364622	up
ASMM9PARTA028133	Foxa3	0.001469045	4.6386027	up
ASMM9PARTA039433	Creb3l3	0.000402	3.0500357	up
ASMM9PARTA034319	Snw1	0.00742151	2.413834	up
ASMM9PARTA037436	Rnase6	0.0000066	2.1696317	up
ASMM9PARTA023049	Igf1	0.0000594	7.778342	up
ASMM9PARTA031796	Ccl4	0.0000137	3.4641204	up
ASMM9PARTA029518	Cldn3	0.001098972	3.028855	up
ASMM9PARTA019859	Zfp456	0.004211577	2.6347463	up
ASMM9PARTA022425	Gm5483	0.0000175	4.5535755	up
ASMM9PARTA038500	Prss8	0.002494392	2.0111601	up
ASMM9PARTA027841	Fgfr4	0.000505	3.4631393	up
ASMM9PARTA031778	Akr1c12	0.000835	3.4179604	up
ASMM9PARTA041521	D330028D13Rik	0.00222751	2.308562	up
ASMM9PARTA022286	Agmat	0.00000254	2.726005	up
ASMM9PARTA028864	Pet2	0.002817479	2.4380143	up
ASMM9PARTA038652	C8b	0.00024	2.2138696	up
ASMM9PARTA038468	Qprt	0.001100669	2.3098123	up
ASMM9PARTA039373	BC016495	0.00000407	2.8812213	up
ASMM9PARTA036363	Slc16a10	0.000609	2.0354002	up
ASMM9PARTA023692	Gm10639	0.000000879	44.005497	up
ASMM9PARTA043521	Gprc5a	0.001049031	8.000126	up
ASMM9PARTA030995	Sdc4	0.000154	2.2519653	up
ASMM9PARTA023518	Cry2	0.00915407	2.5259638	up
ASMM9PARTA037101	Pibf1	0.00300567	2.1851645	up
ASMM9PARTA042594	Hnrnpl	0.0000131	4.2074776	up
ASMM9PARTA028583	Mup2	0.000576	4.033117	up
ASMM9PARTA029115	Ugdh	0.001040847	2.4593058	up
CUST_301_PI426409190	Ccl21b	0.00000177	20.291157	up
ASMM9PARTA037372	Niacr1	0.000281	5.6197925	up
ASMM9PARTA025528	Rpa1	0.00000429	2.6643112	up
ASMM9PARTA035680	Mybphl	0.007865036	2.0384648	up
ASMM9PARTA043209	Nags	0.013987481	4.3649054	up
ASMM9PARTA032730	Ppp4c	0.00000741	2.5375283	up
ASMM9PARTA030538	Lox	0.005529475	4.8754272	up
ASMM9PARTA029410	Cat	0.005655123	2.0568285	up
ASMM9PARTA032217	Pdlim1	0.000712	3.0753212	up
ASMM9PARTA027611	Cd1d1	0.001594411	2.2484355	up
ASMM9PARTA034718	Kdelr2	0.0000384	2.010544	up
ASMM9PARTA037296	D730039F16Rik	0.00023	3.5145981	up
ASMM9PARTA029368	C4b	0.0000815	2.8149116	up
ASMM9PARTA021431	1110020G09Rik	0.0000413	2.4329476	up
ASMM9PARTA023169	Vegfa	0.000516	2.7881508	up
ASMM9PARTA031704	Sytl4	0.000106	3.8239303	up
ASMM9PARTA044394	Suz12	0.000176	2.3202207	up
ASMM9PARTA030354	Krt18	0.000258	14.124053	up
ASMM9PARTA037468	Prelid2	0.000254	3.4856331	up
ASMM9PARTA027052	Mup14	0.000066	4.0962605	up
ASMM9PARTA020162	Olfr287	0.0000283	3.4687903	up
ASMM9PARTA027812	Ces1c	0.0000665	97.91323	up
ASMM9PARTA043400	Smg5	0.014851349	3.860313	up
ASMM9PARTA028216	Lcn2	0.000097	16.299862	up
ASMM9PARTA036318	Chst13	0.018690148	2.2081044	up
ASMM9PARTA024920	Hsd17b13	0.000897	13.791368	up
ASMM9PARTA029133	Sparc	0.0000316	2.0778835	up
ASMM9PARTA026143	Gml	0.00000124	42.146217	up

ASMM9PARTA021573	Hhipl1	0.001580138	2.0586634	up
ASMM9PARTA024273	Heph	0.0000252	2.572323	up
ASMM9PARTA028041	Igfbp1	0.001003954	72.06436	up
ASMM9PARTA028676	Rbbp4	0.000139	2.2319345	up
ASMM9PARTA032433	Akr1e1	0.0000105	2.3874621	up
ASMM9PARTA022180	Dscaml1	0.00187705	4.9332957	up
ASMM9PARTA031986	Siva1	0.0000736	2.028586	up
ASMM9PARTA029285	Apoc2	0.0000168	31.691927	up
ASMM9PARTA039353	Ugt3a2	0.003386629	2.617361	up
ASMM9PARTA041493	Igsf11	0.008826348	2.3468804	up
ASMM9PARTA031072	Aadat	0.006909219	2.1991866	up
ASMM9PARTA029238	St3gal1	0.020810049	4.504363	up
ASMM9PARTA043801	Fgb	0.000113	11.481888	up
ASMM9PARTA021113	Rbfox3	0.00000991	2.0557194	up
ASMM9PARTA032825	Nmu	0.008421083	2.093813	up
ASMM9PARTA032954	Xpo4	0.023522532	2.8404458	up
ASMM9PARTA034167	Ost4	0.000143	3.1899166	up
ASMM9PARTA024373	Ralgps2	0.011027404	3.9167283	up
ASMM9PARTA027001	H2-Q7	0.0000105	4.103018	up
ASMM9PARTA027853	Hmgcr	0.000401	3.3769875	up
ASMM9PARTA022268	4930420K17Rik	0.000051	2.050795	up
ASMM9PARTA040053	Spin1	0.001593003	3.4519377	up
ASMM9PARTA024350	Lgals3	0.002844939	5.1167016	up
ASMM9PARTA039318	Zgpat	0.00330595	2.9541237	up
ASMM9PARTA028139	Itgav	0.000386	2.2432432	up
ASMM9PARTA039119	Otub1	0.0000348	3.73413	up
ASMM9PARTA032786	Cyp39a1	0.02573643	2.105585	up
ASMM9PARTA026406	Hnrnpc	0.0000217	2.020353	up
ASMM9PARTA043978	Adm2	0.02216197	2.2131064	up
ASMM9PARTA038252	Serpinc1	0.000749	2.8415508	up
ASMM9PARTA027647	Cdh2	0.00000125	2.1119945	up
ASMM9PARTA030789	Oxt	0.00000417	4.102523	up
ASMM9PARTA029858	Hcrt	9.61E-08	24.200073	up
ASMM9PARTA025515	Ces3a	0.024452582	3.798743	up
ASMM9PARTA035366	1700029I01Rik	0.000899	2.4929442	up
ASMM9PARTA033712	Gmfb	0.00000643	4.1477666	up
ASMM9PARTA020713	Treml2	0.004515789	2.0475903	up
ASMM9PARTA030741	Slpi	0.00012	2.3125417	up
ASMM9PARTA041441	Ehmt1	0.002932166	2.2254846	up
ASMM9PARTA019886	Mup21	0.000251	4.117441	up
ASMM9PARTA043954	Pla2g12a	0.0000473	2.8609784	up
ASMM9PARTA042288	Mast4	0.0000322	3.3681803	up
ASMM9PARTA031468	Ahsg	0.00000792	63.267982	up
ASMM9PARTA030019	Gast	0.005117104	2.398121	up
ASMM9PARTA029070	Tff2	0.001862454	2.9928102	up
ASMM9PARTA036814	Ascc2	0.034408186	2.1254933	up
ASMM9PARTA042803	Auts2	0.023788653	2.0385022	up
ASMM9PARTA029376	Cst7	0.0000147	28.451078	up
ASMM9PARTA036138	Gpr160	0.003222467	2.720788	up
ASMM9PARTA029329	Nr2f2	0.00000434	2.7463613	up
ASMM9PARTA027514	Epx	0.001142116	2.2969182	up
ASMM9PARTA020844	Gpr101	0.0000266	2.8552547	up
ASMM9PARTA032745	Raet1d	0.008178433	6.9452195	up
ASMM9PARTA025339	Taok2	0.0000623	2.1198497	up
ASMM9PARTA044542	Kng2	0.034501813	3.7658525	up
ASMM9PARTA032877	Rbpms	0.0000302	7.87497	up
ASMM9PARTA036324	Rpap3	0.00000501	3.1804552	up

ASMM9PARTA023991	Gtpbp2	0.000101	6.130596	up
ASMM9PARTA036806	Scpep1	0.000247	2.693094	up
ASMM9PARTA036721	Ms4a6c	0.016382767	2.2200701	up
ASMM9PARTA041892	Apol9b	0.000094	2.4350379	up
ASMM9PARTA027427	Btc	0.020730374	4.0981374	up
ASMM9PARTA035135	Rnf125	0.016935294	4.8795605	up
ASMM9PARTA032664	Pdlim5	0.006587386	2.5099127	up
ASMM9PARTA044762	Tas2r118	0.024353009	2.5062876	up
ASMM9PARTA044231	Zfp748	0.0000972	2.2528467	up
ASMM9PARTA029552	Cd14	0.000101	9.017542	up
ASMM9PARTA032848	Mgst1	0.0000472	3.7809577	up
ASMM9PARTA041996	4933427D06Rik	0.01168011	2.3441842	up
ASMM9PARTA043748	8030462N17Rik	0.004912888	2.8925185	up
ASMM9PARTA029965	Kcnk7	0.001631738	3.5135715	up
ASMM9PARTA037317	Slamf9	0.00011	2.6655169	up
ASMM9PARTA030926	Xdh	0.000151	2.9719703	up
ASMM9PARTA020141	Rsl1	0.000335	2.2151175	up
ASMM9PARTA041866	Sdad1	0.0064692	2.714591	up
ASMM9PARTA033890	4930455C21Rik	0.000816	2.0300548	up
ASMM9PARTA020403	Mup20	0.004008365	2.2794495	up
ASMM9PARTA028967	Tgif1	0.002707013	5.6542225	up
ASMM9PARTA044801	Yipf6	0.0000196	2.4330988	up
ASMM9PARTA027494	Bglap	0.000364	2.3509836	up
ASMM9PARTA035715	Xrcc6bp1	0.000033	3.8774858	up
ASMM9PARTA043449	Tmem59l	0.0000333	2.402623	up
ASMM9PARTA029947	Gal	0.000038	3.5598576	up
ASMM9PARTA034121	Rps23	0.000306	2.1048717	up
CUST_299_PI426409190	E130304F04Rik	0.00000108	3.5103202	up
ASMM9PARTA026207	Lman1	0.001720772	2.3811665	up
ASMM9PARTA023237	Lrrc61	0.00088	5.1497025	up
ASMM9PARTA033482	Ankrd36	0.027580054	2.1191888	up
ASMM9PARTA030661	Ccl9	0.007576852	2.1715953	up
ASMM9PARTA028157	Nppb	0.001280286	2.4996884	up
ASMM9PARTA031696	Lhcgr	0.011774275	2.0191789	up
ASMM9PARTA030449	Pon1	0.002029699	32.559986	up
ASMM9PARTA032243	Irf7	0.000172	3.7917817	up
ASMM9PARTA028318	Itih3	0.000185	3.1124713	up
ASMM9PARTA021768	Slc7a2	0.036042158	3.4497433	up
ASMM9PARTA039113	BC021614	0.000414	5.6115894	up
ASMM9PARTA029837	Cyp1a2	0.03180321	2.565639	up
ASMM9PARTA024728	Cacna1c	0.008767873	2.3442287	up
ASMM9PARTA036473	3110007F17Rik	0.000218	2.1131096	up
ASMM9PARTA025222	Rpap2	0.0000112	3.180098	up
ASMM9PARTA044938	H2-Q6	0.000061	2.869144	up
ASMM9PARTA031598	Schip1	0.0000199	3.878496	up
ASMM9PARTA038379	Pcdhb4	0.000295	2.6566732	up
ASMM9PARTA030879	Ccl2	0.003864357	8.909244	up
ASMM9PARTA030873	Rbp4	0.0000221	8.998395	up
ASMM9PARTA021618	Scoc	0.002892829	2.0524821	up
ASMM9PARTA032569	Il22	0.0000482	3.3923943	up
ASMM9PARTA044577	Dand5	0.009242218	2.6468854	up
ASMM9PARTA044611	Ugt1a6b	0.017883386	3.0295238	up
ASMM9PARTA039143	Csde1	0.0010392	2.1164172	up
ASMM9PARTA042986	Cyp2r1	0.000334	2.4225907	up
ASMM9PARTA025511	Zfp809	0.02032749	2.5243971	up
ASMM9PARTA040545	Olfr26	0.041912567	2.1035762	up
ASMM9PARTA021646	Tnpo1	0.00026	2.1353512	up

ASMM9PARTA039242	Zfp52	0.018240057	2.114476	up
ASMM9PARTA024523	Celf4	0.000126	31.656782	up
ASMM9PARTA039330	Ifi27l2b	0.007289839	10.862768	up
ASMM9PARTA038827	Fgg	0.000381	15.540054	up
ASMM9PARTA031375	Ankrd1	0.013789926	5.9971714	up
ASMM9PARTA035141	Rab32	0.0028951	2.463117	up
ASMM9PARTA033670	Stard3nl	0.0000391	2.0058455	up
ASMM9PARTA044835	Acsm3	0.00081	9.307004	up
ASMM9PARTA036809	4930503E14Rik	0.000135	3.0085163	up
ASMM9PARTA021869	Rbpms	0.00000108	20.253654	up
ASMM9PARTA031019	Ccl3	0.0000154	11.9296665	up
ASMM9PARTA023356	Igf1	0.0000745	4.6334696	up
ASMM9PARTA039598	Fgl1	0.000222	12.549015	up
ASMM9PARTA036186	Lrrc8e	0.006220999	3.0200787	up
ASMM9PARTA041661	Otop1	0.0000666	2.195905	up
ASMM9PARTA029202	Sntb2	0.014195433	2.4726825	up
ASMM9PARTA027996	Gpr65	0.0000536	2.5031261	up
ASMM9PARTA037349	Hyls1	0.007830146	2.352997	up
ASMM9PARTA027195	Arf5	0.0000955	2.477853	up
ASMM9PARTA030446	P2rx7	0.0000466	2.2264807	up
ASMM9PARTA033359	Bcmo1	0.0000623	5.2967863	up
ASMM9PARTA023555	Ncam1	0.0000882	2.203308	up
ASMM9PARTA039414	Defb7	0.012819974	3.9362533	up
ASMM9PARTA034233	Fabp4	0.0000552	29.398123	up
ASMM9PARTA044600	Ces3a	0.000457	4.116418	up
ASMM9PARTA024981	Mup1	0.002101019	3.4760346	up
ASMM9PARTA030989	Stat3	0.0000517	2.0114586	up
ASMM9PARTA041636	Nup214	0.000491	2.962114	up
ASMM9PARTA026835	Krtap22-2	0.009635109	2.6987276	up
ASMM9PARTA043526	Eif5	0.00013	3.4526772	up
ASMM9PARTA038214	Ftcd	0.023490246	3.7166464	up
ASMM9PARTA038525	Ghitm	0.000189	2.1687162	up
ASMM9PARTA035781	Prdm16	0.011750553	4.48461	up
ASMM9PARTA029506	Atox1	0.000344	2.4388926	up
ASMM9PARTA038540	Arhgef5	0.000204	2.697708	up
ASMM9PARTA036069	0610007L01Rik	0.00248166	3.8975668	up
ASMM9PARTA042071	Apol9a	0.003922888	2.0051203	up
ASMM9PARTA039388	Etf1	0.000165	2.2974086	up
ASMM9PARTA032311	Sult1d1	0.000936	60.817657	up
ASMM9PARTA041881	Ttc34	0.005233511	2.199763	up
ASMM9PARTA027626	Egfr	0.001027634	6.3252134	up
ASMM9PARTA031936	Shox2	0.000934	2.2560925	up
ASMM9PARTA044481	Tes	0.007768372	5.3008294	up
ASMM9PARTA025692	Mau2	0.00550533	2.2039864	up
ASMM9PARTA023995	Lgals9	0.000736	2.2455807	up
ASMM9PARTA039937	Ces2c	0.028537132	2.085048	up
ASMM9PARTA042954	Crebl2	0.005826204	2.3372114	up
ASMM9PARTA032344	Agxt	0.000277	5.1507273	up
ASMM9PARTA023341	Apoc1	0.004358233	5.8044868	up
ASMM9PARTA033471	Myl7	0.002140165	3.056162	up
ASMM9PARTA029204	Serpina1a	0.0000288	6.874818	up
ASMM9PARTA024236	Pvrl2	0.01866922	2.2335699	up
ASMM9PARTA024554	Slc25a2	0.0000234	5.084941	up
ASMM9PARTA039185	Lactb2	0.003722836	2.1924458	up
ASMM9PARTA032130	Dmp1	0.0000294	2.2168965	up
ASMM9PARTA032235	Prok2	0.001523164	2.1034334	up
ASMM9PARTA030978	Rlim	0.0002	2.029492	up

ASMM9PARTA029091	Sfrp2	0.000518	3.3584335	up
ASMM9PARTA029167	Cxcl2	0.013472946	3.3868766	up
ASMM9PARTA034601	Ece2	0.000132	3.0650053	up
ASMM9PARTA028032	Gsta2	0.0000747	49.720745	up
ASMM9PARTA030440	Nfe2l2	0.001895828	2.2326274	up
ASMM9PARTA032470	Abhd2	0.000115	7.6303153	up
CUST_291_PI426409190	Ccl21c	1.65E-08	14.897777	up
ASMM9PARTA037977	Krt8	0.0000418	9.841718	up
ASMM9PARTA039789	Cyp2c70	0.0000855	29.30503	up
ASMM9PARTA021322	Smurf1	0.003281579	2.2037911	up
ASMM9PARTA025104	Tnnt3	0.005287184	3.044461	up
ASMM9PARTA032461	Hp	0.00000257	19.80505	up
ASMM9PARTA022998	Acp5	0.000147	2.543584	up
ASMM9PARTA028833	Serpine1	0.001650085	4.822824	up
ASMM9PARTA024812	Eda2r	0.000212	3.9022021	up
ASMM9PARTA028348	Hmx3	0.005121595	2.6906996	up
ASMM9PARTA032408	Cyp11a1	0.000599	2.0628052	up
ASMM9PARTA028993	Sult2a2	0.000305	3.4232147	up
ASMM9PARTA026645	Gng12	0.000166	2.2343452	up
ASMM9PARTA023776	Nmi	0.000925	2.0037444	up
ASMM9PARTA036769	1300002K09Rik	0.002001623	19.304834	up
ASMM9PARTA031028	Sdc1	0.001504632	24.963526	up
ASMM9PARTA031711	Usp18	0.027192796	2.5585656	up
ASMM9PARTA041797	Creb5	0.009989169	3.4842727	up
ASMM9PARTA024722	Nqo2	0.000875	4.823249	up
ASMM9PARTA027686	Cyp3a11	0.04274928	3.5208766	up
ASMM9PARTA030410	Nid1	0.0000983	3.796286	up
ASMM9PARTA032281	Ly96	0.0000395	2.0528438	up
ASMM9PARTA034700	Gprasp1	0.001109777	2.0680022	up
ASMM9PARTA040038	Olfr657	0.026063085	2.6222227	up
ASMM9PARTA036556	Acot12	0.014452996	3.6061325	up
ASMM9PARTA025029	Apol6	0.0000404	2.0441713	up
ASMM9PARTA020569	Thoc2	0.025761178	2.721228	up
ASMM9PARTA042896	Ubash3b	0.00000413	2.475525	up
ASMM9PARTA034962	Zcchc10	0.0000811	2.2487483	up
ASMM9PARTA034011	Krt20	0.001222408	8.8194895	up
ASMM9PARTA023746	Enpp2	0.001136237	2.0716453	up
ASMM9PARTA029926	Gsc	0.00117492	2.3331435	up
ASMM9PARTA036538	Tmem55a	0.000676	2.198758	up
ASMM9PARTA027376	Cdkn1a	0.03258461	2.8570483	up
ASMM9PARTA029974	Dlx3	0.049765475	2.4345927	up
ASMM9PARTA022143	Nipal1	0.018504541	2.6998262	up
ASMM9PARTA019828	H2-K1	0.00000646	2.5109093	up
ASMM9PARTA029370	Cast	0.000141	2.5134616	up
ASMM9PARTA035066	Ooep	0.000144	4.3999467	up
ASMM9PARTA025377	Erlin1	0.01218445	2.676347	up
ASMM9PARTA039324	Afm	0.0000422	8.265686	up
ASMM9PARTA038869	Ces2a	0.002418158	2.2267132	up
ASMM9PARTA029030	Serpina3m	0.000336	46.34972	up
ASMM9PARTA022629	Sh3d19	0.004746981	2.4568977	up
ASMM9PARTA022848	Gm10375	0.002467378	2.443335	up
ASMM9PARTA031662	Akr1c13	0.006583128	3.8930511	up
ASMM9PARTA044004	Bst2	0.00000186	9.533976	up
ASMM9PARTA031683	Apoh	0.000127	11.041059	up
ASMM9PARTA028957	Sox11	0.000151	2.2061996	up
ASMM9PARTA033719	Apoc3	0.000439	5.953626	up
ASMM9PARTA031523	Rax	0.0000413	3.4520302	up

ASMM9PARTA037155	Sgms2	0.003885118	3.7184405	up
ASMM9PARTA042973	Slc9a4	0.016005505	0.367603676	down
ASMM9PARTA030215	Hbegf	0.000000309	0.226434441	down
ASMM9PARTA028532	Junb	0.000186	0.467343879	down
ASMM9PARTA021588	Gm13152	0.0000164	0.444869542	down
ASMM9PARTA031909	Zfp238	0.03263887	0.299774138	down
ASMM9PARTA022401	Crygc	0.000253	0.235101058	down
ASMM9PARTA021442	Gm13051	0.00000567	0.170937234	down
ASMM9PARTA032642	Scn9a	0.0000245	0.459402002	down
ASMM9PARTA023435	Gm7168	0.013747676	0.453811723	down
ASMM9PARTA028618	Ngp	0.004853929	0.480417233	down
ASMM9PARTA029903	Dgcr6	0.006847616	0.459636662	down
ASMM9PARTA039711	Mpp4	0.0000123	0.076737107	down
ASMM9PARTA024338	Zfand2b	0.046620134	0.476965022	down
ASMM9PARTA028955	Sstr2	0.0000753	0.496898163	down
ASMM9PARTA040295	Olfr1466	0.001250902	0.36796149	down
ASMM9PARTA037705	Tnfrsf25	0.000159	0.379046722	down
ASMM9PARTA024109	Mup13	0.0000816	0.314225688	down
ASMM9PARTA043059	Mkx	0.002725761	0.283742535	down
ASMM9PARTA031321	Slc30a3	2.98E-08	0.473645264	down
ASMM9PARTA024643	Islr2	0.03289369	0.419841632	down
ASMM9PARTA037509	Brd8	0.0000734	0.393042982	down
ASMM9PARTA035300	Hddc3	0.000000395	0.328512468	down
ASMM9PARTA031499	Eif2s3y	0.0000789	0.479982383	down
ASMM9PARTA043821	Tmem72	0.0000605	0.418247643	down
ASMM9PARTA023907	Mup19	0.000264	0.392114345	down
ASMM9PARTA034527	Sostdc1	0.0000476	0.326821171	down
ASMM9PARTA024076	Mup8	0.002036876	0.483978614	down
ASMM9PARTA044023	E030010A14Rik	0.000308	0.381288612	down
ASMM9PARTA026601	Dlc1	0.000217	0.464019617	down
ASMM9PARTA030353	Klk1b1	0.0000183	0.181659906	down
ASMM9PARTA043955	D630045J12Rik	0.000242	0.48816126	down
ASMM9PARTA041431	Slc24a2	0.002699476	0.426236638	down
ASMM9PARTA025882	6030458C11Rik	0.000281	0.282420217	down
ASMM9PARTA027332	Cap1	0.0000264	0.292745206	down
CUST_249_PI426409190	Gm3286	0.0000429	0.441805882	down
ASMM9PARTA042455	Gimap5	0.000491	0.373249743	down
ASMM9PARTA027962	Gjb3	0.000251	0.402663458	down
ASMM9PARTA028840	Rcvrn	0.004386	0.491746841	down
ASMM9PARTA034614	Dnase1l2	0.000748	0.495306329	down
ASMM9PARTA036237	Ubiad1	0.000531	0.236124125	down
ASMM9PARTA043880	4932414N04Rik	0.003104006	0.410123068	down
ASMM9PARTA029727	Cd19	0.021617334	0.437583141	down
ASMM9PARTA039910	Oit1	0.008046963	0.488589672	down
ASMM9PARTA032497	Fabp1	0.016139716	0.311071598	down
ASMM9PARTA042775	Myh8	0.00491546	0.305742209	down
ASMM9PARTA032377	Cldn2	0.023225222	0.491812405	down
ASMM9PARTA032653	Adar	0.023826703	0.472710338	down
ASMM9PARTA034985	Kcnv1	0.0000476	0.495992431	down
ASMM9PARTA029910	Epm2a	1.98E-08	0.051198676	down
ASMM9PARTA025621	BC005561	0.0000302	0.167559536	down
ASMM9PARTA034488	Cntnap2	0.00000822	0.249473548	down
ASMM9PARTA029152	Ywhae	0.000000343	0.342773158	down
ASMM9PARTA021788	Thg1l	0.000064	0.230841719	down
ASMM9PARTA030429	Kif11	0.0222601	0.488956263	down
ASMM9PARTA020683	Igsf9b	0.004473134	0.441796239	down
ASMM9PARTA028385	Hsd3b5	0.00000115	0.097729194	down

ASMM9PARTA031795	Odf2	0.000318	0.499939207	down
ASMM9PARTA030160	Fgf5	0.021406036	0.485728802	down
ASMM9PARTA024583	Rgs17	0.0000062	0.37330538	down
ASMM9PARTA032422	Insm1	0.0000495	0.490687849	down
ASMM9PARTA027570	Cxcr5	0.000485	0.390621735	down
ASMM9PARTA028678	Nppa	0.0000614	0.283605742	down
ASMM9PARTA025197	Kalrn	0.00000261	0.481357922	down
ASMM9PARTA029830	Chi3l3	0.000235	0.46868177	down
ASMM9PARTA032293	Lmna	0.000409	0.37848065	down
ASMM9PARTA023834	Rergl	0.002326847	0.354727543	down
ASMM9PARTA026754	Fau	0.000019	0.154699763	down
ASMM9PARTA027239	Slc29a1	0.0000173	0.483106374	down
ASMM9PARTA033099	Stap1	0.012436161	0.478582019	down
ASMM9PARTA025592	Gm14548	0.021181205	0.482440679	down
ASMM9PARTA024525	Rdh7	0.02523876	0.414652767	down
ASMM9PARTA030236	Fos	0.000172	0.48102614	down
ASMM9PARTA028736	S100a9	0.0000106	0.499931584	down
ASMM9PARTA038804	Kcne2	0.0000321	0.458549278	down
ASMM9PARTA032566	Ptprcap	0.005428916	0.493290947	down
ASMM9PARTA030315	Slc6a4	0.0000447	0.441296704	down
CUST_250_PI426409190	Gm3286	0.0000155	0.443938611	down
ASMM9PARTA029273	Tcl1	0.000000127	0.014135804	down
ASMM9PARTA031964	Tmod4	0.015848607	0.321909443	down
ASMM9PARTA029742	Egfbp2	0.00000951	0.23347499	down
ASMM9PARTA044396	Cd300lh	0.0000408	0.236074051	down
ASMM9PARTA026589	Zfp513	0.000401	0.404291032	down
ASMM9PARTA027890	Hes3	0.017761111	0.377102801	down
ASMM9PARTA032708	Rgs17	0.0000234	0.489698938	down
ASMM9PARTA032273	Crlf2	0.000473	0.441060355	down
ASMM9PARTA024544	Fam150b	0.002158269	0.445263809	down
ASMM9PARTA040613	Olfr435	0.000117	0.364702302	down
ASMM9PARTA039527	Trib1	0.00014	0.461173141	down
ASMM9PARTA029147	Ttk	0.002395154	0.47989612	down
ASMM9PARTA027936	Inadl	0.0296823	0.450680683	down
ASMM9PARTA036550	2410076I21Rik	0.000267	0.319978661	down
ASMM9PARTA036691	Als2	0.000777	0.4082203	down
ASMM9PARTA020269	Olfr1329	0.032082494	0.475689371	down
ASMM9PARTA025418	2210021J22Rik	0.000468	0.496336491	down
ASMM9PARTA028048	Fosb	0.0000213	0.454175405	down
ASMM9PARTA030020	Nr6a1	0.000109	0.429017656	down
ASMM9PARTA042802	C730048C13Rik	0.00000496	0.183598453	down
ASMM9PARTA036292	Pan3	0.0000142	0.256213944	down
ASMM9PARTA030649	Notch4	0.001635765	0.487849998	down
ASMM9PARTA029325	Adam17	0.003874467	0.432732585	down
ASMM9PARTA039758	Spon1	0.0000426	0.386575292	down
ASMM9PARTA038655	Ugt2b38	0.000769	0.345928031	down
ASMM9PARTA023296	Gm3448	0.0000154	0.260556994	down
ASMM9PARTA041022	Homer1	0.0000625	0.143984859	down
ASMM9PARTA024985	Col4a5	0.0000103	0.474040522	down
ASMM9PARTA044796	Cebpe	0.005017891	0.475794819	down
ASMM9PARTA034873	Ceacam12	0.002435127	0.30555981	down
ASMM9PARTA042225	Ltbp4	0.0002	0.491334433	down
ASMM9PARTA043957	Wbscr28	0.000149	0.461222361	down
ASMM9PARTA025787	Vmn1r176	0.007232964	0.478677892	down
ASMM9PARTA028780	Syt1	0.00000467	0.293032307	down
ASMM9PARTA029622	Clca1	0.009162277	0.486049646	down
ASMM9PARTA031272	Sf1	0.038174484	0.43577859	down

ASMM9PARTA031437	Polk	0.000238	0.353910792	down
ASMM9PARTA022650	Slc2a9	0.003816413	0.320703392	down
ASMM9PARTA038489	Celsr3	0.010112805	0.407052544	down
ASMM9PARTA039985	Zfp418	0.04104932	0.43289706	down
ASMM9PARTA032737	Acpp	0.02064879	0.419578894	down
ASMM9PARTA042570	Mylk3	0.018191954	0.483113049	down
ASMM9PARTA020931	Mageb4	0.026329242	0.48557573	down
ASMM9PARTA042028	Kcns3	0.04523851	0.226949448	down
ASMM9PARTA026146	Slc10a1	0.00000856	0.098413815	down
ASMM9PARTA024003	Psen2	0.011228817	0.455768779	down
ASMM9PARTA026587	Gm13138	0.0000857	0.461782958	down
ASMM9PARTA044243	Paqr3	0.013341891	0.412755448	down
ASMM9PARTA044201	Gadd45gip1	0.0000119	0.262386361	down
ASMM9PARTA025601	Mup11	0.000000199	0.284587279	down
ASMM9PARTA028079	Ltf	0.0000171	0.454283209	down
ASMM9PARTA040144	Olfr1457	0.001572979	0.347708616	down
ASMM9PARTA040843	Olfr978	0.000736	0.431760399	down
ASMM9PARTA037030	4930511M11Rik	0.013188097	0.388463564	down
ASMM9PARTA035535	Srpx2	0.02585578	0.430954162	down
ASMM9PARTA028641	Mc3r	0.009461132	0.294415526	down
ASMM9PARTA043423	Pacsin1	0.00000492	0.422701559	down
ASMM9PARTA038744	Fam64a	0.000263	0.443228403	down
ASMM9PARTA042825	Xylt1	0.035959613	0.468453542	down
ASMM9PARTA040381	Olfr727	0.00471029	0.342129833	down
ASMM9PARTA041786	Iqub	0.001261203	0.487423546	down
ASMM9PARTA020324	Skap1	0.000167	0.219821189	down
ASMM9PARTA031037	Try4	0.008049136	0.298372313	down
ASMM9PARTA028410	Pdha2	0.038102333	0.494383727	down
ASMM9PARTA021996	Gucy2g	0.005902305	0.46511563	down
ASMM9PARTA025940	D330028D13Rik	0.00017	0.170914832	down
ASMM9PARTA034749	Nkapl	0.000238	0.349011183	down
ASMM9PARTA021674	Bdnf	0.0000212	0.466203792	down
ASMM9PARTA025525	Nfatc1	0.011591441	0.402988823	down
ASMM9PARTA038893	Defb9	0.0000361	0.278837165	down
ASMM9PARTA019833	Try5	0.017210985	0.193596037	down
ASMM9PARTA026103	Myt1	0.004039791	0.329951441	down
ASMM9PARTA043662	Vmac	0.00845134	0.330779036	down
ASMM9PARTA025894	Vmn1r113	0.0000115	0.442193033	down
ASMM9PARTA023570	Pde7a	0.002749907	0.477669112	down
ASMM9PARTA025548	Dpy19l2	0.007144816	0.476049973	down
ASMM9PARTA024893	Med16	0.048209075	0.490917342	down
ASMM9PARTA027918	Egr1	0.00000122	0.392822088	down
ASMM9PARTA030528	Ids	0.018273843	0.485688343	down
ASMM9PARTA020329	Olfr406-ps	0.024377141	0.4317105	down
ASMM9PARTA031308	Clec3b	0.0000546	0.35358776	down
ASMM9PARTA031634	Dusp1	0.000067	0.373300196	down
ASMM9PARTA021408	Trpm3	0.00000332	0.431228591	down
ASMM9PARTA035988	Dhx34	0.008895337	0.425384207	down
ASMM9PARTA026879	Amh	0.010493986	0.408379123	down
ASMM9PARTA040559	Olfr641	0.002854717	0.37871337	down
ASMM9PARTA033543	Folr4	0.000437	0.309975665	down
ASMM9PARTA039337	Tmem189	0.0185322	0.494481341	down
ASMM9PARTA024663	Fgd2	0.002699563	0.414719799	down
ASMM9PARTA043314	Spata21	0.03825752	0.371856419	down
ASMM9PARTA025271	Barhl1	0.035410836	0.333145561	down
ASMM9PARTA040239	Olfr164	0.03436939	0.405984834	down
ASMM9PARTA030500	Klk1b11	0.000000274	0.074594361	down

ASMM9PARTA041741	Tph2	0.0000878	0.204844511	down
ASMM9PARTA027245	Mup12	0.000829	0.414763817	down
ASMM9PARTA034590	Fam187a	0.0000235	0.213284284	down
ASMM9PARTA028286	Ltb	0.006315122	0.404416667	down
ASMM9PARTA043625	Sik2	0.009175265	0.414264039	down
ASMM9PARTA025080	Sgsm1	0.0000272	0.257968053	down
ASMM9PARTA032326	Arc	0.00000108	0.179377345	down
ASMM9PARTA029274	Thrb	0.021862203	0.205487942	down
ASMM9PARTA023546	Serac1	0.008477282	0.244295994	down
ASMM9PARTA038106	Pth2	0.000109	0.415717134	down
ASMM9PARTA025385	2210021J22Rik	0.00000228	0.402087445	down
ASMM9PARTA033122	Spib	0.000379	0.208397198	down
ASMM9PARTA035171	Dusp6	0.012823841	0.497708798	down
ASMM9PARTA040340	Olfr1238	0.011584684	0.306861487	down
ASMM9PARTA033706	Rsc1a1	0.01768161	0.220316014	down
ASMM9PARTA031047	Rhag	0.00011	0.261335083	down
ASMM9PARTA023099	Gas7	0.00189725	0.3737123	down
ASMM9PARTA041362	Pus7l	0.014184736	0.370009529	down
ASMM9PARTA034470	Sys1	0.0000285	0.458461129	down
ASMM9PARTA030919	Tdgf1	0.046951074	0.346677373	down
ASMM9PARTA033257	Doc2g	0.000000408	0.491823121	down
ASMM9PARTA026741	Mrpl15	0.000169	0.395209695	down
ASMM9PARTA031999	Bhmt	0.00000935	0.135460032	down
ASMM9PARTA041276	Pxt1	0.004590172	0.381893993	down
ASMM9PARTA023691	Gm13125	0.001326092	0.141887921	down
ASMM9PARTA023419	Ccnb1ip1	0.000232	0.229183755	down
ASMM9PARTA024843	Lmo4	0.000125	0.375995203	down
ASMM9PARTA039186	Tnpo2	0.00000107	0.459237125	down
ASMM9PARTA036903	Unkl	0.00415621	0.404864856	down
ASMM9PARTA041459	Lrrc56	0.000676	0.425549498	down
ASMM9PARTA030085	Erf	0.012908177	0.45431231	down
ASMM9PARTA039317	Gdap1l1	0.000166	0.386096451	down
ASMM9PARTA042712	6530418L21Rik	0.0000627	0.433467243	down
ASMM9PARTA019871	Dnmt3b	0.000064	0.338452335	down
ASMM9PARTA029990	Gem	0.000446	0.429752413	down
ASMM9PARTA038627	Hoxb2	0.00000523	0.441047788	down
ASMM9PARTA024138	Mup7	0.00000108	0.279095473	down
ASMM9PARTA027201	Commd7	0.000222	0.469340122	down
ASMM9PARTA020035	Gm5409	0.0000329	0.467364891	down
ASMM9PARTA044724	Olfr741	0.041489735	0.385973775	down
ASMM9PARTA034885	4930549C01Rik	0.000619	0.469983405	down
ASMM9PARTA021365	Wfdc15b	0.000105	0.48202307	down
ASMM9PARTA028663	Prg2	0.00000119	0.175515555	down
ASMM9PARTA023422	Arhgef16	0.000000411	0.465175432	down
ASMM9PARTA041481	Zswim4	0.0000192	0.147287001	down
ASMM9PARTA039572	Rod1	0.037881777	0.476646354	down
ASMM9PARTA038608	Oosp1	0.002457429	0.36532236	down
ASMM9PARTA026893	Vit	0.000125	0.338283687	down
ASMM9PARTA029549	Aspm	0.04410372	0.440597733	down
ASMM9PARTA031815	Nr4a3	0.004178593	0.497477095	down
ASMM9PARTA030228	Kap	0.0000254	0.071096444	down
ASMM9PARTA026040	Ipcef1	0.0000152	0.149747754	down
ASMM9PARTA044308	Mrvi1	0.018769069	0.472869268	down
ASMM9PARTA023537	Catsper4	0.003176924	0.367400657	down
ASMM9PARTA032990	Git2	0.0000183	0.47472038	down
ASMM9PARTA027144	Mup17	0.000000146	0.302127345	down
ASMM9PARTA031089	Sema6c	0.003034416	0.49963609	down

ASMM9PARTA030958	Sorl1	0.00000452	0.149844162	down
ASMM9PARTA040880	Hrh4	0.004948984	0.360725102	down
ASMM9PARTA032874	Rbck1	0.001522778	0.352511523	down
ASMM9PARTA026257	Zfp647	0.022127716	0.417488493	down
ASMM9PARTA037033	Ccdc83	0.000321	0.338653868	down
ASMM9PARTA027080	Fbxl13	0.005876007	0.424467135	down
ASMM9PARTA032314	Rbm3	0.0000873	0.452523538	down
ASMM9PARTA035348	Iqcf3	0.001044892	0.324744839	down
ASMM9PARTA044389	Gse1	0.000585	0.318707435	down
ASMM9PARTA025799	Tmem40	0.000179	0.370807374	down
ASMM9PARTA044572	Unc93a	0.001949501	0.377923622	down
ASMM9PARTA043728	Me3	0.00000423	0.437925888	down
ASMM9PARTA022806	Gm13078	0.021160778	0.41041406	down
ASMM9PARTA023527	Corin	0.0000377	0.465840867	down
ASMM9PARTA020610	Ccdc27	0.004097562	0.443864796	down
ASMM9PARTA028405	Notch3	0.000204	0.366340261	down
ASMM9PARTA041052	Slc6a5	0.003714943	0.440113629	down
ASMM9PARTA022340	Phf21b	0.001635738	0.498560829	down
ASMM9PARTA026633	Pde4b	0.011835448	0.480360093	down
ASMM9PARTA025774	Eomes	0.013837613	0.361407465	down
ASMM9PARTA039051	Vmn1r8	0.021540051	0.413593837	down
ASMM9PARTA020811	Gm106	0.042625967	0.498456778	down
ASMM9PARTA023899	Oxr1	0.000241	0.322562103	down
ASMM9PARTA042054	Lpin1	0.02393558	0.473953753	down
ASMM9PARTA030994	Sykb	0.000127	0.270543885	down
ASMM9PARTA042717	Zfp747	0.000524	0.277571858	down
ASMM9PARTA036460	Zswim1	0.0000392	0.402493528	down
ASMM9PARTA021232	BC048644	0.014547979	0.369697658	down
ASMM9PARTA025760	Vmn1r117	0.000000968	0.434305307	down
ASMM9PARTA020142	Gm13154	0.00000223	0.371131561	down
ASMM9PARTA037075	5730419I09Rik	0.000172	0.479790919	down
ASMM9PARTA031954	Neu2	0.000487	0.428661876	down
ASMM9PARTA041560	Otop2	0.002250629	0.353663616	down
ASMM9PARTA032151	Igfbpl1	0.000809	0.44158443	down
ASMM9PARTA040861	Olfr30	0.0000707	0.418869624	down
ASMM9PARTA036161	Slc22a16	0.000857	0.334293234	down
ASMM9PARTA023305	Slc14a2	0.02637166	0.464379104	down
ASMM9PARTA019794	Zfp457	0.00017	0.217704799	down
ASMM9PARTA034514	4921530L21Rik	0.00700462	0.365511905	down
ASMM9PARTA029071	Tgm3	0.0000328	0.220615436	down
ASMM9PARTA021928	Cyp2b23	0.000348	0.392665526	down
ASMM9PARTA025900	Gpr19	0.000876	0.348488202	down
ASMM9PARTA042103	Klf8	0.004883245	0.404240074	down
ASMM9PARTA036795	Kif2b	0.000251	0.269359181	down
ASMM9PARTA040864	Olfr796	0.021858174	0.473780198	down
ASMM9PARTA040808	Leap2	0.000139	0.389914609	down
ASMM9PARTA024528	Sidt1	0.0000415	0.428355452	down
ASMM9PARTA037637	Ccdc116	0.01454149	0.491639184	down
ASMM9PARTA021137	Plac1l	0.000275	0.301403328	down
ASMM9PARTA041201	Rbm45	0.00000697	0.117660015	down
ASMM9PARTA020852	Tmem2	0.004486588	0.483824372	down
ASMM9PARTA026534	Cuedc1	0.000845	0.26893848	down
ASMM9PARTA039721	Hkdc1	0.00000898	0.44028638	down
ASMM9PARTA020245	Ear12	0.000311	0.225203603	down
ASMM9PARTA025956	Pate-n	0.0000115	0.205355183	down
ASMM9PARTA040779	Zc3h12a	0.0000387	0.346283769	down
ASMM9PARTA027022	Ins2	0.014031069	0.477579756	down

ASMM9PARTA038186	Cacng6	0.008964797	0.366543547	down
ASMM9PARTA037382	Pbp2	0.00630391	0.454935127	down
ASMM9PARTA041393	Ccnb1	0.000488	0.408993606	down
ASMM9PARTA044638	Dgkq	0.002215023	0.342088999	down
ASMM9PARTA043264	Prss35	0.00000017	0.406529977	down
ASMM9PARTA033400	Efemp2	0.009318534	0.458939298	down
ASMM9PARTA021964	Chrna9	0.04733354	0.438333395	down
ASMM9PARTA033165	Aurkc	0.036170002	0.420278682	down
ASMM9PARTA031139	Xlr3c	0.0000111	0.429040351	down
ASMM9PARTA029459	Uty	0.0000289	0.474480043	down
ASMM9PARTA036056	Ankrd33b	0.036068376	0.278879935	down
ASMM9PARTA021256	Tas2r122	0.00017	0.377055878	down
ASMM9PARTA033269	Klk1b27	0.000119	0.38153231	down
ASMM9PARTA039707	D19Wsu162e	0.016259165	0.480898796	down
ASMM9PARTA030295	Klk1	0.00000383	0.309312286	down
ASMM9PARTA027266	Cd79a	0.028763643	0.25046783	down
ASMM9PARTA033414	Retnla	0.0000285	0.147527338	down
ASMM9PARTA024275	Pax4	0.002149344	0.451847473	down
ASMM9PARTA030247	H2-Ea-ps	0.00000373	0.180102989	down
ASMM9PARTA037378	4930588N13Rik	0.009200743	0.471459115	down
ASMM9PARTA022755	Dab2	0.000215	0.319370824	down
ASMM9PARTA038317	2310034C09Rik	0.02548631	0.456629845	down
ASMM9PARTA025240	Zfp605	0.000196	0.060955598	down
ASMM9PARTA040940	Olfr520	0.010135816	0.32534108	down
ASMM9PARTA032061	Casp9	0.0000204	0.1676868	down
ASMM9PARTA022266	D430042O09Rik	0.00000298	0.461460503	down
ASMM9PARTA028071	Il12rb1	0.000154	0.47967136	down
ASMM9PARTA022667	Gm595	0.039595358	0.302110304	down
ASMM9PARTA025578	Ucma	0.002863975	0.476993415	down
ASMM9PARTA025137	Telo2	0.03299269	0.452947025	down
ASMM9PARTA022728	Kif19a	0.012253807	0.475640025	down
ASMM9PARTA042724	Fam100b	0.00000504	0.396488775	down
ASMM9PARTA034950	Tmem35	0.037913263	0.393186827	down
ASMM9PARTA027104	Aatk	0.0000275	0.452087332	down
ASMM9PARTA033308	Egr4	0.00000177	0.285297946	down
ASMM9PARTA041571	Mtrr	0.03828319	0.440571818	down
ASMM9PARTA031895	Dkkl1	0.000023	0.483600619	down
ASMM9PARTA025598	Plekha8	0.0000181	0.494882862	down
ASMM9PARTA023494	Wtap	4.04E-09	0.031462811	down
ASMM9PARTA040967	BC031353	0.002297535	0.40781426	down
ASMM9PARTA022055	Btbd16	0.001195442	0.495289647	down
ASMM9PARTA027168	Pld5	0.042666536	0.484051706	down
ASMM9PARTA044159	Zfp516	0.00000884	0.489752948	down
ASMM9PARTA041630	Ppwd1	0.0000512	0.285697299	down
ASMM9PARTA042640	Mael	0.0000521	0.366272688	down
ASMM9PARTA027349	LOC100048884	0.0000231	0.248135424	down
ASMM9PARTA037449	Sucnr1	0.029970655	0.485580045	down
ASMM9PARTA031728	S100a8	0.001085106	0.487720538	down
ASMM9PARTA037057	Fam164b	0.024428518	0.444966974	down
ASMM9PARTA044875	Rxfp1	0.004049994	0.425257181	down
ASMM9PARTA028264	Pcnt	0.042218003	0.496031746	down
ASMM9PARTA027595	Foxd4	0.000676	0.221990168	down
ASMM9PARTA029861	Nr4a1	0.0000345	0.2396998	down
ASMM9PARTA035817	Wdr96	0.04961447	0.457189945	down
ASMM9PARTA029595	Fgf8	0.003596607	0.428435302	down
ASMM9PARTA039413	Cdca7l	0.010998045	0.247353381	down
ASMM9PARTA039430	Tbx22	0.003016145	0.480964623	down

ASMM9PARTA038183	Nxph3	0.002594362	0.497930105	down
ASMM9PARTA030053	Fcnb	0.049639612	0.45100775	down
ASMM9PARTA021821	Med1	0.000000712	0.043025313	down
ASMM9PARTA030909	S100a5	0.00000941	0.007860551	down
ASMM9PARTA023536	Dlgap1	0.010963332	0.487633326	down
ASMM9PARTA039649	Uts2r	0.000000867	0.197836989	down
ASMM9PARTA040512	Olfr143	0.029612243	0.498301986	down
ASMM9PARTA026827	Pde4b	0.006547238	0.488341196	down
ASMM9PARTA038735	Calml4	0.000198	0.439853508	down
ASMM9PARTA021440	Baiap2	0.00000323	0.43205148	down
ASMM9PARTA022090	Olfr367-ps	0.005624322	0.424954591	down
ASMM9PARTA033372	Gprc5b	0.000881	0.416962728	down
ASMM9PARTA021303	Gm904	0.04626398	0.342016915	down
ASMM9PARTA037993	Pcdhb16	0.0000428	0.462512934	down
ASMM9PARTA026985	Aqp1	0.0000856	0.449955904	down
ASMM9PARTA032496	Ear7	0.0000289	0.125511963	down
ASMM9PARTA037782	Supt16h	0.000536	0.371455047	down
ASMM9PARTA034987	1700019N19Rik	0.009215388	0.449747993	down
ASMM9PARTA028172	Klk1b5	0.000153	0.340378951	down
ASMM9PARTA042880	Tmem164	0.0000915	0.450962659	down
ASMM9PARTA027690	Ear2	0.00000814	0.242600267	down
ASMM9PARTA043966	Gmeb2	0.001528723	0.190544024	down
ASMM9PARTA028365	Lmo2	0.00000178	0.445117224	down
ASMM9PARTA037765	Mesdc1	0.00000719	0.136068097	down
ASMM9PARTA021314	Timm8a2	0.007666159	0.42203529	down
ASMM9PARTA029477	Camk4	0.0000144	0.456399786	down
ASMM9PARTA029767	Cxcr2	0.003962452	0.34452615	down
ASMM9PARTA027972	Gzmk	0.00022	0.299988294	down
ASMM9PARTA030501	Klk1b26	0.0000274	0.184984956	down
ASMM9PARTA028309	Hlx	0.015858162	0.423731022	down
ASMM9PARTA043294	3110035E14Rik	0.0000108	0.462555101	down
ASMM9PARTA044092	Zfp811	0.0000987	0.435961258	down
ASMM9PARTA020865	ORF19	0.000103	0.372990769	down
ASMM9PARTA037340	Spaca3	0.011417726	0.406437812	down
ASMM9PARTA024447	Islr2	0.0000502	0.468932297	down
ASMM9PARTA042117	Psmb11	0.020527583	0.470610382	down
ASMM9PARTA044266	Scd4	0.017677516	0.476565011	down
ASMM9PARTA031022	Kdm5d	0.00000987	0.481560959	down
ASMM9PARTA027456	Speg	0.000304	0.482471753	down
ASMM9PARTA028098	Cd79b	0.0000317	0.286383088	down
ASMM9PARTA044088	A530054K11Rik	0.00000732	0.426091364	down
ASMM9PARTA021267	Lrrc8b	0.001054654	0.413018662	down
ASMM9PARTA038130	Ear10	0.000000362	0.214187479	down
ASMM9PARTA030334	Sik1	0.004887472	0.480421872	down
ASMM9PARTA044613	Btbd6	0.0000102	0.399599889	down
ASMM9PARTA025714	Eif5a	0.001025027	0.413191246	down
ASMM9PARTA032436	Irx4	0.0000528	0.49433827	down
ASMM9PARTA027289	Zfp963	0.0000246	0.117660195	down
ASMM9PARTA039168	Defb11	0.0000138	0.275106668	down
ASMM9PARTA031186	Prss3	0.0000798	0.495682998	down
ASMM9PARTA043669	Retnlg	0.000762	0.38681429	down
ASMM9PARTA020937	7420426K07Rik	0.028842604	0.480785873	down
ASMM9PARTA035463	Ypel3	0.000102	0.483375975	down
ASMM9PARTA022526	Neurl1b	0.00000667	0.455872437	down
ASMM9PARTA023996	Il1rap	0.0000404	0.491431571	down
ASMM9PARTA027360	Aldoa	0.0000288	0.451597508	down
ASMM9PARTA027005	Slc7a9	0.000437	0.46457929	down

ASMM9PARTA022081	Nrp2	0.000247	0.499287442	down
ASMM9PARTA025273	Efemp2	0.00000317	0.448102695	down
ASMM9PARTA041885	Lpar1	0.000134	0.255179441	down
ASMM9PARTA042216	D330045A20Rik	0.0000219	0.48107513	down
ASMM9PARTA027673	Fosl2	0.0000218	0.450510965	down
ASMM9PARTA030322	Klk1b24	0.0000703	0.271431027	down
ASMM9PARTA040975	Olfr585	0.000681	0.455877238	down
ASMM9PARTA038351	Hsd17b11	0.000105	0.437128239	down
ASMM9PARTA041331	BC026439	0.006384262	0.482822713	down
ASMM9PARTA037597	Aldh1a3	0.001617957	0.394004916	down
ASMM9PARTA035120	Ttc14	0.000049	0.367692344	down
ASMM9PARTA033718	Zfp110	0.018406352	0.476445261	down
ASMM9PARTA025886	Hmga1	0.000188	0.489959433	down
ASMM9PARTA034368	Klhl10	0.037427932	0.470546694	down
ASMM9PARTA027435	Cort	0.00000844	0.468392017	down
ASMM9PARTA041911	Rspo2	0.000119	0.493041216	down
ASMM9PARTA041918	1110012J17Rik	0.0000798	0.498240787	down
ASMM9PARTA028187	Ncoa2	0.0000767	0.408131281	down
ASMM9PARTA022239	Apobec4	0.006100148	0.392494963	down
ASMM9PARTA045010	Mrgprb8	0.000346	0.349066518	down
ASMM9PARTA036919	4930563D23Rik	0.005648275	0.387879931	down
ASMM9PARTA033587	Syne1	0.049603894	0.46259368	down
ASMM9PARTA033242	Ucn	0.0000268	0.246108926	down
ASMM9PARTA022906	Apold1	0.000084	0.382631851	down
ASMM9PARTA033534	Pvrl3	0.02094496	0.481103991	down
ASMM9PARTA042913	Dmrt3	0.0000596	0.440267751	down
ASMM9PARTA021920	Fam65c	0.005946101	0.355848965	down
ASMM9PARTA027446	Cux2	0.00000714	0.494651629	down
ASMM9PARTA027162	Cckbr	0.000157	0.485405389	down
ASMM9PARTA038507	Pigt	0.0000156	0.283801069	down
ASMM9PARTA031550	Casp8ap2	0.0000469	0.362896029	down
ASMM9PARTA030791	Furin	0.00000359	0.475618715	down
ASMM9PARTA041030	Cpz	0.000136	0.43524052	down
ASMM9PARTA043502	Rnf24	0.03757431	0.409359983	down
ASMM9PARTA027477	Cr2	0.000399	0.122298592	down
ASMM9PARTA037189	Ston1	0.000000392	0.071503707	down
ASMM9PARTA030278	Foxd3	0.002113074	0.318211833	down
ASMM9PARTA027730	Mpp3	0.0000418	0.423920224	down
ASMM9PARTA023550	BC048609	0.017351313	0.425830757	down
ASMM9PARTA035223	1110059M19Rik	0.0000611	0.449016974	down
