# Supplementary material for: Microarray Profile of Long Noncoding RNA and Messenger RNA Expression in a Model of Alzheimer’s Disease
Source: Life (Basel). 2020 May 14;10(5):64. doi: 10.3390/life10050064 (PMC7281340; doi:10.3390/life10050064)
Supplement: Supplementary file 1 [file life-10-00064-s001.zip › life-787240-supplemenatry-to be published - PDF/life-787240-supplementary/Table S9.pdf]

life

Supplementary

Microarray Profile of Long Noncoding RNA and Messenger RNA Expression in a Model of Alzheimer's Disease

Linlin Wang †, Li Zeng †, Hailun Jiang, Zhuorong Li * and Rui Liu *

Institute of Medicinal Biotechnology, Chinese Academy of Medical Sciences and Peking Union Medical College, Beijing 100050, China; wanglinlin@wfmc.edu.cn (L.W.); zengsheng@imb.pumc.edu.cn (L.Z.); jianghailun@imb.pumc.edu.cn (H.J.)

* Correspondence: lizhourong@imb.pumc.edu.cn (Z.L.); +86-10-8352017; .liurui@imb.pumc.edu.cn (R.L.); Tel.: +86-10-67087731

Table S9. Top 10 aberrantly expressed lncRNAs in 1-month-old, 3-month-old, 6-month-old, and 9-month-old APP/PS1 mice as compared to age-matched WT control mice.

	lncRNA Name	*p*-Value	Fold-Change	Regulation
1-month-old APP/PS1 mice versus age-matched WT mice	mouselincRNA0297	0.03853617	2.4391737	up
	AL732590.2	0.002783807	2.15185	up
	A230108P19Rik	0.001155623	2.6593003	up
	mouselincRNA0294	0.024343088	2.5895295	up
	mouselincRNA0737	0.001355704	3.430497	up
	humanlincRNA1590	0.0000437	5.9778385	up
	NRON.1	0.003656608	3.9517019	up
	humanlincRNA2213	0.017903363	2.0292795	up
	mouselincRNA0380	0.001012912	2.3298893	up
	Gm16706	0.003379951	3.3808007	up
	AK039487	0.001401456	0.362343036	down
	Mogat1	0.007517785	0.404617429	down
	D230002A01Rik	0.005323117	0.342491804	down
	4933436C20Rik	0.000151	0.413698244	down
	Gm4759	0.027513668	0.462699567	down
	AK015813	0.00000139	0.451870259	down
	A530058N18Rik	0.000441	0.427147769	down
	AK017111	0.000132	0.337344016	down
	AK018924	0.030719675	0.416587029	down
	AK050516	0.0000168	0.490624124	down
3-month-old APP/PS1 mice versus age-matched WT mice	BC075635	0.035933297	2.031355	up
	4933434I20Rik	0.009978537	2.0194137	up
	humanlincRNA1040	0.040220477	2.0302415	up
	mouselincRNA1286	0.002579583	2.456823	up
	Gm12940	0.0000735	3.5512953	up
	mouselincRNA0294	0.018257162	2.6640735	up
	humanlincRNA1590	0.0000642	4.6550817	up
	AK039862	0.000282	10.566481	up
	4632427E13Rik	0.003185133	2.1290658	up

	Gm16706	0.028662696	2.2541282	up
	AK039125	0.04244341	0.412248327	down
	humanlincRNA0676	0.01711409	0.495437516	down
	mouselincRNA1108	0.024073955	0.389109585	down
	mouselincRNA1426	0.000292	0.288348306	down
	mouselincRNA1009	0.031525224	0.468723422	down
	AK032255	0.007611876	0.339942518	down
	2410057H14Rik	0.006866116	0.499634767	down
	4930448N21Rik	0.002114768	0.425981788	down
	BC079904	0.02010789	0.43873808	down
	mouselincRNA0637	0.014044236	0.456293078	down
6-month-old APP/PS1 mice versus age-matched WT mice	Abhd1	0.000000243	3.1196003	up
	mouselincRNA1286	0.003538504	2.2654934	up
	mouselincRNA1524	0.006277942	2.1662855	up
	4933436C20Rik	0.000129	2.420025	up
	A230108P19Rik	0.021290345	2.4069388	up
	mouselincRNA0737	0.004286149	2.6884916	up
	Gm11019	0.025719354	2.0346353	up
	humanlincRNA1590	0.023626897	3.5407078	up
	4732418A04Rik	0.000194	4.9253507	up
	AK039862	0.001835104	4.3281155	up
	Gm10497	0.000034	0.399063366	down
	Gm10497	0.000034	0.399063366	down
	Gm10497	0.000034	0.399063366	down
	AK042016	0.000569	0.43446526	down
	TCR-beta chain	0.00000429	0.292112979	down
	AK017111	0.000589	0.281338745	down
	AK039014	0.025748411	0.39605576	down
	Gm12092	0.009861143	0.450306819	down
	uc.197	0.000323	0.435374605	down
	3110099E03Rik	0.002467179	0.226432662	down
9-month-old APP/PS1 mice versus age-matched WT mice	Gm16834	0.0000174	2.4332428	up
	Gm16836	0.0000959	2.4933932	up
	mouselincRNA1286	0.003183203	2.4396448	up
	Gm14158	0.000285	2.3584278	up
	mouselincRNA1524	0.00046	2.0816717	up
	Gm15086	0.020345824	2.7550943	up
	Gm12940	0.000724	2.2870247	up
	mouselincRNA0155	0.040218018	2.235328	up
	mouselincRNA0737	0.018313346	2.563409	up
	AK144771	0.000183	2.120443	up
	Gm4759	0.011067896	0.275980455	down
	mouselincRNA0004	0.000337	0.449832802	down
	Gm16206	0.017974988	0.383945252	down
	F630042J09Rik	0.02230519	0.484179156	down
	humanlincRNA2085	0.00000106	0.345139247	down
	mouselincRNA1108	0.013267624	0.342984529	down
	Gm11508	0.00000196	0.251871925	down
	AK017111	0.0000171	0.240110278	down
	AK039014	0.017692	0.436916719	down
	D12Ertd551e	0.00432953	0.488454045	down
